# Discovery and molecular basis of subtype-selective cyclophilin inhibitors

**DOI:** 10.1038/s41589-022-01116-1

**Published:** 2022-09-26

**Authors:** Alexander A. Peterson, Aziz M. Rangwala, Manish K. Thakur, Patrick S. Ward, Christie Hung, Ian R. Outhwaite, Alix I. Chan, Dmitry L. Usanov, Vamsi K. Mootha, Markus A. Seeliger, David R. Liu

**Affiliations:** 1 Merkin Institute of Transformative Technologies in Healthcare, Broad Institute of MIT and Harvard, Cambridge, MA USA; 2 Department of Chemistry and Chemical Biology, Harvard University, Cambridge, MA USA; 3 Howard Hughes Medical Institute, Harvard University, Cambridge, MA USA; 4 Department of Pharmacological Sciences, Stony Brook University, Stony Brook, NY USA; 5 Broad Institute of MIT and Harvard, Cambridge, MA USA; 6 Howard Hughes Medical Institute and Departments of Molecular Biology and Medicine, Massachusetts General Hospital, Boston, MA USA; 7 Department of Systems Biology, Harvard Medical School, Boston, MA USA

**Keywords:** Chemical biology, Combinatorial libraries

## Abstract

Although cyclophilins are attractive targets for probing biology and therapeutic intervention, no subtype-selective cyclophilin inhibitors have been described. We discovered novel cyclophilin inhibitors from the in vitro selection of a DNA-templated library of 256,000 drug-like macrocycles for cyclophilin D (CypD) affinity. Iterated macrocycle engineering guided by ten X-ray co-crystal structures yielded potent and selective inhibitors (half maximal inhibitory concentration (IC_50_) = 10 nM) that bind the active site of CypD and also make novel interactions with non-conserved residues in the S2 pocket, an adjacent exo-site. The resulting macrocycles inhibit CypD activity with 21- to >10,000-fold selectivity over other cyclophilins and inhibit mitochondrial permeability transition pore opening in isolated mitochondria. We further exploited S2 pocket interactions to develop the first cyclophilin E (CypE)-selective inhibitor, which forms a reversible covalent bond with a CypE S2 pocket lysine, and exhibits 30- to >4,000-fold selectivity over other cyclophilins. These findings reveal a strategy to generate isoform-selective small-molecule cyclophilin modulators, advancing their suitability as targets for biological investigation and therapeutic development.

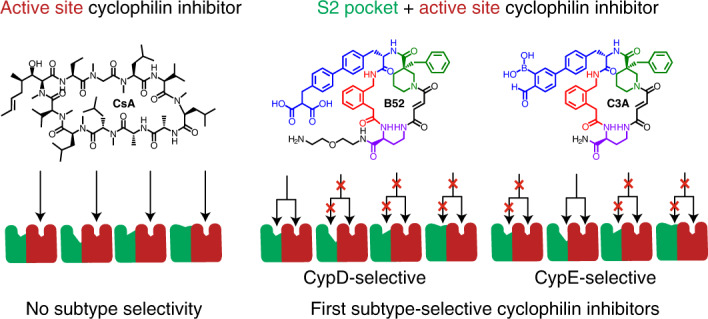

## Abstract (web summary)

DNA-templated compound library screening and structure-guided hit optimization resulted in the identification of selective macrocyclic inhibitors of cyclophilin isoforms CypD and CypE.

## Main

The human cyclophilin family consists of 17 proteins containing a structurally conserved peptidyl-prolyl-isomerase (PPIase) domain^1^. At least 12 members of this family catalyze the *cis*-*trans* isomerization of peptidyl–proline bonds^1,2^, a rate-limiting step in the folding of many proteins^3,4^. Cyclophilin D (CypD) is unique as a mitochondrial cyclophilin^1,3^ and a key regulator of the mitochondrial permeability transition pore (mPTP), a transient channel on the inner mitochondrial membrane that opens under oxidative stress or high mitochondrial matrix calcium levels^5–8^. Inhibition^5,6,8,9^ or knockout^5–8^ of CypD causes the mPTP to be more resistant to pore opening events. Prolonged mPTP opening causes osmotic imbalance, mitochondrial swelling and rupture, and cell death^7,8,10^. This pathway has been implicated in a variety of conditions associated with oxidative stress, including ischemia-reperfusion injury (IRI)^8,11^, many neurodegenerative disorders^12–18^, liver diseases^19^, aging and autophagy^20,21^, and diabetes^22^. Inhibition of CypD is thus considered a potential therapeutic strategy for IRI^7,8^, Alzheimer’s disease^13^, Huntington’s disease^14^, multiple sclerosis^16^, Parkinson’s disease^18^, amyotrophic lateral sclerosis^12^, X-linked adrenoleukodystrophy^17^, liver cirrhosis^19^, and diabetes-related diseases^22^. While the exact structure^23–25^, function^5,10,20^, and regulatory pathways^5,10^ of the mPTP are still debated, CypD is a well-established regulator of mPTP function. CypD inhibitors could therefore also illuminate other protein regulators and structural components of the mPTP.

While inhibiting CypD is attractive for studying the mPTP and for treating conditions linked to oxidative stress, the 16 other known cyclophilin isoforms play diverse and important biological roles^3,4,26–29^. Cyclophilin subtype selectivity is therefore critical for biological probes and for potential therapeutics to minimize unwanted side-effects from off-target cyclophilin perturbation. To our knowledge, no subtype-selective cyclophilin inhibitors have been described for any cyclophilin isoforms^1,3^. Efforts to develop selective cyclophilin inhibitors are stymied by the high sequence identity (61–86%) and highly conserved structures of human PPIase domains^1^.

**Extended Data Fig. 1:**
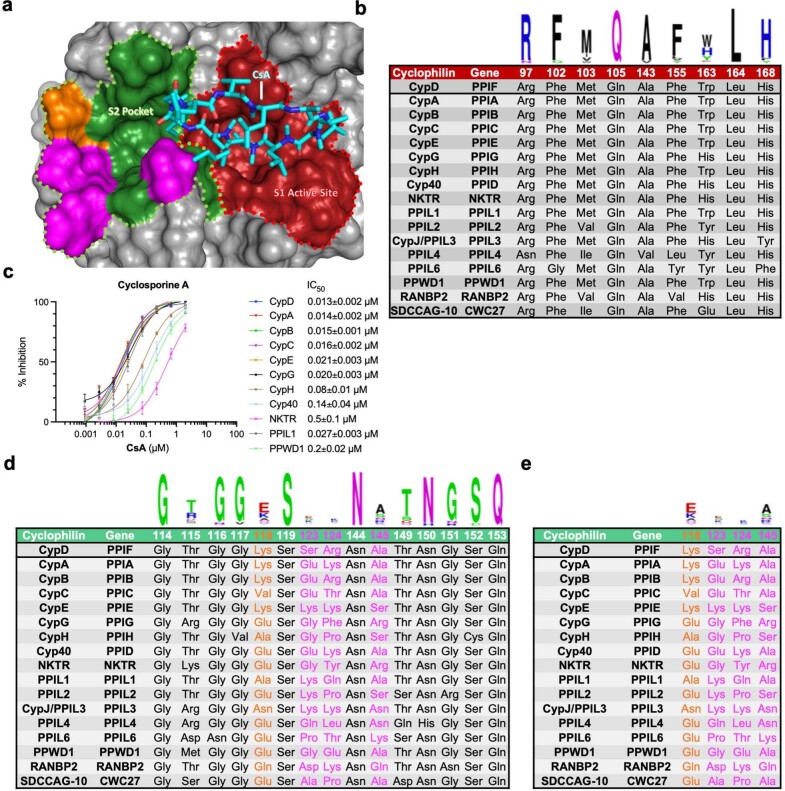
Exclusive active site binding results in promiscuous cyclophilin inhibition. **a**, Co-crystal structure of CsA bound to CypD (PDB ID 2Z6W). CsA (cyan) binds to the active site (red) of CypD, a binding mode that does not engage more diversified residues that lie in the S2 pocket (green), including the primary gatekeepers (magenta) and the semiconserved K118 residue (orange). **b**, Active site (S1 pocket) residues with WebLogo plots, showing high conservation between the 17 cyclophilin isoforms with corresponding gene and protein identifier. **c**, Isomerization of the reporter peptide Suc-AAPF-AMC was measured with 5 nM cyclophilin and varying concentrations of CsA. CsA shows potent, but promiscuous inhibitory activity against all prolyl isomerase-active cyclophilins. Selective inhibition could rather be achieved through interactions with S2 pocket residues of cyclophilin subtypes (**d**). **e**, Cyclophilin S2 pocket residues that are the primary diversification sites within the active site-S2 pocket groove with WebLogo plots. Typical cyclophilin inhibitors such as CsA do not engage in interactions with these residues. All residue numbering is in reference to CypD. IC_50_ values reflect mean ± s.e.m. of three technical replicates. Data points and error bars reflect mean ± s.d. of individual assays at one dose. Source data

A key structural feature shared among all cyclophilin isoforms is a shallow and highly conserved PPIase active site^1,30^ (Extended Data Fig. 1a,b). Classic cyclophilin inhibitors such as cyclosporine A (CsA) bind to this active site^1,30^ and thus equipotently inhibit most cyclophilins (Extended Data Fig. 1a,c and Supplementary Fig. 1). Adjacent to the active site is the S2 pocket, a distinct exo-site that forms part of a long substrate-binding groove for peptides (Extended Data Fig. 1a). S2 pocket residues are much more diverse than active site residues among cyclophilins, providing an opportunity for isoform-selective binding^1^ (Extended Data Fig. 1d). In particular, three gatekeeper residues (positions 123, 124, and 145 in CypD) and one far S2 pocket residue (position 118 in CypD) are highly diverse among cyclophilins (Extended Data Fig. 1e). While S2 pocket binding has been recognized as a strategy to achieve isoform-selective inhibition^1^, current small-molecule cyclophilin inhibitors when profiled show poor isoform selectivity and are not known to exploit interactions with non-conserved S2 exo-site residues^3,30–37^.

In this study we report the development of novel cyclophilin inhibitors from a DNA-templated macrocycle library and iterated structural biology and small-molecule engineering, yielding potent and isoform-selective CypD and CypE inhibitors. These findings establish a strategy for the development of subtype-selective cyclophilin modulators through engineering interactions with unique S2 pocket exo-site residues.

## Results

### Selection of a library of 256,000 DNA-templated macrocycles

**Fig. 1:**
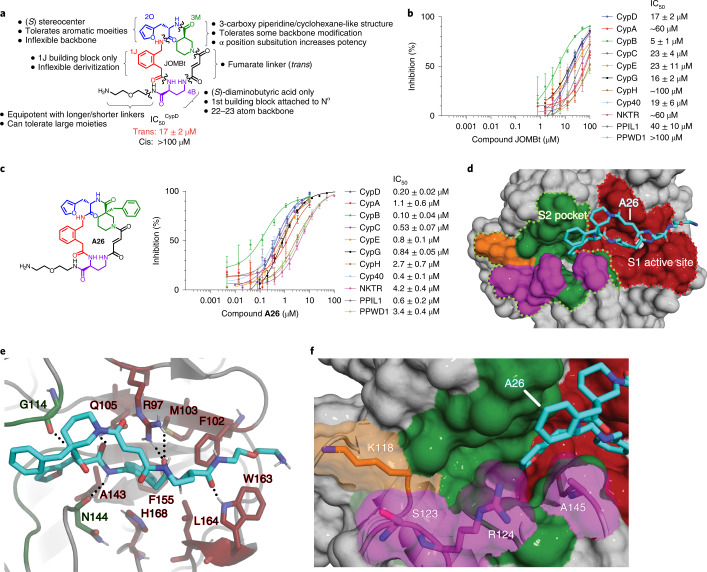
Selection of a DNA-templated library of 256,000 macrocycles for CypD affinity reveals novel cyclophilin inhibitors. **a**, Generalized trends in inhibition potency of CypD prolyl-isomerase activity from cyclophilin inhibition profiles of library hits and **A**-series macrocycles. **b**, JOMBt, showing weak and promiscuous cyclophilin inhibition of prolyl-isomerase activity. **c**, Compound **A26** showing improved CypD potency but promiscuous inhibition. **d**, X-ray co-crystal structure of compound **A26** (cyan) bound to CypD (PDB ID 7TGT, 1.06 Å resolution), shown as a space-filling model. **A26** has a dual-binding mode involving the active site (red) and S2 pocket (green) of CypD. **e**, Active site binding interactions with **A26**. The phenyl group provides the primary hydrophobic interactions with F102, M103, A143, F155, L164, and H168. Black dashes show predicted hydrogen bonding interactions with R97, Q105, G114, N144, and W163 and the backbone of the **A26**. **f**, S2 pocket binding pose of the furan of **A26**, exhibiting a shallow interaction that does not engage non-conserved residues K118 (orange), S123 (magenta), and R124 (magenta) on the far side of the pocket. IC_50_ values reflect mean ± s.e.m. of three technical replicates. Data points and error bars reflect mean ± s.d. of individual assays at one dose. Source data

**Extended Data Fig. 2:**
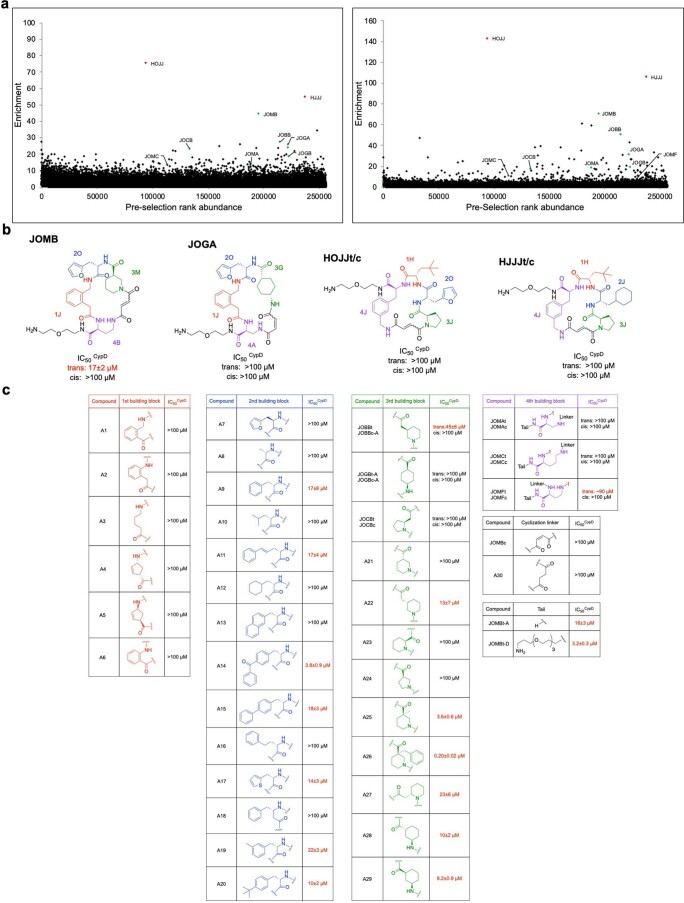
Overview of macrocycle library hits from the CypD binding selection and JOMBt structure-activity relationships. **a**, Two replicates of this selection yielded consistent enrichment of the JO** family of macrocycles. Library hits re-synthesized in DNA-free form resulted in novel CypD inhibitors JOMBt and JOBBt, the starting points of CypD inhibitors developed in this study. **b**, Structure JOMB, JOGA, and H*JJ macrocycles that enriched well in the DNA-templated macrocycle library selection. JOMB in the trans isomer (JOMBt) exhibits weak prolyl isomerase inhibition. **c**, Library hits and derivatives of JOMBt and their corresponding prolyl isomerase inhibition IC_50_ values for CypD. Each listed compound has the JOMBt structure except for the drawn building block substitution. Note, library encoded macrocycles that denote an ‘-A’ nomenclature (JOGBt-A, JOGBc-A, JOBBc-A) include the same primary amide tail shown in JOMBt-A. Values and error bars reflect mean ± s.e.m. of three technical replicates. Source data

To identify new cyclophilin inhibitors in an unbiased manner, we performed the selection of a DNA-templated library of 256,000 macrocycles^38^ for binding immobilized CypD. Analysis of selection data revealed a highly conserved group of putative cyclophilin binders: the JO** family, where each letter identifies a library building block at one of the four macrocycle positions, and * indicates broad structural variation (Extended Data Fig. 2a). A set of highly enriched macrocycles was synthesized in DNA-free form as both the *cis* (ending in **c**) and *trans* (ending in **t**) olefin isomers, then assayed for their ability to inhibit cyclophilin prolyl isomerization on a peptide substrate (succinate-AAPF-AMC). Two library hits show modest inhibition of CypD prolyl-isomerase activity, JOMBt (half maximal inhibitory concentration ($\mathrm{{IC}^{CypD}_{50}}$) = 17 μM) and JOBBt ($\mathrm{{IC}^{CypD}_{50}}$ = 45 μM) (Fig. 1a and Extended Data Fig. 2b,c). These results were verified by surface plasmon resonance, which yielded dissociation constants of: JOMBt $K^{CypD}_{\mathrm{D}}$ = 19 μM and JOBBt $K^{CypD}_{\mathrm{D}}$ = 45 μM (Supplementary Fig. 2a–c). Other strongly enriched hits from this selection include HOJJt/c and HJJJt/c, which show no prolyl-isomerase inhibition for either isomer (Extended Data Fig. 2b) and may bind CypD without inhibiting PPI activity.

We used JOMBt as a scaffold to reveal structure–activity relationships and improve potency against CypD (Fig. 1a and Extended Data Fig. 2c; **A** series). The synthesis and evaluation of 48 JOMBt analogs revealed several key insights, including a highly conserved first-position 1J building block (**A1**–**A6**), stereochemistry and an aromatic moiety requirement at the second building block (**A7**–**A20**), certain piperidine cyclohexane structures with specific connectivity and stereochemistry at the third building block (**A21**–**A29**, JOGBt/c-A, JOCBt/c-A, JOGAt/c), preferred diaminobutyric acid derivatives at the fourth building block (JOMAt/c, JOMCt/c, JOMFt/c, JOGAt/c), required fumarate-like macrocycle linker (**A30**, JOMBc, JOBBc), and no preference for a series of tail moieties (JOMBtA, JOMBtD) (Fig. 1a and Extended Data Fig. 2c).

Adding a benzyl group to the tertiary carbon in the piperidine ring of JOMBt (resulting in **A26**) increases CypD potency 85-fold to $\mathrm{{IC}^{CypD}_{50}}$ = 0.20 μM (Extended Data Fig. 2c). We assayed compounds against a full panel of 11 cyclophilins with prolyl-isomerase activity (Supplementary Fig. 3a) to assess subtype selectivity. JOMBt and **A26** show promiscuous cyclophilin inhibition (Fig. 1b,c and Supplementary Fig. 4a,c), similar to that of CsA (Extended Data Fig. 1c).

### Co-crystal structures reveal inhibitor binding mode

**Extended Data Fig. 3:**
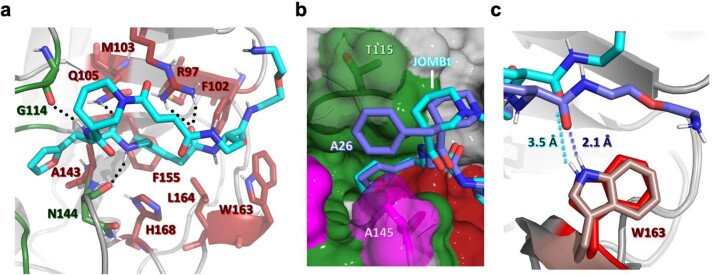
X-ray co-crystal structure of JOMBt:CypD compared to A26:CypD. **a**, Active site binding pose of JOMBt (PDB ID 7TGS, 1.75 Å resolution) showing almost identical interactions with CypD as **A26** (Fig. 1d, e). Black dashes indicate predicted hydrogen bonds. Notable differences between the binding mode of JOMBt and **A26** include **b**, An extra hydrophobic contact between the benzyl group of **A26** (purple) and T115 (green) is absent with JOMBt (cyan). **c**, An extra hydrogen bond between **A26** (purple) and W163 (salmon or red) that JOMBt (cyan) lacks. The W163–JOMBt distance is 3.5 Å (cyan dashes), compared to 2.1 Å for W163–**A26** (purple dashes). Van der Waals surfaces of T115 and A145 (magenta) are shown in the structure with **A26**.

To illuminate the molecular basis of CypD inhibition by JOMBt and **A26** we solved X-ray co-crystal structures of each compound bound to CypD (Fig. 1d–f and Extended Data Fig. 3a–c, Protein Data Bank (PDB) IDs 7TGS, 7TGT). Both compounds share a similar binding mode, with the phenyl ring of the 1J building block anchoring the macrocycle in the hydrophobic pocket of the active site, supplemented with key hydrogen bonding interactions between the macrocycle backbone and active site residues (Fig. 1d,e and Extended Data Fig. 3a). **A26** engages CypD more extensively than JOMBt. The benzyl group of **A26** engages in hydrophobic contacts with T115 (Extended Data Fig. 3b), while the macrocycle backbone forms a closer hydrogen bond with the side-chain N–H on W163 (Extended Data Fig. 3c). While this benzyl group increases potency, it does not influence CypD selectivity, likely owing to hydrophobic contacts with semi-conserved S2 pocket residue T115 in other cyclophilins (Extended Data Fig. 1d). Indeed, replacing the benzyl group with –H (JOMBt) or –Me (**A25**) resulted in almost identical selectivity profiles (Supplementary Fig. 4a–c).

Both crystal structures revealed very shallow placement of the furan building block just inside the S2 pocket (Fig. 1f and Extended Data Fig. 3a,b). Given our hypothesis that S2 pocket engagement could yield subtype selectivity, we reasoned that replacing this furan with other groups might increase CypD selectivity. Consistent with this possibility, macrocycle **A14**, in which the furan of JOMBt is replaced with a benzophenone, shows increased selectivity over CypG, CypH, and NKTR (Supplementary Fig. 4d).

### S2 pocket ligands influence CypD selectivity and potency

**Fig. 2:**
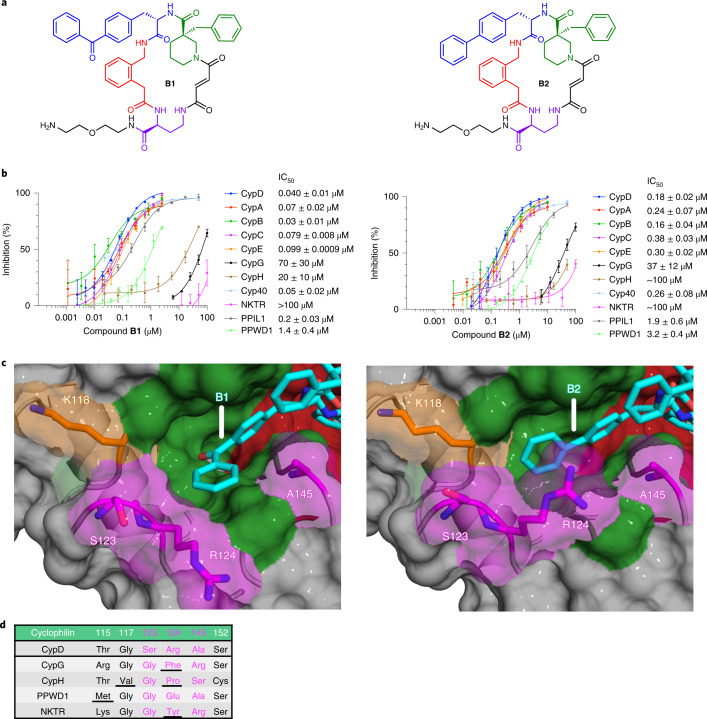
Compounds with large S2 pocket binding groups show reduced potency against cyclophilins with sterically occluded S2 pockets. **a**, Structure of **B1**, **B2**. **b**, Cyclophilin prolyl-isomerase inhibition screens for **B1** and **B2**. **c**, Co-crystal structures of **B1** (PDB ID 7TGU, 1.21 Å resolution) or **B2** (PDB ID 7TGV, 1.46 Å resolution) bound to CypD, viewing the S2 pocket. Active site binding is identical to that of **A26** (Fig. 1e). **d**, Residues within the S2 pocket of cyclophilins inhibited less potently by **B1** and **B2**, with important residues underlined. The benzophenone or biphenyl group of **B1** or **B2**, respectively, fills the S2 pocket more completely, resulting in selectivity over cyclophilins with more sterically occluded or inflexible S2 pockets. CypD by contrast contains a relatively un-occluded S2 pocket and flexible R124 residue. IC_50_ values reflect mean ± s.e.m. of three technical replicates. Data points and error bars reflect mean ± s.d. of individual assays at one dose. Source data

Encouraged by the above results, we synthesized a new series of macrocycles (**B** series) using **A26** as a scaffold, varying potential S2-binding groups to increase CypD selectivity. Large hydrophobic groups that replace the furan in **A26**, such as benzophenone **B1** ($\mathrm{{IC}^{CypD}_{50}}$ = 0.040 μM) or biphenyl **B2** ($\mathrm{{IC}^{CypD}_{50}}$ = 0.18 μM), retain CypD potency, but no longer potently inhibit NKTR, CypH, or CypG, with some selectivity over PPWD1 (Fig. 2a,b and Supplementary Fig. 5a,b). The S2 pockets in these cyclophilins are more sterically occluded or rigid than in the other seven tested cyclophilins, likely clashing with large hydrophobic moieties in **B1** and **B2** (Fig. 2d). To gain further insight into the basis of emerging CypD subtype selectivity, we solved co-crystal structures of **B1**, **B2**, and a smaller derivative **B3** bound to CypD (Fig. 2c and Supplementary Fig. 5c; PDB IDs 7TGU, 7TGV, 7TH1). These structures revealed deeper S2 pocket binding by the benzophenone and biphenyl groups of **B1** and **B2**, respectively, while retaining the original active site interactions of the parent compound **A26**. We also observed migration of the R124 side-chain contingent on the size of the S2-binding moiety. As the ligand grew in size from **B3** → **B2** → **B1**, the R124 side-chain gradually moved from tucked in the pocket to fully solvent-exposed (Fig. 2c and Supplementary Fig. 5c).

These observations suggest the need to displace some residues within the S2 pocket during ligand binding to achieve partial CypD subtype selectivity. While the polarity and hydrophilicity of R124 in CypD minimizes the energetic cost of doing so, CypG, CypH, NKTR, and PPWD1 contain large hydrophobic groups or inflexible residues buried in the S2 pocket, and moving their side-chains out of the pocket would likely incur a large energetic penalty (Fig. 2d). This observation was supported by additional macrocycles for which the largest, most hydrophobic derivatives (such as **B5**) are more selective for CypD than smaller or more hydrophilic analogs (**B4**, **B6**, **B7**) (Supplementary Fig. 6a–d).

To further improve selectivity for CypD, we diversified the biphenyl ring of **B2**, which was positioned in our co-crystal structures near K118, S123 and R124 within CypD’s S2 pocket. We reasoned that establishing favorable interactions with these residues might further improve subtype selectivity. While derivatives that contain various alkyl, alkoxy, phenols, or heterocycles at varying positions on the distal ring of the biphenyl (**B8**–**B14**) provide no additional benefit, these biphenyl derivatives maintain the same selectivity profile as **B2** (Supplementary Fig. 7a–g). We also observed that *para*-carbonyl moieties, such as **B15** ($\mathrm{{IC}^{CypD}_{50}}$ = 0.038 μM), show promising potency with a preference of 2- to 20-fold for CypD over CypC and Cyp40 (**B15**–**B19**; Supplementary Fig. 8a–e). Replacement of the acetyl group of **B15** with a carboxylate resulted in a tenfold decrease in CypD potency (**B20**, $\mathrm{{IC}^{CypD}_{50}}$ = 0.3 μM; Supplementary Fig. 9a) but drastically improved selectivity over CypC and Cyp40 to at least ninefold. This trend was also observed in a closely related analog (**B21**, $\mathrm{{IC}^{CypD}_{50}}$ = 0.17 μM; Supplementary Fig. 9b; PDB ID 7TH6).

**Fig. 3:**
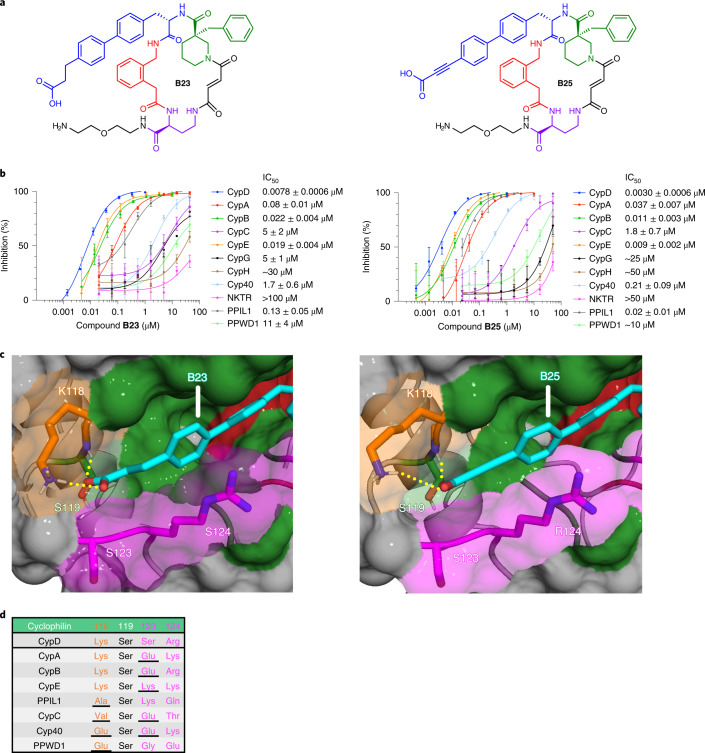
Carboxylate-containing biphenyl derivatives of B2 offer enhanced CypD selectivity. **a**, Structure of **B23** and **B25**. **b**, Cyclophilin prolyl-isomerase inhibition screens for **B23** and **B25. c**, Co-crystal structures of **B23** (PDB ID 7TH7, 1.18 Å resolution) and **B25** (PDB ID 7THC, 1.57 Å resolution) bound to CypD, viewing the S2 pocket. Active site binding is identical to that of **A26** (Fig. 1e). Yellow dashes indicate predicted hydrogen bonds. **d**, List of residues on the far side of the S2 pocket of cyclophilins that are proximal to the ligand carboxylates, with important deleterious interactions underlined. The biphenyl group with 3-carbon carboxylates, **B23** and **B25**, achieve strong selectivity over cyclophilins without a residue homologous to CypD K118. **B23** and **B25** show similar inhibition potencies for CypD and other cyclophilins that contain a Lys residue homologous to CypD K118, but slightly attenuated depending on the identity of residue 123. IC_50_ values reflect mean ± s.e.m. of three technical replicates. Data points and error bars reflect mean ± s.d. of individual assays at one dose. Source data

Potency against CypD was rescued upon distancing the carboxylate from the biphenyl group by one or two carbons (**B22**, $\mathrm{{IC}^{CypD}_{50}}$ = 0.04 μM; B23, $\mathrm{{IC}^{CypD}_{50}}$ = 0.0078 μM; Fig. 3a,b and Supplementary Fig. 9c,d). **B23** shows a dramatic improvement in subtype selectivity, with >500-fold selectivity over CypC, Cyp40, PPWD1, CypH, CypG, NKTR, 25- to 40-fold selectivity over CypA and PPIL1, and ~3-fold selectivity over CypE and CypB. Changing the carboxylate linker to a vinyl group (**B24**, $\mathrm{{IC}^{CypD}_{50}}$ = 0.0019 μM; Supplementary Fig. 9e) or ethynyl group (**B25**, $\mathrm{{IC}^{CypD}_{50}}$ = 0.0030 μM; Fig. 3a,b and Supplementary Fig. 9f) results in potency and selectivity profiles that are similar to **B23**. The selectivity and potency of these carboxylates are attenuated upon migrating the position of the carboxylate (**B26**, **B27**; Supplementary Fig. 10a,b), or masking the negative charge of the carboxylate through an ester or nitro group (**B28**–**B30**) (Supplementary Fig. 10c–e). Replacing the carboxylate with an amine results in a 500- to 1,000-fold reduction in potency for CypD, and a changed selectivity profile that favors PPWD1 and CypC (**B31**, **B32**) (Supplementary Fig. 11a, b). Nitriles **B33** and **B34** also exhibit a similar linker-length-dependent potency profile, with **B34** showing similar potency to **B23**, yet with reduced selectivity (Supplementary Fig. 12a,b). Collectively, these results suggest that carboxylate-containing biphenyl-substituted inhibitors gain CypD potency though a novel interaction that is both charge selective and hydrogen-bond dependent. Moreover, **B23**, **B24**, and **B25** establishes that iterative diversification deeper into the S2 pocket with precisely tailored chemical properties can result in enhanced selectivity for CypD.

### Structural and mutagenesis studies improve CypD selectivity

**Extended Data Fig. 4:**
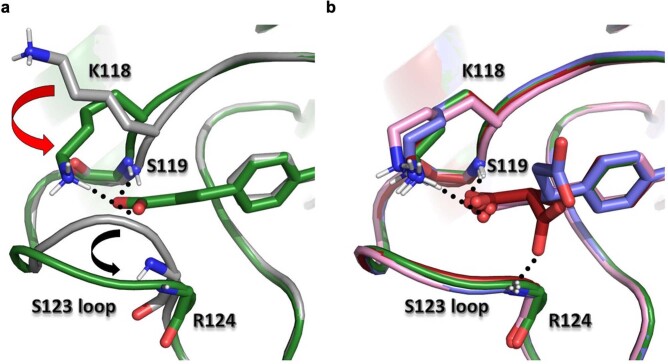
Carboxylate-containing S2-binding moieties induce K118 side-chain and S123 backbone migration. **a**, Superposition of co-crystal structures of CypD with **B2** (gray) and **B23** (green). The side-chain of K118 typically is oriented away from the S2 pocket, as shown in co-crystal structures that do not contain carboxylate ligands, such as that of **B2**. A properly placed carboxylate group (**B23**) migrates K118’s side-chain into the S2 pocket (red arrow), forming a salt bridge along with a hydrogen bond with S119’s peptide backbone (black dashes). Also shown is the S123 loop migration that occurs when carboxylate-containing biphenyl groups bind deep into the S2 pocket (black arrow). **b**, Co-crystal structure superposition of CypD with **B23** (green), **B25** (pink), **B52** (red) and **B53** (purple). All properly placed carboxylate-containing ligands induce the same K118 and S123 conformational change. Dicarboxylates such as **B52** and **B53** also present the second carboxylate towards the S123 gatekeeper residue, the former exhibiting a hydrogen bond (black dashes) with the S123–R124 peptide backbone.

We solved a co-crystal structure of **B23** or **B25** bound to CypD (Fig. 3c; PDB IDs 7TH7 and 7THC). While retaining the same interactions with CypD as **B2**, we also observed a novel hydrogen bond with the backbone of S119 and a salt bridge with the side-chain of K118, which is usually oriented away from the S2 pocket (Extended Data Fig. 4a). The latter interaction explains why cyclophilins that replace the K118 residue with a glutamate (Cyp40, PPWD1), or a hydrophobic residue (CypC, PPIL1) are much less potently inhibited by **B23** and **B25** than cyclophilins that retain lysine at this position (CypD, CypA, CypB, CypE; Fig. 3d). The importance of this interaction was further supported by assaying several prolyl-isomerase-active CypD K118 mutants (Supplementary Fig. 3c). CypD K118A and CypD K118E are less potently inhibited by **B23** and **B25** (**B23**, $\mathrm{{IC}^{CypD(K118A)}_{50}}$ = 0.07 μM, $\mathrm{{IC}^{CypD(K118E)}_{50}}$ = 0.18 μM; **B25**, $\mathrm{{IC}^{CypD(K118A)}_{50}}$ = 0.019 μM, $\mathrm{{IC}^{CypD(K118E)}_{50}}$ = 0.06 μM) than wild-type CypD (**B23**, $\mathrm{{IC}^{CypD}_{50}}$ = 0.0078 μM; **B25**, $\mathrm{{IC}^{CypD}_{50}}$ = 0.003 μM), consistent with the role of a salt bridge with K118 in enhancing CypD potency and selectivity (Supplementary Fig. 13b,c).

These observations refined our model for **B23** binding. Given the proximal placement of the carboxylate of **B23** to S123 and R124, these two residues may also contribute to the partial selectivity of **B23** for CypD. We therefore generated S123E, R124A, and R124K CypD mutants, which retain PPI activity (Supplementary Fig. 3c), and assayed inhibition by **B23** and **B25**. **B23** and **B25** exhibit reduced potency against CypD S123E as compared to wild-type CypD (Supplementary Fig. 14b,c). CypD S123E shares the exact same S2 pocket residues with CypB (Supplementary Fig. 14f), and for both proteins we observe a decrease in IC_50_ of threefold compared with wild-type CypD for **B23** and **B25** (**B23**, $\mathrm{{IC}^{CypB}_{50}}$ = 0.022 μM, $\mathrm{{IC}^{CypD(S123E)}_{50}}$ = 0.023 μM; **B25**, $\mathrm{{IC}^{CypB}_{50}}$ = 0.011 μM, $\mathrm{{IC}^{CypD(S123E)}_{50}}$ = 0.008 μM). We reasoned that S123 can better tolerate the carboxylate on **B23** in comparison to a negatively charged Glu at this position in CypB. This hypothesis was further supported by the conformational difference in the S123 backbone loop between co-crystal structures of **B2** versus **B23** bound to CypD (Extended Data Fig. 4a,b). Migration of the S123 loop in the **B23**–CypD structure compared to **B2**–CypD suggests that this conformational shift must be tolerated for any cyclophilin bound to **B23** (Extended Data Fig. 4a,b). Indeed, other cyclophilins with Glu at the 123 position (CypA, CypB, CypC, Cyp40) show weaker inhibition by **B23** compared to CypD. Surprisingly, neither **B23** nor **B25** show any appreciable difference in potency for CypD R124A or R124K compared to wild-type CypD, suggesting that R124 plays a minimal role in **B23** or **B25** binding (Supplementary Fig. 15b,c). Collectively, these observations suggest that the S123 gatekeeper plays a role in the selectivity profile of **B23** and **B25**.

Using these co-crystal structures and mutational analyses, we sought to achieve improved CypD selectivity over cyclophilins that share K118 (CypA, CypB, CypE) by installing carboxylate groups predicted to be positioned closer to the non-conserved S123 in CypD. Several of the resulting inhibitors retain similar potencies to **B23**, but none offer improvements in selectivity (**B35**–**B39**; Supplementary Fig. 16a–e). Many deleterious modifications were observed, including functionalizing the ethylene linker of **B23**, or altering the position of the carboxylate on the biphenyl group (**B40**–**B50**; Supplementary Fig. 17a–k). These modifications presumably change the location and binding mode of the carboxylate with respect to K118 and S119, thereby decreasing potency with no selectivity benefit. We reasoned that to gain further CypD selectivity, we must maintain the carboxylate’s interactions with K118 and S119 and add groups that can be presented to the S123 position. As the analogous S123 position is a more sterically bulky lysine in CypE, and a negatively charged glutamate in CypA and CypB, presenting a carboxylate group closer to this region could contribute additional CypD selectivity over these cyclophilins (Extended Data Fig. 1d,e). We subsequently made a disubstituted nitrile/carboxylate biphenyl derivative (**B51**), similar to **B34**, and two dicarboxylate moieties (**B52**, **B53**) to test this hypothesis.

**Fig. 4:**
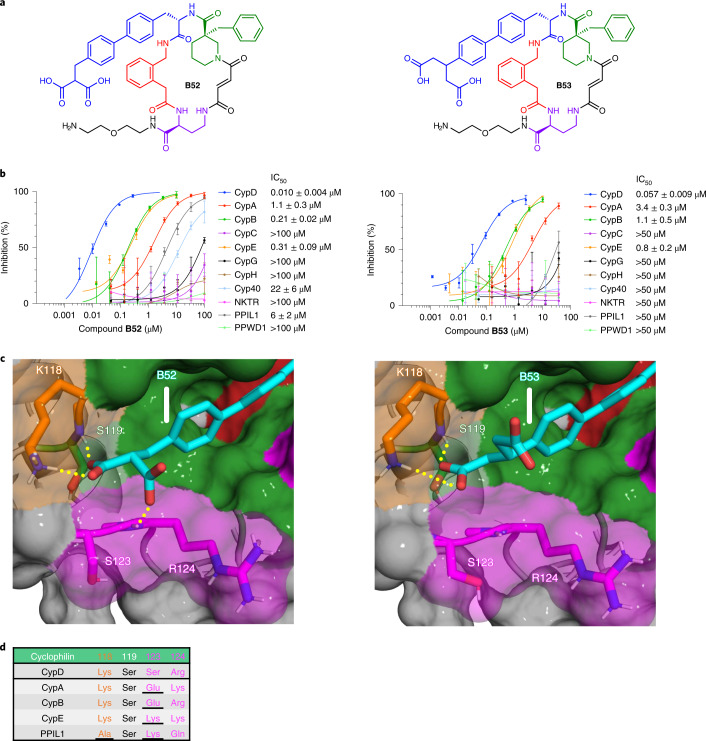
Biphenyl dicarboxylates achieve strong CypD selectivity. **a**, Structure of **B52** and **B53**. **b**, Cyclophilin prolyl-isomerase inhibition screens for **B52** and **B53**. **c**, Co-crystal structures of **B52** (PDB ID 7THD, 1.16 Å resolution) and **B53** (PDB ID 7THF, 1.10 Å resolution) bound to CypD, viewing the S2 pocket. Active site binding is identical to **A26** (Fig. 1e). Yellow dashes indicate predicted hydrogen bonds. **d**, List of residues on the far side of the S2 pocket of cyclophilins that are proximal to the ligand carboxylates. Both compounds retain CypD potency similar to that of mono-carboxylate **B23**, while enhancing selectivity over CypA, CypB, CypE, and PPIL1. The malonic and glutaric acids of **B52** and **B53**, respectively, position the carboxylate in a similar pose as **B23** (Fig. 3c), while presenting a second carboxylate to the S123 residue. **B52** forms a predicted hydrogen bond with the peptide backbone of S123–R124. R124 is pushed out of the S2 pocket, consistent with other macrocycles containing large S2-binding groups such as **B1**. **B52** and **B53** achieve selectivity over CypA and CypB through charge repulsion with a glutamate at the analogous 123 position, while creating a steric clash with PPIL1 and the lysine of CypE at this same position. IC_50_ values reflect mean ± s.d. of four independent replicates (each comprising three technical replicates). Graphs show a representative single independent replicate (independent replicate 1 is shown, containing three technical replicates) with data points and error bars reflecting mean ± s.d. of individual assays at one dose. Further independent replicates are shown in Supplementary Fig. 18b,c. Source data

**Extended Data Fig. 5:**
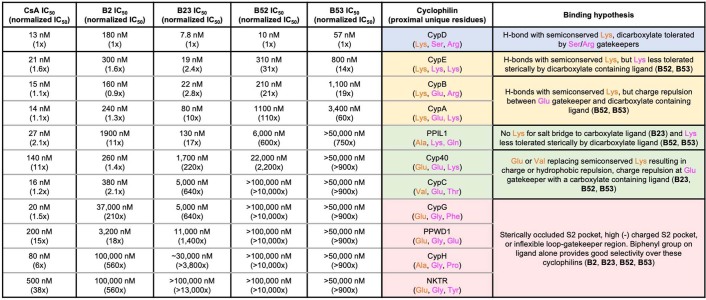
Analysis of CypD-selective prolyl isomerase inhibition based on S2 pocket containing residues. IC_50_ values for each cyclophilin are accompanied by fold difference normalized to ${\mathrm{IC}}^{\mathrm{CypD}}_{50}$. Residues listed next to each cyclophilin are the important proximal residues near the carboxylate-containing ligands. CsA does not bind the S2 pocket and shows almost no cyclophilin isoform selectivity, while **B2’s** large biphenyl group exhibits selectivity over cyclophilins with sterically occluded S2 pockets. Further selectivity over CypC, Cyp40, and PPIL1 is achieved through interactions between CypD’s K118 residue and carboxylate-containing ligands such as **B23**. Installation of dicarboxylates in **B52** and **B53** result in CypD selectivity by presenting a second carboxylate near the analogous S123 position on CypD. Potency for CsA, **B2**, and **B23** values reflect mean of three technical replicates. Potency for **B52** and **B53** values reflect mean of four independent replicates.

While the nitrile of **B51** only hampers the selectivity compared to the **B23** carboxylate (Supplementary Fig. 18a), **B52** and **B53**, which contain malonate or glutarate moieties, respectively, retain CypD potency (**B52** $\mathrm{{IC}^{CypD}_{50}}$ = 0.010 μM; **B53** $\mathrm{{IC}^{CypD}_{50}}$ = 0.057 μM) compared to **B23**, while further improving CypD selectivity (Fig. 4a,b and Supplementary Fig. 18b,c). **B52** and **B53** show ~15- to 30-fold selectivity for inhibiting CypD over the most closely related cyclophilins, CypE and CypB, 60- to 900-fold selectivity over CypA and PPIL1, and >900-fold selectivity against the remaining six cyclophilins (Extended Data Fig. 5). The 100-fold selectivity of **B52** and **B53** for CypD over CypA is especially noteworthy since CypA is the most abundantly expressed cyclophilin in human cells, and one of the most abundantly expressed intracellular proteins (Supplementary Table 1). We then solved co-crystal structures of **B52** and **B53** bound to CypD (Fig. 4c; PDB IDs 7THD, 7THF). We observed a similar binding mode as **B23**, maintaining a salt bridge with K118, but now presenting a second carboxylate in the proximity of the S123 and R124 residues, as designed. For **B52**, we also observed a novel hydrogen bond with the backbone atoms between S123 and R124. We therefore attributed the selectivity of **B52** and **B53** for CypD over CypA, CypB, and CypE to the presentation of the second carboxylate near S123 (Fig. 4d).

To test this basis of selectivity, we assayed **B52** and **B53** against CypD gatekeeper mutants S123E, R124A, and R124K (Supplementary Figs 14d,e and 15d,e), along with K118A and K118E mutants (Supplementary Fig. 13d,e). We observed similar trends as seen with monocarboxylates **B23** and **B25** compared with wild-type CypD—decreased potency for CypD S123E (**B52** $\mathrm{{IC}^{CypD(S123E)}_{50}}$ = 0.24 μM; **B53** $\mathrm{{IC}^{CypD(S123E)}_{50}}$ = 0.8 μM) roughly equal to CypB (**B52** $\mathrm{{IC}^{CypB}_{50}}$ = 0.21 μM; **B53** $\mathrm{{IC}^{CypB}_{50}}$ = 1.1 μM), little to no ablation of potency for the CypD R124A and R124K mutants, and a decrease in potency for CypD K118A (**B52** $\mathrm{{IC}^{CypD(K118A)}_{50}}$ = 0.073 μM; **B53** $\mathrm{{IC}^{CypD(K118A)}_{50}}$ = 0.9 μM) and K118E (**B52** $\mathrm{{IC}^{CypD(K118E)}_{50}}$ = 0.3 μM; **B53** $\mathrm{{IC}^{CypD(K118E)}_{50}}$ = 4 μM) mutants upon removing the possibility of an inhibitor–protein salt bridge. These results suggest that **B52** and **B53** achieve substantial CypD selectivity on the basis of favorable contacts with its K118 and S123 residues, and on the absence of such interactions with homologous positions in other cyclophilins.

**Extended Data Fig. 6:**
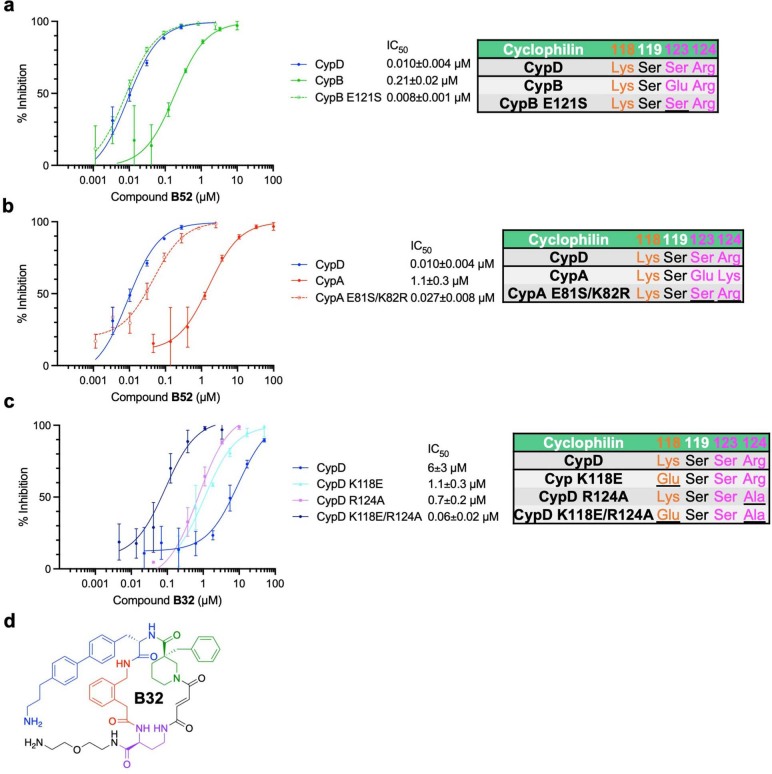
Inhibition potency is dependent on favorable interactions with S2 pocket residues. Dose response curves for **B52** against **a**, CypB E121S mutant and **b**, CypA E81S/K82R double mutant, with residue tables for important dicarboxylate proximal residues. Mutated residues are underlined. **B52** inhibits both CypB and CypA mutants that have the appropriate ‘CypD’ gatekeeper residues at similar potency values compared to wild-type CypD. **c**, Dose response curves for **B32** against wild-type CypD and CypD mutants shown in the table. Two mutations to remove positively charged residues K118 and R124 restore inhibition potency of amine derivative **B32**. **d**, Structure of **B32**. For CypD, CypA, and CypB wild-type IC_50_ data with **B52**, values reflect mean ± s.d. of four independent replicates (each comprising three technical replicates). Graphs show a representative single independent replicate (Independent replicate 1 is shown, containing three technical replicates) with data points and error bars reflecting mean ± s.d. of individual assays at one dose. Further independent replicates are shown in Supplementary Fig. 18b-c. All other IC_50_ values reflect mean ± s.e.m. of three technical replicates, with data points and error bars reflecting mean ± s.d. of individual assays at one dose. Source data

**Extended Data Fig. 7:**
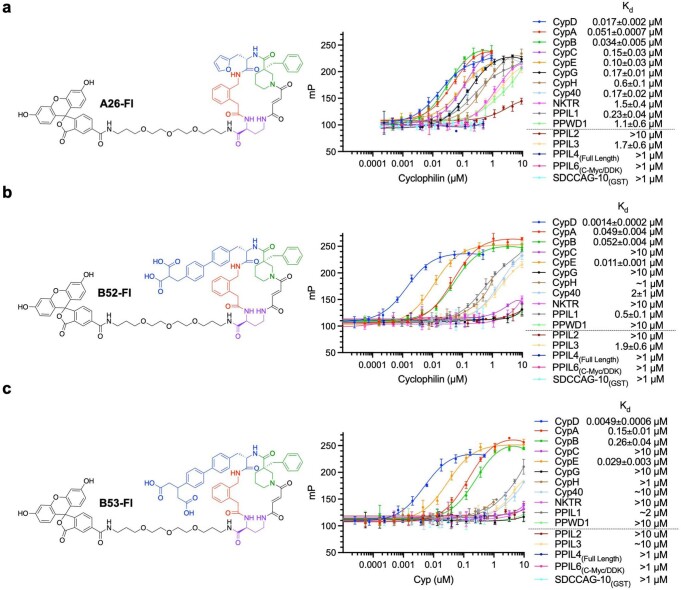
Cyclophilin binding profiles using fluorescence polarization of fluorescein-labeled macrocycles. Each cyclophilin was titrated against 0.5 nM fluorescein-labeled macrocycle **a, A26-Fl**; **b**, **B52-Fl**; or **c**, **B53-Fl**. Trends for selectivity follow those observed in the prolyl-isomerase assay. Cyclophilins in the legend below the dashed line are either prolyl-isomerase inactive or require much higher concentrations to observe prolyl isomerization *in vitro*. K_d_ values and error bars reflect mean ± s.e.m. of three technical replicates. Data points and error bars reflect mean ± s.d. of individual assays at one dose. Source data

To further establish the dependence of CypD inhibitor potency on S2 pocket residues, we sought to rescue the attenuated potency of **B52** and **B53** for both CypB and CypA by installing gatekeeper mutations to match the residues of CypD in this pocket (CypB E121S, CypA E81S/K82R). Both CypB E121S and CypA E81S/K82R mutants retain prolyl-isomerase activity (Supplementary Fig. 3d,e). As expected, both **B52** and **B53** have similar potencies for CypD as CypB E121S (**B52**, $\mathrm{{IC}^{CypD}_{50}}$ = 0.010 μM, $\mathrm{{IC}^{CypB(E121S)}_{50}}$ = 0.008 μM; **B53**, $\mathrm{{IC}^{CypD}_{50}}$ = 0.057 μM, $\mathrm{{IC}^{CypB(E121S)}_{50}}$ = 0.05 μM), rescuing the 15- to 20-fold inhibition potency difference of **B52** and **B53** for CypD versus wild-type CypB (**B52**, $\mathrm{{IC}^{CypB}_{50}}$ = 0.21 μM; **B53**, $\mathrm{{IC}^{CypB}_{50}}$ = 1.1 μM) (Extended Data Fig. 6a and Supplementary Fig. 19d,e). Similarly, we observed **B52** and **B53** to have similar potencies for CypD and CypA E81S/K82R (**B52**, $\mathrm{{IC}^{CypD}_{50}}$ = 0.010 μM, $\mathrm{{IC}^{CypA(E81S/K82R)}_{50}}$ = 0.027 μM; **B53**, $\mathrm{{IC}^{CypD}_{50}}$ = 0.057 μM, $\mathrm{{IC}^{CypA(E81S/K82R)}_{50}}$ = 0.18 μM), rescuing the ~100-fold inhibition potency difference of **B52** and **B53** for CypD versus wild-type CypA (**B52**, $\mathrm{{IC}^{CypA}_{50}}$ = 1.1 μM; **B53**, $\mathrm{{IC}^{CypA}_{50}}$ = 3.4 μM) (Extended Data Fig. 6b, Supplementary Fig. 20d,e). These trends were also conserved to a lesser degree for monocarboxylates **B23** and **B25** (Supplementary Figs 19b,c and 20b,c). Inhibition trends were maintained with fluorescein-labeled **A26** (**A26-Fl**), **B52** (**B52-Fl**), and **B53** (**B53-Fl**), using a fluorescence polarization assay to measure binding against 11 prolyl-isomerase-active and five catalytically impaired or inactive cyclophilins (Extended Data Fig. 7a–c). While **A26-Fl** promiscuously binds the 11 prolyl-isomerase-active cyclophilins, **B52-Fl** and **B53-Fl** exhibit selective CypD binding, corroborating prolyl-isomerase inhibition (Figs 1c and 4b). We also observed weak binding to the five impaired or non-PPIase active cyclophilins by **A26-Fl**, **B52-Fl**, and **B53-Fl**, suggesting that their macrocycle scaffold cannot target these cyclophilins. Collectively, these results confirm that interactions between inhibitors and S2 pocket residues are strong determinants of CypD potency and selectivity.

We also reasoned that we could modify the S2 pocket of CypD to accommodate the amine containing compound **B32**, as it shows an alternate selectivity profile compared to most of our inhibitors, but has poor overall potency (Extended Data Fig. 6c,d and Supplementary Fig. 11b). We observed that replacing cationic residues within the S2 pocket of CypD (K118E, R124A) improves **B32** potency by 5- to 6-fold compared to wild-type CypD ($\mathrm{{IC}^{CypD}_{50}}$ = 6 μM, $\mathrm{{IC}^{CypD(K118E)}_{50}}$ = 1.1 μM, $\mathrm{{IC}^{CypD(R124A)}_{50}}$ = 0.7 μM; Extended Data Fig. 6c). Combining these two mutants (K118E/R124A) synergistically increases potency ($\mathrm{{IC}^{CypD(K118E/R124A)}_{50}}$ = 0.06 μM), a 100-fold improvement compared to wild-type CypD (Extended Data Fig. 6c). **B32** exhibits good selectivity for CypD K118E/R124A over wild-type cyclophilins, with at least 13-fold preference over the nearest cyclophilin, PPWD1 (Supplementary Fig. 11b) and >100-fold selectivity over CypA. Overall, **B32** and CypD K118E/R124A provide another cyclophilin–ligand pair with selective inhibition dependent on S2 pocket identity.

### CypD inhibitors are active in mitochondria

The lack of reliable functional CypD assays in intact cells makes it difficult to directly measure CypD inhibition in intact cell culture. Consistent with multiple previous reports, our efforts to use a calcein–Co^2+^ assay and mitochondrial membrane potential dyes in cells to characterize our compounds with CsA as a control failed to reliably assess mPTP opening in a CypD-inhibition-dependent manner and were also prone to confounding signals^39–44^. For these reasons, CypD inhibitors have mostly been characterized biochemically in vitro, or in isolated mitochondria^13,16,45–47^.

**Fig. 5:**
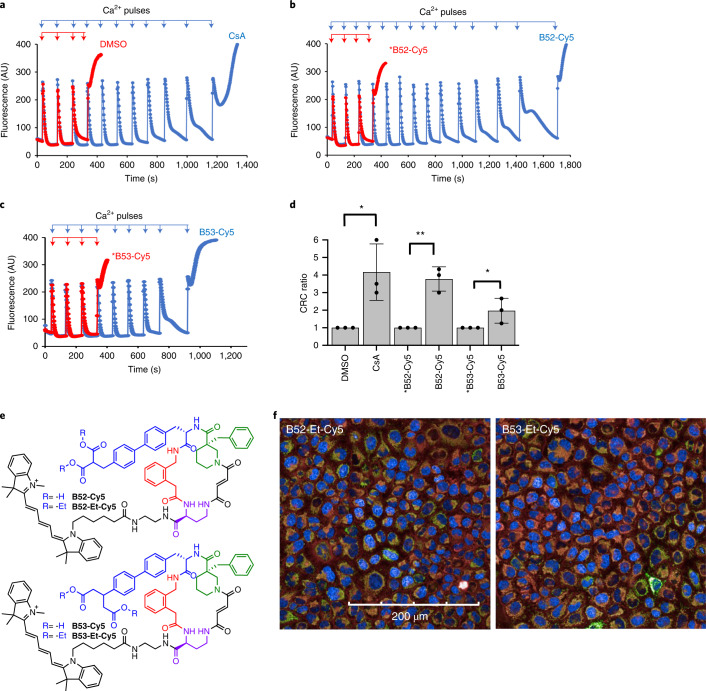
Cy5-conjugated cyclophilin D inhibitors delay calcium induced opening of the mPTP in isolated mouse liver mitochondria and enter human cells as ester prodrugs. The calcium retention capacity of mitochondria was determined in isolated mouse liver mitochondria (0.5 µg mL^−1^) in response to pulses of 60 µM CaCl_2_ in the presence of the indicated CypD inhibitors (or inactive enantiomers). Concentrations used were 2 µM CsA, 10 µM **B52-Cy5**, 10 µM *B52-Cy5, 20 µM **B53-Cy5**, 20 µM *B53-Cy5. Mitochondrial uptake of extramitochondrial Ca^2+^ was assessed by monitoring the fluorescence of Calcium green 5n, depicted in arbitrary units (AU). The rapid increase in fluorescence after several pulses of Ca^2+^ are taken up corresponds to mitochondrial Ca^2+^ release through mPTP opening. **a**–**c**, Traces are shown from a representative experiment, with all assays performed on the same mitochondrial preparation and day. **d**, Quantitation of calcium retention capacity (CRC) reported as the ratio of the number of Ca^2+^ pulses required to induce mPTP opening in the listed condition relative to DMSO control conditions on the same mitochondrial preparation and day. **e**, Structures of Cy5-conjugated CypD-selective inhibitors and prodrugs. **f**, Fluorescence microscopy of HeLa cells co-incubated with ester prodrugs **B52-Et-Cy5** and **B53-Et-Cy5** (red), co-stained with mitochondrial (green) and nuclear (blue) dyes (Mitotracker Green and Hoechst 33342, respectively). Both prodrugs show good plasma membrane permeability and co-localization with Mitotracker Green, quantified in Extended Data Fig. 8a–i. Calcium retention data are from three independent experiments/mitochondrial isolations. Data bars and error bars represent mean ± s.d. DMSO versus CsA, *P* = 0.0135; *B52-Cy5 versus B52-Cy5, *P* = 0.0011; *B53-Cy5 versus B53-Cy5, *P* = 0.0382. *P < 0.05, **P < 0.005 by one-sided Student’s *t*-test. Microscopy images are a representative image of three technical replicates. Scale bars, 200 µm. Source data

We tested the ability of our CypD-selective macrocycles to inhibit CypD in active mitochondria isolated from mouse liver. First, we verified that modifying groups on the ‘tail’ position of **B52** and **B53** (for example, **B52-A**, **B53-A**) do not affect potency or selectivity, and that their enantiomers do not inhibit any cyclophilin (*B52-A, *B53-A) (Supplementary Fig. 21a–d). Since CypD is exclusively found in mitochondria, we appended Cy5, an established mitochondrial localization group^48^, to all four macrocycles at the ‘tail’ position (**B52-Cy5**, **B53-Cy5**, *B52-Cy5, *B53-Cy5). We observed minimal change in potency or selectivity for CypD in vitro upon addition of the Cy5 group (Supplementary Fig. 22a–d). We then tested compounds **B52-Cy5** and **B53-Cy5** in isolated mouse liver mitochondria, measuring their calcium retention before a mPTP opening event. In comparison to the dimethysulfoxide (DMSO) control, we observed substantially increased calcium retention capacity before mPTP opening when pretreated with CsA, **B52-Cy5**, or **B53-Cy5** (Fig. 5a–d and Supplementary Fig. 23a,b). Importantly, inactive enantiomers *B52-Cy5 and *B53-Cy5 do not inhibit CypD in vitro (Fig. 5a–d and Supplementary Fig. 23a,b) and do not influence calcium retention capacity before mPTP opening, strongly supporting CypD-dependent inhibition of mPTP opening by **B52-Cy5** and **B53-Cy5**.

**Extended Data Fig. 8:**
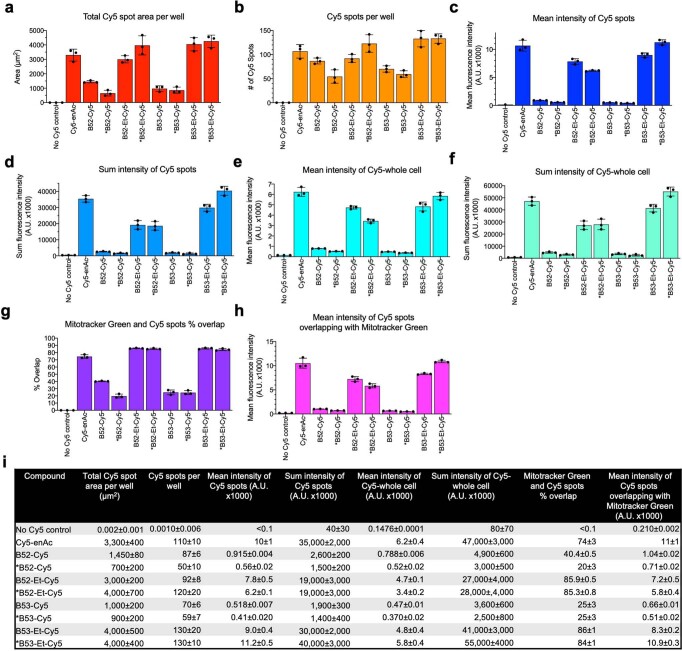
Quantification of mitochondrial localization in HeLa cells by fluorescence microscopy of Cy5-conjugated compounds. HeLa cells were treated with Cy5-conjugated compounds and analyzed for **a**, total identifiable Cy5 spots; **b**, Cy5 spots per well; **c**, mean fluorescence intensity of identified Cy5 spots; **d**, sum of fluorescence intensity of identified Cy5 spots; **e**, mean Cy5 fluorescence intensity per cell; **f**, sum of Cy5 fluorescence in all measured cells; **g**, percent of Cy5 spots that overlap >70% with Mitotracker Green co-stain; and **h**, fluorescence intensity of Cy5 spots that overlap >70% with Mitotracker Green. **i**, values of data shown in **a**-**h**. Values and error bars reflect mean ± s.d. of three technical replicates. Source data

**Extended Data Fig. 9:**
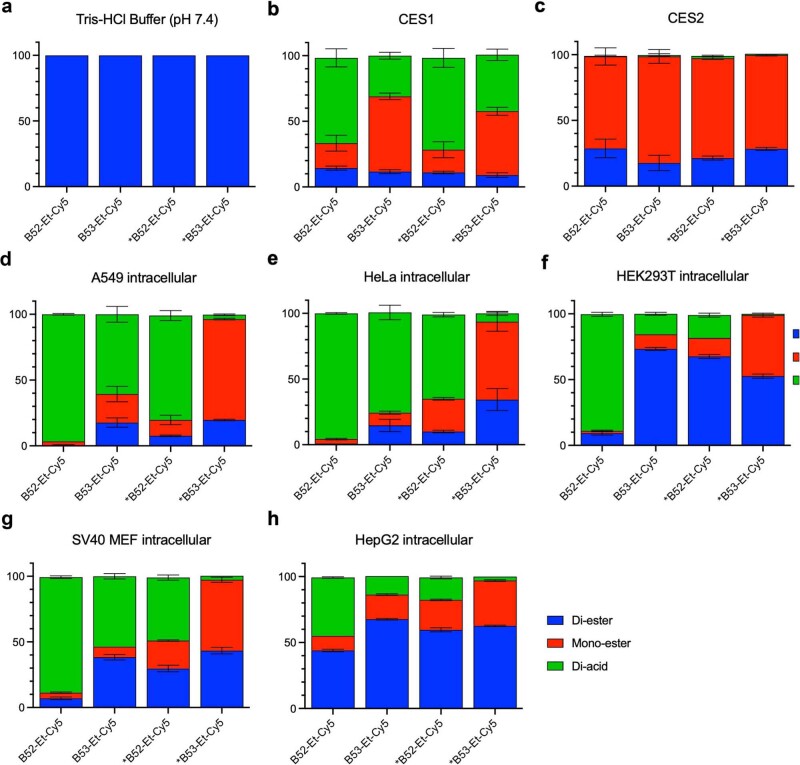
Hydrolysis of ester prodrug CypD inhibitors. Compounds were evaluated for their ability to be hydrolyzed from di-ester to mono-ester, or to di-acid CypD inhibitors. Each reaction was analyzed by LC-MS, and ion abundances for each are shown as a percent of the total sum. These were conducted under conditions of: **a**, Tris-HCl buffer only; **b**, 250 nM carboxylesterase 1 (CES1); **c**, 250 nM carboxylesterase 2 (CES2); **d**, incubated with A549 cells for 48 h and intracellular fraction isolated; **e**, incubated with HeLa cells for 48 h and intracellular fraction isolated; **f**, incubated with HEK293T cells for 48 h and intracellular fraction isolated; **g**, incubated with MEFs for 48 h and intracellular fraction isolated; **h**, incubated with HepG2 cells for 36 h and intracellular fraction isolated. Esters show good stability in buffer and are only cleaved under esterase conditions, or intracellularly, with **B52-Et-Cy5** showing the most rapidly hydrolyzed esters. Values and error bars reflect mean ± s.d. of three technical replicates. Source data

As expected, while **B52-Cy5** and **B53-Cy5** engage CypD in isolated mitochondria, we did not observe efficient plasma membrane permeability of **B52-Cy5**, **B53-Cy5**, or their inactive enantiomers by fluorescence microscopy (Extended Data Fig. 8a–i and Supplementary Fig. 24b–f), presumably because of the presence of dicarboxylate groups. To improve cell permeability, we used a pro-drug strategy and prepared both sets of active and inactive enantiomers as ethyl esters (**B52-Et-Cy5**, **B53-Et-Cy5**, *B52-Et-Cy5, *B53-Et-Cy5) (Fig. 5e and Supplementary Fig. 24a). We observed strong mammalian cell permeability and mitochondrial localization for all four ester-containing compounds (Fig. 5f, Extended Data Fig. 8a–i and Supplementary Fig. 24b–f). These ester derivatives did not potently inhibit the prolyl-isomerase activity of CypD, consistent with the importance of the dicarboxylate groups (Supplementary Fig. 25). All four pro-drug compounds were hydrolyzed to their active dicarboxylate forms in vitro with human carboxylesterase (CES1), and **B52-Et-Cy5**, *B52-Et-Cy5, and **B53-Et-Cy5** were readily converted to the corresponding di-acids intracellularly by endogenous esterases in a variety of human (A549, HeLa, HEK293T, HepG2) and mouse (mouse embryonic fibroblasts (MEFs)) cell lines (Extended Data Fig. 9a–h).

These findings, coupled with robust CypD-dependent inhibition of mPTP opening in isolated mitochondria, suggest that pro-drug versions of Cy5-conjugated **B52** and **B53** can access mitochondria and release active CypD-selective inhibitors in mammalian cells. These observations make esterified **B52** and **B53** attractive candidates for potent and selective probes of CypD activity in biological systems.

### Development of CypE-selective inhibitors

Since CypD-selective inhibitors **B52** and **B53** were designed from a slightly promiscuous inhibitor **B2**, we next explored the possibility of selectively modulating a different cyclophilin by using covalent inhibition. While most covalent inhibitors target catalytic residues, the non-conserved residues in cyclophilin S2 pockets are both solvent-exposed and non-catalytic. Many cyclophilins contain non-catalytic lysines in their S2 pocket (Extended Data Fig. 1d,e). Although non-catalytic lysines are typically protonated at physiological pH, aryl boronic acid carbonyls have been shown to modify lysine and N-terminal groups covalently and reversibly through the formation of iminoboronates. Previous studies have incorporated aryl boronic acid carbonyl warheads on inhibitors to covalently modify non-catalytic lysines^49^. We hypothesized that installing this reactive group on the biphenyl of inhibitor **B2** might allow a reversible covalent interaction with one of these cyclophilins, potentially providing a new subtype-selective inhibitor. We synthesized ketone (**C1A**, **C2A**) and aldehyde boronic acids (**C3A**, **C4A**) that could place this warhead close to cyclophilin S2 pocket lysine residues (Supplementary Fig. 26a–d).

**Fig. 6:**
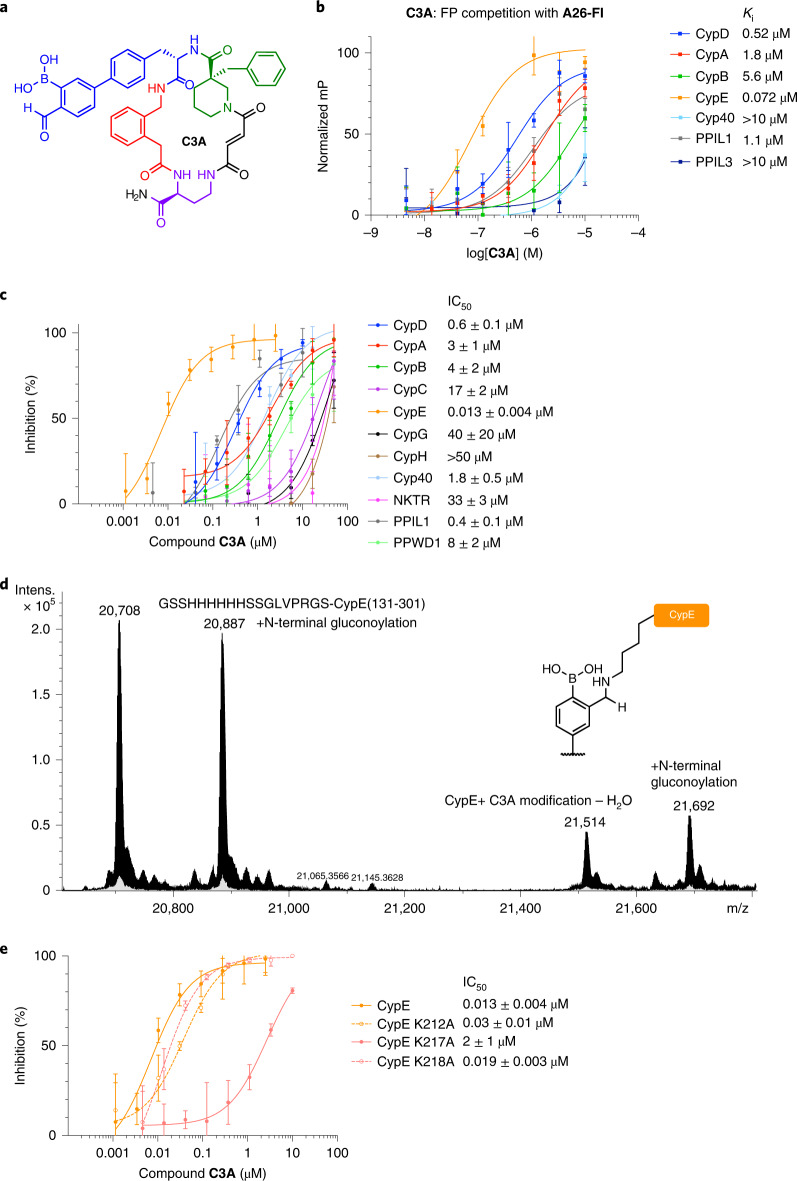
Aryl-carbonyl boronic acid C3A achieves selective inhibition of CypE. **a**, Structure of **C3A**. **b**, **C3A** fluorescence polarization (FP) competition with **A26-Fl** against cyclophilins with S2 pocket lysines. **c**, Cyclophilin prolyl-isomerase inhibition screen of **C3A**, showing potency and selectivity for CypE. **d**, Mass spectroscopy trace of CypE incubated with **C3A** and reduced with sodium cyanoborohydride. **C3A** shows an adduct consistent with CypE + amine–H_2_O (+806 Da), the result of iminoboronate formation followed by reductive amination. The mass of CypE is 20,708 Da, The CypE preparation also included N-terminal gluconoylation. **e**, Prolyl-isomerase inhibition by **C3A** against CypE S2 pocket lysine to alanine mutants. For the FP assay, the *y*-axis is normalized to internal control wells containing **A26-Fl** only (100%) and **A26-Fl** with cyclophilin (0%). Values reflect mean of three technical replicates and error bars reflect s.d. of individual assays at one dose. For the prolyl-isomerase assay in **c** and the CypE wild-type dose–response curve in **e**, IC_50_ values reflect mean ± s.d. of four independent replicates (each comprising three technical replicates). Graph shows a representative single independent replicate (Independent replicate 3 is shown, containing three technical replicates) with data points and error bars reflecting mean ± s.d. of individual assays at one dose. Further independent replicates are shown in Supplementary Fig. 28b. For the prolyl-isomerase assay in **e**, IC_50_ values for the CypE mutants reflect mean ± s.e.m. of three technical replicates, while data points and error bars reflect mean ± s.d. of individual assays at one dose. Source data

We screened these four compounds in a fluorescence polarization competition assay with **A26-Fl** against seven lysine-containing cyclophilins (Supplementary Fig. 26a–d), identifying **C3A** as a potent inhibitor of **A26-Fl** binding to CypE (*K*_i_ of 0.072 µM) (Fig. 6a,b and Supplementary Fig. 26c). Additionally, **C3A** showed ≥10-fold selectivity for CypE over other tested cyclophilins. Shifting the aldehyde group to the meta position of the ring (**C4A**) attenuated CypE potency and selectivity, suggesting improper placement of **C4A**’s aryl boronic acid carbonyl towards the lysines of CypE (Supplementary Fig. 26d). Replacing the aldehyde with a ketone (**C1A**, **C2A**) also decreased potency for CypE compared to **C3A** (Supplementary Fig. 26a,b). Compounds **C5A** and **C6A** containing either the aldehyde or the boronic acid alone, respectively, showed reduced potency by 16-fold and 100-fold, respectively, relative to **C3A** (Supplementary Fig. 27a–c). Since the potency of **C3A** is contingent on both the boronic acid and the aldehyde, these results suggest **C3A** is acting covalently through iminoboronate formation with CypE, consistent with the lysine-rich S2 pocket of CypE (Extended Data Fig. 1d,e).

Macrocycle **C3A** is also a potent inhibitor against CypE prolyl-isomerase activity, with an IC_50_ of 0.013 µM (Fig. 6c and Supplementary Fig. 28b). **C3A** is also very selective for CypE with an IC_50_ that is at least 30- to 4,000-fold more potent for CypE than the other ten cyclophilins screened. Once again, replacement of the aldehyde with a ketone (**C1A**, $\mathrm{{IC}^{CypE}_{50}}$ = 0.9 μM), or removal of either the boronic acid (**C5A**, $\mathrm{{IC}^{CypE}_{50}}$ = 1 μM) or the aldehyde (**C6A**, $\mathrm{{IC}^{CypE}_{50}}$ = 4 μM) drastically reduces both the potency and selectivity compared to **C3A**, again supporting that **C3A** inhibits CypE covalently (Supplementary Fig. 28a–e).

**Extended Data Fig. 10:**
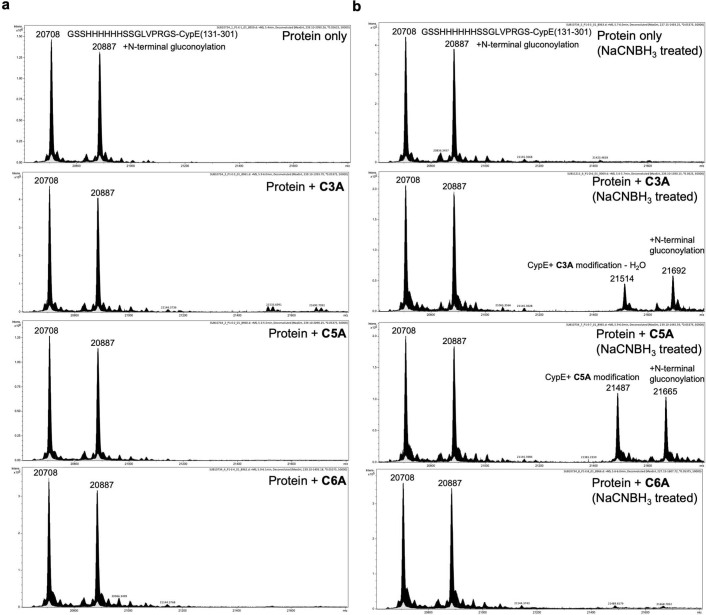
Mass spectroscopy analysis of compounds co-incubated with CypE. **a**, Mass spectroscopy analysis of lysine covalent modification of CypE with **C3A**, **C5A**, and **C6A**. **b**, Mass spectroscopy analysis of lysine covalent modification after treatment with NaCNBH_3_ to trap the iminoboronate as a reduced lysine-linked secondary amine, highlighting relevant m/z regions. **C3A** and **C5A** show covalent modification (+779 Da and +806 Da, respectively) of CypE after reductive amination with NaCNBH_3_.

Mass spectrometry did not detect a clear lysine-modified covalent adduct between **C3A** and CypE (Extended Data Fig. 10a). We speculated that reducing the iminoboronate intermediate with sodium cyanoborohydride (NaCNBH_3_) might trap the covalent adduct. Indeed, co-incubating **C3A** in fivefold excess for 1 h with CypE followed by treatment with 25 mM NaCNBH_3_ resulted in observation of a +806 Da adduct, corresponding to the reductive amination product minus water (Fig. 6d), but not for boronic acid **C6A** (Extended Data Fig. 10b). The loss of water has been reported for other aryl boronic acid carbonyl inhibitors treated with NaCNBH_3_^49^. These findings suggest that **C3A** functions in a reversible covalent manner. We also observed +779 Da covalent adduct formation under these conditions with aldehyde-only **C5A**, suggesting that carbonyl groups at this position can form imines with S2 pocket lysines of CypE (Extended Data Fig. 10b). Nonetheless, the 75-fold higher CypE inhibition potency of **C3A** over **C5A** suggests that this reversible interaction is substantially stronger for **C3A**. Little or no covalent modification for 12 other cyclophilins was observed when treated with **C3A** and NaCNBH_3_, establishing the selectivity of this covalent interaction (Supplementary Fig. 29a,b). To determine which CypE lysine residue is covalently modified by **C3A**, we generated alanine point mutants at each of the three S2 pocket lysines of CypE (K212A, K217A, K218A). Binding and inhibition potency by **C3A** for CypE K212A and K218A mutants was similar to wild-type CypE (Fig. 6e and Supplementary Figs 30b and 31b). By contrast, **C3A** was 40-fold less potent at competing **A26-Fl** from CypE K217A ($K^{\mathrm{CypE(K217A)}}_\mathrm{i}$ = 3.1 µM) compared to wild-type CypE ($K^{\mathrm{CypE}}_\mathrm{i}$ = 0.072 µM), and was 150-fold less potent at inhibiting K217A prolyl-isomerase activity ($\mathrm{{IC}^{CypE(K217A)}_{50}}$ = 2 μM) compared to wild-type CypE ($\mathrm{{IC}^{CypE}_{50}}$ = 0.013 µM).

Collectively, these results indicate that our model for achieving subtype-selective cyclophilin inhibition can be translated to other cyclophilin proteins, resulting in potent and selective CypE inhibition through reversible covalent bonding with the S2 pocket K217 residue of CypE.

## Discussion

We tailored promiscuous initial ligands from a DNA-templated library that bind cyclophilin active sites through iterated cycles of structural studies, mutagenesis, and small-molecule engineering to generate what are, to our knowledge, the first reported potent and subtype-selective inhibitors of CypD and of CypE. Cyclophilin subtype selectivity emerged through carefully engineered engagement with poorly conserved residues in the S2 pocket, an exo-site adjacent to each cyclophilin’s active site. Our results also suggest the feasibility of discovering subtype-selective cyclophilin ligands through parallel selections using a panel of subtypes to identify putative selective binders through their selective enrichment.

Structure–activity relationships and mutational analyses refined our model for CypD selectivity, which we applied to develop other selective cyclophilin inhibitors. CsA, an active-site-binding cyclophilin inhibitor, shows minimal selectivity for CypD over other cyclophilins and mutants (Extended Data Figs 1a–c and 5, Supplementary Table 2, and Supplementary Figs 13a, 14a, 15a, 19a, 20a and 31a). By contrast, our inhibitors that bind both the active site and S2 pocket of CypD offer improved selectivity (Extended Data Fig. 5 and Supplementary Table 2). The inclusion of large hydrophobic groups (**B1**, **B2**) into the S2 pocket eliminated off-target inhibition of CypG, CypH, and NKTR owing to the presence of inflexible or hydrophobic residues within their S2 pockets (Fig. 2a–d, Extended Data Fig. 5, and Supplementary Table 2). CypD, by contrast, contains a displaceable R124 residue that migrates upon ligand binding (Supplementary Figs 5a–c and 32).

Selectivity for CypD was further improved by installing carboxylates (**B23**, **B25**) that can hydrogen bond with S119 and K118, achieving selectivity over non-lysine-containing PPIL1, Cyp40, CypC, and PPWD1 (Fig. 3a–d, Extended Data Fig. 5, and Supplementary Table 2). To confer selectivity over K118-containing CypA, CypB, and CypE, we installed a second carboxylate (**B52**, **B53**) to interact with the gatekeeper residues on each cyclophilin, which show high potency and selectivity for CypD (Fig. 4a–d). CypD S123 can sterically and electronically tolerate dicarboxylates, while Glu at the analogous position in CypA and CypB presumably repels the dicarboxylate, and the more bulky lysine of CypE may sterically clash with the dicarboxylate (Fig. 4d, Extended Data Fig. 5, and Supplementary Table 2). Complementing these trends is the overall migration of S2 pocket residues in CypD to accommodate ligand binding (Extended Data Fig. 4b and Supplementary Fig. 32). Conformational flexibility of K118, S123, and R124 is dependent on the size and functionality of the binding ligand, as growing the S2-binding moiety of the ligand, while installing hydrogen-bond interactions, induces this residue migration (Supplementary Video 1).

Dicarboxylate groups on the inhibitors developed in this study thus provide a chemical environment that CypD, but not other cyclophilins, can accommodate. This model was further supported with molecular footprinting analysis of JOMBt compared to **B52** (Supplementary Fig. 33). JOMBt is reliant on weak binding energy per residue on highly conserved cyclophilin active site residues, while **B52** owes its potent CypD binding to unique S2 pocket residues that are poorly conserved between other family members. The conformational flexibility of K118, S123 and R124 is consistent with previous reports^1^ that suggest the S2 pocket of each cyclophilin may confer endogenous substrate-binding selectivity, whereby prolyl isomerization of a peptide substrate at the active site is only accessible if the S2 pocket can tolerate upstream amino acids. Indeed, the S2 pockets of each cyclophilin vary in their tolerance of different groups in our inhibitors. This observation is consistent with the S2 exo-site serving as a substrate-binding pocket separate from the active site that confers substrate selectivity. We further established that S2 pocket interactions are the basis of observed subtype selectivity through rational mutagenesis of the S2 pocket of CypD to accommodate a positively charged amine-containing ligand **B32** (Extended Data Fig. 6c,d and Supplementary Table 2).

We further extended these insights to achieve selective CypE inhibition. **C3A** selectively targets CypE though reversible covalent bonding with its S2 pocket K217 residue (Fig. 6a–e). To our knowledge, **C3A** represents the first CypE-selective inhibitor and the second type of subtype-selective cyclophilin inhibitor after **B52** and **B53** (Supplementary Table 2). The aryl aldehyde boronic acid forms a reversible covalent bond with the K217 residue of CypE, while the presence of the boronic acid minimizes binding to the other cyclophilins. CypE is one of many cyclophilin proteins reported as a structural component of the spliceosome, but the assessment of its mechanistic role has been hampered by the lack of a cyclophilin-selective inhibitor^26^. The use of CypE-selective inhibitors such as **C3A** may serve to illuminate the role of CypE in the spliceosome without confounding inhibition of the seven other spliceosome-associated cyclophilins^26^.

The CypD-selective inhibitors developed in this work increase calcium retention capacity and delay mPTP opening in isolated mitochondria (Fig. 5a–d and Supplementary Fig. 23a,b). Ester pro-drug derivatives of these CypD-selective inhibitors also exhibit strong plasma membrane permeability, mitochondrial localization, and the ability to release active inhibitor in mammalian cells (Fig. 5e,f, Supplementary Figs 24a–f and 25, and Extended Data Figs. 8a–i and 9a–h). These compounds may serve as tools to explore the mechanism of action of the mPTP or as probes of the role of CypD in disease models with mPTP-oxidative stress as a phenotype, such as neurodegenerative disorders, IRI, liver diseases, and mitochondrial disorders.

More broadly, this study establishes that subtype-selective cyclophilin inhibition can be achieved through the design of ligands that interact with unique residues in cyclophilin exo-site S2 pockets (Extended Data Fig. 1d,e and Supplementary Table 2). Applying this strategy to develop additional selective cyclophilin inhibitors may unlock the biological or therapeutic potential of this family of targets, which have been relatively unexplored compared to others such as kinases, phosphatases, and proteases.

## Methods

### General

Fmoc-protected amino acids and peptide coupling reagents were purchased from Chem-Impex International. Boronic acids or aryl halides were purchased from Combi-Blocks and Enamine, verified >95% pure by the manufacturer’s standards. All other chemical reagents (verified >95% pure by manufacturer), PPIL4 (full length), and PPIL6(C-Myc/DDK), were purchased from Millipore-Sigma. SDCCAG-10(GST) was purchased from Abnova. Human recombinant CES1 and CES2 were purchased from R&D Systems. All purchased proteins were verified >85% pure by the manufacturer using SDS-PAGE. Nuclear magnetic resonance (NMR) spectra were gathered using a Bruker Ascend 400 MHz NMR. Quantification of DNA was completed using a NanoDrop One Microvolume UV-Vis Spectrophotometers (ThermoFisher Scientific). Preparative high-performance liquid chromatography (HPLC) reverse phase purification was conducted with an Agilent 6100 Quadrupole liquid chromatography–mass spectrometry (LC–MS) system using a Kinetex 5 µm C18 100 Å, AXIA Packed LC Column 150 ×30.0 mm (Phenomenex). Silica gel column chromatography was conducted with a Biotage SP1 Flash Chromatography system. Recombinantly expressed proteins were purified using an AKTA pure fast protein liquid chromatography (FPLC). Quantitative PCR analysis was conducted using a CFX96 Touch Deep Well Real-Time PCR System (Bio-Rad). ^1^H-NMR spectra for all reported compounds are provided in Supplementary Note 2 of the Supplementary Information. HRMS data is provided in Supplementary Tables 7 and 8. HPLC purity analysis of important compounds is included within Supplementary Note 2 of the Supplementary Information. All tested compounds were quantified with CH_2_Cl_2_ internal standard by ^1^H-NMR.

### General mammalian cell culture conditions

HEK293T (American Type Culture Collection (ATCC) CRL-3216), HeLa (ATCC CCL-2), MEFs (ATCC CRL-2991), HepG2 (ATCC HB-8065), and A549 (ATCC CCL-185) cells were purchased from ATCC and cultured and passaged in Dulbecco’s modified Eagle’s medium (DMEM) plus GlutaMAX (ThermoFisher Scientific) for HEK293T/HeLa/MEFs, minimum essential medium (MEM; Corning) for HepG2, and F-12K (ATCC) for A549, each supplemented with 10% (v/v) fetal bovine serum (FBS; Gibco, qualified). All cell types were incubated, maintained, and cultured at 37 °C with 5% CO_2_. Cell lines were authenticated by their respective suppliers and tested negative for mycoplasma.

### HPLC purity analyses of key compounds

Analytical analyses for compound purity was performed using an Agilent 6100 Quadrupole LC–MS system with a Kinetex 5 µm C18 100 Å LC Column 150 ×2.1 mm (Phenomenex). Five microliters of 0.5–1 mM compound in DMSO-d_6_ was injected and run for 3 min at 10% water/acetonitrile with 0.1% trifluoroacetic acid to elute off DMSO-d_6_. From 3 min to 15 min, gradient was increased to 100% acetonitrile, and held for 2 more min. Compound peaks were identified using the mass spectrometry trace. Purity analyses was quantified by percentage area of the compound peak at 214 nm absorbance relative to all identified peaks within the 3–17 min analysis window (to exclude the DMSO peak at 1.5 min).

### Generation of His_6_-Cyp expression constructs

Geneblock sequences for PPIF (UniProtKB Entry P30405: residues 45–207), PPIA (UniProtKB Entry P62937: residues 1–165), PPIB (UniProtKB Entry P23284: residues 34–216), PPID (UniProtKB Entry Q08752: residues 1–183), PPIH (UniProtKB Entry O43447: residues 1–177), PPIL1 (UniProtKB Entry Q9Y3C6: residues 1–166), and PPIL3 (UniProtKB Entry Q9H2H8: residues 1–161) were purchased from IDT and cloned into 2BT (UC Berkeley QB3 MacroLab) using ligation independent cloning. Gatekeeper mutations for PPIA and PPIB were introduced using site-directed mutagenesis with Q5 DNA polymerase (NEB) and primers from IDT. All constructs were verified using Sanger sequencing. *Escherichia coli* containing the constructs were cultured overnight at 37 °C in 2× YT medium (31 g in 1 L) containing 100 µg mL^−1^ ampicillin.

PPIL2 (UniProtKB Entry Q13356: residues 280–457) cloned into pET28a LIC (Addgene Plasmid #25601), PPIG (UniProtKB Entry Q13427: residues 1–179) cloned into pET28a LIC (Addgene Plasmid #25137), PPIE (UniProtKB Entry Q9UNP9: residues 131–301) cloned into pET28a LIC (Addgene Plasmid #25605), PPWD1 (UniProtKB Entry Q96BP3: residues 473–646) cloned into pET28a LIC (Addgene Plasmid #25600), PPIC (UniProtKB Entry P45877: residues 24–212) cloned into pET28a LIC (Addgene Plasmid #25606), and NKTR (UniProtKB Entry P30414: residues 7–179) cloned into pET28a LIC (Addgene Plasmid #25597) were purchased as bacterial agar stabs and expressed in BL21DE3 cells. *E. coli* containing the constructs were cultured overnight at 37 °C in 2× YT medium (31 g in 1 L) containing 50 µg mL^−1^ kanamycin.

### Site-directed mutagenesis of CypD

Mutant CypD constructs were generated with primers for one-piece uracil-specific excision reactions (USER) containing a mutant overhang by PCR amplification of starting plasmids (Supplementary Table 3). PCR product was purified on microcentrifuge membrane columns (MinElute, Qiagen) and quantified by Nanodrop. Fragment (0.2 pmol, 7.5 µL) was combined in a 10 µL reaction mixture containing 0.75 µL (15 U) DpnI (NEB), 0.75 µL USER mix (endonuclease VIII and uracil-DNA glycosylase, NEB), 1 µL 10× CutSmart Buffer (NEB). Reactions were incubated at 37 °C for 30 min followed by heating to 80 °C for 3 min and slow cooling to 12 °C at 0.1 °C s^−1^. Constructs were directly transformed into One Shot Mach1 T1 Phage-Resistant Chemically Competent *E. coli* (Invitrogen) by heat shock for 30 s and streaked on 100 µg mL^−1^ carbenicillin containing LB agar. Selected colonies with correct construct were verified by Sanger sequencing and cultured overnight in 2× YT supplemented with 100 µg mL^−1^ carbenicillin. Plasmid was extracted by microcentrifuge membrane columns (QIAprep Spin Miniprep Kit, Qiagen) as per manufacturer’s instructions and quantified by Nanodrop.

### Site-directed mutagenesis of CypA, CypB, CypD (only for K175I mutant), and CypE

The K175I mutation for reduction of surface entropy^50^ was introduced into the PPIF (CypD) construct and the gatekeeper residue mutations were introduced into the PPIA (CypA), PPIB (CypB), and PPIE (CypE) constructs via Quikchange site-directed mutagenesis (Agilent Technologies). Primers for the respective mutations are included in Supplementary Table 4. The template plasmid (~100 ng, 1 µL) was combined in a 25 µL mixture with forward and reverse primers (125 ng, 1.25 µL each), Q5 High-Fidelity DNA Polymerase (NEB) (1 µL, 2000 U mL^−1^), deoxynucleoside triphosphate (dNTP) mix (10 mM, 1 µL), Q5 Reaction Buffer (NEB) (5×, 5 µL), and deionized H_2_O (14 µL). Reactions were incubated at 98 °C for 120 s, followed by 30 cycles of 98 °C (10 s, melting), 55–60 °C (30 s, annealing), and 72 °C (5 min, elongation), followed by a final elongation cycle of 7 min. PCR products were transformed into *E. coli* DH5α competent cells with a heat shock at 42 °C for 45 s and streaked onto Luria Broth agar plates containing 100 µg mL^−1^ ampicillin (2BT) or 50 µg mL^−1^ kanamycin (pET28α LIC). Single colonies were isolated for inoculation and plasmid extraction via microcentrifuge membrane columns (QIAprep Spin Miniprep Kit, Qiagen), and mutations were verified by Sanger sequencing.

### Recombinant expression and isolation of CypD and CypD mutants

CypD proteins were obtained from expression plasmids (Supplementary Table 5) by transforming into One Shot BL21(DE3) chemically competent *E. coli* (Invitrogen) by heat shock at 42 °C for 30 s. Cells were streaked onto agar plates containing 100 µg mL^−1^ carbenicillin and incubated at 37 °C for 16 h. Individual colonies were grown up in a 2-L culture of LB medium supplemented with 100 µg mL^−1^ carbenicillin at 37 °C until optical density reached 0.8. The culture was then cooled to 16 °C for 1 h and protein production was induced by adding 2 mL of 1 M isopropyl β-d-1-thiogalactopyranoside and left to incubate for 16 h. Cells were pelleted at 4,000*g* for 5 min at 4 °C and resuspended in 50 mL cold Tris-HCl pH 8.0, 50 mM NaCl, 5% glycerol (NiA Low Salt). Two Pierce protease inhibitor tablets (ThermoFisher Scientific) was added to the suspension and cells were subsequently lysed using an Avestin Emulsiflex-C3 homogenizer at 17,000–20,000 p.s.i. Lysed cells were pelleted, and supernatant was purified by FPLC affinity chromatography using a Histrap HP 5 mL (GE) and a gradient of 0–100% NiA low salt/NiB low salt (NiB low salt: NiA low salt + 500 mM imidazole). Protein eluted off around 60–70%, confirmed by SDS-PAGE analysis of fractions. Isolated fractions were additionally purified by FPLC cation exchange, using a HiTrap SP 5 ml column (GE) and a gradient of 0–100% SA buffer/SB buffer (SA buffer: Tris-HCl pH 7.0, 1 mM dithiothreitol (DTT), 5% glycerol; SB buffer: SA buffer + 1 M NaCl). Fractions corresponding to >90% pure CypD came off around 40%, confirmed by SDS-PAGE. Combined fractions were dialyzed in 20 mM Tris-HCl pH 8.0, 50 mM NaCl, 1 mM DTT, 5% glycerol, using a Slid-A-Lyzer molecular weight cutoff = 3,000 dialysis cassette according to the manufacturer’s instructions, overnight at 4 °C. Protein purity was >90% on the basis of SDS-PAGE gel electrophoresis and Coomassie staining. Pooled fractions were concentrated, flash frozen in liquid nitrogen, and stored at −80 °C. Protein was quantified using Pierce BCA Protein Assay Kit.

### Recombinant expression and purification of CypA, CypB, CypC, CypE, CypG, CypH, Cyp40, NKTR, PPIL1, PPIL2, PPIL3, PPWD1

The expression plasmids for wild-type and mutant protein constructs (Supplementary Table 5) were transformed into *E. coli* BL21(DE3) competent cells with heat shock at 42°C for 45 s. Cells were streaked onto Luria Broth agar plates containing 100 µg mL^−1^ ampicillin (2BT) or 50 µg mL^−1^ kanamycin (pET28α LIC) and incubated overnight at 37 °C. Single colonies were inoculated into 2× YT medium supplemented with 1% glucose, 1 mM Mg^2+^, and 100 µg mL^−1^ ampicillin (2BT) or 50 µg mL^−1^ kanamycin (pET28α LIC) and shaken at 37 °C until reaching an OD_600_ = ~0.6–0.8. The cultures were then cooled to 16 °C for 1 h, and protein production was induced with 1 mM isopropyl β-d-1-thiogalactopyranoside overnight for 16–18 h. Cells were pelleted at 4,000*g* for 10 min at 4 °C before being homogenized in an Avestin Emulsiflex-C3 High Pressure Homogenizer at 17,000–20,000 p.s.i three times and resuspended in buffer containing 20 mM Tris pH 8.0, 50 mM NaCl, and 5% glycerol. Lysates were centrifuged for 1 h at 17,000 r.p.m. at 4 °C in a Sorvall SLC6000 Fixed-Angle Rotor. Protein was purified from the supernatant via nickel affinity chromatography followed by cation exchange chromatography and size exclusion chromatography. First, the recombinant His_6_-tagged proteins were purified with Ni(II)-affinity chromatography (HisTrap FF, GE Healthcare). The supernatant was run over the column, which was subsequently washed twice with buffer to remove non-specific binding. Two-milliliter fractions were eluted over 12 column volumes by increasing the imidazole concentration to 500 mM. For PPIL2 and PPIL3, the His_6_-tag was cleaved with TEV (2BT) or thrombin (pET28α LIC) overnight in pH 8.0 dialysis buffer containing 20 mM Tris base, 100 mM NaCl, 5 mM BME, 5% glycerol with a 3 kDa molecular weight cutoff filter. Proteins both with or without His-tag were diluted to reduce NaCl concentration in pH 8.0 buffer containing 20 mM Tris, 1 mM DTT, and 5% glycerol and loaded onto a cation exchange column (HiTrap SP, GE Healthcare). The column was washed with six column volumes of buffer to remove non-specific binding. Two-milliliter fractions were eluted over 12 column volumes with increasing salt gradient up to 1 M NaCl. Pooled fractions containing protein were concentrated and loaded onto a size exclusion column (HiLoad 16/600 Superdex 200 prep grade, GE Healthcare). Protein was eluted in pH 7.3 buffer containing 50 mM NaH_2_PO_4_, 100 mM NaCl, and 2 mM EDTA. Protein purity was >90% on the basis of SDS-PAGE gel electrophoresis and Coomassie staining. Pooled fractions were concentrated, flash frozen in liquid nitrogen, and stored at −80 °C. Protein concentration was quantified using Pierce BCA Protein Assay Kit.

### Recombinant expression and purification of CypA E81S/K82R, CypB E121S, CypE (131–301) K212A, K217A, and K218A mutants

CypA, CypB, and CypE mutants were purified in a similar manner to wild-type with modification as indicated (see Supplementary Table 5 for expression plasmids). Following initial nickel affinity chromatography and overnight dialysis with thrombin-mediated His_6_-tag cleavage, the remaining protein was again run over a Ni(II)-affinity chromatography column (HisTrap FF, GE Healthcare) and flow-through and wash were collected to remove any unbound protein and His_6_ tags. Protein purity in flow-through and wash was >95% on the basis of SDS-PAGE gel electrophoresis and Coomassie staining. Pooled flow-through and wash was concentrated, flash frozen in liquid nitrogen, and stored at −80 °C. Protein concentration was quantified using Pierce BCA Protein Assay Kit.

### In vitro selection of a 256,000-member DNA-templated library with human His_6_-CypD

The selection protocol utilized a 256,000-member library and recombinant His_6_-CypD_45–__207_ adapted from previous work^38^. Resuspended magnetic Ni-NTA beads (DynabeadsHis-Tag Isolation and Pulldown, Invitrogen) (25 μL) were immobilized on a MagJET Separation Rack (ThermoFisher) with the supernatant subsequently removed. Beads were washed twice with 300 μL of 50 mM sodium phosphate pH 8.0, 300 mM NaCl, 0.01% Tween-20, 2 mM TCEP (Tris(2-carboxyethyl)phosphine) (PBST). Forty micrograms of CypD protein was loaded onto solid support in 300 μL PBST, incubated on rotary for 1 h at 4 °C, and washed twice with 200 μL of 50 mM Tris-HCl pH 8.0, 150 mM NaCl, 0.05% Tween-20, 2 mM TCEP (TBST). Immobilized protein was suspended in 100 μL blocking buffer (TBST + 0.1 mg mL^−1^ bovine serum albumin + 0.6 mg mL^−1^ yeast total RNA) for 25 min at 4 °C. After supernatant was removed, 20 pmol second-generation 256,000-member DTS library was suspended with bead immobilized protein in 50 µL blocking buffer and incubated for 1 h at 4 °C. Supernatant was removed (FT fraction) and beads were washed three times with TBST, retaining the supernatants (W1, W2, W3). CypD was eluted off Ni-NTA beads by incubating with 50 µL PBST supplemented with 300 mM imidazole (Elution Buffer) for 5 min. The elution was PCR amplified and barcoded with Illumina Primers as previously reported^38^. PCR amplicons were purified by PAGE, extracted, and quantified with KAPA quantitative PCR analysis and QuBit (Invitrogen). High-throughput DNA sequencing was performed on an Illumina MiSeq according to the manufacturer’s instructions for sample preparation. Reads generated were analyzed using the Python scripts (shared in Supplementary Note 1 of Supplementary Information) to quantify library barcodes and calculate change in percentage abundance for each library member compared to the pre-selection library.

### Chymotrypsin coupled prolyl-isomerase assay

Adapting a previously described protocol^51^, with an Agilent Bravo Velocity 11 with a 384ST head, 90 µL assay buffer (25 mM HEPES pH 7.3, 100 mM NaCl, 0.01% Triton X-100) containing 5.28 nM cyclophilin was added into a flat, clear-bottom, black 384-well plate pre-chilled in a Corning CoolBox at 2–3 °C. Five microliters of compound pre-dissolved in 5% DMSO/assay buffer was then added to appropriate wells and incubated at 2–3 °C for 5 min. Five microliters of 0.5 mM α-chymotrypsin from bovine pancreas (Type II, lyophilized powder, Millipore-Sigma) in 20% 1 mM HCl/Assay Buffer was then added to each well and incubated at 2–3 °C for 5 min. The plate was then quickly transferred to a FLIPR Tetra High-Throughput Cellular Screening System (Molecular Devices). Using the internal liquid handling system of the FLIPR, 1 µL 0.5 mM Suc-AAPF-AMC (Chymotrypsin Substrate II, Millipore-Sigma) dissolved in 0.55 M LiCl/2,2,2-trifluoroethanol was added to each well, which was then mixed for 5 s, followed by immediate fluorescence measurements every 1 s for 330 s using a 360–380 nm excitation LED module and a 400–460 nm emission filter. Final concentrations of the plate include 5 nM cyclophilin, 0.25% DMSO, 25 µM α-chymotrypsin, 5 µM Suc-AAPF-AMC. Raw fluorescence data was analyzed by non-linear regression analysis with Prism v.9 by fitting one-phase association curves to each well. Analyses were halted after the calculated maximum fluorescent value owing to observed fluorescent bleaching during the sustained fluorescent plateau. Rate constants calculated for each well (s^−1^) were then normalized to substrate only (no prolyl-isomerase) and enzyme + substrate only controls. IC_50_ values were calculated as the value at which 50% inhibition was achieved on the non-linear regression fitted curve of each compound.

### Anisotropy binding assay

Adapted from previous work^52^, titrated cyclophilin in assay buffer (25 mM HEPES pH 7.3, 100 mM NaCl, 0.01% Triton X-100) was incubated with 0.5 nM fluorescein-labeled macrocycle for 6 h at room temperature in a flat-bottom black untreated 96-well plate (Corning). Final assay volume was 100 µL with 0.25% DMSO. Fluorescence anisotropy was then measured using a Tecan Spark plate reader using 492 nm/523 nm excitation/emission settings. Raw fluorescence polarization data was analyzed by non-linear regression analysis with Prism v.9.3.1 by fitting to a one site-total binding equation, providing a dissociation constant (*K*_D_) for each cyclophilin–compound pair.

### Competition anisotropy binding assay with A26-Fl

Adapted from previous work^52^, cyclophilin in assay buffer (25 mM HEPES pH 8.0, 100 mM NaCl, 0.01% Triton X-100) at a pre-determined concentration from Supplementary Table 6 was incubated with 0.5 nM **A26-Fl** for 10 min at room temperature in a flat-bottom black untreated 96-well plate (Corning). Competitor macrocycle was then added to each well and incubated for 24 h. Final assay volume was 100 µL with 0.25% DMSO. Fluorescence anisotropy was then measured using a Tecan Spark plate reader using 492 nm/523 nm excitation/emission settings. Raw fluorescence polarization data was normalized to protein + fluorescent probe (0%) and buffer + fluorescent probe (100%) and *K*_i_ values were calculated using one site-competitive binding equation with Prism v.9.3.1, importing *K*_D_ values from **A26-Fl** (Extended Data Fig. 7).

### Surface plasmon resonance analysis of CypD ligands

Adapting a previously used protocol^53^, employing a Biacore T200 SPR, a Sensor S NTA (Biacore) chip was pre-loaded with 0.3 M NiCl_2_ and His_6_-CypD (50.24 nM, 1 µg mL^−1^) within a solution of 10 mM HEPES, 150 mM NaCl, 0.005% Tween-20 and 1 mM TCEP (surface plasmon resonance (SPR) buffer). Protein was covalently immobilized subsequently with injection of 200 mM 1-ethyl-3-(3-dimethylaminopropyl)carbodiimide (EDC) 50 mM *N*-hydroxysuccinimide (NHS). EDC–NHS-activated chip was quenched by flowing 1 M ethanolamine over the chip. Compounds were dissolved in 1% DMSO/SPR buffer and flowed over the SPR chip. Binding analysis was conducted from raw sensorgram resonance unit (RU) values, where individual replicates represent kinetic fitted RU value during time of compound administration at each dose. RU values were normalized to DMSO treatment and the maximum RU value of 200 µM JOMBt. These values were evaluated using Prism v.9 by fitting one site-specific binding to calculate *K*_D_ values.

### Assessment of covalent modification of CypE and other cyclophilin proteins

In PCR strips, CypE or other cyclophilin in assay buffer (25 mM HEPES pH 8.0, 100 mM NaCl, 0.01% Triton X-100) was incubated with compound for 1 h at room temperature. Final concentrations were 20 µM CypE, 100 µM compound, 0.5% DMSO, at a final volume of 20 µL. For analysis of lysine-iminoboronate modification, samples were directly submitted to Harvard’s Center for Mass Spectroscopy for LC–MS analysis. For amine-lysine modification, samples were treated with 5 μL of 125 mM sodium cyanoborohydride dissolved in 25 mM ammonium bicarbonate solution (pH 8.0) and incubated for 4 h at room temperature. For all other wild-type cyclophilins, protein was treated for 4 h with **C3A**, followed by 16 h treatment with NaCNBH_3_. The final concentration of NaCNBH_3_ was 25 mM. Samples were then submitted for LC–MS as described above.

### Co-crystallization of CypD K175I PPI domain (45–207) with macrocycle compounds

Macrocycles were incubated with 15 mg mL^−1^ CypD K175I (45–207) (**JOMBt**, **B1**, **B2**, **B3** at 1:3; **A26** at 1:1.5; **B21**, **B23**, **B25**, **B52**, **B53** at 1:2) for 15 min on ice. Crystals were obtained by mixing 1 µL of the protein–drug complex with 1 µL mother liquor (**JOMBt**: 19% PEG 3350, 0.5 M KH_2_PO_4_ pH 7.3; **A26**: 29% PEG 3350, 0.5 M KH_2_PO_4_ pH 7.3; **B1**: 23% PEG 3350, 0.5 M KH_2_PO_4_ pH 7.3; **B2**: 28% PEG 3350, 0.5 M KH_2_PO_4_ pH 7.3; **B3**: 28% PEG 3350, 0.5 M KH_2_PO_4_ pH 7.3; **B21**: 20% PEG 3350, 0.5 M KH_2_PO_4_ pH 7.3; **B23**: 21% PEG 3350, 0.5 M KH_2_PO_4_ pH 8.2; **B25**: 13% PEG 3350, 0.5 M KH_2_PO_4_ pH 8.0, 7.5% glycerol; **B52**: 15% PEG 3350, 0.5 M KH_2_PO_4_ pH 6.0, 1 mM NaCl; **B53**: 22.5% PEG 3350, 0.5 M KH_2_PO_4_ pH 6.0, 1 mM NaCl) and equilibrating against 1 mL of the same reservoir solution at room temperature. Large single rectangular crystals formed within 24 h and were equilibrated in a cryoprotective crystallography buffer composed of mother liquor containing 30% glycerol then snap frozen in liquid nitrogen. Diffraction data was collected from single crystals at 100 K at the FMX (CypD–**JOMBt**: *λ* = 0.97933 Å; CypD–**A26**: *λ* = 0.97933 Å; CypD–**B1**: *λ* = 0.97933 Å; CypD–**B2**: *λ* = 0.97933 Å; CypD–**B3**: *λ* = 0.97933 Å; CypD–**B21**: *λ* = 0.92016 Å; CypD–**B23**: *λ* = 0.97931 Å) and the AMX (CypD–**B25**: *λ* = 0.92011 Å; CypD–**B52**: *λ* = 0.92011 Å; CypD–**B53**: *λ* = 0.92011 Å) beamlines at the National Synchrotron Light Source II operated by Brookhaven National Laboratory at the indicated wavelengths.

### Crystal structure refinement

Diffraction data for **JOMBt**, **A26**, **B1**, **B2**, and **B3** was indexed, integrated, and scaled with autoPROC, and diffraction data for **B21**, **B23**, **B25**, **B52**, and **B53** was indexed, integrated, and scaled with FastDP. Both programs rely on additional functionality within XDS and CCP4. Phases were assigned via molecular replacement in Phaser^54^ with the apo structure of CypD K175I (PDB ID 2BIT) for CypD–JOMBt and subsequently with our previously solved structures of CypD K175I complexed with macrocyclic inhibitors as the search model. All refinements to the model were made in PHENIX^55^. Model building was performed in Coot^56^ with ligands and waters fit into the initial |*F*_o_| − |*F*_c_|. Macrocycle restraints were generated using eLBOW (**JOMBt**, **A26**, **B1**, **B2**, **B3**) and the ProDrg server^57^ (**B21**, **B23**, **B25**, **B52**, **B53**). The coordinates of the holo-structures of CypD have been deposited in the PDB. Additional crystallographic and data collection statistics are listed in Supplementary Table 9. Ligand electron densities are displayed in Supplementary Fig. 34.

### Molecular footprinting analysis

Molecular footprints, defined as per-residue decomposition of the Van der Waals, electrostatic, and hydrogen bonding energies between the ligand and the receptor, were generated with the crystal structures in the DOCK6.9 molecular modeling software as described by Balius et al.^58^. In brief, two crystal structures were structurally superimposed in UCSF Chimera on the basis of lowest pairwise root-mean square deviation using the Needleman–Wunsch alignment algorithm. Both the reference ligand and **B52** were saved in relation to the CypD–**B52** protein structure and were parameterized using the GAFF force field and the Gasteiger charging method. The ligands were then rigidly docked into the receptor using DOCK6.9, and the pairwise interaction energies for the top fifty contributing residues were visualized using matplotlib in Python.

### Ligand morph movie

The video morphing between the crystal structures of CypD K175I in its apo state (PDB: 2BIT) through **JOMBt**, **A26**, **B2**, **B23**, and **B52** was made in the molecular visualization software suite UCSF Chimera^59^. The six structures were structurally superimposed on the basis of lowest pairwise root-mean square deviation using the Needleman–Wunsch alignment algorithm and morphed with corkscrew interpolation at an interpolation rate of 150 steps between each conformation. Each crystallized ligand pose was saved as a separate Mol2 file in relation to the final trajectory structure. The trajectory was visualized with ligands using a per-frame script and recorded forwards and backwards.

### Isolation of mouse liver mitochondria

All procedures used in animal studies were approved by the Institutional Animal Care and Use Committee at Massachusetts General Hospital. Female C57BL/6J mice (Jackson Labs) age 10–12 weeks were anesthetized by isoflurane. The liver from one mouse was rinsed in ice-cold PBS, minced in ice-cold isolation buffer containing 0.28 M sucrose, 10 mM Hepes-KOH pH 7.2, 0.2 mM EDTA and 1% (w/v) bovine serum albumin, gently homogenized with four strokes of a tight-fitting Teflon pestle at 1,000 r.p.m., and then centrifuged for 10 min at 600*g* at 4 °C. The supernatant was recovered and centrifuged for 10 min at 8,000*g* at 4 °C. The loose outer buffy coat was rinsed off and the pellet resuspended gently in isolation buffer and the spins were repeated. The remaining buffer coat layer was rinsed off and the pellet resuspended in buffer containing 137 mM KCl, 10 mM Hepes-KOH pH 7.2, 2.5 mM MgCl_2_ for a final concentration between 40–80 mg mL^−1^ as assessed by Bradford. The suspension was kept on ice and all assays performed within 4–6 h following isolation. Quality control was done with every preparation by adding 250 mcg mitochondria to 500 µL assay buffer containing 137 mM KCl, 10 mM Hepes-KOH pH 7.2, 2.5 mM MgCl_2_, 5 mM each of glutamate and malate, and 3 mM KH_2_PO_4_. Sequential 150 µM ADP pulses were then delivered. Only preparations with respiratory control ratio >6 (as assessed by the ratio of ADP-coupled state 3 respiration to state 4 respiration) were used for further experiments. Measurements were made in a custom-built spectrometer.

### Mitochondrial calcium retention capacity assays

Two hundred and fifty micrograms of mouse liver mitochondria isolated as above were added to 500 µL assay buffer containing 125 mM KCl, 20 mM Hepes-KOH pH 7.2, 1 mM MgCl_2_, 5 mM each of glutamate and malate, and 3 mM KH_2_PO_4_. 0.5 µM Calcium Green 5N (Molecular Probes) was included to monitor extramitochondrial free Ca^2+^. Fluorescence was continuously monitored in the custom-built fluorimeter described above, with excitation 470 nm and emission 520–560 nm. Sequential 60 µM CaCl_2_ pulses were delivered until Ca^2+^ uptake ceased and an abrupt release of previously taken up Ca^2+^ was observed, consistent with mPTP opening. The calcium retention capacity ratio was obtained by normalizing the number of Ca^2+^ pulses that could be taken up in a given condition by that taken up under DMSO control conditions, as previously described^60^. Student’s *t*-test was used to determine statistical significance between predefined comparisons (DMSO versus CsA, inactive versus active enantiomers of **B52-Cy5**, and inactive versus active enantiomers of **B53-Cy5**).

### Fluorescence microscopy on Hela cells

Cells were seeded into 96-well, black, clear-bottom, TC-treated plates, in DMEM supplemented with 10% FBS 24 h prior at a density where wells were ~80% confluent at the time of the experiment. Medium was removed and cells were then stained with 50 µL mixture containing Cy5-labeled compound (6 µM) in Kreb’s ringer solution HEPES buffered pH 7.2 (KRB), for 1 h at 37 °C. Then, 50 µL Hoechst 33342 (8 µM) and Mitotracker Green FM (0.1 µM) in KRB was added directly to wells containing cells and incubated for 15 min at 37 °C. Medium was removed and cells were washed twice with 100 µL KRB. Cells were then kept adherent to the wells in 100 µL KRB for imaging. Fluorescence microscopy was conducted on an Opera Phenix Plus High-Content Screening System, using Alexa 488, Alexa 647, and Hoescht 33342 channels. Images were analyzed using Harmony v.4.9.

### In vitro esterase activity on ester pro-drug Cy5 derivatives of CypD inhibitors

In PCR tubes, ester compound (6 µM) and either buffer only, CES1 (0.25 µM), or CES2 (0.25 µM) was diluted in 100 mM Tris-HCl buffer, pH 7.4, with a final DMSO concentration of 1%. Samples were maintained on a PCR block at 37 °C for 8 h. After incubation, samples were diluted with 20 µL acetonitrile and analyzed by LC–MS at Harvard’s Center for Mass Spectroscopy. Di-ester, mono-ester, and di-acid abundances were quantified by total ion count of the primary isotope and the three compounds were summed and each one expressed as a fraction of the total sum.

### Cellular esterase activity on ester pro-drug Cy5 derivatives of CypD inhibitors

Cells were seeded in 96-well, clear, flat-bottom, TC-treated plates in DMEM supplemented with 10% FBS for HEK293T, HeLa, and MEFs, F-12K supplemented with 10% FBS for A549, or MEM supplemented with 10% FBS for HepG2. Cells were seeded 24 h prior at a density where wells were ~70% confluent at the time of the experiment. Medium was removed and cells were incubated with 100 µL mixture containing each Cy5-conjugated compound (6 µM) in their respective medium at 1% DMSO for 48 h (36 h for HepG2) at 37 °C at 5% CO_2_. Supernatant was removed, and cells were washed twice with 100 µL PBS pH 7.4 (Gibco). Cells were then lysed for 15 min with 30 µL 10 mM Tris-HCl pH 8.0 buffer containing 0.05% SDS. Lysates were diluted with 100 µL acetonitrile and filtered. Lysates were analyzed by LC–MS at Harvard’s Center for Mass Spectroscopy. Di-ester, mono-ester, and di-acid abundances were quantified by total ion count of the primary isotope, and the three compounds were summed and each one expressed as a fraction of the total sum.

### paxdb^4.1^ quantification of human cyclophilin abundance

Relative human cyclophilin abundances were calculated using paxdb^4.1^, filtered for *Homo sapiens* and the gene identifier of each cyclophilin (Supplementary Table 1). Parts per million values were reported from calculated whole organism (integrated) abundance.

### Reporting summary

Further information on research design is available in the Nature Research Reporting Summary linked to this article.

## Online content

Any methods, additional references, Nature Research reporting summaries, source data, extended data, supplementary information, acknowledgements, peer review information; details of author contributions and competing interests; and statements of data and code availability are available at 10.1038/s41589-022-01116-1.

## Supplementary information

Supplementary Information Supplementary Tables 1–9, Supplementary Figs 1–35, Supplementary Notes 1 and 2.

Reporting Summary

Supplementary Video 1 Video of ligand-dependent CypD S2 pocket conformational changes. The video progresses between individual morphed transitions in the following order: apo-CypD (PDB: 2BIT) → CypD–JOMBt→ CypD–A26 → CypD–B2 → CypD–B23 → CypD–B52, with interspersed still images depicting the protein–ligand hydrogen bonds in black. Key CypD residues are colored as follows: active site residues (red), S2 pocket residues (green), K118 (orange), primary gatekeeper residues (magenta). Ligands are shown in light blue.

Supplementary Data 1 Table of prolyl-isomerase inhibition IC_50_ vales for reported compounds.

## Data Availability

High-throughput sequencing data for both replicates of His_6_-CypD selection and pre-selection library using a 256,000-member DNA-templated library are available on the NCBI Sequence Read Archive under accession PRJNA797008. The X-ray co-crystal structures of CypD–JOMBt, CypD–**A26**, CypD–**B1**, CypD–**B2**, CypD–**B3**, CypD–**B21**, CypD–**B23**, CypD–**B25**, CypD–**B52**, CypD–**B53** are available on the PDB (IDs 7TGS, 7TGT, 7TGU, 7TGV, 7TH1, 7TH6, 7TH7, 7THC, 7THD, 7THF, respectively). The CypD + CsA co-crystal structure used is PDB 2Z6W. Human cyclophilin abundance was calculated using paxdb^4.1^ (https://www.pax-db.org). Source data are provided with this paper.

## Source data

Source Data Fig. 1 Prolyl-isomerase inhibition biochemical data.
Source Data Fig. 2 Prolyl-isomerase inhibition biochemical data.
Source Data Fig. 3 Prolyl-isomerase inhibition biochemical data.
Source Data Fig. 4 Prolyl-isomerase inhibition biochemical data.
Source Data Fig. 5 Mitochondrial calcium retention data.
Source Data Fig. 6 Prolyl-isomerase inhibition and fluorescence polarization biochemical data.
Source Data Extended Data Fig. 1 Prolyl-isomerase inhibition biochemical data.
Source Data Extended Data Fig. 2 DNA-templated library selection enrichment data.
Source Data Extended Data Fig. 6 Prolyl-isomerase inhibition biochemical data.
Source Data Extended Data Fig. 7 Fluorescence polarization biochemical data.
Source Data Extended Data Fig. 8 Fluorescence microscopy data.
Source Data Extended Data Fig. 9 Pro-drug esterase cleavage data.

## Code availability

Custom python scripts for analyzing selection data are provided in Supplementary Note 1 within the Supplementary Information. UCSF DOCK6.9 was used for molecular footprinting analyses. Crystal structures solved using the Phenix Software Suite (v.1.171-3660). Structures analyzed in PyMOL (v.2.5.2) and UCSF Chimera (v.1.16).
