# Supplementary material for: Discovery and molecular basis of subtype-selective cyclophilin inhibitors
Source: Nat Chem Biol. 2022 Sep 26;18(11):1184–95. doi: 10.1038/s41589-022-01116-1 (PMC9596378; doi:10.1038/s41589-022-01116-1)
Supplement: Supplementary file 1 — Supplementary Tables 1–9, Supplementary Figs 1–35, Supplementary Notes 1 and 2. [file 41589_2022_1116_MOESM1_ESM.pdf]

nature portfolio

https://doi.org/10.1038/s41589-022-01116-1

Supplementary information

Discovery and molecular basis of subtype-selective cyclophilin inhibitors

In the format provided by the authors and unedited

Supplementary Information

Discovery and molecular basis of subtype-selective cyclophilin inhibitors

Alexander A. Peterson, Aziz M. Rangwala, Manish K. Thakur, Patrick S. Ward, Christie Hung, Ian R. Outhwaite, Alix I. Chan, Dmitry L. Usanov, Vamsi K. Mootha, Markus A. Seeliger*, David R. Liu*

* Correspondence: drliu@fas.harvard.edu, markus.seeliger@stonybrook.edu

Supplementary Table 1 | Calculated abundance of each human cyclophilin family member from paxdb[4.1]

Supplementary Table 2 | Overview of all cyclophilin-selective inhibitors reported in this work

Supplementary Table 3 | Plasmids and primers used for USER cloning of CypD mutant expression constructs

Supplementary Table 4 | Plasmids and primers used for cloning of CypA, CypB, and CypE mutant expression constructs

Supplementary Table 5 | Plasmids used for recombinant expression of cyclophilin proteins

Supplementary Table 6 | Concentrations of cyclophilins used in competition anisotropy binding assay with A26-Fl

Supplementary Table 7 | High-resolution mass spectroscopy results for intermediates reported in this work

Supplementary Table 8 | High-resolution mass spectroscopy results for macrocycles reported in this work

Supplementary Table 9 | Crystal diffraction statistics for all CypD-inhibitor co-crystal structures reported in this work

Supplementary Figure 1 | Cyclosporine A interactions with active site residues of CypD

Supplementary Figure 2 | Surface plasmon resonance measurement of JOMBt and JOBBt binding to immobilized CypD

Supplementary Figure 3 | Prolyl isomerase activity of cyclophilins on Suc-AAPF-AMC

Supplementary Figure 4 | Functionalization of piperidine α-carbon improves CypD potency, while replacing the second building block improves CypD selectivity

Supplementary Figure 5 | Large S2 binding groups improve selectivity for CypD

Supplementary Figure 6 | Large hydrophobic S2 binding groups impart selectivity for CypD over CypG, CypH, NKTR, and PPWD1

Supplementary Figure 7 | Biphenyl and bi-aromatic derivatives of B2 showing no improvement in selectivity for CypD

Supplementary Figure 8 | Para-carbonyl-containing biphenyl moieties show partial selectivity over CypC

Supplementary Figure 9 | Para-carboxy-containing biphenyl groups provide enhanced selectivity and potency for CypD

Supplementary Figure 10 | Masking or moving the carboxylate of B23 results in loss in potency and selectivity for CypD

Supplementary Figure 11 | Inverting ligand charge abrogates CypD potency and shifts the cyclophilin selectivity profile

Supplementary Figure 12 | Nitrile-containing biphenyl groups show similar CypD inhibition potencies and linker dependencies as carboxylates

Supplementary Figure 13 | Residue K118 is necessary for potent CypD inhibition with carboxylate-containing inhibitors

Supplementary Figure 14 | S123 gatekeeper position dictates inhibitor potency

Supplementary Figure 15 | Prolyl isomerase inhibition activity on CypD gatekeeper mutants

Supplementary Figure 16 | Derivatives of B23 that show similar selectivity and potency profiles for CypD

Supplementary Figure 17 | Derivatives of B23 that show reduced potency and selectivity for CypD

Supplementary Figure 18 | B23 derivatives that can simultaneously interact with CypD residues K118 and S119 and present a carboxylate near gatekeeper residues

Supplementary Figure 19 | The CypB E121S gatekeeper mutant is inhibited more potently compared to wild-type CypB by carboxylate-containing inhibitors

Supplementary Figure 20 | The CypA E81S/K82R gatekeeper mutant is inhibited more potently compared to wild-type CypA with carboxylate-containing inhibitors

Supplementary Figure 22 | Prolyl isomerase inhibition by CypD-selective inhibitors used in mitochondrial models of mPTP

Supplementary Figure 23 | Additional independent replicates of calcium retention assays in mitochondria

Supplementary Figure 24 | Representative images of Cy5-conjugated CypD inhibitor localization in HeLa cells

Supplementary Figure 25 | CypD inhibition of Cy5-conjugated CypD inhibitors

Supplementary Figure 26 | Fluorescence polarization competition with A26-Fl against lysine-containing cyclophilins

Supplementary Figure 27 | Fluorescence polarization competition with A26-Fl against CypE with control compounds C5A and C6A

Supplementary Figure 28 | Prolyl isomerase inhibition by C1A, C3A, C5A, and C6A

Supplementary Figure 29 | Mass spectroscopy analysis of C3A covalent modification on 13 cyclophilins

Supplementary Figure 30 | CypE mutant FP analysis

Supplementary Figure 31 | Prolyl isomerase inhibition screening of CypE lysine mutants reveals K217 residue as site of C3A covalent modification

Supplementary Figure 32 | Ligand-dependent conformation of primary gatekeeper residues S123 and R124 in the S2 pocket

Supplementary Figure 33 | Molecular footprinting analysis demonstrates that key binding interactions of B52 are unique to CypD

Supplementary Figure 34 | Electron densities of ligands from CypD-inhibitor co-crystal structures

Supplementary Figure 35 | SDS-PAGE of recombinantly expressed cyclophilin proteins

Supplementary Note 1:
Python Scripts
- Analysis of CypD selection high-throughput sequencing data
- Calculating enrichment values of library members

Supplementary Note 2:
Synthetic Methods
- Generalized procedures
- Synthesis of intermediates
- Synthesis of final macrocycles

^{1}H-NMR Spectra
HPLC Chromatograms
Supplementary References

Supplementary Table 1 | Calculated abundance of each human cyclophilin family member from paxdb[4.1]. CypD is the third most abundant cyclophilin, while CypB and CypA are the second and first, respectively (see Wang, M., Herrmann, C. J., Simonovic, M., Szklarczyk, D. & von Mering, C. Version 4.0 of PaxDb: Protein abundance data, integrated across model organisms, tissues, and cell-lines. *Proteomics* **15**, 3163–3168 (2015)). CypA is one of the most abundantly expressed proteins in human cells. Therefore, selectivity over CypA is the most important for CypD selective inhibition in biological models. Compounds **B52/B53**, **B32**, and **C3A** *in vitro* show >100-fold selectivity for CypD, CypD K118E/R124A, and CypE, respectively, over CypA.

Cyclophilin	Gene	Protein abundance: H.Sapiens whole integrated (ppm)	Normalized CypD abundance
**CypD**	**PPIF**	187	1
**CypA**	**PPIA**	4317	23.1
**CypB**	**PPIB**	248	1.33
**CypC**	**PPIC**	0.226	0.00121
**Cyp40**	**PPID**	13.5	0.0722
**CypE**	**PPIE**	61.7	0.330
**CypG**	**PPIG**	24.2	0.129
**CypH**	**PPIH**	11.9	0.0636
**PPIL1**	**PPIL1**	17.8	0.0952
**PPIL2**	**PPIL2**	3.14	0.0168
**CypJ/PPIL3**	**PPIL3**	4.62	0.0247
**PPIL4**	**PPIL4**	2.14	0.0114
**PPIL6**	**PPIL6**	0.167	0.000893
**PPWD1**	**PPWD1**	29.2	0.156
**RanBP2**	**RanBP2**	35.5	0.190
**SDCCAG**	**CWC27**	1.82	0.00973
**NKTR**	**NKTR**	0.108	0.000577

Supplementary Table 2 | Overview of all cyclophilin-selective inhibitors reported in this work. Inhibitor IC_{50} values from prolyl-isomerase inhibition on the targeted cyclophilin are highlighted in dashed boxes. The inhibition potencies against all other cyclophilins are normalized to this IC_{50} value and are shown as a fold-difference. Boxes are shaded according to the fold-difference value. Each cyclophilin is inhibited selectively due to the identity of its unique S2 pocket residues and the S2 pocket binding moiety of the inhibitor.

No S2 binding

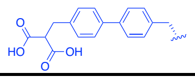

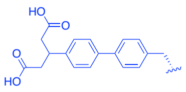

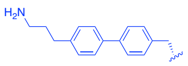

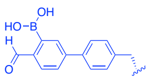

Non-selective	CypD selective		CypD K118E/R124A selective	CypE selective	
**CsA**	**B52**	**B53**	**B32**	**C3A**	**Residues proximal to biphenyl**
13 nM (1x)	10 nM (1x)	57 nM (1x)	100x	46x	CypD (Lys, Ser, Arg)
2x	31x	14x	120x	13 nM (1x)	CypE (Lys, Lys, Lys)
1x	21x	19x	50x	310x	CypB (Lys, Glu, Arg)
1x	110x	60x	130x	230x	CypA (Lys, Glu, Lys)
2x	600x	750x	33x	31x	PPIL1 (Ala, Lys, Gln)
11x	2200x	>900x	67x	140x	Cyp40 (Glu, Glu, Lys)
1x	>10000x	>900x	20x	1300x	CypC (Val, Glu, Thr)
2x	>10000x	>900x	180x	3100x	CypG (Glu, Gly, Phe)
15x	>10000x	>900x	13x	620x	PPWD1 (Glu, Gly, Glu)
6x	>10000x	>900x	>830x	>3800x	CypH (Ala, Gly, Pro)
38x	>10000x	>900x	>830x	2500x	NKTR (Glu, Gly, Tyr)
			60 nM (1x)		CypD K118E/R124A (Glu, Ser, Ala)
Reported non-binder for PPIL2, PPIL6, SDCCAG-10. Unknown for PPIL3, PPIL4.	No active site binding by fluorescence polarization **B52-Fl**.	No active site binding by fluorescence polarization **B53-Fl.**	Predicted weak binding due to weak potency of **A26-Fl** by fluorescence polarization, which has poor S2-pocket occupancy		PPIL2, PPIL3, PPIL4, PPIL6, SDCCAG-10

Color Gradient: 100.00 90.00 80.00 70.00 60.00 50.00 40.00 30.00 20.00 10.00 9.00 8.00 7.00 6.00 5.00 4.00 3.00 2.00 1.00

Formatting Rules: Red ≥100x Gray=10x Green ≤1x

Supplementary Table 3 | Plasmids and primers used for USER cloning of CypD mutant expression constructs.

Mutation (plasmid name)	Starting plasmid	Primer (forward, reverse)
CypD K118A (2BT-CypD (45-207) K118A)	2BT-CypD (45-207)	AGGCGGGGCG/ideoxyU/CCATCTACGGAAGCCGCTTTC<br>ACGCCCCGCC/ideoxyU/GTGCCATTGTGGTTGGTG
CypD K118E (2BT-CypD (45-207) K118E)	2BT-CypD (45-207)	AGGCGGGGAA/ideoxyU/CCATCTACGGAAGCCGCTTTCC<br>ATTCCCCGCC/ideoxyU/GTGCCATTGTGGTTGGTG
CypD S123E (2BT-CypD (45-207) S123E)	2BT-CypD (45-207)	ACGGAGAACGC/ideoxyU/TTCCTGACGAGAACTTTAC<br>AGCGTTCTCCG/ideoxyU/AGATGGACTTCCCG
CypD R124A (2BT-CypD (45-207) R124A)	2BT-CypD (45-207)	AGCGCGTTTCC/ideoxyU/GACGAGAACTTTACACTGAAG<br>AGGAAACGCGC/ideoxyU/TCCGTAGATGGACTTCC
CypD R124K (2BT-CypD (45-207) R124K)	2BT-CypD (45-207)	AGCAAATTTCC/ideoxyU/GACGAGAACTTTACACTGAAG<br>AGGAAATTTGC/ideoxyU/TCCGTAGATGGACTTC
CypD K118E/R124A (2BT-CypD (45-207) K118E/R124A)	2BT-CypD-R124A (45-207)	AGCGCGTTTCC/ideoxyU/GACGAGAACTTTACACTGAAG<br>AGGAAACGCGC/ideoxyU/TCCGTAGATGGATTCCC

Supplementary Table 4 | Plasmids and primers used for cloning of CypA, CypB, and CypE mutant expression constructs.

Mutation (plasmid name)	Starting plasmid	Primer (forward, reverse)
CypD K175I (2BT-CypD (45-207) K175I)	2BT-CypD (45-207)	5'-GTTCGGTCACGTCATAGAGGGCATGGAC-3'<br>5'-GTCCATGCCCTCTATGACGTGACCGAAC-3'
CypB E121S (2BT-CypB (34-216) E121S)	2BT-CypB (34-216)	5'-GGAAAGAGCATCTACGGTAGCCGCTTCCCCGATGAG-3'<br>5'-CTCATCGGGGAAGCGGCTACCGTAGATGCTCTTTCC-3'
CypA K82R (2BT-CypA (1-165) K82R)	2BT-CypA (1-165)	5'-GCAAGTCCATCTATGGGGAGCGCTTTGAAGATGAGAACTTCATCC-3'<br>5'-GGATGAAGTTCTCATCTTCAAAGCGCTCCCCATAGATGGACTTGC-3'
CypA E81S/K82R (2BT-CypA (1-165) E81S/K82R)	2BT-CypA K82R (1-165)	5'-GCAAGTCCATCTATGGGAGCCGCTTTGAAGATGAGAACTTCATCC-3'<br>5'-GGATGAAGTTCTCATCTTCAAAGCGGCTCCCATAGATGGACTTGC-3'
CypE K212A (pET28α LIC-CypE (131-301) K212A)	pET28α LIC-CypE (131-301)	5'-CCACAATGGCACTGGGGGCGCGTCCATCTATGGGAAG-3'<br>5'-CGAACTTCTTCCCATAGATGGACGCGCCCCCAGTGCCATTG-3'
CypE K217A (pET28α LIC-CypE (131-301) K217A)	pET28α LIC-CypE (131-301)	5'-GGGCAAGTCCATCTATGGGGCGAAGTTCGATGATG-3'<br>5'-GTTTTCATCATCGAACTTCGCCCCATAGATGGAC-3'
CypE K218A (pET28α LIC-CypE (131-301) K218A)	pET28α LIC-CypE (131-301)	5'-GCAAGTCCATCTATGGGAAGGCGTTCGATGATGAAAAC-3'<br>5'-GGATAAAGTTTTCATCATCGAACGCCTTCCCATAGATGG-3'

Supplementary Table 5 | Plasmids used for recombinant expression of cyclophilin proteins.

Expression plasmid	Cyclophilin protein
2BT-CypD (45-207)	CypD
2BT-CypD (45-207) K118A	CypD K118A
2BT-CypD (45-207) K118E	CypD K118E
2BT-CypD (45-207) S123E	CypD S123E
2BT-CypD (45-207) R124A	CypD R124A
2BT-CypD (45-207) R124K	CypD R124K
2BT-CypD (45-207) K118E/R124A	CypD K118E/R124A
2BT-CypD (45-207) K175I	CypD K175I
2BT-CypA (1-165)	CypA
2BT-CypA (1-165) K82R	CypA K82R
2BT-CypA (1-165) E81S/K82R	CypA E81S/K82R
2BT-CypB (34-216)	CypB
2BT-CypB (34-216) E121S	CypB E121S
pET28α LIC-CypC (24-212)	CypC
pET28α LIC-CypE (131-301)	CypE
pET28α LIC-CypE (131-301) K212A	CypE K212A
pET28α LIC-CypE (131-301) K217A	CypE K217A
pET28α LIC-CypE (131-301) K218A	CypE K218A
pET28α LIC-CypG (1-179)	CypG
2BT-CypH (1-177)	CypH
2BT-Cyp40 (1-183)	Cyp40
pET28α LIC-NKTR (7-179)	NKTR
2BT-PPIL1 (1-166)	PPIL1
pET28α LIC-PPIL2 (280-457)	PPIL2
2BT-PPIL3 (1-161)	PPIL3
pET28a LIC-PPWD1 (473-646)	PPWD1

Supplementary Table 6 | Concentrations of cyclophilins used in competition anisotropy binding assay with A26-Fl.

Cyclophilin	Concentration (nM)
CypD	40
CypA	80
CypB	100
CypE	150
CypE K212A	150
CypE K217A	150
CypE K218A	150
Cyp40	300
PPIL1	600
PPIL3	2000

Supplementary Table 7 | High-resolution mass spectroscopy results for intermediates reported in this work. Experiments and formula confirmation were performed by Harvard University's Center for Mass Spectroscopy.

Compound ID	Molecular Formula	Calculated [M+H]$^+$	Found [M+H]$^+$	Calculated Δ ppm
4F	$C_{23}H_{24}N_2O_6$	425.171	425.1707	-0.7
I1	$C_{21}H_{19}NO_4$	348.1241	348.1245	1.1
I2	$C_{21}H_{19}NO_4$	348.1241	348.1245	1.1
I3	$C_{20}H_{19}NO_4$	336.1241	336.1246	1.5
I4	$C_{22}H_{23}NO_4$	366.17	366.1701	0.3
I5	$C_{29}H_{24}N_2O_4$	465.1809	465.1811	0.4
I6a	$C_{19}H_{25}BO_4$	329.1919	329.1921	0.6
I6b	$C_{13}H_{15}BO_4$	245.0991	245.099	-0.4
I7a	$C_{16}H_{21}BO_5$	303.1409	303.1411	0.7
I7b	$C_{10}H_{11}BO_5$	221.0627	221.0625	-0.9
I8a	$C_{10}H_9BO_5$	219.047	219.0469	-0.5
I8b	$C_9H_7BO_5$	205.0314	205.031	-2
I9a	$C_{12}H_{11}F_3O_6S$	341.0301	341.0304	0.9
I9b	$C_{17}H_{23}BO_5$	341.1531	341.1526	-1.5
I9c	$C_{11}H_{13}BO_5$	235.0783	235.0783	0
I9d	$C_{10}H_{11}BO_5$	221.0627	221.0626	-0.5
I10	$C_9H_8BNO_4$	204.0474	204.047	-2
I11	$C_{14}H_{17}BO_5$	275.1096	275.1098	0.7
I12a	$C_{16}H_{23}BO_4$	289.1617	289.1619	0.7
I12b	$C_{10}H_{13}BO_4$	207.0834	207.0832	-1
I13a	$C_{16}H_{21}BO_4$	287.146	287.1461	0.3
I13b	$C_{16}H_{21}BO_4$	205.0678	205.0675	-1.5
I14a	$C_{12}H_{15}BrO_4S$	356.9767	356.9769	0.6
I14b	$C_{18}H_{27}BO_6S$	405.1514	405.1512	-0.5
I14c	$C_{12}H_{17}BO_6S$	323.0731	323.0734	0.9
I15a	$C_{11}H_{11}F_3O_6S$	351.0121	351.0124	0.9
I15b	$C_{16}H_{23}BO_5$	329.1531	329.1531	0
I15c	$C_{10}H_{13}BO_5$	223.0783	223.0782	-0.4
I15d	$C_9H_{11}BO_5$	209.0627	209.0624	-1.4
I16a	$C_{15}H_{21}BO_5$	291.1409	291.1413	1.4
I16b	$C_9H_{11}BO_5$	209.0627	209.0624	-1.4
I17a	$C_{11}H_9BrO_3$	268.9808	268.9809	0.4
I17b	$C_{17}H_{21}BO_5$	317.1555	317.1558	0.9
I17c	$C_{11}H_{11}BO_5$	235.0772	235.0768	-1.7
I17d	$C_9H_7BO_5$	205.0314	205.0311	-1.5
I18a	$C_{17}H_{21}BO_5$	317.1555	317.1557	0.6
I18b	$C_{11}H_{11}BO_5$	235.0772	235.0766	-2.6
I18c	$C_9H_7BO_5$	205.0314	205.0311	-1.5
I19a	$C_{12}H_{12}O_4$	221.0808	221.081	0.9
I19b	$C_{13}H_{11}F_3O_6S$	375.0121	375.0123	0.5
I19c	$C_{18}H_{23}BO_5$	353.1531	353.1532	0.3
I19d	$C_{12}H_{13}BO_5$	247.0783	247.0782	-0.4
I19e	$C_{10}H_9BO_5$	219.047	219.0469	-0.5
I20a	$C_{12}H_{11}F_3O_6S$	341.0301	341.0309	2.3
I20b	$C_{17}H_{23}BO_5$	341.1531	341.1526	-1.5
I20c	$C_{11}H_{13}BO_5$	235.0783	235.0783	0
I20d	$C_{10}H_{11}BO_5$	221.0627	221.0626	-0.5
I21a	$C_{16}H_{21}BO_4$	287.146	287.1463	1
I21b	$C_{10}H_{11}BO_4$	205.0678	205.0676	-1
I22a	$C_{16}H_{20}BNO_4$	324.1378	324.138	0.6
I22b	$C_{10}H_{10}BNO_4$	218.063	218.0628	-0.9
I22c	$C_9H_8BNO_4$	204.0474	204.047	-2
I23	$C_{18}H_{27}BO_6$	373.1793	373.1792	-0.3
I24a	$C_{19}H_{27}BrO_4$	421.0985	421.0986	0.2
I24b	$C_{25}H_{39}BO_6$	469.2732	469.2732	0
I24c	$C_{19}H_{29}BO_6$	387.1949	387.1949	0
I25a	$C_{23}H_{26}BrNO_5$	476.1067	476.1067	0
I25b	$C_{32}H_{26}BrNO_5$	606.0887	606.088	-1.2
I25c	$C_{47}H_{49}BrN_6O_7$	911.2738 (Na$^+$ adduct)	911.2718	-2.2
I26a	$C_{23}H_{26}BrNO_5$	476.1067	476.1061	-1.3
I26b	$C_{32}H_{26}BrNO_5$	606.0887	606.0877	-1.6
I26c	$C_{47}H_{49}BrN_6O_7$	911.2738 (Na$^+$ adduct)	911.2715	-2.5
I27a	$C_{22}H_{24}BrNO_5$	484.073	484.0728	-0.4
I27b	$C_{31}H_{24}BrNO_5$	592.073	592.0724	-1
I27c	$C_{46}H_{47}BrN_6O_7$	875.2762	875.2757	-0.6
I28a	$C_{22}H_{24}BrNO_5$	484.073	484.0725	-1
I28b	$C_{31}H_{24}BrNO_5$	592.073	592.0727	-0.5
I28c	$C_{46}H_{47}BrN_6O_7$	875.2762	875.2753	-1
I29a	$C_{20}H_{29}BO_6$	399.1949	399.1951	0.5
I29b	$C_{14}H_{19}BO_6$	317.1167	317.117	0.9
I30a	$C_{15}H_{19}BrO_4$	365.0359	365.0359	0.0
I30b	$C_{21}H_{31}BO_6$	413.2106	413.2106	0.0
I30c	$C_{15}H_{21}BO_6$	331.1323	331.1327	1.2
(4Br)B6	$C_{43}H_{52}BrN_7O_7$	858.3184	858.3193	1
(4Br)B6-A	$C_{39}H_{43}BrN_6O_6$	771.25	771.25	0
(4Br)B6-B	$C_{41}H_{48}BrN_7O_6$	814.2922	814.2908	-1.7
(4Br)B6-FI	$C_{39}H_{43}BrN_6O_6$	1332.4499	1332.4489	-0.8
*(4Br)B6-A	$C_{39}H_{43}BrN_6O_6$	771.25	771.25	0
*(4Br)B6-B	$C_{41}H_{48}BrN_7O_6$	814.2922	814.291	-1.5

Supplementary Table 8 | High-resolution mass spectroscopy results for macrocycles reported in this work. Experiments and formula confirmation were performed by Harvard University's Center for Mass Spectroscopy.

Compound ID	Molecular Formula	Calculated $[M+H]^+$	Found $[M+H]^+$	Calculated Δ ppm
JOMBt	$C_{34}H_{45}N_7O_8$	680.3402	680.3398	-0.6
JOMBc	$C_{34}H_{45}N_7O_8$	680.3402	680.3404	0.3
JOMBt-A	$C_{30}H_{36}N_6O_7$	593.2718	593.2721	0.5
JOMBt-D	$C_{40}H_{57}N_7O_{10}$	796.424	796.4238	-0.3
JOBBt	$C_{35}H_{47}N_7O_8$	694.3559	694.3556	-0.4
JOBBc-A	$C_{31}H_{38}N_6O_7$	607.2875	607.2874	-0.2
JOGAt	$C_{34}H_{45}N_7O_8$	680.3402	680.3403	0.1
JOGAc	$C_{34}H_{45}N_7O_8$	680.3402	680.3405	0.4
JOGBt-A	$C_{31}H_{38}N_6O_7$	607.2875	607.2875	0
JOGBc-A	$C_{31}H_{38}N_6O_7$	607.2875	607.2876	0.2
JOCBt	$C_{34}H_{45}N_7O_8$	680.3402	680.3402	0
JOCBc	$C_{34}H_{45}N_7O_8$	680.3402	680.3403	0.1
JOMAt	$C_{33}H_{43}N_7O_8$	666.3246	666.324	-0.9
JOMAc	$C_{33}H_{43}N_7O_8$	666.3246	666.3246	0
JOMCt	$C_{35}H_{47}N_7O$	694.3559	694.3559	0
JOMCc	$C_{35}H_{47}N_7O$	694.3559	694.356	0.1
JOMFt	$C_{34}H_{45}N_7O_8$	680.3402	680.3403	0.1
JOMFc	$C_{34}H_{45}N_7O_8$	680.3402	680.3399	-0.4
HOJJt	$C_{37}H_{51}N_7O_8$	722.3872	722.387	-0.3
HOJJc	$C_{37}H_{51}N_7O_8$	722.3872	722.3867	-0.7
HJJJt	$C_{39}H_{59}N_7O_7$	738.4549	738.4544	-0.7
HJJJc	$C_{39}H_{59}N_7O_7$	738.4549	738.4543	-0.8
A1	$C_{33}H_{43}N_7O_8$	666.3246	666.3246	0.0
A2	$C_{33}H_{43}N_7O_8$	666.3246	666.3244	-0.3
A3	$C_{30}H_{45}N_7O_8$	632.3402	632.3404	0.3
A4	$C_{31}H_{43}N_7O_8$	642.3246	642.3245	-0.2
A5	$C_{31}H_{43}N_7O_8$	642.3246	642.3245	-0.2
A6	$C_{32}H_{41}N_7O_8$	652.3089	652.3088	-0.2
A7	$C_{34}H_{45}N_7O_8$	680.3402	680.3401	-0.1
A8	$C_{30}H_{43}N_7O_7$	614.3297	614.3295	-0.3
A9	$C_{36}H_{47}N_7O_7$	690.361	690.3608	-0.3
A10	$C_{33}H_{49}N_7O_7$	656.3766	656.3765	-0.2
A11	$C_{38}H_{49}N_7O_7$	716.3766	716.3763	-0.4
A12	$C_{36}H_{53}N_7O_7$	696.4079	696.4075	-0.6
A13	$C_{40}H_{49}N_7O_7$	740.3766	740.3767	0.1
A14	$C_{43}H_{51}N_7O_8$	794.3872	794.3872	0.0
A15	$C_{42}H_{51}N_7O_7$	766.3923	766.3921	-0.3
A16	$C_{37}H_{49}N_7O_7$	704.3766	704.3762	-0.6
A17	$C_{34}H_{45}N_7O_7S$	696.3174	696.3174	0.0
A18	$C_{37}H_{49}N_7O$	704.3766	704.3764	-0.3
A19	$C_{37}H_{49}N_7O_7$	704.3766	704.3763	-0.4
A20	$C_{40}H_{55}N_7O_7$	746.4236	746.4234	-0.3
A21	$C_{34}H_{45}N_7O_8$	680.3402	680.3402	0.0
A22	$C_{35}H_{47}N_7O_8$	694.3559	694.3556	-0.4
A23	$C_{34}H_{45}N_7O_8$	680.3402	680.3404	0.3
A24	$C_{33}H_{43}N_7O_8$	666.3246	666.3244	-0.3
A25	$C_{35}H_{47}N_7O_8$	694.3559	694.3557	-0.3
A26	$C_{41}H_{51}N_7O_8$	770.3872	770.3866	-0.8
A27	$C_{35}H_{47}N_7O_8$	694.3559	694.3554	-0.7
A28	$C_{35}H_{47}N_7O_8$	694.3559	694.3557	-0.3
A29	$C_{35}H_{47}N_7O_8$	694.3559	694.3551	-1.2
A30	$C_{34}H_{47}N_7O_8$	682.3559	682.3559	0.0
B1	$C_{50}H_{57}N_7O_8$	884.4341	884.4338	-0.3
B2	$C_{49}H_{57}N_7O_7$	856.4392	856.4383	-1.1
B3	$C_{44}H_{55}N_7O$	794.4236	794.423	-0.8
B4	$C_{45}H_{55}N_7O_7$	806.4236	806.4234	-0.2
B5	$C_{47}H_{61}N_7O_7$	836.4705	836.4704	-0.1
B6	$C_{43}H_{53}N_7O_7$	780.4079	780.4075	-0.5
B7	$C_{43}H_{53}N_7O_8$	796.4028	796.4021	-0.9
B8	$C_{50}H_{59}N_7O_7$	870.4549	870.4546	-0.3
B9	$C_{50}H_{59}N_7O_7$	870.4549	870.4546	-0.3
B10	$C_{50}H_{59}N_7O_8$	886.4498	886.45	0.2
B11	$C_{51}H_{59}N_7O_8$	898.4498	898.4495	-0.3
B12	$C_{49}H_{57}N_7O_8$	872.4341	872.4341	0
B13	$C_{48}H_{56}N_8O_7$	857.4345	857.4343	-0.2
B14	$C_{51}H_{57}N_7O_8$	896.4341	896.4343	0.2
B15	$C_{51}H_{59}N_7O_8$	898.4498	898.4497	-0.1
B16	$C_{49}H_{56}N_8O_9$	901.4243	901.4237	-0.7
B17	$C_{51}H_{59}N_7O_9$	914.4447	914.4448	0.1
B18	$C_{50}H_{58}N_8O_8$	899.445	899.4453	0.3
B19	$C_{50}H_{59}N_7O_9S$	934.4168	934.4166	-0.2
B20	$C_{50}H_{57}N_7O_9$	900.4291	900.4289	-0.2
B21	$C_{51}H_{59}N_7O_9$	914.4447	914.4443	-0.4
B22	$C_{51}H_{59}N_7O_9$	914.4447	914.4448	0.1
B23	$C_{52}H_{61}N_7O_9$	928.4604	928.4604	0
B24	$C_{52}H_{59}N_7O_9$	926.4447	926.445	0.3
B25	$C_{52}H_{57}N_7O_9$	924.4291	924.4295	0.4
B26	$C_{52}H_{61}N_7O_9$	928.4604	928.4607	0.3
B27	$C_{53}H_{63}N_7O_9$	942.476	942.4762	0.2
B28	$C_{53}H_{63}N_7O_9$	942.476	942.4754	-0.6
B29	$C_{54}H_{65}N_7O_9$	956.4917	956.4918	0.1
B30	$C_{51}H_{60}N_8O_9$	929.4556	929.4558	0.2
B31	$C_{51}H_{62}N_8O_7$	899.4814	899.4813	-0.1
B32	$C_{52}H_{64}N_8O_7$	913.4971	913.4974	0.3
B33	$C_{50}H_{56}N_8O_7$	881.4345	881.4344	-0.1
B34	$C_{51}H_{58}N_8O_7$	895.4501	895.4499	-0.2
B35	$C_{53}H_{61}N_7O_{10}$	956.4553	956.4552	-0.1
B36	$C_{52}H_{57}N_7O_{10}$	940.424	940.4244	0.4
B37	$C_{53}H_{61}N_7O_{10}$	956.4553	956.4553	0
B38	$C_{51}H_{59}N_7O_{10}$	930.4396	930.4401	0.5
B39	$C_{52}H_{58}N_8O_9$	939.44	939.4404	0.4
B40	$C_{51}H_{57}N_7O_{10}$	928.424	928.4243	0.3
B41	$C_{53}H_{63}N_7O_9$	942.476	942.476	0
B42	$C_{53}H_{61}N_7O_9$	940.4604	940.4605	0.1
B43	$C_{51}H_{59}N_7O_{11}S$	978.4066	978.4062	-0.4
B44	$C_{52}H_{61}N_7O_{10}$	944.4553	944.4553	0
B45	$C_{52}H_{61}N_7O_{10}$	944.4553	944.456	0.7
B46	C52H57N7O10	940.424	940.424	0
B47	$C_{52}H_{57}N_7O_{10}$	940.424	940.4243	0.3
B48	$C_{53}H_{59}N_7O_{10}$	954.4396	954.4397	0.1
B49	$C_{53}H_{61}N_7O_{10}$	956.4553	956.455	-0.3
B50	$C_{53}H_{61}N_7O_9$	940.4604	940.4606	0.2
B51	$C_{52}H_{58}N_8O_9$	939.44	939.4401	0.1
B52	$C_{53}H_{61}N_7O_{11}$	972.4502	972.4488	-1.4
B53	$C_{54}H_{63}N_7O_{11}$	986.4658	986.4653	-0.5
A26-Fl	$C_{68}H_{73}N_7O_{16}$	1244.5187	1244.5199	1
B52-Fl	$C_{80}H_{83}N_7O_{19}$	1446.5816	1446.5822	0.4
B53-Fl	$C_{81}H_{85}N_7O_{19}$	1460.5973	1460.5986	0.9
B52-A	$C_{49}H_{52}N_6O_{10}$	885.3818	885.3819	0.1
B53-A	$C_{50}H_{54}N_6O_{10}$	899.3974	899.3976	0.2
*B52-A	$C_{49}H_{52}N_6O_{10}$	885.3818	885.3822	0.5
*B53-A	$C_{50}H_{54}N_6O_{10}$	899.3974	899.3978	0.4
C1A	$C_{47}H_{51}BN_6O_9$	855.3883	855.3872	-1.7
C2A	$C_{47}H_{51}BN_6O_9$	855.3883	855.3878	-1.5
C3A	$C_{46}H_{49}BN_6O_9$	841.3727	841.3725	-0.2
C4A	$C_{46}H_{49}BN_6O_9$	841.3727	841.3726	-0.1
C5A	$C_{46}H_{48}N_6O_7$	797.3657	797.3643	-1.8
C6A	$C_{45}H_{49}BN_6O_8$	813.37789	813.3779	0
B52-Cy5	$C_{83}H_{94}N_9O_{11}^+$	1392.7067	1392.7074	0.5
B53-Cy5	$C_{84}H_{96}N_9O_{11}^+$	1406.7224	1406.72	-1.7
*B52-Cy5	$C_{83}H_{94}N_9O_{11}^+$	1392.7067	1392.7048	-1.4
*B53-Cy5	$C_{84}H_{96}N_9O_{11}^+$	1406.7224	1406.72	-1.7
B52-Et-Cy5	$C_{87}H_{102}N_9O_{11}^+$	1448.7693	1448.7652	-2.8
B53-Et-Cy5	$C_{88}H_{104}N_9O_{11}^+$	1462.785	1462.7824	-1.8
*B52-Et-Cy5	$C_{87}H_{102}N_9O_{11}^+$	1448.7693	1448.7672	-1.4
*B53-Et-Cy5	$C_{88}H_{104}N_9O_{11}^+$	1462.785	1462.7844	-0.4
Cy5-enAc	$C_{36}H_{47}N_4O_2^+$	567.3694	567.3687	-1.2

Supplementary Table 9 | Crystal diffraction statistics for all CypD-inhibitor co-crystal structures reported in this work. Statistics for the highest-resolution shell are shown in parentheses.

	CypD + JOMBt	CypD + A26	CypD + B1	CypD + B2	CypD + B3	CypD + B21	CypD + B23	CypD + B25	CypD + B52	CypD + B53
Wavelength	0.97933 Å	0.97933 Å	0.97933 Å	0.97933 Å	0.97933 Å	0.92016 Å	0.97931 Å	0.92011 Å	0.92011 Å	0.92011 Å
Resolution range	46.482 - 1.746 (1.776 - 1.746)	37.923 - 1.055 (1.073 - 1.055)	44.534 - 1.211 (1.232 - 1.211)	37.700 - 1.461 (1.486 - 1.461)	44.684 - 1.522 (1.549 - 1.522)	30.96 - 0.97 (1.000 - 0.97)	25.72 - 1.18 (1.21 - 1.18)*	25.83 - 1.572 (1.61 - 1.572)	25.73 - 1.16 (1.19 - 1.16)	25.68 - 1.104 (1.143 - 1.104)
Space group	P 21 21 21	P 1 21 1	P 21 21 21	P 1 21 1	P 21 21 21	P 43	P 2 21 21	P 2 21 21	P 2 21 21	P 2 21 21
Unit cell	38.505 57.879 78.01 90 90 90	36.061 67.087 38.662 90 101.218 90	40.195 60.141 66.264 90 90 90	35.838 67.41 38.455 90 101.371 90	40.279 60.265 66.592 90 90 90	38.632 38.632 103.509 90 90 90	38.542 67.177 69.071 90 90 90	38.564 67.19 69.58 90 90 90	38.408 67.032 69.324 90 90 90	38.492 66.885 68.968 90 90 90
Total reflections	219423 (11765)	495647 (7380)	470396 (2064)	138577 (7413)	302677 (16985)	1028544 (9410)	528630 (783)	196553 (168)	804168 (37928)	934857 (53061)
Unique reflections	17266 (869)	78970 (2430)	43493 (799)	31004 (1557)	25507 (1301)	82203 (2612)	46222 (393)	18407 (161)	62319 (4063)	71217 (4690)
Multiplicity	12.7 (13.5)	6.3 (3.0)	10.8 (2.6)	4.5 (4.8)	11.9 (13.1)	12.5 (3.6)	11.4 (2.0)	10.7 (1.0)	12.9 (9.3)	13.1 (11.3)
Completeness (%)	94.2 (99.9)	95.5 (59.6)	87.8 (33.4)	99.70 (99.8)	99.9 (100.0)	92.15 (61.6)	76.6 (9.1)	71.1 (8.7)	99.0 (88.5)	98.6 (89.1)
Mean I/sigma(I)	12.5 (2.3)	15.03 (2.3)	23.0 (2.1)	9.9 (3.8)	9.1 (3.2)	13.1 (0.9)	19.00 (1.80)	33.70 (5.30)	13.50 (2.90)	9.60 (3.00)
Wilson B-factor	19.17	9.05	11.42	9.55	13.97	7.71	9.38	9.58	9.65	8.9
R-merge	0.136 (1.195)	0.057 (0.461)	0.051 (0.428)	0.116 (1.046)	0.165 (1.607)	0.107 (1.364)	0.078 (0.508)	0.056 (0.035)	0.112 (0.595)	0.180 (0.596)
R-meas	0.142 (1.241)	0.062 (0.558)	0.053 (0.538)	0.131 (1.167)	0.173 (1.671)	0.112 (1.615)	0.02682 (0.1685)	0.01992 (0.06542)	0.03362 (0.2297)	0.0529 (0.2299)
R-pim	0.040 (0.333)	0.024 (0.307)	0.015 (0.317)	0.060 (0.507)	0.050 (0.456)	0.029 (0.828)	0.01896 (0.1192)	0.01409 (0.04626)	0.02377 (0.1625)	0.0374 (0.1625)
CC1/2	0.996 (0.772)	0.999 (0.798)	1 (0.766)	0.994 (0.574)	0.996 (0.911)	0.997 (0.241)	0.998 (0.803)	0.998 (0.963)	0.995 (0.921)	0.994 (0.888)
CC*	0.999 (0.954)	1 (0.961)	1 (0.951)	0.998 (0.91)	0.999 (0.98)	0.999 (0.635)	1 (0.987)	1 (0.997)	1 (0.987)	0.999 (0.98)
Reflections used in refinement	17146 (1755)	78345 (5507)	43488 (1922)	30862 (3055)	25407 (2507)	82203 (4868)	42114 (2767)	18375 (288)	62243 (5800)	71160 (6445)
Reflections used for R-free	848 (80)	3807 (270)	2108 (103)	1531 (157)	1275 (117)	1997 (108)	1831 (120)	1826 (27)	2011 (188)	1998 (186)
R-work	0.1702 (0.1894)	0.1364 (0.2102)	0.1513 (0.2112)	0.1654 (0.1734)	0.1690 (0.2280)	0.1365 (0.3333)	0.1350 (0.1812)	0.1339 (0.1678)	0.1357 (0.1795)	0.1369 (0.1698)
R-free	0.1842 (0.2096)	0.1447 (0.2210)	0.1604 (0.2169)	0.1786 (0.1975)	0.1886 (0.2689)	0.1519 (0.3275)	0.1473 (0.1933)	0.1635 (0.2256)	0.1516 (0.1875)	0.1477 (0.1917)
CC(work)	0.946 (0.932)	0.974 (0.915)	0.969 (0.896)	0.962 (0.935)	0.962 (0.923)	0.978 (0.650)	0.972 (0.927)	0.966 (0.946)	0.969 (0.955)	0.970 (0.940)
CC(free)	0.946 (0.892)	0.977 (0.931)	0.956 (0.909)	0.952 (0.922)	0.952 (0.929)	0.983 (0.689)	0.967 (0.915)	0.949 (0.821)	0.952 (0.960)	0.955 (0.940)
Number of non-hydrogen atoms	1387	1570	1462	1454	1524	1539	1557	1555	1479	1556
macromolecules	1248	1258	1270	1279	1275	1277	1269	1244	1244	1272
ligands	49	56	65	63	58	67	68	68	71	72
solvent	90	256	127	112	191	195	220	243	164	212
Protein residues	163	163	163	163	163	163	163	163	163	163
RMS(bonds)	0.007	0.007	0.008	0.012	0.008	0.012	0.009	0.007	0.011	0.006
RMS(angles)	0.99	1.04	1.08	1.51	1.299	1.28	1.2	1.062	1.327	0.88
Ramachandran favored (%)	96.3	95.68	95.65	95.68	96.32	96.89	95.65	95.65	95.03	95.03
Ramachandran allowed (%)	3.7	4.32	4.35	4.32	3.68	3.11	4.35	4.35	4.97	4.97
Ramachandran outliers (%)	0	0	0	0	0	0	0	0	0	0
Rotamer outliers (%)	0	0	0	0.72	1.44	0.71	0	0	0	0
Clashscore	1.18	0	0.77	0	2.31	2.68	1.14	1.57	2.35	1.91
Average B-factor	22.3	12.17	15.3	12.06	18.44	10.72	12.77	13.25	12.43	12.37
macromolecules	21.31	9.54	14.69	11.39	16.8	9.09	10.43	10.64	10.56	10.21
ligands	25.97	12.59	14.13	12.8	19.38	9.61	11.32	12.01	11.04	11.26
solvent	33.89	24.99	21.98	19.23	29.13	21.78	26.7	26.97	27.18	25.7
Number of TLS groups	8	10	7	8	9	9	9	7	9	8

*Data was collected to 1.18 Å resolution but cut off for refinement to 1.3 Å resolution to improve completeness.

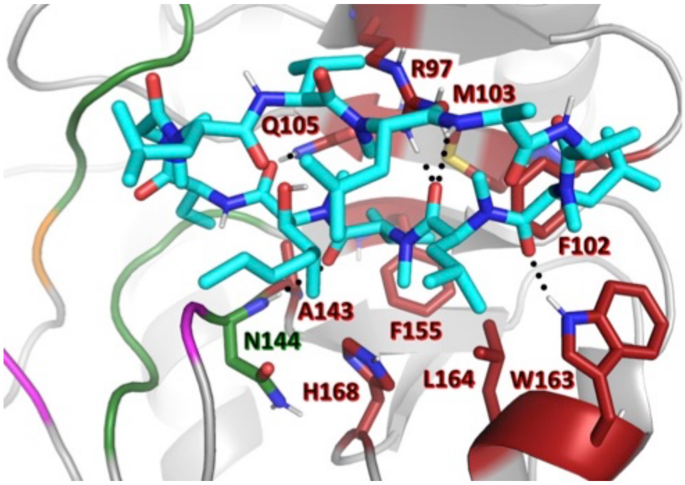

Supplementary Figure 1 | Cyclosporine A interactions with active site residues of CypD (PDB ID 2Z6W). CsA makes primary contacts with active site residues (red) and minimally interacts with S2 pocket residues (green, magenta, orange), with the exception of N144 which lies at the interface between the two pockets. Primary hydrophobic interactions are between Val on CsA and F155 at the base of the active site pocket, surrounded by M103, L164 and H168. Predicted hydrogen bond contacts with CypD residues (black dashes) are shown with R97, Q105, N144, and W163. These residues are highly conserved between isoforms, contributing to the promiscuous cyclophilin inhibition by CsA (Extended Data 1c).

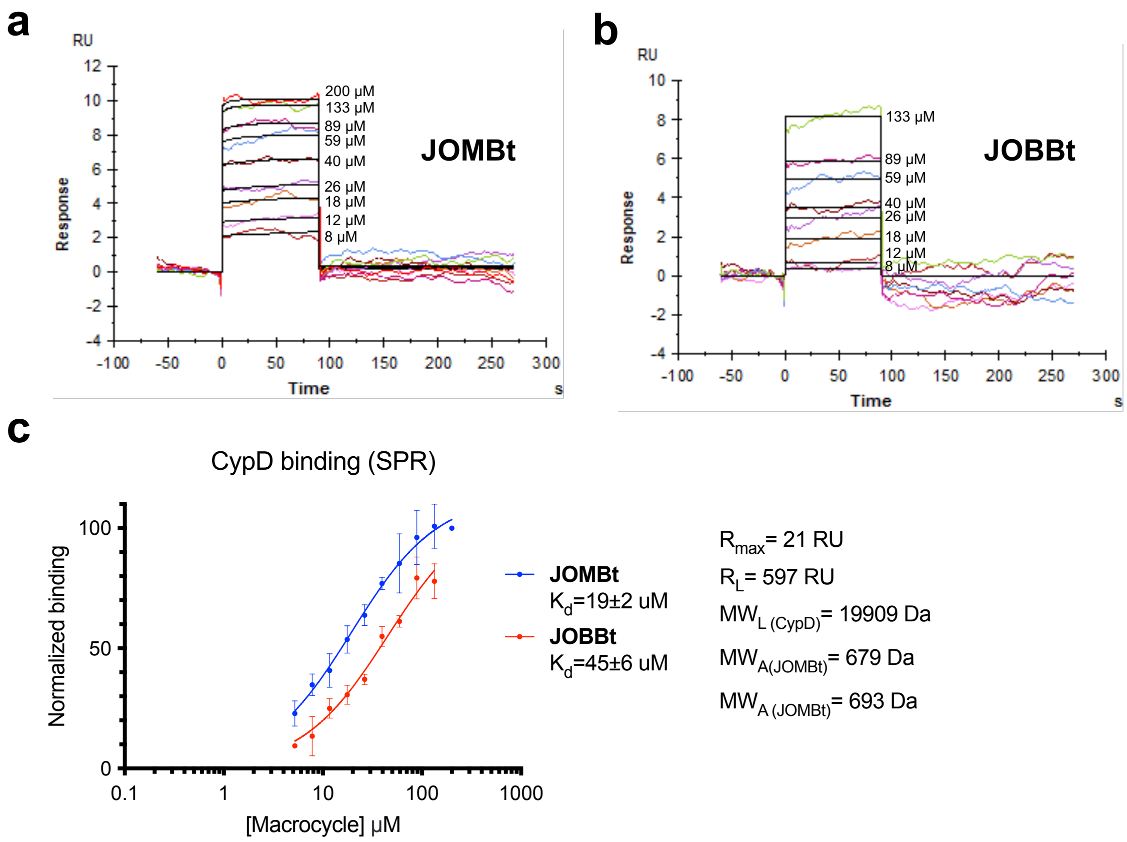

Supplementary Figure 2 | Surface plasmon resonance measurement of JOMBt and JOBBt binding to immobilized CypD. Representative sensorgram of **a**, JOMBt and **b**, JOBBt, flowed over amine immobilized CypD. Black lines are calculated kinetic fits for each dose. **c**, Binding dose response of JOMBt and JOBBt, where sensorgram RU values were normalized to DMSO treated baseline and 200 μM JOMBt. Individual replicates represent fitted kinetic RU value during compound administration at each dose. Shown are SPR parameters including theoretical R_{max}, based off amount of immobilized CypD (R_L) and molecular weights of assayed material ($MW_{L (CypD)}$, $MW_{A(JOMBt)}$, $MW_{A(JOMBt)}$). K_d values reflect mean±SEM of three technical replicates. Data points and error bars reflect mean±SD of individual assays at one dose.

a

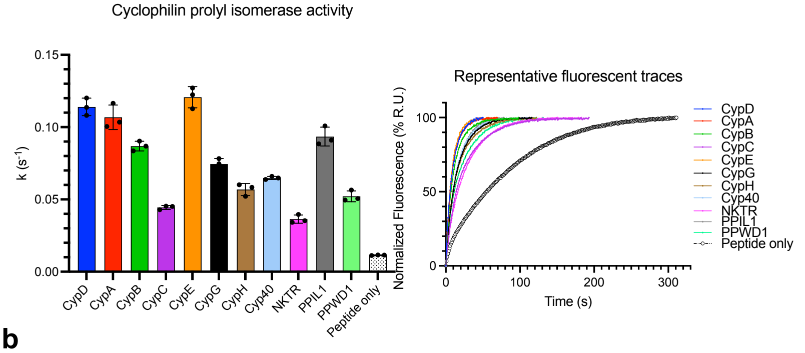

Cyclophilin	Prolyl-isomerization rate constant (k, s^{-1})	Signal/ Background	Z' Factor
CypD	0.114±0.006	10.4	0.82
CypA	0.107±0.009	9.7	0.71
CypB	0.087±0.003	7.9	0.87
CypC	0.044±0.001	4	0.89
CypE	0.121±0.007	11	0.80
CypG	0.074±0.004	6.7	0.80
CypH	0.057±0.004	5.2	0.73
Cyp40	0.065±0.001	5.9	0.93
NKTR	0.036±0.003	3.3	0.62
PPIL1	0.093±0.007	8.5	0.74
PPWD1	0.052±0.004	4.7	0.69
Peptide only	0.0110±0.0002	-	-

b

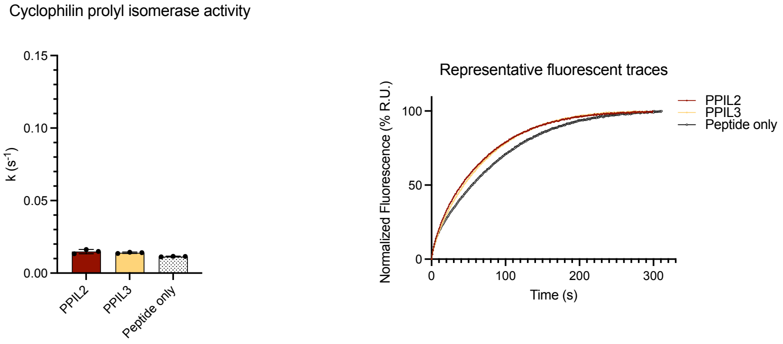

Cyclophilin	Prolyl-isomerization rate constant (k, s^{-1})	Signal/ Background	Z' Factor
PPIL2	0.015±0.002	1.4	-0.65
PPIL3	0.0140±0.0005	1.3	0.30
Peptide only	0.0110±0.0002	-	-

c

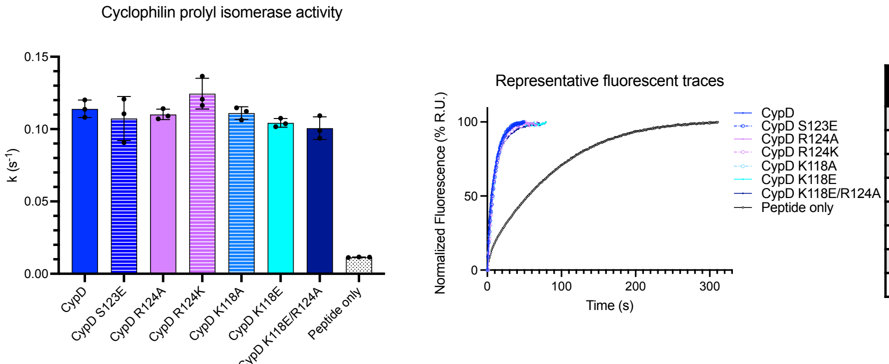

CypD mutant	Prolyl-isomerization rate constant (k, s^{-1})	Signal/ Background	Z' Factor
WT	0.114±0.006	10.4	0.82
K118A	0.111±0.004	10.1	0.87
K118E	0.104±0.003	9.5	0.90
S123E	0.11±0.01	10	0.69
R124A	0.110±0.004	10	0.87
R124K	0.12±0.01	10.9	0.72
K118E/R124K	0.101±0.008	9.2	0.73
Peptide only	0.0110±0.0002	-	-

d

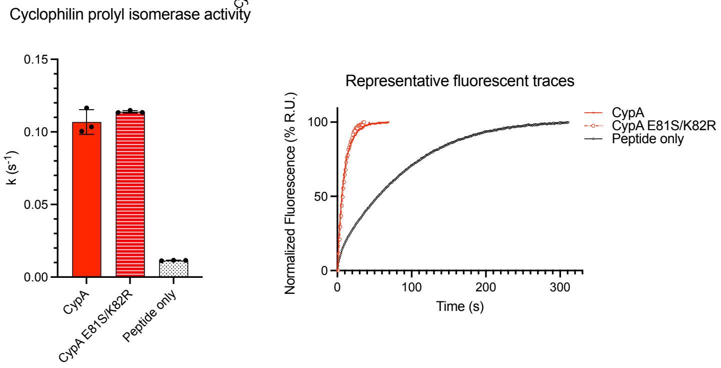

CypA mutant	Prolyl-isomerization rate constant (k^{-1})	Signal/ Background	Z' Factor
WT	0.107±0.009	9.7	0.71
E81S/K82R	0.114±0.008	10.4	0.76
Peptide only	0.0110±0.0002	-	-

e

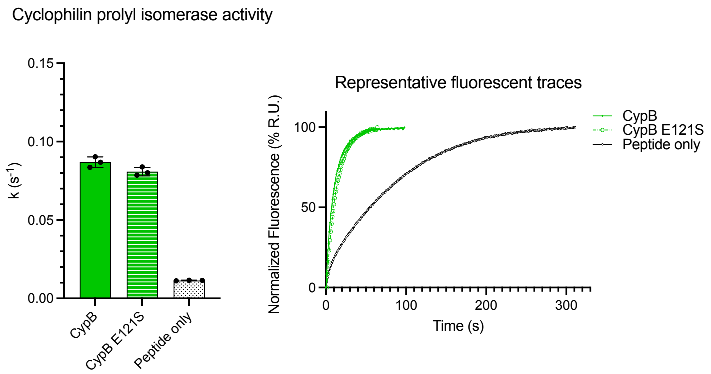

CypB mutant	Prolyl-isomerization rate constant (k, s^{-1})	Signal/ Background	Z' Factor
WT	0.087±0.003	7.9	0.87
E121S	0.081±0.003	7.4	0.86
Peptide only	0.0110±0.0002	-	-

f

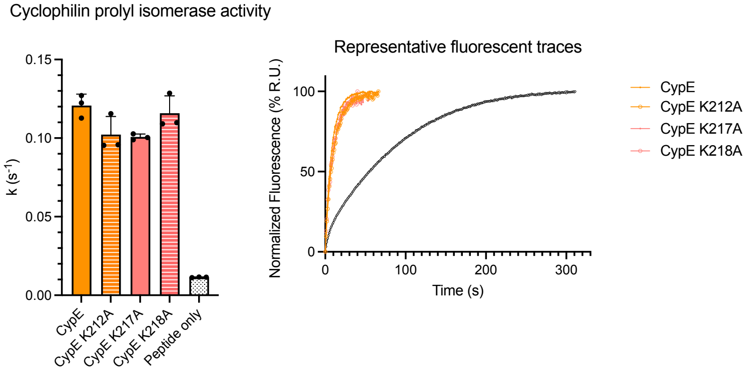

CypE mutant	Prolyl-isomerization rate constant (k, s^{-1})	Signal/ Background	Z' Factor
WT	0.121±0.007	11	0.80
CypE K212A	0.10±0.01	9.1	0.66
CypE K217A	0.101±0.002	9.2	0.93
CypE K218A	0.12±0.01	10.9	0.72
Peptide only	0.0110±0.0002	-	-

Supplementary Figure 3 | Prolyl isomerase activity of cyclophilins on Suc-AAPF-AMC. Rate constant, signal to background ratio (rate constant of cyclophilin divided by rate constant of peptide), and Z' values for: **a**, all wild-type cyclophilins screened; **b**, prolyl isomerase-inactive cyclophilins; **c**, CypD mutants; **d**, CypA E81S/K82R double mutant; **e**, CypB E121S mutant; **f**, CypE mutants. Also included are one representative kinetic trace of each cyclophilin during the assay timescale, normalized to fluorescence at t=0 and maximum fluorescent value. Analyses were halted after calculated maximum fluorescent value due to observed fluorescent bleaching during the sustained fluorescent plateau. Values and error bars reflect mean±SD of three technical replicates.

a

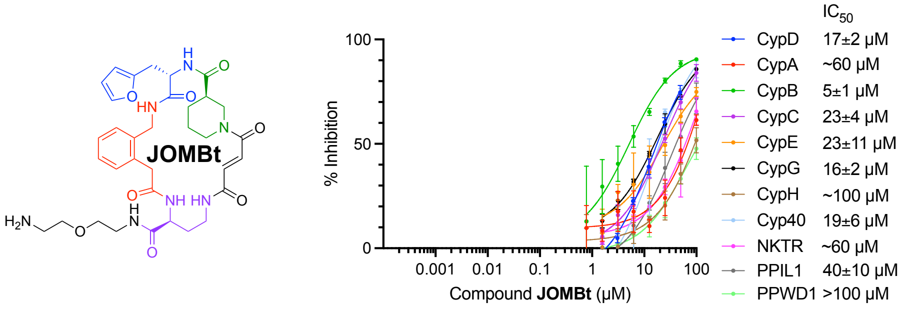

b

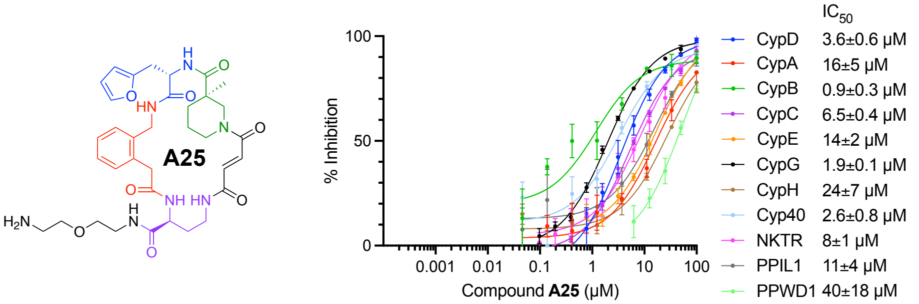

c

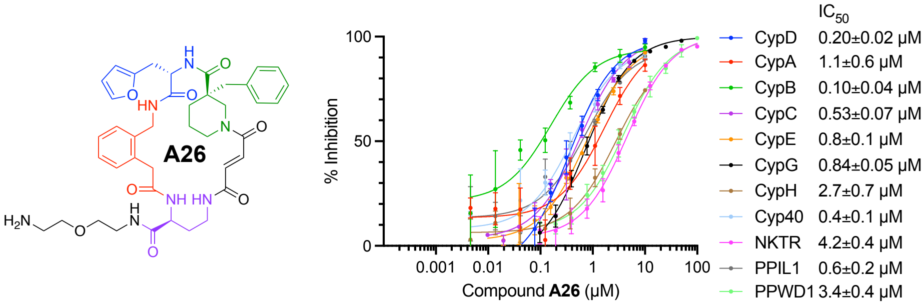

d

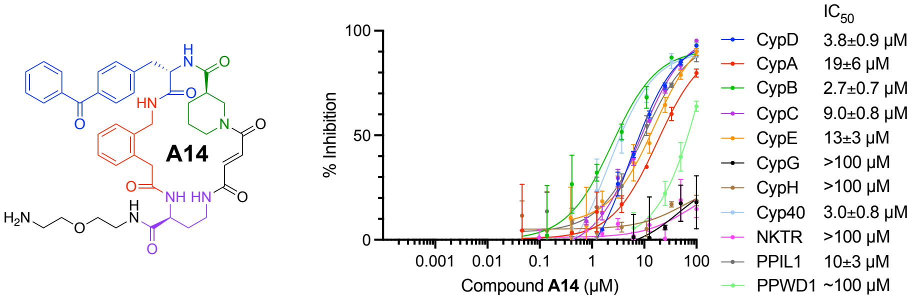

Supplementary Figure 4 | Functionalization of piperidine α-carbon improves CypD potency, while replacing the second building block improves CypD selectivity. a, Structure and cyclophilin inhibition dose response profile shown for **a,** JOMBt; **b, A25**; **c, A26**; and **d, A14**. While iterative functionalization of the α-carbon with a methyl or benzyl group improves potency for CypD, these compounds are still promiscuous cyclophilin inhibitors, with no gain in selectivity compared to JOMBt. In contrast, diversifying the second building block with larger moieties such as a benzophenone only marginally improve potency, but greatly increase selectivity over CypG, CypH, NKTR, and PPWD1. Crystal structures of JOMBt and **A26** corroborate these observations, as the second building block is positioned within the S2 pocket, while the α-carbon on the piperidine is near a conserved Thr residue (Fig. 1f, Extended Data 3b). IC_{50} values reflect mean±SEM of three technical replicates. Data points and error bars reflect mean±SD of individual assays at one dose.

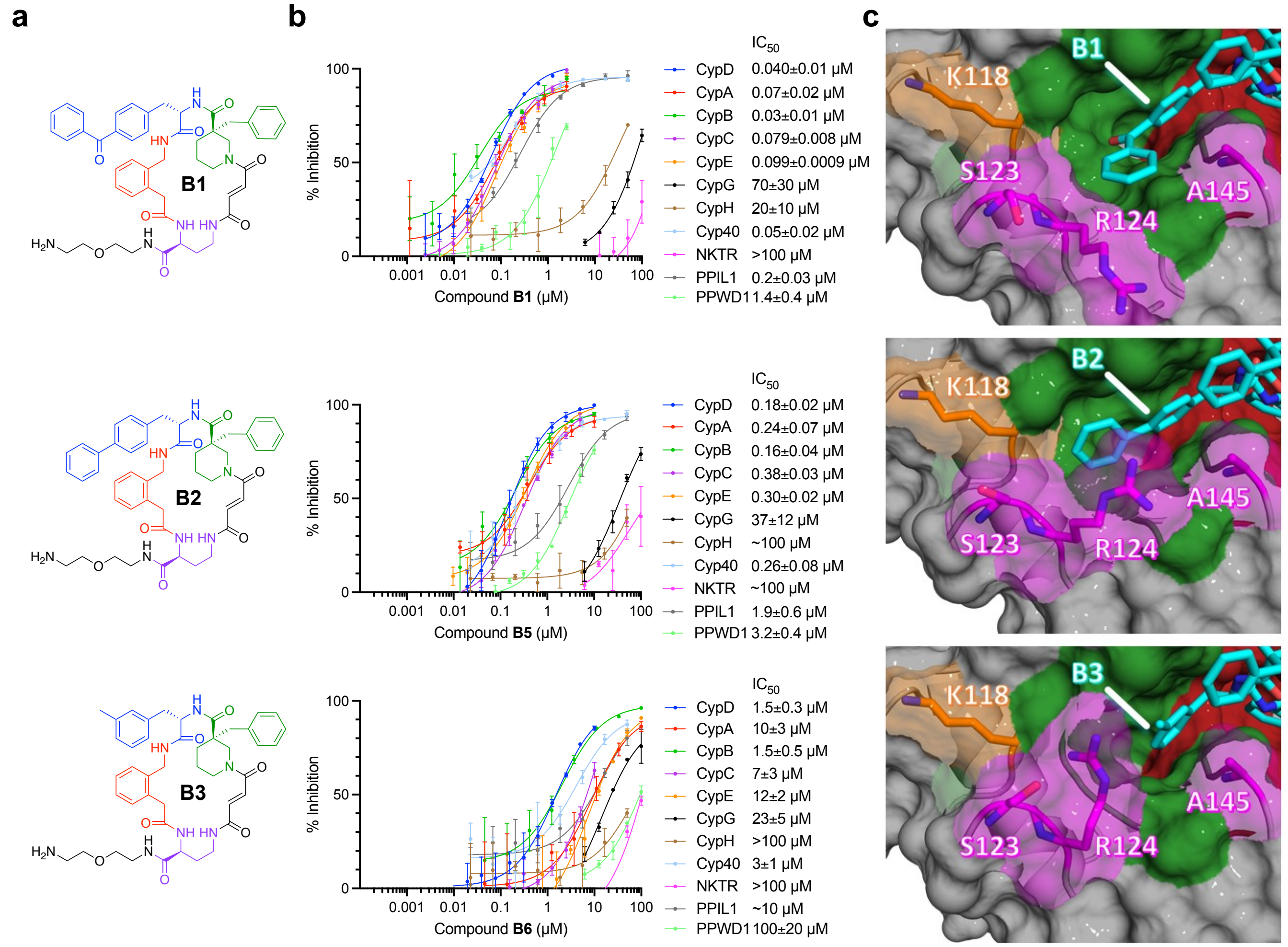

Supplementary Figure 5 | Large S2 binding groups improve selectivity for CypD. a, Structure of **B1, B2,** and **B3**. **b,** Prolyl-isomerase cyclophilin inhibition profile for **B1, B2,** and **B3**. **c**, Co-crystal structures of **B1** (PDB ID 7TGU, 1.21 Å resolution), **B2** (PDB ID 7TGV, 1.46 Å resolution), and **B3** (PDB ID 7TH1 1.52 Å resolution) bound to CypD, viewing the S2 pocket. Active site binding is identical to that of **A26** (Fig. 1e). The large benzophenone and biphenyl of **B1** and **B2**, respectively, fill the S2 pocket more completely than **B3**, which contains a *m*-methylphenyl group. This interaction imparts selectivity over CypG, CypH, NKTR, and PPWD1, which have sterically occluded or more structurally rigid S2 pocket residues. Macrocycles with larger S2-binding groups cause R124 to flip out of the S2 pocket in CypD (see Supplementary Fig. 32, Supplementary Video 1). IC_{50} values reflect mean±SEM of three technical replicates. Data points and error bars reflect mean±SD of individual assays at one dose.

a

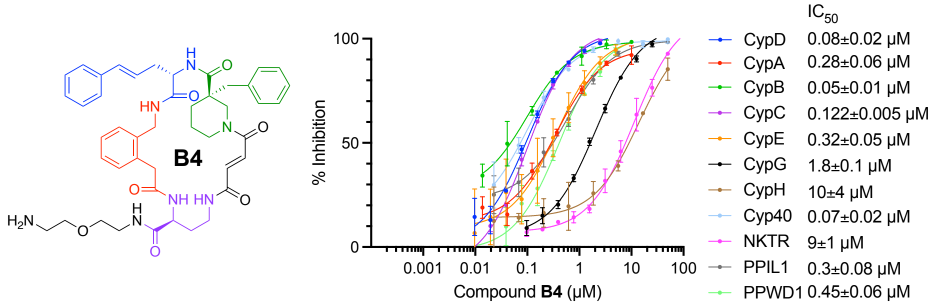

b

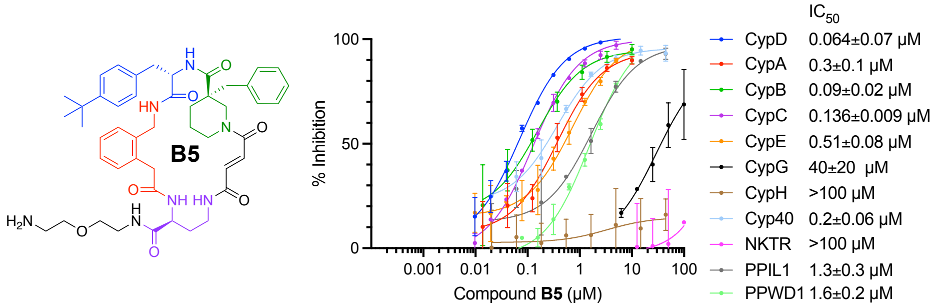

c

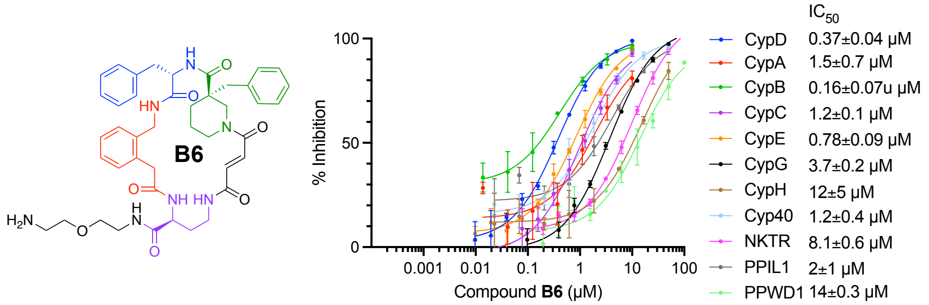

d

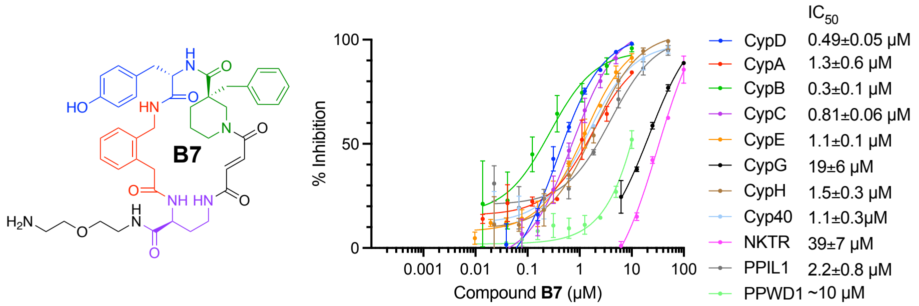

Supplementary Figure 6 | Large hydrophobic S2 binding groups impart selectivity for CypD over CypG, CypH, NKTR, and PPWD1. Structure and cyclophilin inhibition dose response data for **a, B4**; **b, B5**; **c, B6**; and **d, B7**. Small S2 binding moieties such as the phenylalanine in **B6** do not provide sufficient steric clash with S2 residues, even for cyclophilins with sterically occluded S2 pockets. Binding deeper into S2 pocket with *tert*-butyl phenylalanine in **B5** results in selectivity over CypG, CypH, and NKTR. An intermediate size styryl-alanine in **B4** offers moderate selectivity. **B4** and **B5** also exhibit slightly improved potency for CypD compared to **B6.** Using a tyrosine in **B7** offers little selectivity for CypH, and selectivity over NKTR and CypG, but a decrease in potency compared to **B4** and **B5**. IC_{50} values reflect mean±SEM of three technical replicates. Data points and error bars reflect mean±SD of individual assays at one dose.

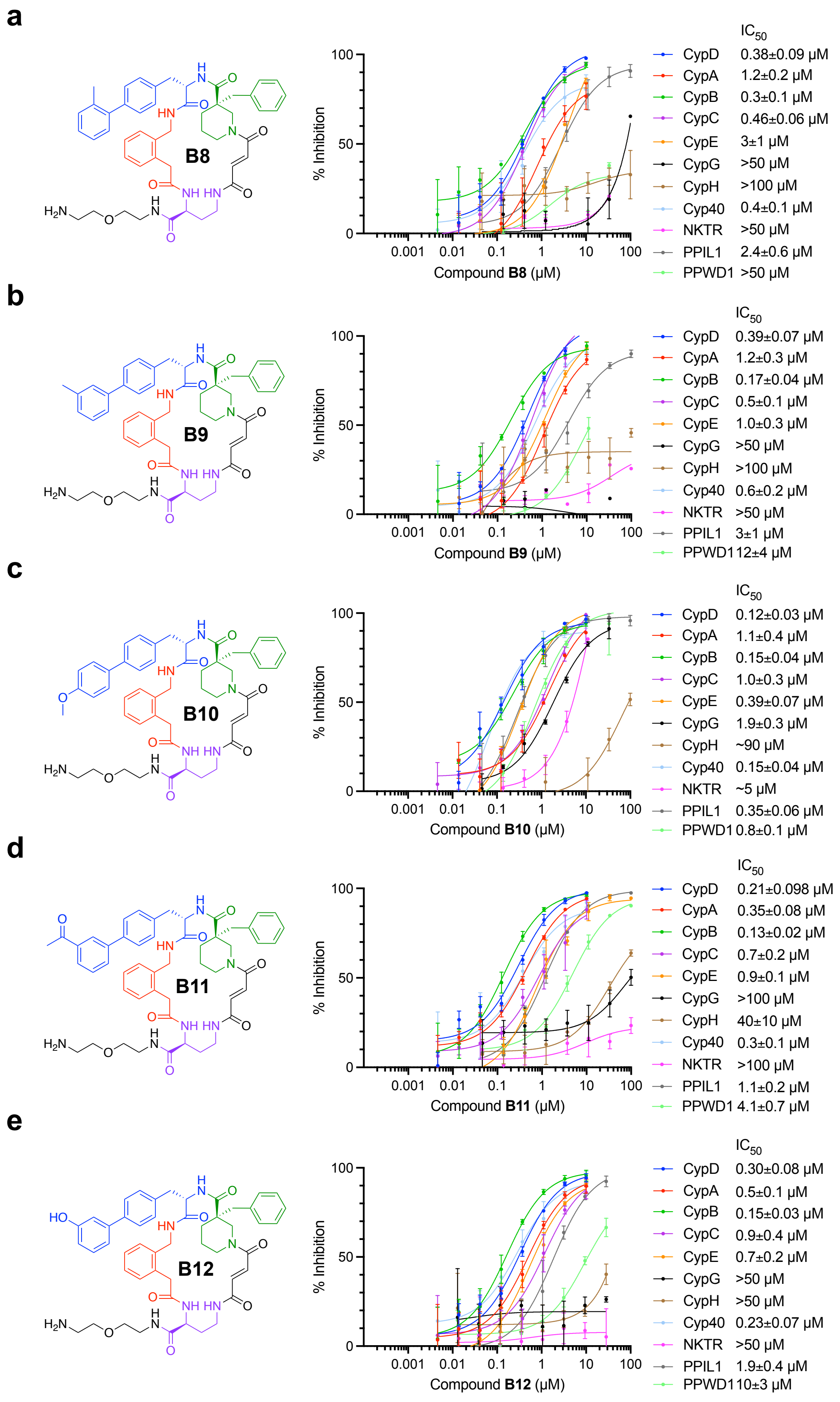
a
B8
IC50
CypD 0.38±0.09 μM
CypA 1.2±0.2 μM
CypB 0.3±0.1 μM
CypC 0.46±0.06 μM
CypE 3±1 μM
CypG >50 μM
CypH >100 μM
Cyp40 0.4±0.1 μM
NKTR >50 μM
PPIL1 2.4±0.6 μM
PPWD1 >50 μM
% Inhibition
Compound B8 (μM)
b
B9
IC50
CypD 0.39±0.07 μM
CypA 1.2±0.3 μM
CypB 0.17±0.04 μM
CypC 0.5±0.1 μM
CypE 1.0±0.3 μM
CypG >50 μM
CypH >100 μM
Cyp40 0.6±0.2 μM
NKTR >50 μM
PPIL1 3±1 μM
PPWD1 12±4 μM
% Inhibition
Compound B9 (μM)
c
B10
IC50
CypD 0.12±0.03 μM
CypA 1.1±0.4 μM
CypB 0.15±0.04 μM
CypC 1.0±0.3 μM
CypE 0.39±0.07 μM
CypG 1.9±0.3 μM
CypH ~90 μM
Cyp40 0.15±0.04 μM
NKTR ~5 μM
PPIL1 0.35±0.06 μM
PPWD1 0.8±0.1 μM
% Inhibition
Compound B10 (μM)
d
B11
IC50
CypD 0.21±0.098 μM
CypA 0.35±0.08 μM
CypB 0.13±0.02 μM
CypC 0.7±0.2 μM
CypE 0.9±0.1 μM
CypG >100 μM
CypH 40±10 μM
Cyp40 0.3±0.1 μM
NKTR >100 μM
PPIL1 1.1±0.2 μM
PPWD1 4.1±0.7 μM
% Inhibition
Compound B11 (μM)
e
B12
IC50
CypD 0.30±0.08 μM
CypA 0.5±0.1 μM
CypB 0.15±0.03 μM
CypC 0.9±0.4 μM
CypE 0.7±0.2 μM
CypG >50 μM
CypH >50 μM
Cyp40 0.23±0.07 μM
NKTR >50 μM
PPIL1 1.9±0.4 μM
PPWD1 10±3 μM
% Inhibition
Compound B12 (μM)

f

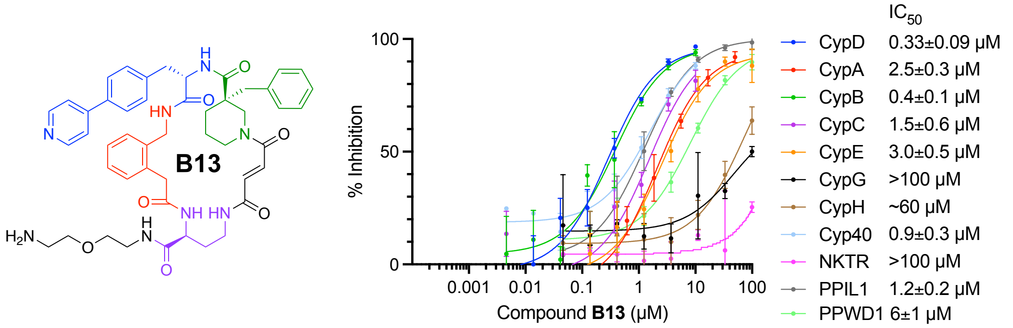

g

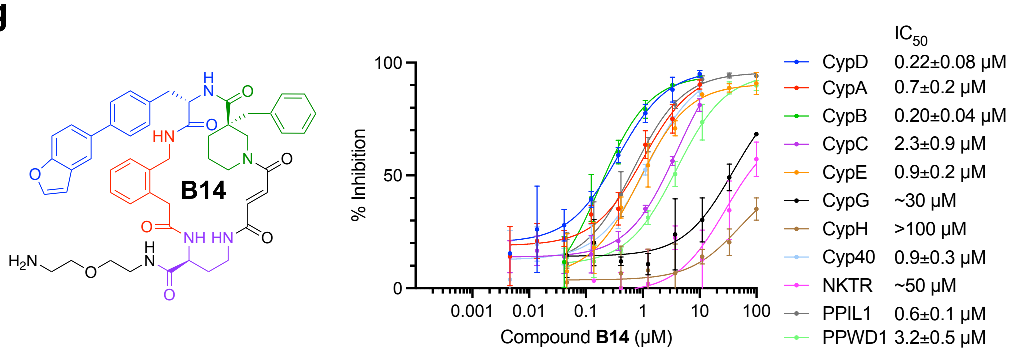

Supplementary Figure 7 | Biphenyl and bi-aromatic derivatives of B2 showing no improvement in selectivity for CypD. Structure and cyclophilin inhibition dose response data for **a, B8**; **b, B9**; **c, B10**; **d, B11**; **e, B12**; **f, B13**; and **g, B14**. These compounds exhibit similar gain in selectivity over CypG, CypH, NKTR, and PPWD1 as in **B2**, but no further selectivity for CypD. IC_{50} values reflect mean±SEM of three technical replicates. Data points and error bars reflect mean±SD of individual assays at one dose.

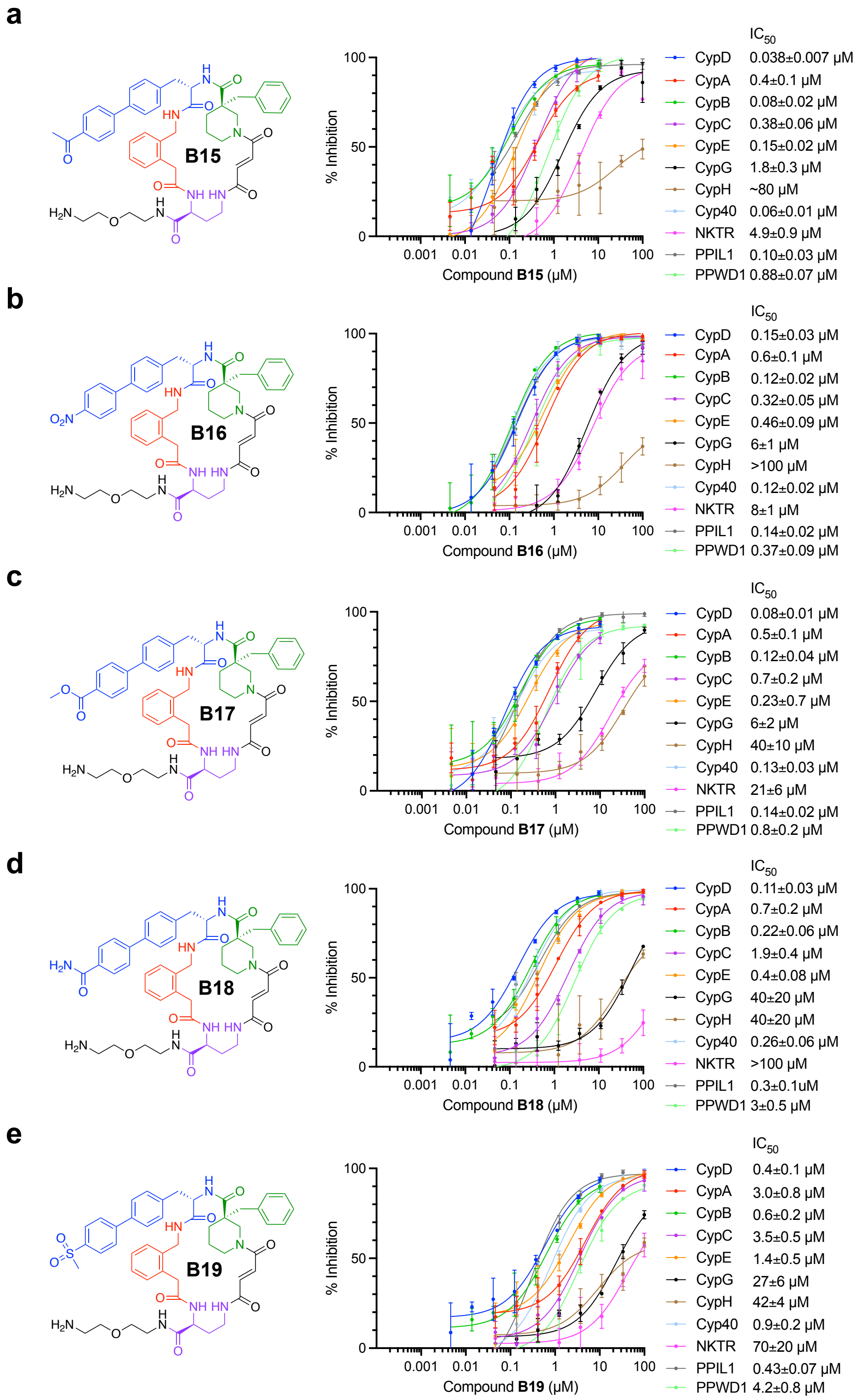

Supplementary Figure 8 | Para-carbonyl-containing biphenyl moieties show partial selectivity over CypC. Structure and cyclophilin inhibition dose response data for **a, B15**; **b, B16**; **c, B17**; **d, B18**; and **e, B19**. **B15**, **B17**, **B18**, and **B19** show reduced potency for CypC compared to CypD. These para-carbonyl biphenyl groups maintain a similar selectivity profile over CypG, CypH, and NKTR as **B2**. IC_{50} values reflect mean±SEM of three technical replicates. Data points and error bars reflect mean±SD of individual assays at one dose.

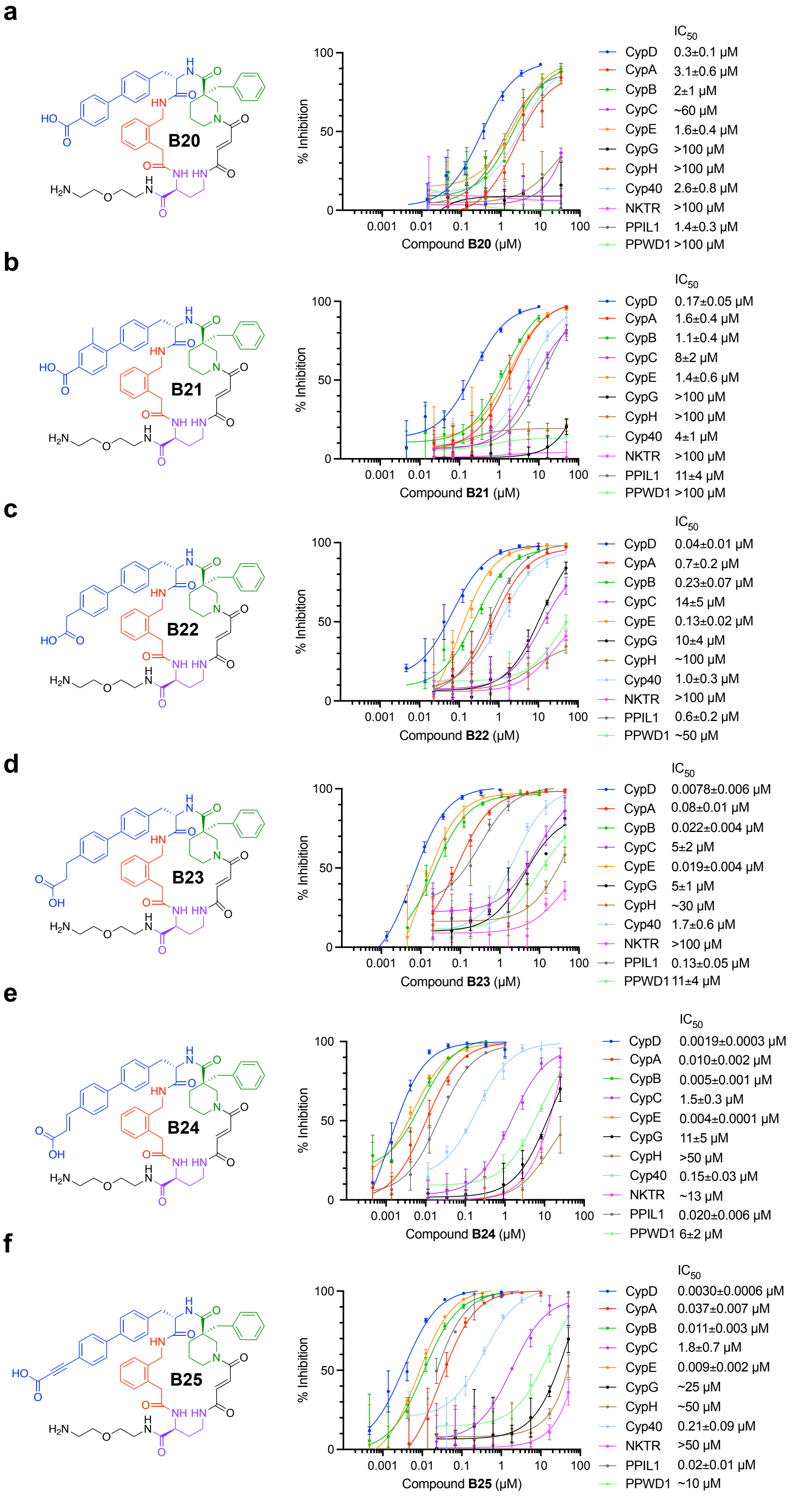

Supplementary Figure 9 | Para-carboxy-containing biphenyl groups provide enhanced selectivity and potency for CypD. Structure and cyclophilin inhibition dose response data for

a, B20; **b, B21**; **c, B22**; **d, B23**; **e, B24**; and **f, B25**. The carboxylate in **B20** and **B21** provides a high degree selectivity over CypC and Cyp40. Extension of the carboxylate from the biphenyl with a methylene in **B22** further improves potency for CypD and selectivity over CypC, Cyp40, PPIL1 and CypA. Extension with an ethylene in **B23** gives high potency for CypD, strong selectivity over CypC and Cyp40, and appreciable selectivity over CypA and PPIL1. Replacement of **B23**'s ethylene linker between the biphenyl and carboxylate with a vinyl or ethynyl linker in **B24** and **B25**, respectively, mimics **B23** potency and selectivity profile, but with slightly lower selectivity over PPIL1. IC_{50} values reflect mean±SEM of three technical replicates. Data points and error bars reflect mean±SD of individual assays at one dose.

.

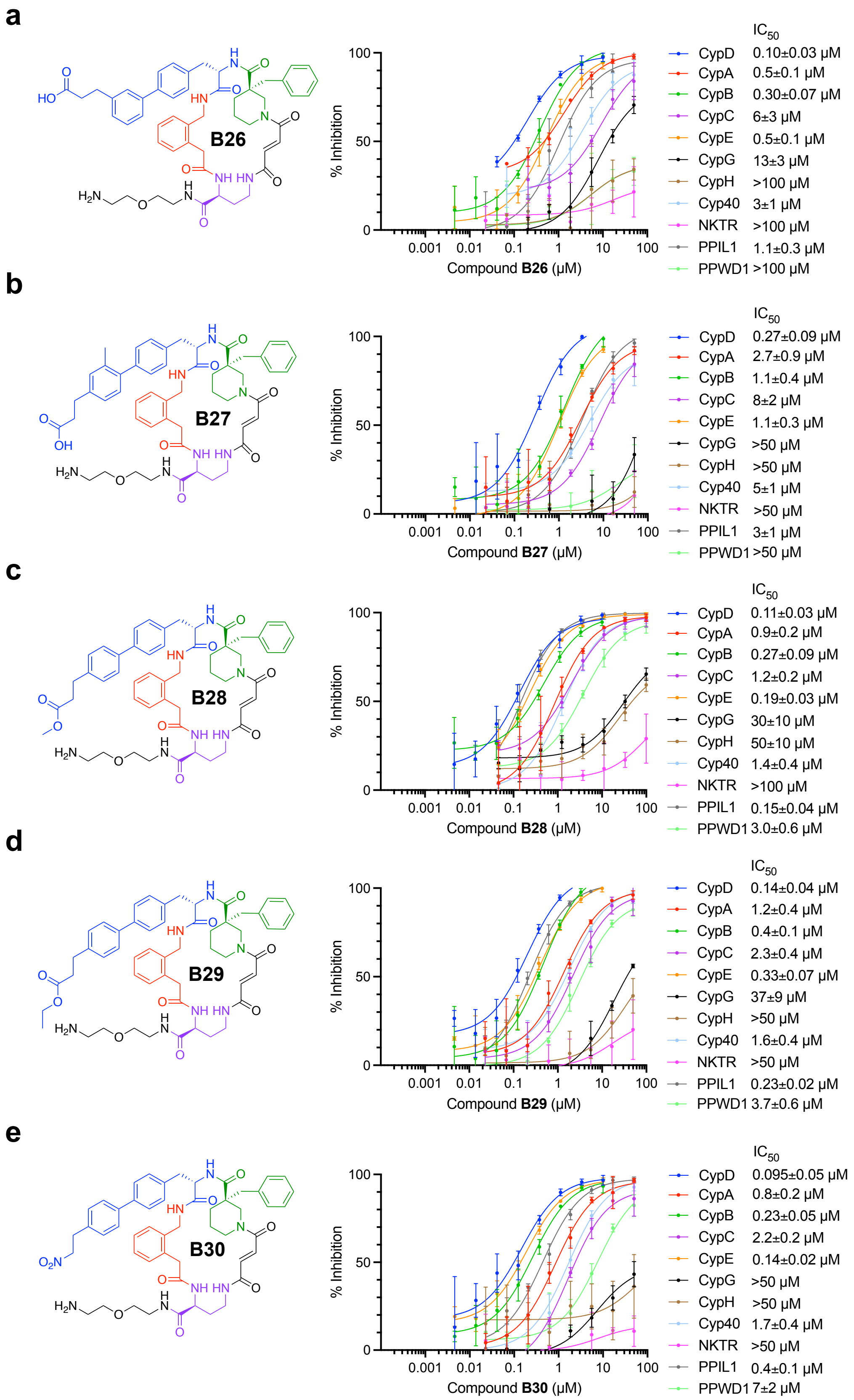

Supplementary Figure 10 | Masking or moving the carboxylate of B23 results in loss in potency and selectivity for CypD. Structure and cyclophilin inhibition dose response data for

a, B26; **b, B27**; **c, B28**; **d, B29**; and **e, B30.** Compared to **B23**, alternate placement of the carboxylate on the biphenyl results in decreased potency and selectivity, shown with *meta* placement of the carboxylate in **B26** or adjusting biphenyl substitution in **B27**. Presumably, **B27** exhibits a binding mode similar to that shown in the **B21** co-crystal structure (PDB ID 7TH6, 0.97 Å resolution), where the *ortho*-methyl migrates the biphenyl out of the S2 pocket. Masking the negative charge of the carboxylate with a methyl ester, ethyl ester, or a net-neutral nitro group in **B28**, **B29**, and **B30** respectively, results in the same loss in potency and selectivity. IC_{50} values reflect mean±SEM of three technical replicates. Data points and error bars reflect mean±SD of individual assays at one dose.

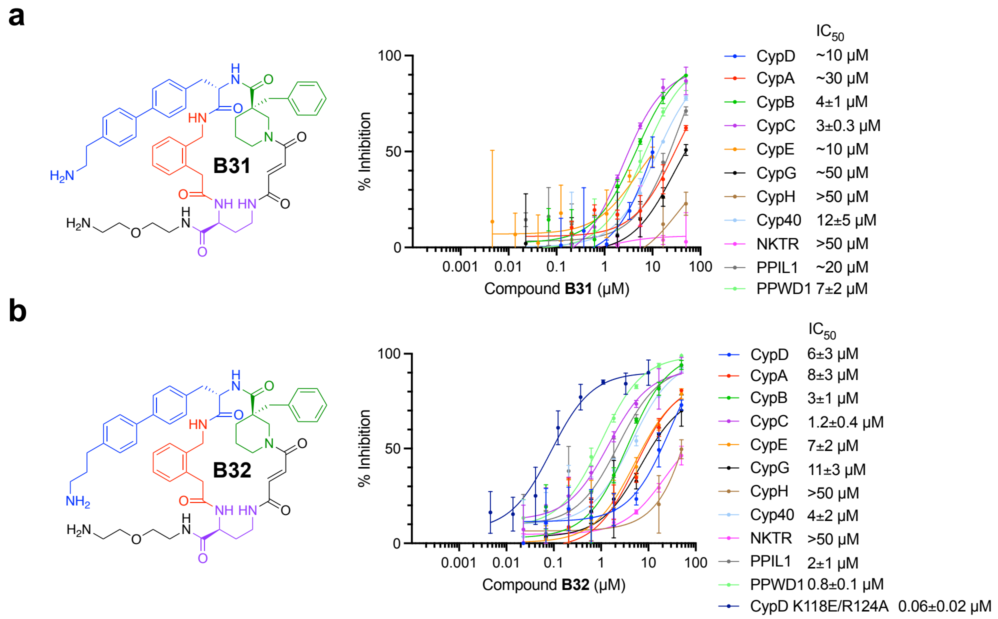

Supplementary Figure 11 | Inverting ligand charge abrogates CypD potency and shifts the cyclophilin selectivity profile. Structure and cyclophilin inhibition dose response data for **a, B31**; and **b, B32**. Inserting an amine with a net positive charge results in >1,000-fold reduction in CypD potency, presumably due to charge-charge repulsion at the K118 residue. **B31** and **B32** also show some selectivity preference for CypC and PPWD1, respectively. Both of these cyclophilins have a negatively charged glutamate residue replacing CypD's K118 residue. The inhibition potency of **B32** is restored against the charge-inverted mutant CypD K118E/R124A, preferentially inhibiting this mutant over any wild-type cyclophilin. IC_{50} values reflect mean±SEM of three technical replicates. Data points and error bars reflect mean±SD of individual assays at one dose.

a

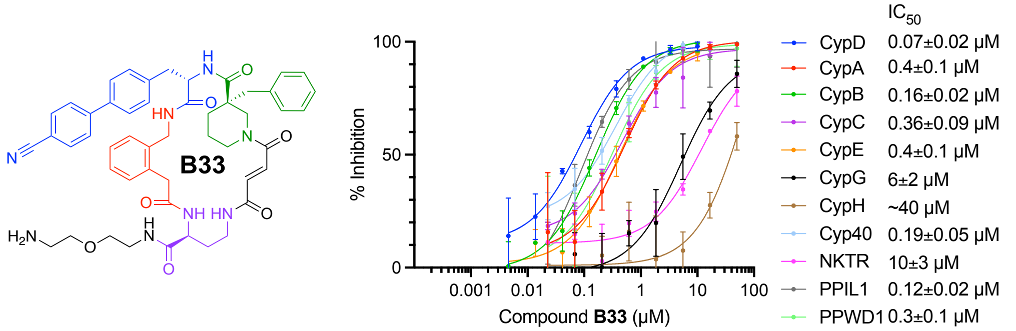

b

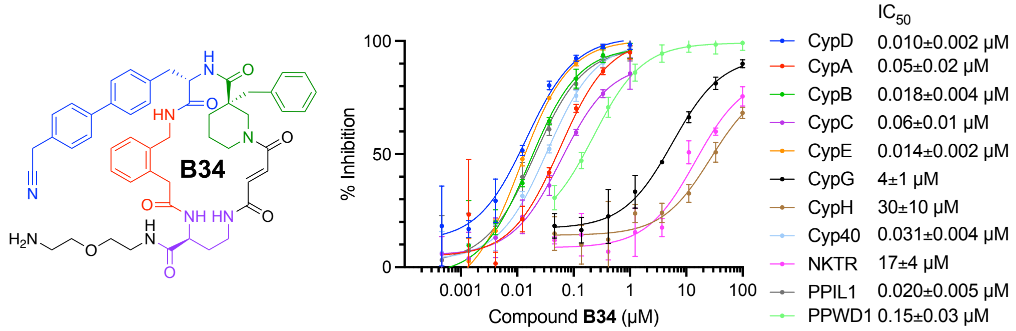

Supplementary Figure 12 | Nitrile-containing biphenyl groups show similar CypD inhibition potencies and linker dependencies as carboxylates. Structure and cyclophilin inhibition dose response data for **a, B33**; and **b, B34**. The increased potency of **B34** compared to **B33** is consistent with the same trend we observed for **B22** and **B23**. IC_{50} values reflect mean±SEM of three technical replicates. Data points and error bars reflect mean±SD of individual assays at one dose.

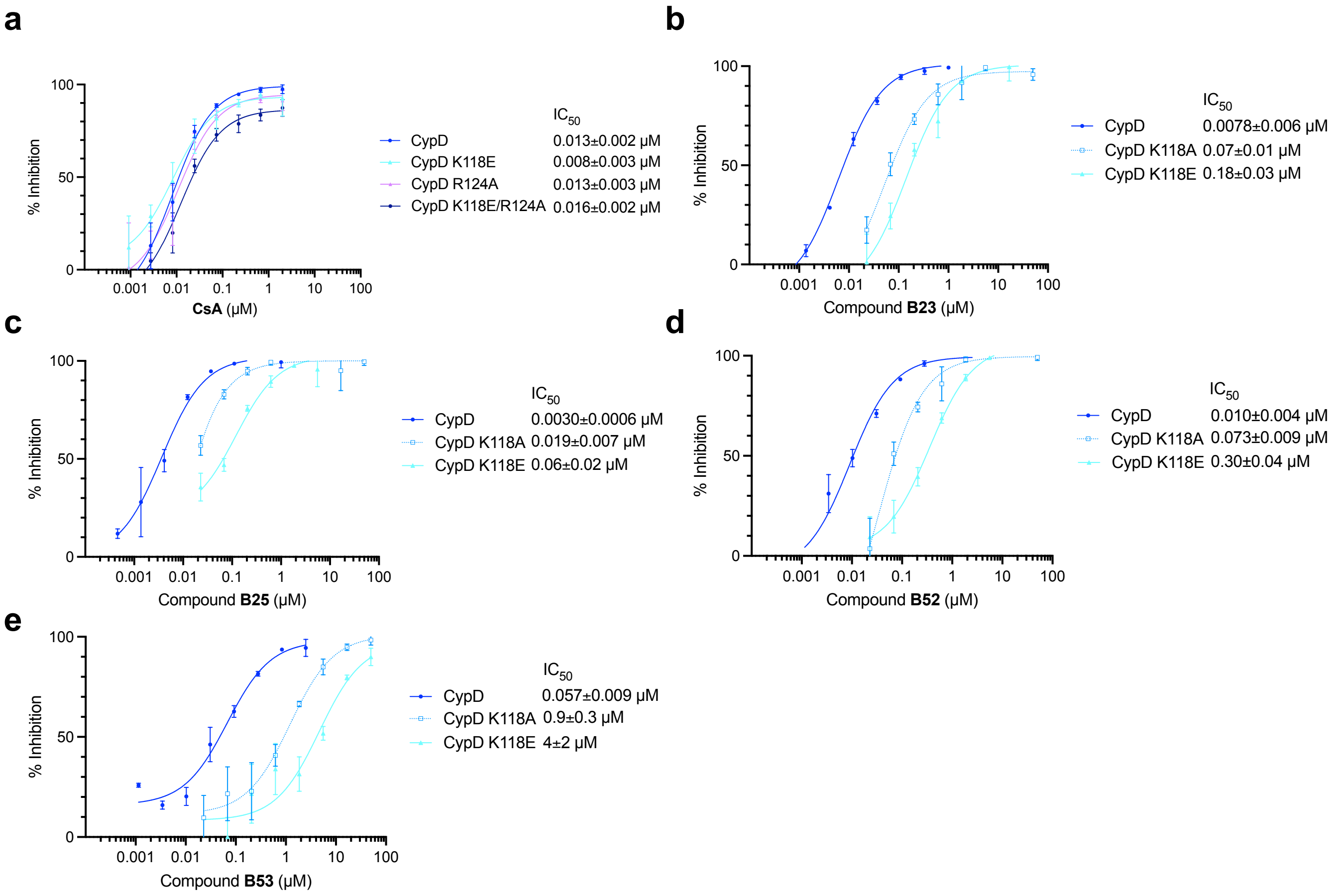

Supplementary Figure 13 | Residue K118 is necessary for potent CypD inhibition with carboxylate-containing inhibitors. Dose response curves of CypD, CypD K118A, and CypD K118E with **a, CsA**; **b, B23**; **c, B25**; **d, B52**; and **e, B53**. **CsA** as an active site ligand does not show appreciable changes in potency for CypD mutants compared to wild-type CypD. A salt bridge was observed in each crystal structure between a carboxylate on **B23**, **B25**, **B52**, and **B53**, and residue K118 (Fig. 3c, Fig. 4c). Mutation to a neutral alanine results in a 6- to 20-fold decrease in potency compared to wild-type CypD. Mutation to a negatively charged glutamate results in a 20- to 100-fold decrease in potency compared to wild-type CypD. For CypD wild-type IC_{50} data with **B52** and **B53**, values reflect mean±SD of four independent replicates (each comprising three technical replicates). Graphs show a representative single independent replicate (Independent replicate 1 is shown, containing three technical replicates) with data points and error bars reflecting mean±SD of individual assays at one dose. Further independent replicates are shown in Supplementary Fig. 18b-c. All other IC_{50} values reflect mean±SEM of three technical replicates, with data points and error bars reflecting mean±SD of individual assays at one dose.

a

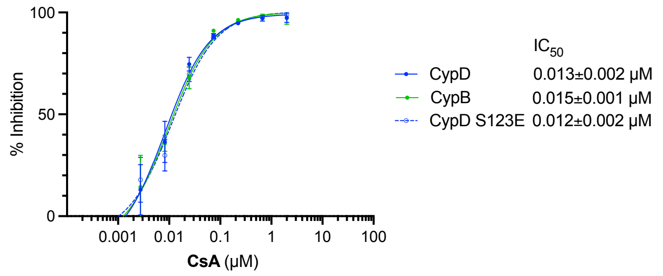

b

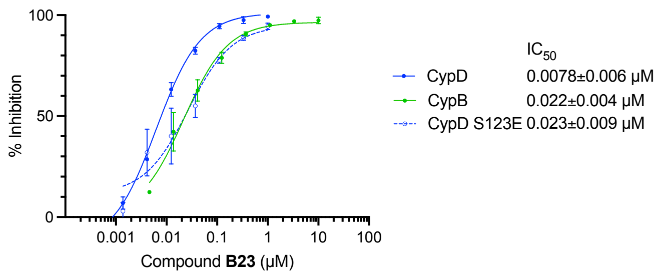

c

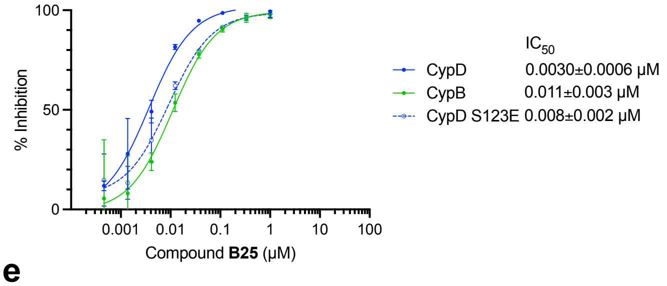

d

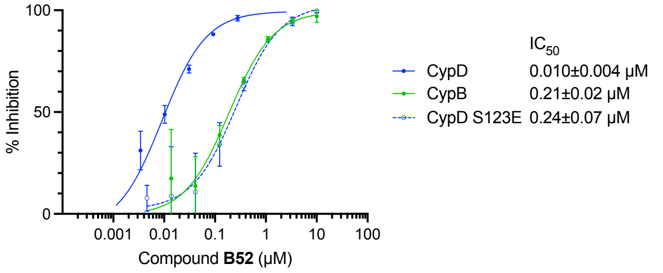

e

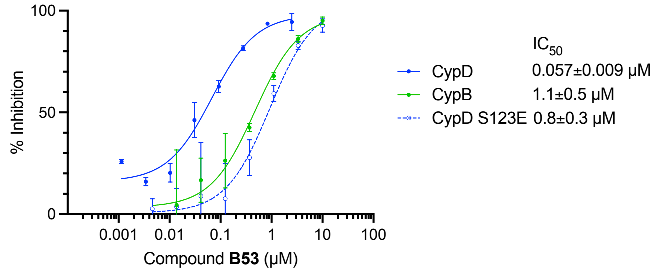

f

Cyclophilin	118	119	123	124
CypD	Lys	Ser	Ser	Arg
CypB	Lys	Ser	Glu	Arg
CypD S123E	Lys	Ser	Glu	Arg

Supplementary Figure 14 | S123 gatekeeper position dictates inhibitor potency. Dose response curves of CypD, CypB, and CypD S123E, shown for **a, CsA**; **b, B23**; **c, B25**. **d, B52**; and **e, B53. f,** List of residues on the far side of the S2 pocket of cyclophilins that are proximal to the ligand carboxylates, with the CypD mutation underlined**. CsA** as an active site ligand does not show appreciable differences in potency between wild-type CypD, wild-type CypB, and CypD S123E. Wild-type CypB and wild-type CypD share identical S2 pockets with the exception of the analogous CypD S123 residue, which is a glutamate in CypB. CypD S123E thus provides the same S2 pocket as CypB WT, resulting in almost identical potencies for these two proteins for all inhibitors shown. For CypD and CypB wild-type IC_{50} data with **B52** and **B53**, values reflect mean±SD of four independent replicates (each comprising three technical replicates). Graphs show a representative single independent replicate (Independent replicate 1 is shown, containing three technical replicates) with data points and error bars reflecting mean±SD of individual assays at one dose. Further independent replicates are shown in Supplementary Fig. 18b-c. All other IC_{50} values reflect mean±SEM of three technical replicates, with data points and error bars reflecting mean±SD of individual assays at one dose.

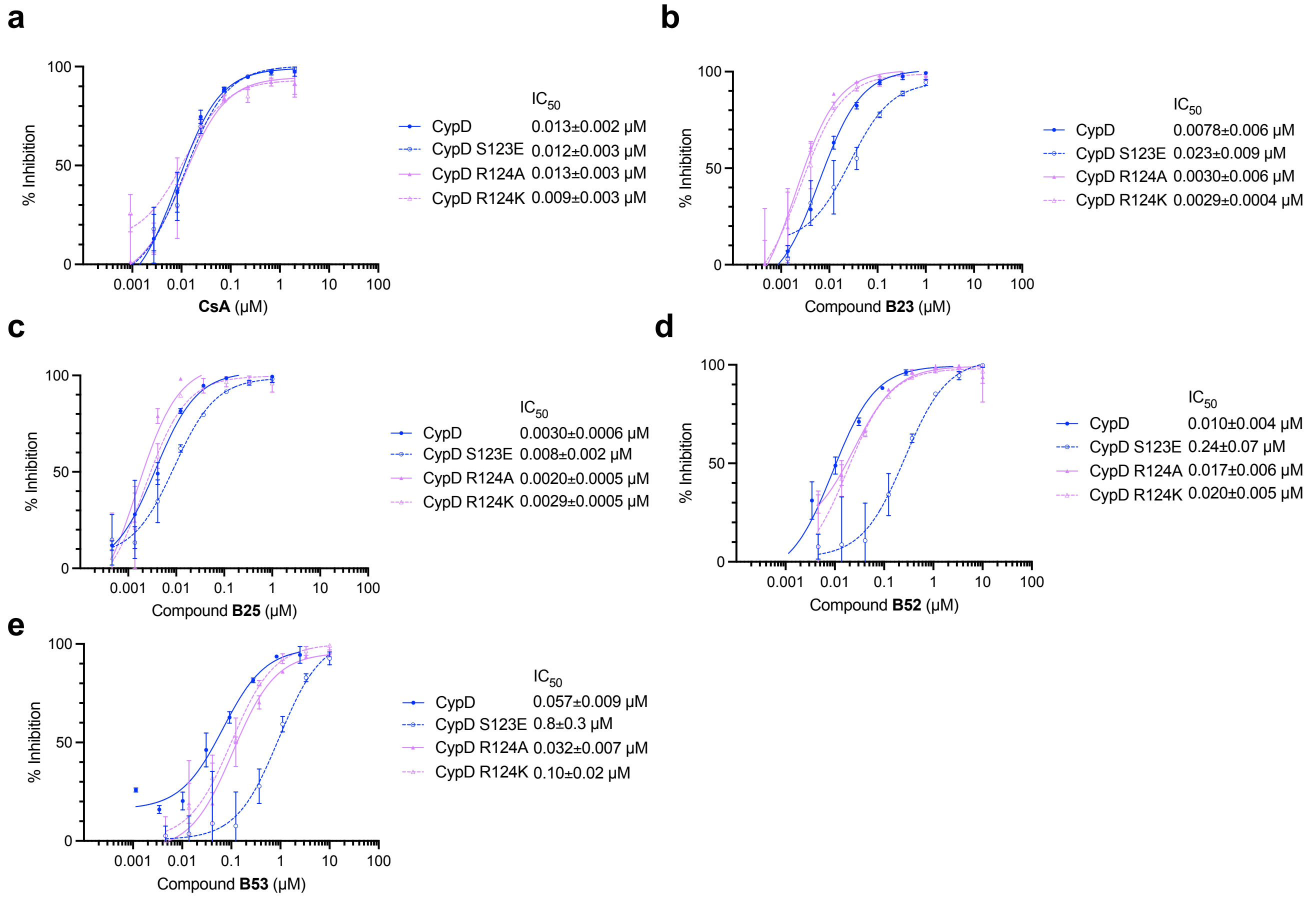

Supplementary Figure 15 | Prolyl isomerase inhibition activity on CypD gatekeeper mutants. Dose response curves of CypD and CypD S123E, CypD R124A, and CypD R124K mutants, shown for **a, CsA**; **b, B23**; **c, B25**; **d, B52**; and **e, B53**. **CsA** as an active site ligand does not show appreciable differences in potency between wild-type CypD and CypD mutants. Each carboxylate-containing ligand shows a modest drop in potency for CypD S123E compared to wild-type CypD, indicating a transient interaction with this residue. Little to no drop-in potency for R124A or R124K mutants suggest that the carboxylates for each compound do not interact with this residue directly. For CypD wild-type IC_{50} data with **B52** and **B53**, values reflect mean±SD of four independent replicates (each comprising three technical replicates). Graphs show a representative single independent replicate (Independent replicate 1 is shown, containing three technical replicates) with data points and error bars reflecting mean±SD of individual assays at one dose. Further independent replicates are shown in Supplementary Fig. 18b-c. All other IC_{50} values reflect mean±SEM of three technical replicates, with data points and error bars reflecting mean±SD of individual assays at one dose.

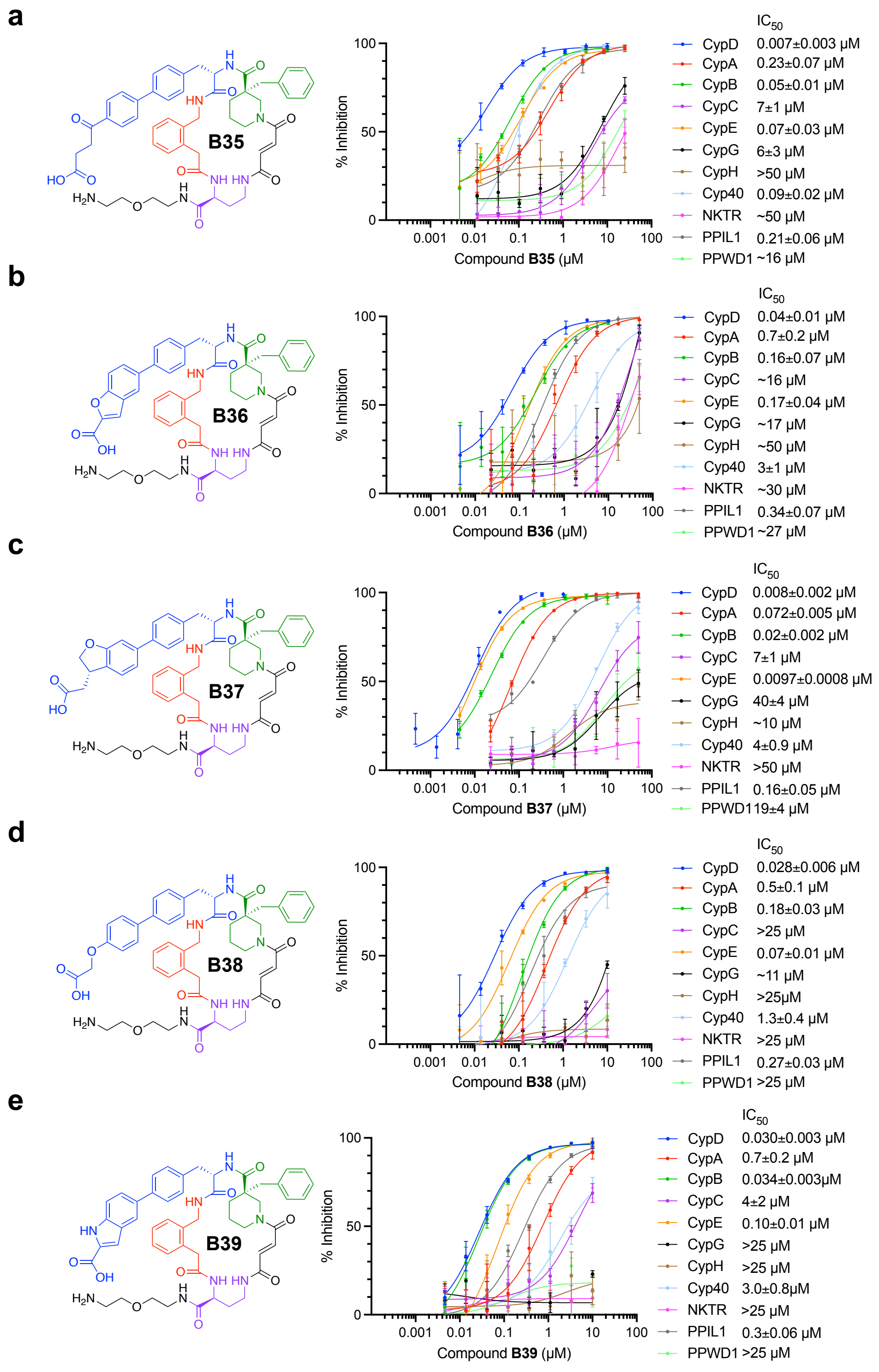

Supplementary Figure 16 | Derivatives of B23 that show similar selectivity and potency profiles for CypD. Structure and cyclophilin inhibition dose response data for **a, B35**; **b, B36**; **c, B37**; **d, B38**; and **e, B39**. Compared to **B23**, all compounds maintain the same CypD potency and selectivity profile. Presumably, these compounds retain the interactions with K118 and S119 shown in the **B23** co-crystal structure (Fig. 3c). IC_{50} values reflect mean±SEM of three technical replicates. Data points and error bars reflect mean±SD of individual assays at one dose.

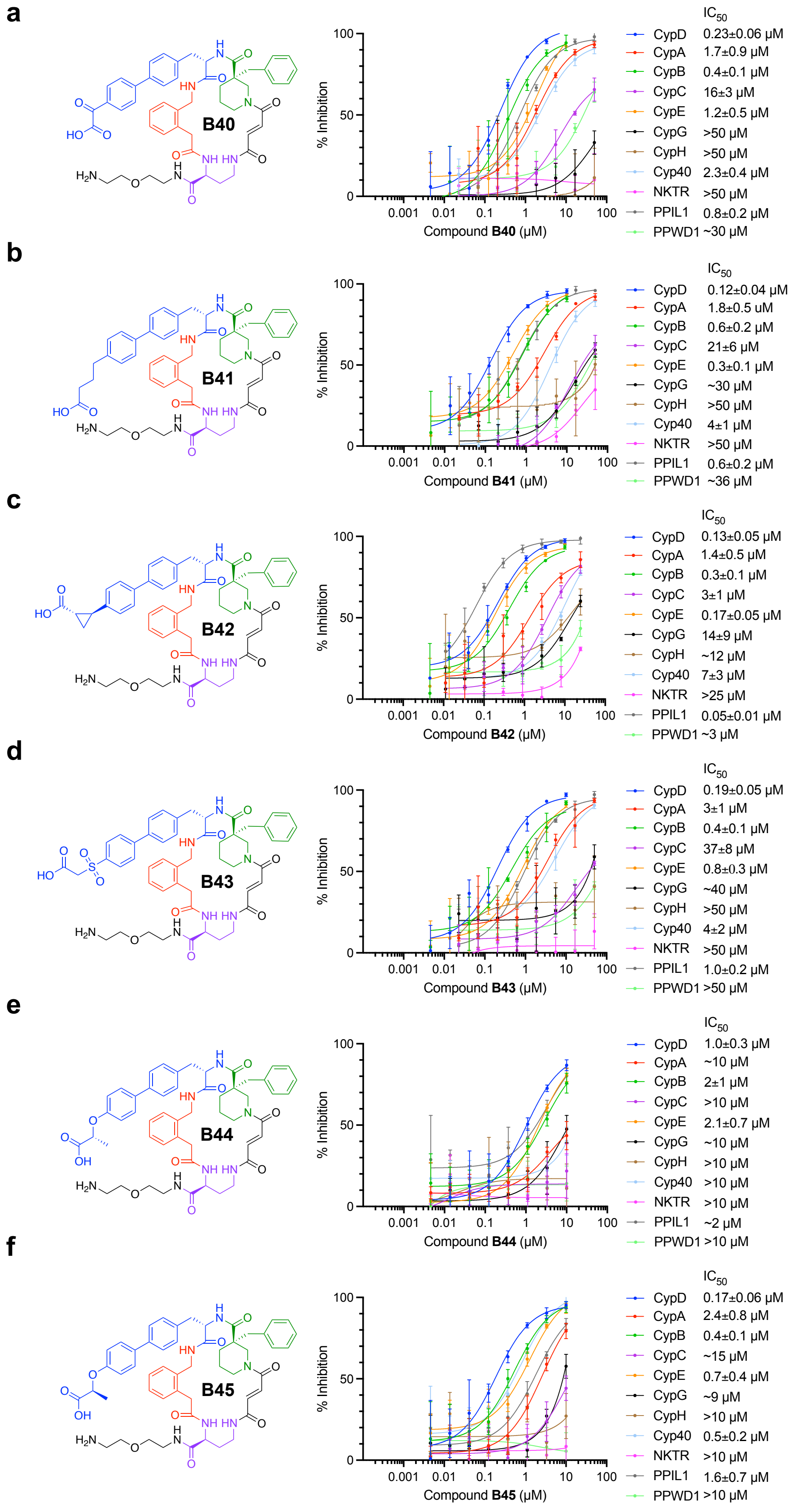

a
B40
HO
O
H2N
O
HN
NH HN
N
IC50
CypD 0.23±0.06 µM
CypA 1.7±0.9 µM
CypB 0.4±0.1 µM
CypC 16±3 µM
CypE 1.2±0.5 µM
CypG >50 µM
CypH >50 µM
Cyp40 2.3±0.4 µM
NKTR >50 µM
PPIL1 0.8±0.2 µM
PPWD1 ~30 µM
% Inhibition
Compound B40 (µM)
b
B41
IC50
CypD 0.12±0.04 µM
CypA 1.8±0.5 uM
CypB 0.6±0.2 µM
CypC 21±6 µM
CypE 0.3±0.1 µM
CypG ~30 µM
CypH >50 µM
Cyp40 4±1 µM
NKTR >50 µM
PPIL1 0.6±0.2 µM
PPWD1 ~36 µM
Compound B41 (µM)
c
B42
IC50
CypD 0.13±0.05 µM
CypA 1.4±0.5 µM
CypB 0.3±0.1 µM
CypC 3±1 µM
CypE 0.17±0.05 µM
CypG 14±9 µM
CypH ~12 µM
Cyp40 7±3 µM
NKTR >25 µM
PPIL1 0.05±0.01 µM
PPWD1 ~3 µM
Compound B42 (µM)
d
B43
IC50
CypD 0.19±0.05 µM
CypA 3±1 µM
CypB 0.4±0.1 µM
CypC 37±8 µM
CypE 0.8±0.3 µM
CypG ~40 µM
CypH >50 µM
Cyp40 4±2 µM
NKTR >50 µM
PPIL1 1.0±0.2 µM
PPWD1 >50 µM
Compound B43 (µM)
e
B44
IC50
CypD 1.0±0.3 µM
CypA ~10 µM
CypB 2±1 µM
CypC >10 µM
CypE 2.1±0.7 µM
CypG ~10 µM
CypH >10 µM
Cyp40 >10 µM
NKTR >10 µM
PPIL1 ~2 µM
PPWD1 >10 µM
Compound B44 (µM)
f
B45
IC50
CypD 0.17±0.06 µM
CypA 2.4±0.8 µM
CypB 0.4±0.1 µM
CypC ~15 µM
CypE 0.7±0.4 µM
CypG ~9 µM
CypH >10 µM
Cyp40 0.5±0.2 µM
NKTR >10 µM
PPIL1 1.6±0.7 µM
PPWD1 >10 µM
Compound B45 (µM)

g

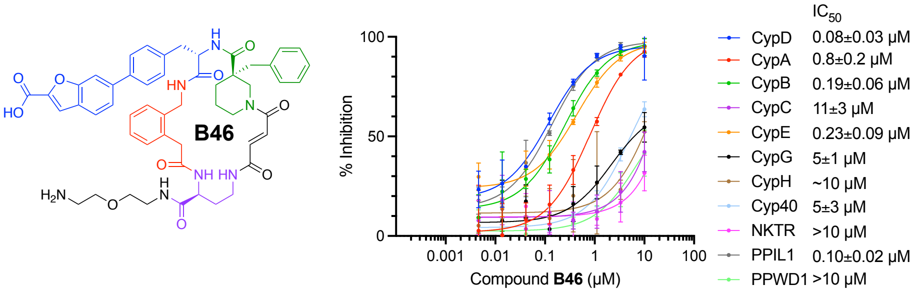

h

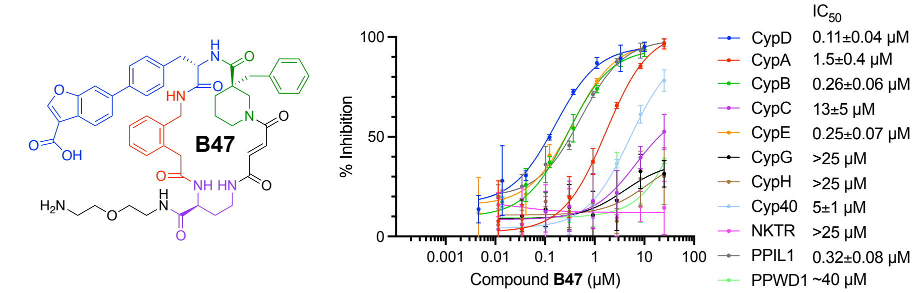

i

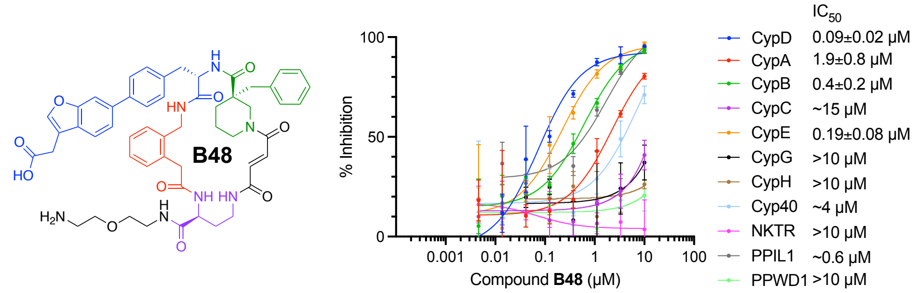

j

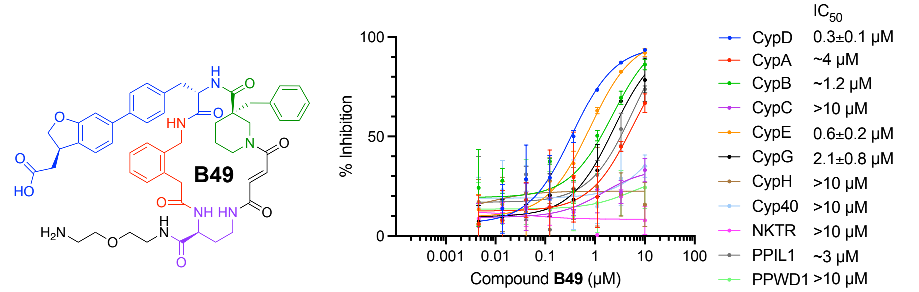

k

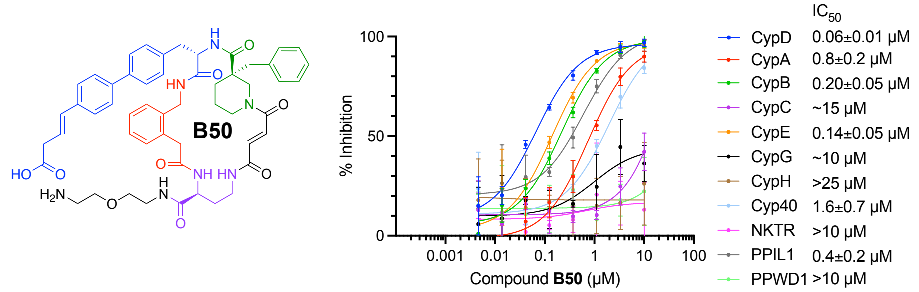

Supplementary Figure 17 | Derivatives of B23 that show reduced potency and selectivity for CypD. Structure and cyclophilin inhibition dose response data for **a, B40**; **b, B41**; **c, B42**; **d, B43**; **e, B44**; **f, B45**; **g, B46**; **h, B47**; **i, B48**; **j, B49**; and **k, B50**. Functionalization of the ethylene linker for **B23** commonly results in loss of potency and selectivity, shown in **B40**, **B42**, **B43**, **B44**, and **B45**, suggesting these extra functional groups interfere with the carboxylate's binding with

K118 and S119 residues. Extension of the linker further than **B23**'s ethylene (**B41** and **B50**) also reduces CypD potency. Other versions of structural rigidity introduced to the carboxylate, such as heterocycles, must be precisely placed, as **B42**, **B46**, **B47**, **B48**, and **B49** all show reduced potency compared to other heterocycle-carboxylates, such as **B37** and **B39** (Supplemental Fig. 16). IC_{50} values reflect mean±SEM of three technical replicates. Data points and error bars reflect mean±SD of individual assays at one dose.

a

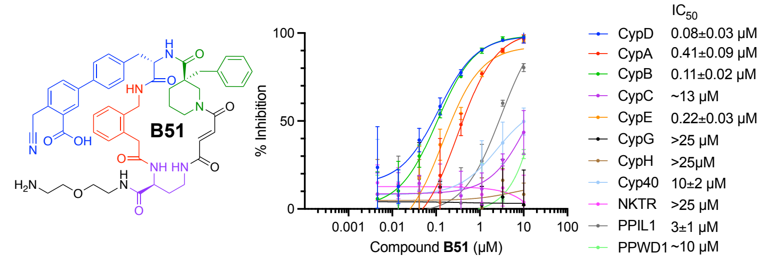

b

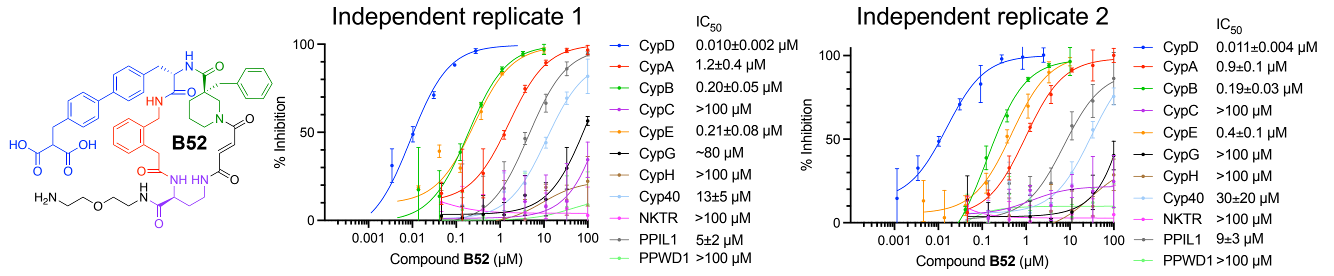

B52 IC$_{50}$	
CypD	0.010±0.004 μM
CypA	1.1±0.3 μM
CypB	0.21±0.02 μM
CypC	>100 μM
CypE	0.31±0.09 μM
CypG	>100 μM
CypH	>100 μM
Cyp40	22±6 μM
NKTR	>100 μM
PPIL1	6±2 μM
PPWD1	>100 μM

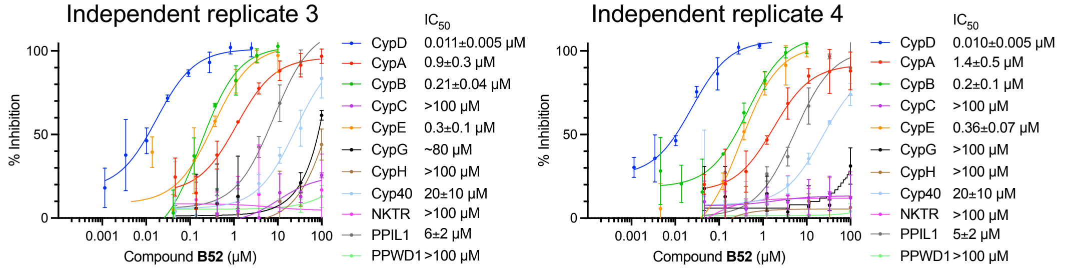

c

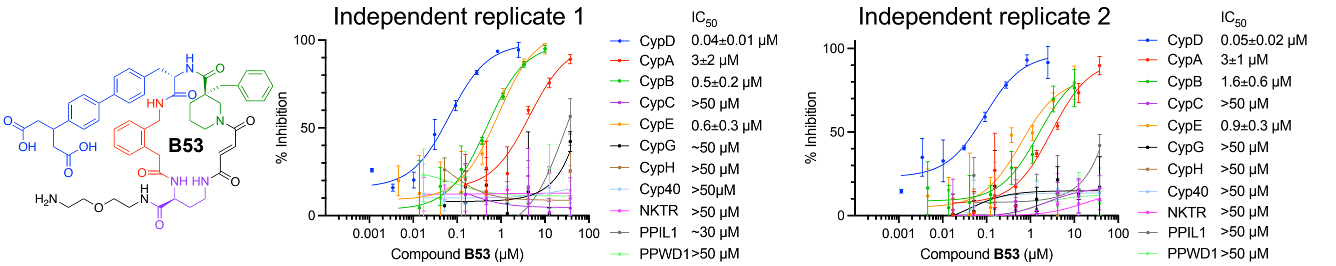

B53 IC$_{50}$	
CypD	0.057±0.009 μM
CypA	3.4±0.3 μM
CypB	1.1±0.5 μM
CypC	>50 μM
CypE	0.8±0.2 μM
CypG	>50 μM
CypH	>50 μM
Cyp40	>50 μM
NKTR	>50 μM
PPIL1	>50 μM
PPWD1	>50 μM

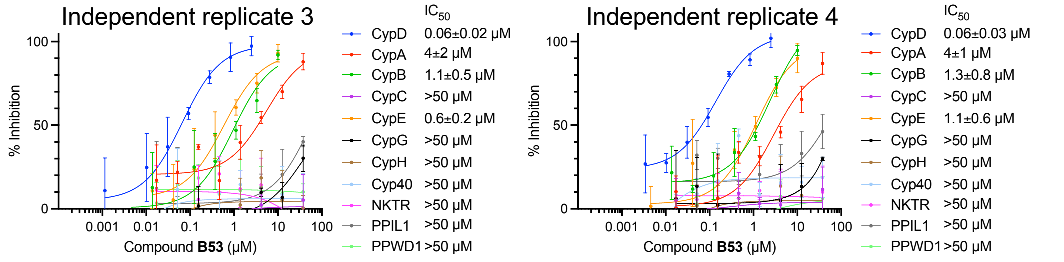

Supplementary Figure 18 | B23 derivatives that can simultaneously interact with CypD residues K118 and S119 and present a carboxylate near gatekeeper residues. Structure and cyclophilin inhibition dose response data for **a, B51**; **b, B52**; and **c, B53**. Nitrile derivatives that previously showed high potency but poor selectivity for CypD (Supplemental Fig. 12) were functionalized with a carboxylate, shown in **B51**. This change resulted in loss of potency, but similar selectivity profiles as **B23**. A large improvement in selectivity was observed when dicarboxylate groups were used. For both **B52** and **B53**, one carboxylate is oriented to interact with K118 and S119, while the second carboxylate is directed towards the gatekeeper residue

S123 of CypD (Fig. 4c). For **B52** and **B53**, four independent replicates (each comprising three technical replicates) are shown, with their respective fitted values that reflect mean±SEM of three technical replicates. Data points and error bars reflect mean±SD of individual assays at one dose. Tables show final reported IC_{50} values that reflect mean±SD of four independent replicates, who's values are used for the entirety of this work. Independent replicate 1 was collected with a separately synthesized compound batch than independent replicates 2, 3, and 4. For **B51**, IC_{50} values reflect mean±SEM of three technical replicates, with data points and error bars reflecting mean±SD of individual assays at one dose.

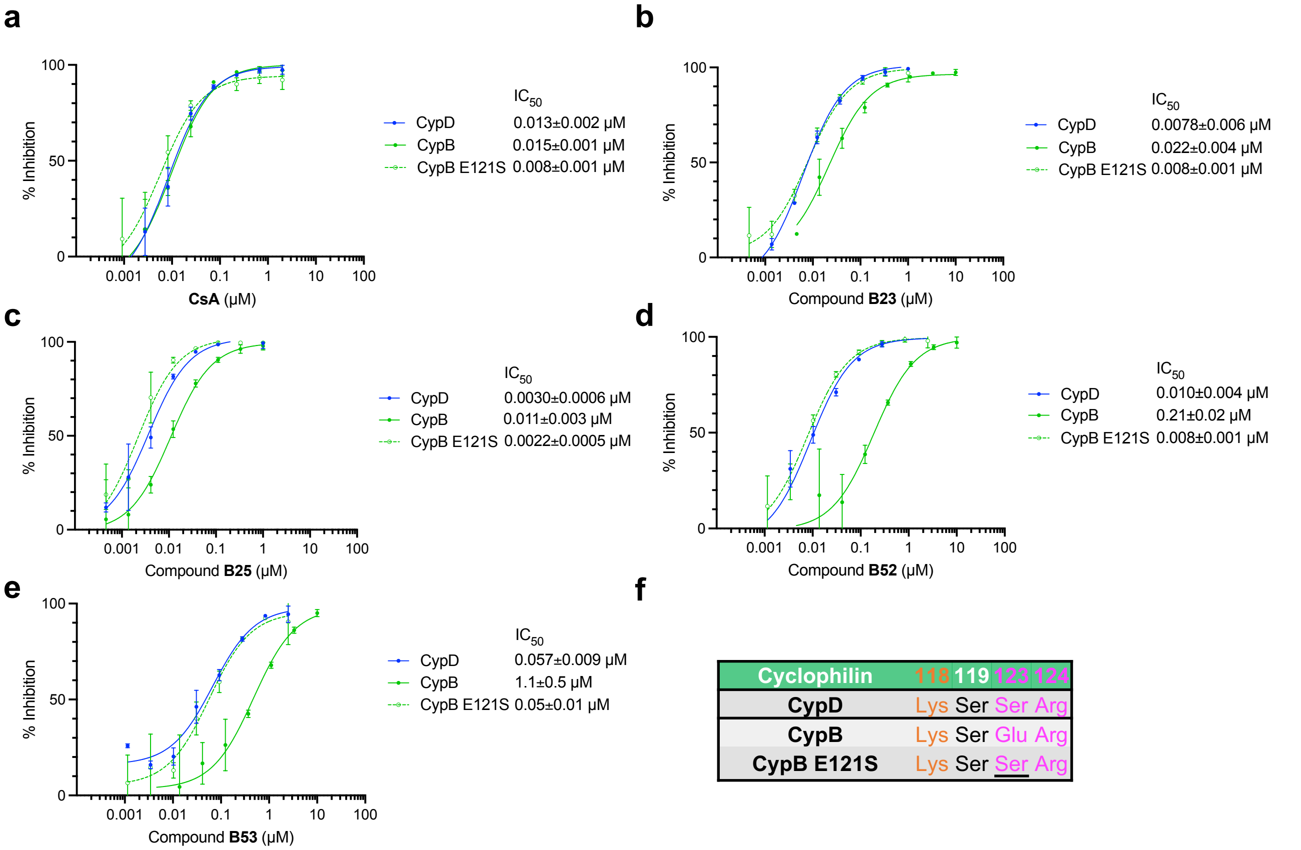

Cyclophilin	118	119	123	124
CypD	Lys	Ser	Ser	Arg
CypB	Lys	Ser	Glu	Arg
CypB E121S	Lys	Ser	Ser	Arg

Supplementary Figure 19 | The CypB E121S gatekeeper mutant is inhibited more potently compared to wild-type CypB by carboxylate-containing inhibitors. Dose response curves of CypD, CypB, and CypB E121S, shown for **a, CsA**; **b, B23**; **c, B25**; **d, B52**: and **e, B53**. **f,** List of residues on the far side of the S2 pocket of cyclophilins that are proximal to ligand carboxylates, with the CypB mutation underlined**. CsA** as an active site ligand does not show appreciable differences in potency between wild-type cyclophilins and the tested cyclophilin mutants. For carboxylate-containing inhibitors, wild-type CypB shows attenuated potency compared to wild-type CypD. Carboxylate-containing compounds inhibit wild-type CypD and CypB E121S equipotently, as this CypB mutant contains S2 pocket residues that mimic those in CypD's S2 pocket. For wild-type CypD and CypB IC_{50} data with **B52** and **B53**, values reflect mean±SD of four independent replicates (each comprising three technical replicates). Graphs show a representative single independent replicate (Independent replicate 1 is shown, containing three technical replicates) with data points and error bars reflecting mean±SD of individual assays at one dose. Further independent replicates are shown in Supplementary Fig. 18b-c. All other IC_{50} values reflect mean±SEM of three technical replicates, with data points and error bars reflecting mean±SD of individual assays at one dose.

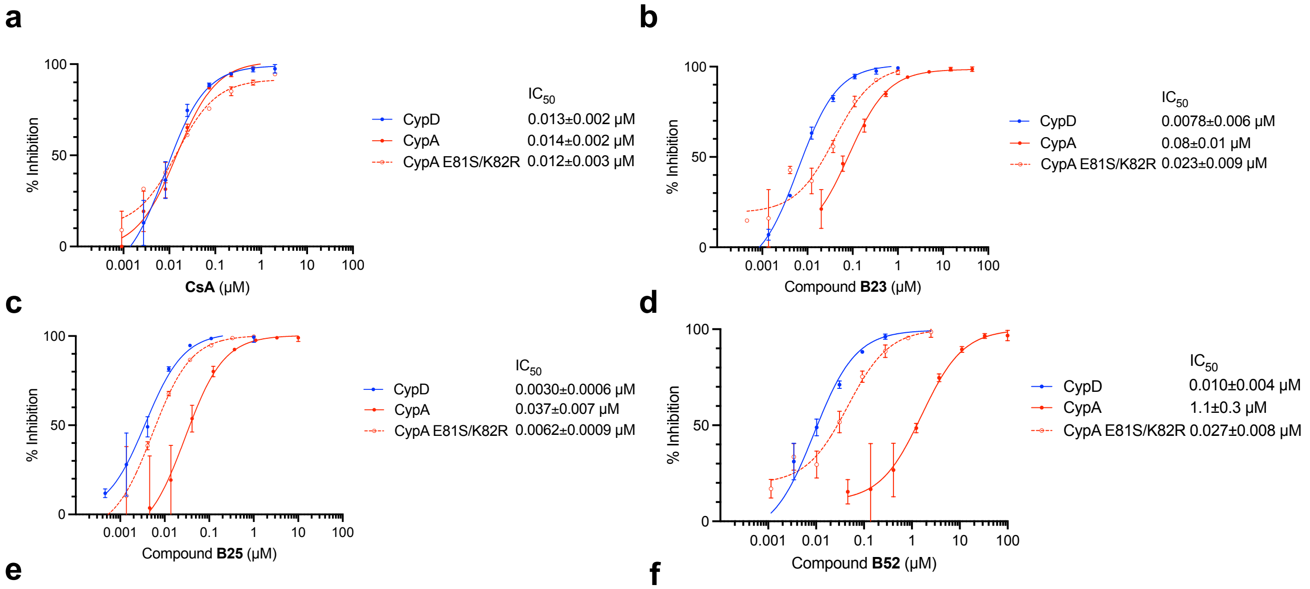

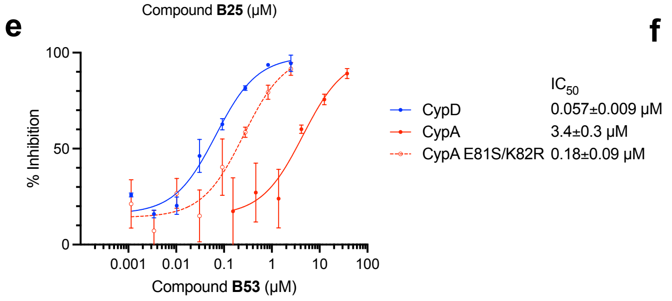

f

Cyclophilin	118	119	123	124
**CypD**	Lys	Ser	Ser	Arg
**CypA**	Lys	Ser	Glu	Lys
**CypA E81S/K82R**	Lys	Ser	Ser	Arg

Supplementary Figure 20 | The CypA E81S/K82R gatekeeper mutant is inhibited more potently compared to wild-type CypA with carboxylate-containing inhibitors. Dose response curves of CypD, CypA, and CypA E81S/K82R, shown for **a, CsA**; **b, B23**; **c, B25**; **d, B52**; **e, B53**. **f,** List of residues on the far side of the S2 pocket of cyclophilins that are proximal to the ligand carboxylates, with CypA mutations underlined**. CsA** as an active site ligand does not show appreciable changes in potency between the wild-type cyclophilins and mutants. For carboxylate-containing inhibitors, wild-type CypA shows attenuated potency compared to wild-type CypD. Carboxylate-containing compounds inhibit wild-type CypD and CypA E81S/K82R equipotently, as this CypA mutant contains S2 pocket residues that mimic those in CypD's S2 pocket. For CypD and CypA wild-type IC_{50} data with **B52** and **B53**, values reflect mean±SD of four independent replicates (each comprising three technical replicates). Graphs show a representative single independent replicate (Independent replicate 1 shown, containing three technical replicates) with data points and error bars reflecting mean±SD of individual assays at one dose. Further independent replicates are shown in Supplementary Fig. 18b-c. All other IC_{50} values reflect mean±SEM of three technical replicates, with data points and error bars reflecting mean±SD of individual assays at one dose.

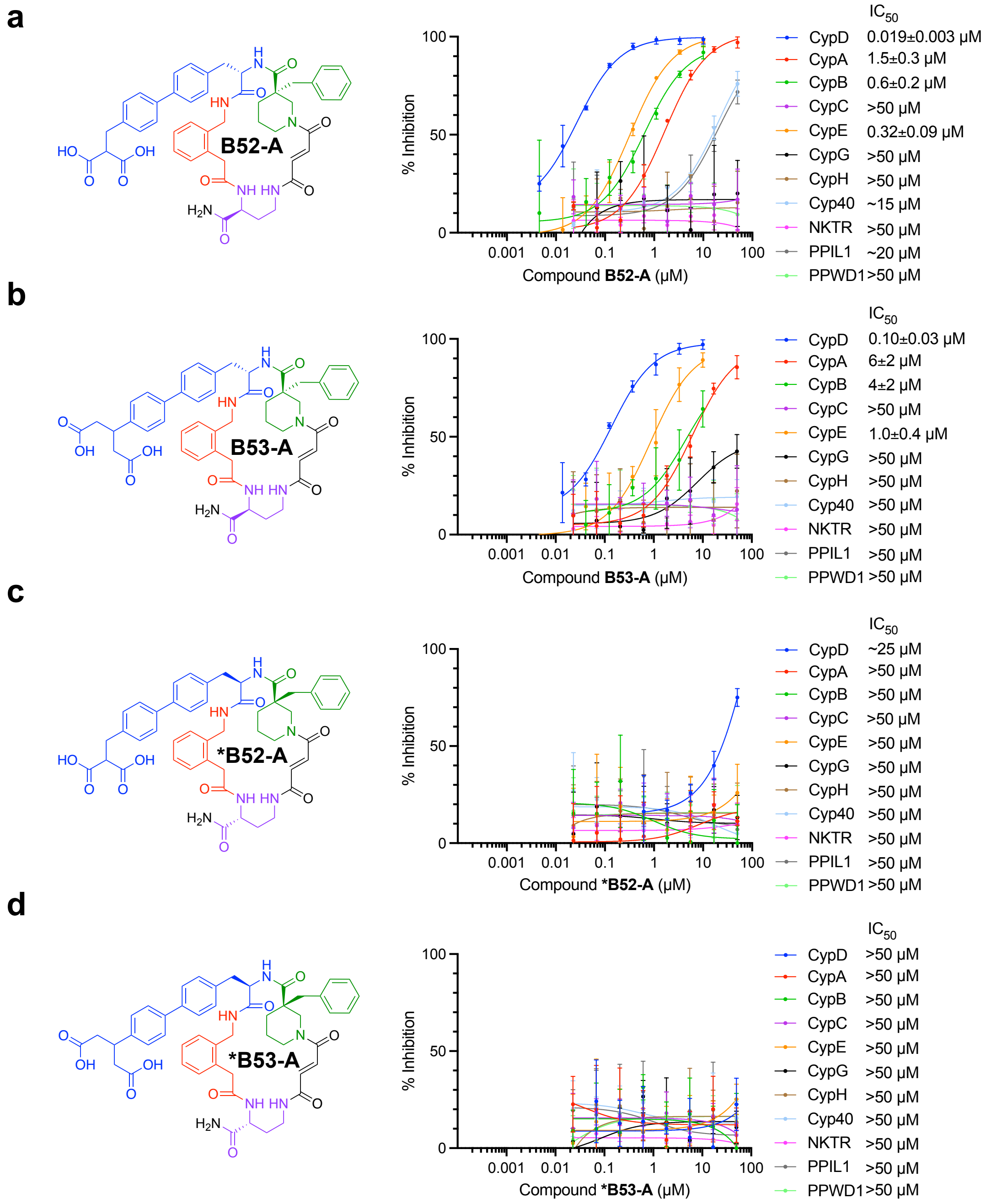

Supplementary Figure 21 | Prolyl isomerase inhibition of additional CypD-selective inhibitors. Structure and cyclophilin inhibition dose response data for **a, B52-A**; **b, B53-A**; **c, *B52-A**; and **d, *B53-A**. **B52-A** and **B53-A** retain the same selectivity profile as their associated analogs **B52** and **B53**, respectively, with a 2- to 3-fold decrease in potency. Enantiomers ***B52-A** and ***B53-A** show no substantial inhibition activity on any cyclophilins tested. IC_{50} values reflect mean±SEM of three technical replicates. Data points and error bars reflect mean±SD of individual assays at one dose.

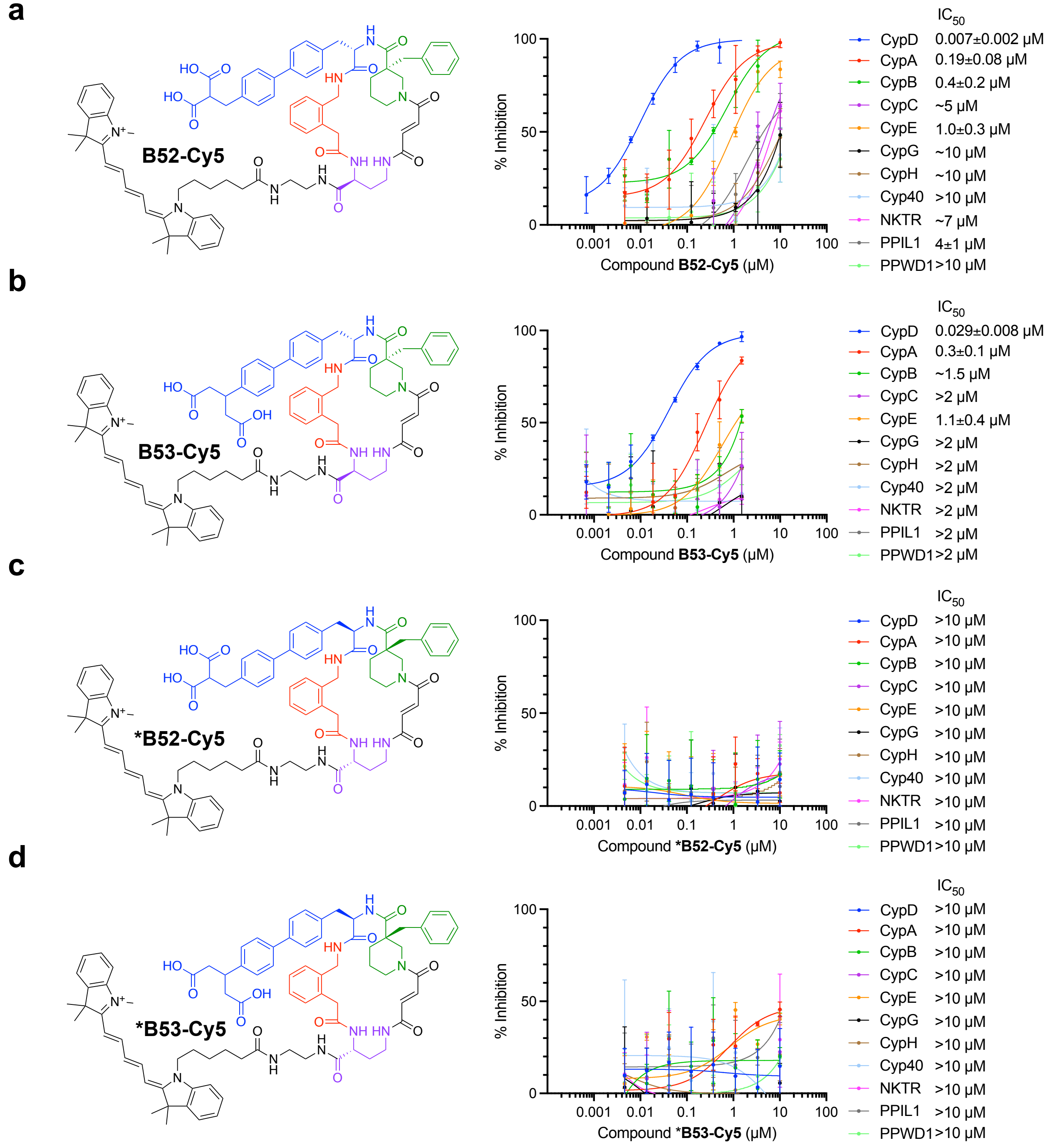

Supplementary Figure 22 | Prolyl isomerase inhibition by CypD-selective inhibitors used in mitochondrial models of mPTP. Structure and cyclophilin inhibition dose response data for **a, B52-Cy5**; **b, B53-Cy5**; **c, *B52-Cy5**; and **d, *B53-Cy5**. **B52-Cy5** and **B53-Cy5** retain the same selectivity profile as their associated analogs, **B52** and **B53**, respectively. Enantiomers ***B52-Cy5** and ***B53-Cy5** show no substantial inhibition profile on any cyclophilins tested. IC_{50} values reflect mean±SEM of three technical replicates. Data points and error bars reflect mean±SD of individual assays at one dose.

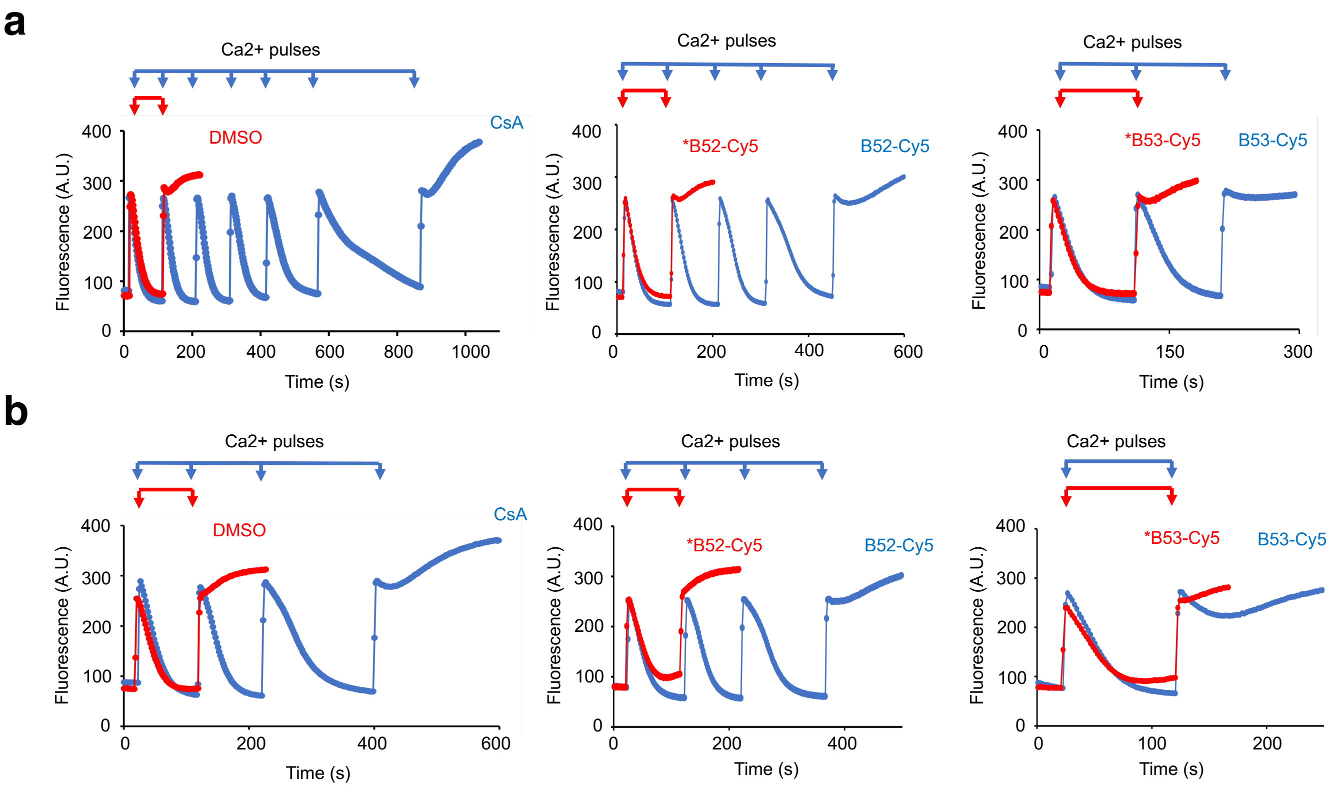

Supplementary Figure 23 | Additional independent replicates of calcium retention assays in mitochondria. The calcium retention capacity was determined in additional preparations of isolated mouse liver mitochondria (0.5 µg/mL) in response to pulses of 60 µM $CaCl_2$ in the presence of the indicated CypD inhibitors (or inactive enantiomers). Concentrations used were 2 µM **CsA**, 10 µM **B52-Cy5**, 10 µM ***B52-Cy5**, 20 µM **B53-Cy5**, and 20 µM ***B53-Cy5**. Mitochondrial uptake of extra-mitochondrial Ca^{2+} was assessed by monitoring the fluorescence of Calcium-green 5n, depicted in arbitrary units (A.U.). The rapid increase in fluorescence after several pulses of Ca^{2+} are taken up corresponds to mitochondrial Ca^{2+} release via mPTP. **a** All assays performed on the same mitochondrial preparation and day. **b**, All assays performed on a different mitochondrial preparation and day.

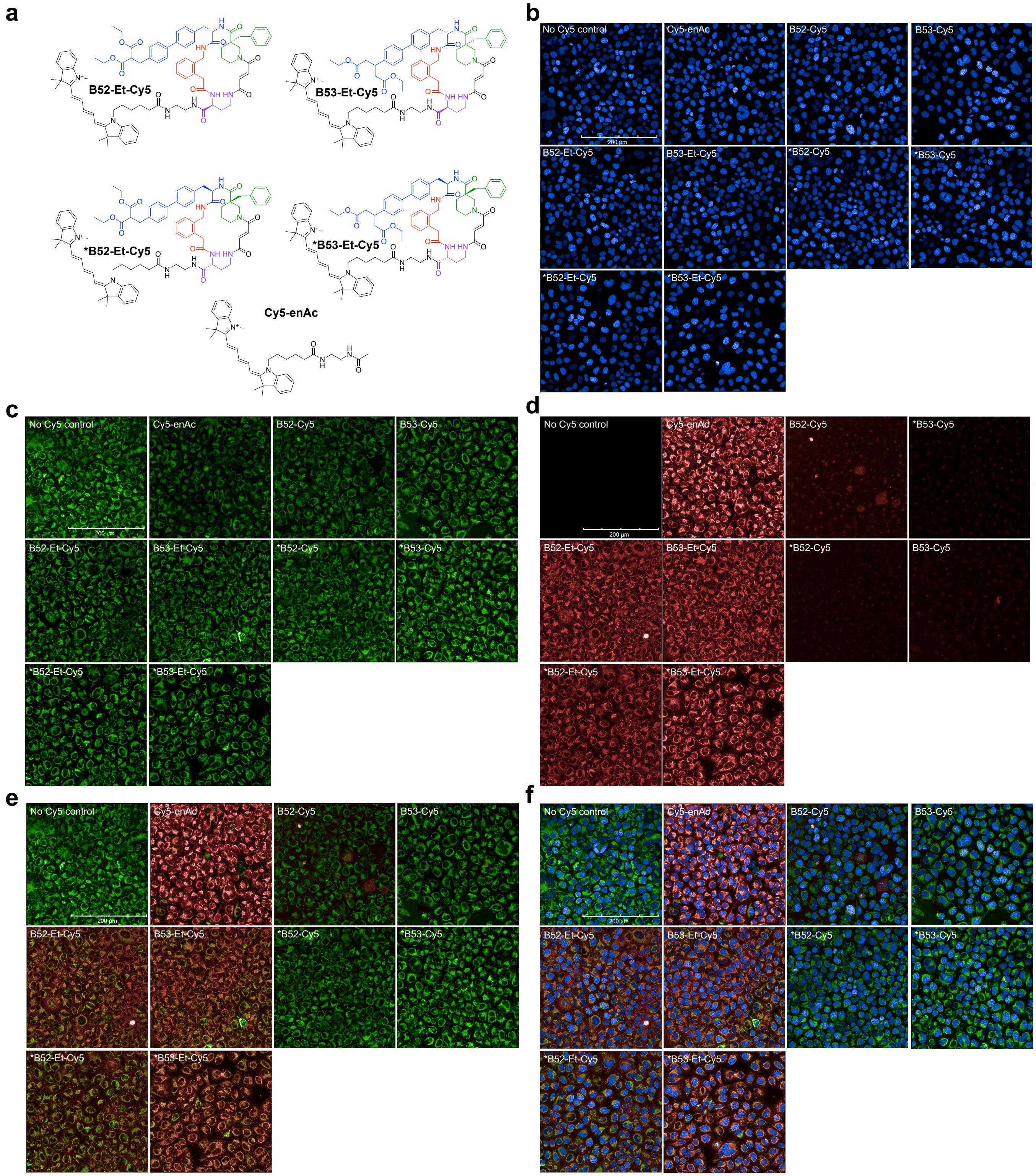

Supplementary Figure 24 | Representative images of Cy5-conjugated CypD inhibitor localization in HeLa cells. HeLa cells were co-treated with Cy5 conjugated compounds shown in (**a**) and Supplemental Fig. 22, and co-stained with Mitotracker Green, Hoechst 33342 and imaged by fluorescence microscopy. Images include the following channels; **b,** Hoechst 33342 nuclear stain; **c**, Mitotracker Green mitochondrial stain; **d**, Deep red channel showing Cy5-

conjugated compound; **e**, overlay of Mitotracker Green and Cy5 channels; **f**, overlay of Hoechst 33342, Mitotracker Green, and Cy5 channels. **Cy5-enAc** (and ester derivatives **B52-Et-Cy5**, **B53-Et-Cy5**, ***B52-Et-Cy5**, and ***B53-Et-Cy5** show both good mitochondrial localization via overlap with Mitotracker Green and sufficient mitochondrial fluorescence. In contrast, dicarboxylate derivatives **B52-Cy5**, **B53-Cy5**, ***B52-Cy5**, and ***B53-Cy5** show poor mitochondrial localization and fluorescence. Microscopy images shown are a representative image of three technical replicates. Scale bars, 200 μm.

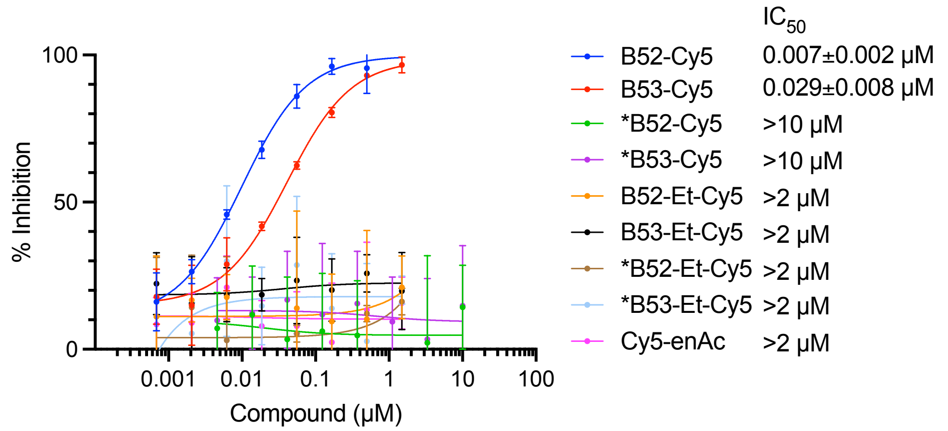

Supplementary Figure 25 | CypD inhibition of Cy5-conjugated CypD inhibitors. CypD prolyl-isomerase inhibition of Cy5-conjugated compounds is shown. Only compounds with correct stereochemistry and exposed dicarboxylate moieties are potent CypD inhibitors. **Cy5-enAc** alone does not inhibit CypD. IC_{50} values reflect mean±SEM of three technical replicates. Data points and error bars reflect mean±SD of individual assays at one dose.

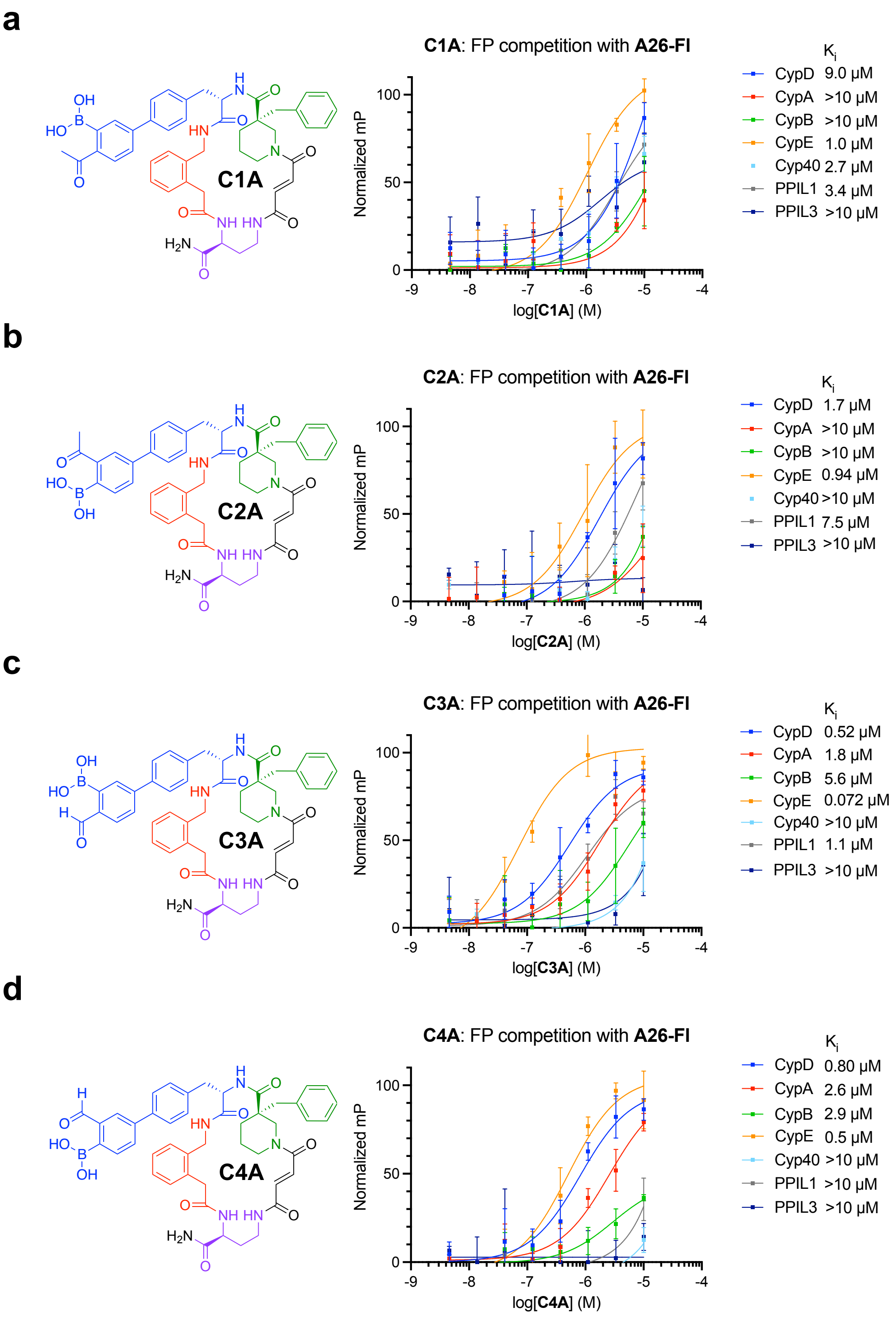

Supplementary Figure 26 | Fluorescence polarization competition with A26-Fl against lysine-containing cyclophilins. Structure and FP competition with 0.5 nM **A26-Fl** dose response data for **a, C1A**; **b, C2A**; **c, C3A**; and **d, C4A. C3A** shows selectivity for CypE, while regiosiomer **C4A** and acetyl derivatives **C1A** and **C2A** show attenuated binding to CypE. Y-axes are normalized to internal control wells containing **A26-Fl** only (100%) and **A26-Fl** with cyclophilin (0%). Data points and error bars reflect mean±SD of three technical replicates. K_i values reflect the mean of three technical replicates.

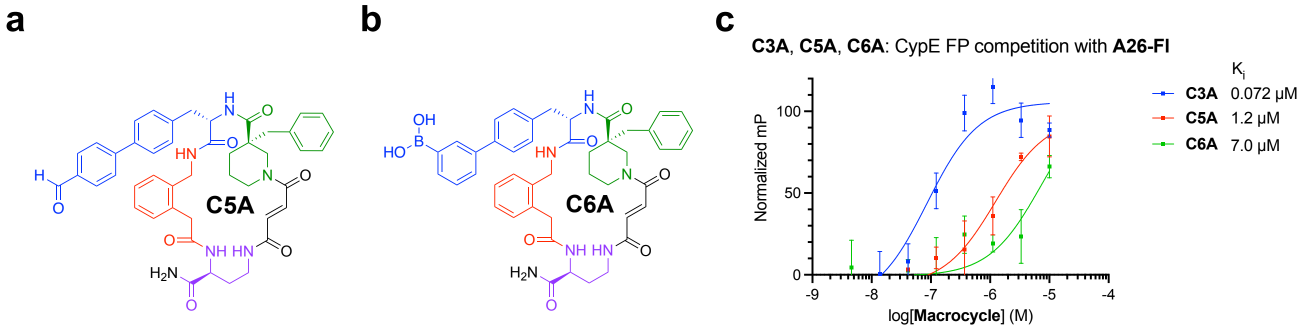

Supplementary Figure 27 | Fluorescence polarization competition with A26-Fl against CypE with control compounds C5A and C6A. Structures of compounds **a, C5A**; and **b, C6A**. **c,** Dose response of **C3A**, **C5A**, and **C6A** against CypE. Removal of either the aldehyde or boronic acid from **C3A** results in attenuated inhibition of CypE. Y-axes are normalized to internal control wells containing **A26-Fl** only (100%) and **A26-Fl** with cyclophilin (0%). Data points and error bars reflect mean±SD of three technical replicates. K_i values reflect mean of three technical replicates.

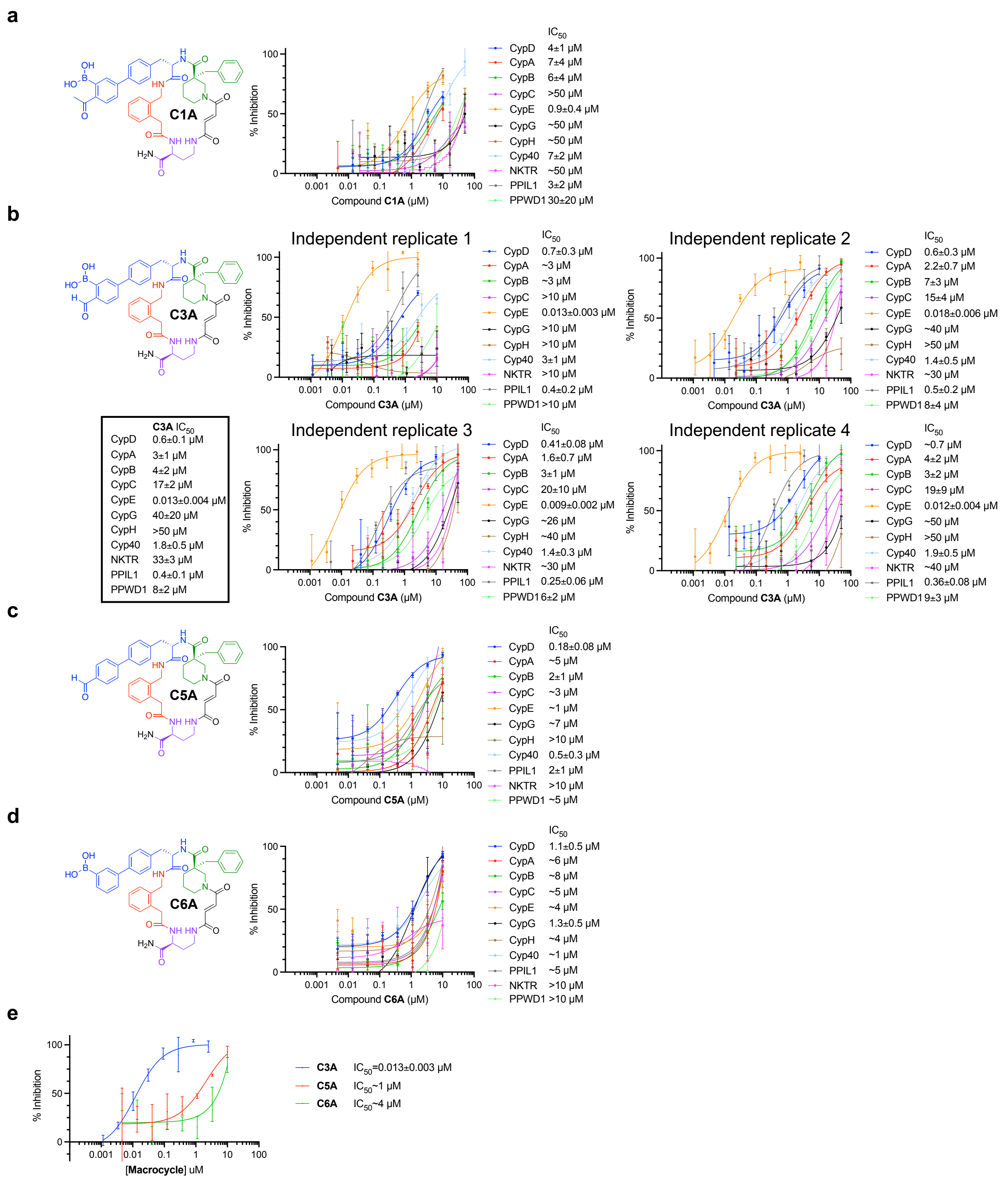

Supplementary Figure 28 | Prolyl isomerase inhibition by C1A, C3A, C5A, and C6A. Structure and cyclophilin inhibition dose response data for **a, C1A**; **b, C3A**; **c, C5A**; and **d, C6A**. **C3A** shows good selectivity and potency for CypE, while retaining only the aldehyde in **C4A** or the boronic acid in **C5A** results in loss in CypE potency and promiscuous cyclophilin inhibition**.**

Replacing the aldehyde with an acetyl group in **C1A** also results in loss of potency and selectivity for CypE. **e,** Dose response curves of **C3A**, **C4A**, and **C5A** against CypE, showing the necessity of both parts of the covalent warhead for CypE potency. For **C3A**, four independent replicates (each comprising three technical replicates) are shown, with their respective fitted values that reflect mean±SEM of three technical replicates. Data points and error bars reflect mean±SD of individual assays at one dose. Table shows final reported IC_{50} values that reflect mean±SD of four independent replicates for CypD, CypA, CypB, CypE, Cyp40 and PPIL1, and mean±SD of three independent replicates (Independent replicates 2-4) for CypC, CypG, CypH, NKTR, and PPWD1. Table IC_{50} values are used for the entirety of this work. Independent replicate 1 was collected with a separately synthesized compound batch than was used in independent replicates 2, 3, and 4.

a

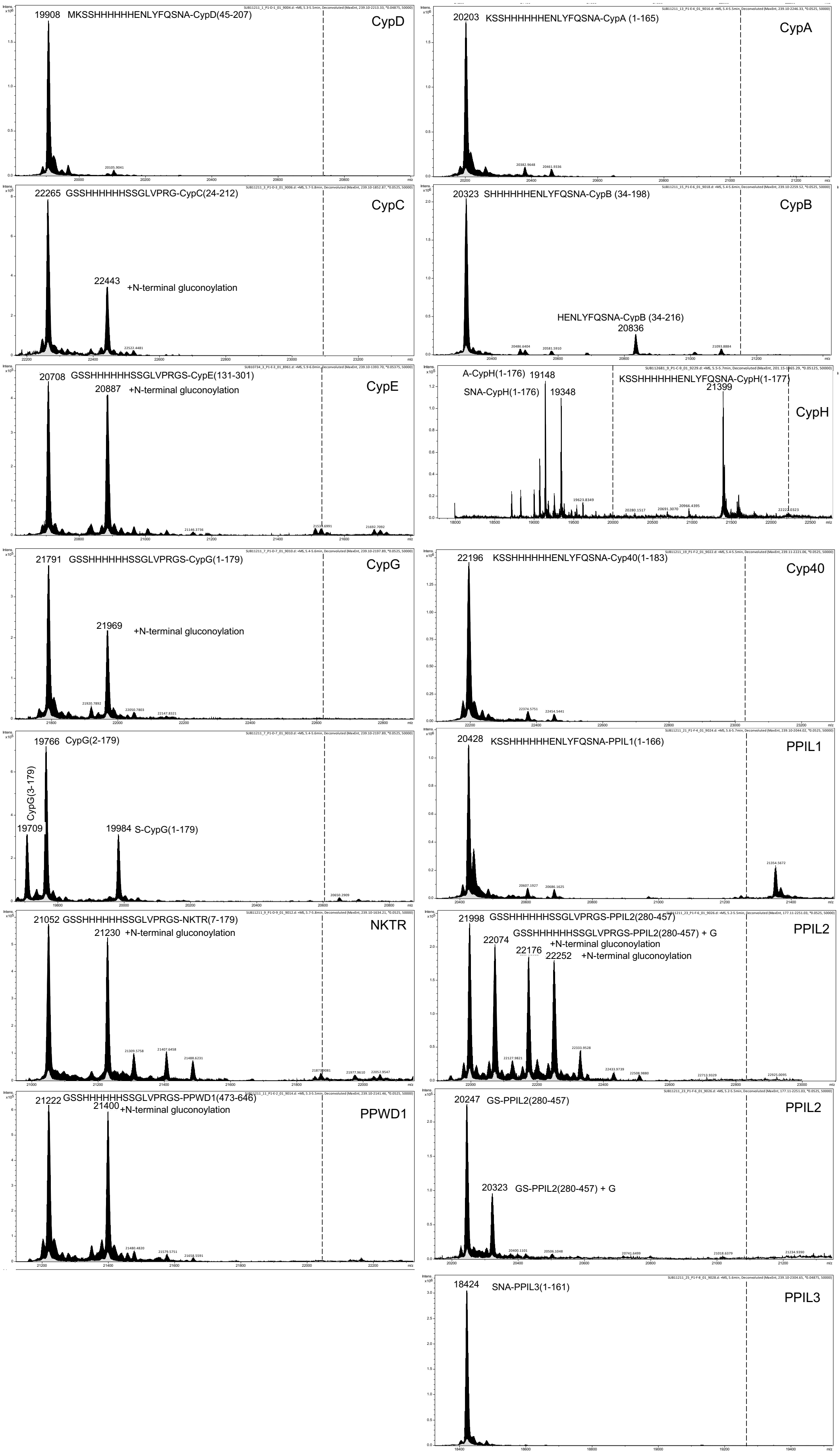
19908 MKSSHHHHHHENLYFQSNA-CypD(45-207)
CypD
20203 KSSHHHHHHENLYFQSNA-CypA (1-165)
CypA
22265 GSSHHHHHHSSGLVPRG-CypC(24-212)
CypC
22443 +N-terminal gluconoylation
20323 SHHHHHHENLYFQSNA-CypB (34-198)
CypB
HENLYFQSNA-CypB (34-216)
20836
20708 GSSHHHHHHSSGLVPRGS-CypE(131-301)
20887 +N-terminal gluconoylation
CypE
A-CypH(1-176) 19148
SNA-CypH(1-176) 19348
KSSHHHHHHENLYFQSNA-CypH(1-177)
21399
CypH
21791 GSSHHHHHHSSGLVPRGS-CypG(1-179)
CypG
21969 +N-terminal gluconoylation
22196 KSSHHHHHHENLYFQSNA-Cyp40(1-183)
Cyp40
19766 CypG(2-179)
CypG(3-179)
19709
19984 S-CypG(1-179)
20428 KSSHHHHHHENLYFQSNA-PPIL1(1-166)
PPIL1
21052 GSSHHHHHHSSGLVPRGS-NKTR(7-179)
21230 +N-terminal gluconoylation
NKTR
21998 GSSHHHHHHSSGLVPRGS-PPIL2(280-457)
22074 GSSHHHHHHSSGLVPRGS-PPIL2(280-457) + G
22176 +N-terminal gluconoylation
22252 +N-terminal gluconoylation
PPIL2
21222 GSSHHHHHHSSGLVPRGS-PPWD1(473-646)
21400 +N-terminal gluconoylation
PPWD1
20247 GS-PPIL2(280-457)
PPIL2
20323 GS-PPIL2(280-457) + G
18424 SNA-PPIL3(1-161)
PPIL3

b

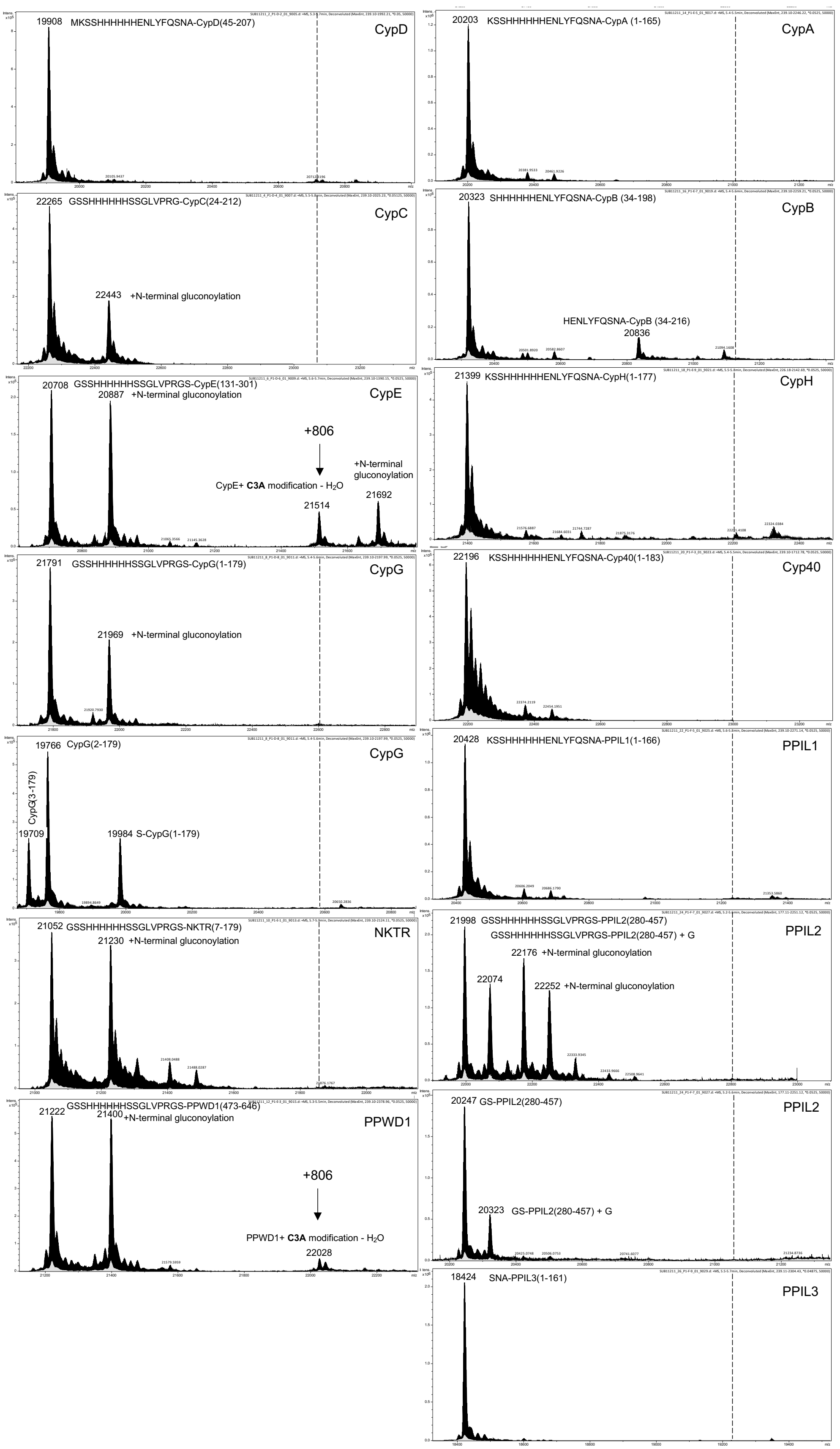

Supplementary Figure 29 | Mass spectroscopy analysis of C3A covalent modification on 13 cyclophilins. a, Mass spectroscopy analysis of lysine covalent modification and **b**, after

treatment with $NaCNBH_3$ to trap iminoboronate as lysine-modified secondary amine, highlighting relevant m/z regions. Primary protein peaks are shown along with expected region of **C3A** modification (+824: **C3A** modification non-reductive, represented as a dashed line for the most abundant ion peak(s) in panel **a**, +806: **C3A** –H_2O reductive amination covalent adduct, represented as a dashed line on cyclophilins with no observed covalent modification for the most abundant ion peak in panel **b**). We observed small amounts of covalent modification for PPWD1, however with a diminished peak intensity compared to CypE's +806 covalent adduct, suggesting minimal adduct formation in solution. We observed some C-terminal truncation for CypB of residues that are not anticipated to affect active site or S2 pocket integrity, prolyl-isomerase catalytic activity, or inhibitor binding. We also observed a mixture of CypH, CypG and PPIL2 constructs that include various cleaved and non-cleaved His-tag forms, with representative mass spectra shown for each primary peak. N-terminal His-tag or cleaved cyclophilin constructs are not anticipated to affect active site or S2 pocket integrity, prolyl-isomerase catalytic activity, or inhibitor binding. Various recombinantly expressed constructs show N-terminal gluconoylation. Some proteins when co-treated with $NaCNBH_3$ showed +14 mass off the primary peak not present in non-reductive conditions (see CypC, CypH, NKTR, Cyp40, and PPIL1) suggesting various methylation events via reductive amination.

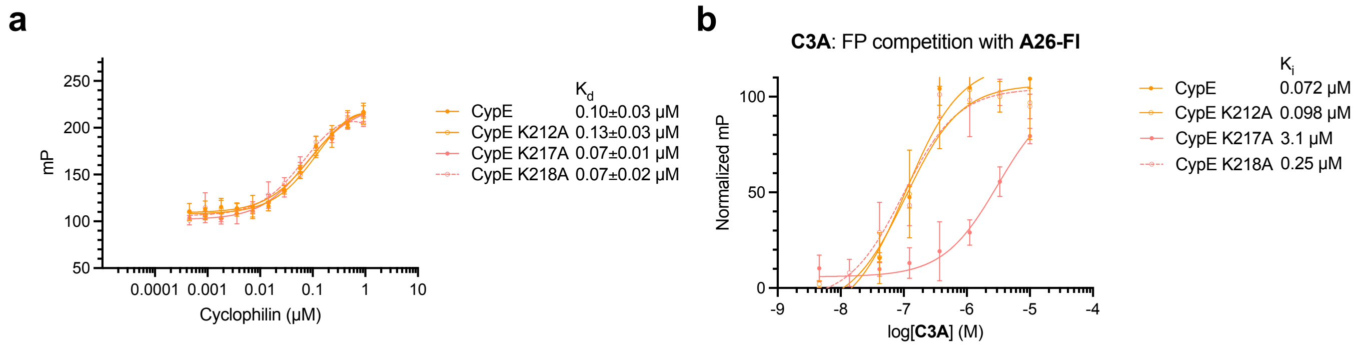

Supplementary Figure 30 | CypE mutant FP analysis. a, Protein titration against 0.5 nM **A26-Fl**. K_d values and error bars reflect mean±SEM of three technical replicates. Data points and error bars reflect SD of individual assays at one dose. **b,** FP competition with 0.5 nM **A26-Fl** dose response data **C3A** CypE proteins**. C3A** has significantly attenuated binding for only the K217A mutant compared to wild-type. Y-axes are normalized to internal control wells containing **A26-Fl** only (100%) and **A26-Fl** with cyclophilin (0%). Data points and error bars reflect mean±SD of three technical replicates. K_i values reflect the mean of three technical replicates.

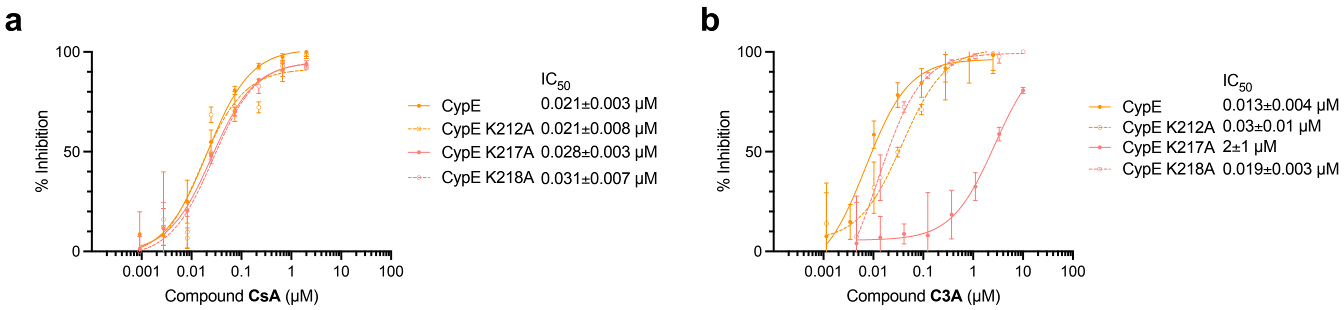

Supplementary Figure 31 | Prolyl isomerase inhibition screening of CypE lysine mutants reveals K217 residue as site of C3A covalent modification. Dose response curves of CypD, CypE K212A , CypE K217A, and CypE K218A shown for **a, CsA** and **b, C3A**. **CsA** as an active site ligand does not show appreciable differences in potency between wild-type CypE and the tested CypE mutants. Loss of **C3A** inhibition potency for only the K217A mutant compared to wild-type or K212A and K218A mutants verifies that K217 is the being covalently modified by **C3A**. For wild-type CypE IC_{50} data with **C3A**, values reflect mean±SD of four independent replicates (each comprising three technical replicates). Graphs show a representative single independent replicate (Independent replicate 3 is shown, containing three technical replicates) with data points and error bars reflecting mean±SD of individual assays at one dose. Further independent replicates are shown in Supplementary Fig. 28b. All other IC_{50} values reflect mean±SEM of three technical replicates, with data points and error bars reflecting mean±SD of individual assays at one dose.

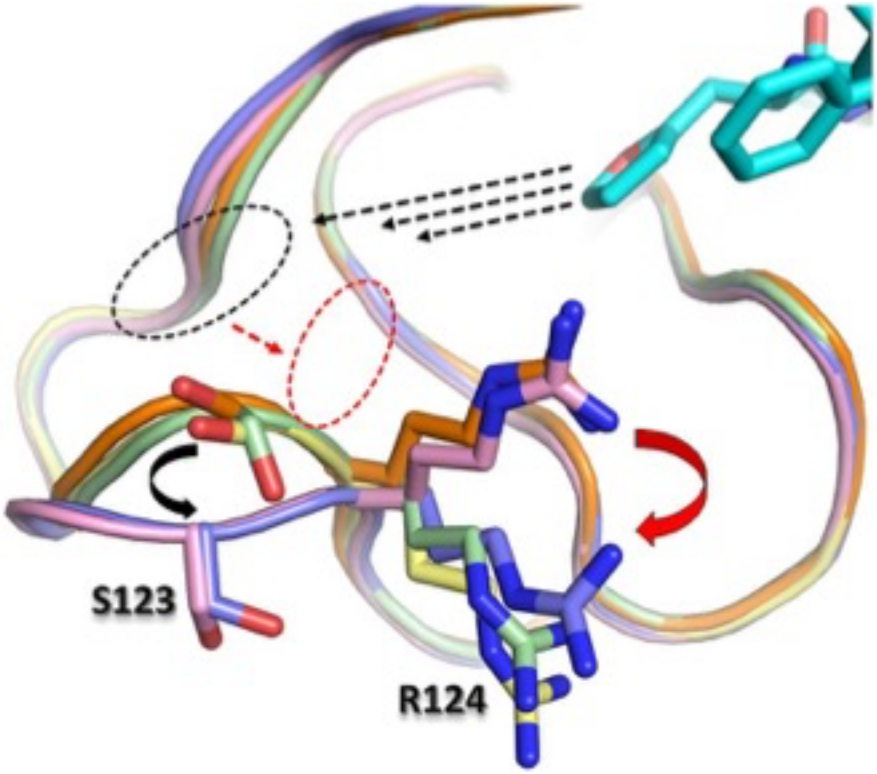

Supplementary Figure 32 | Ligand-dependent conformation of primary gatekeeper residues S123 and R124 in the S2 pocket. Superposition of co-crystal structures containing various inhibitors bound to CypD. **A26** co-crystal structure (orange) shown along with **A26** (cyan) as a reference for gatekeeper positions, shows little influence of ligand on residue conformation. Binding deeper into the S2 pocket (black arrows and circle) with larger carboxylate-containing ligands such as **B25** (pink) causes the S123 containing loop region to flip outwards of the pocket. Binding deeper and out of the pocket (red circles and arrows) causes the R124 residue to flip out of the pocket, shown by **B1** (green) and **B21** (yellow). Dicarboxylate-containing ligands such as **B53** (purple) exhibit conformational change of both the S123 loop as well as the side-chain of R124.

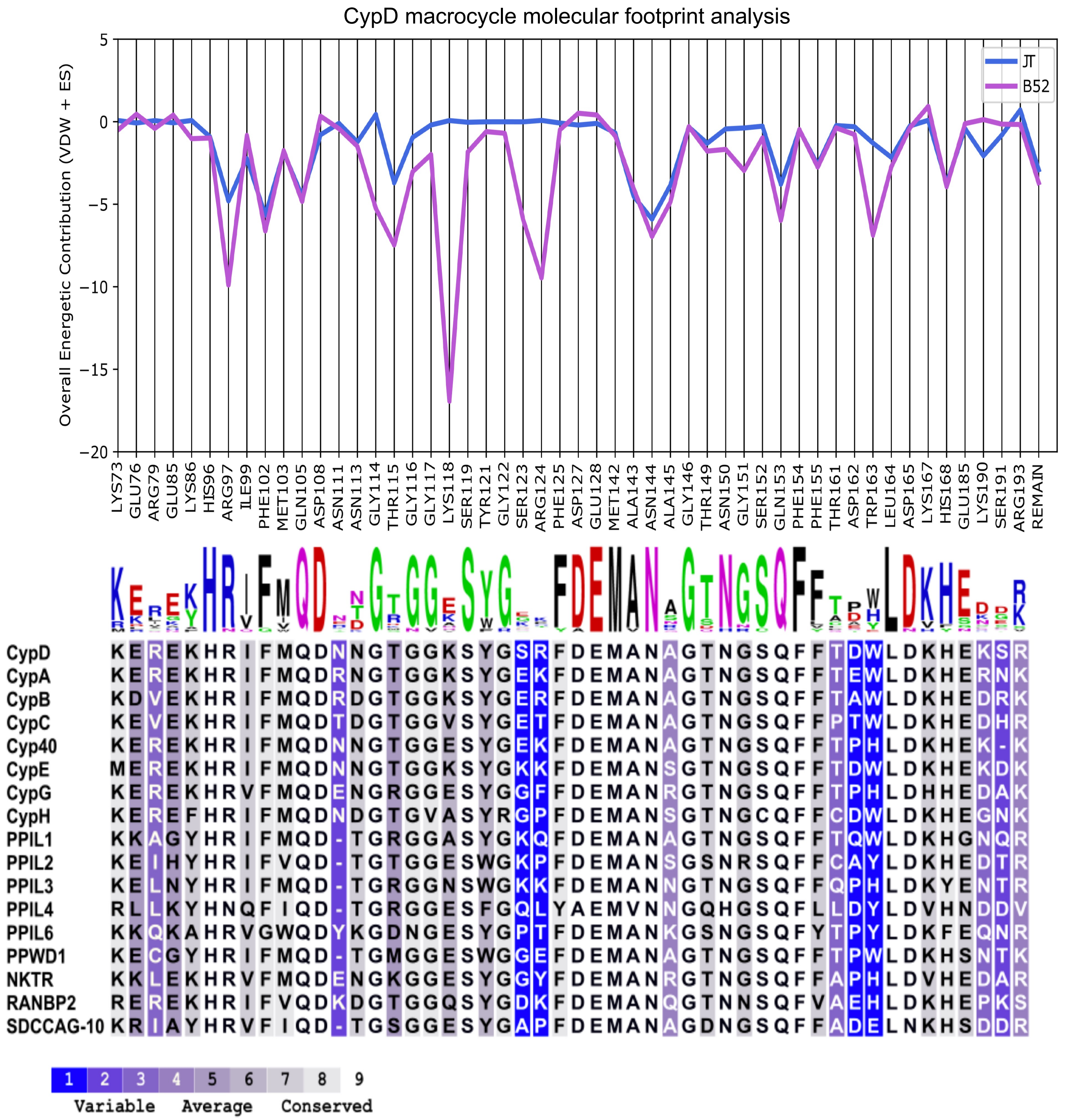

Supplementary Figure 33 | Molecular footprinting analysis demonstrates that key binding interactions of B52 are unique to CypD. Graph of the predicted overall energetic contribution (sum of Van der Waals and electrostatic energy) of the top fifty contributing residues in CypD to compounds **JOMBt** and **B52** aligned with a sequence alignment and web logo of all 17 human cyclophilins. Energetic contributions calculated using footprinting function in DOCK6.9. Coloring scheme of sequence alignment corresponds to sequence conservation score as calculated by the ConSurf webserver (https://consurf.tau.ac.il/). Note, this analysis shows R124 as a contributing residue for **B52** binding, in contrast to CypD mutant data, which suggested that this residue does not interact with **B52**. But we attribute this discrepancy to **B52**’s observed hydrogen bond with backbone atoms between S123 and R124, instead of side-chain moieties.

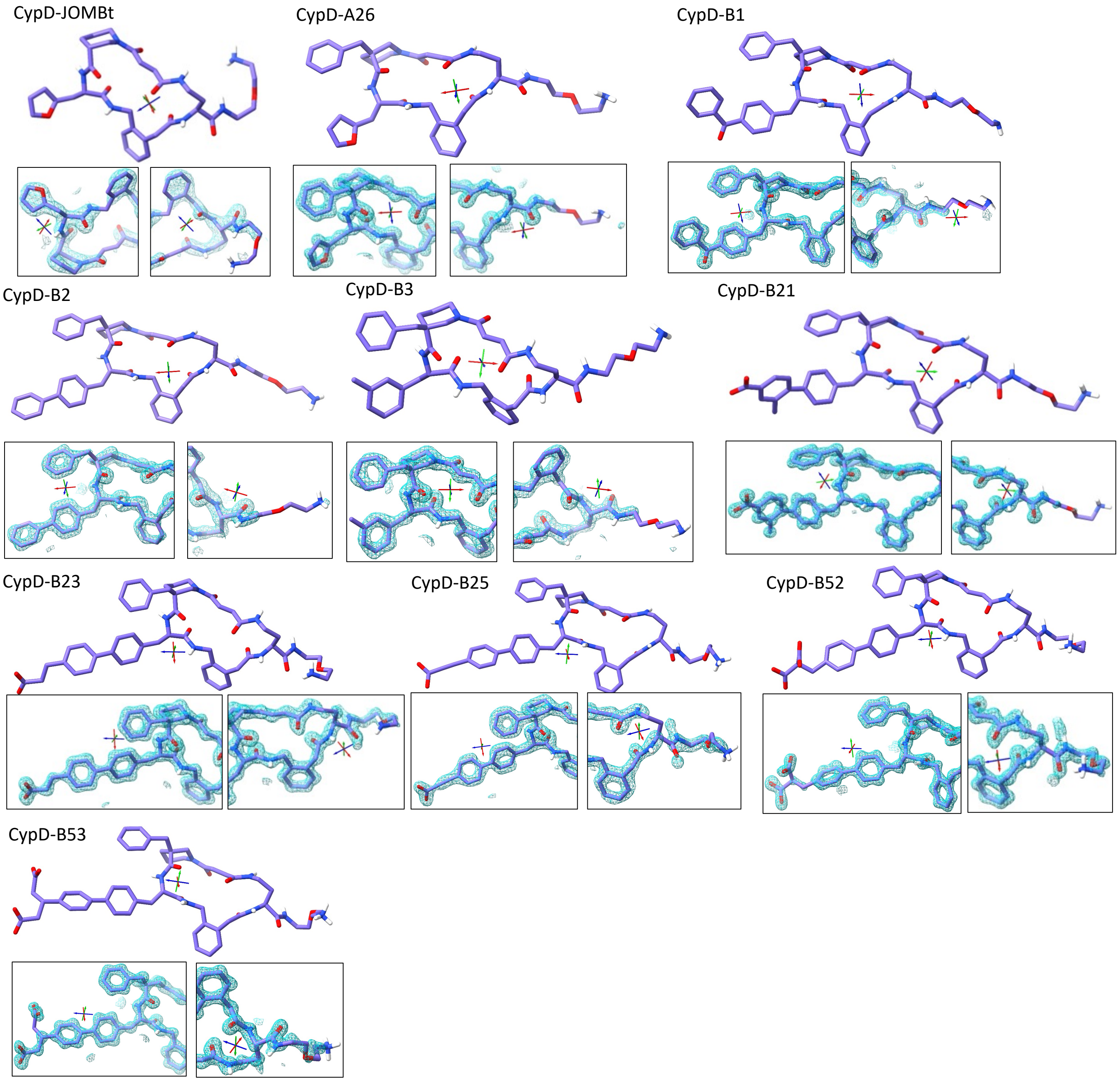

Supplementary Figure 34 | Electron densities of ligands from CypD-inhibitor co-crystal structures

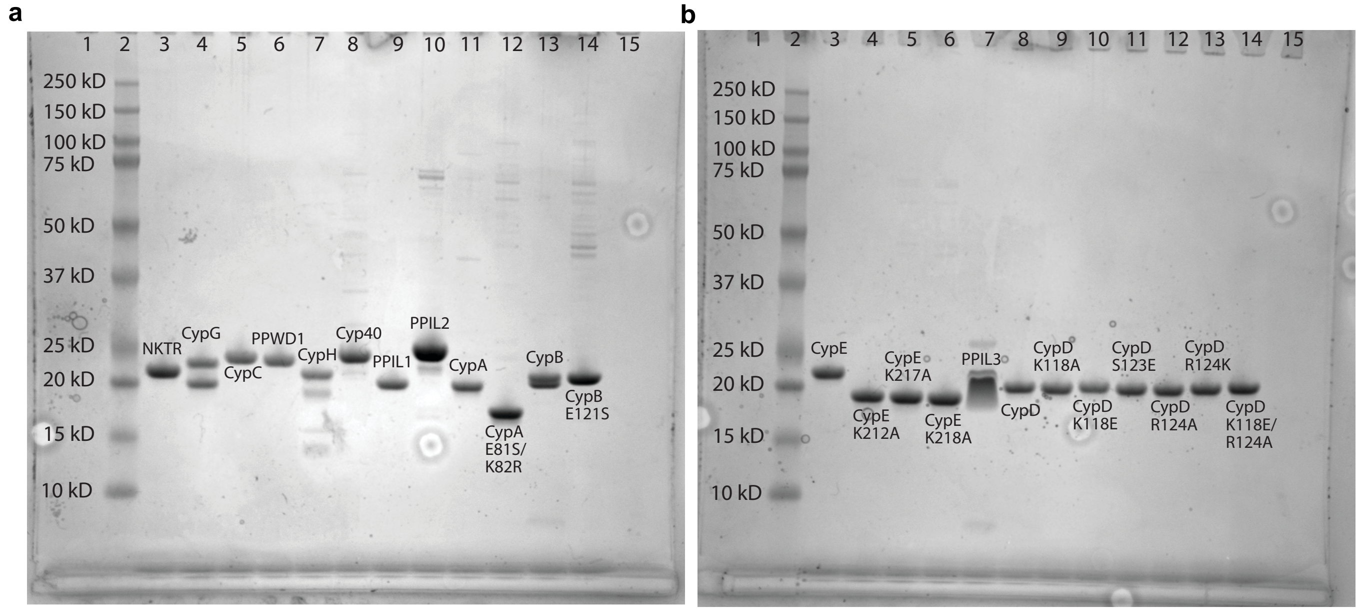

Supplementary Figure 35 | SDS-PAGE of recombinantly expressed cyclophilin proteins.
a, The gel shows representative samples of the following:
Lane 1-Blank;
Lane-2: Protein Ladder;
Lane 3- GSSHHHHHHSSGLVPRGS-NKTR(7-179);
Lane 4-Mixture of GSSHHHHHHSSGLVPRGS-CypG(1-179), S-CypG(1-179), CypG(2-179), CypG(3-179), see Supplementary Figure 29 for confirmation of mixture;
Lane 5- GSSHHHHHHSSGLVPRG-CypC(24-212);
Lane 6- GSSHHHHHHSSGLVPRGS-PPWD1(473-646);
Lane 7- Mixture of KSSHHHHHHENLYFQSNA-CypH(1-177), A-CypH(1-176), and SNA-CypH(1-176), Supplementary Figure 29 for confirmation of mixture;
Lane 8-KSSHHHHHHENLYFQSNA-Cyp40(1-183);
Lane 9- KSSHHHHHHENLYFQSNA-PPIL1(1-166);
Lane 10- Mixture of GS-PPIL2(280-457), GSSHHHHHHSSGLVPRGS-PPIL2(280-457), GS-PPIL2(280-457) + G, and GSSHHHHHHSSGLVPRGS-PPIL2(280-457) + G, see Supplementary Figure 29 for confirmation of mixture
Lane 11- KSSHHHHHHENLYFQSNA-CypA (1-165);
Lane 12- SNA-CypA E81S/K82R (1-165);
Lane 13- Mixture of SHHHHHHENLYFQSNA-CypB (34-198) and HENLYFQSNA-CypB (34-216); see Supplementary Figure 29 for confirmation of mixture
Lane 14- SNA-CypB E121S (34-216);
Lane 15-blank
b, The gel shows representative samples of the following:
Lane 1-Blank;
Lane-2: Protein Ladder;
Lane 3- GSSHHHHHHSSGLVPRGS-CypE(131-301);
Lane 4- GS-CypE K212A (131-301);
Lane 5- GS-CypE K217A (131-301);
Lane 6- GS-CypE K218A (131-301);
Lane 7- SNA-PPIL3(1-161);
Lane 8- MKSSHHHHHHENLYFQSNA-CypD(45-207);

Lane 9- MKSSHHHHHHENLYFQSNA-CypD K118A (45-207);
Lane 10- MKSSHHHHHHENLYFQSNA-CypD K118E (45-207);
Lane 11- MKSSHHHHHHENLYFQSNA-CypD S123E (45-207);
Lane 12- MKSSHHHHHHENLYFQSNA-CypD R124A (45-207);
Lane 13- MKSSHHHHHHENLYFQSNA-CypD R124K (45-207);
Lane 14- MKSSHHHHHHENLYFQSNA-CypD K118E/R124A (45-207);
Lane 15-blank.
All wild-type recombinant proteins were also verified by LC-MS through experiments conducted in Supplemental Figure 29.

Supplementary Note 1

Python Scripts

Analysis of CypD selection high-throughput sequencing data

```
a few notes about this code:
* assumes your files fastq files are all unzipped (not fastq.gz's) and in the same folder
* set your filepath and directory of this folder below
* assumes filenames follow the form 'proteinName_L001_R1_001.fastq' (MiSeq output format).
* the 'S#' indices (e.g. S1) should be mapped to whatever variable bases
 are in the primer in a separate .csv (barcode keys.csv), saved in the same file as your
 fastq data (in the form ['S1' , 'TCACT']). these should be the first bases in each line
 of the sequence.
* don't put underscores in the sample filename, it messes up the regex to do the offset
 mapping (e.g. use input-library instead of input_library)

'''

import collections   #for Counter
#import itertools
import glob       #useful for getting all files in a directory
import os        # for filepaths http://stackoverflow.com/questions/3964681/find-all-files-in-directory-with-extension-
txt-with-python
import re        # for regex
import csv       # for converting to/from csv
#import pandas      # for dealing with arrays (calculating enrichments)


'''***CHANGE THE FILEPATH***'''

#first, find the sequence files, place filepath below
filepath = '---'
os.chdir(filepath)  # sets the directory to look in

#dictionaries of anticodons
scaffolds ={"TGGA" : "A", "CAAC" : "B", "TTAA" : "C", "ACAA" : "D",
    "TGAG" : "E", "TTCC" : "F", "TATA" : "G", "AAAT" : "H",
    "CTAC" : "I", "TCTA" : "J", "AAAC" : "K", "AAAA" : "L",
    "CAAA" : "M", "ACCT" : "N", "TCCT" : "O", "TTAC" : "P",
    "TAAT" : "Q", "TAAC" : "R", "AATC" : "S", "CTAT" : "T",
    "TGAT" : "U", "TTTT" : "V", "CTTT" : "W", "AATT" : "X",
    "TATC" : "Y", "AACC" : "Z", "TCAC" : "UU","CACA" : "VV",
    "CATT" : "WW","ACTT" : "XX","TATT" : "YY","TCTT" : "ZZ"}

codons_1 = {"AAAGCC" : "A", "AAGCCT" : "B", "TTTGGC" : "C", "GTTCCT" : "D",
    "CATACG" : "E", "CTCATG" : "F", "TGTCTC" : "G", "CTACAG" : "H",
    "CAGCTA" : "I", "CTGAGA" : "J", "AGCTCT" : "K", "TGTTCG" : "L",
    "AAGAGC" : "M", "AGCAGA" : "N", "GATCGA" : "O", "TCAGTC" : "P",
    "TACTGC" : "Q", "ATACGC" : "R", "GATTCC" : "S", "TGAAGC" : "T"}
codons_2 = {"TTCAGC" : "A", "ATCGAC" : "B", "GCAATC" : "C", "AAGTCC" : "D",
    "ATCCGT" : "E", "ACTCGA" : "F", "TCTTGC" : "G", "CACAAG" : "H",
    "TTAGCC" : "I", "AGTCCT" : "J", "GCATGA" : "K", "CAGACT" : "L",
    "TTCCAG" : "M", "GGCAAT" : "N", "TCGAGA" : "O", "CTAAGG" : "P",
    "AGGCTA" : "Q", "TCACTG" : "R", "TTGCTC" : "S", "AGCTTC" : "T"}
codons_3 = {"TCCGAT" : "A", "TGCACA" : "B", "GAGTCT" : "C", "CTGAAG" : "D",
    "TCGACT" : "E", "CGTCAT" : "F", "AGGTTG" : "G", "TACGGA" : "H",
    "GTAAGC" : "I", "CGTAGA" : "J", "TGACAC" : "K", "GTAGTG" : "L",
    "GTTCAG" : "M", "GACTAG" : "N", "AAACCG" : "O", "AATGGG" : "P",
    "AGAGAG" : "Q", "CGGTAA" : "R", "ACAGCA" : "S", "ACAAGG" : "T"}
```

```
# list of all codons, making a Counter object with all codons value 1 to compare enrichments
allcodons = []
for SKey in scaffolds.iterkeys():
  for key1 in codons_1.iterkeys():
    for key2 in codons_2.iterkeys():
      for key3 in codons_3.iterkeys():
        allcodons.append(codons_1[key1] + codons_2[key2] + codons_3[key3] +scaffolds[SKey])
allCodonCount = collections.Counter()
for codon in allcodons:
  allCodonCount[codon]+=1
writer = csv.writer(open('allcodons.csv', 'wb'))    # adapted from
http://stackoverflow.com/questions/8685809/python-writing-a-dictionary-to-a-csv-file-with-one-line-for-every-key-
value
for barcode, count in allCodonCount.iteritems():
  writer.writerow([barcode, count])

#open the file of the selection to be analyzed. extract out the sequences.
allFiles = glob.glob('*.fastq')               # makes a list of all the fastq files in that directory
#print allFiles
                        # https://docs.python.org/2/library/csv.html#module-csv
#with open(filepath + 'barcode keys.csv','rU') as csvfile:  # rU prevents the universal newline error
http://stackoverflow.com/questions/6726953/open-the-file-in-universal-newline-mode-using-csv-module-django
#  keyDict = {}
#  keys = csv.reader(csvfile, delimiter = ',')
#  for row in keys:
#    keyDict[row[0]] = row[1]              # makes a dictionary for the offsets

summarycsv = csv.writer(open('sequencing summary.csv', 'wb'))    # adapted from
http://stackoverflow.com/questions/8685809/python-writing-a-dictionary-to-a-csv-file-with-one-line-for-every-key-
value
summarycsv.writerow(['Selection', 'Total Reads', 'Valid Reads'])

for filename in allFiles:
  file = filepath + filename
  fasta = open(file)              # opens the fasta file
  fastaList = fasta.readlines()              # converts the file to a list http://stackoverflow.com/questions/328059/create-
a-list-that-contain-each-line-of-a-file
  seqList = fastaList[1::4]               # gets every 4th line, the sequences

  '''***CHANGE THIS IF FILENAME FORMAT HAS CHANGED***'''
  findIndices = re.search('(?<=_).*(?=_L)',filename)    # for MiSeq: get the selection indices, which are the first
strings between the underscores. adapted from http://stackoverflow.com/questions/15033905/regex-that-extracts-
text-between-tags-but-not-the-tags
#  findIndices = re.search('(?<=_).*(?=.R1.fastq)',filename)    # for NextSeq: get the selection indices, which are the
first strings between the underscores. adapted from http://stackoverflow.com/questions/15033905/regex-that-
extracts-text-between-tags-but-not-the-tags
  indices = findIndices.group(0)             # yields S# (whatever the sequencing index is) from the filename

  validseqs = []                   # list of valid sequences
  offset = ''
#  offset = keyDict[indices]               # is there an offset in the first 0-5 bases?
  for seq in seqList:
    if offset == seq[0:len(offset)]:          # if the first bases match the offset
      matchedSeq = re.search('GAGTGGGATG....TAG......ATCAT......AACTT......GTGTACAGGG', seq) #changed to
be 4Ns for scaffold
      # regex that finds the library sequence within the sequences nucleotides
      if matchedSeq is not None:           # if a matching sequence is found, add it
        template = matchedSeq.group(0)       # this should only find one result in matchedSeq.group, hence the 0
index
```

```
    #match sequences to codons
    if template[10:14] in scaffolds.keys():       #check if all 4 codons are valid
      if template[17:23] in codons_1.keys():
        if template[28:34] in codons_2.keys():
          if template[39:45] in codons_3.keys():
            scaff = scaffolds[template[10:14]] #all 4 are valid, so start mapping
            A = codons_1[template[17:23]]
            B = codons_2[template[28:34]]
            C = codons_3[template[39:45]]
            decoded = A + B + C + scaff
            validseqs.append(decoded +'\n')   #add this sequence to the list

  '''***CHANGE THIS IF FILENAME FORMAT HAS CHANGED***'''
  findProtein = re.search('.*(?=_S)', filename)    #find the protein name, similar logic as for the indices. same for
MiSeq and NextSeq
  protein = findProtein.group(0)

  #extractedFileName = indices + '_' + protein + '_decoded.txt'
  #testSeqsExtracted = open(extractedFileName, 'w')         #make a new file to write extracted sequences to
(https://docs.python.org/2/tutorial/inputoutput.html)
  #testSeqsExtracted.writelines(validseqs)
  print 'Selection ' + indices + ' (' + protein + ') has ' + str(len(validseqs)) + ' valid sequences out of ' +
str(len(seqList)) + ' reads.'
  summarycsv.writerow([protein, str(len(seqList)), str(len(validseqs))])

  #now looking through these decoded sequence files and counting them
  seqCounts = collections.Counter()
  for seq in validseqs:
    seqCounts[seq.rstrip()] +=1                  #rstrip() to get rid of the newline \n to make keys match
  #print 'done with counter'
  totCount = seqCounts + allCodonCount #add one to each so that we have all codons represented, and don't
divide by 0.
  for count in totCount.iteritems():
    #print type(count[1])
    #print len(validseqs)
    totCount[count[0]] = float(count[1])/float(len(validseqs)) #normalize to that selection

  #print len(seqCounts.keys())
  #print len(allCodonCount.keys())
  #print len(totCount.keys())
  counterFileName = indices + '_' + protein + '_seqCounts.csv'
  #export to csv
  writer = csv.writer(open(counterFileName, 'wb'))    # adapted from
http://stackoverflow.com/questions/8685809/python-writing-a-dictionary-to-a-csv-file-with-one-line-for-every-key-
value
#  writer.writerow(['Sequence', 'Scaffold', 'A Codon', 'B Codon', 'C Codon', 'Raw Count']) #header row
  for barcode, count in totCount.iteritems():
#    writer.writerow([barcode, barcode[0:2], barcode[2:5], barcode[5:8], barcode[8:11], count]) #barcodes, split by
individual codon, and total count
    writer.writerow([barcode, count, seqCounts[barcode]])
  #print 'done writing lines'



#dictionaries of REV codons
# scaffolds = {"4A":'TCCA', "4B":'GTTG', "4C":'TTAA', "4D":'TTGT', "4E":'CTCA',
#       "4F":'GGAA', "4G":'TATA', "4H":'ATTT', "4I":'GTAG', "4J":'TAGA',
#       "4K":'GTTT', "4L":'TTTT', "4M":'TTTG', "4N":'AGGT', "4O":'AGGA',
#       "4P":'GTAA', "4Q":'ATTA', "4R":'GTTA', "4S":'GATT', "4T":'ATAG',
```

```
#	"4U":'ATCA', "4V":'AAAA', "4W":'AAAG', "4X":'AATT', "4Y":'GATA',
#	"4Z":'GGTT',"4UU":'GTGA',"4VV":'TGTG',"4WW":'AATG',"4XX":'AAGT',
#	"4YY":'AATA',"4ZZ":'AAGA'}
# codons_1 = {"1A":'GGCTTT', "1B":'AGGCTT', "1C":'GCCAAA', "1D":'AGGAAC',
#	"1E":'CGTATG', "1F":'CATGAG', "1G":'GAGACA', "1H":'CTGTAG',
#	"1I":'TAGCTG', "1J":'TCTCAG', "1K":'AGAGCT', "1L":'CGAACA',
#	"1M":'GCTCTT', "1N":'TCTGCT', "1O":'TCGATC', "1P":'GACTGA',
#	"1Q":'GCAGTA', "1R":'GCGTAT', "1S":'GGAATC', "1T":'GCTTCA'}
# codons_2 =  {"2A":	'GCTGAA', "2B":	'GTCGAT', "2C":	'GATTGC', "2D":	'GGACTT',
#	"2E":	'ACGGAT', "2F":	'TCGAGT', "2G":	'GCAAGA', "2H":	'CTTGTG',
#	"2I":	'GGCTAA', "2J":	'AGGACT', "2K":	'TCATGC', "2L":'AGTCTG',
#	"2M":	'CTGGAA', "2N":	'ATTGCC', "2O":	'TCTCGA', "2P":	'CCTTAG',
#	"2Q":	'TAGCCT', "2R":	'CAGTGA', "2S":	'GAGCAA', "2T":	'GAAGCT'}
# codons_3 =  {"3A":	'ATCGGA', "3B":	'TGTGCA', "3C":	'AGACTC', "3D":	'CTTCAG',
#	"3E":	'AGTCGA', "3F":	'ATGACG', "3G":	'CAACCT', "3H":	'TCCGTA',
#	"3I":	'GCTTAC', "3J":'TCTACG', "3K":	'GTGTCA', "3L":	'CACTAC',
#	"3M":	'CTGAAC', "3N":	'CTAGTC', "3O":	'CGGTTT', "3P":	'CCCATT',
#	"3Q":	'CTCTCT', "3R":	'TTACCG', "3S":	'TGCTGT', "3T":	'CCTTGT'}
```

Calculating enrichment values of library members

```
this code will calculate enrichments. Put all of the *seqCounts.csv files from the other
code into a new folder.
Set the input lib and file path
updated 10/24/2016 for ULib notation (ABCXX)
updated regenerated library input 11/8/2017
'''
import glob
import csv
import os
import re

#first, find the sequence files
filepath = '----' #'insert filepath for selection sequencing reads (from sequencing script)
os.chdir(filepath)  # sets the directory to look in
inputLibFile = '---' #set the filepath for the input library

with open(filepath + inputLibFile,'rU') as csvfile:
  inputDict = {}
  keys = csv.reader(csvfile, delimiter = ',')
  for row in keys:
    inputDict[row[0]] = [row[1],row[2]]	# makes a dictionary for the input enrichments

'''change input text string if not input'''
allFiles = [i for i in glob.glob('*.csv') if 'seqCounts' in i and 'reg_lib' not in i]
# makes a list of all the seq Counts files in that directory except for the input lib
#print allFiles

for file in allFiles:
  with open(filepath + file,'rU') as csvfile:
    selectDict = {}
    keys = csv.reader(csvfile, delimiter = ',')
    for row in keys:
      selectDict[row[0]] = [row[1],row[2]]	# makes a dictionary for the {barcode: selection enrichments, seq
counts}

  enrichCalc = {}
  for key in selectDict.keys():
```

```
    enrichCalc[key] = float(selectDict[key][0])/float(inputDict[key][0])
  #print file
#  findIndices = re.search('.*?(?=_)',file)    # get the selection indices. .*? is non-greedy
#  indices = findIndices.group(0)  # yields A-1, or whatever the sequencing index is, from the filename

  findProtein = re.search('(?<=[0-9]_).*(?=_seqCounts)', file)    #find the protein name, similar logic as for the indices
  protein = findProtein.group(0)

  counterFileName = protein + '_enrichment_vs_input.csv'
  writer = csv.writer(open(counterFileName, 'wb'))    # adapted from
http://stackoverflow.com/questions/8685809/python-writing-a-dictionary-to-a-csv-file-with-one-line-for-every-key-
value
  writer.writerow(['Sequence', 'A Codon', 'B Codon', 'C Codon', 'Scaffold', 'Raw Selection Freq','Raw Selection
Count','Pre-enrich Freq', 'Pre-enrich Count', 'Enrichment']) #header row
  for barcode, enrich in enrichCalc.iteritems():
    writer.writerow([barcode, barcode[0], barcode[1], barcode[2], barcode[3:],
    selectDict[barcode][0], selectDict[barcode][1], inputDict[barcode][0], inputDict[barcode][1], enrich]) #barcodes,
split by individual codon, and total count
```

Supplementary Note 2

Synthetic Methods

Generalized procedures

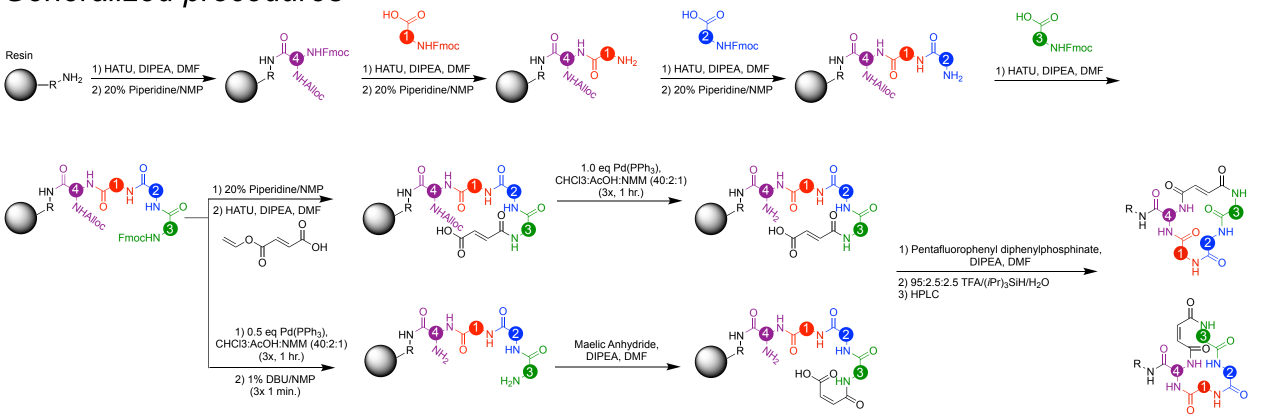

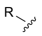

Resin	Tail Moiety
Rink Amide	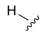
Bis-(2-aminoethyl)-ether trityl	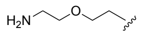
Universal PEG NovaTag™	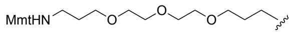
Universal NovaTag™	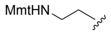

General procedure for solid-phase peptide synthesis of macrocycles inhibitors: Adapted from previous work[1], appropriate resin shown in above scheme (0.05-0.2 mmol) was swelled in ~10-15 mL *N,N'*-dimethylformamide (DMF) for 1 hour in a peptide synthesis vessel with mixing provided by N_2 bubbling. In a separate flask, *N*-allyloxycarbonyl-*N'*-Fmoc-amino acid (4th building block, 5 eq) and 1-[Bis(dimethylamino)methylene]-1*H*-1,2,3-triazolo[4,5-*b*]pyridinium 3-oxid hexafluorophosphate (HATU, 4.75 equiv.) was dissolved in ~10-15 mL DMF then treated with *N,N'*-diisopropylethylamine (DIPEA, 10 eq) for 5 minutes, observing a color change. This mixture was added to the pre-swollen resin and mixed at RT for 1-2 hours. The supernatant was then eluted from the reaction vessel leaving isolated resin. Resin was washed three times with ~5 mL DMF, then three times with ~5 mL N-methyl-2-pyrrolidone (NMP). Fmoc cleavage of resin bound amino acid was facilitated by suspending the resin with three 5-minute treatments of ~10 mL of 20% piperidine/NMP. After Fmoc cleavage, beads were washed three times with ~5 mL NMP and three times with ~5 mL DMF. The 1st, 2nd, and 3rd building blocks were then sequentially coupled onto resin bound 4th building block utilizing the same described protocol for the 4th building block. Macrocycles with Fmoc-2-aminomethyl-phenylacetic acid as a building block were instead treated three separate times with 2 eq/1.9 eq of this building block/HATU for 8 hours each. Upon coupling of the final 3rd building block, terminal Fmoc group was left on the peptide. 3rd building blocks with piperidine like structures were allowed to be coupled onto resin for 16 hours.

For cis-alkene isomer, resin was washed three times with $CHCl_3$ then suspended in degassed $CHCl_3$, acetic acid, and N-methylmorpholine (40:2:1 ratio). Allyloxy deprotection was then afforded by treating resin three times at RT for 1 hour each with tetrakis(triphenylphosphine)palladium(0) (0.5 eq total). Resin was then washed with ~20 mL 5% DIPEA/DMF, ~20 mL 5% (w/v) sodium diethyldithiocarbamate trihydrate in DMF, ~20 mL of 5% (w/v) hydroxybenzotriazole monohydrate in DMF, then finally with ~20 mL 1:1 CH_2Cl_2/DMF. In a separate flask, maleic anhydride (10 eq) was dissolved in DMF and treated with DIPEA (20 eq), turning a dark red/black color. Mixture was then added to resin and allowed to mix at RT for 1 hour. Resin was then washed five times with DMF to remove as much maleic anhydride as possible. Fmoc deprotection was then afforded by mixing resin with ~10 mL of 1% 1,8-Diazabicyclo[5.4.0]undec-7-ene (DBU) in DMF, with three, 1 minute treatments. Resin was then washed three times with 20% DIPEA/DMF.

For trans-alkene isomer, the terminal, 3rd building block Fmoc was cleaved using previously described deconditions with 20% piperidine/NMP. In a separate flask allyl-fumarate monoester[1] (10 eq) was dissolved in DMF with HATU (9.5 eq) and treated with DIPEA (20 eq), observing a change of color to dark red/black. Mixture was then added to resin and allowed to mix at RT for 1 hour. Resin was then washed three times with DMF and three times with $CHCl_3$. Resin was then suspended in degassed $CHCl_3$, acetic acid, and N-methylmorpholine (40:2:1 ratio). Concurrent allyloxy and allyl deprotection was then afforded by treating resin three times at RT for 1 hour each with tetrakis(triphenylphosphine)palladium(0) (1.0 eq total). Resin was then washed with ~20 mL 5% DIPEA/DMF, ~20 mL 5% (w/v) sodium diethyldithiocarbamate trihydrate in DMF, ~20 mL of 5% (w/v) hydroxybenzotriazole monohydrate in DMF, then finally with ~20 mL CH_2Cl_2/DMF.

For both cis and trans isomer, final macrocyclization was afforded by treating resin with pentafluorophenyl diphenylphosphinate (5 eq), anhydrous DIPEA (10 eq) dissolved in ~10 mL anhydrous DMF for 3-16 hours. Resin was then washed three times with DMF, three times with CH_2Cl_2 and allowed to dry in the fume hood. Macrocycle product was then cleaved off resin with two treatments of ~15 mL solution containing 95% trifluoroacetic acid (TFA), 2.5% triisopropylsilane (TIPS) and 2.5% water. Macrocycles sensitive to hydrosilane reduction were cleaved without TIPS. Resin was then washed two times with TFA. Combined supernatants were then dried to an oily residue through rotary evaporation. The peptide was the precipitated out of solution with Et_2O cooled to -78°C. Ether was then decanted and isolated precipitate was dissolved in a minimal amount of 3:1 DMF/water, and filtered through 0.22 μL Ultrafree-MC Centrifugal Filters (Millipore Sigma). For hydrophobic macrocycles, no ether precipitation was conducted and sample was directly dissolved in 3:1 DMF/water. Sample was then purified by reverse phase HPLC, with a gradient of 10-60% acetonitrile/water containing 0.1% TFA over 40 minutes. Fractions that contained product was then freeze dried to produce a white powder. Yields for reported macrocycles varied from 1-10% of the initial resin loading.

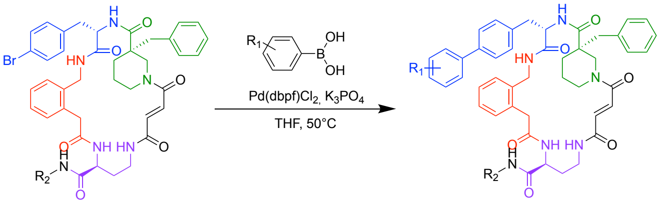

Generalized procedure A for Suzuki-Miyaura cross-coupling of macrocycles: Adapting conditions described previously[2], into a 10 mL screw-top vial, bromo-macrocycle (0.011 mmol, 1.0 eq), boronic acid (0.11 mmol, 10 eq), [1,1'Bis(di-*tert*-butylphosphino)ferrocene]dichloropalladium (II) (0.0002 mmol, 0.2 eq), and potassium phosphate tribasic (0.23 mmol, 20 eq) was combined and sealed with a septum. Flask was evacuated and refilled with N_2 three times and left under N_2. Degassed THF (0.05 M), by cycling between vacuum and N_2 with concurrent sonication, was then added to the sealed flask and allowed to stir at 50° C for 16 hours. After cooling, the reaction was cooled and solvent was removed under high-vac. Solid residue was dissolved in a minimal amount of 3:1 DMF/water with 5% TFA and filtered through 0.22 μL Ultrafree®-MC Centrifugal Filters (Milipore-Sigma). Sample was then purified by reverse HPLC, with a 10-60% or 10-100% gradient of acetonitrile/water containing 0.1%TFA depending on hydrophobicity of compound. Fractions that contained product was then freeze dried to produce a white powder.

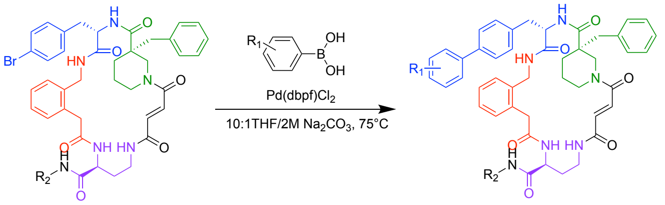

Generalized procedure B for Suzuki-Miyaura cross-coupling of macrocycles: Into a 10 mL screw-top vial, bromo-macrocycle (0.011 mmol, 1.0 eq), boronic acid (0.11 mmol, 10 eq), [1,1'Bis(di-*tert*-butylphosphino)ferrocene]dichloropalladium (II) (0.0002 mmol, 0.2 eq), were combined and sealed with a septum. Flask was evacuated and refilled with N_2 three times and left under N_2. Degassed 10:1 THF/2M Na_2CO_3 (0.05 M) was added and reaction as heated to at 75° C for 16 hours. After cooling, the reaction was cooled and solvent was removed under high-vac. Solid residue was dissolved in a minimal amount of 3:1 DMF/water with 5% TFA and filtered through 0.22 μL Ultrafree®-MC Centrifugal Filters (Milipore-Sigma). Sample was then purified by reverse HPLC, with a 10-60% or 10-100% gradient of acetonitrile/water containing 0.1%TFA depending on hydrophobicity of compound. Fractions that contained product was then freeze dried to produce a white powder.

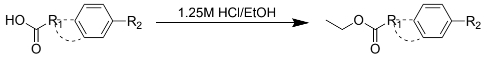

General conditions for ethyl ester protection of carboxylic acids: In a 50 mL roundbottom flask, ester (1 mmol, 1 eq) was suspended in 1.25M HCl/EtOH at RT for 16 hours. Reaction was then diluted slowly with 100 mL saturated $NaHCO_3$, and aqueous layer was extracted three times with EtOAc. Combined organic layers were washed with saturated NaCl, dried over Na_2SO_4, and concentrated by rotary evaporation to afford ester product.

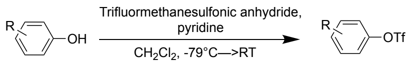

Generalized conditions for triflation of phenols: Into a N_2 flushed 100 mL roundbottom flask, phenol derivative (1.0 mmol, 1.0 eq), was dissolved in CH_2Cl_2 and cooled to -78°C in a dry ice/acetone bath. After sequential dropwise addition of pyridine (3.0 mmol, 3.0 eq) and trifluoromethanesulfonic anhydride (1.1mmol, 1.1 eq), reaction was allowed to stir for 16 hours, warming up to RT. Reaction was then diluted with 100 mL saturated $NaHCO_3$, and aqueous layer was extracted three times with EtOAc. Combined organic layers were washed with saturated NaCl, dried over Na_2SO_4, and concentrated by rotary evaporation to afford triflate product.

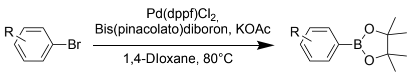

Generalized Miyaura borylation conditions: Into a 100 mL roundbottom flask, aryl bromide (1.0 mmol, 1.0 eq), bis(pinacolato)diboron (2.0 mmol, 2.eq), [1,1'Bis(diphenylphosphino)ferrocene]dichloropalladium (II) (0.1 mmol, 0.1 eq), and potassium acetate (3.0 mmol, 3 eq) was combined and sealed with a septum. Flask was evacuated and refilled with N_2 three times and left under N_2. Degassed 1,4-Dioxane (0.1 M), by cycling between vacuum and N_2 with concurrent sonication, was then added to the sealed flask and allowed to stir at 80° C for 16 hours. After cooling, the reaction was cooled and diluted with 1M HCl and transferred to a separatory funnel. Aqueous layer was extracted three times with EtOAc and combined organic layers were washed with saturated NaCl, dried over Na_2SO_4, and concentrated by rotary evaporation. Residue was purified by column chromatography to afford product.

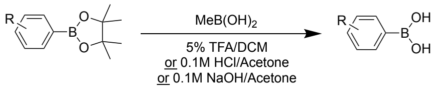

General pinacol deprotection conditions: Adapting conditions described previously[3], In a 50 mL roundbottom flask, pinacol boronic ester (1 mmol, 1 eq) and methylboronic acid (10 mmol, 10 eq) were suspended in 5% TFA/CH_2Cl_2, 0.1M HCl/Acetone, or 0.1M NaOH/Acetone depending on acid sensitivity of substrate and allowed to stir at RT for 16 hours. Solvent was removed by rotary evaporation and the residue was resuspended in 0.1M HCl. HCl was removed by rotary evaporation, removing excess methylboronic acid and methylboronic acid pinacol ester. This cycle was repeated three times until no methylboronic acid was present by NMR analysis. For acid sensitive substrates, residue was resuspended in water during rotary evaporation steps. For substrates dissolved in 0.1M NaOH/Acetone, solution was neutralized to ~pH 3, filtered, and concentrated by rotary evaporation. Excess $MeB(OH)_2$ was removed as described above using water.

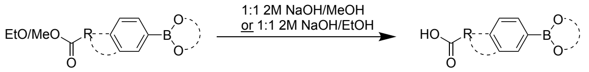

General conditions for saponification of methyl/ethyl esters: In a 50 mL roundbottom flask, ester (1 mmol, 1 eq) was suspended in 1:1 2M NaOH/MeOH (0.1M) for methyl esters or 1:1 2M NaOH/EtOH (0.1M) for ethyl esters and stirred at RT for 15 hours. Reaction was neutralized by diluting reaction with 50 mL 1M HCl, and aqueous layer was extracted three times with EtOAc. Combined organic layers were washed with saturated NaCl, dried over Na_2SO_4, and concentrated by rotary evaporation to isolated carboxylic acid.

Synthesis of intermediates

^{1}H-NMR spectra are provided in a separate part of the Supplementary Information. HRMS characterization for intermediates are shown in Supplementary Table 7.

4F

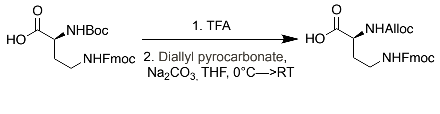

4F (**128**): In a 500 mL roundbottom flask, N^{α}-Boc-N^{γ}-Fmoc-L-2,4-diaminobutyric acid (10 mmol) was dissolved in 20 mL TFA and stirred at RT for 1 hour. TFA was then removed by rotary evaporation, leaving an oily solution. Oil was dissolved in 1:1 tetrahydrofuran (THF)/water (0.05 M), sodium carbonate (30 mmol, 3 eq) was added to the flask, and cooled to 0°C. Diallyl pyrocarbonate (15 mmol, 1.5 eq) was then added dropwise to the flask and the reaction was capped and allowed to stir for 5 hours, warming up to RT. THF was removed by rotary evaporation and the aqueous layer was transferred to a separatory funnel and washed two times with 100 mL Et_2O. Aqueous layer was then acidified to pH 1 with 12N HCl, resulting in white precipitate. Aqueous layer was then extracted three times with 100 mL of ethyl acetate (EtOAc). Combined organic layers were washed with saturated NaCl, dried over Na_2SO_4, and concentrated by rotary evaporation to afford **4F** a yellow foam. Yield: 82%

I1

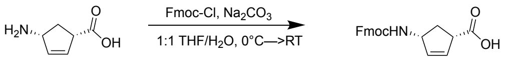

I1 (129): In a 250 mL roundbottom flask, (-)-(1*S*,4*R*)-4-Aminocyclopent-2-enecarboxylic acid (8 mmol), 9-Fluorenylmethoxycarbonyl chloride (8.8 mmol, 1.1 eq), and sodium carbonate (40 mmol, 6 eq) were dissolved in 1:1 THF/water (0.1 M) and stirred at RT for 16 hour. THF was removed by rotary evaporation and ether was added to the aqueous suspension. Aqueous layer was washed two times with ether, then acidified to pH2 with 12N HCl. Aqueous layer was then extracted three times with 100 mL of ethyl acetate (EtOAc). Combined organic layers were washed with saturated NaCl, dried over Na_2SO_4, and concentrated by rotary evaporation to afford **I1** as a white solid. Yield: 96%

I2

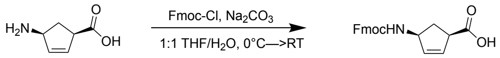

I2 (130): Compound was synthesized as described for compound **I1**. Synthesis afforded **I2** as a white solid. Yield: 89%

I3

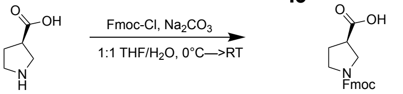

I3 (131): In a 250 mL roundbottom flask, (R)-(−)-Pyrrolidine-3-carboxylic acid (2 mmol), 9-Fluorenylmethoxycarbonyl chloride (2.2 mmol, 1.1 eq), and sodium carbonate (12 mmol, 6 eq) were dissolved in 1:1 THF/water (0.1 M) and stirred at RT for 16 hour. THF was removed by rotary evaporation and ether was added to the aqueous suspension. Aqueous layer was washed two times with ether, then acidified to pH2 with 12N HCl. Aqueous layer was then extracted three times with 100 mL of ethyl acetate (EtOAc). Combined organic layers were washed with saturated NaCl, dried over Na_2SO_4, and concentrated by rotary evaporation to afford **I3** as a white solid. Yield: 78%

I4

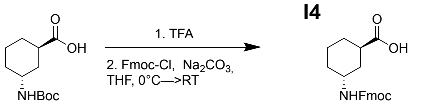

I4 (132): In a 250 mL roundbottom flask, (1R,3R)-3-([(tert-Butoxy)carbonyl]amino)cyclohexane-1-carboxylic acid (1 mmol) was dissolved in 20 mL TFA and stirred at RT for 1 hour. TFA was then removed by rotary evaporation, leaving an oily solution. Residue was dissolved in 1:1 THF/water (0.1M) at 0°C, followed by addition of 9-Fluorenylmethoxycarbonyl chloride (1.1 mmol, 1.1 eq), and sodium carbonate (6 mmol, 6 eq). The reaction was stirred 16 hours, warming to RT. THF was removed by rotary evaporation and ether was added to the aqueous suspension. Aqueous layer was washed two times with ether, then acidified to pH2 with 12N HCl. Aqueous layer was then extracted three times with 100 mL of ethyl acetate (EtOAc). Combined organic layers were washed with saturated NaCl, dried over Na_2SO_4, and concentrated by rotary evaporation to afford **I4** as a yellow solid with some impurities. Compound was carried onto next step without further purification. Yield: 45%

I5

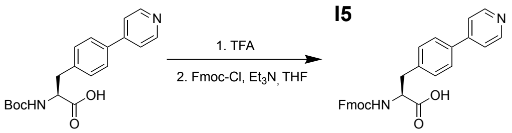

I5 (133): In a 250 mL roundbottom flask, N-Boc-4-(4-pyridinyl)-L-phenylalanine (0.4 mmol) was dissolved in 20 mL TFA and stirred at RT for 1 hour. TFA was then removed by rotary evaporation, leaving an oily solution. Residue was suspended in THF (0.1M), and water was added until all material dissolved. 9-Fluorenylmethoxycarbonyl chloride (0.44 mmol, 1.1 eq) dissolved in THF was then added, followed by triethylamine (2.0 mmol, 5 eq) and the reaction was stirred 16 hour at RT. THF was removed by rotary evaporation and reaction as partitioned between

EtOAc and 1M HCl. Aqueous layer was then extracted three times with 100 mL of ethyl acetate (EtOAc). Combined organic layers were washed with saturated NaCl, dried over Na_2SO_4, and concentrated by rotary evaporation. Crude product was purified by column chromatography (0-20% MeOH/CH_2Cl_2) to afford **I5** as a white solid. Yield: 88%

I6a

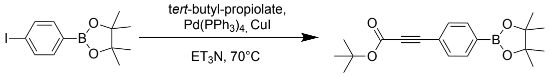

I6a (134): Adapting a previously reported protocol[4], in a N_2 flushed 100mL roundbottom flask, a solution of 4-Iodophenylboronic acid pinacol ester, was dissolved in degassed triethylamine. Then, Tetrakis(triphenylphosphine)-palladium(0), Copper(I) iodide and *tert*-butyl propiolate were added successively and the reaction was heated at 70°C overnight. Reaction was then concentrated by roatry evaporation and crude material was directly purified by column chromatography (0-50% EtOAc/Hex). Product **I6a** was isolated as a yellow solid. Yield: 28%

I6b

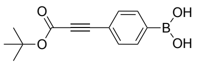

I6b (135): Compound was synthesized using **general pinacol deprotection conditions** with **I6a** as starting material, substituting 0.1N HCl/Acetone as solvent. Solvent was removed by rotary evaporation and the residue was resuspended in H_2O and once again removed by rotary evaporation to remove excess $MeB(OH)_2$. This step was repeated until all $MeB(OH)_2$ was removed as seen on ^1H NMR. Product **I6b** was isolated as a white solid with some minor impurities. Product was carried onto next step without further purification. Yield: quantitative

I7a

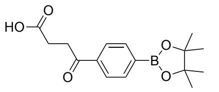

I7a (136): Compound was synthesized using **generalized Miyaura borylation conditions** with 3-(4-Bromobenzoyl)propionic acid. Column chromatography conditions: 0-20% MeOH/CH_2Cl_2. Product **I7a** was isolated as a yellow solid with some excess bis(pinacolato)diboron and extra impurities. Product was carried onto next step without further purification. Yield: approx. quantitative.

I7b

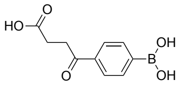

I7b (137): Compound was synthesized using **general pinacol deprotection conditions** with **I7a** and 5% TFA/CH_2Cl_2 as solvent. Product **I7b** was isolated as a yellow solid with some minor impurities. Product was carried onto next step without further purification. Yield: 88%

I8a

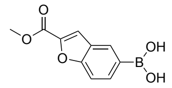

I8a (138): Compound was synthesized using **general pinacol deprotection conditions** with Methyl 5-(tetramethyl-1,3,2-dioxaborolan-2-yl)-1-benzofuran-2-carboxylate and 5% TFA/CH_2Cl_2 as solvent. Product **I8a** was isolated as a white solid. Yield: 98%

I8b

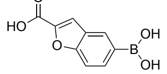

I8b (139): Compound was synthesized using **general conditions for saponification of methyl/ethyl esters** with **I8a**, using 1:1 2M NaOH/MeOH as solvent. Product **I8b** was isolated as white solid with some small impurities. Product was carried onto next step without further purification. Yield: quantitative

I9a

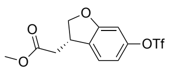

I9a (140): Compound was synthesized using **generalized conditions for triflation of phenols** with (S)-Methyl 2-(6-hydroxy-2,3-dihydrobenzofuran-3-yl)acetate. Product **I9a** was isolated as a brown oil. Yield: 78%

I9b

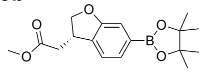

I9b (141): Compound was synthesized using **generalized Miyaura borylation conditions** with **I9a** and no acidic workup. Crude reaction was directly purified by column chromatography (0-50%EtOAc/Hex) to afford product **I9b** as white powder with small amounts of bis(pinacolato)diboron. Product was carried onto next step without further purification. Yield: 67%

I9c

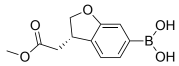

I9c (142): Compound was synthesized using **general pinacol deprotection conditions** with **I9b** and 5% TFA/CH_2Cl_2 as solvent. Product **I9c** was isolated as a white solid. Yield: 90%

I9d

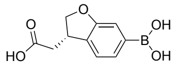

I9d (143): Compound was synthesized using **general conditions for saponification of methyl/ethyl esters** with **I9c**,using 1:1 2M NaOH/MeOH as solvent. Product **I9d** was isolated as white solid with small impurities. Product was carried onto next step without further purification. Yield: 90%

I10

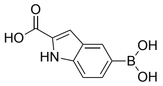

I10 (144): Compound was synthesized using **general pinacol deprotection conditions** with 5-(Tetramethyl-1,3,2-dioxaborolan-2-yl)-1H-indole-2-carboxylic acid and 5% TFA/CH_2Cl_2 as solvent. Product **I10** was isolated as a white solid. Yield: quantitative

I11

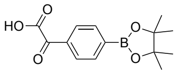

I11 (145): Compound was synthesized using **general conditions for saponification of methyl/ethyl esters** with methyl 2-oxo-2-[4-(tetramethyl-1,3,2-dioxaborolan-2-yl)phenyl]acetate, using 1:1 2M NaOH/MeOH as solvent. Product **I11** was isolated as white solid with small impurities. Product was carried onto next step without further purification. Yield: quantitative

I12a

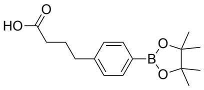

I12a (146): Compound was synthesized using **generalized Miyaura borylation conditions** with 4-(4-Bromophenyl)butanoic acid. Column chromatography conditions: 0-20% MeOH/CH_2Cl_2. Product **I12a** was isolated as a yellow solid with some excess bis(pinacolato)diboron and extra impurities. Product was carried onto next step without further purification Yield: approx. 37%

I12b

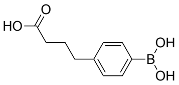

I12b (147): Compound was synthesized using **general pinacol deprotection conditions** with **I12a** and 5% TFA/CH_2Cl_2 as solvent. Product **I12b** was isolated as a yellow solid. Yield: quantitative

I13a

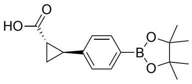

I13a (148): Compound was synthesized using **generalized Miyaura borylation conditions** with (1S,2S)-2-(4-Bromophenyl)cyclopropanecarboxylic acid. Column chromatography conditions: 0-20% MeOH/CH_2Cl_2. Product **I13a** was isolated as a yellow solid with some excess bis(pinacolato)diboron and small impurities. Product was carried onto next step without further purification Yield: approx. quantitative

I13b

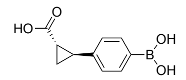

I13b (149): Compound was synthesized using **general pinacol deprotection conditions** with **I13a** and 5% TFA/CH_2Cl_2 as solvent. Product **I13b** was isolated as a yellow solid with minor impurities and trace amounts of **I13a**. Product was carried on without further purification. Yield: 86%

I14a

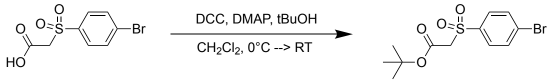

I14a (150): Adapting from a previously reported protocol[5], into a 50mL roundbottom flask, 2-(4-bromobenzenesulfonyl)acetic acid (0.7 mmol, 1 eq), 4-Dimethylaminopyridine (0.11 mmol, 0.16eq), and *tert*-butanol (2.8 mmol, 4 eq) were dissolved in CH_2Cl_2 (0.1 M) and cooled in an ice bath. N, N′-Dicyclohexylcarbodiimide dissolved in 2 mL CH_2Cl_2 was then added and stirred for 15 minutes, observing a white precipitate. Reaction was then warmed to RT and allowed to stir for 16 hours. Precipitate was filtered through celite and reaction concentrated by rotary evaporation. Residue was dissolved in 100mL EtOAc and 100 mL saturated $NaHCO_3$. Organic layer was isolated and aqueous layer was extracted two more times with 100 mL EtOAc. Combined organic layers were washed with saturated NaCl, dried over Na_2SO_4, and concentrated by rotary evaporation. Product **I14a** was isolated as a white solid. Yield: 85%

I14b

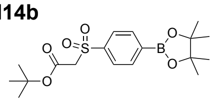

I14b (151): Compound was synthesized using **generalized Miyaura borylation conditions** with **I14a** and no acidic workup. Crude reaction was directly purified by column chromatography (0-50%EtOAc/Hex) to afford product **I14b** as white powder with trace amounts of bis(pinacolato)diboron. Product was carried onto next step without further purification. Yield: 88%.

I14c

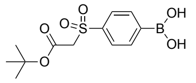

I14c (152): Compound was synthesized using **general pinacol deprotection conditions** with **I14b** and 0.1M NaOH/Acetone as solvent. Product **I14c** was isolated as a white solid with methylboronic acid and other impurities identified on H-NMR. Purification by column chromatography was attempted on crude **I14c** product, but full oxidation of the boronic acid to a phenol was observed. Therefore, crude **I14c** product was carried onto further reactions without further purification. Yield: approx. quantitative

I15a

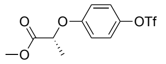

I15a (153): Compound was synthesized using **generalized conditions for triflation of phenols** with Methyl (r)-(+)-2-(4-hydroxyphenoxy)propanoate. Product **I15a** was isolated as a yellow oil. Yield: quantitative

I15b

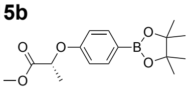

I15b (154): Compound was synthesized using **generalized Miyaura borylation conditions** with **I15a** and no acidic workup. Crude reaction was directly purified by column chromatography (0-50%EtOAc/Hex) to afford product **I15b** as white powder with trace amounts of bis(pinacolato)diboron. Product was carried onto next step without further purification Yield: 67%

I15c

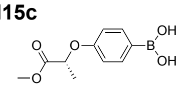

I15c (155): Compound was synthesized using **general pinacol deprotection conditions** with **I15b** and 5% TFA/CH_2Cl_2 as solvent. Product **I15c** was isolated as a white solid with trace amounts of **I15b** excess $MeB(OH)_2$. Product was carried onto next step without further purification. Yield: quantitative

I15d

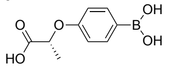

I15d (156): Compound was synthesized using **general conditions for saponification of methyl/ethyl esters** with **I15c**, using 1:1 2M NaOH/MeOH as solvent. Product **I15d** was isolated as brown solid with trace amounts of saponified **I15b**. Product was carried on without further purification. Yield: 61%

I16a

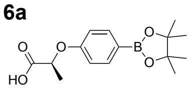

I16a (157): Compound was synthesized using **generalized Miyaura borylation conditions** with (2S)-2-(4-bromophenoxy)propanoic acid. Column chromatography conditions: 0-20% MeOH/CH_2Cl_2. Product **I16a** was isolated as a yellow solid with some excess bis(pinacolato)diboron and extra impurities. Product was carried onto next step without further purification Yield: approx. 89%

I16b

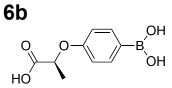

I16b (158): Compound was synthesized using **general pinacol deprotection conditions** with **I16a** and 5% TFA/CH_2Cl_2 as solvent. Product **I16b** was isolated as a white solid with some impurities and excess $MeB(OH)_2$. Product was carried on without further purification. Yield: quantitative

I17a

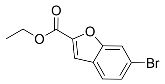

I17a (159): Compound was synthesized using **general conditions for ethyl ester protection of carboxylic acids** using 6-Bromobenzofuran-2-carboxylic acid. Product **I17a** was isolated as a white solid with a small amount of starting material impurities. Product was carried onto next step without further purification. Yield: quantitative

I17b

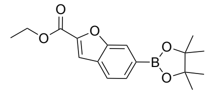

I17b (160): Compound was synthesized using **generalized Miyaura borylation conditions** with **I17a** and no acidic workup. Crude reaction was directly purified by column chromatography (0-50%EtOAc/Hex) to afford product **I17b** as clear solid with small amounts of bis(pinacolato)diboron. Product was carried onto next step without further purification Yield: 88%

I17c

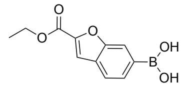

I17c (161): Compound was synthesized using **general pinacol deprotection conditions** with **I17b** and 5% TFA/CH_2Cl_2 as solvent. Product **I17c** was isolated as a white solid. Yield: 83%

I17d

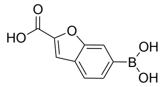

I17d (162): Compound was synthesized using **general conditions for saponification of methyl/ethyl esters** with **I17c**,using 1:1 2M NaOH/EtOH as solvent. Product **I17d** was isolated as brown solid. Yield: quantitative

I18a

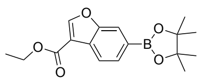

I18a (163): Compound was synthesized using **generalized Miyaura borylation conditions** with Ethyl 6-bromobenzofuran-3-carboxylate and no acidic workup. Crude reaction was directly purified by column chromatography (0-50%EtOAc/Hex) to afford product **I18a** as white solid with trace amounts of bis(pinacolato)diboron. Product was carried onto next step without further purification Yield: 94%

I18b

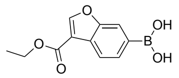

I18b (164): Compound was synthesized using **general pinacol deprotection conditions** with **I18a** and 5% TFA/CH_2Cl_2 as solvent. Product **I18b** was isolated as a white solid. Yield: quantitative

I18c

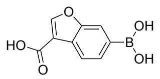

I18c (165): Compound was synthesized using **general conditions for saponification of methyl/ethyl esters** with **I18b**, using 1:1 2M NaOH/EtOH as solvent. Product **I18c** was isolated as white solid. Yield: 98%

I19a

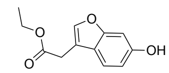

I19a (166): Compound was synthesized using **general conditions for ethyl ester protection of carboxylic acids** using 2-(6-Hydroxybenzofuran-3-yl)acetic acid. Product **I19a** was isolated as a brown oil. Yield: 86%

I19b

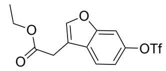

I19b (167): Compound was synthesized using **generalized conditions for triflation of phenols** with **I19a**. Product **I9b** was isolated as a brown oil. Yield: quantitative

I19c

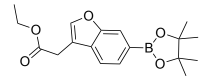

I19c (168): Compound was synthesized using **generalized Miyaura borylation conditions** with **I19b** and no acidic workup. Crude reaction was directly purified by column chromatography (0-50%EtOAc/Hex) to afford product **I19c** as white solid with trace amounts of bis(pinacolato)diboron. Product was carried onto next step without further purification Yield: 76%

I19d

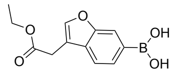

I19d (169): Compound was synthesized using **general pinacol deprotection conditions** with **I19c** and 5% TFA/CH_2Cl_2 as solvent. Product **I19d** was isolated as a white solid. Yield: 82%

I19e

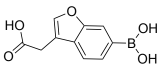

I19e (170): Compound was synthesized using **general conditions for saponification of methyl/ethyl esters** with **I19d**, using 1:1 2M NaOH/EtOH as solvent. Product **I19e** was isolated as white solid. Yield: 96%

I20a

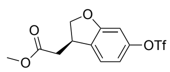

I20a (171): Compound was synthesized using **generalized conditions for triflation of phenols** with (R)-Methyl 2-(6-hydroxy-2,3-dihydrobenzofuran-3-yl)acetate. Product **I20a** was isolated as a clear oil. Yield: quantitative

I20b

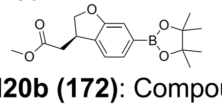

I20b (172): Compound was synthesized using **generalized Miyaura borylation conditions** with **I20a** and no acidic workup. Crude reaction was directly purified by column chromatography (0-50%EtOAc/Hex) to afford product **I20b**

as white powder with small amounts of bis(pinacolato)diboron. Product was carried onto next step without further purification Yield: 81%

I20c

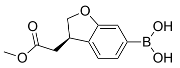

I20c (173): Compound was synthesized using **general pinacol deprotection conditions** with **I20b** and 5% TFA/CH_2Cl_2 as solvent. Product **I20c** was isolated as a white solid. Yield: quantitative

I20d

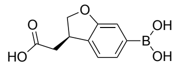

I20d (174): Compound was synthesized using **general conditions for saponification of methyl/ethyl esters** with **I20c**, using 1:1 2M NaOH/MeOH as solvent. Product **I20d** was isolated as white solid with minor impurities. Product was carried on without further purification. Yield: 42%

I21a

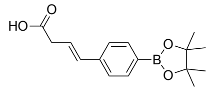

I21a (175): Compound was synthesized using **generalized Miyaura borylation conditions** with (3E)-4-(4-bromophenyl)but-3-enoic acid. Column chromatography conditions: 0-20% MeOH/CH_2Cl_2. Product **I21a** was isolated as a yellow solid with some excess bis(pinacolato)diboron and extra impurities. Product was carried onto next step without further purification Yield: approx. 93%

I21b

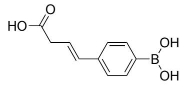

I21b (176): Compound was synthesized using **general pinacol deprotection conditions** with **I21a** and 5% TFA/CH_2Cl_2 as solvent. Product **I21b** was isolated as a yellow solid. Yield: 86%

I22a

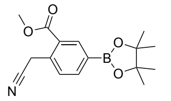

I22a (177): Compound was synthesized using **generalized Miyaura borylation conditions** with Methyl 5-bromo-2-(cyanomethyl)benzoate and no acidic workup. Crude reaction was directly purified by column chromatography (0-50%EtOAc/Hex) to afford product **I22a** as white powder with trace amounts of bis(pinacolato)diboron. Product was carried onto next step without further purification Yield: 97%

I22b

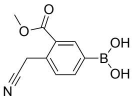

I22b (178): Compound was synthesized using **general pinacol deprotection conditions** with **I22a** and 5% TFA/CH_2Cl_2 as solvent. Product **I22b** was isolated as a white solid. Yield: quantitative 80%

I22c

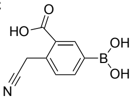

I22c (179): Compound was synthesized using **general conditions for saponification of methyl/ethyl esters** with **I22b**, using 1:1 2M NaOH/MeOH as solvent. Product **I20d** was isolated as white solid with some minor impurities. Product was carried on without further purification. Yield: quantitative

I23

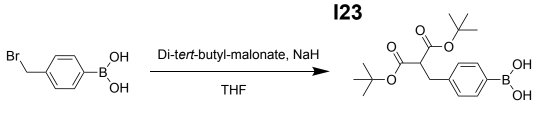

I23 (180): Adapting a previously reported protocol[6], Into a N_2 flushed 50 mL roundbottom flask, sodium hydride (60% dispersion in mineral oil) (1.47 mmol, 1.05 eq) was dissolved in anhydrous THF (0.1 M) followed by dropwise addition of di-*tert*-butyl-malonate. Once gas finished forming from the solution, 4-(Bromomethyl)phenylboronic acid dissolved in 1mL THF was added to the flask, which was allowed to stir at room temperature for 16 hours. The reaction was then diluted with saturated NH_4Cl and extracted three times with EtOAc. Combined organic layers were washed with saturated NaCl, dried over Na_2SO_4, and concentrated by rotary evaporation. Residue was purified by silica gel chromatography (0-10% MeOH/CH_2Cl_2) to afford product as white solid. Yield: 84%

I24a

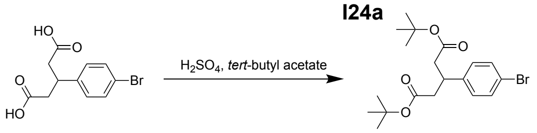

I24a (181): Adopting a previously reported protocol[7], into a N_2 flushed 50 mL roundbottom flask, B-(4-Bromophenyl)glutaric acid (0.7 mmol, 1.0 eq) was suspended in *tert*-butyl-acetate (0.2M), followed by addition of concentrated H_2SO_4 (0.7 mmol, 1 eq). Reaction was allowed to stir at room temperature for 16 hours. Reaction was diluted with sat. bicarbonate and extracted three times with EtOAc. Combined organic layers were washed with saturated NaCl, dried over Na_2SO_4, and concentrated by rotary evaporation. Residue was purified by column chromatography (0-50% EtOAc/Hex) to afford product as white solid. Yield: 47%

I24b

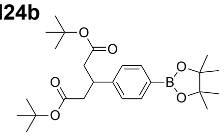

I24b (182): Compound was synthesized using **generalized Miyaura borylation conditions procedure** with **I24a** and no acidic workup. Crude reaction was directly purified by column chromatography (0-50%EtOAc/Hex) to afford product as white powder with trace amounts of bis(pinacolato)diboron. Yield: 97%

I24c

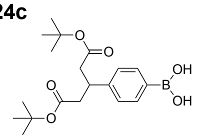

I24c (183): Compound was synthesized **using general pinacol deprotection conditions** with **I24b** and substituting 0.1N NaOH/Acetone as solvent. After 16 hours, reaction was neutralized to ~pH 2-3 with 1N HCl and filtered to remove salts. Solvent was removed by rotary evaporation and the residue was resuspended in H_2O and once again removed by rotary evaporation to remove excess $MeB(OH)_2$. This step was repeated until all $MeB(OH)_2$ was removed as seen on ^1H NMR. Product was isolated as a white solid with small impurities. Product was carried on to next step without further purification Yield: quantitative

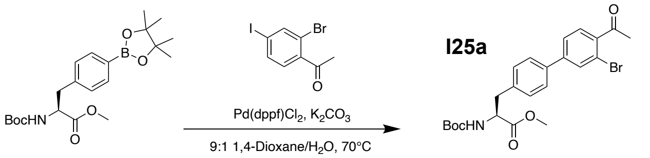

I25a (184): Adapting a previously reported protocol[8], a mixture of Boc-4-borono pinacol ester-L phenylalanine methyl ester (1.98 mmol, 1 eq.), 1-(2-Bromo-4-iodophenyl)ethanone (2.56 mmol, 1.3 eq.), [1,1-Bis(diphenylphosphino)ferrocene]dichloropalladium(II) (0.19 mmol, 0.1 eq.), and potassium carbonate (7.90 mmol, 4 eq.) was dissolved in 18 mL of degassed 1,4-dioxane and 2 mL of degassed water (0.1 M) and stirred at 70°C in a sealed round bottom flask overnight. The reaction was then cooled down to room temperature, diluted with water, extracted three times with ethyl acetate (EtOAc), washed with brine, dried over sodium sulfate (Na_2SO_4), and evaporated under reduced pressure. The crude product was then dry loaded onto a 25G SNAP column, purified by flash column chromatography using 0-50% EtOAc/Hexanes, and yielded **I25a** as a yellow foam. Yield: 42% yield

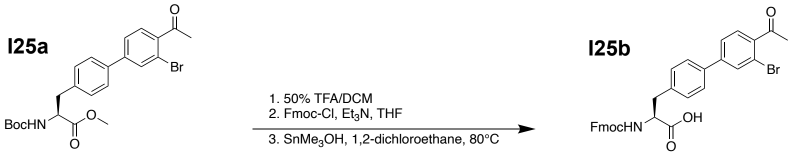

I25b (185): Compound **I25a** (0.84 mmol, 1 eq.) was dissolved in 4.5 mL trifluoroacetic acid and 4.5 mL dichloromethane (0.1 M solution) to remove the Boc protecting group. The reaction was stirred for one hour at room temperature and the solvent was evaporated under reduced pressure and dried on high vacuum overnight. Then, a mixture of the crude Boc deprotected product and sodium bicarbonate (4.21 mmol, 5 eq.) was dissolved in 2 mL of 1,4-dioxane and 4 mL of water and stirred. 9-Fluorenylmethoxycarbonyl chloride (1.05 mmol, 1.25 eq.) was separately dissolved in 2 mL of 1,4-dioxane and added to the reaction. The reaction was stirred overnight at room temperature, then suspended in water, extracted three times with EtOAc, dried over Na_2SO_4, and evaporated under reduced pressure. To deprotect the methyl ester, a mixture of the crude Fmoc protected product and trimethyltin hydroxide (1.26 mmol, 1.5 eq.) was dissolved in 8 mL of 1,2-dichloroethane (0.1 M) and stirred at 80°C under reflux for three hours. The reaction was then cooled down to room temperature, suspended in ethyl acetate, washed two times with 1 M hydrochloric acid, and dried over Na_2SO_4. The crude product was then dry loaded onto a 10G SNAP column, purified by flash column chromatography using 0-100% EtOAc/Hexanes, and yielded **I25b** as a yellow solid. Yield: 76%

I25c

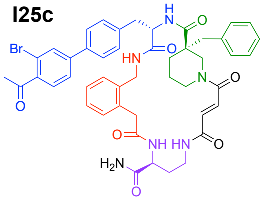

I25c (186): Macrocycle was synthesized using **general procedure for solid-phase peptide synthesis of macrocycles inhibitors**. **I25c** was obtained as a white powder. Yield: 7%
Resin: Rink Amide
4th Building Block: N^{α}-Fmoc-N^{γ}-allyloxycarbonyl-L-2,4-diaminobutyric acid
1st Building Block**:** Fmoc-2-aminomethyl-phenylacetic acid
2nd Building Block: **I25b**
3rd Building Block: (S)-Fmoc-3-benzyl-piperidine-3-carboxylic acid

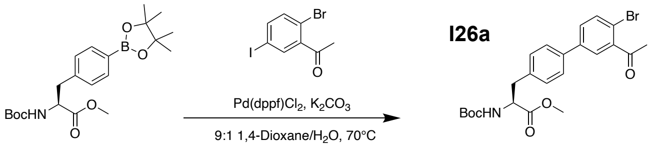

I26a (187): Compound was synthesized and purified as described in **I25a** with 1-(2-Bromo-5-iodophenyl)ethanone, affording **I26a** as a yellow foam. Yield: 37% yield

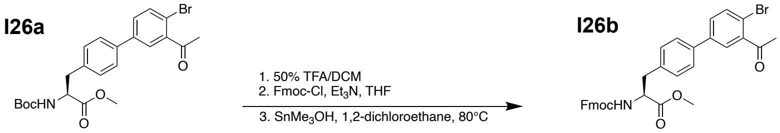

I26b (188): Compound was synthesized and purified as described in **I25b,** with **I26a** (0.73 mmol, 1 eq.), affording **I26b** as a yellow solid. Yield: 55%

I26c

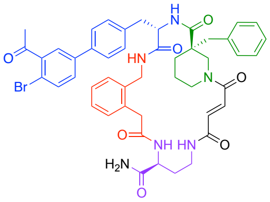

I26c (189): Macrocycle was assembled using **general procedure for solid-phase peptide synthesis of macrocycles inhibitors** and was obtained as a white powder. Yield: 7%
Resin: Rink Amide
4th Building Block: N^{α}-Fmoc-N^{γ}-allyloxycarbonyl-L-2,4-diaminobutyric acid
1st Building Block**:** Fmoc-2-aminomethyl-phenylacetic acid
2nd Building Block: **I26b**
3rd Building Block: (S)-Fmoc-3-benzyl-piperidine-3-carboxylic acid

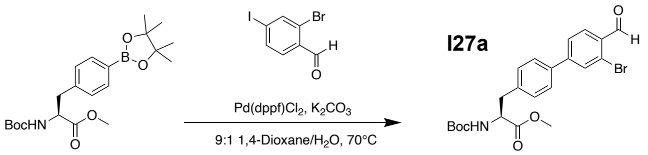

I27a (190): Compound was synthesized and purified as described in **I25a** with 2-Bromo-4-iodobenzaldehyde, affording **I27a** as a yellow foam. Yield: 46%

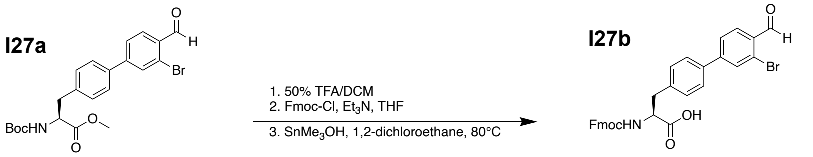

I27b (191): Compound was synthesized and purified as described in **I25b**, with **I27a** (0.91 mmol, 1 eq.), affording **I27b** as a yellow solid. Yield: 63%

I27c

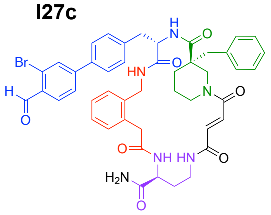

I27c (192): Macrocycle was assembled using **general procedure for solid-phase peptide synthesis of macrocycle inhibitors** and was obtained as a white powder. Yield: 1%
Resin: Rink Amide
4th Building Block: Nα-Fmoc-Nγ-allyloxycarbonyl-L-2,4-diaminobutyric acid
1st Building Block: Fmoc-2-aminomethyl-phenylacetic acid
2nd Building Block: **I27b**
3rd Building Block: (S)-Fmoc-3-benzyl-piperidine-3-carboxylic acid

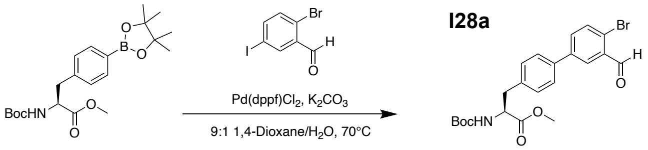

I28a (193): Compound was synthesized and purified as described in **I25a** with 2-Bromo-5-iodobenzaldehyde, affording **I28a** as a yellow foam. Yield: 42%

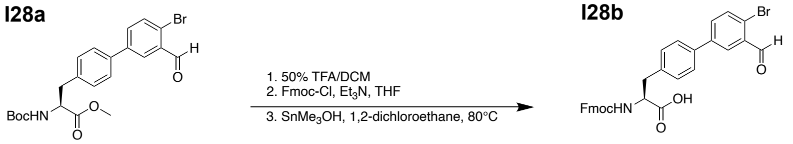

I28b (194): Compound was synthesized and purified as described in **I25b**, with **I28a** (0.87 mmol, 1eq.), affording **I28b** as a yellow solid. Yield: 36%

I28c

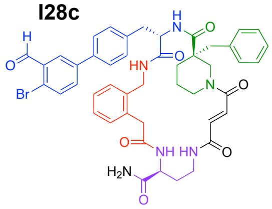

I28c (195): Macrocycle was assembled using **general procedure for solid-phase peptide synthesis of macrocycles inhibitors** and was obtained as a white powder. Yield: 1%
Resin: Rink Amide
4th Building Block: Nα-Fmoc-Nγ-allyloxycarbonyl-L-2,4-diaminobutyric acid
1st Building Block: Fmoc-2-aminomethyl-phenylacetic acid

2nd Building Block: **I28b**
3rd Building Block: (S)-Fmoc-3-benzyl-piperidine-3-carboxylic acid

I29a

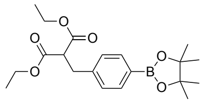

I29a (196): Compound was synthesized using **generalized Miyaura borylation conditions** with Diethyl 2-(4-bromobenzyl)malonate and no acidic workup. Crude reaction was directly purified by column chromatography (0-50%EtOAc/Hex) to afford product as white powder with small amounts of bis(pinacolato)diboron. Yield: approx quantitative

I29b

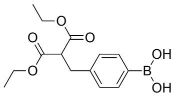

I29b (197): Compound was synthesized using **general pinacol deprotection conditions** with **I29a** and 5% TFA/CH_2Cl_2 as solvent. Product **I29b** was isolated as a white solid. Yield: quantitative

I30a

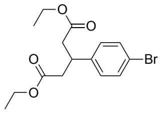

I30a (198): Compound was synthesized using **general conditions for ethyl ester protection of carboxylic acids** using B-(4-bromophenyl)glutaric acid. Product **I30a** was isolated as a brown oil. Yield: 86%

I30b

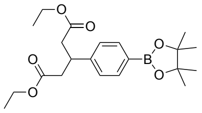

I30b (199): Compound was synthesized using **generalized Miyaura borylation conditions** with **I30a** and no acidic workup. Crude reaction was directly purified by column chromatography (0-50%EtOAc/Hex) to afford product as white powder with trace amounts of bis(pinacolato)diboron. Yield: 88%

I30c

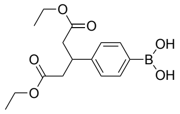

I30c (200): Compound was synthesized using **general pinacol deprotection conditions** with **I30b** and 5% TFA/CH_2Cl_2 as solvent. Product **I30b** was isolated as a white solid. Yield: quantitative

(4Br)B6

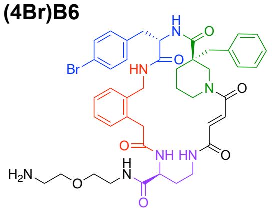

(4Br)B6 (201): Macrocycle was assembled using **general procedure for solid-phase peptide synthesis of macrocycle inhibitors**.
Resin: Bis-(2-aminoethyl)-ether trityl
4th Building Block: N^{α}-Fmoc-N^{γ}-allyloxycarbonyl-L-2,4-diaminobutyric acid
1st Building Block: Fmoc-2-aminomethyl-phenylacetic acid
2nd Building Block: Fmoc-4-bromo-L-phenylalanine
3rd Building Block: (S)-Fmoc-3-benzyl-piperidine-3-carboxylic acid

(4Br)B6-A

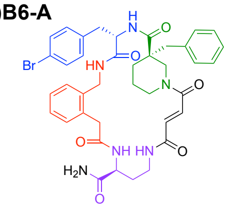

(4Br)B6-A (202): Macrocycle was assembled using **general procedure for solid-phase peptide synthesis of macrocycle inhibitors**.
Resin: NovaPEG Rink Amide
4th Building Block: N^{α}-Fmoc-N^{γ}-allyloxycarbonyl-L-2,4-diaminobutyric acid
1st Building Block: Fmoc-2-aminomethyl-phenylacetic acid
2nd Building Block: Fmoc-4-bromo-L-phenylalanine
3rd Building Block: (S)-Fmoc-3-benzyl-piperidine-3-carboxylic acid

(4Br)B6-B

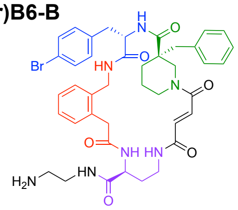

(4Br)B6-B (203): Macrocycle was assembled using **general procedure for solid-phase peptide synthesis of macrocycle inhibitors**.
Resin: Universal NovaTag
4th Building Block: N^{α}-Fmoc-N^{γ}-allyloxycarbonyl-L-2,4-diaminobutyric acid
1st Building Block: Fmoc-2-aminomethyl-phenylacetic acid
2nd Building Block: Fmoc-4-bromo-L-phenylalanine
3rd Building Block: (S)-Fmoc-3-benzyl-piperidine-3-carboxylic acid

(4Br)B6-Fl

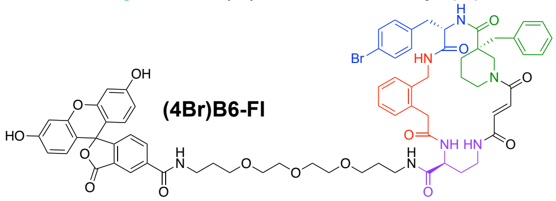

(4Br)B6-Fl (204): Macrocycle was assembled using **general procedure for solid-phase peptide synthesis of macrocycle inhibitors**. Prior to acidic cleavage, resin was suspended with three 1-hour treatments of 1M hydroxybenzotriazole monohydrate dissolved in 1:1 CH_2Cl_2/trifluoroethanol to remove Mmt group. 5-carboxyfluorescein (5 eq) and (HATU, 4.75 equiv.) were dissolved in a separate flask in ~10-15 mL DMF then treated with *N,N'*-diisopropylethylamine (DIPEA, 10 eq) for 5 minutes, observing a color change. This flask was then transferred to resin swollen in ~10 mL DMF and allowed to mix for 24 hours. Fluorescein labeled peptide were then cleaved from resin and purified as described in general procedure.
Resin: Universal PEG NovaTag™_Resin
4th Building Block: N^{α}-Fmoc-N^{γ}-allyloxycarbonyl-L-2,4-diaminobutyric acid
1st Building Block**:** Fmoc-2-aminomethyl-phenylacetic acid
2nd Building Block: Fmoc-4-bromo-L-phenylalanine
3rd Building Block: (S)-Fmoc-3-benzyl-piperidine-3-carboxylic acid

***(4Br)B6-A**

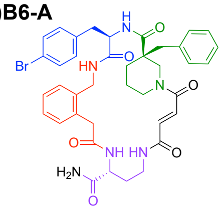

***(4Br)B6-A (205)**: Macrocycle was assembled using **general procedure for solid-phase peptide synthesis of macrocycle inhibitors**.
Resin: NovaPEG Rink Amide
4th Building Block: N^{α}-Fmoc-N^{γ}-allyloxycarbonyl-D-2,4-diaminobutyric acid

1st Building Block: Fmoc-2-aminomethyl-phenylacetic acid
2nd Building Block: Fmoc-4-bromo-L-phenylalanine
3rd Building Block: (R)-Fmoc-3-benzyl-piperidine-3-carboxylic acid

***(4Br)B6-B**

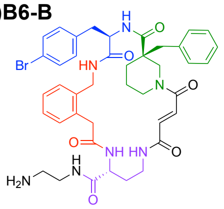

***(4Br)B6-B (206)**: Macrocycle was assembled using **general procedure for solid-phase peptide synthesis of macrocycle inhibitors**.
Resin: Universal NovaTag
4th Building Block: N^{α}-Fmoc-N^{γ}-allyloxycarbonyl-D-2,4-diaminobutyric acid
1st Building Block: Fmoc-2-aminomethyl-phenylacetic acid
2nd Building Block: Fmoc-4-bromo-L-phenylalanine
3rd Building Block: (R)-Fmoc-3-benzyl-piperidine-3-carboxylic acid

Synthesis of final macrocycles

^{1}H-NMR spectra are provided in a separate part of the Supplementary Information. HRMS characterization for intermediates are shown in Supplementary Table 8.

JOMBt

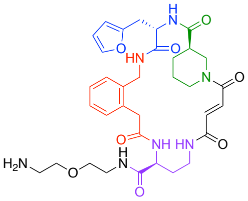

JOMBc

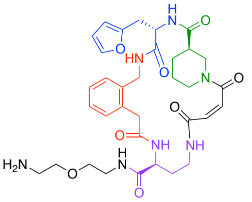

JOMBt/c: Macrocycle was assembled using **general procedure for solid-phase peptide synthesis of macrocycle inhibitors**.
Resin: Bis-(2-aminoethyl)-ether trityl
4th Building Block: N^{α}-Fmoc-N^{γ}-allyloxycarbonyl-L-2,4-diaminobutyric acid
1st Building Block: Fmoc-2-aminomethyl-phenylacetic acid
2nd Building Block: Fmoc-β-(2-furyl)-Ala-OH
3rd Building Block: Fmoc-D-nipecotic acid

JOMBt-A

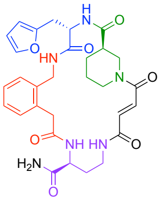

JOMBt-A: Macrocycle was assembled using **general procedure for solid-phase peptide synthesis of macrocycle inhibitors**.
Resin: NovaPEG Rink Amide
4th Building Block: N^{α}-Fmoc-N^{γ}-allyloxycarbonyl-L-2,4-diaminobutyric acid
1st Building Block: Fmoc-2-aminomethyl-phenylacetic acid
2nd Building Block: Fmoc-β-(2-furyl)-Ala-OH
3rd Building Block: Fmoc-D-nipecotic acid

JOMBt-D

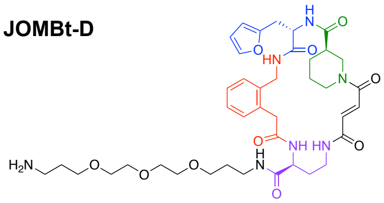

JOMBt-D: Macrocycle was assembled using **general procedure for solid-phase peptide synthesis of macrocycle inhibitors**.
Resin: Universal PEG NovaTag™_Resin
4th Building Block: N$^{\alpha}$-Fmoc-N$^{\gamma}$-allyloxycarbonyl-L-2,4-diaminobutyric acid
1st Building Block: Fmoc-2-aminomethyl-phenylacetic acid
2nd Building Block: Fmoc-β-(2-furyl)-Ala-OH
3rd Building Block: Fmoc-D-nipecotic acid

JOBBt

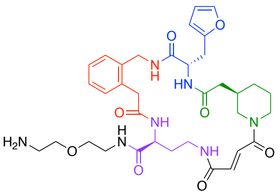

JOBBt: Macrocycle was assembled using **general procedure for solid-phase peptide synthesis of macrocycle inhibitors**.
Resin: Bis-(2-aminoethyl)-ether trityl
4th Building Block: N$^{\alpha}$-Fmoc-N$^{\gamma}$-allyloxycarbonyl-L-2,4-diaminobutyric acid
1st Building Block: Fmoc-2-aminomethyl-phenylacetic acid
2nd Building Block: Fmoc-β-(2-furyl)-Ala-OH
3rd Building Block: (R)-(1-Fmoc-piperidin-3-yl)acetic acid

JOBBc-A

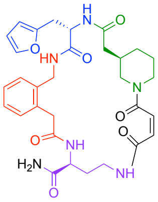

JOBBc-A: Macrocycle was assembled using **general procedure for solid-phase peptide synthesis of macrocycle inhibitors**.
Resin: NovaPEG Rink Amide
4th Building Block: N$^{\alpha}$-Fmoc-N$^{\gamma}$-allyloxycarbonyl-L-2,4-diaminobutyric acid
1st Building Block: Fmoc-2-aminomethyl-phenylacetic acid
2nd Building Block: Fmoc-β-(2-furyl)-Ala-OH
3rd Building Block: (R)-(1-Fmoc-piperidin-3-yl)acetic acid

JOCBt

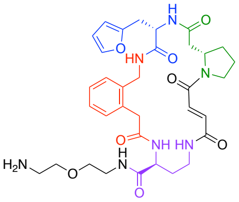

JOCBc

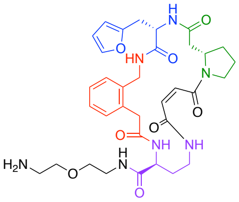

JOCBt/c: Macrocycle was assembled using **general procedure for solid-phase peptide synthesis of macrocycle inhibitors**.
Resin: Bis-(2-aminoethyl)-ether trityl
4th Building Block: N$^{\alpha}$-Fmoc-N$^{\gamma}$-allyloxycarbonyl-L-2,4-diaminobutyric acid
1st Building Block: Fmoc-2-aminomethyl-phenylacetic acid
2nd Building Block: Fmoc-β-(2-furyl)-Ala-OH
3rd Building Block: Fmoc-L-β-HomoPro-OH

JOGAt

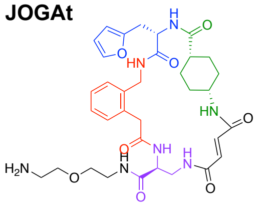

JOGAc

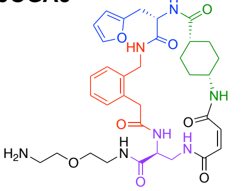

JOGAt/c: Macrocycle was assembled using **general procedure for solid-phase peptide synthesis of macrocycle inhibitors**.
Resin: Bis-(2-aminoethyl)-ether trityl
4th Building Block: N^{α}-Fmoc-N^{β}-allyloxycarbonyl-L-2,3-diamino-propionic acid
1st Building Block: Fmoc-2-aminomethyl-phenylacetic acid
2nd Building Block: Fmoc-β-(2-furyl)-Ala-OH
3rd Building Block: Fmoc-cis-4-aminocyclohexane carboxylic acid

JOGBt-A

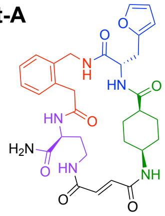

JOGBc-A

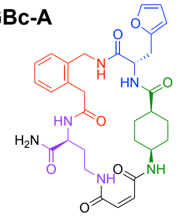

JOGBt/c-A: Macrocycle was assembled using **general procedure for solid-phase peptide synthesis of macrocycle inhibitors**.
Resin: NovaPEG Rink Amide
4th Building Block: N^{α}-Fmoc-N^{γ}-allyloxycarbonyl-L-2,4-diaminobutyric acid
1st Building Block: Fmoc-2-aminomethyl-phenylacetic acid
2nd Building Block: Fmoc-β-(2-furyl)-Ala-OH
3rd Building Block: Fmoc-cis-4-aminocyclohexane carboxylic acid

JOMAt

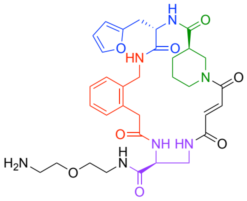

JOMAc

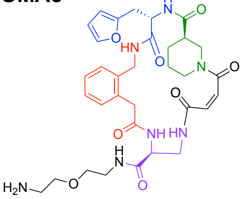

JOMAt/c: Macrocycle was assembled using **general procedure for solid-phase peptide synthesis of macrocycle inhibitors**.
Resin: Bis-(2-aminoethyl)-ether trityl
4th Building Block: N^{α}-Fmoc-N^{β}-allyloxycarbonyl-L-2,3-diamino-propionic acid
1st Building Block: Fmoc-2-aminomethyl-phenylacetic acid
2nd Building Block: Fmoc-β-(2-furyl)-Ala-OH
3rd Building Block: Fmoc-D-nipecotic acid

JOMCt

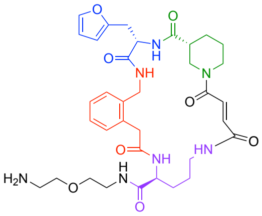

JOMCc

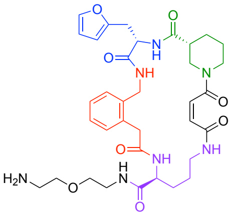

JOMCt/c: Macrocycle was assembled using **general procedure for solid-phase peptide synthesis of macrocycle inhibitors**.
Resin: Bis-(2-aminoethyl)-ether trityl
4th Building Block: N^{α}-Fmoc-N^{δ}-allyloxycarbonyl-L-ornithine

1st Building Block: Fmoc-2-aminomethyl-phenylacetic acid
2nd Building Block: Fmoc-β-(2-furyl)-Ala-OH
3rd Building Block: Fmoc-D-nipecotic acid

JOMFt

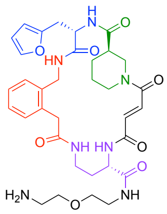

JOMFc

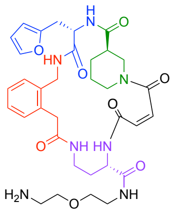

JOMFt/c: Macrocycle was assembled using **general procedure for solid-phase peptide synthesis of macrocycle inhibitors**.
Resin: Bis-(2-aminoethyl)-ether trityl
4th Building Block: 4F (N^{α}-allyloxycarbonyl-N^{β}-Fmoc-L-2,4-diamino-butyric acid)
1st Building Block: Fmoc-2-aminomethyl-phenylacetic acid
2nd Building Block: Fmoc-β-(2-furyl)-Ala-OH
3rd Building Block: Fmoc-D-nipecotic acid

HOJJt

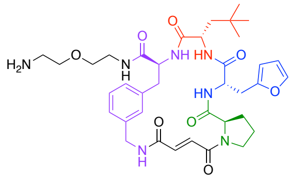

HOJJc

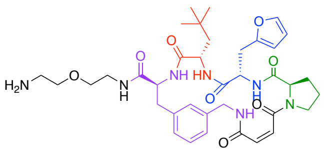

HOJJt/c: Macrocycle was assembled using **general procedure for solid-phase peptide synthesis of macrocycle inhibitors**.
Resin: Bis-(2-aminoethyl)-ether trityl
4th Building Block: 4J (Fmoc-3-(allyloxycarbonyl-aminomethyl)-L-phenylalanine)
1st Building Block: Fmoc-neopentylglycine
2nd Building Block: Fmoc-β-(2-furyl)-Ala-OH
3rd Building Block: Fmoc-D-Proline

HJJJt

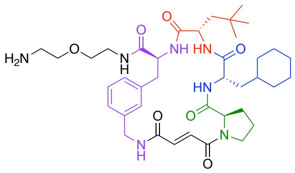

HJJJc

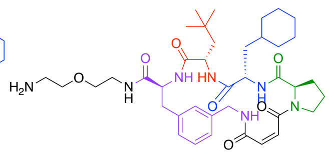

HJJJt/c: Macrocycle was assembled using **general procedure for solid-phase peptide synthesis of macrocycle inhibitors**.
Resin: Bis-(2-aminoethyl)-ether trityl
4th Building Block: 4J (Fmoc-3-(allyloxycarbonyl-aminomethyl)-L-phenylalanine)
1st Building Block: Fmoc-neopentylglycine
2nd Building Block**:** Fmoc-ß-cyclohexyl-L-alanine
3rd Building Block: Fmoc-D-Proline

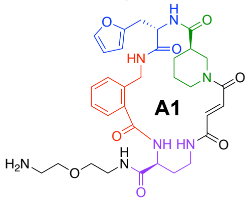

A1

A1: Macrocycle was assembled using **general procedure for solid-phase peptide synthesis of macrocycle inhibitors**.
Resin: Bis-(2-aminoethyl)-ether trityl
4th Building Block: N^{α}-Fmoc-N^{γ}-allyloxycarbonyl-L-2,4-diaminobutyric acid
1st Building Block: 2-(Fmoc-aminomethyl)benzoic acid

2nd Building Block: Fmoc-β-(2-furyl)-Ala-OH
3rd Building Block: Fmoc-D-nipecotic acid

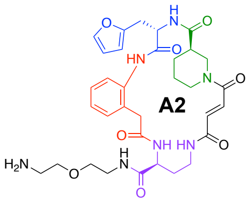

A2: Macrocycle was assembled using **general procedure for solid-phase peptide synthesis of macrocycle inhibitors.**
Resin: Bis-(2-aminoethyl)-ether trityl
4th Building Block: N^{α}-Fmoc-N^{γ}-allyloxycarbonyl-L-2,4-diaminobutyric acid
1st Building Block: Fmoc-(2-aminophenyl)acetic acid
2nd Building Block: Fmoc-β-(2-furyl)-Ala-OH
3rd Building Block: Fmoc-D-nipecotic acid

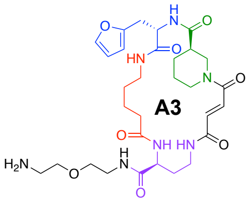

A3: Macrocycle was assembled using **general procedure for solid-phase peptide synthesis of macrocycle inhibitors.**
Resin: Bis-(2-aminoethyl)-ether trityl
4th Building Block: N^{α}-Fmoc-N^{γ}-allyloxycarbonyl-L-2,4-diaminobutyric acid
1st Building Block**:** Fmoc-5-aminovaleric acid
2nd Building Block: Fmoc-β-(2-furyl)-Ala-OH
3rd Building Block: Fmoc-D-nipecotic acid

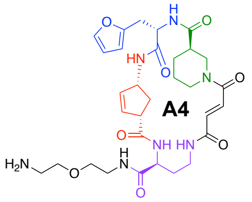

A4: Macrocycle was assembled using **general procedure for solid-phase peptide synthesis of macrocycle inhibitors.**
Resin: Bis-(2-aminoethyl)-ether trityl
4th Building Block: N^{α}-Fmoc-N^{γ}-allyloxycarbonyl-L-2,4-diaminobutyric acid
1st Building Block: (Fmoc-(-)-(1*S*,4*R*)-4-Aminocyclopent-2-enecarboxylic acid) (AAP_02_046) I1
2nd Building Block: Fmoc-β-(2-furyl)-Ala-OH
3rd Building Block: Fmoc-D-nipecotic acid

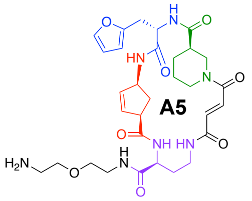

A5: Macrocycle was assembled using **general procedure for solid-phase peptide synthesis of macrocycle inhibitors.**
Resin: Bis-(2-aminoethyl)-ether trityl
4th Building Block: N^{α}-Fmoc-N^{γ}-allyloxycarbonyl-L-2,4-diaminobutyric acid
1st Building Block: Fmoc-(+)-(1*R*,4*S*)-4-Aminocyclopent-2-enecarboxylic acid (AAP_02_047) I2
2nd Building Block: Fmoc-β-(2-furyl)-Ala-OH
3rd Building Block: Fmoc-D-nipecotic acid

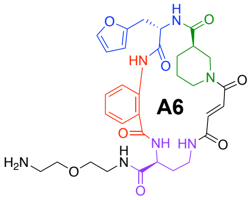

A6: Macrocycle was assembled using **general procedure for solid-phase peptide synthesis of macrocycle inhibitors.**
Resin: Bis-(2-aminoethyl)-ether trityl
4th Building Block: N^{α}-Fmoc-N^{γ}-allyloxycarbonyl-L-2,4-diaminobutyric acid
1st Building Block**:** *N*-Fmoc-anthranilic acid
2nd Building Block: Fmoc-β-(2-furyl)-Ala-OH
3rd Building Block: Fmoc-D-nipecotic acid

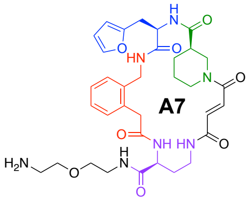

A7: Macrocycle was assembled using **general procedure for solid-phase peptide synthesis of macrocycle inhibitors.**
Resin: Bis-(2-aminoethyl)-ether trityl
4th Building Block: N^{α}-Fmoc-N^{γ}-allyloxycarbonyl-L-2,4-diaminobutyric acid
1st Building Block: Fmoc-2-aminomethyl-phenylacetic acid
2nd Building Block: Fmoc-β-(2-furyl)-D-Ala-OH
3rd Building Block: Fmoc-D-nipecotic acid

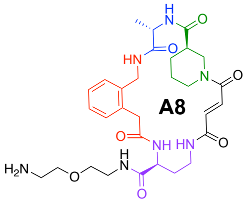

A8: Macrocycle was assembled using **general procedure for solid-phase peptide synthesis of macrocycle inhibitors.**
Resin: Bis-(2-aminoethyl)-ether trityl
4th Building Block: N^{α}-Fmoc-N^{γ}-allyloxycarbonyl-L-2,4-diaminobutyric acid
1st Building Block: Fmoc-2-aminomethyl-phenylacetic acid
2nd Building Block: Fmoc-L-alanine
3rd Building Block: Fmoc-D-nipecotic acid

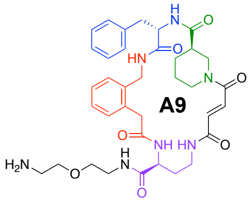

A9: Macrocycle was assembled using **general procedure for solid-phase peptide synthesis of macrocycle inhibitors.**
Resin: Bis-(2-aminoethyl)-ether trityl
4th Building Block: N^{α}-Fmoc-N^{γ}-allyloxycarbonyl-L-2,4-diaminobutyric acid
1st Building Block: Fmoc-2-aminomethyl-phenylacetic acid
2nd Building Block: Fmoc-L-phenylalanine
3rd Building Block: Fmoc-D-nipecotic acid

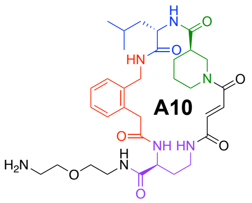

A10: Macrocycle was assembled using **general procedure for solid-phase peptide synthesis of macrocycle inhibitors.**
Resin: Bis-(2-aminoethyl)-ether trityl
4th Building Block: N^{α}-Fmoc-N^{γ}-allyloxycarbonyl-L-2,4-diaminobutyric acid
1st Building Block: Fmoc-2-aminomethyl-phenylacetic acid
2nd Building Block: Fmoc-L-Leucine
3rd Building Block: Fmoc-D-nipecotic acid

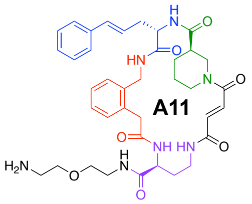

A11: Macrocycle was assembled using **general procedure for solid-phase peptide synthesis of macrocycle inhibitors.**
Resin: Bis-(2-aminoethyl)-ether trityl
4th Building Block: N^{α}-Fmoc-N^{γ}-allyloxycarbonyl-L-2,4-diaminobutyric acid
1st Building Block: Fmoc-2-aminomethyl-phenylacetic acid
2nd Building Block**:** Fmoc-3-styryl-L-alanine
3rd Building Block: Fmoc-D-nipecotic acid

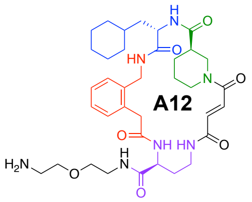

A12: Macrocycle was assembled using **general procedure for solid-phase peptide synthesis of macrocycle inhibitors.**
Resin: Bis-(2-aminoethyl)-ether trityl
4th Building Block: N^{α}-Fmoc-N^{γ}-allyloxycarbonyl-L-2,4-diaminobutyric acid
1st Building Block: Fmoc-2-aminomethyl-phenylacetic acid
2nd Building Block**:** Fmoc-ß-cyclohexyl-L-alanine
3rd Building Block: Fmoc-D-nipecotic acid

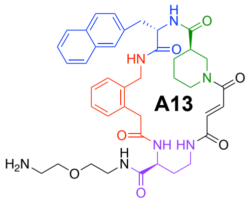

A13: Macrocycle was assembled using **general procedure for solid-phase peptide synthesis of macrocycle inhibitors.**
Resin: Bis-(2-aminoethyl)-ether trityl
4th Building Block: N^{α}-Fmoc-N^{γ}-allyloxycarbonyl-L-2,4-diaminobutyric acid
1st Building Block: Fmoc-2-aminomethyl-phenylacetic acid
2nd Building Block**:** Fmoc-3-(2-naphthyl)-L-alanine
3rd Building Block: Fmoc-D-nipecotic acid

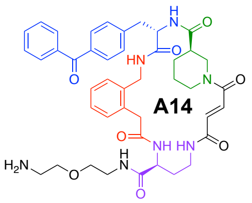

A14: Macrocycle was assembled using **general procedure for solid-phase peptide synthesis of macrocycle inhibitors**.
Resin: Bis-(2-aminoethyl)-ether trityl
4th Building Block: N^{α}-Fmoc-N^{γ}-allyloxycarbonyl-L-2,4-diaminobutyric acid
1st Building Block: Fmoc-2-aminomethyl-phenylacetic acid
2nd Building Block**:** Fmoc-4-benzoyl-L-phenylalanine
3rd Building Block: Fmoc-D-nipecotic acid

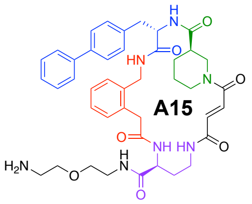

A15: Macrocycle was assembled using **general procedure for solid-phase peptide synthesis of macrocycle inhibitors**.
Resin: Bis-(2-aminoethyl)-ether trityl
4th Building Block: N^{α}-Fmoc-N^{γ}-allyloxycarbonyl-L-2,4-diaminobutyric acid
1st Building Block: Fmoc-2-aminomethyl-phenylacetic acid
2nd Building Block**:** Fmoc-*p*-phenyl-L-phenylalanine
3rd Building Block: Fmoc-D-nipecotic acid

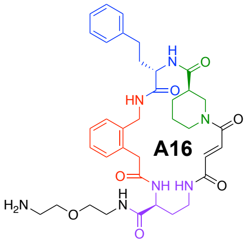

A16: Macrocycle was assembled using **general procedure for solid-phase peptide synthesis of macrocycle inhibitors**.
Resin: Bis-(2-aminoethyl)-ether trityl
4th Building Block: N^{α}-Fmoc-N^{γ}-allyloxycarbonyl-L-2,4-diaminobutyric acid
1st Building Block: Fmoc-2-aminomethyl-phenylacetic acid
2nd Building Block**:** Fmoc-L-Homophenylalanine
3rd Building Block: Fmoc-D-nipecotic acid

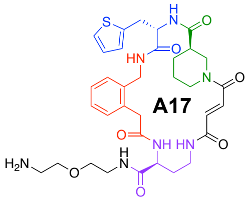

A17: Macrocycle was assembled using **general procedure for solid-phase peptide synthesis of macrocycle inhibitors**.
Resin: Bis-(2-aminoethyl)-ether trityl
4th Building Block: N^{α}-Fmoc-N^{γ}-allyloxycarbonyl-L-2,4-diaminobutyric acid
1st Building Block: Fmoc-2-aminomethyl-phenylacetic acid
2nd Building Block**:** Fmoc-β-(2-thienyl)-L-alanine
3rd Building Block: Fmoc-D-nipecotic acid

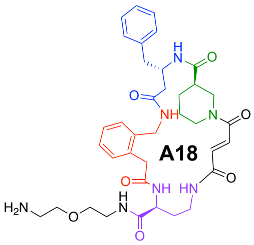

A18: Macrocycle was assembled using **general procedure for solid-phase peptide synthesis of macrocycle inhibitors.**
Resin: Bis-(2-aminoethyl)-ether trityl
4th Building Block: Nα-Fmoc-Nγ-allyloxycarbonyl-L-2,4-diaminobutyric acid
1st Building Block: Fmoc-2-aminomethyl-phenylacetic acid
2nd Building Block**:** Fmoc-L-β-homophenylalanine
3rd Building Block: Fmoc-D-nipecotic acid

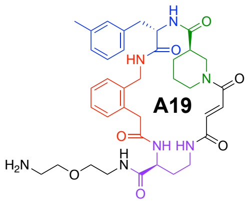

A19: Macrocycle was assembled using **general procedure for solid-phase peptide synthesis of macrocycle inhibitors.**
Resin: Bis-(2-aminoethyl)-ether trityl
4th Building Block: Nα-Fmoc-Nγ-allyloxycarbonyl-L-2,4-diaminobutyric acid
1st Building Block: Fmoc-2-aminomethyl-phenylacetic acid
2nd Building Block**:** Fmoc-3-methyl-L-phenylalanine
3rd Building Block: Fmoc-D-nipecotic acid

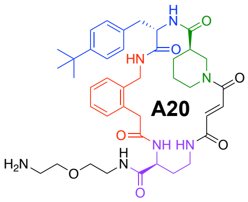

A20: Macrocycle was assembled using **general procedure for solid-phase peptide synthesis of macrocycle inhibitors.**
Resin: Bis-(2-aminoethyl)-ether trityl
4th Building Block: Nα-Fmoc-Nγ-allyloxycarbonyl-L-2,4-diaminobutyric acid
1st Building Block: Fmoc-2-aminomethyl-phenylacetic acid
2nd Building Block**:** Fmoc-4-*tert*-butyl-L-phenylalanine
3rd Building Block: Fmoc-D-nipecotic acid

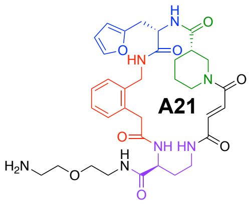

A21: Macrocycle was assembled using **general procedure for solid-phase peptide synthesis of macrocycle inhibitors.**
Resin: Bis-(2-aminoethyl)-ether trityl
4th Building Block: Nα-Fmoc-Nγ-allyloxycarbonyl-L-2,4-diaminobutyric acid
1st Building Block: Fmoc-2-aminomethyl-phenylacetic acid
2nd Building Block: Fmoc-β-(2-furyl)-Ala-OH
3rd Building Block: Fmoc-L-nipecotic acid

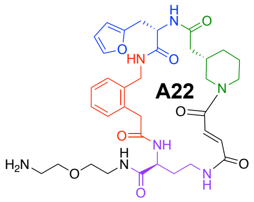

A22: Macrocycle was assembled using **general procedure for solid-phase peptide synthesis of macrocycle inhibitors.**
Resin: Bis-(2-aminoethyl)-ether trityl
4th Building Block: N^{α}-Fmoc-N^{γ}-allyloxycarbonyl-L-2,4-diaminobutyric acid
1st Building Block: Fmoc-2-aminomethyl-phenylacetic acid
2nd Building Block: Fmoc-β-(2-furyl)-Ala-OH
3rd Building Block: (S)-(1-Fmoc-piperidin-3-yl)acetic acid

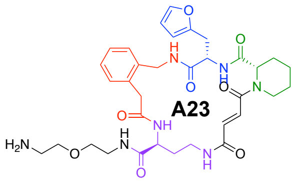

A23: Macrocycle was assembled using **general procedure for solid-phase peptide synthesis of macrocycle inhibitors.**
Resin: Bis-(2-aminoethyl)-ether trityl
4th Building Block: N^{α}-Fmoc-N^{γ}-allyloxycarbonyl-L-2,4-diaminobutyric acid
1st Building Block: Fmoc-2-aminomethyl-phenylacetic acid
2nd Building Block: Fmoc-β-(2-furyl)-Ala-OH
3rd Building Block: Fmoc-L-homoproline

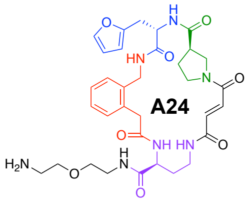

A24: Macrocycle was assembled using **general procedure for solid-phase peptide synthesis of macrocycle inhibitors.**
Resin: Bis-(2-aminoethyl)-ether trityl
4th Building Block: N^{α}-Fmoc-N^{γ}-allyloxycarbonyl-L-2,4-diaminobutyric acid
1st Building Block: Fmoc-2-aminomethyl-phenylacetic acid
2nd Building Block: Fmoc-β-(2-furyl)-Ala-OH
3rd Building Block: **I3**

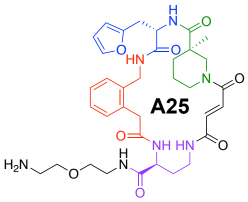

A25: Macrocycle was assembled using **general procedure for solid-phase peptide synthesis of macrocycle inhibitors.**
Resin: Bis-(2-aminoethyl)-ether trityl
4th Building Block: N^{α}-Fmoc-N^{γ}-allyloxycarbonyl-L-2,4-diaminobutyric acid
1st Building Block**:** Fmoc-2-aminomethyl-phenylacetic acid
2nd Building Block: Fmoc-β-(2-furyl)-Ala-OH
3rd Building Block: (R)-Fmoc-3-methyl-piperidine-3-carboxylic acid

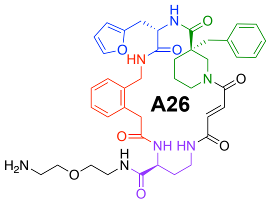

A26: Macrocycle was assembled using **general procedure for solid-phase peptide synthesis of macrocycle inhibitors**.
Resin: Bis-(2-aminoethyl)-ether trityl
4th Building Block: N^{α}-Fmoc-N^{γ}-allyloxycarbonyl-L-2,4-diaminobutyric acid
1st Building Block**:** Fmoc-2-aminomethyl-phenylacetic acid
2nd Building Block: Fmoc-β-(2-furyl)-Ala-OH
3rd Building Block: (S)-Fmoc-3-benzyl-piperidine-3-carboxylic acid

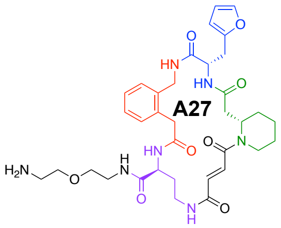

A27: Macrocycle was assembled using **general procedure for solid-phase peptide synthesis of macrocycle inhibitors**.
Resin: Bis-(2-aminoethyl)-ether trityl
4th Building Block: N^{α}-Fmoc-N^{γ}-allyloxycarbonyl-L-2,4-diaminobutyric acid
1st Building Block**:** Fmoc-2-aminomethyl-phenylacetic acid
2nd Building Block: Fmoc-β-(2-furyl)-Ala-OH
3rd Building Block: (S)-(1-Fmoc-piperidin-2-yl)acetic acid

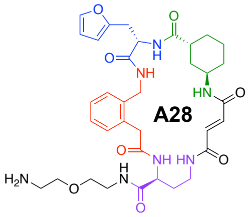

A28: Macrocycle was assembled using **general procedure for solid-phase peptide synthesis of macrocycle inhibitors**.
Resin: Bis-(2-aminoethyl)-ether trityl
4th Building Block: N^{α}-Fmoc-N^{γ}-allyloxycarbonyl-L-2,4-diaminobutyric acid
1st Building Block**:** Fmoc-2-aminomethyl-phenylacetic acid
2nd Building Block: Fmoc-β-(2-furyl)-Ala-OH
3rd Building Block: **I4**

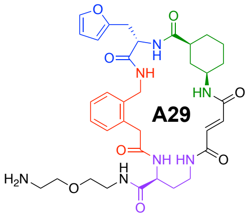

A29: Macrocycle was assembled using **general procedure for solid-phase peptide synthesis of macrocycle inhibitors**.
Resin: Bis-(2-aminoethyl)-ether trityl
4th Building Block: N^{α}-Fmoc-N^{γ}-allyloxycarbonyl-L-2,4-diaminobutyric acid
1st Building Block**:** Fmoc-2-aminomethyl-phenylacetic acid
2nd Building Block: Fmoc-β-(2-furyl)-Ala-OH
3rd Building Block: ((1S,3R)-3-(Fmoc-amino)cyclohexane-1-carboxylic acid)

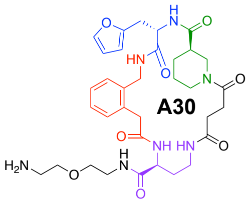

A30: Macrocycle was assembled using **general procedure for solid-phase peptide synthesis of macrocycle inhibitors**, using conditions stipulated in ‘trans’ isomer procedure, but replacing allyl-fumarate-monoester+HATU with succinic anhydride.
Resin: Bis-(2-aminoethyl)-ether trityl
4th Building Block: N^{α}-Fmoc-N^{γ}-allyloxycarbonyl-L-2,4-diaminobutyric acid
1st Building Block: Fmoc-2-aminomethyl-phenylacetic acid
2nd Building Block: Fmoc-β-(2-furyl)-Ala-OH
3rd Building Block: Fmoc-D-nipecotic acid

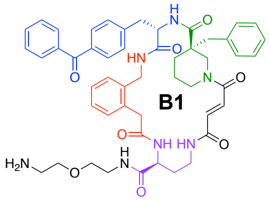

B1: Macrocycle was assembled using **general procedure for solid-phase peptide synthesis of macrocycle inhibitors**.
Resin: Bis-(2-aminoethyl)-ether trityl
4th Building Block: N^{α}-Fmoc-N^{γ}-allyloxycarbonyl-L-2,4-diaminobutyric acid
1st Building Block**:** Fmoc-2-aminomethyl-phenylacetic acid
2nd Building Block: Fmoc-4-benzoyl-L-phenylalanine
3rd Building Block: (S)-Fmoc-3-benzyl-piperidine-3-carboxylic acid

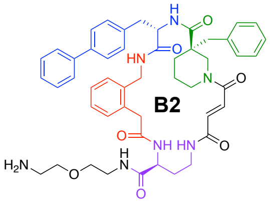

B2: Macrocycle was assembled using **general procedure for solid-phase peptide synthesis of macrocycle inhibitors**.
Resin: Bis-(2-aminoethyl)-ether trityl
4th Building Block: N^{α}-Fmoc-N^{γ}-allyloxycarbonyl-L-2,4-diaminobutyric acid
1st Building Block**:** Fmoc-2-aminomethyl-phenylacetic acid
2nd Building Block**:** Fmoc-*p*-phenyl-L-phenylalanine
3rd Building Block: (S)-Fmoc-3-benzyl-piperidine-3-carboxylic acid

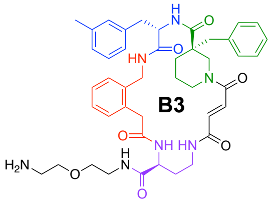

B3: Macrocycle was assembled using **general procedure for solid-phase peptide synthesis of macrocycle inhibitors**.
Resin: Bis-(2-aminoethyl)-ether trityl
4th Building Block: N^{α}-Fmoc-N^{γ}-allyloxycarbonyl-L-2,4-diaminobutyric acid
1st Building Block**:** Fmoc-2-aminomethyl-phenylacetic acid
2nd Building Block**:** Fmoc-3-methyl-L-phenylalanine
3rd Building Block: (S)-Fmoc-3-benzyl-piperidine-3-carboxylic acid

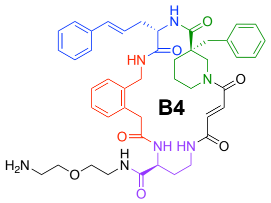

B4: Macrocycle was assembled using **general procedure for solid-phase peptide synthesis of macrocycle inhibitors**.
Resin: Bis-(2-aminoethyl)-ether trityl
4th Building Block: N^{α}-Fmoc-N^{γ}-allyloxycarbonyl-L-2,4-diaminobutyric acid
1st Building Block**:** Fmoc-2-aminomethyl-phenylacetic acid
2nd Building Block**:** Fmoc-3-styryl-L-alanine
3rd Building Block: (S)-Fmoc-3-benzyl-piperidine-3-carboxylic acid

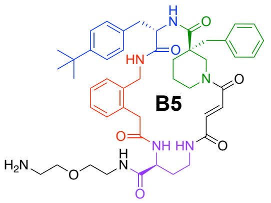

B5: Macrocycle was assembled using **general procedure for solid-phase peptide synthesis of macrocycle inhibitors**.
Resin: Bis-(2-aminoethyl)-ether trityl
4th Building Block: N^{α}-Fmoc-N^{γ}-allyloxycarbonyl-L-2,4-diaminobutyric acid
1st Building Block**:** Fmoc-2-aminomethyl-phenylacetic acid
2nd Building Block**:** Fmoc-4-*tert*-butyl-L-phenylalanine
3rd Building Block: (S)-Fmoc-3-benzyl-piperidine-3-carboxylic acid

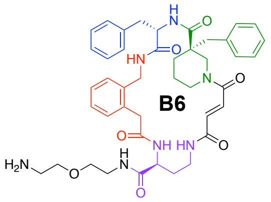

B6: Macrocycle was assembled using **general procedure for solid-phase peptide synthesis of macrocycle inhibitors**.
Resin: Bis-(2-aminoethyl)-ether trityl
4th Building Block: N^{α}-Fmoc-N^{γ}-allyloxycarbonyl-L-2,4-diaminobutyric acid
1st Building Block**:** Fmoc-2-aminomethyl-phenylacetic acid
2nd Building Block: Fmoc-L-phenylalanine
3rd Building Block: (S)-Fmoc-3-benzyl-piperidine-3-carboxylic acid

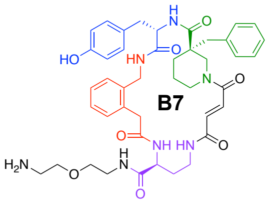

B7: Macrocycle was assembled using **general procedure for solid-phase peptide synthesis of macrocycle inhibitors**.
Resin: Bis-(2-aminoethyl)-ether trityl
4th Building Block: N^{α}-Fmoc-N^{γ}-allyloxycarbonyl-L-2,4-diaminobutyric acid
1st Building Block**:** Fmoc-2-aminomethyl-phenylacetic acid
2nd Building Block**: Fmoc-*O*-*tert*-butyl-L-tyrosine**
3rd Building Block: (S)-Fmoc-3-benzyl-piperidine-3-carboxylic acid

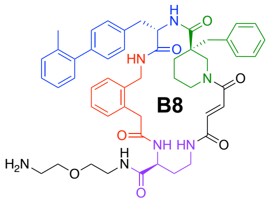

B8

B8: Compound was synthesized using **Generalized procedure A for Suzuki-Miyaura cross-coupling of macrocycles** using **(4Br)B6** and 2-methylphenylboronic acid. Yield: 78%

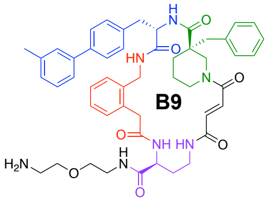

B9

B9: Compound was synthesized using **Generalized procedure A for Suzuki-Miyaura cross-coupling of macrocycles** using **(4Br)B6** and 3-methylphenylboronic acid. Yield: 64%

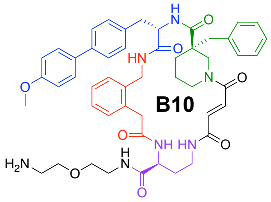

B10

B10: Compound was synthesized using **Generalized procedure A for Suzuki-Miyaura cross-coupling of macrocycles** using **(4Br)B6** and 4-methoxyphenylboronic acid. Yield: 21%

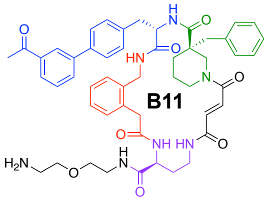

B11

B11: Compound was synthesized using **Generalized procedure A for Suzuki-Miyaura cross-coupling of macrocycles** using **(4Br)B6** and 3-acetylphenylboronic acid. Yield: 37%

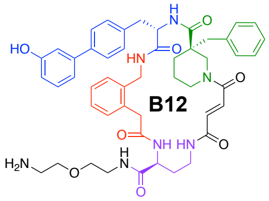

B12

B12: Compound was synthesized using **Generalized procedure A for Suzuki-Miyaura cross-coupling of macrocycles** using **(4Br)B6** and 3-hydroxyphenylboronic acid. Yield: 87%

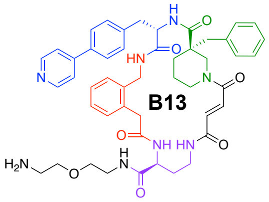

B13

B13: Macrocycle was assembled using **general procedure for solid-phase peptide synthesis of macrocycle inhibitors.**
Resin: Bis-(2-aminoethyl)-ether trityl
4th Building Block: N^{α}-Fmoc-N^{γ}-allyloxycarbonyl-L-2,4-diaminobutyric acid
1st Building Block**:** Fmoc-2-aminomethyl-phenylacetic acid

2nd Building Block: **I5**
3rd Building Block: (S)-Fmoc-3-benzyl-piperidine-3-carboxylic acid

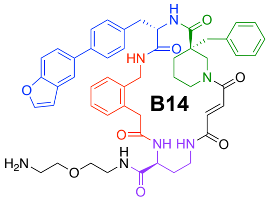

B14: Compound was synthesized using **Generalized procedure A for Suzuki-Miyaura cross-coupling of macrocycles** using **(4Br)B6** and benzofuran-5-yl-5-boronic acid. Yield: quantitative

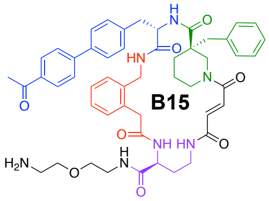

B15 Compound: Compound was synthesized using **Generalized procedure A for Suzuki-Miyaura cross-coupling of macrocycles** using **(4Br)B6** and 4-acetylphenylboronic acid. Yield: 39%

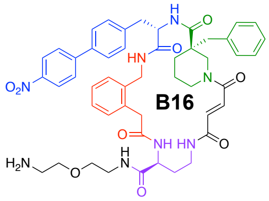

B16: Compound was synthesized using **Generalized procedure A for Suzuki-Miyaura cross-coupling of macrocycles** using **(4Br)B6** and 4-nitrophenylboronic acid. Yield: 60%

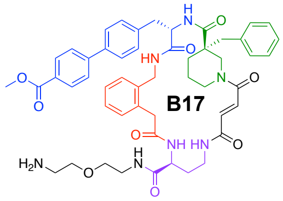

B17: Compound was synthesized using **Generalized procedure A for Suzuki-Miyaura cross-coupling of macrocycles** using **(4Br)B6** and 4-Methoxycarbonylphenylboronic. Yield: 37%

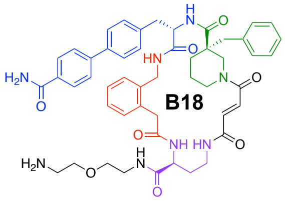

B18: Compound was synthesized using **Generalized procedure A for Suzuki-Miyaura cross-coupling of macrocycles** using **(4Br)B6** and 4-aminocarbonylphenylboronic acid. Yield: 49%

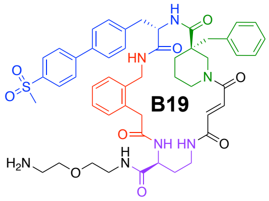

B19: Compound was synthesized using **Generalized procedure A for Suzuki-Miyaura cross-coupling of macrocycles** using **(4Br)B6** and 4-(methanesulfonyl)phenylboronic acid. Yield: 86%

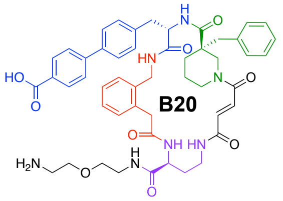

B20: Compound was synthesized using **Generalized procedure A for Suzuki-Miyaura cross-coupling of macrocycles** using **(4Br)B6** and 4-(*tert*-Butoxycarbonyl)phenylboronic acid. *Tert*-butyl protected product was treated with 1 mL TFA for 1 hour, and re-purified under same HPLC conditions to yield carboxylic acid product. Yield: 95%

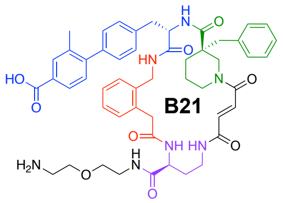

B21: Compound was synthesized using **Generalized procedure A for Suzuki-Miyaura cross-coupling of macrocycles** using **(4Br)B6** and 4-carboxy-2-methylphenylboronic acid. Yield: 35%

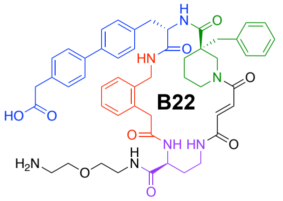

B22: Compound was synthesized using **Generalized procedure A for Suzuki-Miyaura cross-coupling of macrocycles** using **(4Br)B6** and 4-carboxymethylphenylboronic acid. Yield: 19%

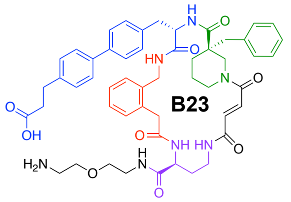

B23: Compound was synthesized using **Generalized procedure A for Suzuki-Miyaura cross-coupling of macrocycles** using **(4Br)B6** and 4-cyanomethylphenylboronic acid. Yield: 21%

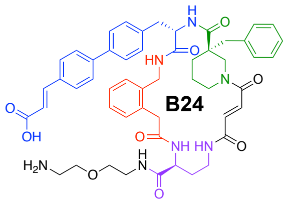

B24: Compound was synthesized using **Generalized procedure B for Suzuki-Miyaura cross-coupling of macrocycles** using **(4Br)B6** and 4-(E-2-carboxyvinyl)phenylboronic acid. Yield: 20%

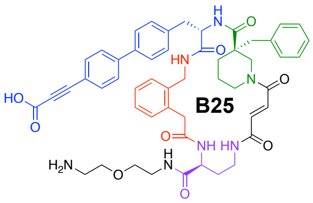

B25: Compound was synthesized using **Generalized procedure A for Suzuki-Miyaura cross-coupling of macrocycles** using **(4Br)B6** and **I6b**. *Tert*-butyl protected product was treated with 1 mL TFA for 1 hour, and re-purified under same HPLC conditions to yield carboxylic acid product. Yield: 15%

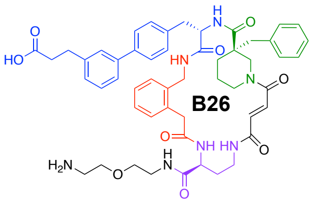

B26: Compound was synthesized using **Generalized procedure A for Suzuki-Miyaura cross-coupling of macrocycles** using **(4Br)B6** and 3-(carboxyethyl)phenylboronic acid. Yield: 9%

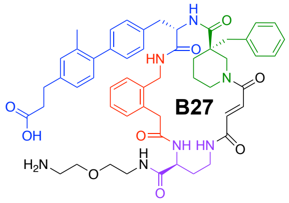

B27: Compound was synthesized using **Generalized procedure A for Suzuki-Miyaura cross-coupling of macrocycles** using **(4Br)B6** and 4-(2-carboxyethyl)-2-methylphenylboronic acid. Yield: 16%

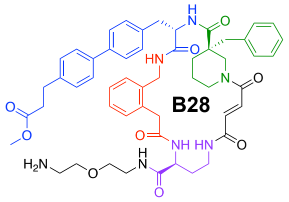

B28: Compound was synthesized using **Generalized procedure A for Suzuki-Miyaura cross-coupling of macrocycles** using **(4Br)B6** and 4-(methoxycarbonylethyl)phenylboronic acid. Yield: 72%

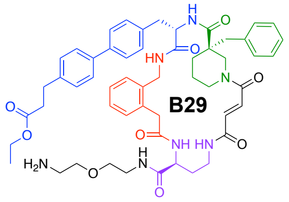

B29: Compound was synthesized using **Generalized procedure A for Suzuki-Miyaura cross-coupling of macrocycles** using **(4Br)B6** and 4-(2-ethoxycarbonylethyl)phenylboronic acid. Yield: 29%

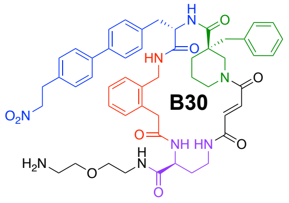

B30: Compound was synthesized using **Generalized procedure A for Suzuki-Miyaura cross-coupling of macrocycles** using **(4Br)B6** and 4-(2-nitroethyl)phenylboronic acid. Yield: 18%

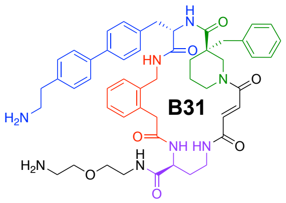

B31: Compound was synthesized using **Generalized procedure A for Suzuki-Miyaura cross-coupling of macrocycles** using **(4Br)B6** and (4-(2-[(tert-Butoxycarbonyl)amino]ethyl)phenyl)boronic acid**.** Prior to HPLC purification, crude reaction was concentrated under vacuum and treated with 0.3 mL TFA for 1 hour for boc-deprotection. Reaction was concentrated and purified as stipulated in generalized procedure. Yield: 48%

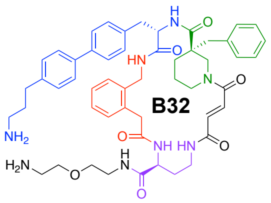

B32: Compound was synthesized using **Generalized procedure A for Suzuki-Miyaura cross-coupling of macrocycles** using **(4Br)B6** and [4-(3-{[(tert-Butoxy)carbonyl]amino}propyl)phenyl]boronic acid. Prior to HPLC purification, crude reaction was concentrated under vaccum and treated with 0.3 mL TFA for 1 hour for boc deprotection. Reaction was concentrated and purified as stipulated in generalized procedure. Yield: 20%

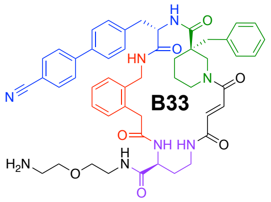

B33: **Compound was synthesized using** Generalized procedure A for Suzuki-Miyaura cross-coupling of macrocycles using **(4Br)B6** and 4-cyanophenylboronic acid. Yield: 30%

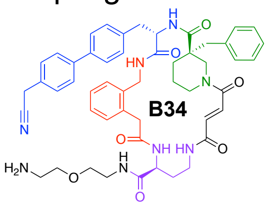

B34: Compound was synthesized using **Generalized procedure A for Suzuki-Miyaura cross-coupling of macrocycles** using **(4Br)B6** and 4-cyanomethylphenylboronic acid. Yield: 46%

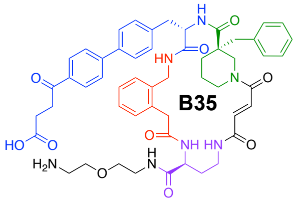

B35: **Compound was synthesized using** Generalized procedure A for Suzuki-Miyaura cross-coupling of macrocycles using **(4Br)B6** and **I7b**. Yield: 6%

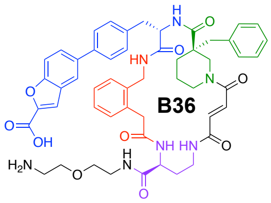

B36: Compound was synthesized using **Generalized procedure A for Suzuki-Miyaura cross-coupling of macrocycles** using **(4Br)B6** and **I8b**. Yield: 12%

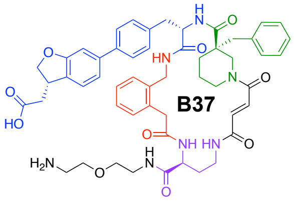

B37: Compound was synthesized using **Generalized procedure A for Suzuki-Miyaura cross-coupling of macrocycles** using **(4Br)B6** and **I9d**. Yield: 25%

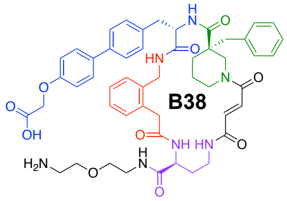

B38: Compound was synthesized using **Generalized procedure A for Suzuki-Miyaura cross-coupling of macrocycles** using **(4Br)B6** and 4-(carboxymethoxy)phenylboronic acid. Yield: 9%

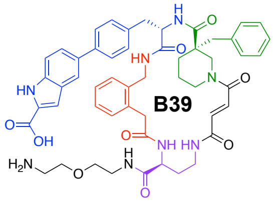

B39: Compound was synthesized using **Generalized procedure B for Suzuki-Miyaura cross-coupling of macrocycles** using **(4Br)B6** and **I10**. Yield: 5%

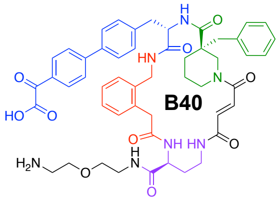

B40: Adpoting a previously reported protocol[9], into a 10 mL screw-top vial, **(4Br)B6** (0.011 mmol, 1.0 eq), **I11** (03_263) (0.11 mmol, 10 eq), Bis(dibenzylideneacetone)palladium(0) (0.0011 mmol, 0.1 eq), Tri-*tert*-butylphosphonium tetrafluoroborate (0.0046 mmol, 0.4 eq),and potassium carbonate (0.11 mmol, 10 eq) were combined and sealed with a septum. Flask was evacuated and refilled with N_2 three times and left under N_2. Degassed 10:1 Tolune/H_2O (0.05 M), was then added to the sealed flask and allowed to stir at 60° C for 16 hours. After cooling, the reaction was cooled and solvent was removed under high-vac. Solid residue was dissolved in a minimal amount of 3:1 DMF/water with 5% TFA and filtered through 0.22 μL Ultrafree®-MC Centrifugal Filters (Milipore-Sigma). Sample was then purified by reverse HPLC, with a 10-60% Fractions that contained product was then freeze dried to produce a white powder. Yield: 10% (03_265)

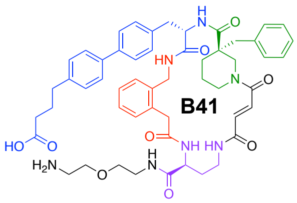

B41: Compound was synthesized using **Generalized procedure A for Suzuki-Miyaura cross-coupling of macrocycles** using **(4Br)B6** and **I12b**. Yield: 14%

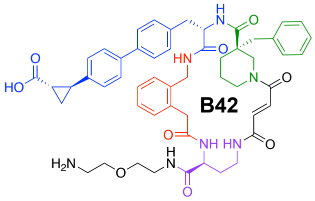

B42: Compound was synthesized using **Generalized procedure B for Suzuki-Miyaura cross-coupling of macrocycles** using **(4Br)B6** and **I13b** . Yield: 31%

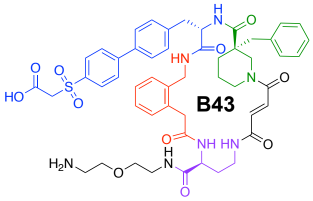

B43: Compound was synthesized using **Generalized procedure A for Suzuki-Miyaura cross-coupling of macrocycles** using **(4Br)B6** and **I14c**. *Tert*-butyl protected product was treated with 1 mL TFA for 1 hour, and re-purified under same HPLC conditions to yield carboxylic acid product. Yield: 11%

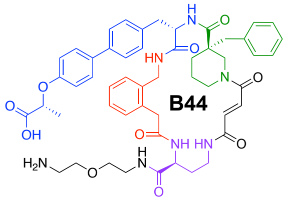

B44: Compound was synthesized using **Generalized procedure A for Suzuki-Miyaura cross-coupling of macrocycles** using **(4Br)B6** and **I15d**. Yield: 8%

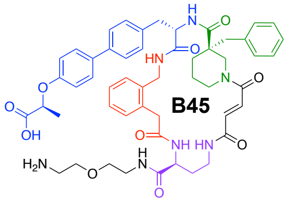

B45: Compound was synthesized using **Generalized procedure A for Suzuki-Miyaura cross-coupling of macrocycles** using **(4Br)B6** and **I16b**. Yield: 16%

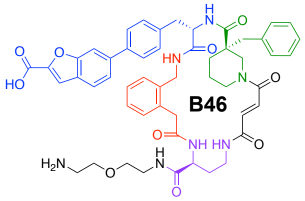

B46: Compound was synthesized using **Generalized procedure B for Suzuki-Miyaura cross-coupling of macrocycles** using **(4Br)B6** and **I17d**. Yield: 4%

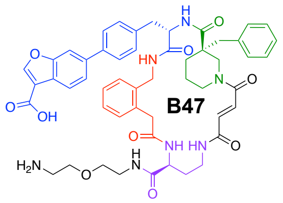

B47: Compound was synthesized using **Generalized procedure B for Suzuki-Miyaura cross-coupling of macrocycles** using **(4Br)B6** and **I18c**. Yield: 8%

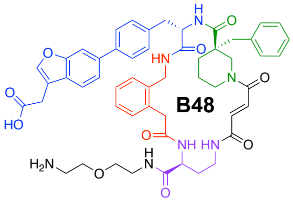

B48: Compound was synthesized using **Generalized procedure A for Suzuki-Miyaura cross-coupling of macrocycles** using **(4Br)B6** and **I19e**. Yield: 5%

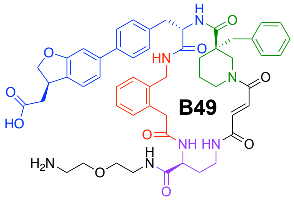

B49: Compound was synthesized using **Generalized procedure A for Suzuki-Miyaura cross-coupling of macrocycles** using **(4Br)B6** and **I20d**. Yield: 5%

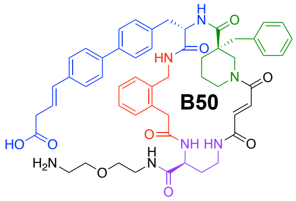

B50: Compound was synthesized using **Generalized procedure B for Suzuki-Miyaura cross-coupling of macrocycles** using **(4Br)B6** and **I21b**. Yield: 8%

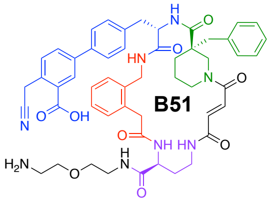

B51: Compound was synthesized using **Generalized procedure B for Suzuki-Miyaura cross-coupling of macrocycles** using **(4Br)B6** and **I22c**. Yield: 18%

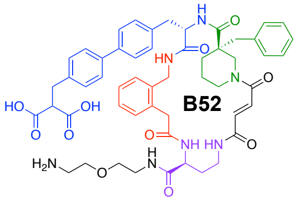

B52: Compound was synthesized using **Generalized procedure A for Suzuki-Miyaura cross-coupling of macrocycles** using **(4Br)B6** and **I23**. *Tert*-butyl protected product was treated with 1 mL TFA for 1 hour, and re-purified under same HPLC conditions to yield carboxylic acid product. Yield: 10%

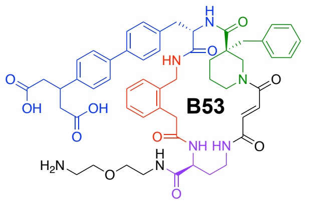

B53: Compound was synthesized using **Generalized procedure A for Suzuki-Miyaura cross-coupling of macrocycles** using **(4Br)B6** and **I24c**. *Tert*-butyl protected product was treated with 1 mL TFA for 1 hour, and re-purified under same HPLC conditions to yield carboxylic acid product. Yield: 21%

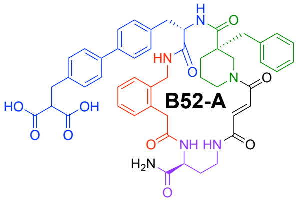

B52-A: Compound was synthesized using **Generalized procedure A for Suzuki-Miyaura cross-coupling of macrocycles** using **(4Br)B6-A** and **I23**. *Tert*-butyl protected product was treated with 1 mL TFA for 1 hour, and re-purified under same HPLC conditions to yield carboxylic acid product. Yield: 40%

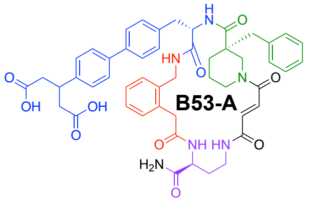

B53-A: Compound was synthesized using **Generalized procedure A for Suzuki-Miyaura cross-coupling of macrocycles** using **(4Br)B6-A** and **I24c**. *Tert*-butyl protected product was treated with 1 mL TFA for 1 hour, and re-purified under same HPLC conditions to yield carboxylic acid product. Yield: 86%

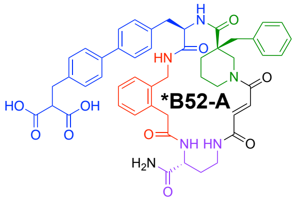

B52-A:** Compound was synthesized using **Generalized procedure A for Suzuki-Miyaura cross-coupling of macrocycles** using **(4Br)B6-A** and **I23**. *Tert*-butyl protected product was treated with 1 mL TFA for 1 hour, and re-purified under same HPLC conditions to yield carboxylic acid product. Yield: 21%.

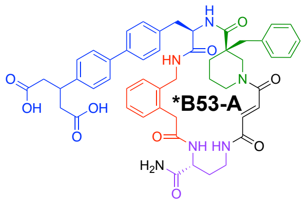

B53-A:** Compound was synthesized using **Generalized procedure A for Suzuki-Miyaura cross-coupling of macrocycles** using **(4Br)B6-A** and **I24c**. *Tert*-butyl protected product was treated with 1 mL TFA for 1 hour, and re-purified under same HPLC conditions to yield carboxylic acid product. Yield: 85%.

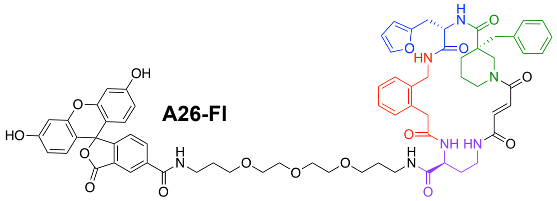

A26-Fl: Macrocycle was assembled using **general procedure for solid-phase peptide synthesis of macrocycle inhibitors**. Prior to acidic cleavage, resin was suspended with three 1-hour treatments of 1M hydroxybenzotriazole monohydrate dissolved in 1:1 CH_2Cl_2/Trifluoroethanol to remove Mmt group. 5-carboxyfluorescein (5 eq) and (HATU, 4.75 equiv.) were dissolved in a separate flask in ~10-15 mL DMF then treated with *N,N'*-diisopropylethylamine (DIPEA, 10 eq) for 5 minutes, observing a color change. This flask was then transferred to resin swollen in ~10 mL DMF and allowed to mix for 24 hours. Fluorescein labeled peptide were then cleaved from resin and purified as described in general procedure.
Resin: Universal PEG NovaTag^{TM}_Resin
4th Building Block: N$^{\alpha}$-Fmoc-N$^{\gamma}$-allyloxycarbonyl-L-2,4-diaminobutyric acid
1st Building Block**:** Fmoc-2-aminomethyl-phenylacetic acid
2nd Building Block: Fmoc-β-(2-furyl)-Ala-OH
3rd Building Block: (S)-Fmoc-3-benzyl-piperidine-3-carboxylic acid

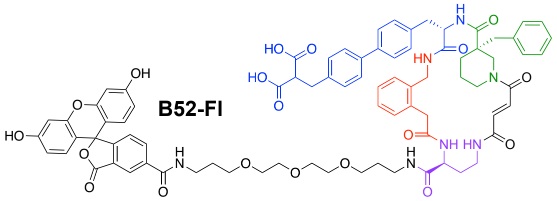

B52-Fl: Compound was synthesized using **Generalized procedure A for Suzuki-Miyaura cross-coupling of macrocycles** using **(4Br)B6-Fl** and **I23**. *Tert*-butyl protected product was treated with 1 mL TFA for 1 hour, and re-purified under same HPLC conditions to yield carboxylic acid product. Yield: 32%.

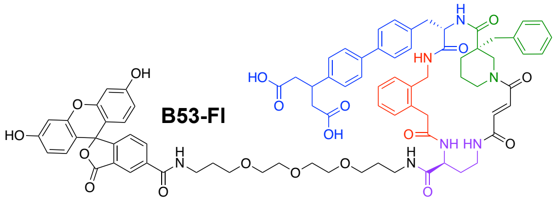

B53-Fl: Compound was synthesized using **Generalized procedure A for Suzuki-Miyaura cross-coupling of macrocycles** using **(4Br)B6-Fl** and **I24c**. *Tert*-butyl protected product was treated with 1 mL TFA for 1 hour, and re-purified under same HPLC conditions to yield carboxylic acid product. Yield: 33%

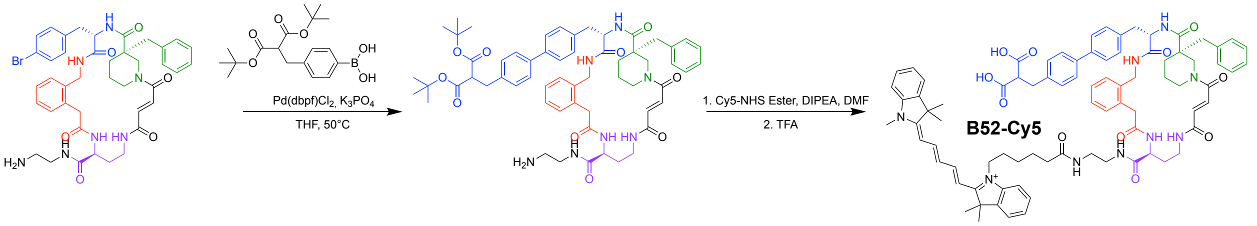

B52-Cy5: Compound was synthesized using **Generalized procedure A for Suzuki-Miyaura cross-coupling of macrocycles** using **(4Br)B6-B** and **I23**. *Tert*-butyl protected product was dissolved in a 1.5 mL Eppendorf tube with 100 uL DMF and mixed with DIPEA (5 eq.). Cy5-NHS ester (Lumiprobe) (2 eq.) dissolved in 50 uL DMF was then added to the flask allowed to stir for 1 hour. Resulting crude mixture was purified under same HPLC conditions to yield Cy5 coupled product. This product was then treated with 1 mL TFA for 1 hour, and re-purified under same HPLC conditions to yield carboxylic acid product. Yield: 25%.

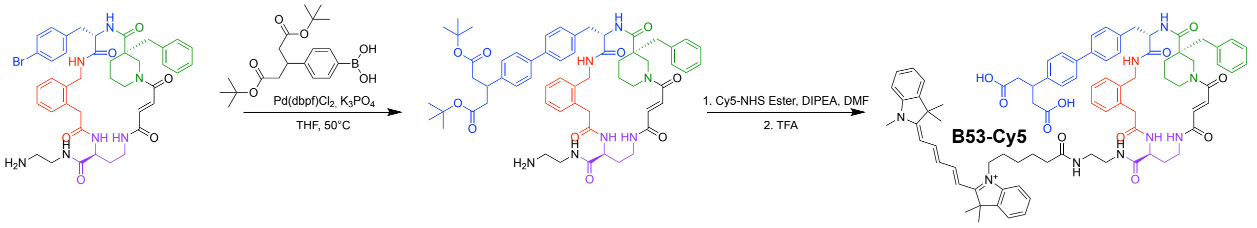

B53-Cy5: Compound was synthesized using **Generalized procedure A for Suzuki-Miyaura cross-coupling of macrocycles** using **(4Br)B6-B** and **I24c**. *Tert*-butyl protected product was dissolved in a 1.5 mL Eppendorf tube with 100 uL DMF and mixed with DIPEA (5 eq.). Cy5-NHS ester (Lumiprobe) (2 eq.) dissolved in 50 uL DMF was then added to the flask allowed to stir for 1 hour. Resulting crude mixture was purified under same HPLC conditions to yield Cy5 coupled product. This product was then treated with 1 mL TFA for 1 hour, and re-purified under same HPLC conditions to yield carboxylic acid product. Yield: 6%.

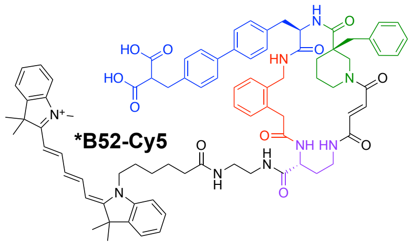

***B52-Cy5:** Compound was synthesized as described in **B52-Cy5** using ***(4Br)B6-B** as starting material. Yield: 6%

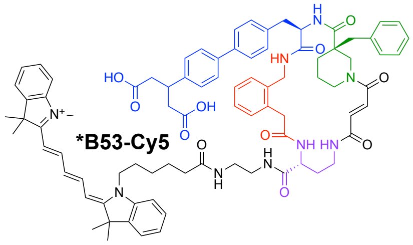

***B53-Cy5:** Compound was synthesized as described in **B53-Cy5** using ***(4Br)B6-B** as starting material. Yield: 8%.

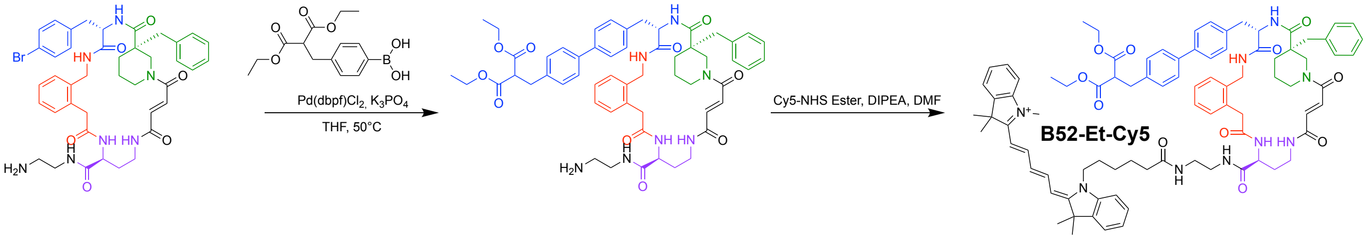

B52-Et-Cy5: Compound was synthesized using **Generalized procedure A for Suzuki-Miyaura cross-coupling of macrocycles** using **(4Br)B6-B** and **I29b**. Suzuki product was dissolved in a 1.5 mL Eppendorf tube with 100 uL DMF and mixed with DIPEA (5 eq.). Cy5-NHS ester (Lumiprobe) (2 eq.) dissolved in 50 uL DMF was then added to the flask allowed to stir for 1 hour. Excess Cy5-NHS ester was quenched with N-Acetylethylenediamine (10 eq.), to make **Cy5-enAc.** Resulting crude mixture was purified under same HPLC conditions to yield the two Cy5 coupled products. Yield: 20%

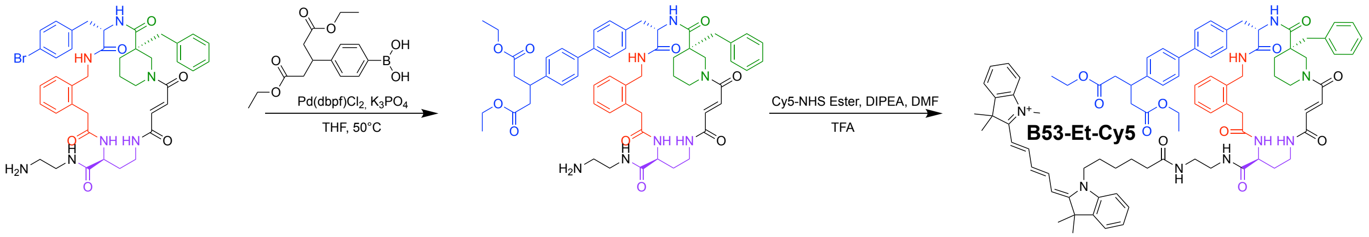

B53-Et-Cy5: Compound was synthesized using **Generalized procedure A for Suzuki-Miyaura cross-coupling of macrocycles** using **(4Br)B6-B** and **I30c**. Suzuki product was dissolved in a 1.5 mL Eppendorf tube with 100 uL DMF and mixed with DIPEA (5 eq.). Cy5-NHS ester (Lumiprobe) (2 eq.) dissolved in 50 uL DMF was then added to the flask allowed to stir for 1 hour. Excess Cy5-NHS ester was quenched with N-Acetylethylenediamine (10 eq.), to make **Cy5-enAc.** Resulting crude mixture was purified under same HPLC conditions to yield the two Cy5 coupled products. Yield: 21%

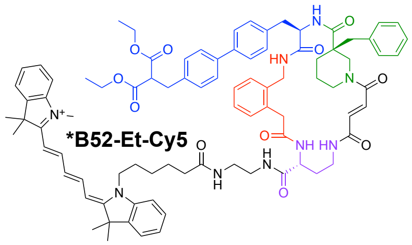

***B52-Et-Cy5:** Compound was synthesized as described in **B52-Et-Cy5** using ***(4Br)B6-B** as starting material.
Yield: 18%

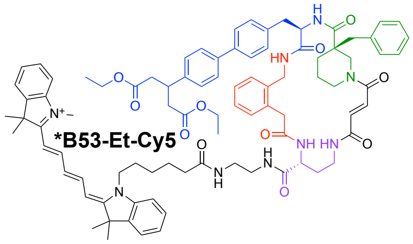

***B53-Et-Cy5:** Compound was synthesized as described in **B53-Et-Cy5** using ***(4Br)B6-B** as starting material.
Yield: 17%.

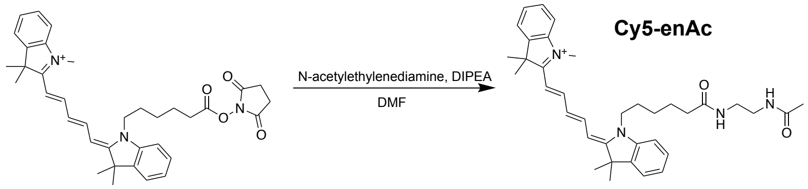

Cy5-enAc: Compound was isolated from excess Cy5-NHS ester that was quenched with N-Acetylethylenediamine (10 eq.) during syntheses of **B52-Et-Cy5** and **B53-Et-Cy5.** HPLC purification was able to separate **Cy5-enAc** successfully from of **B52-Et-Cy5** and **B53-Et-Cy5.**

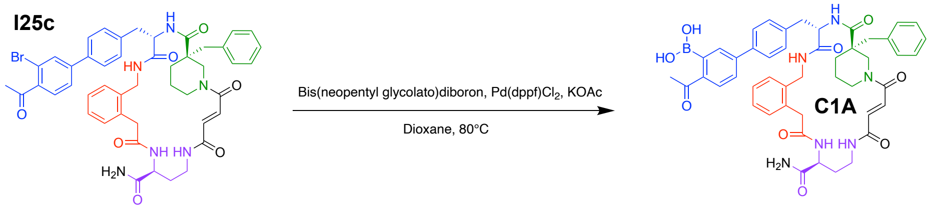

C1A: A mixture of **I25c** (0.008 mmol, 1 eq.), bis (neopentyl glycolato) diboron (0.08 mmol, 10 eq.), [1,1-Bis(diphenylphosphino)ferrocene]dichloropalladium(**II**) (0.001 mmol, 0.2 eq.), and KOAc (0.08 mmol, 10 eq.) was dissolved in 1 mL 1,4-dioxane (0.008 M). The reaction was stirred for thirty minutes at 80°C. The reaction was then cooled down to room temperature, solvent removed under vacuum, dissolved in DMF, and filtered. The crude product was purified by reverse-phase HPLC using 10-80% acetonitrile in water in 0.1% trifluoroacetic acid and lyophilized to afford **C1A**. Yield: 20% yield

<u>Authors Note: Bis(neopentyl glyolato) and the shorter 30-minute reaction time was due to significant protodeborylation observed with bis(pinacolato)diboron and longer reaction times.</u>

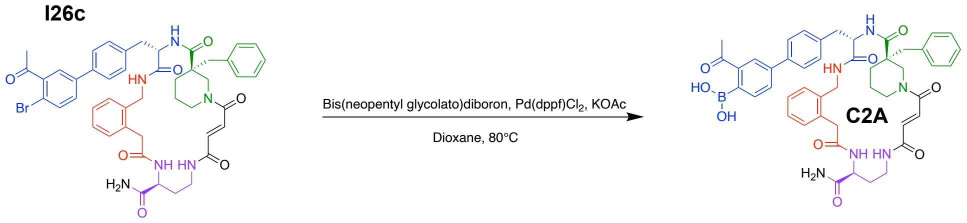

C2A: Compound was synthesized and purified as described in **C1A** with **I26c,** affording **C2A**. Yield: 29%

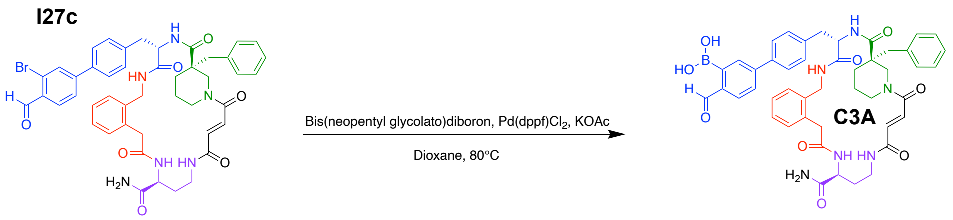

C3A: Compound was synthesized and purified as described in **C1A** with **I27c**, affording **C3A**. Yield: 14%

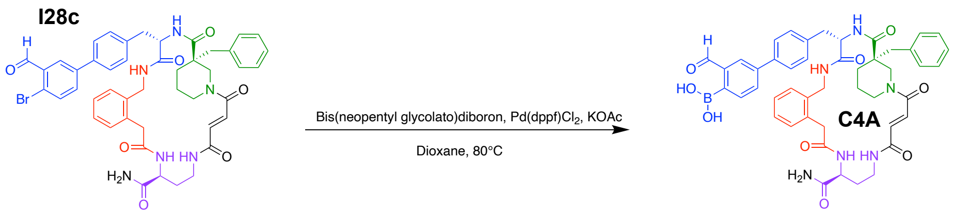

C4A: Compound was synthesized and purified as described in **C1A** with **I28c**, affording **C4A**. Some impurities were observed by ^{1}H-NMR that were unable to be removed by HPLC, compound was tested without further purification. Yield: 4%

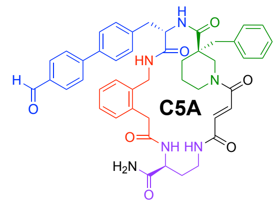

C5A: Compound was synthesized according to **Generalized procedure A for Suzuki-Miyaura cross-coupling of macrocycles** with **(4Br)B6-A** and 4-formylphenylboronic acid, affording **C5A**. Yield: 16%

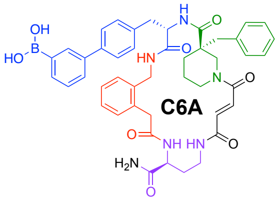

C6A: Compound was synthesized according to **Generalized procedure A for Suzuki-Miyaura cross-coupling of macrocycles** with **(4Br)B6-A** and benzene 1,3-diboronic acid, affording **C6A**. Yield: 13%

^{1}H-NMR Spectra

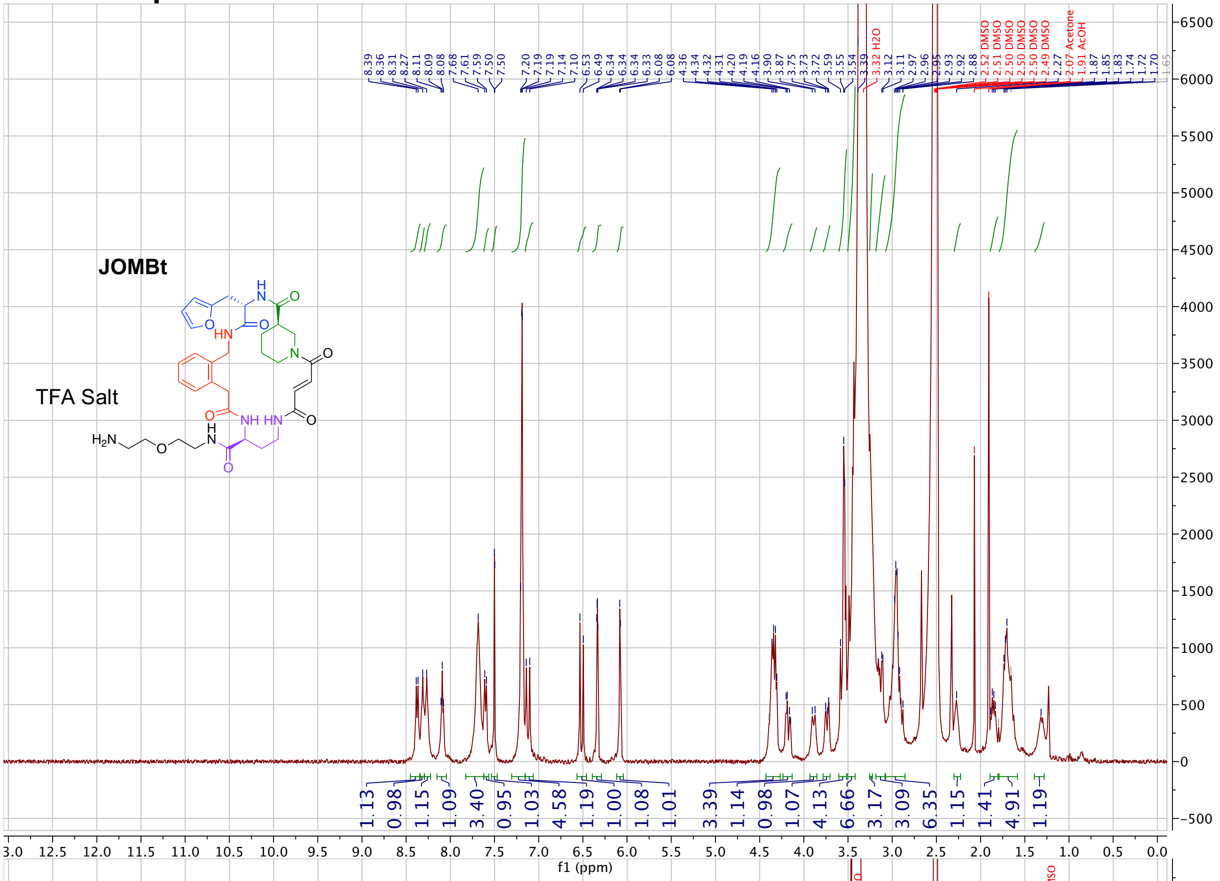

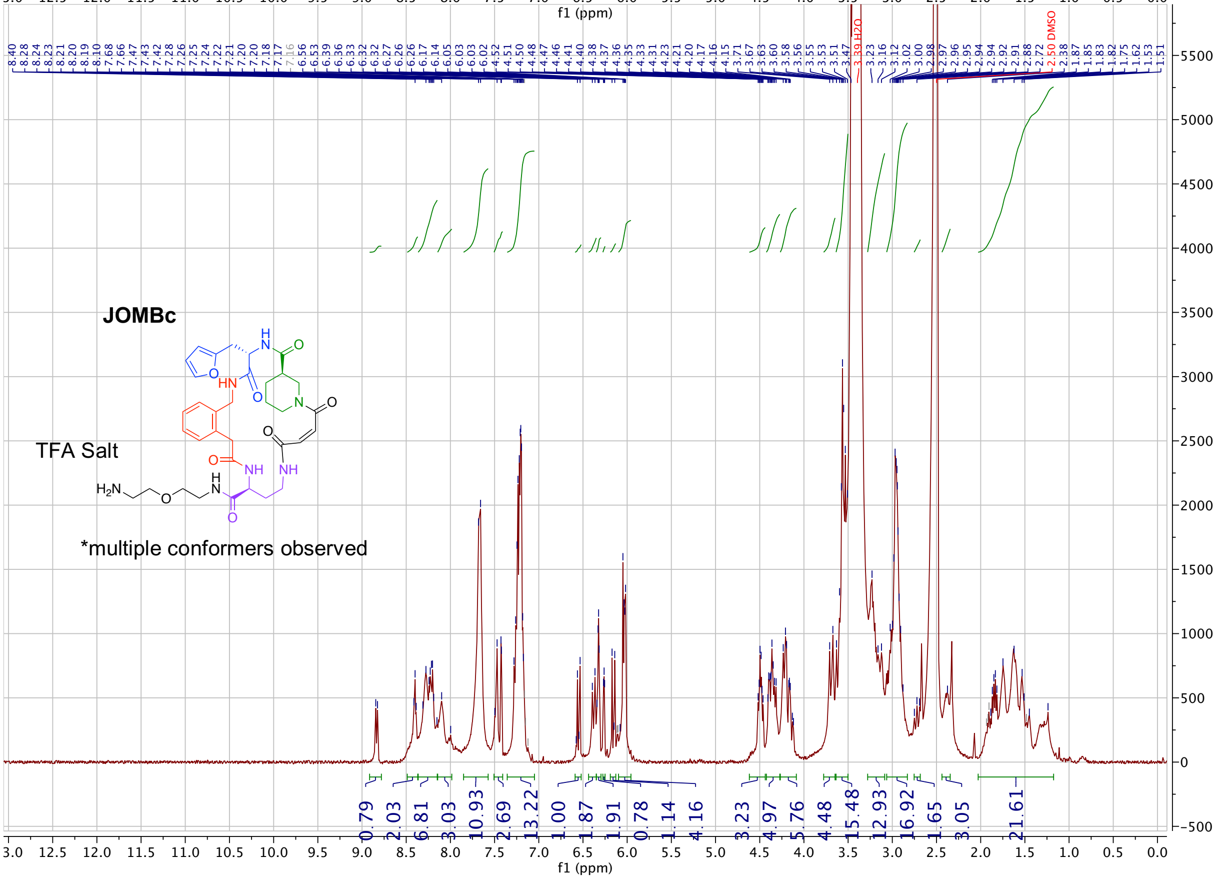

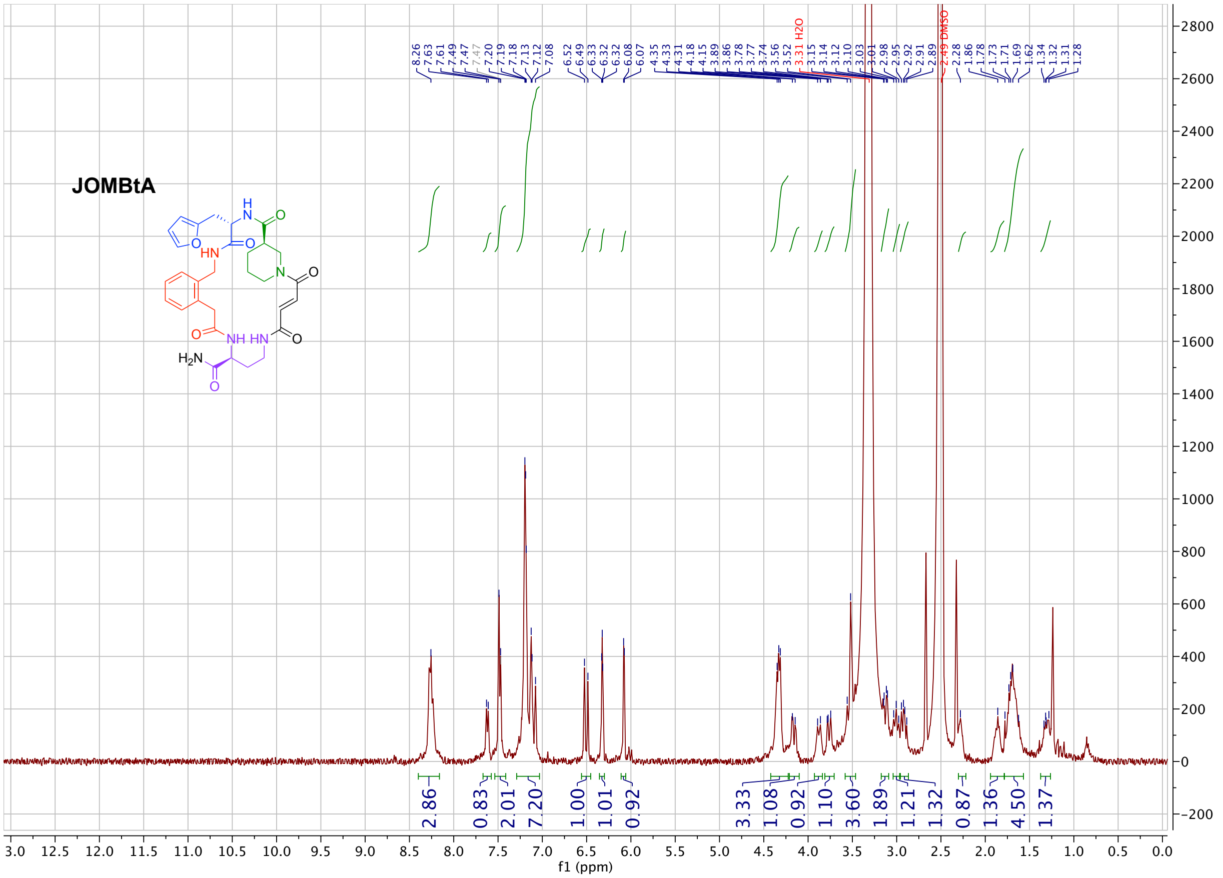
JOMBtA
f1 (ppm)
3.31 H2O
2.49 DMSO

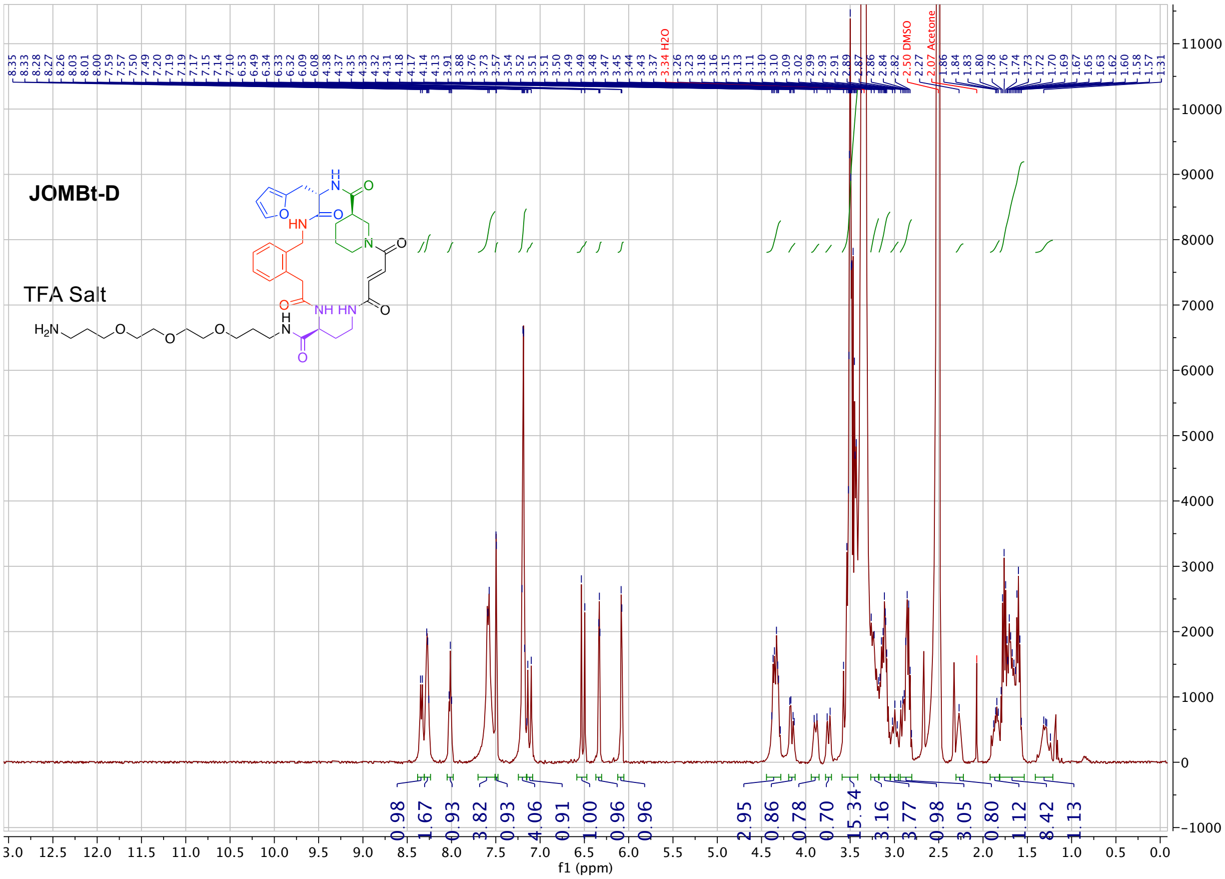
JOMBt-D
TFA Salt
f1 (ppm)
3.34 H2O
2.50 DMSO
2.07 Acetone

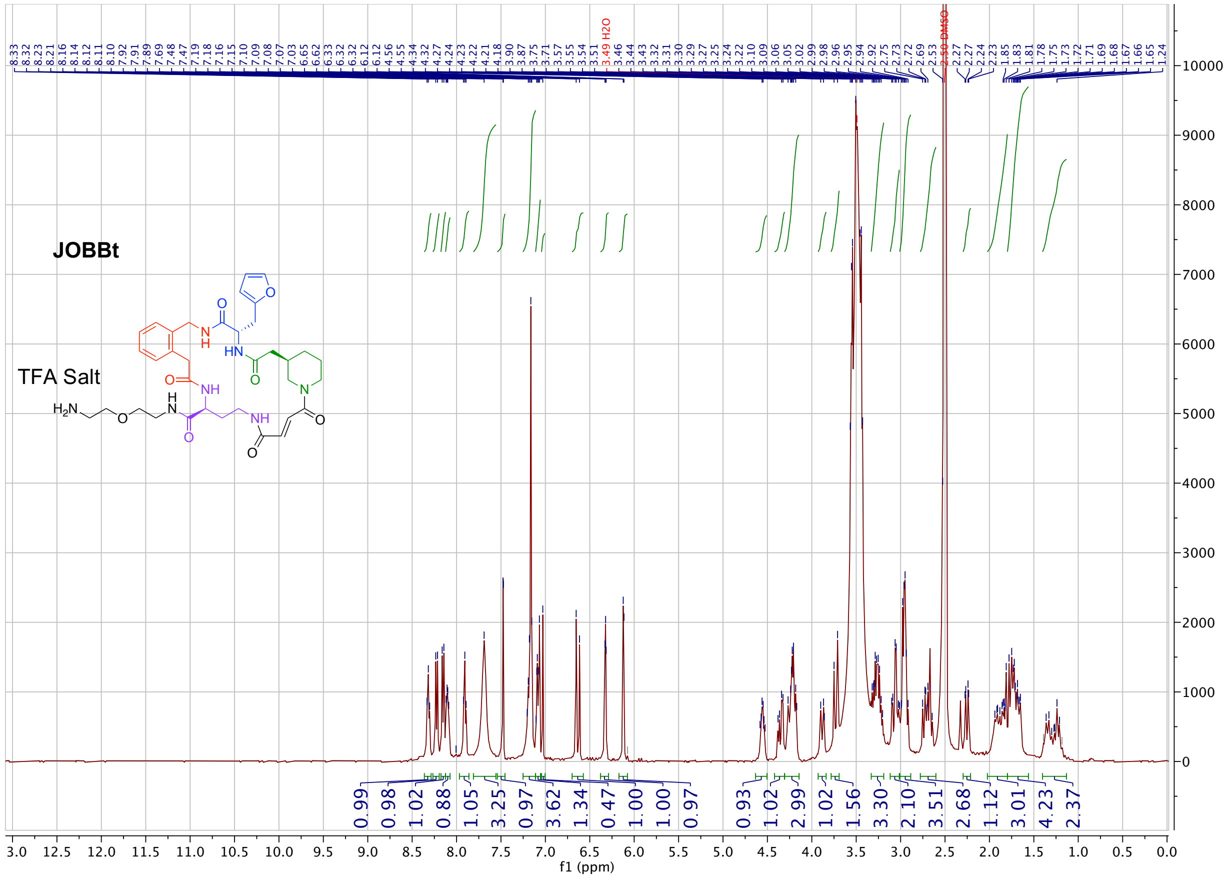
JOBBt
TFA Salt
3.49 H2O
2.50 DMSO
f1 (ppm)

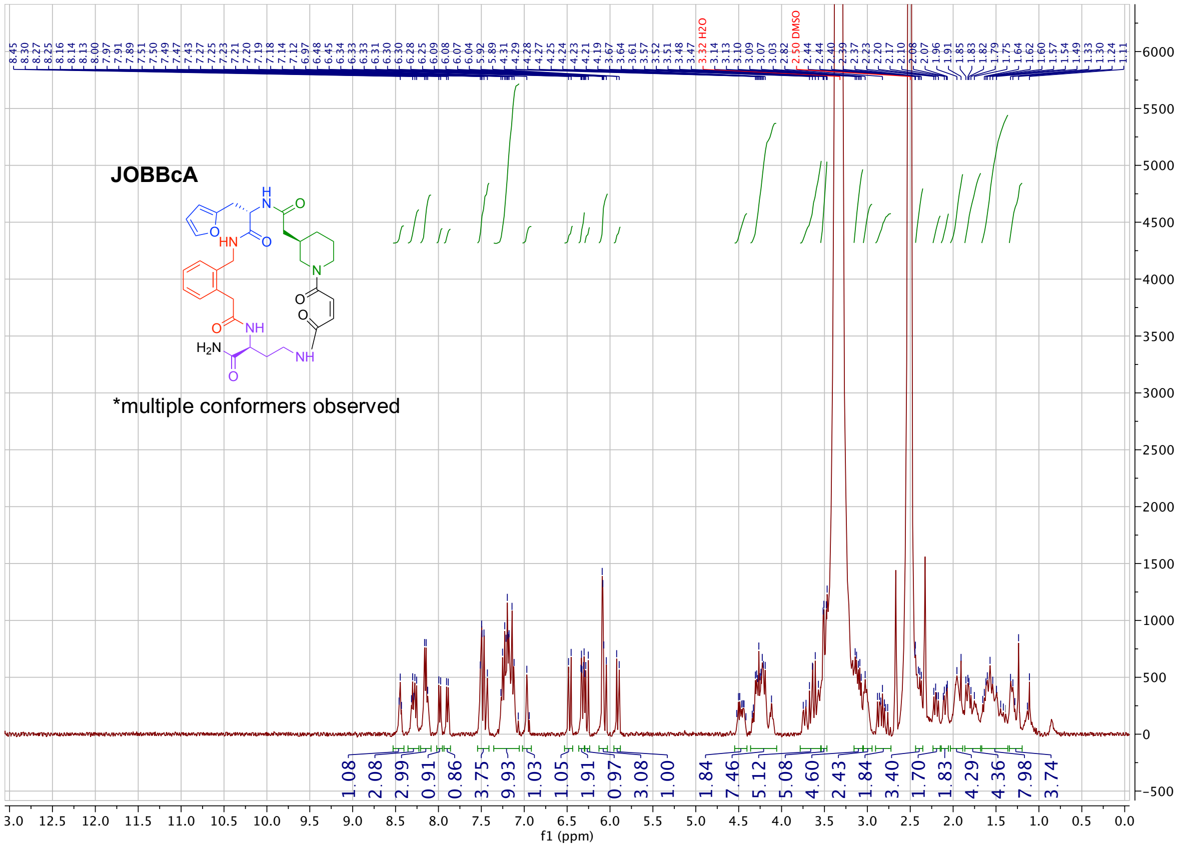
JOBBcA
*multiple conformers observed
3.32 H2O
2.50 DMSO
f1 (ppm)

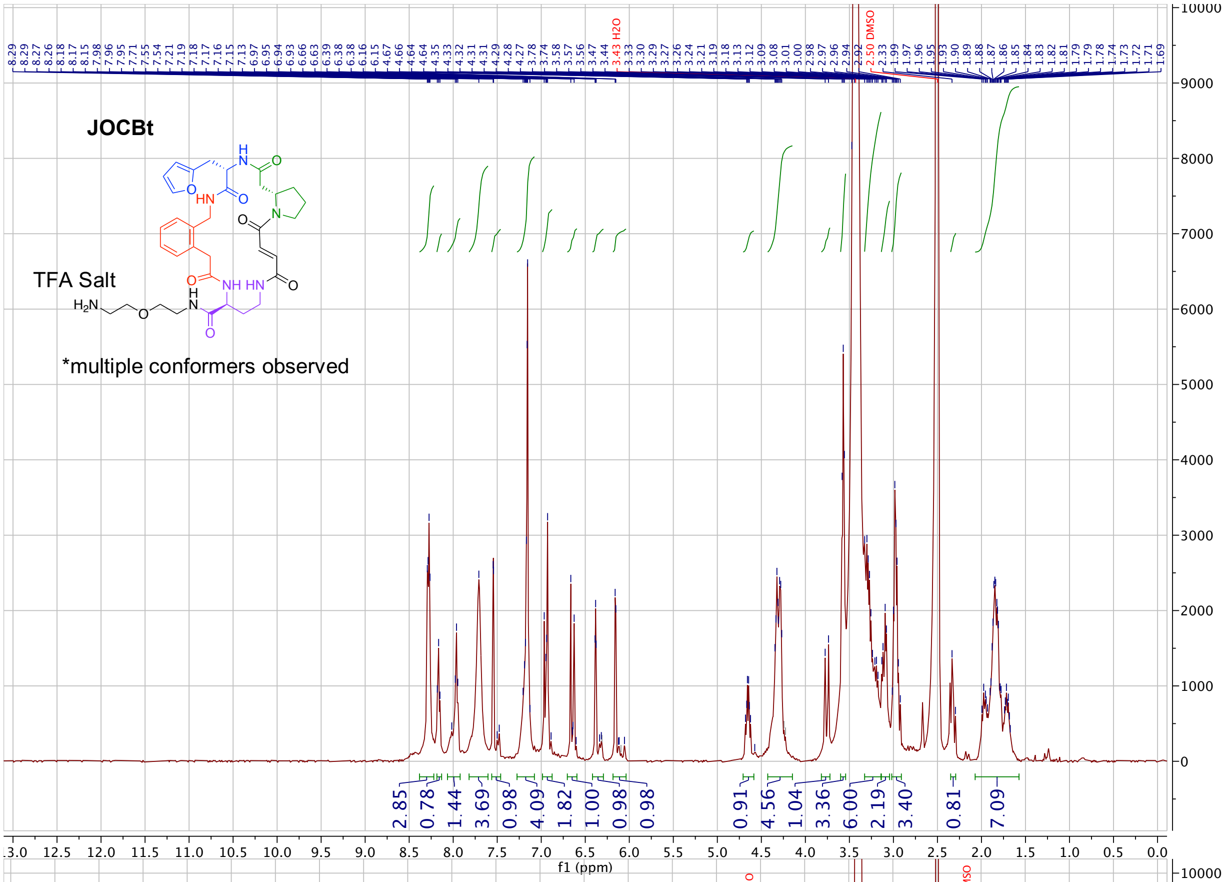
JOCBt
TFA Salt
*multiple conformers observed
f1 (ppm)

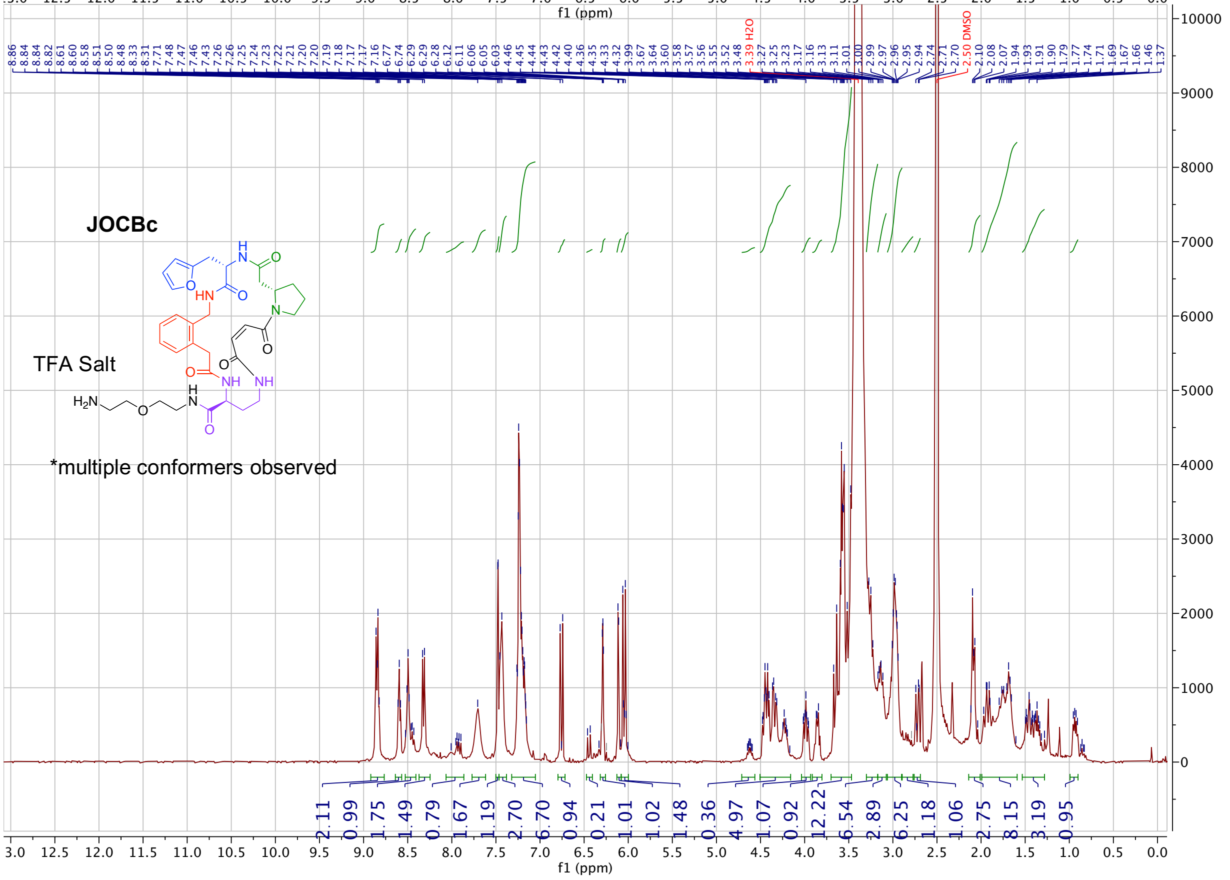
JOCBc
TFA Salt
*multiple conformers observed
f1 (ppm)

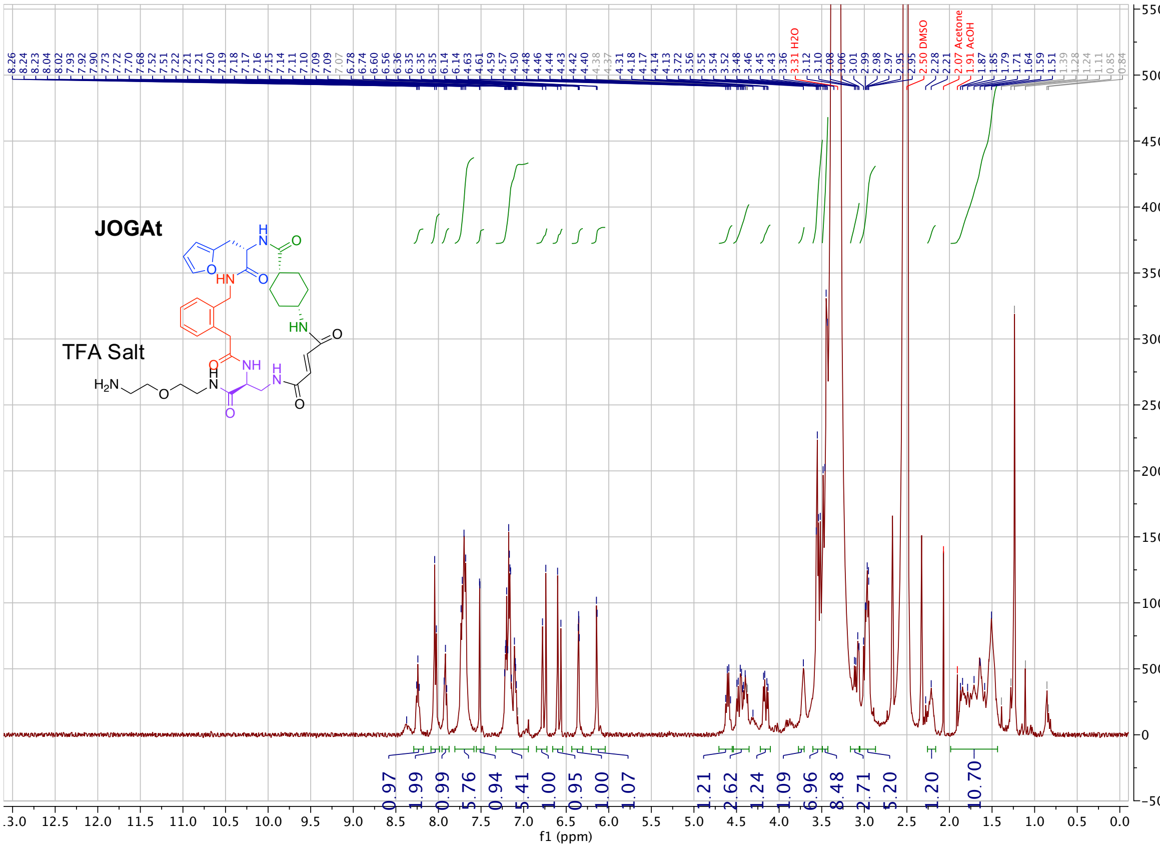
JOGAt
TFA Salt
f1 (ppm)
0.97
1.99
0.99
5.76
0.94
5.41
1.00
0.95
1.00
1.07
1.21
2.62
1.24
1.09
6.96
8.48
2.71
5.20
1.20
10.70
3.31 H2O
2.50 DMSO
2.07 Acetone
1.91 AcOH

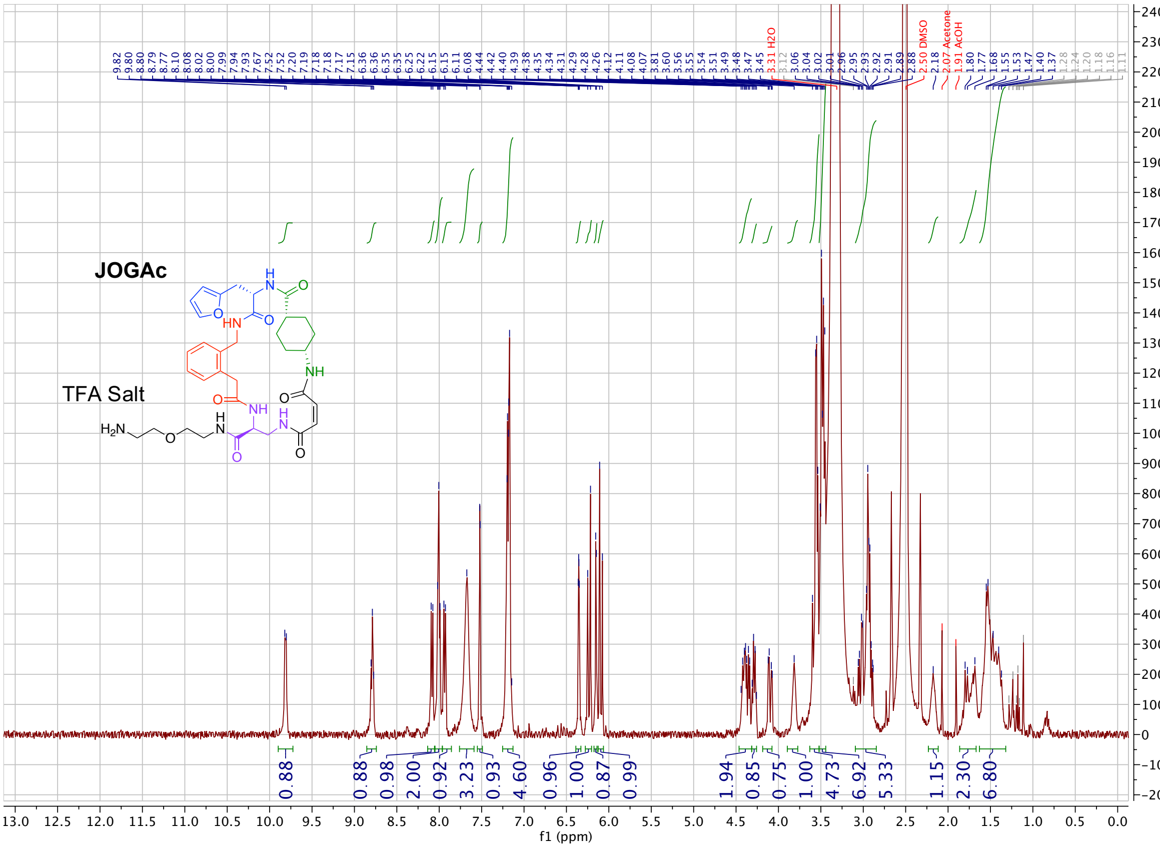
JOGAc
TFA Salt
f1 (ppm)
0.88
0.88
0.98
2.00
0.92
3.23
0.93
4.60
0.96
1.00
0.87
0.99
1.94
0.85
0.75
1.00
4.73
6.92
5.33
1.15
2.30
6.80
3.31 H2O
2.50 DMSO
2.07 Acetone
1.91 AcOH

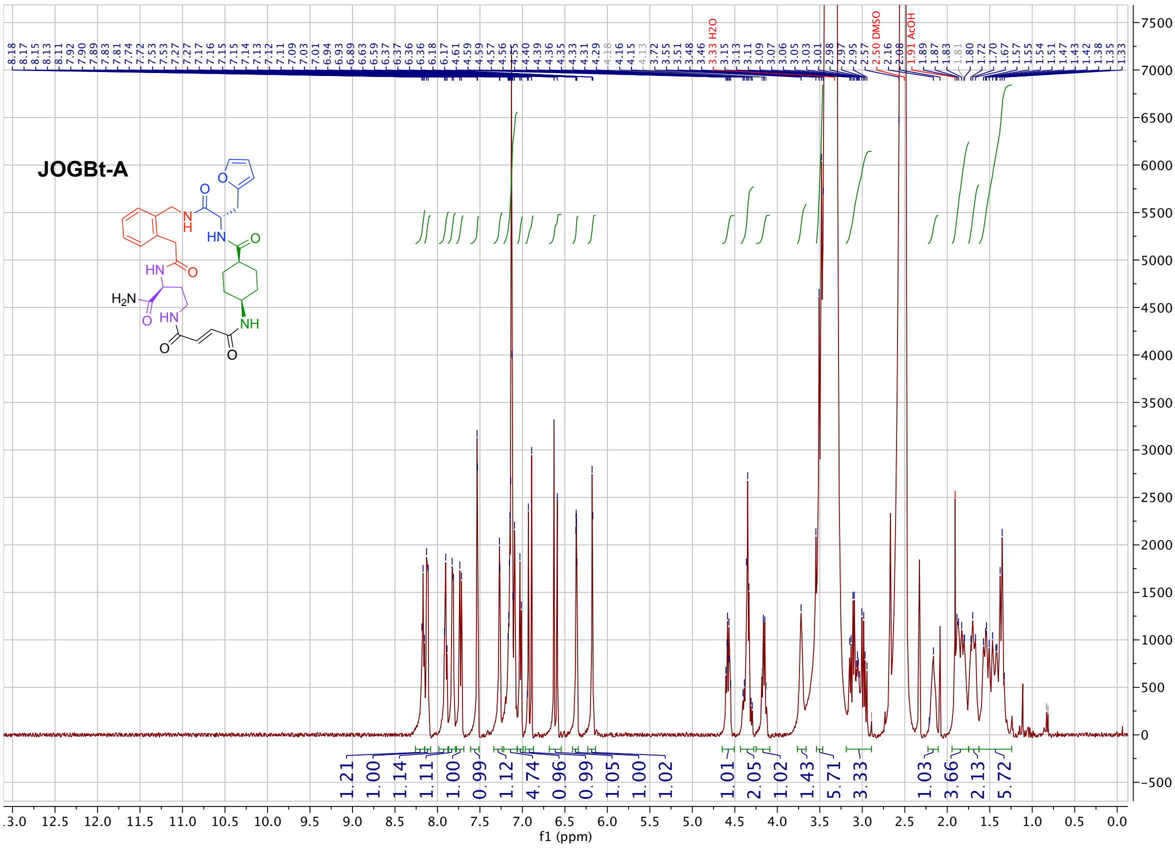
JOGBt-A
f1 (ppm)

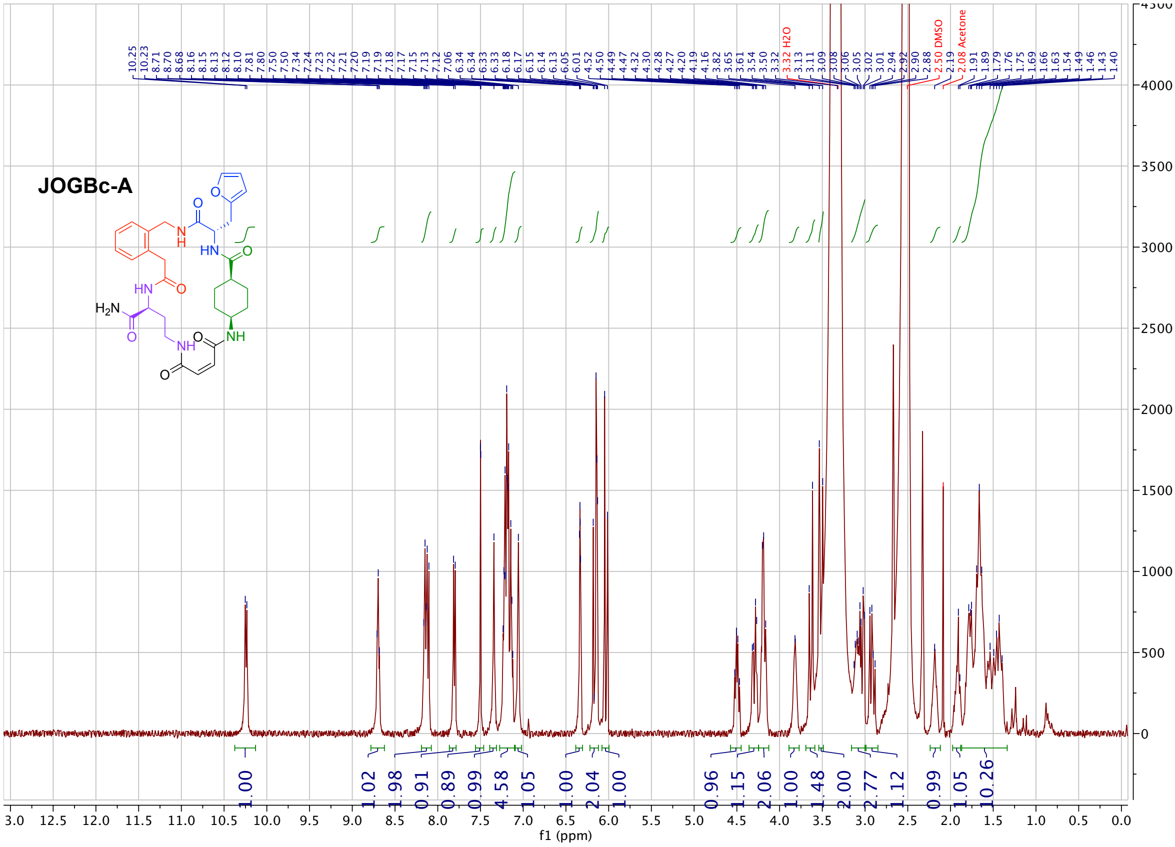
JOGBc-A
f1 (ppm)

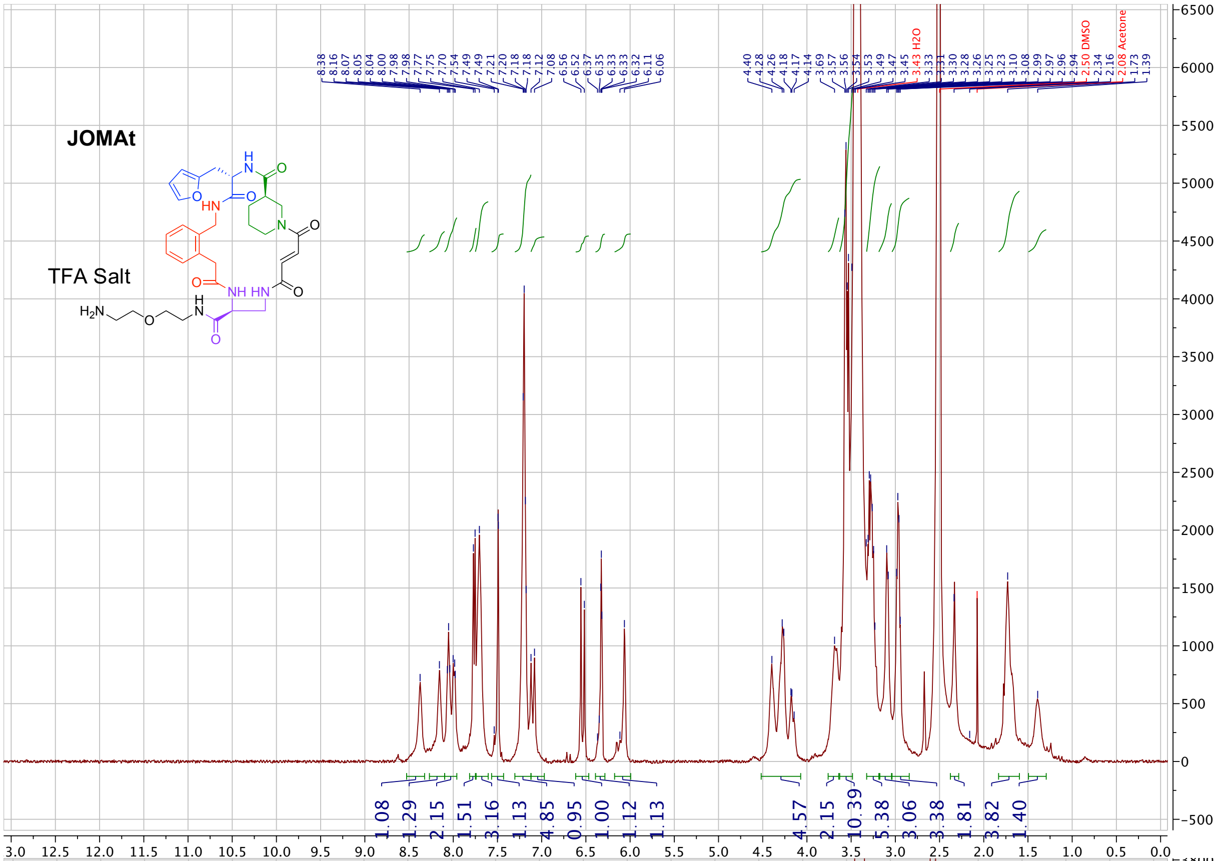
JOMAt
TFA Salt
f1 (ppm)

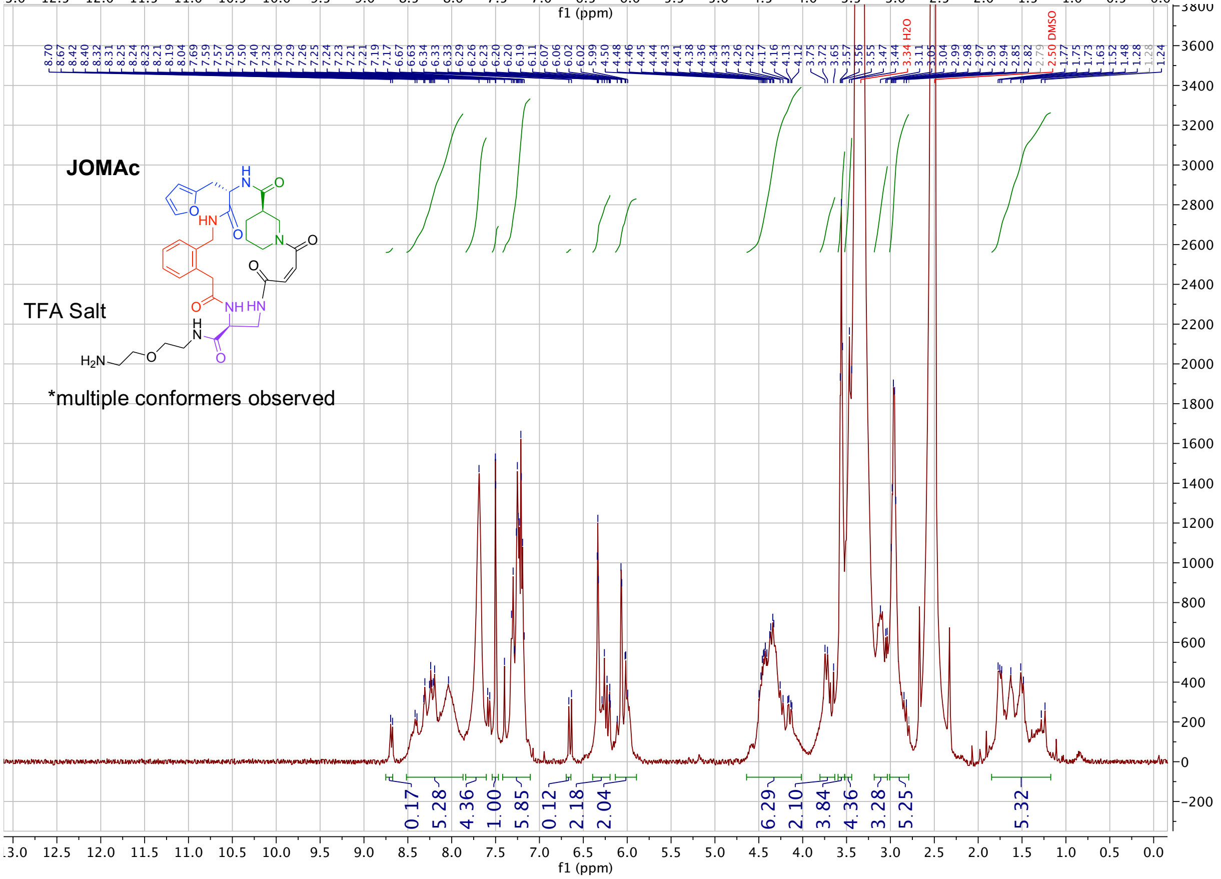
JOMAc
TFA Salt
*multiple conformers observed
f1 (ppm)

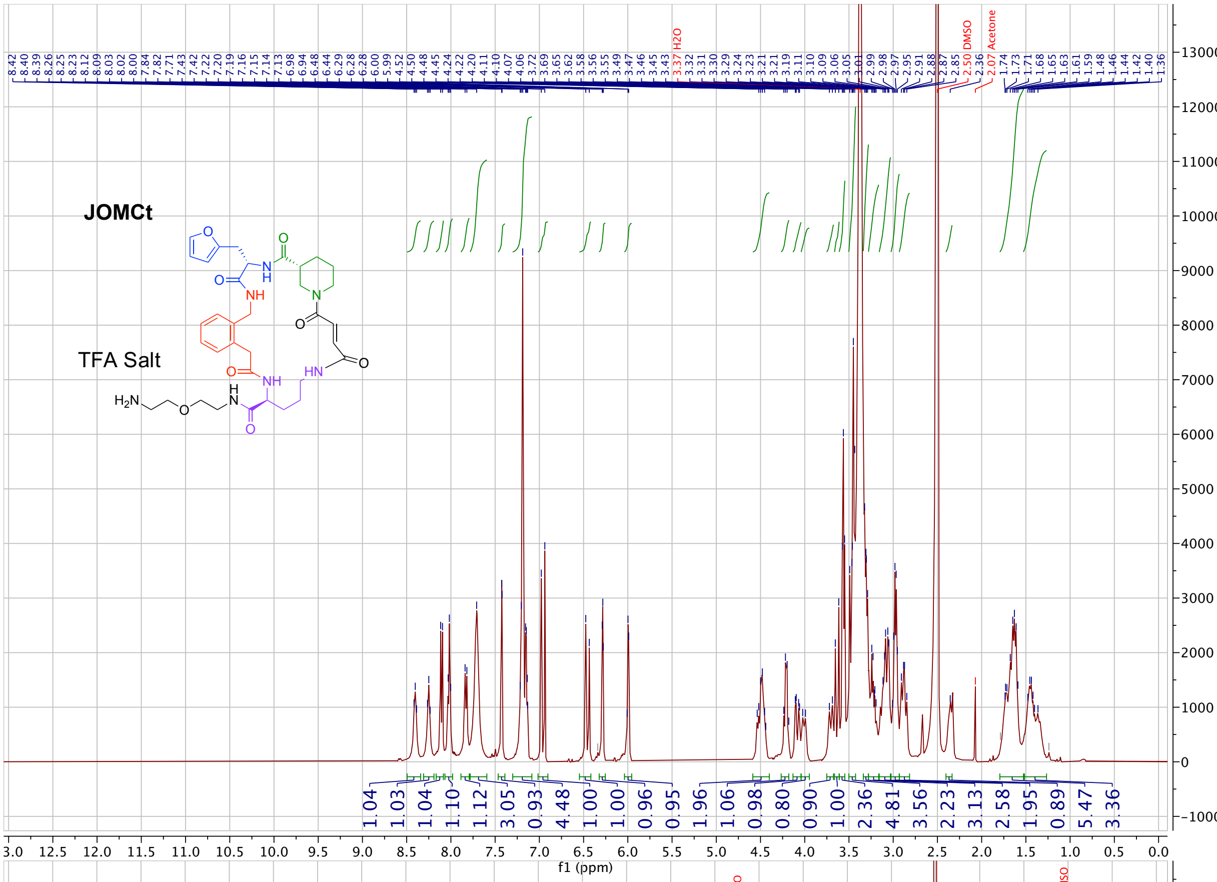
JOMCt
TFA Salt
f1 (ppm)

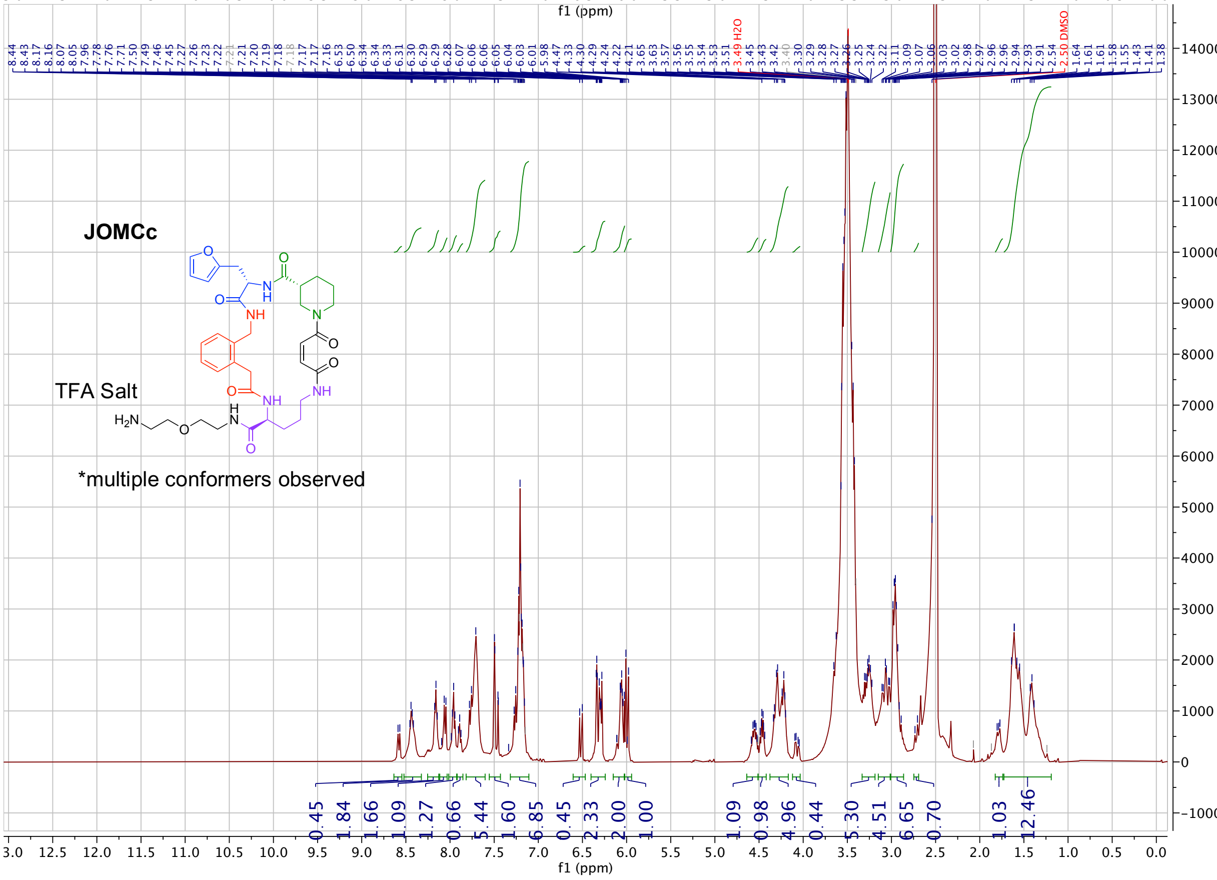
JOMCc
TFA Salt
*multiple conformers observed
f1 (ppm)

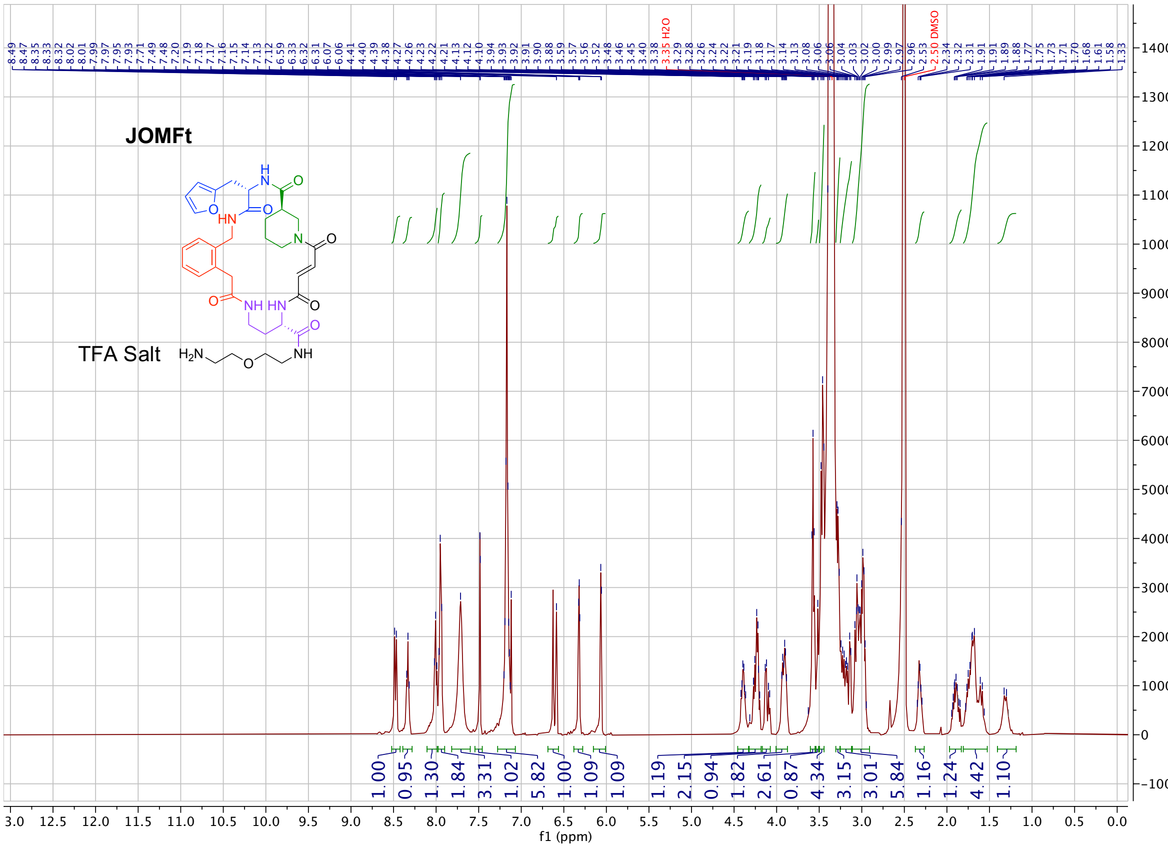
JOMFt
TFA Salt
*multiple conformers observed

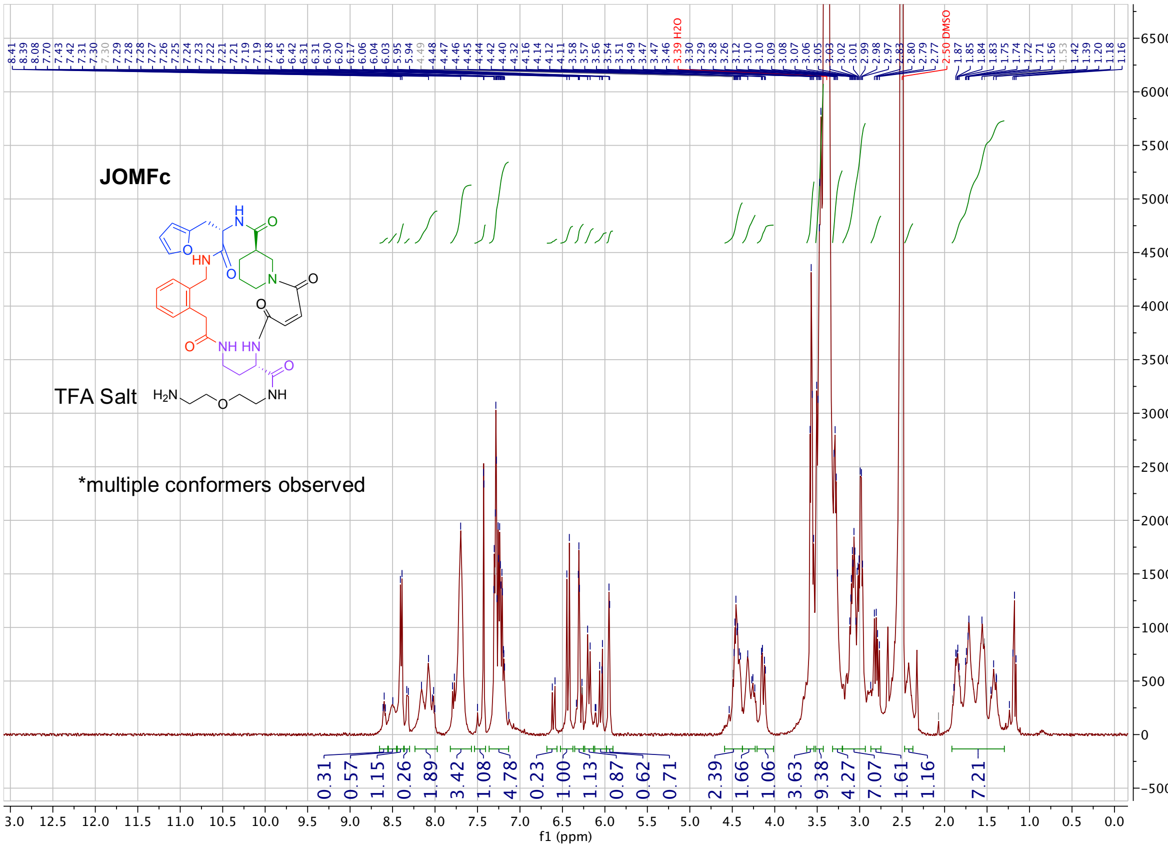
JOMFc
TFA Salt
*multiple conformers observed

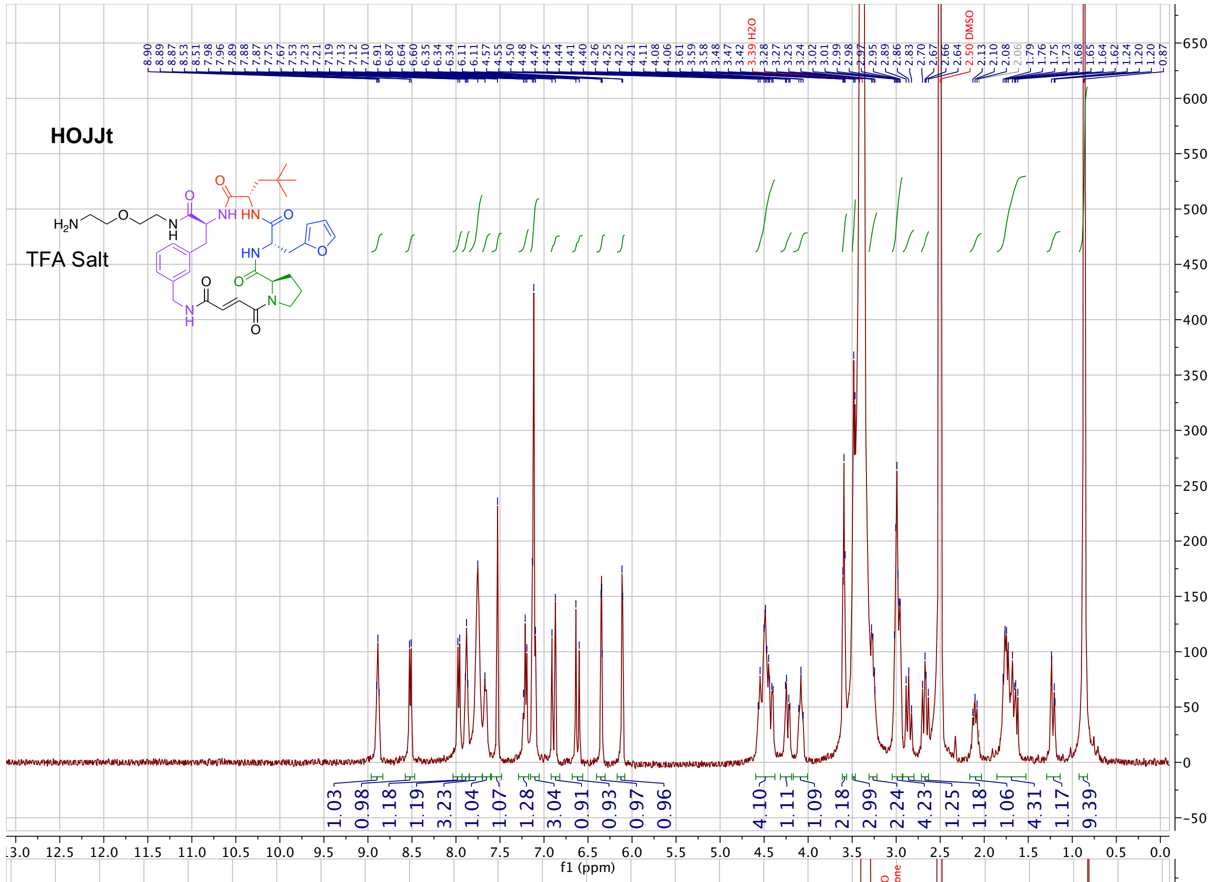
HOJJt
TFA Salt
f1 (ppm)

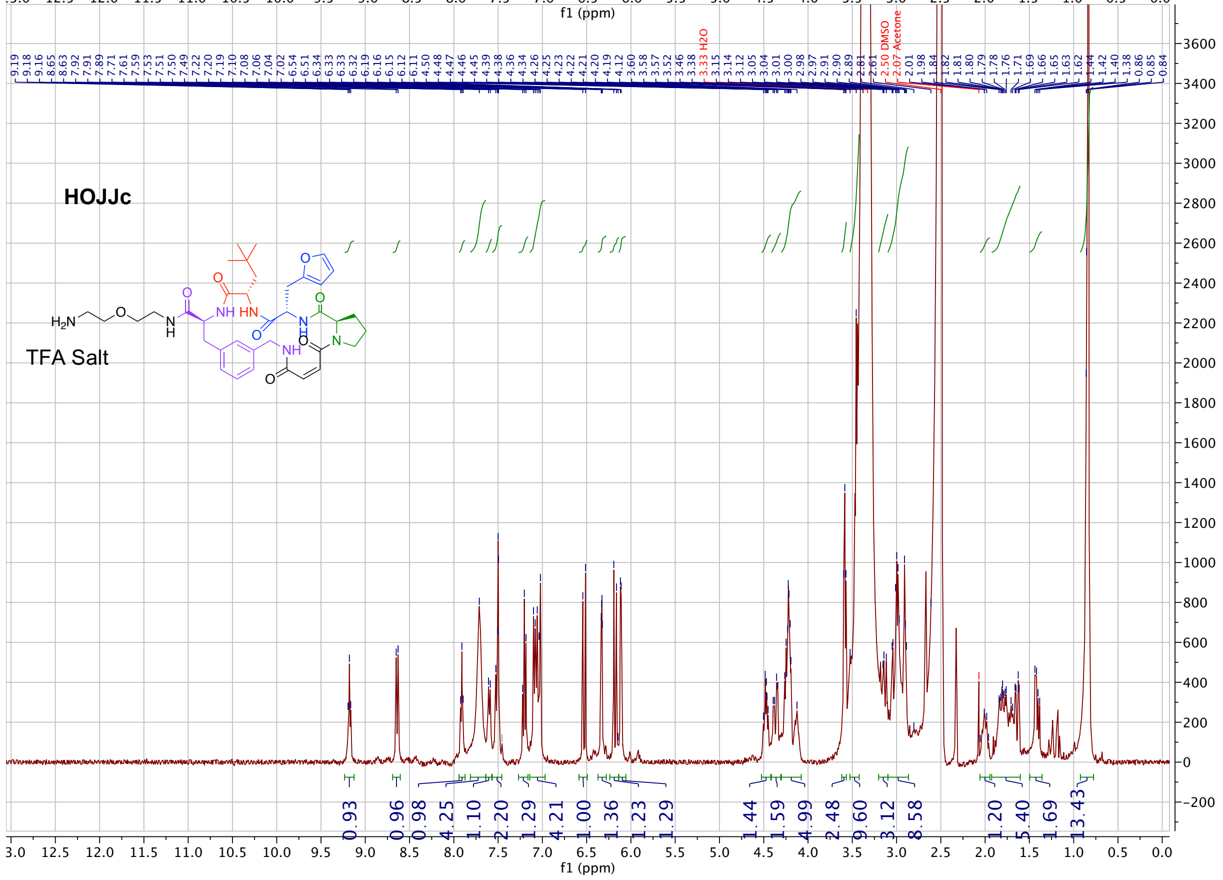
HOJJc
TFA Salt
f1 (ppm)

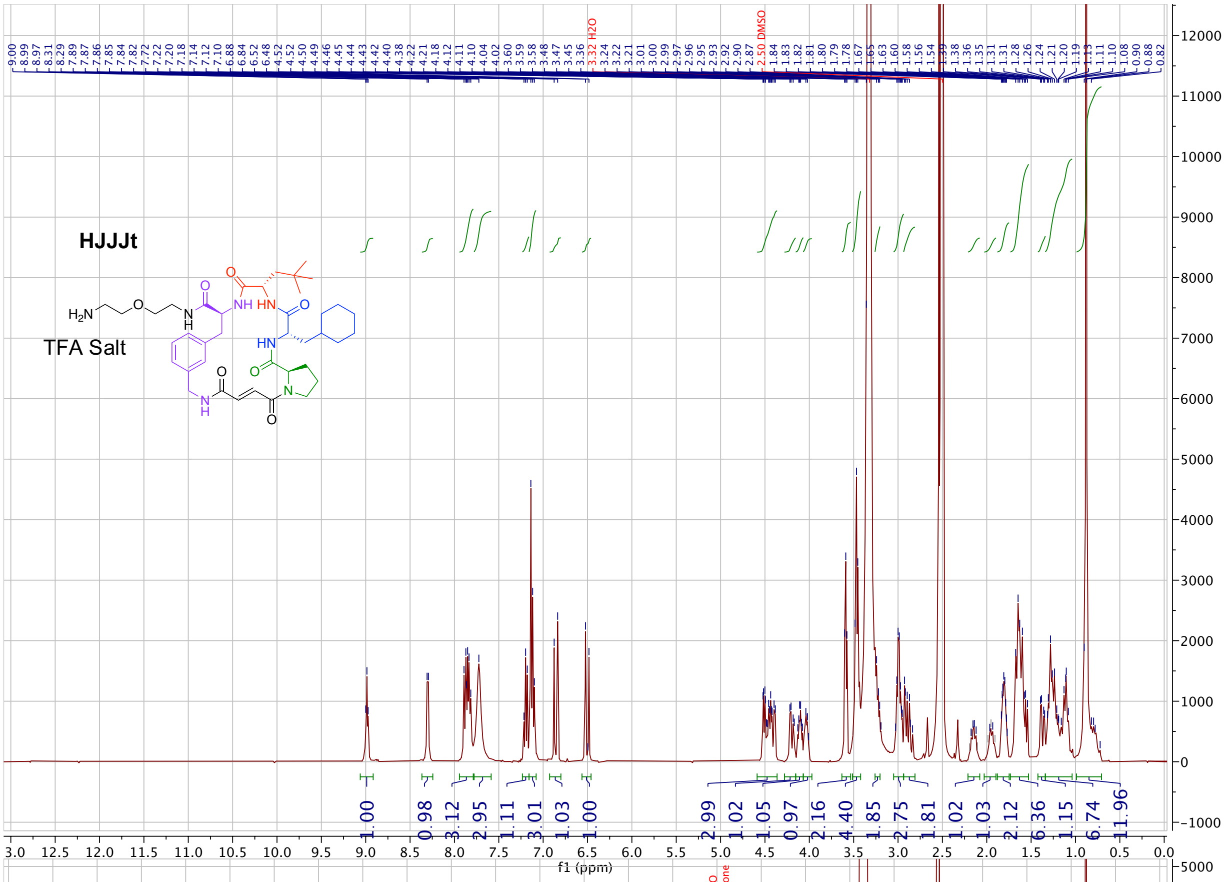
HJJJt
TFA Salt
3.32 H2O
2.50 DMSO
f1 (ppm)

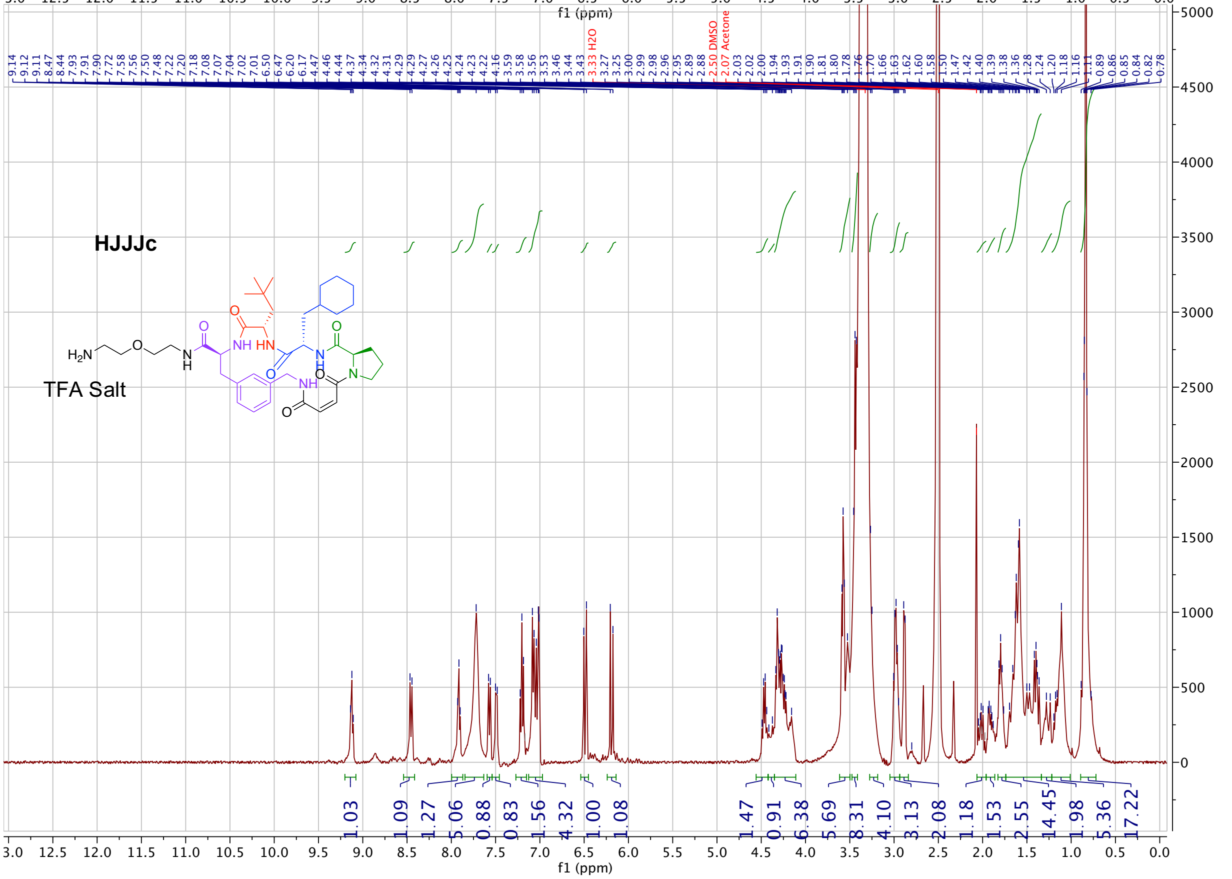
HJJJc
TFA Salt
3.33 H2O
2.50 DMSO
2.07 Acetone
f1 (ppm)

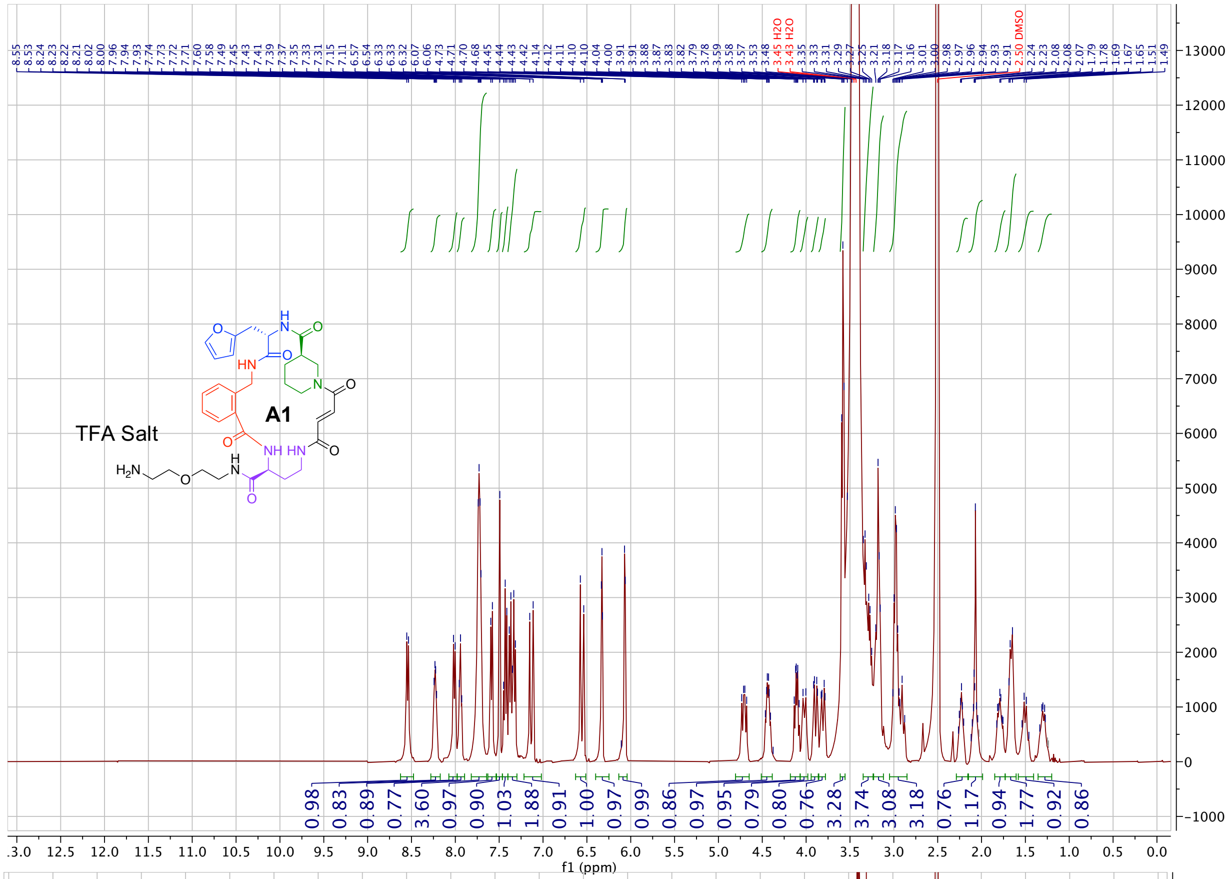
TFA Salt
A1
f1 (ppm)

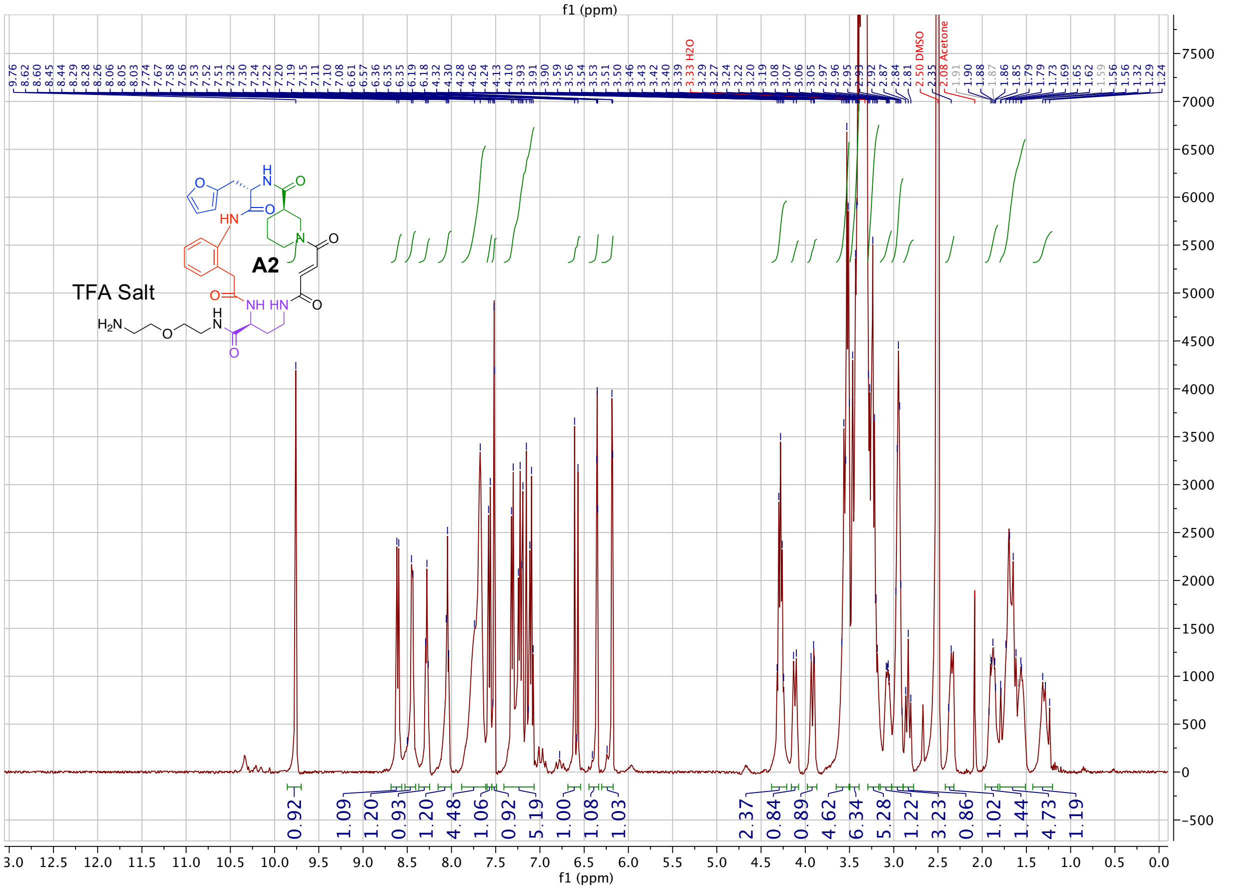
TFA Salt
A2
f1 (ppm)

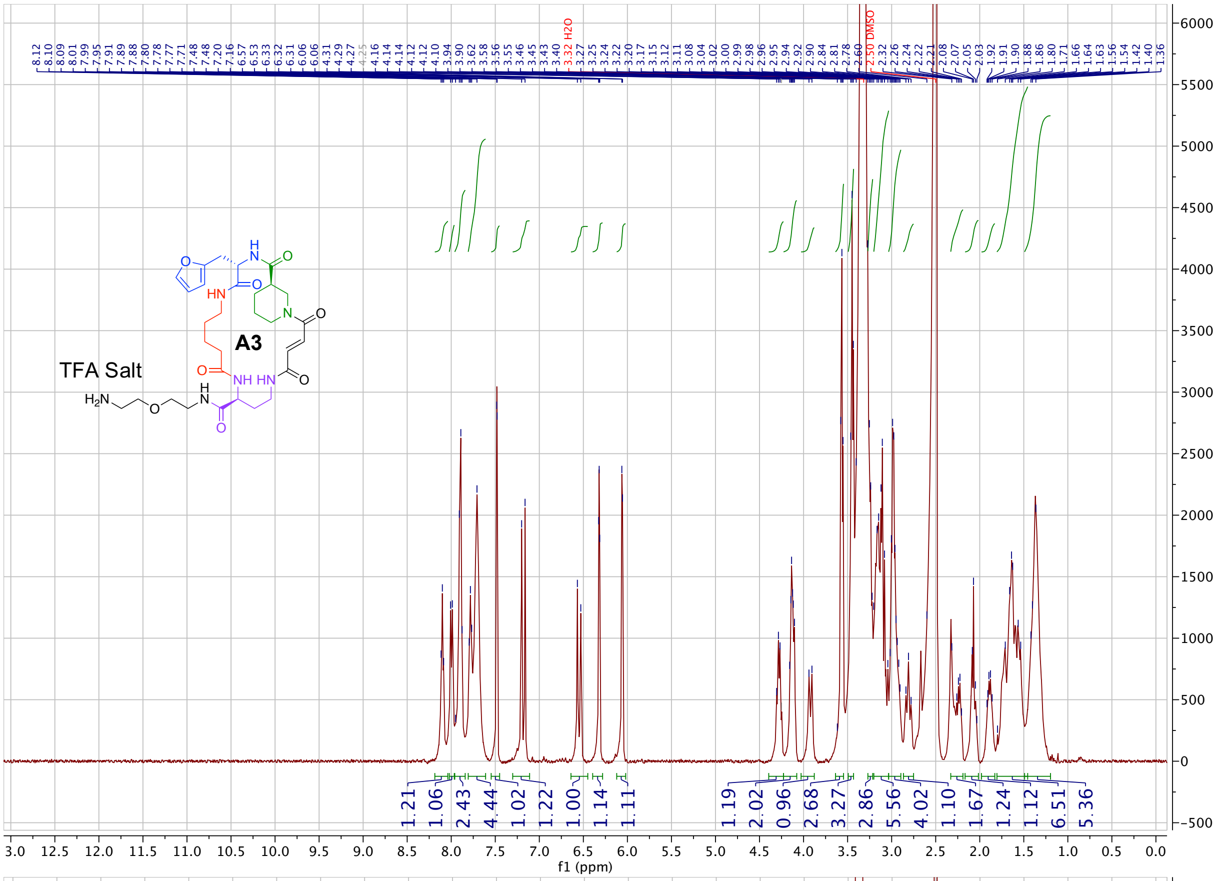
TFA Salt
A3
f1 (ppm)

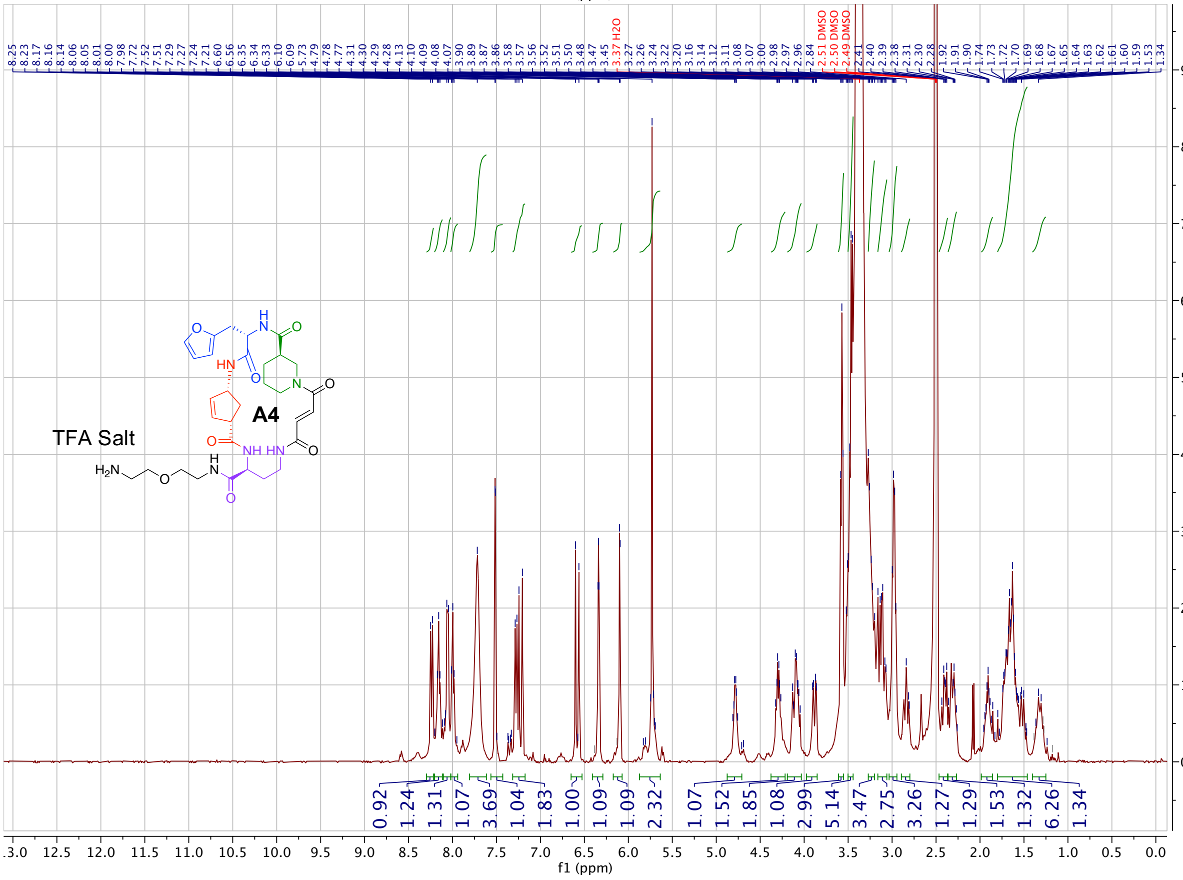
TFA Salt
A4
f1 (ppm)

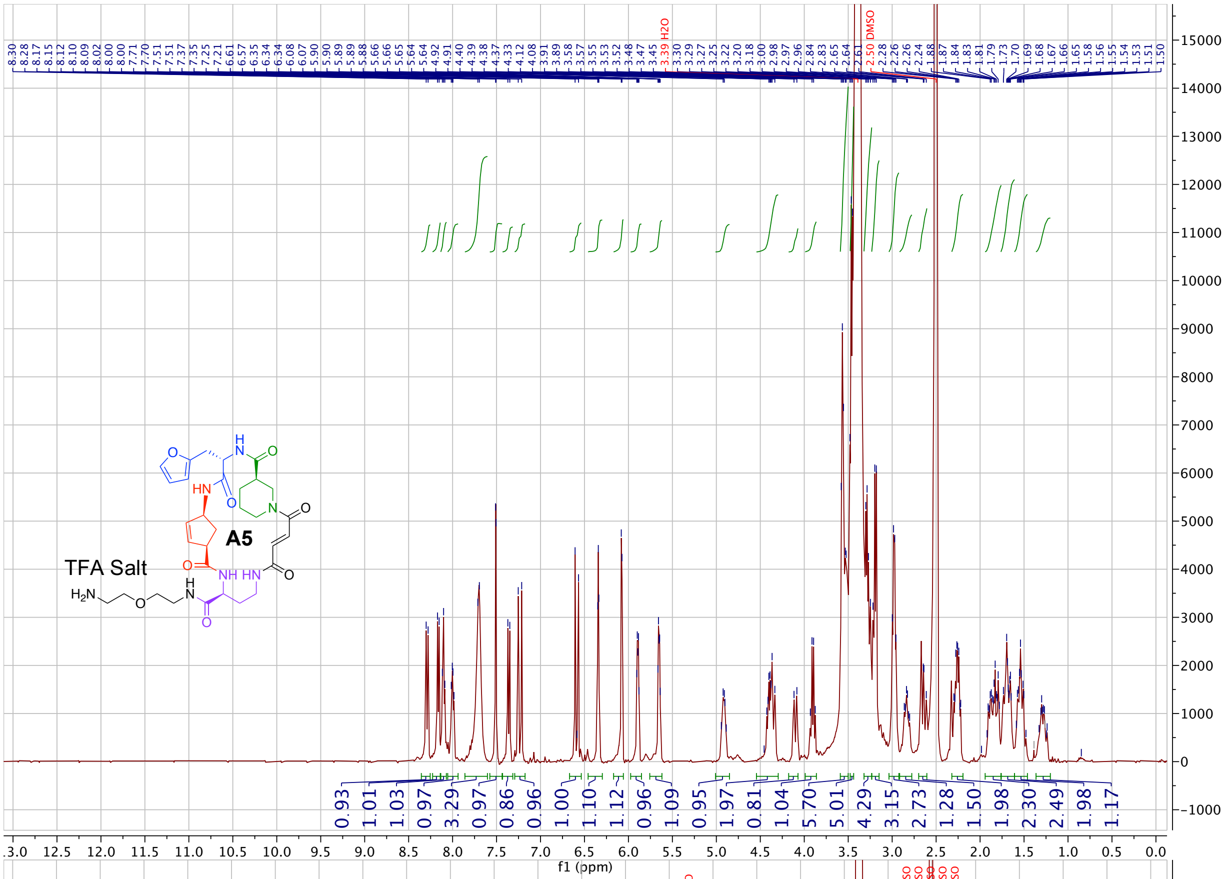
TFA Salt
A5
f1 (ppm)

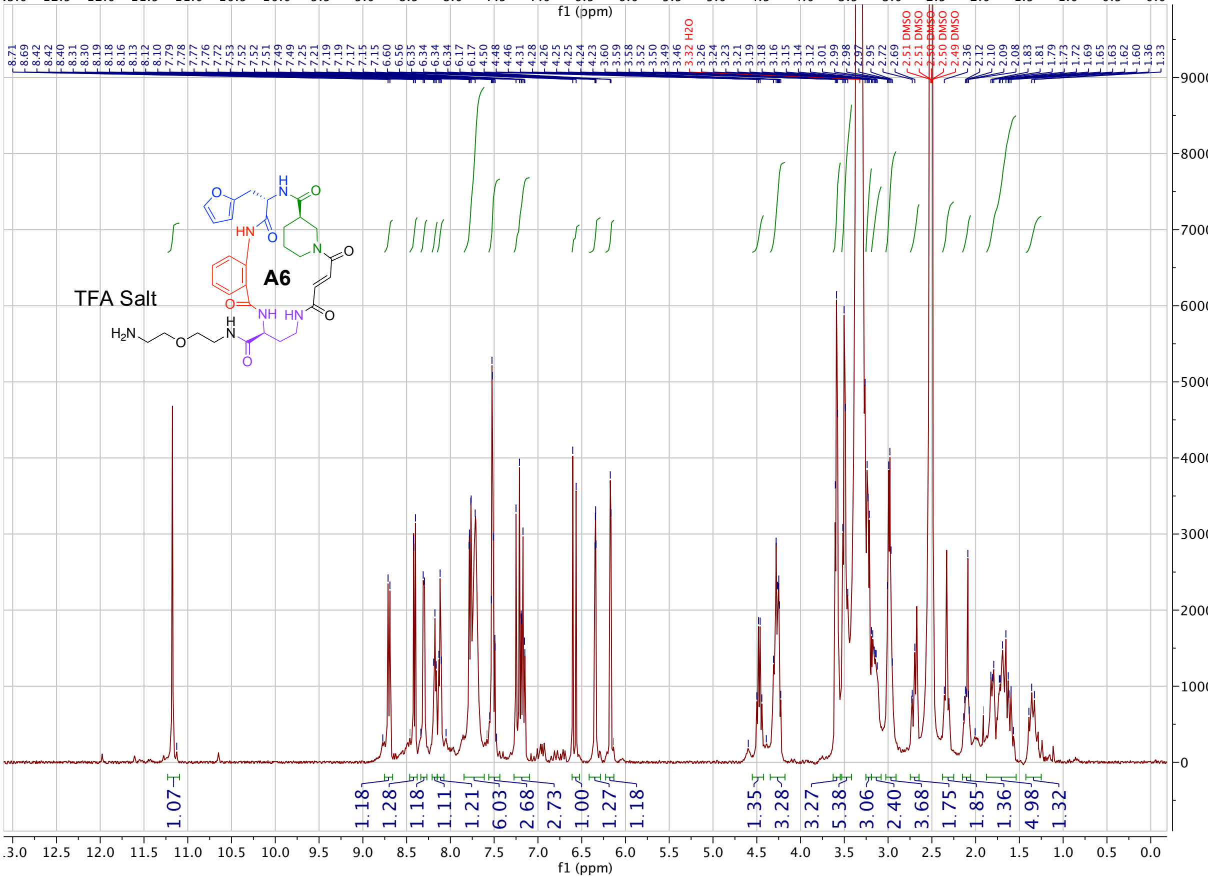
TFA Salt
A6
f1 (ppm)

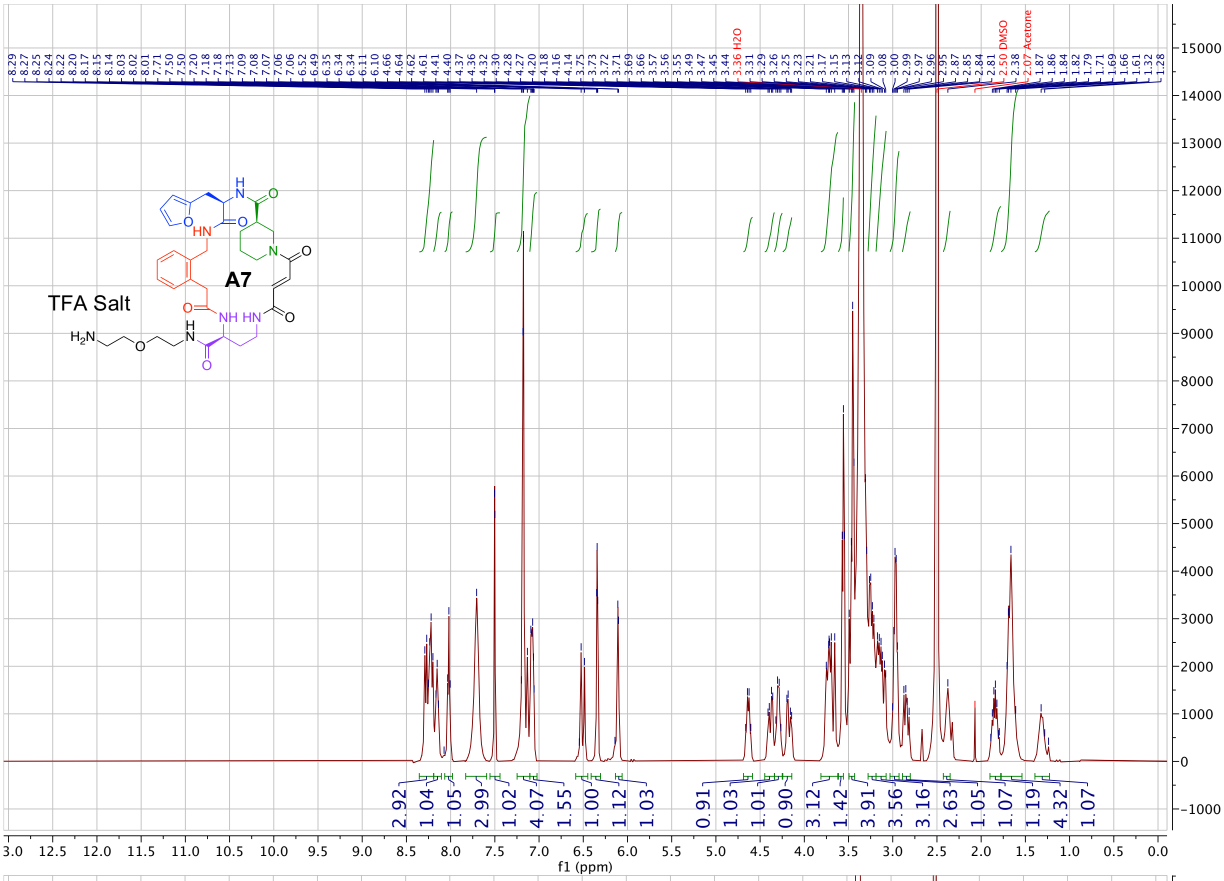
TFA Salt
A7
f1 (ppm)

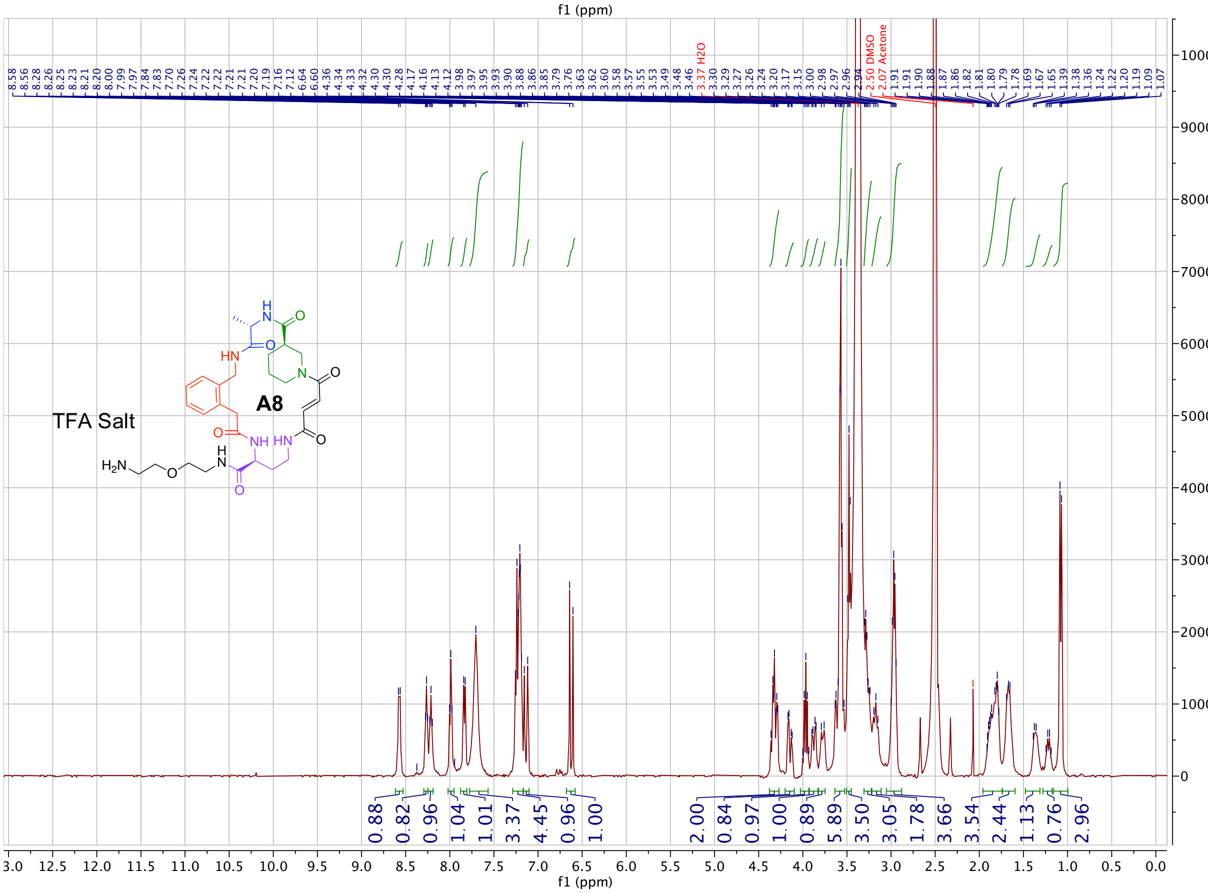
TFA Salt
A8
f1 (ppm)

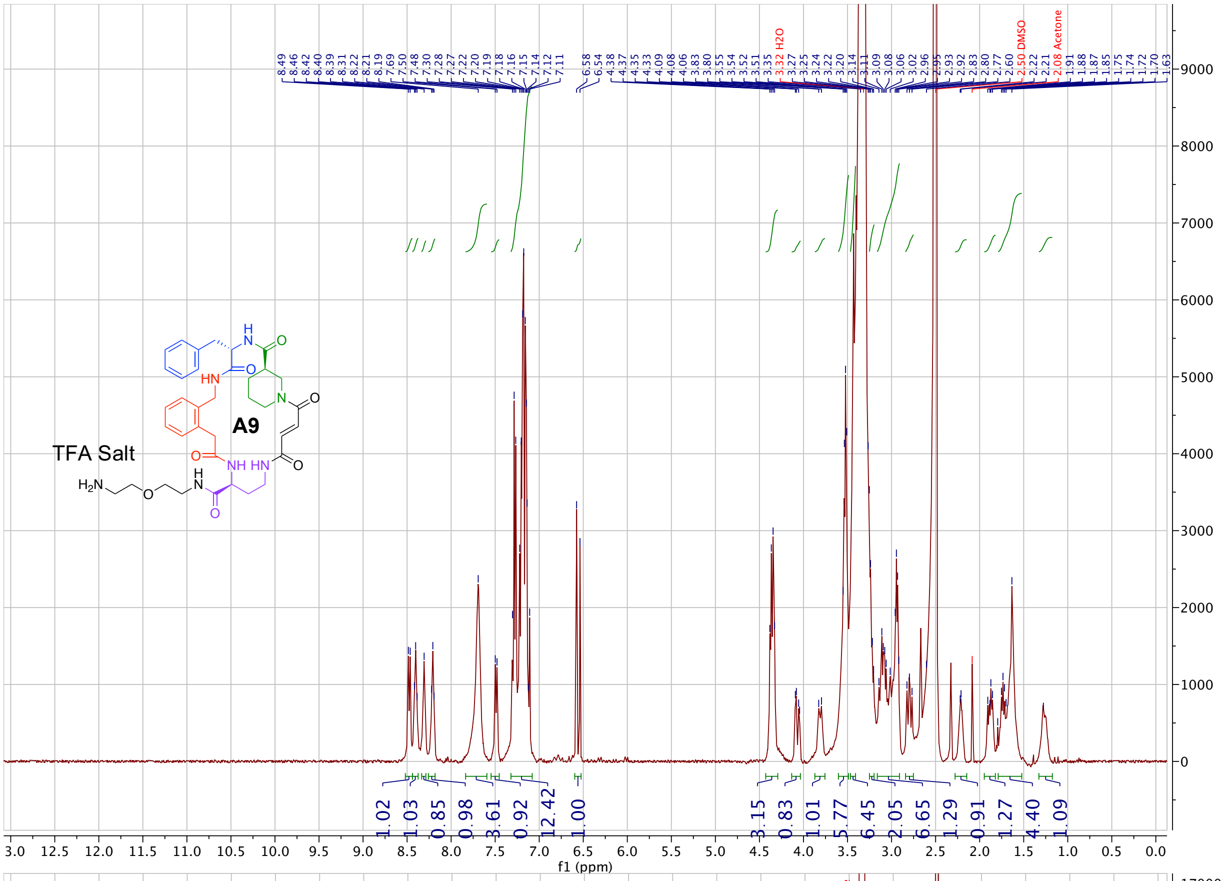
TFA Salt
A9
3.32 H2O
2.50 DMSO
2.08 Acetone
f1 (ppm)

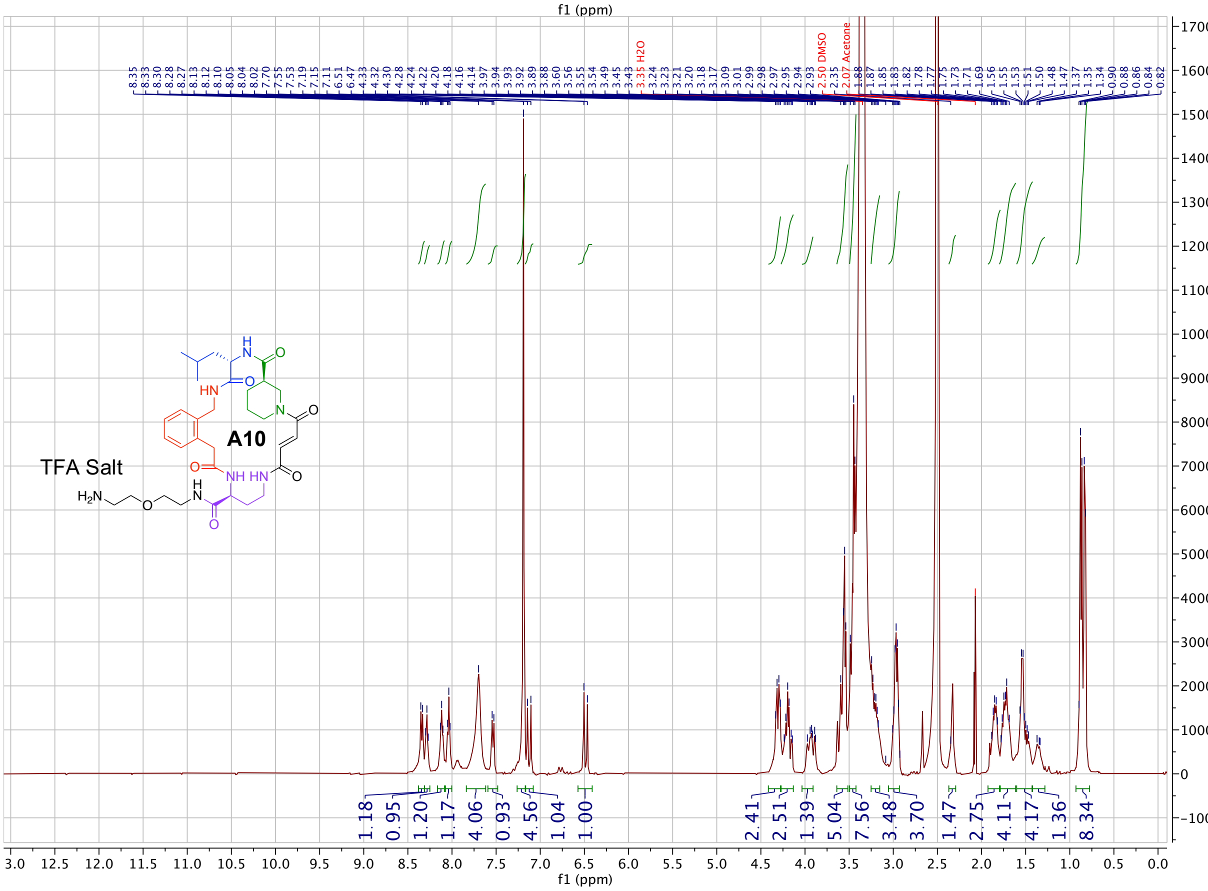
TFA Salt
A10
3.35 H2O
2.50 DMSO
2.07 Acetone
f1 (ppm)

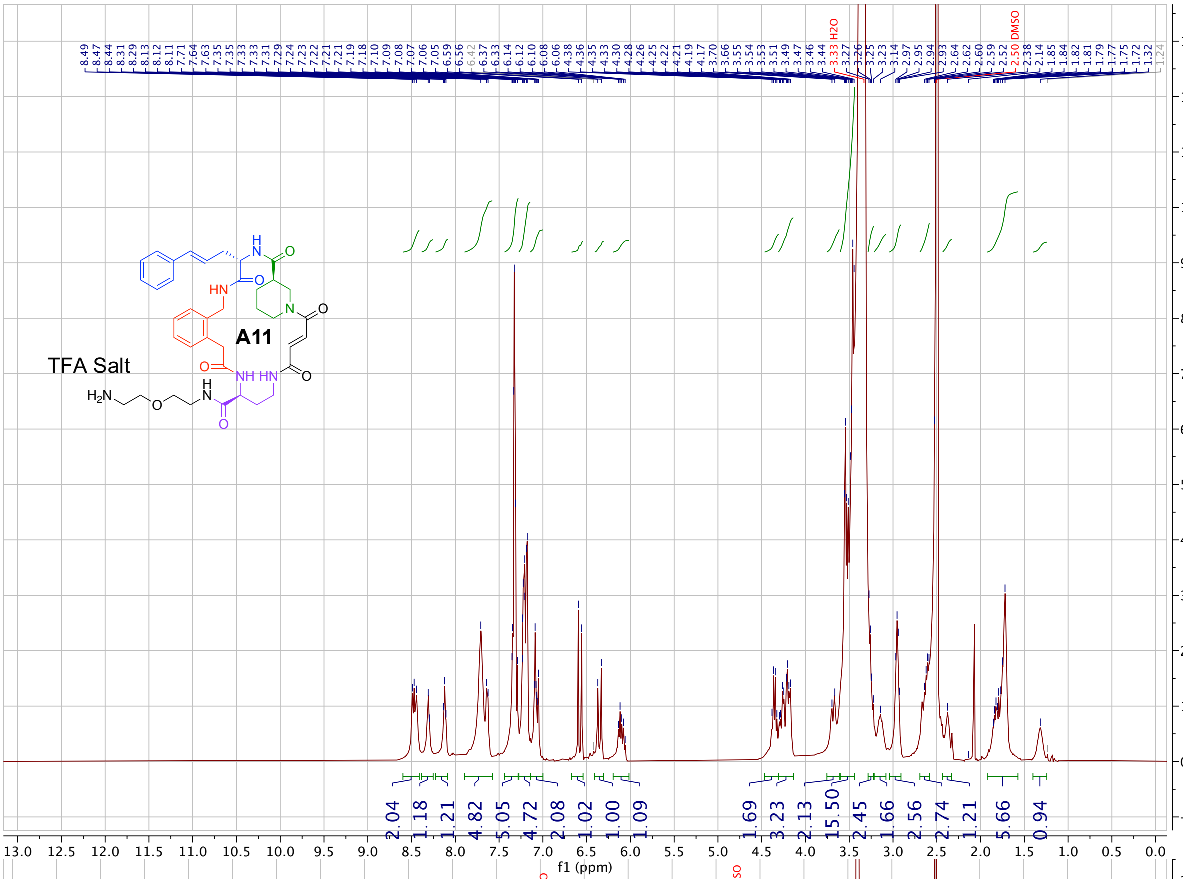
A11
TFA Salt
f1 (ppm)

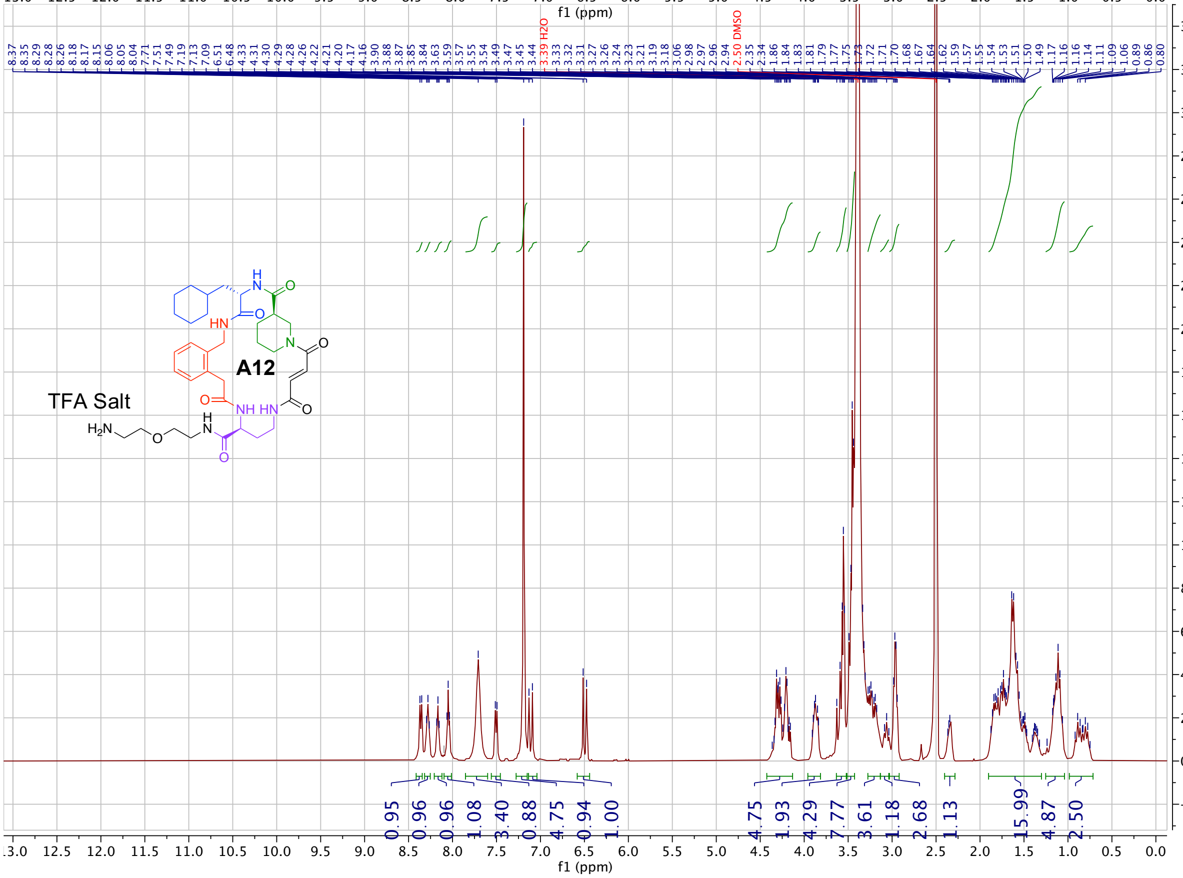
A12
TFA Salt
f1 (ppm)

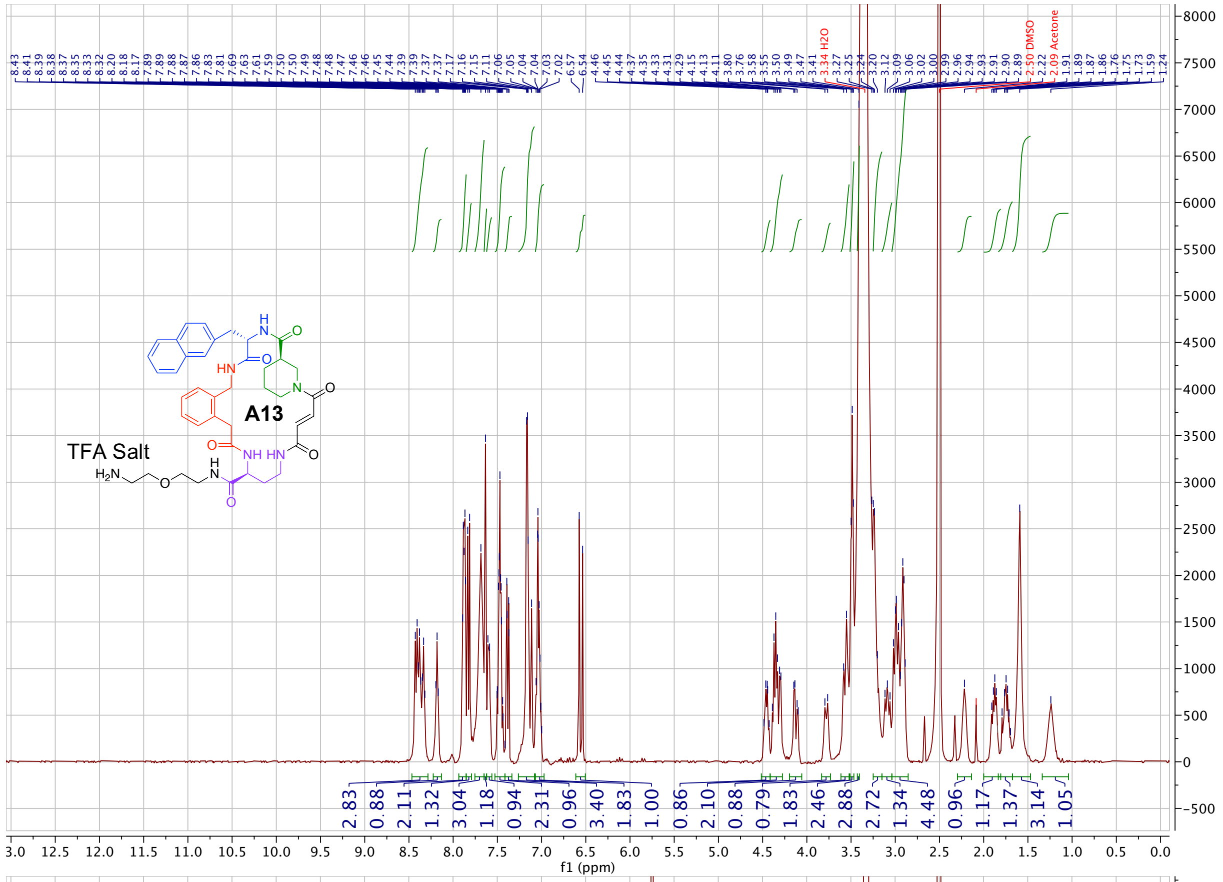
TFA Salt
A13

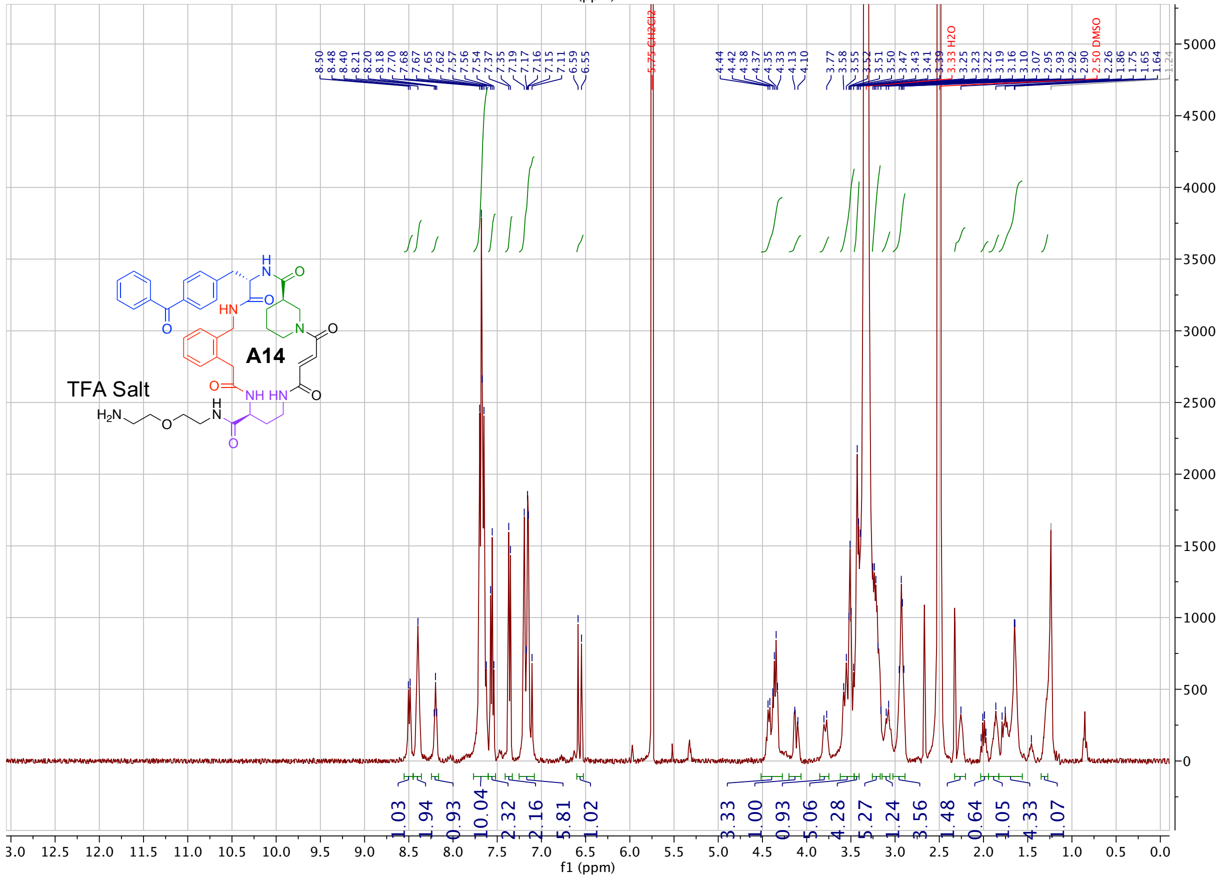
TFA Salt
A14

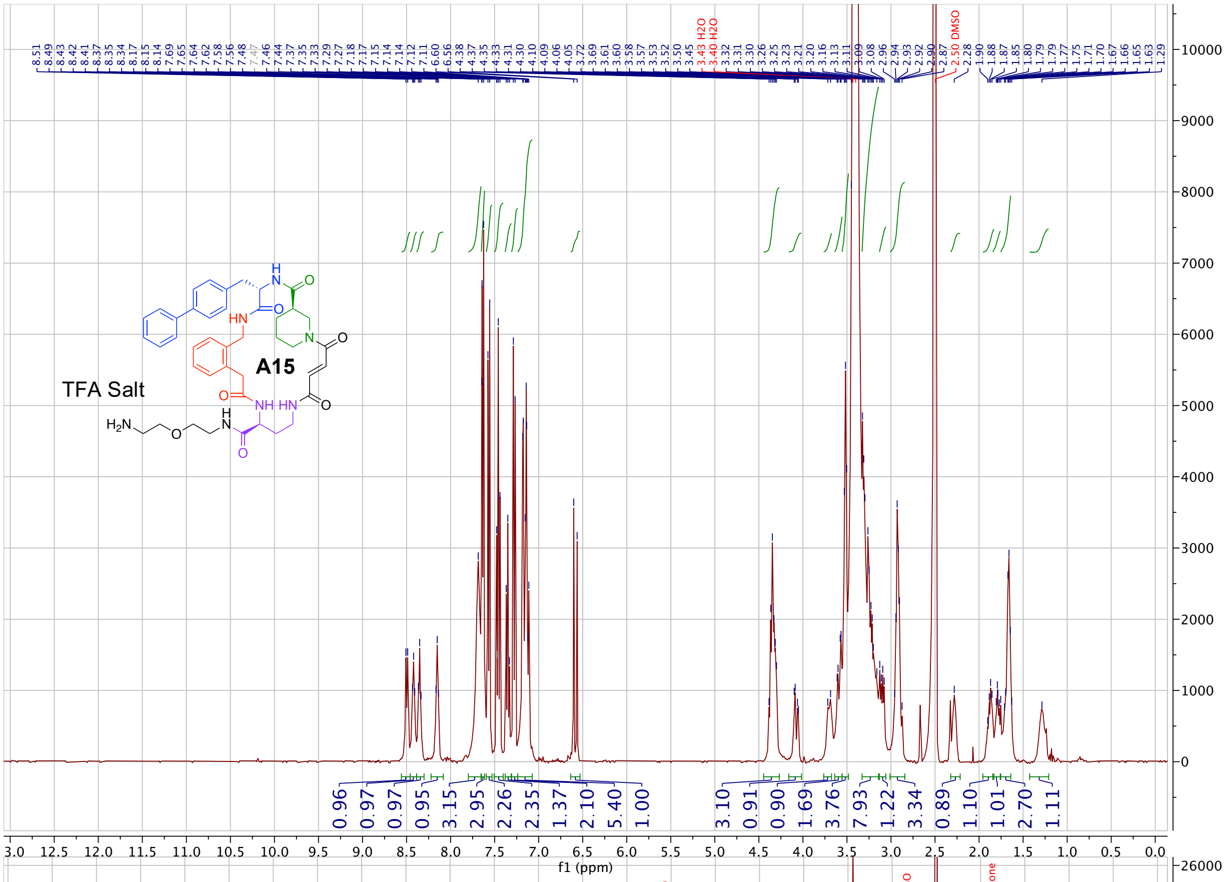
TFA Salt
A15
f1 (ppm)

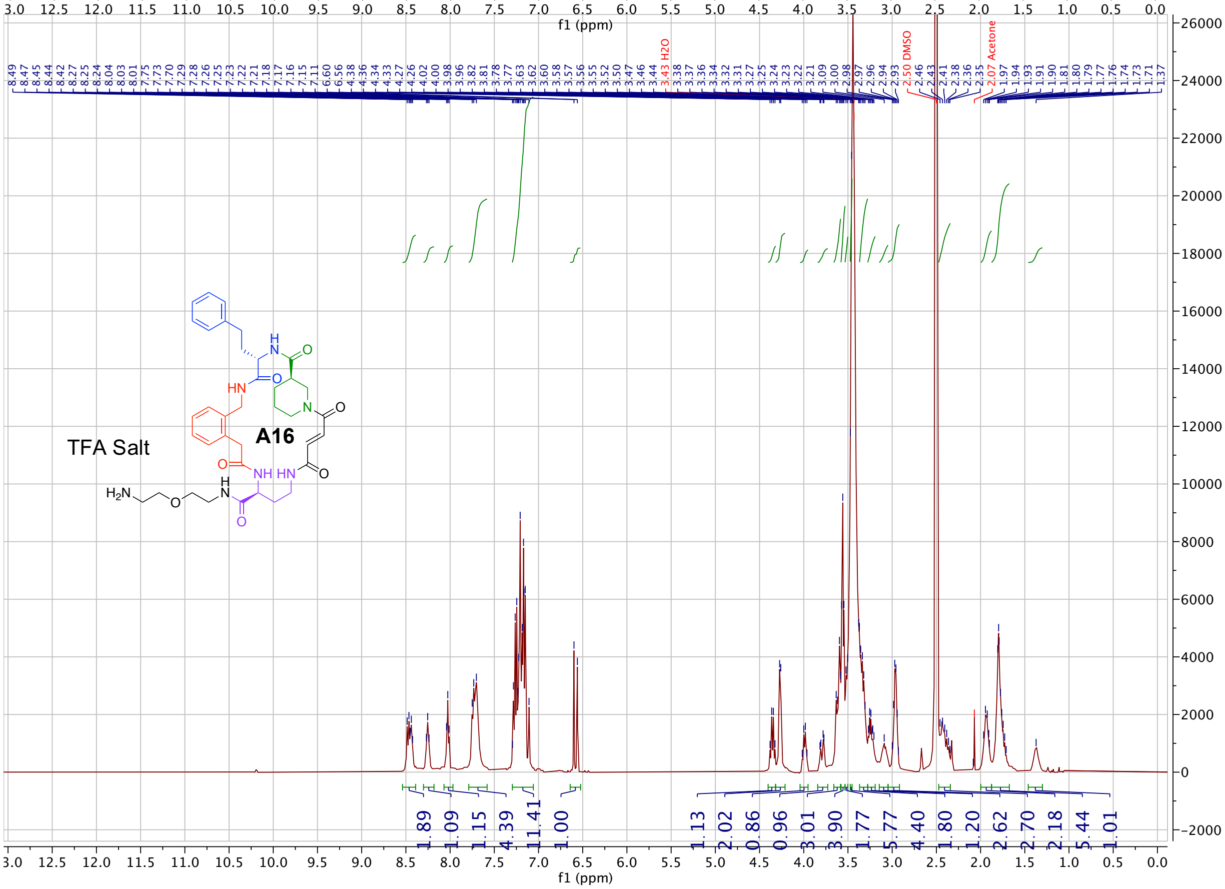
TFA Salt
A16
f1 (ppm)

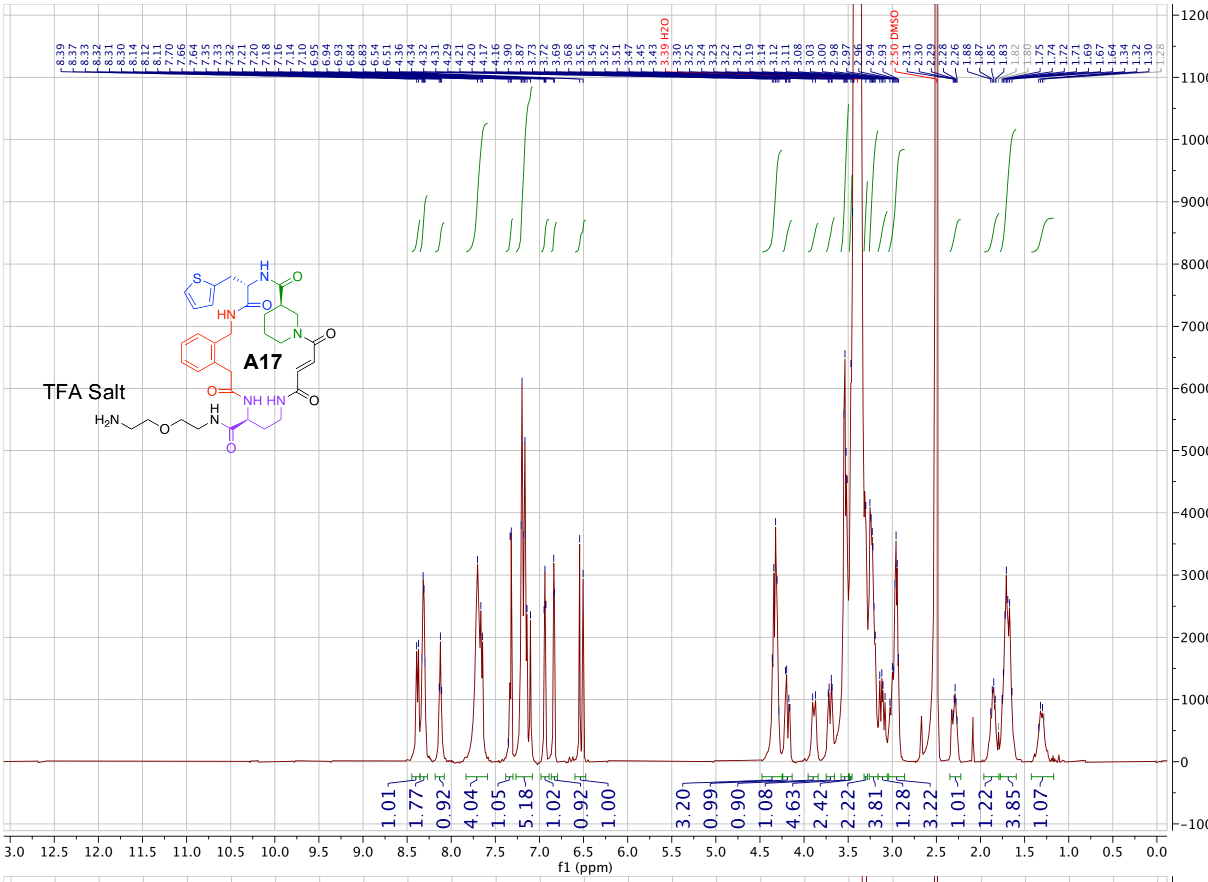
TFA Salt
A17
f1 (ppm)

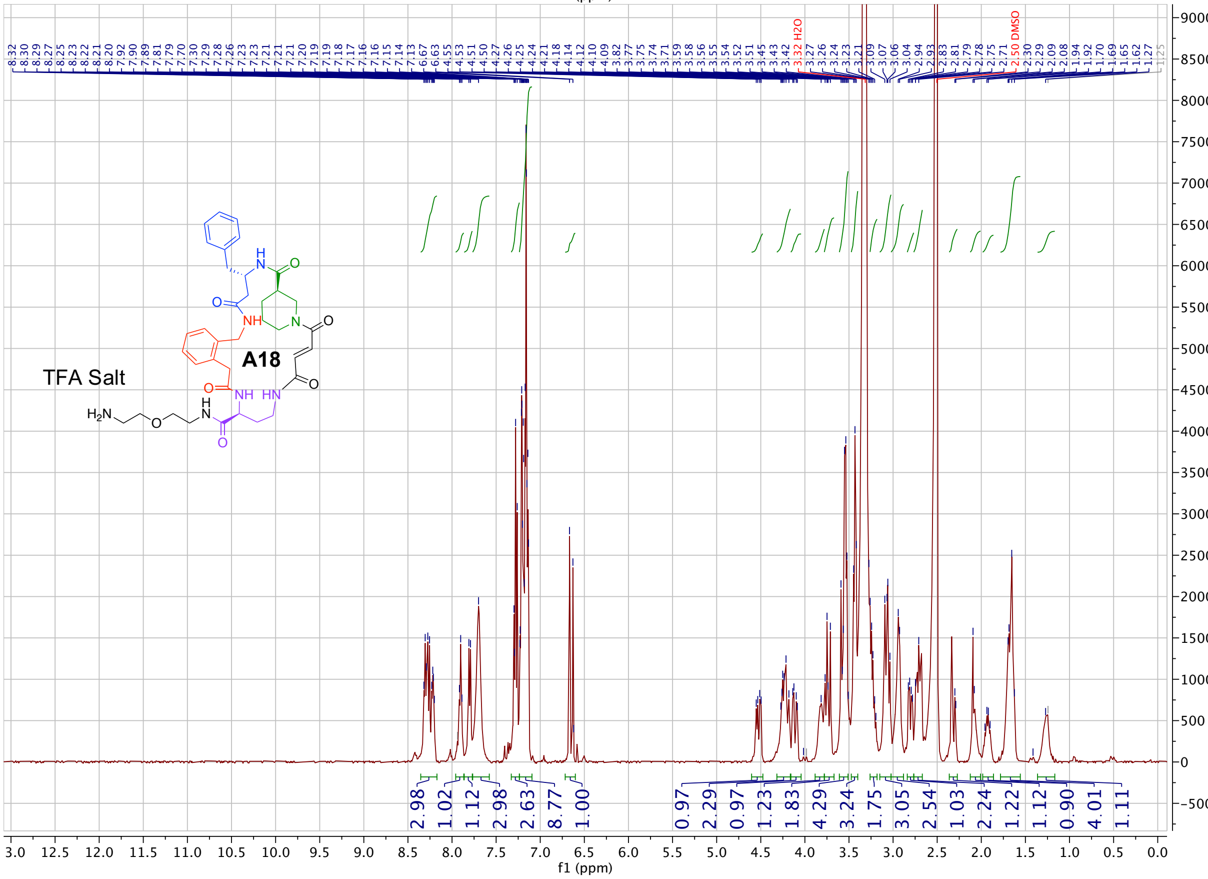
TFA Salt
A18
f1 (ppm)

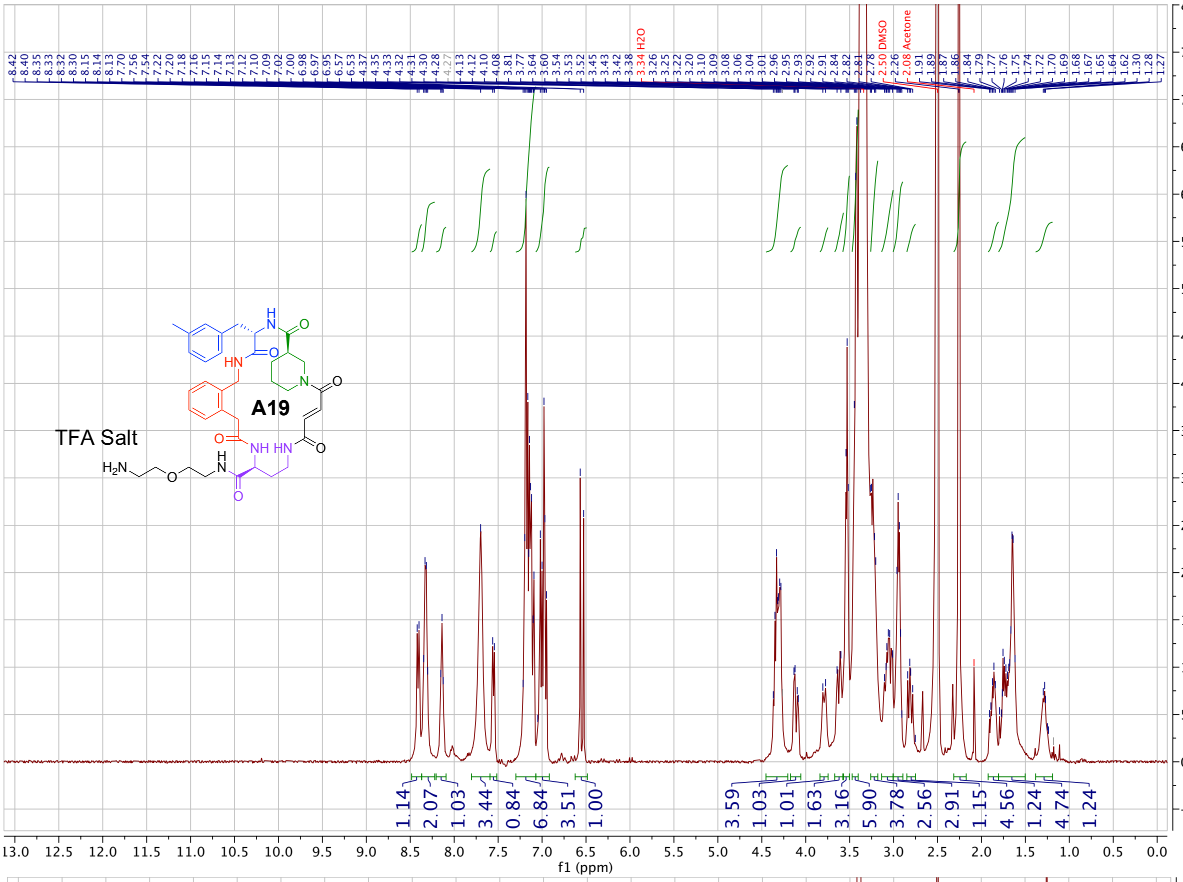
TFA Salt
A19
1.14 2.07 1.03 3.44 0.84 6.84 3.51 1.00 3.59 1.03 1.01 1.63 3.16 5.90 3.78 2.56 2.91 1.15 4.56 1.24 4.74 1.24
f1 (ppm)

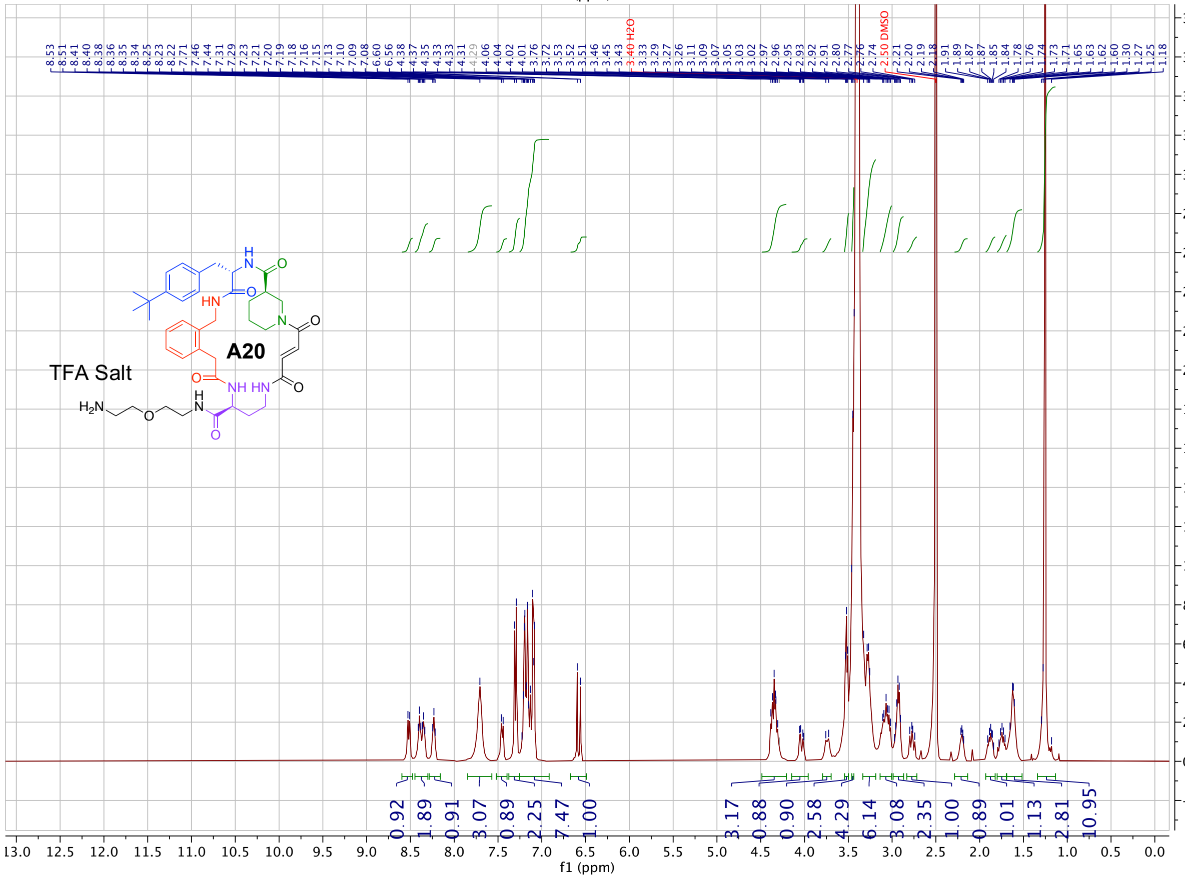
TFA Salt
A20
0.92 1.89 0.91 3.07 0.89 2.25 7.47 1.00 3.17 0.88 0.90 2.58 4.29 6.14 3.08 2.35 1.00 0.89 1.01 1.13 2.81 10.95
f1 (ppm)

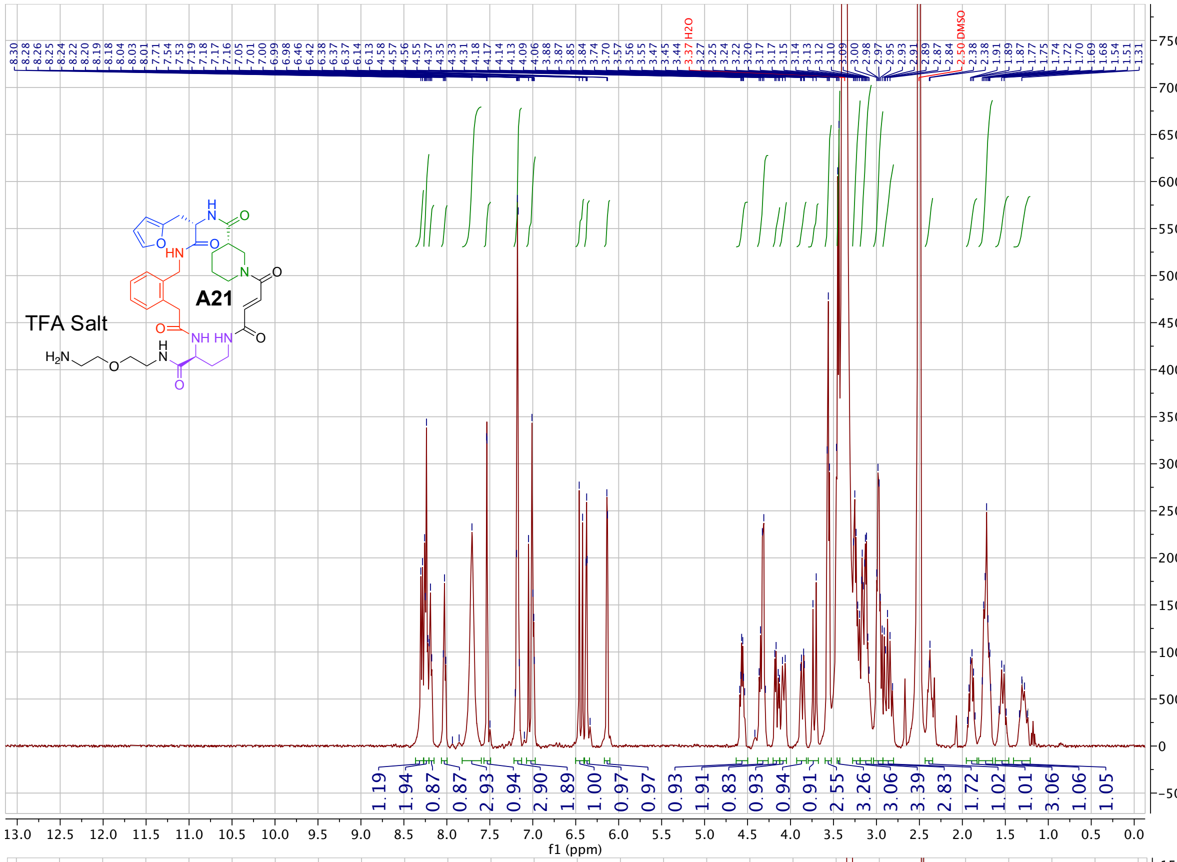
TFA Salt
A21
3.37 H2O
2.50 DMSO
f1 (ppm)

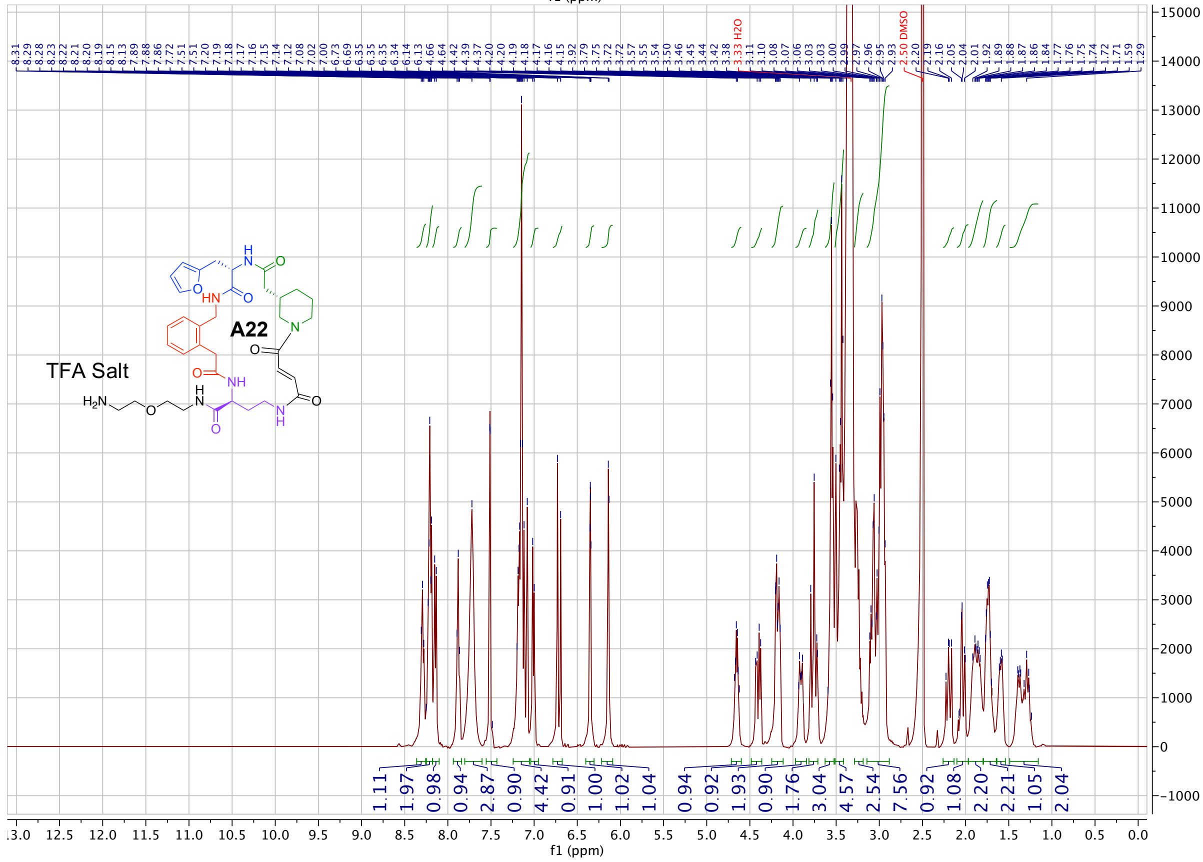
TFA Salt
A22
3.33 H2O
2.50 DMSO
f1 (ppm)

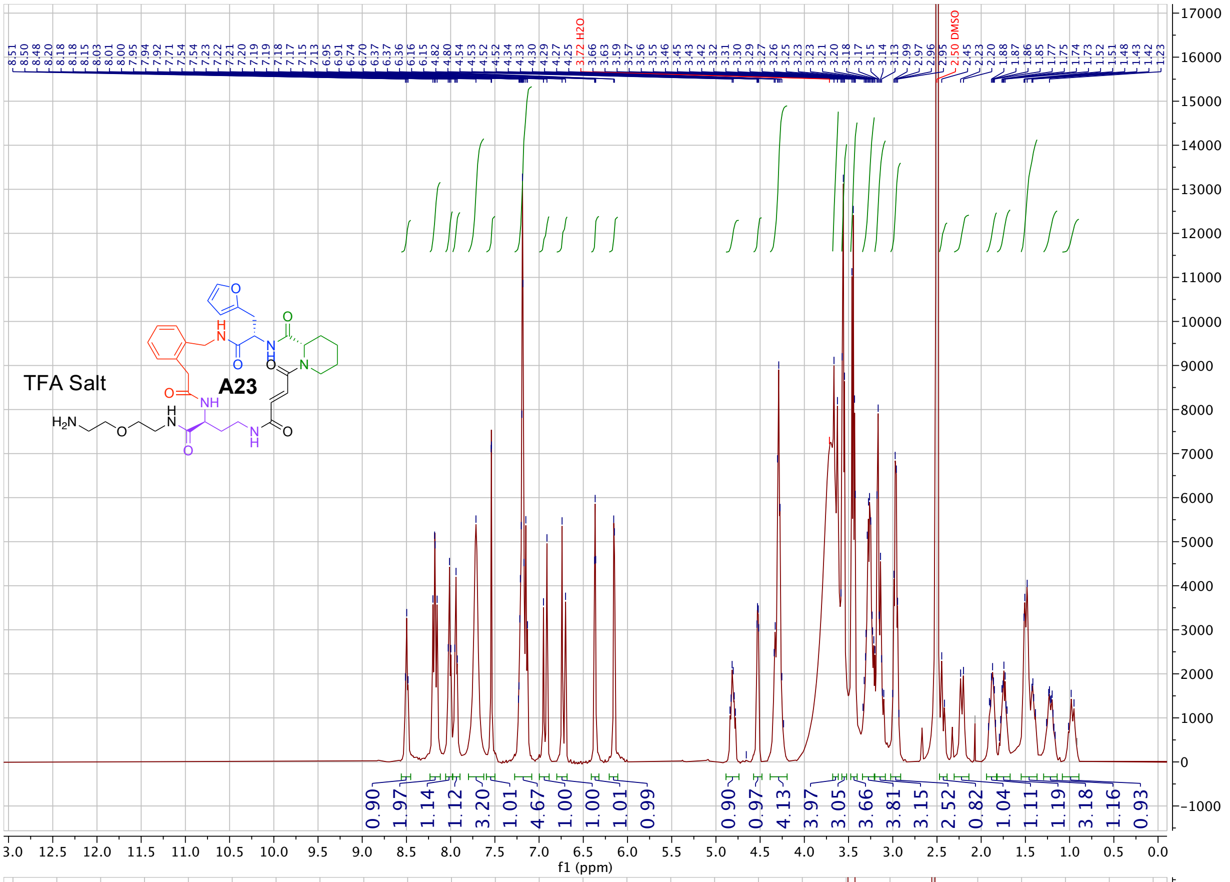
TFA Salt
A23
f1 (ppm)

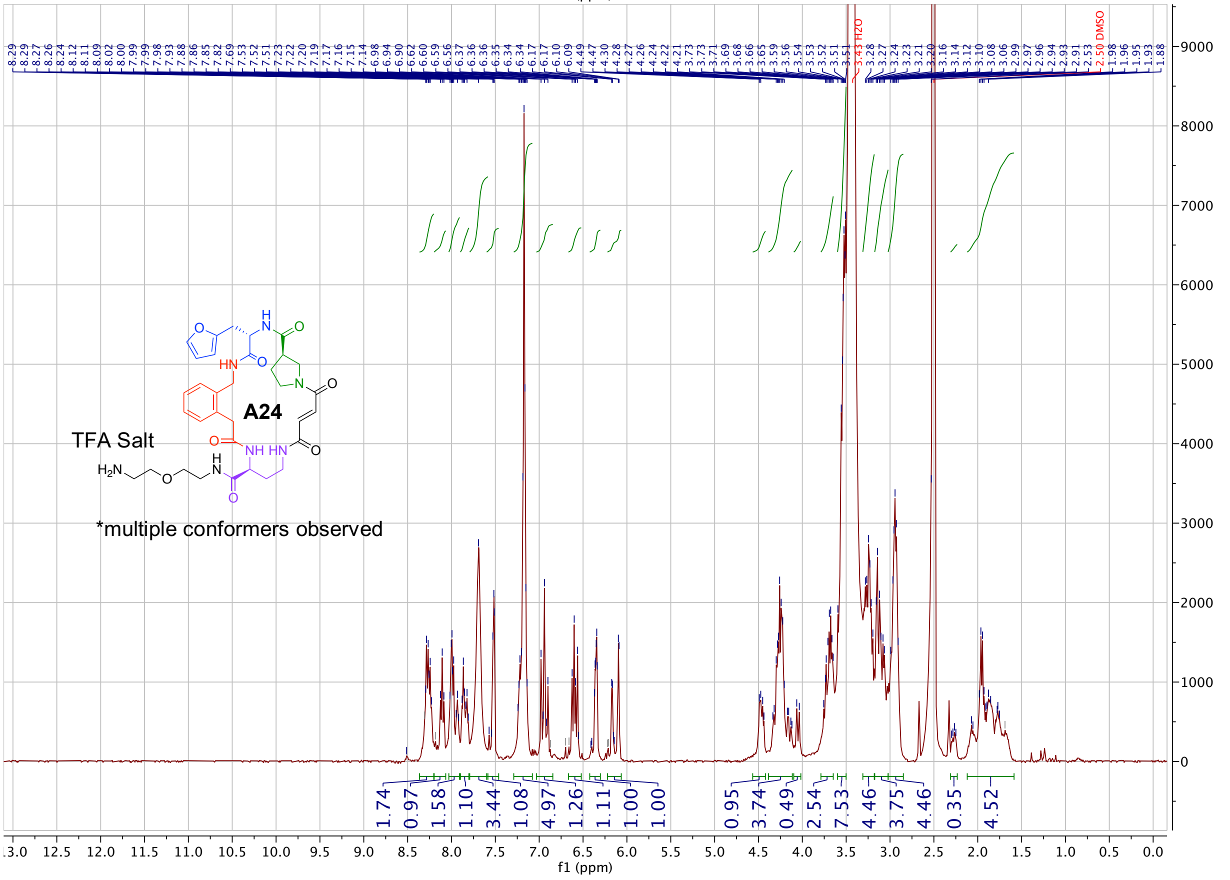
TFA Salt
A24
*multiple conformers observed
f1 (ppm)

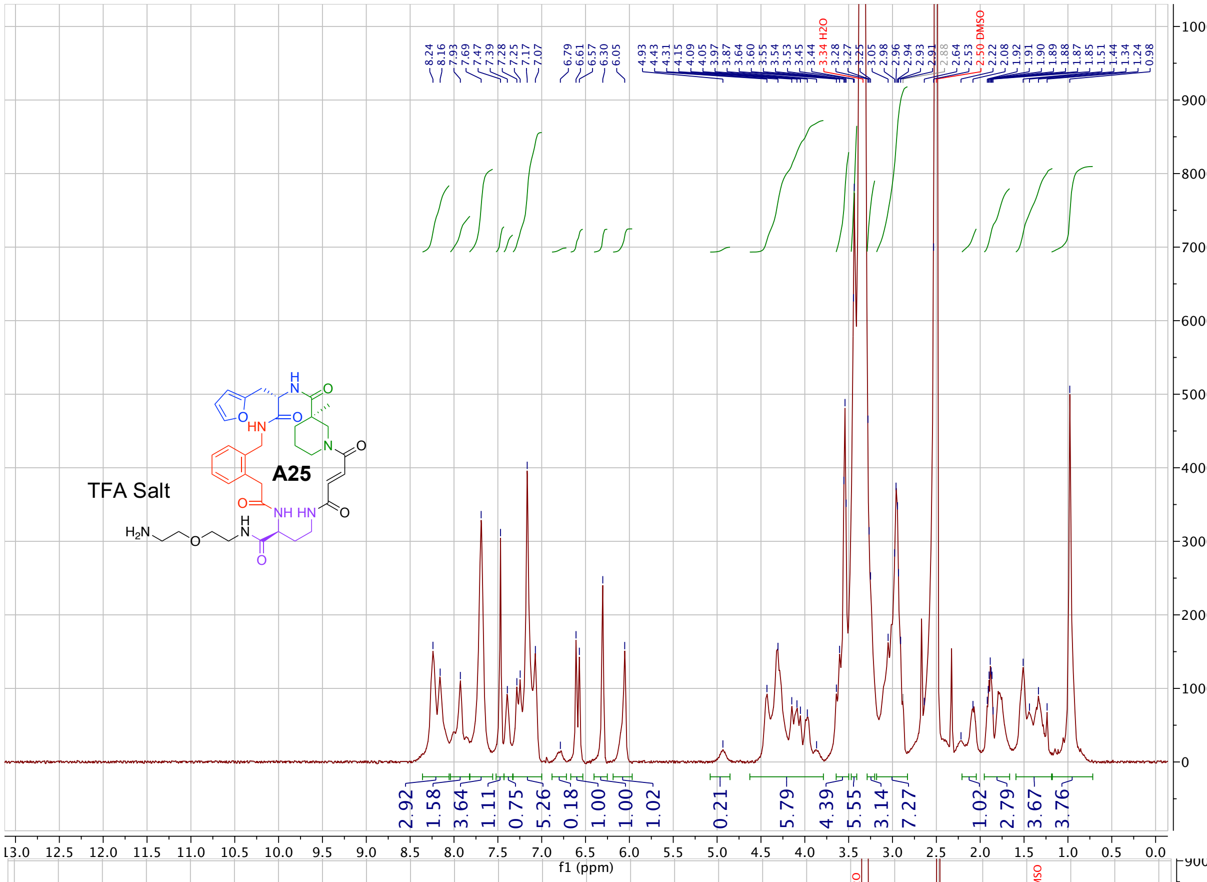
TFA Salt
A25
3.34 H2O
2.50 DMSO
f1 (ppm)

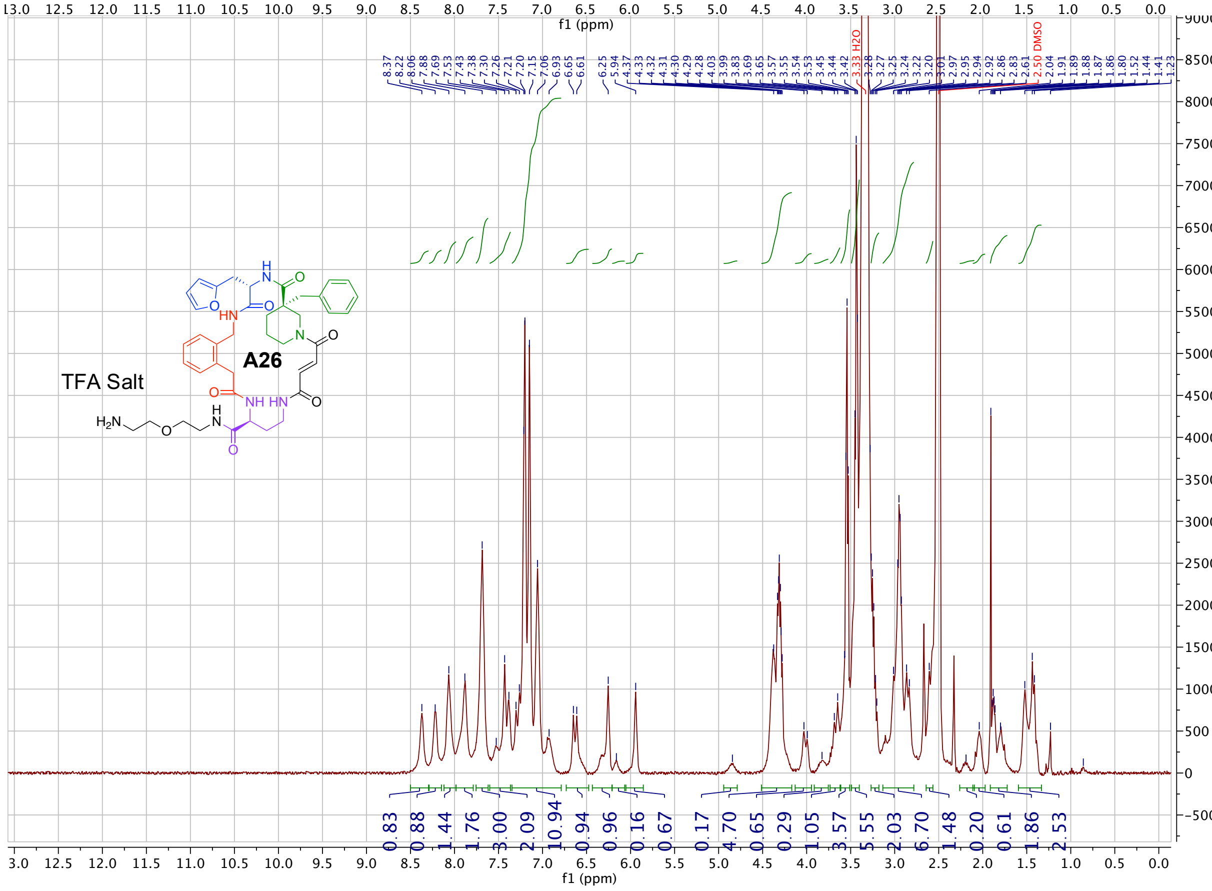
TFA Salt
A26
3.33 H2O
2.50 DMSO
f1 (ppm)

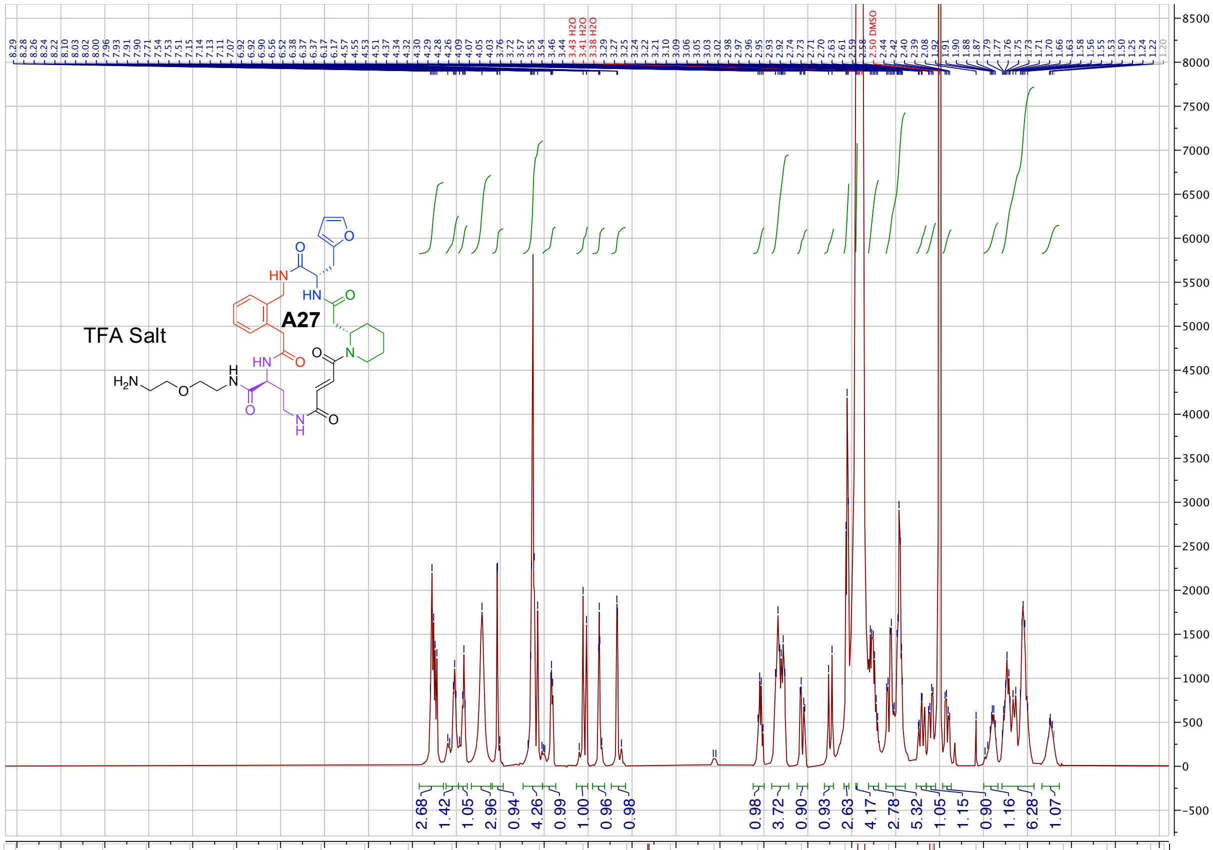
TFA Salt
A27
f1 (ppm)

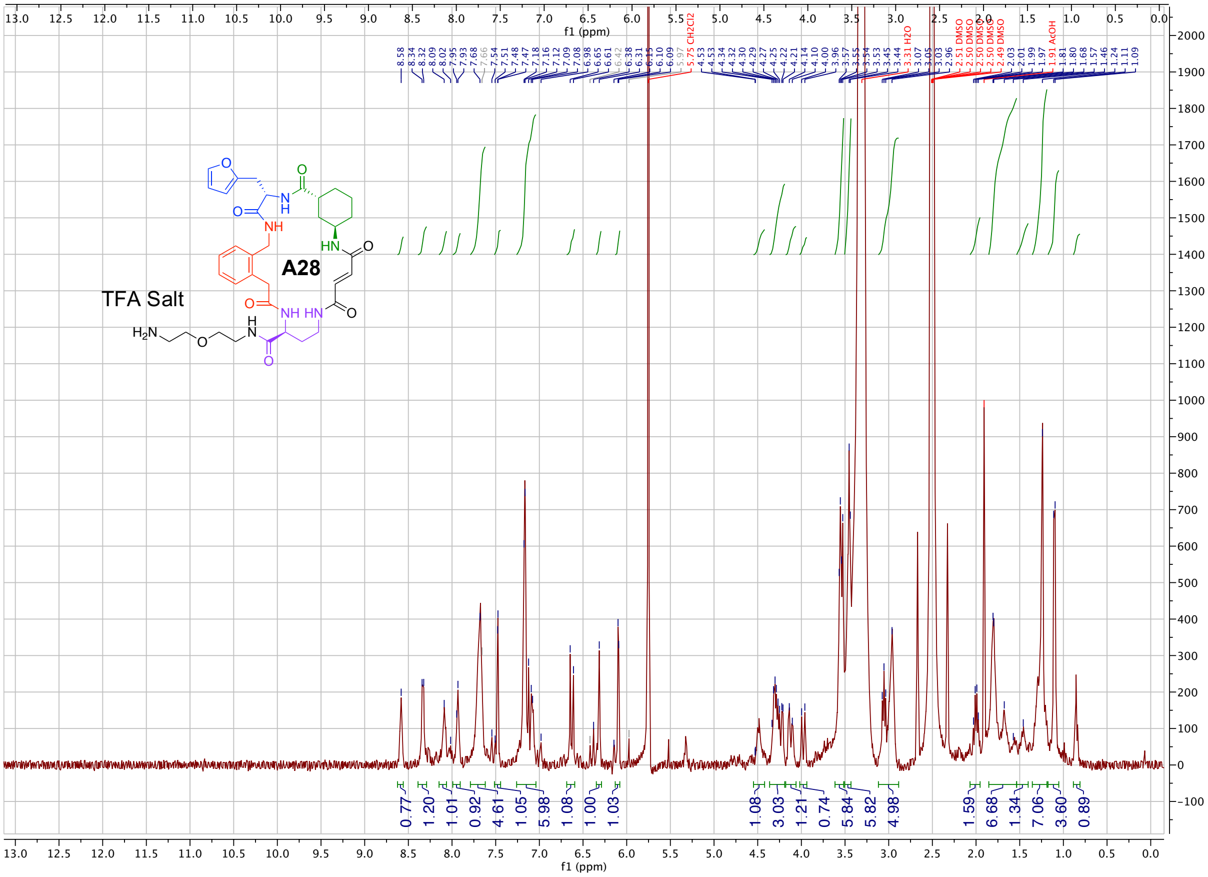
TFA Salt
A28
f1 (ppm)

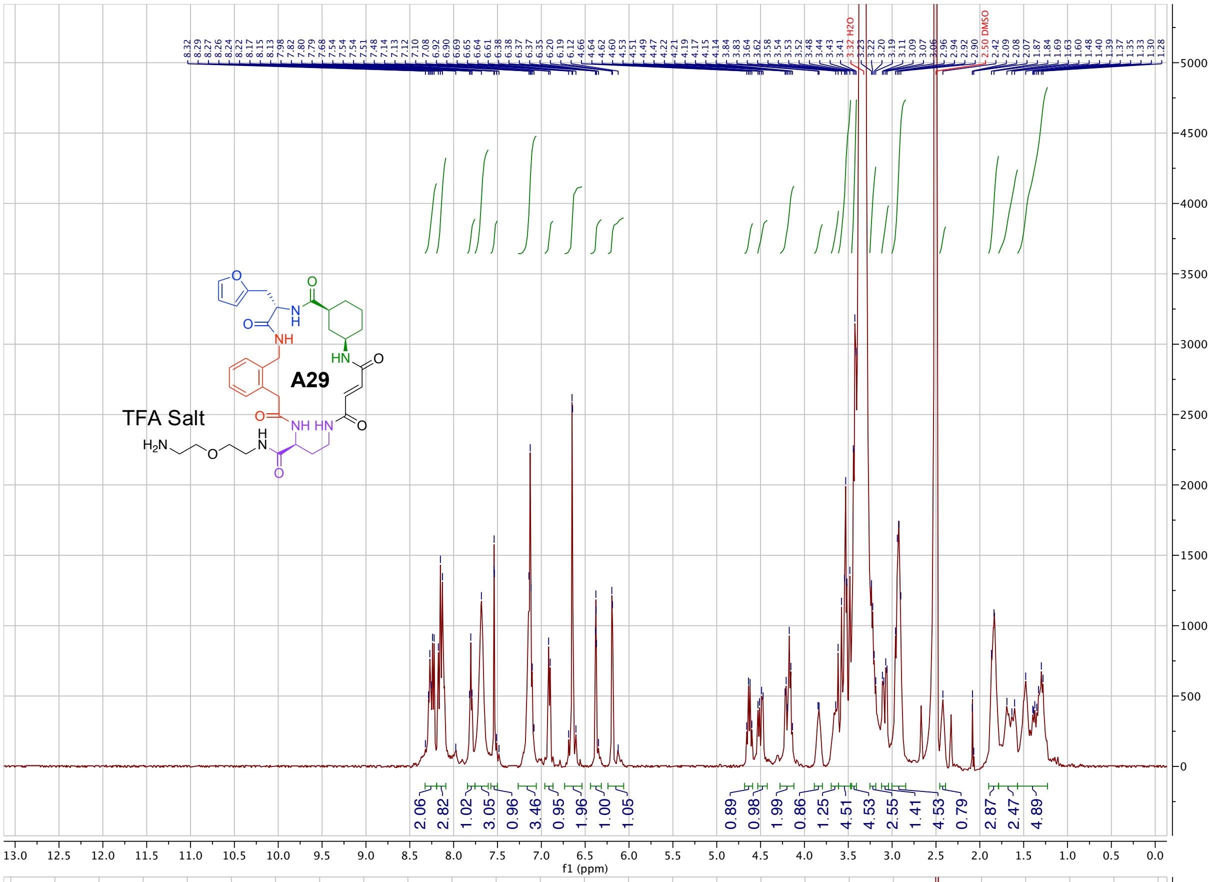

A29
TFA Salt
f1 (ppm)

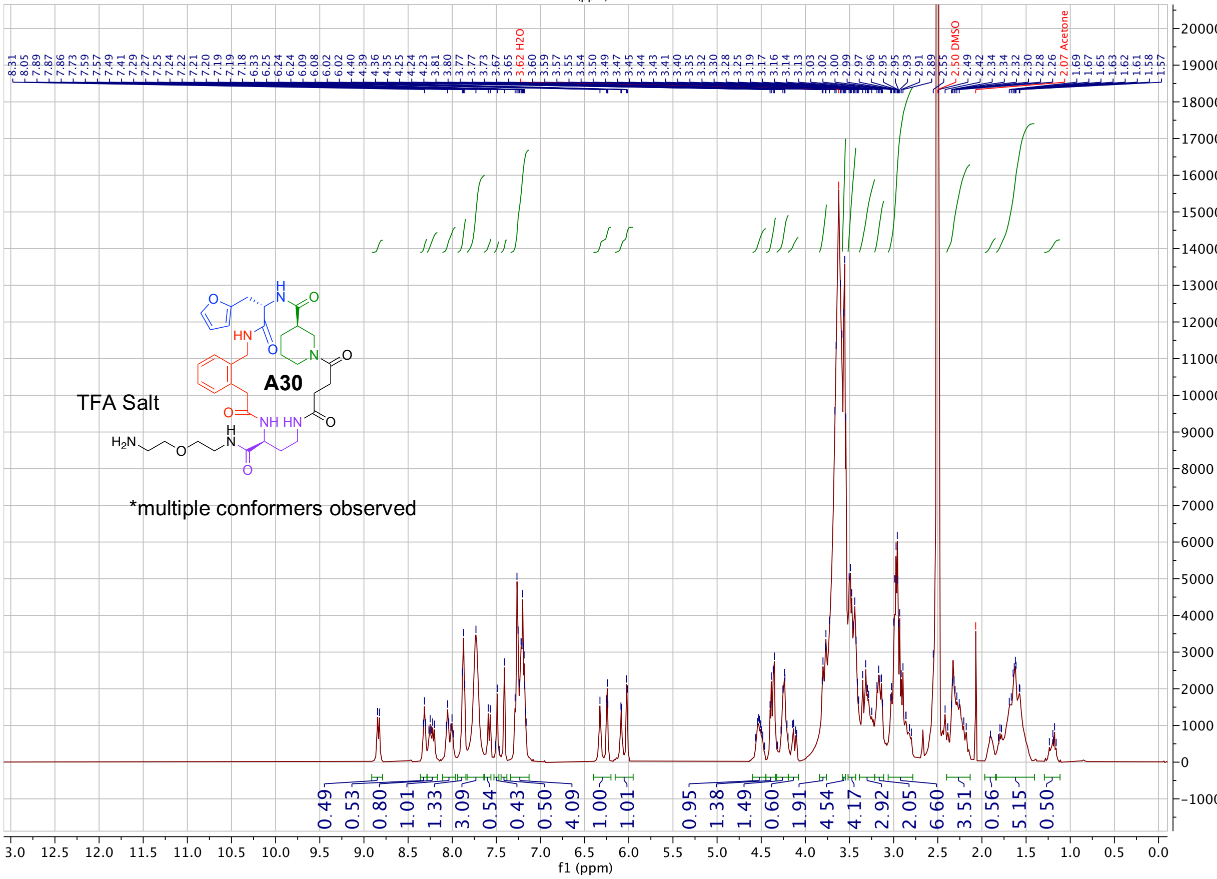

A30
TFA Salt
*multiple conformers observed
f1 (ppm)

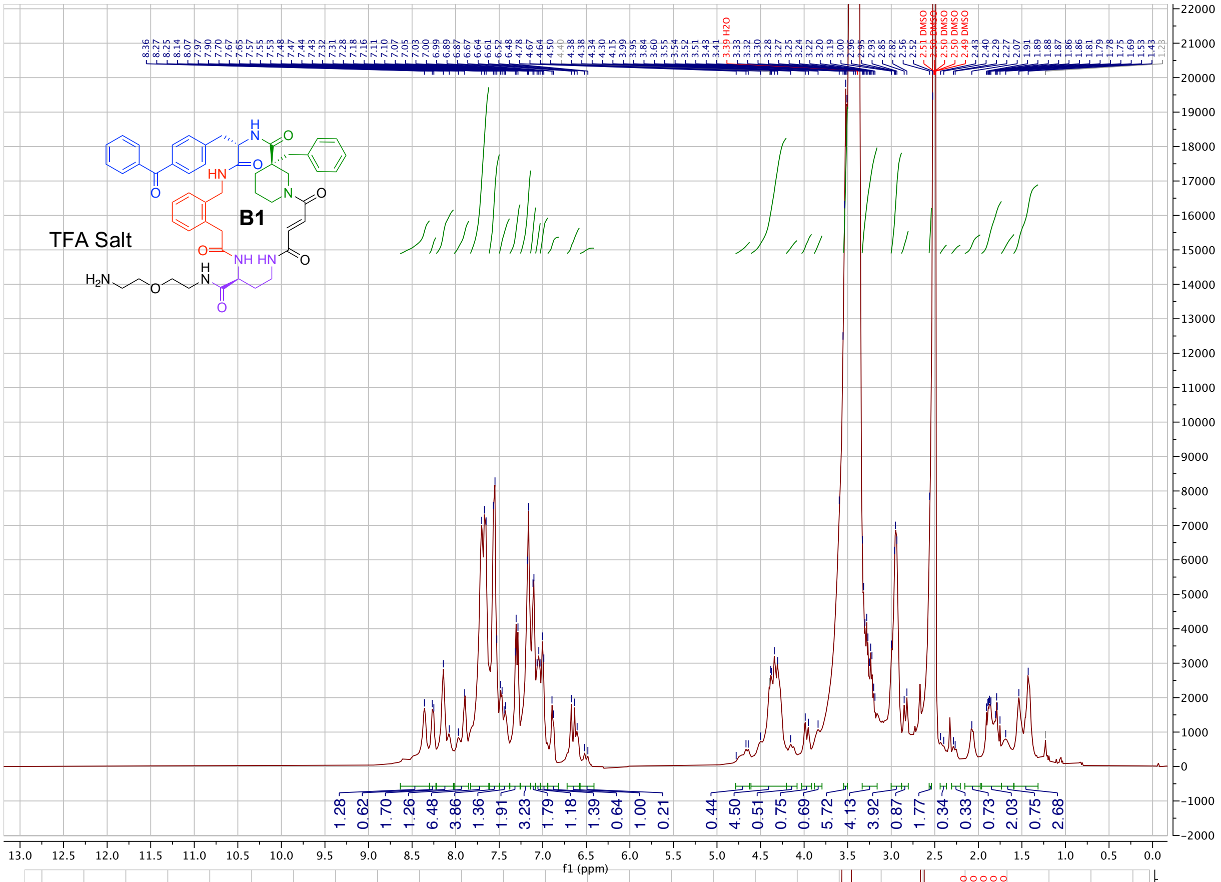
TFA Salt
B1
f1 (ppm)

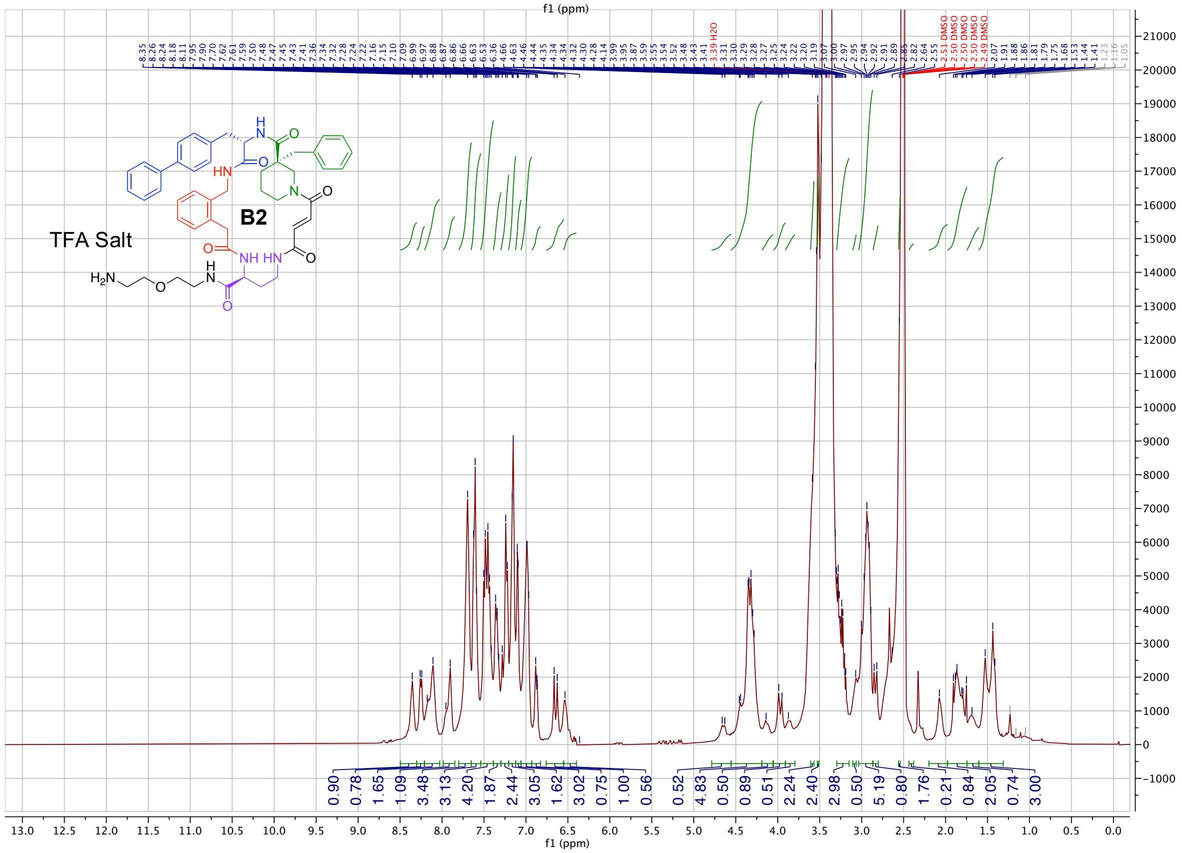
TFA Salt
B2
f1 (ppm)

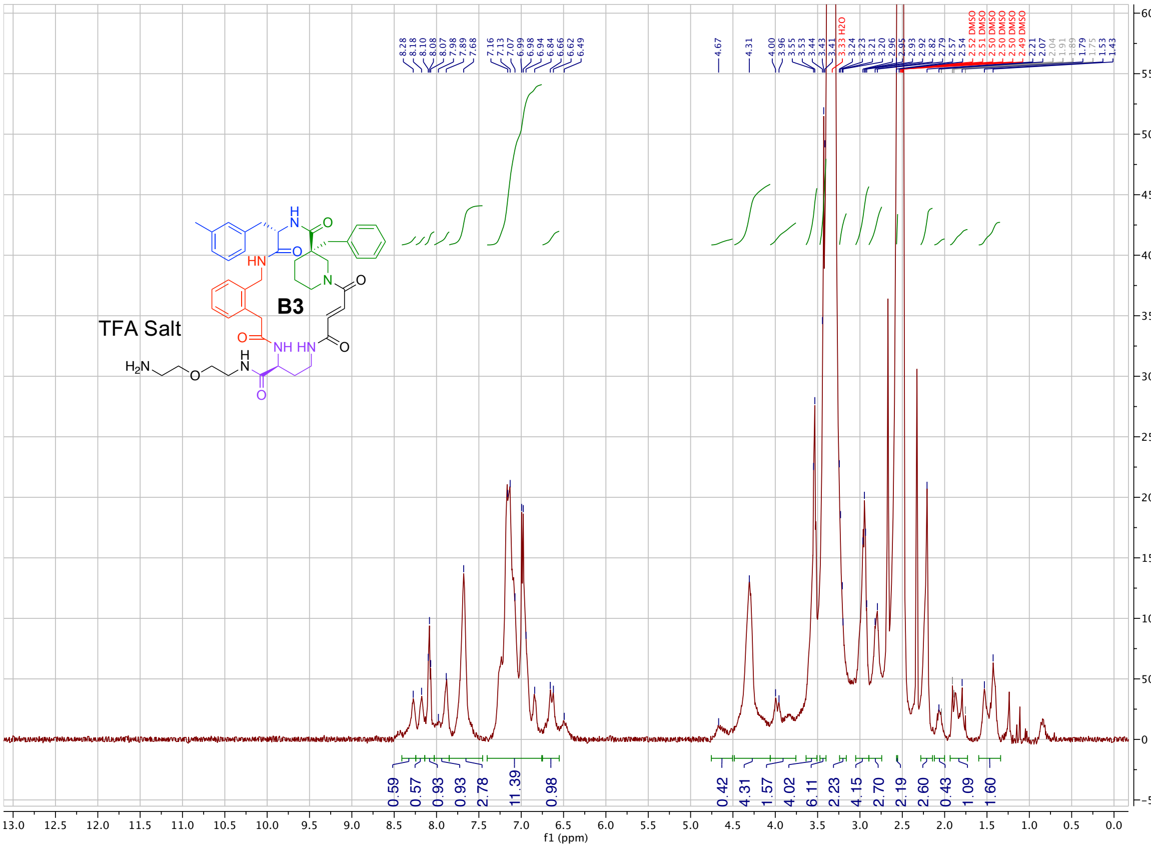
TFA Salt
B3
f1 (ppm)

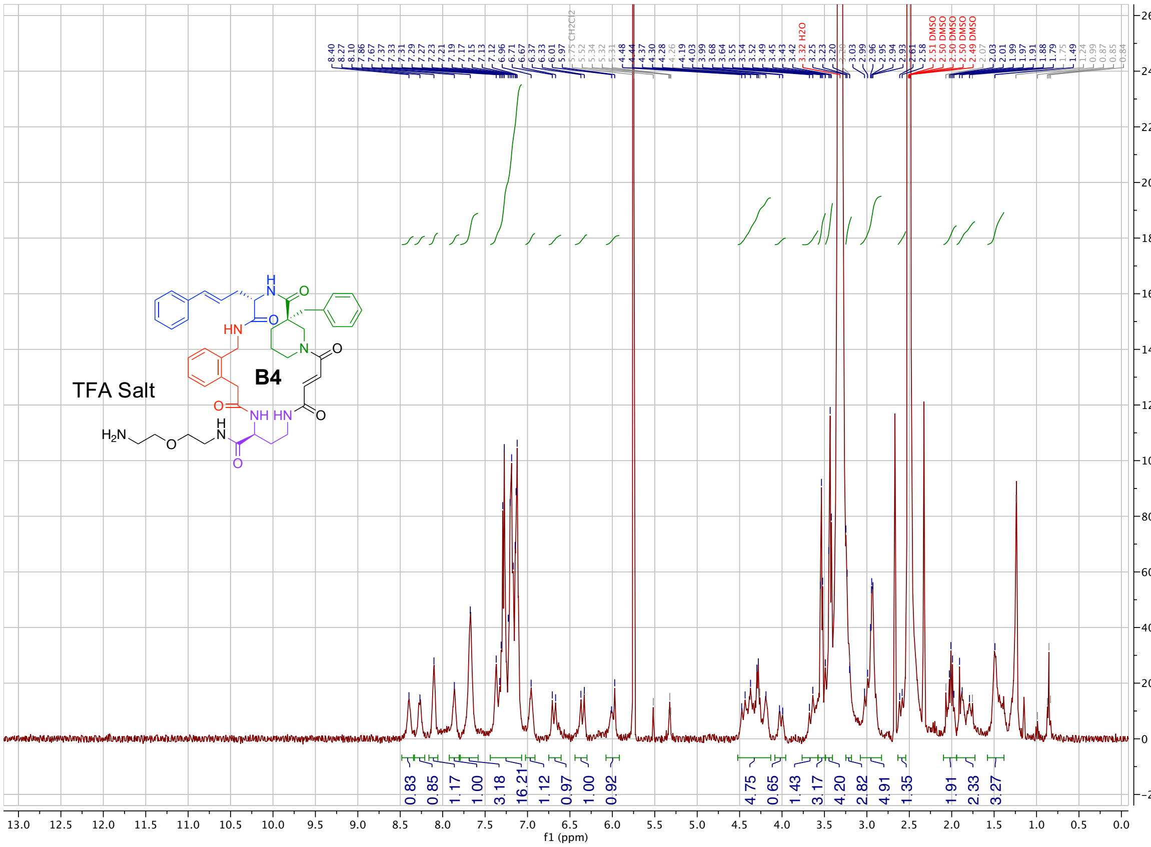
TFA Salt
B4
f1 (ppm)

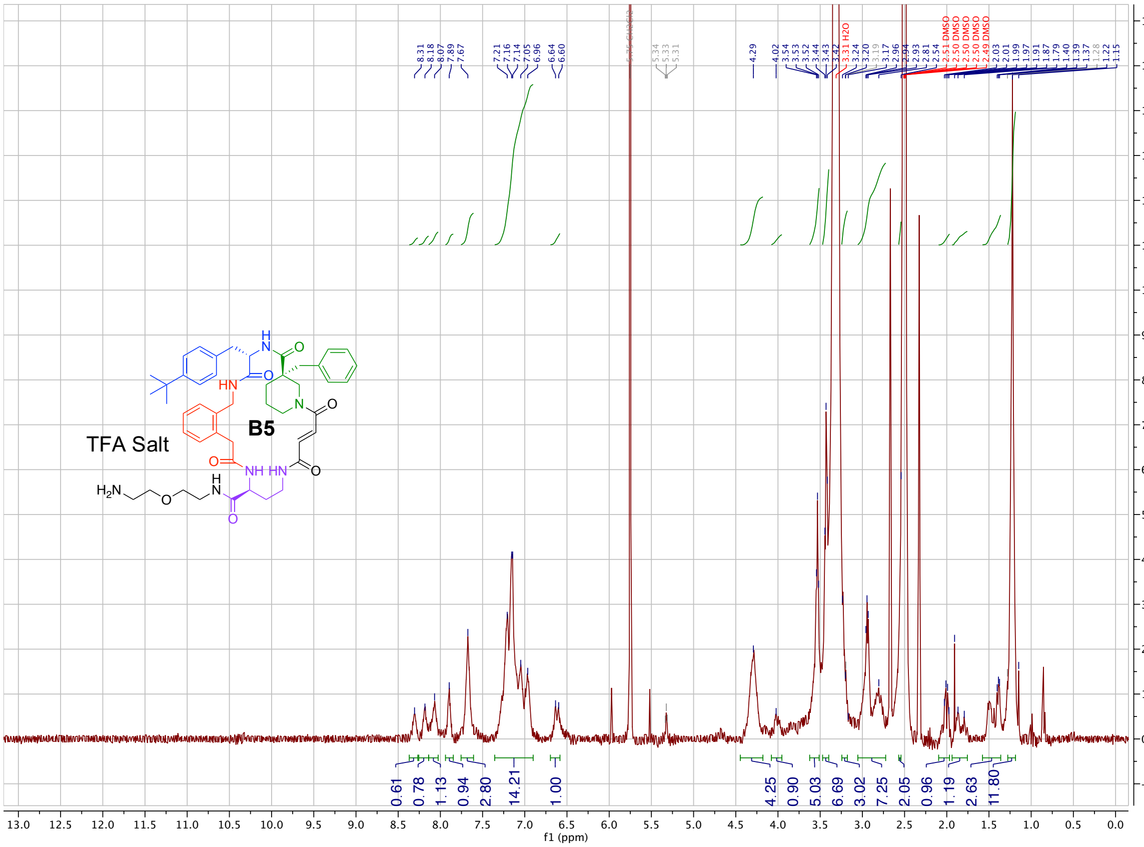
TFA Salt
B5
f1 (ppm)

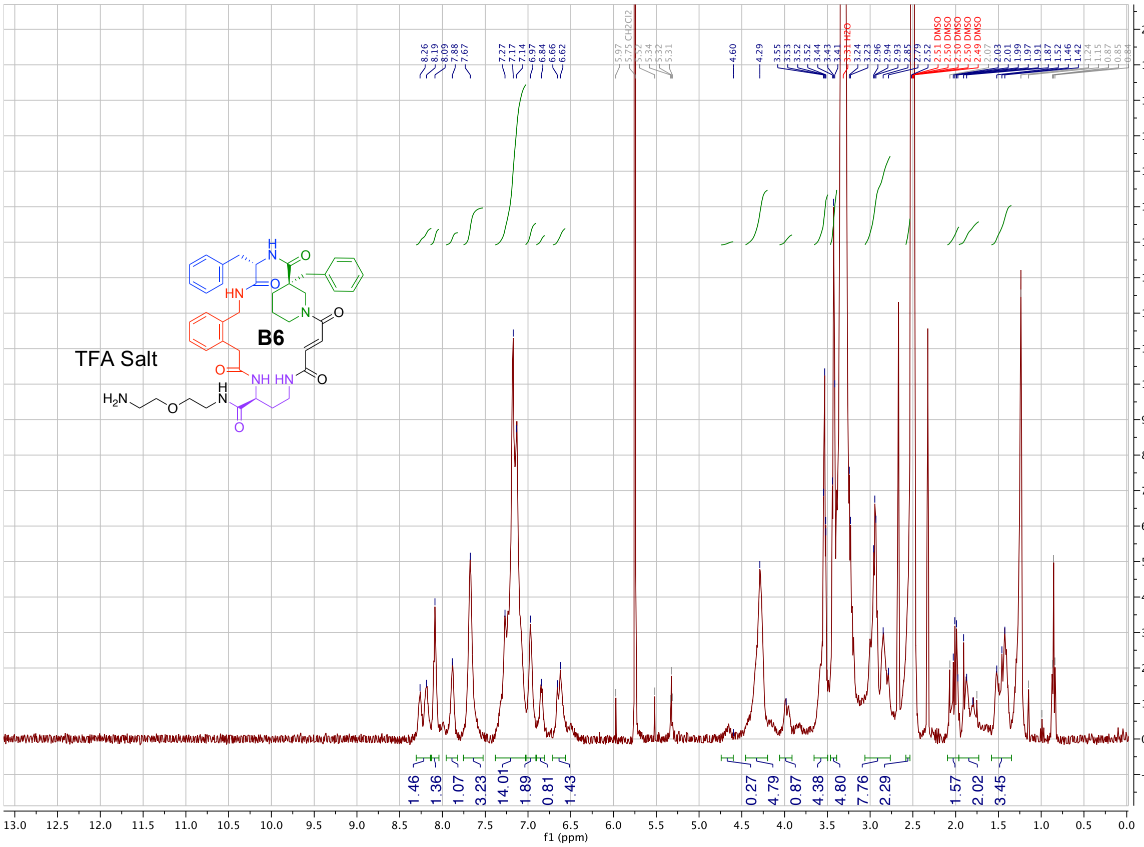
TFA Salt
B6
f1 (ppm)

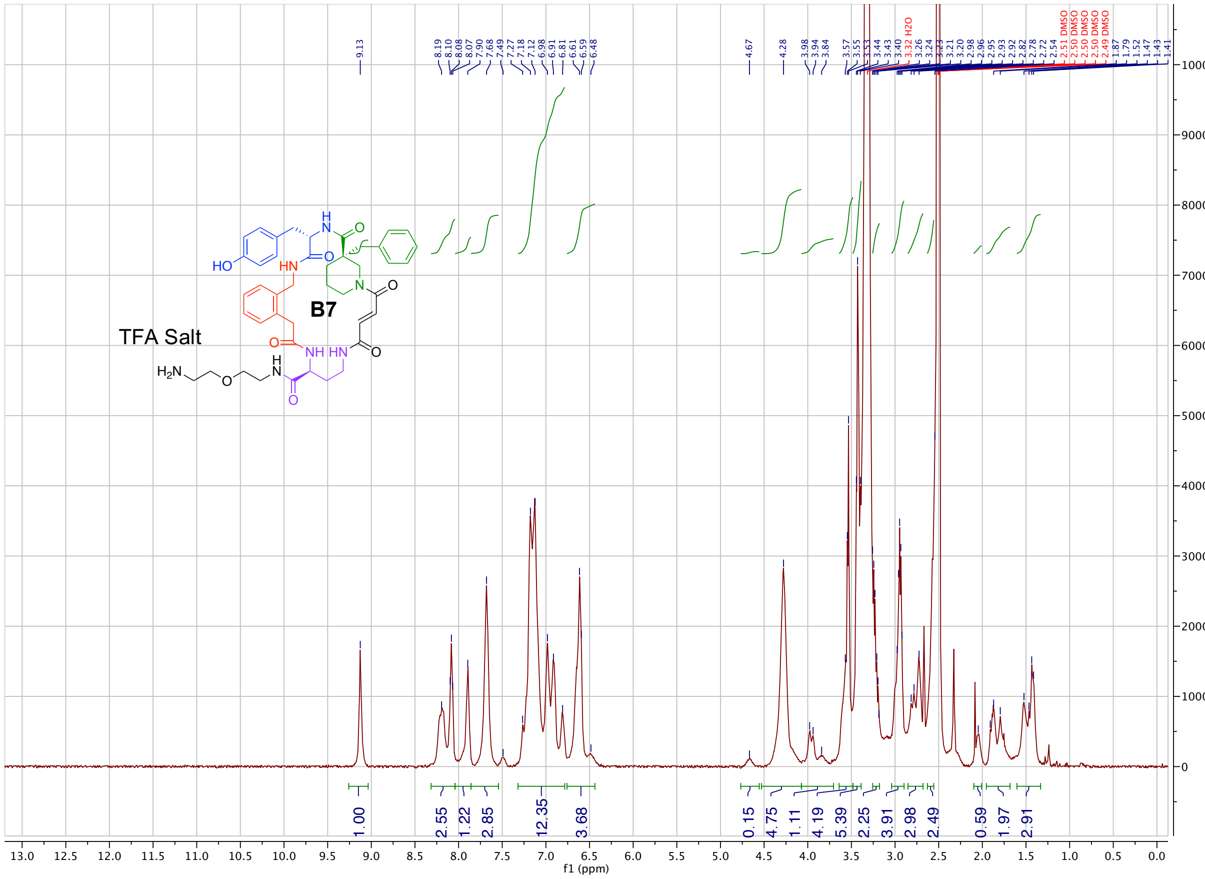

TFA Salt
B7
f1 (ppm)

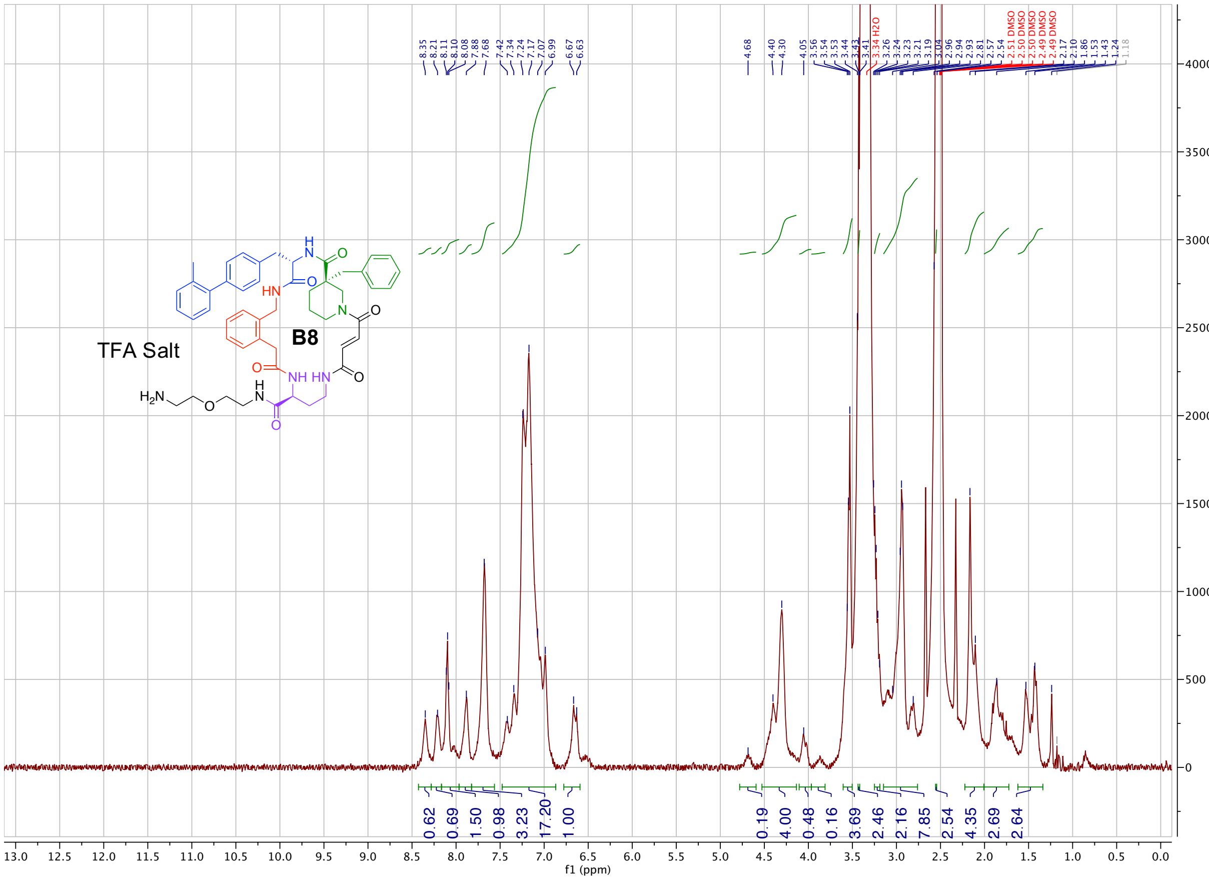

TFA Salt
B8
f1 (ppm)

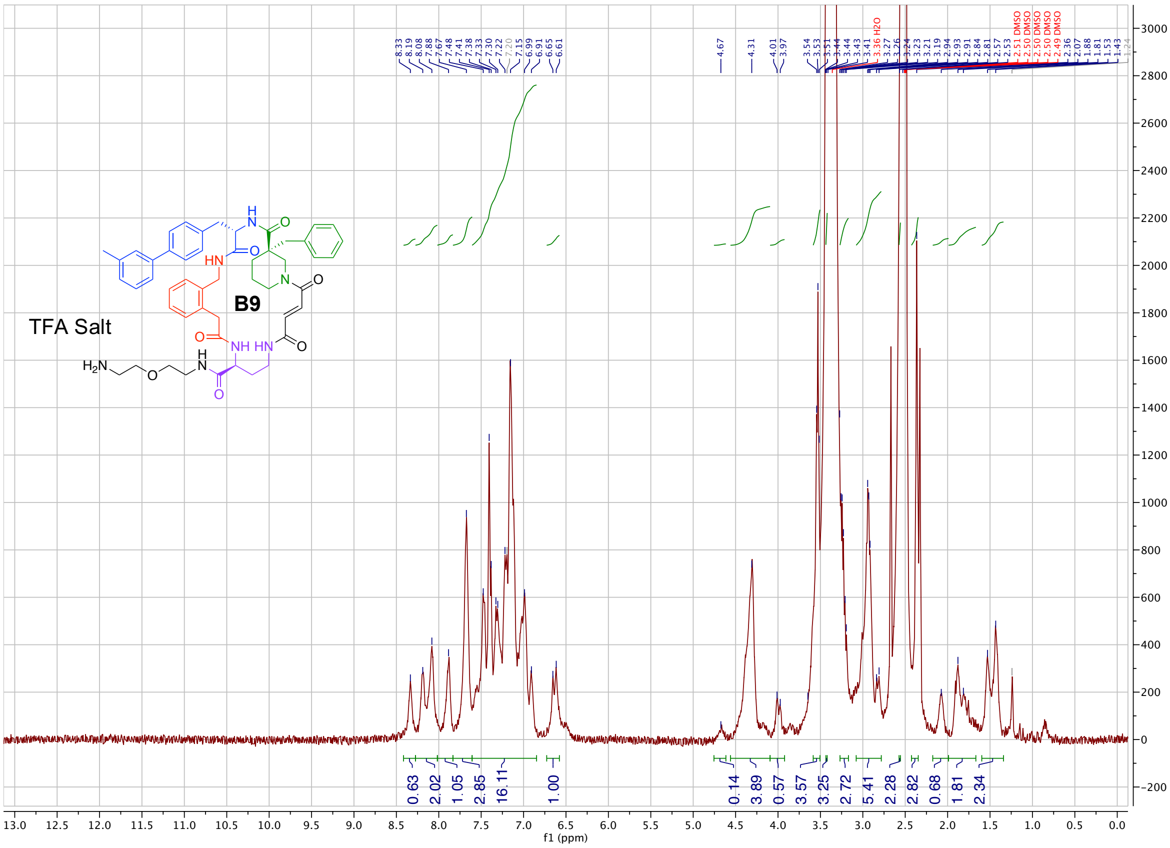
TFA Salt
B9
f1 (ppm)

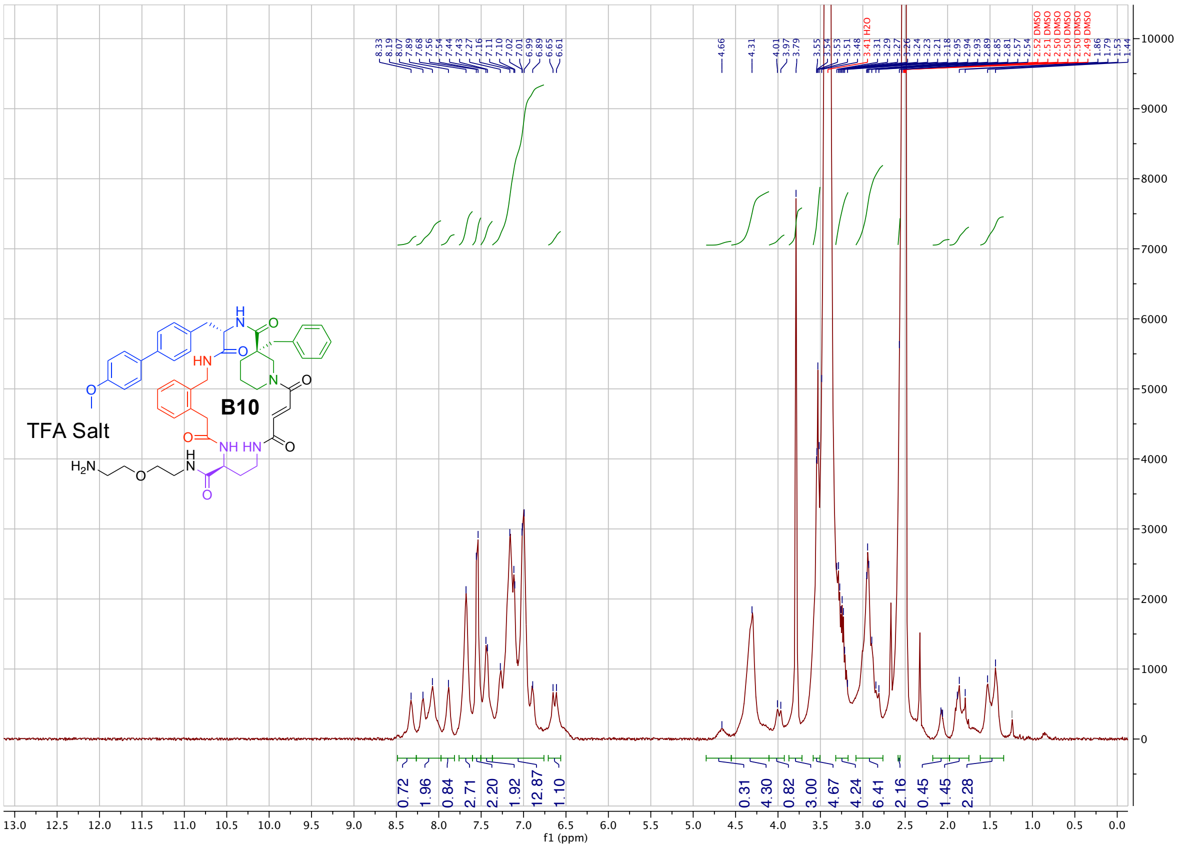
TFA Salt
B10
f1 (ppm)

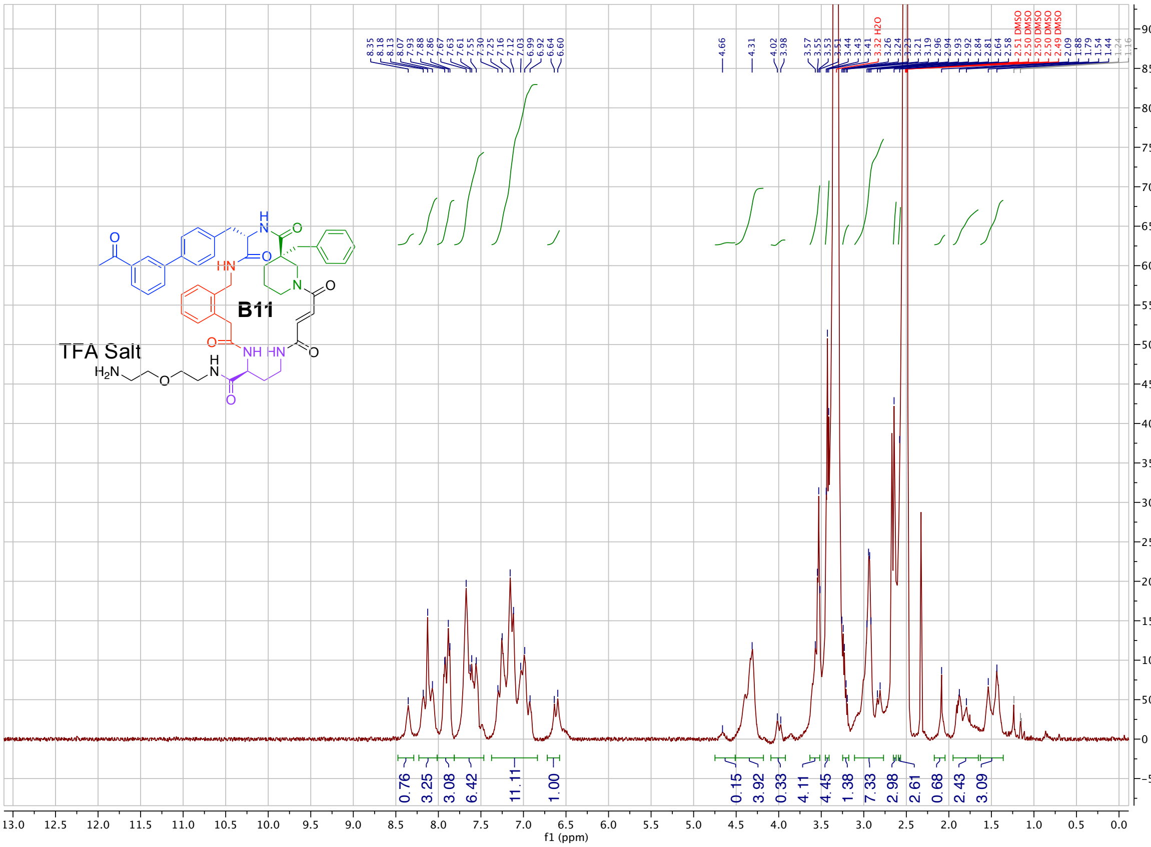
TFA Salt
B11
f1 (ppm)

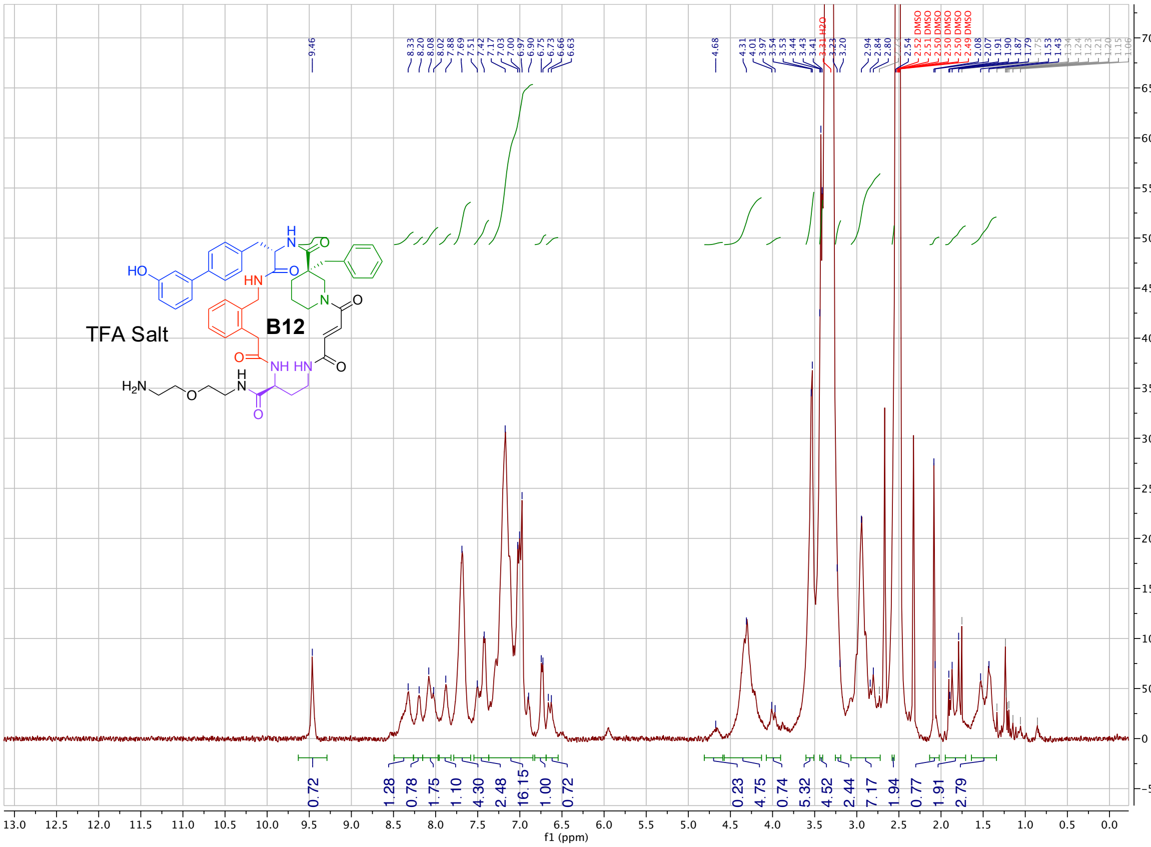
TFA Salt
B12
f1 (ppm)

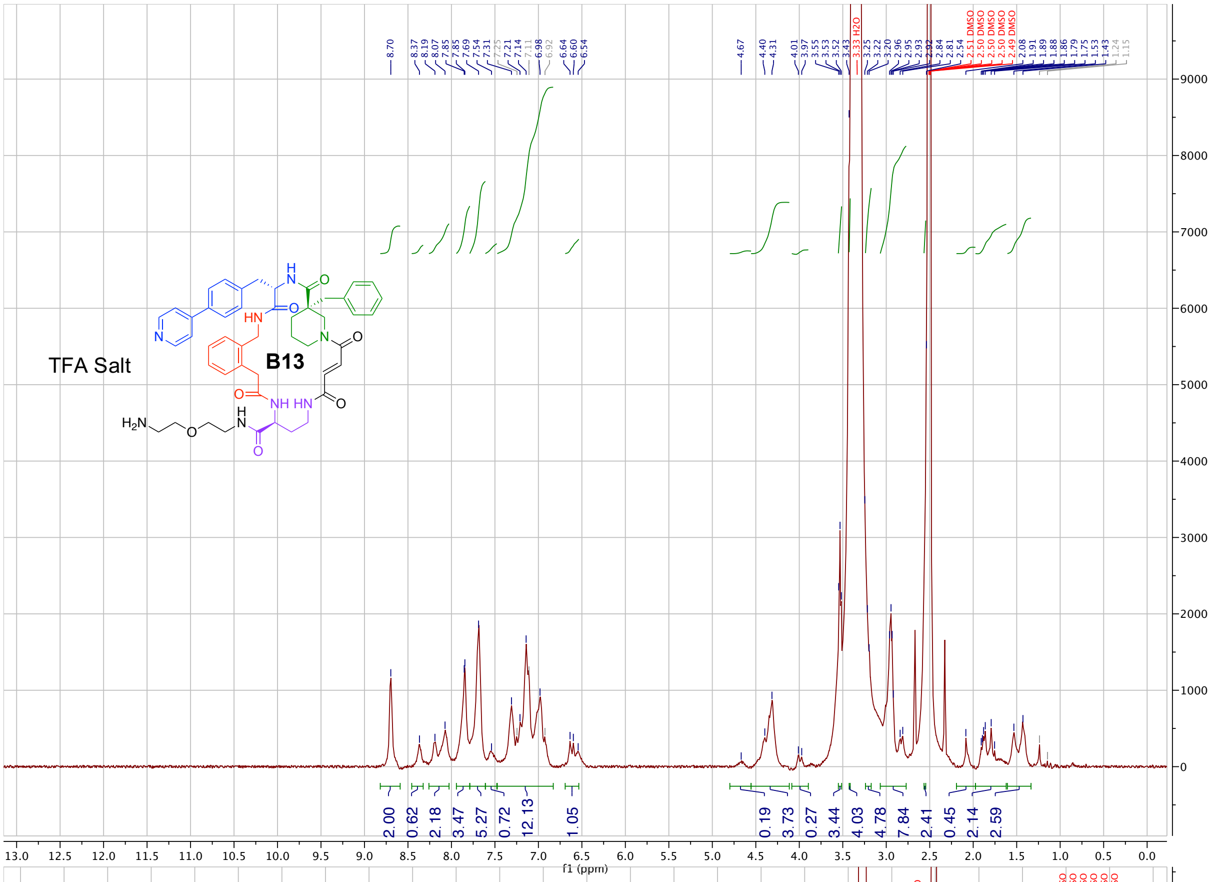
TFA Salt
B13
f1 (ppm)

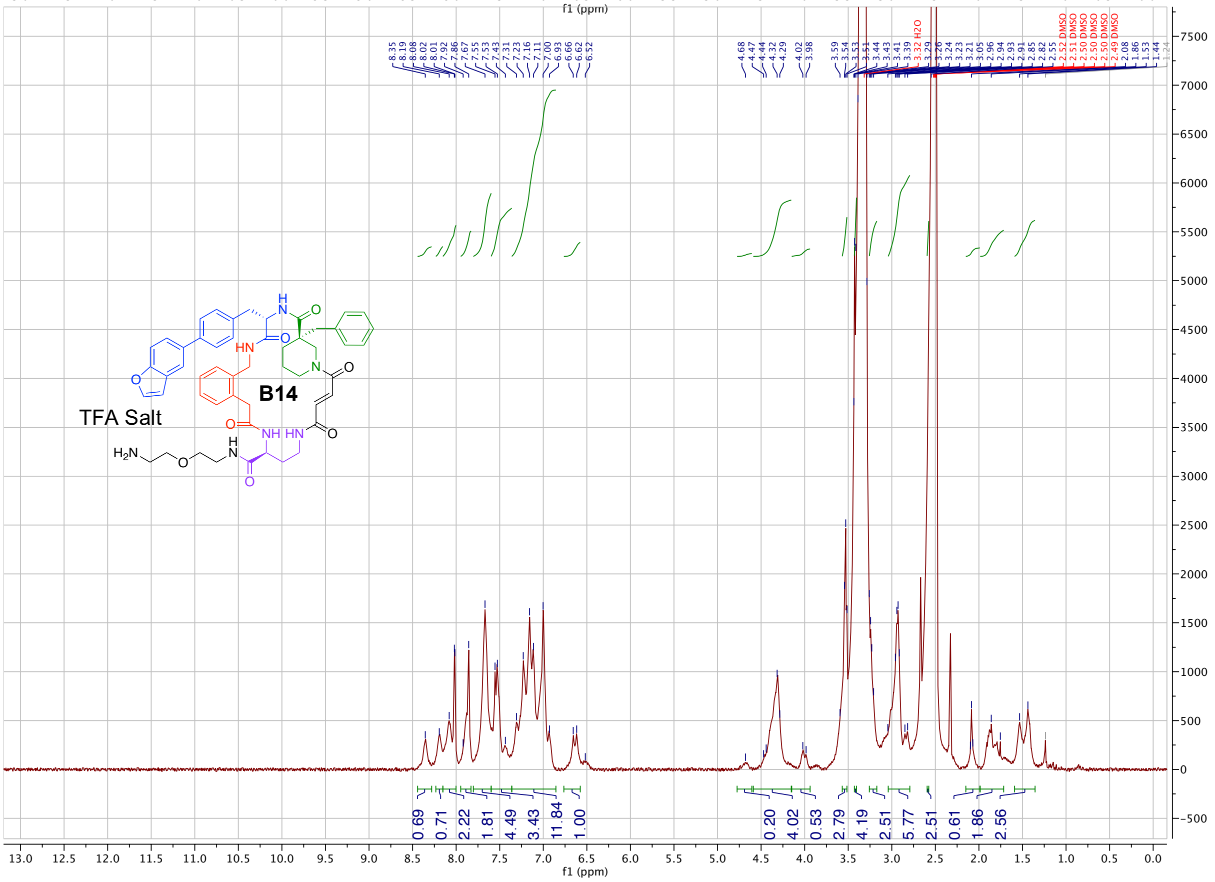
TFA Salt
B14
f1 (ppm)

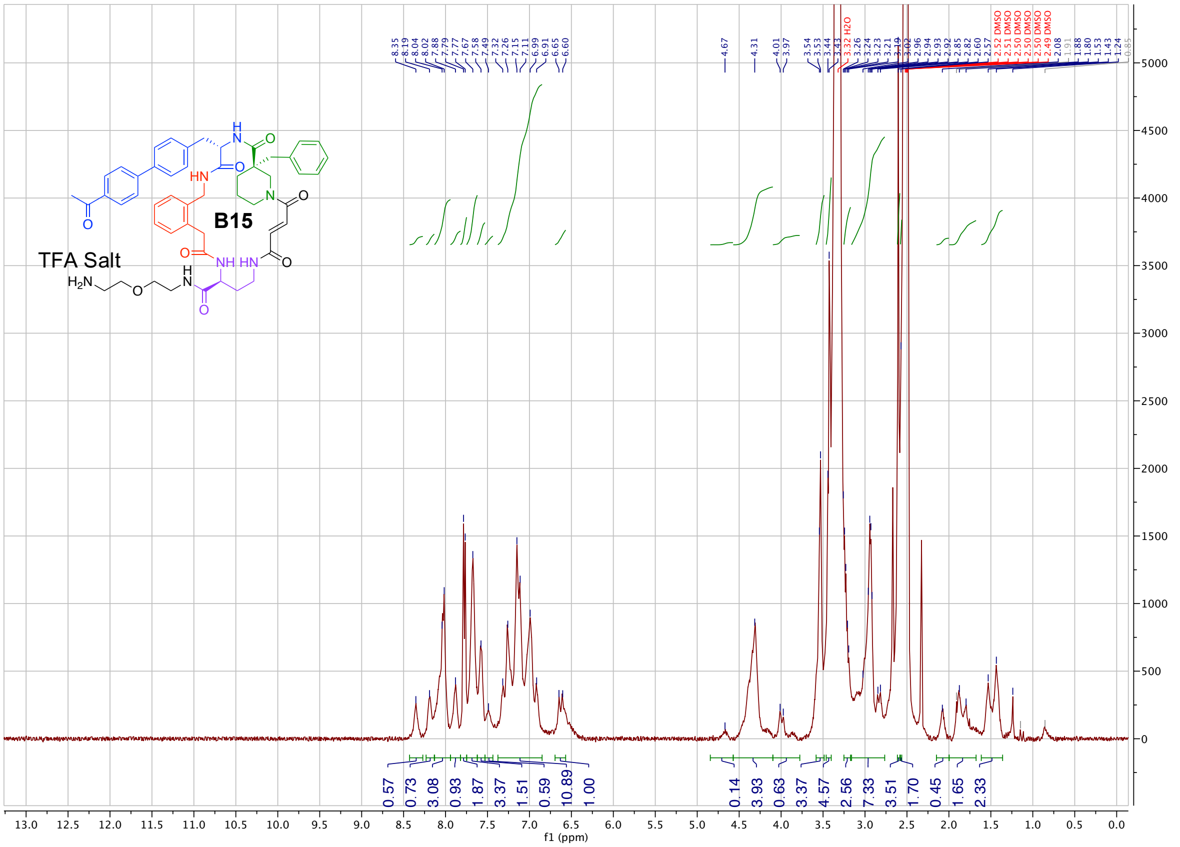
TFA Salt
B15
0.57
0.73
3.08
0.93
1.87
3.37
1.51
0.59
10.89
1.00
0.14
3.93
0.63
3.37
4.57
2.56
7.33
3.51
1.70
0.45
1.65
2.33
f1 (ppm)

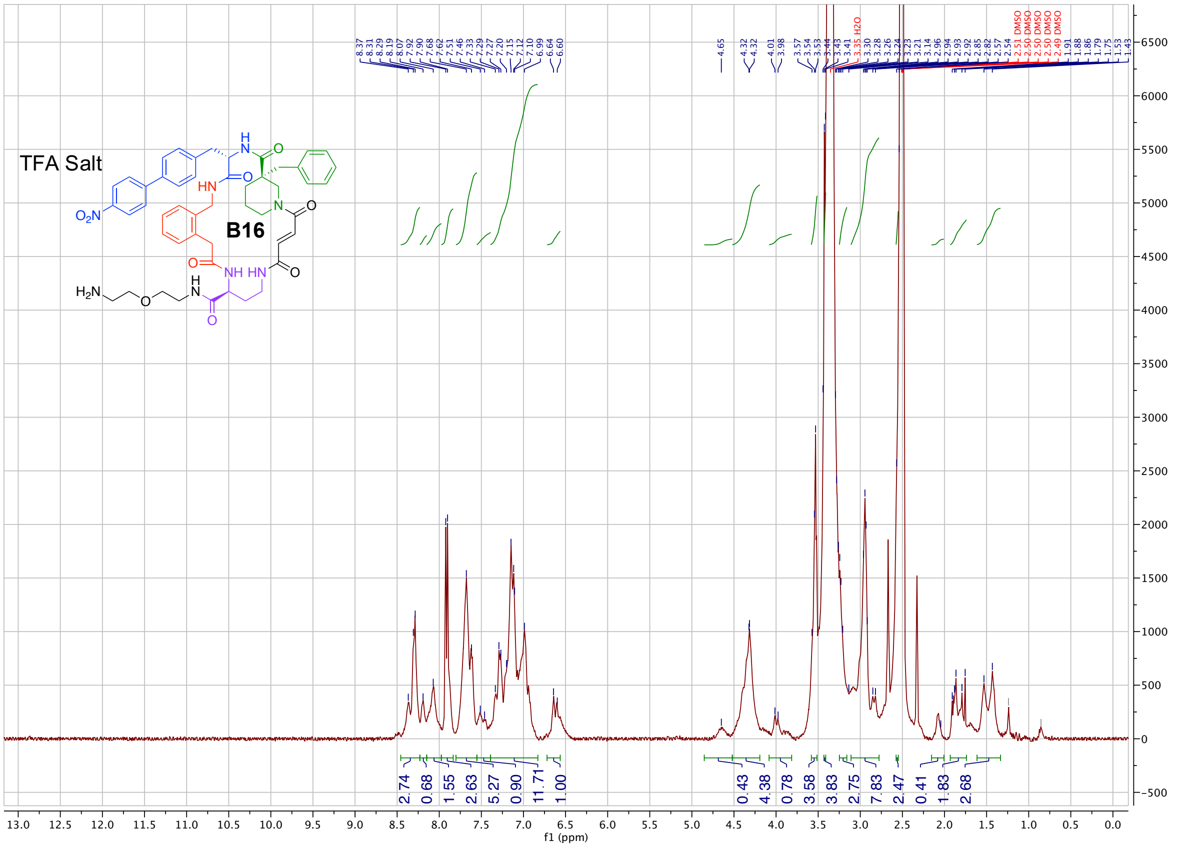
TFA Salt
B16
2.74
0.68
1.55
2.63
5.27
0.90
11.71
1.00
0.43
4.38
0.78
3.58
3.83
2.75
7.83
2.47
0.41
1.83
2.68
f1 (ppm)

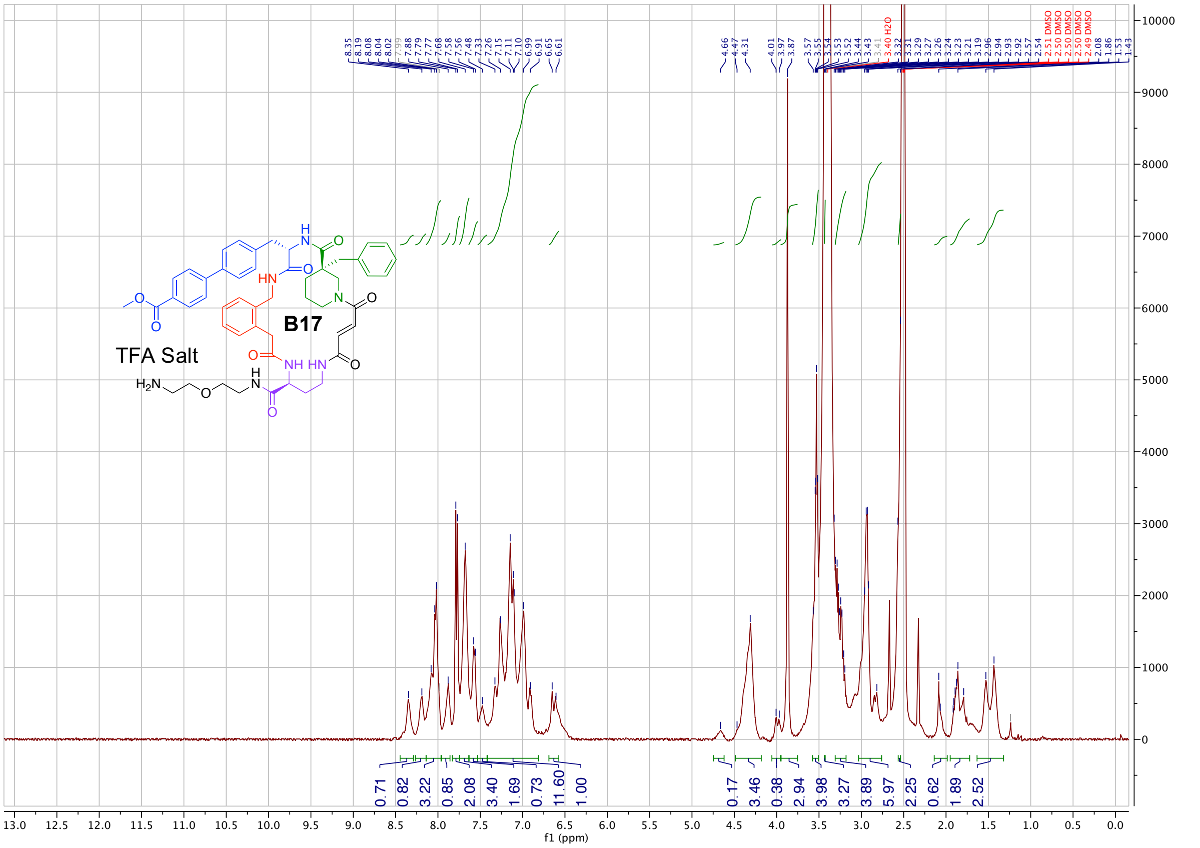
B17
TFA Salt
f1 (ppm)

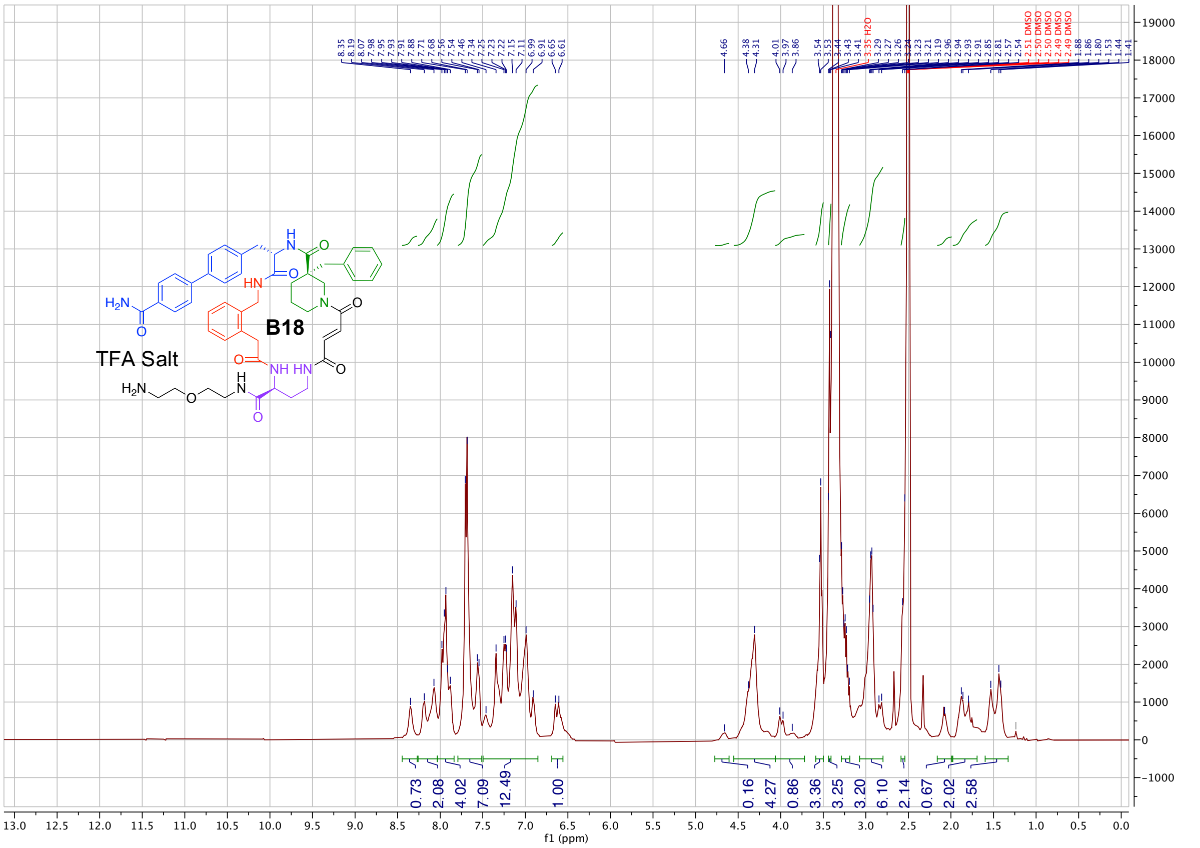
B18
TFA Salt
f1 (ppm)

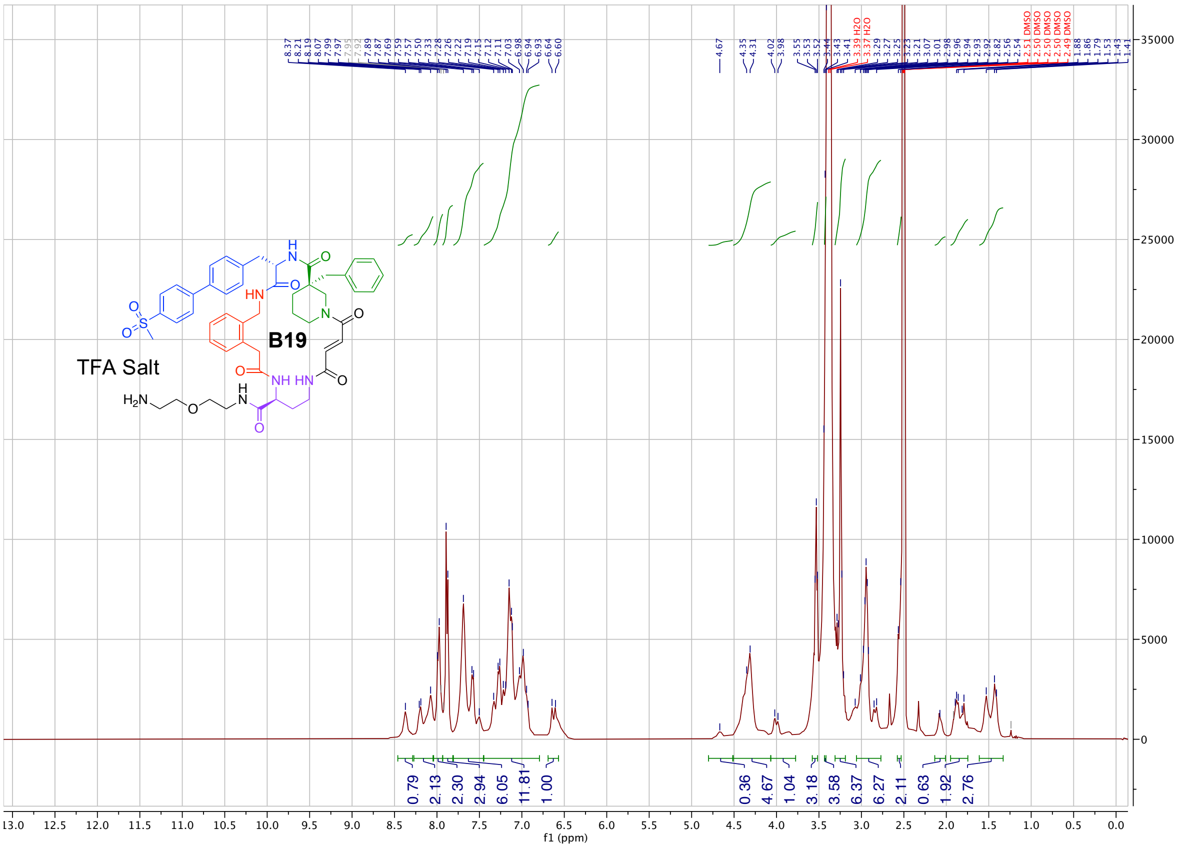
TFA Salt
B19
f1 (ppm)

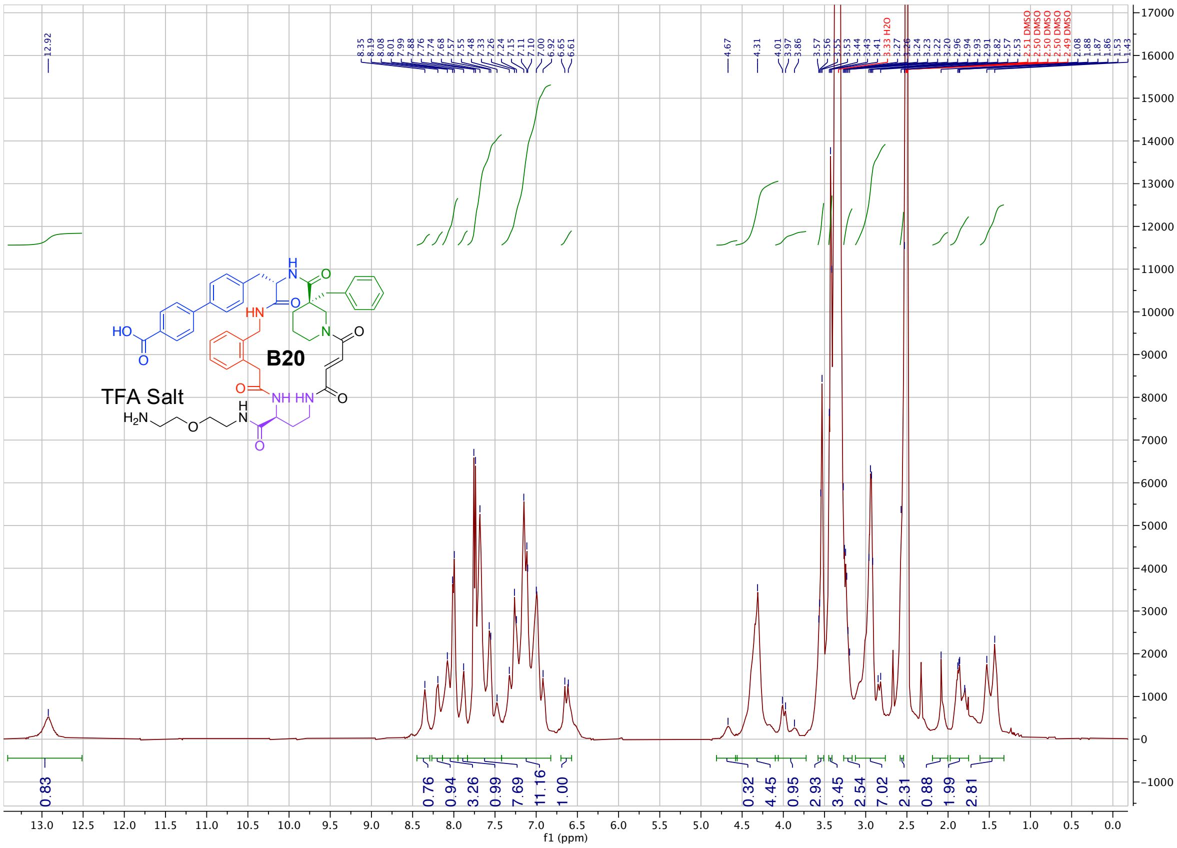
TFA Salt
B20
f1 (ppm)

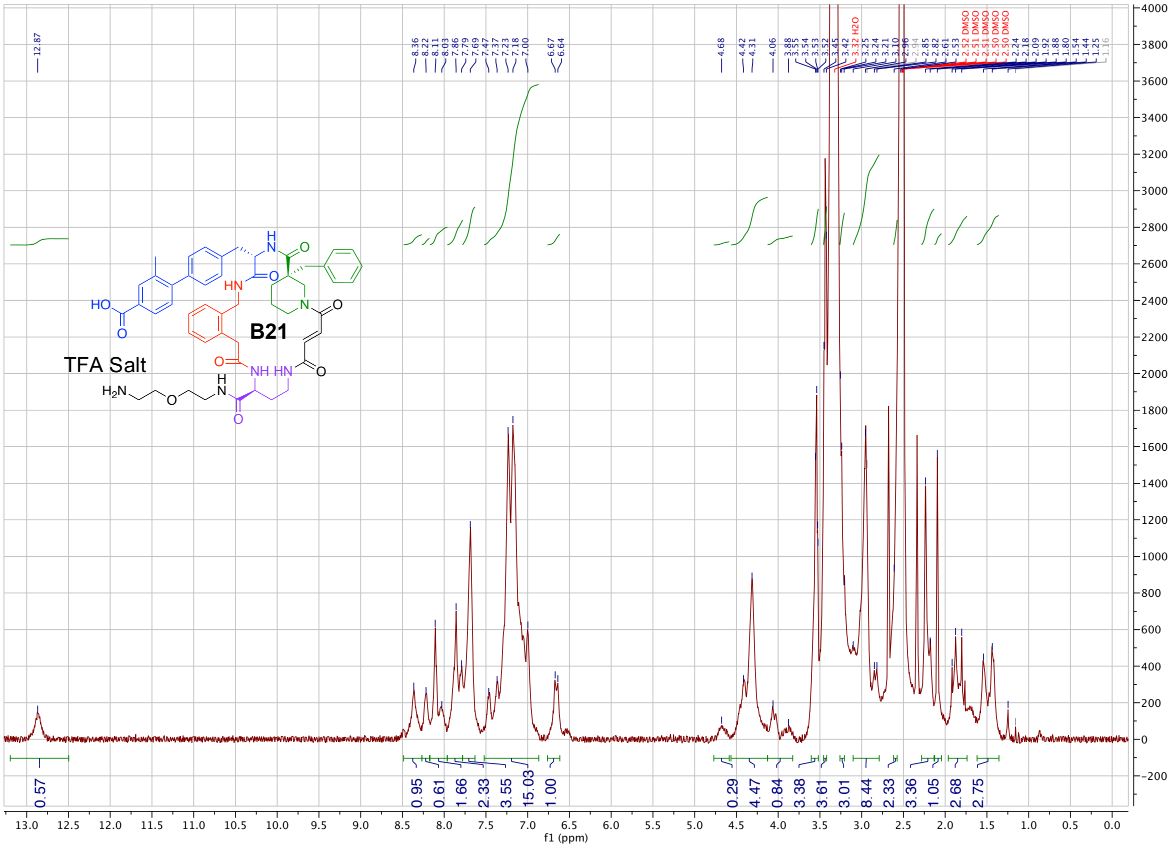

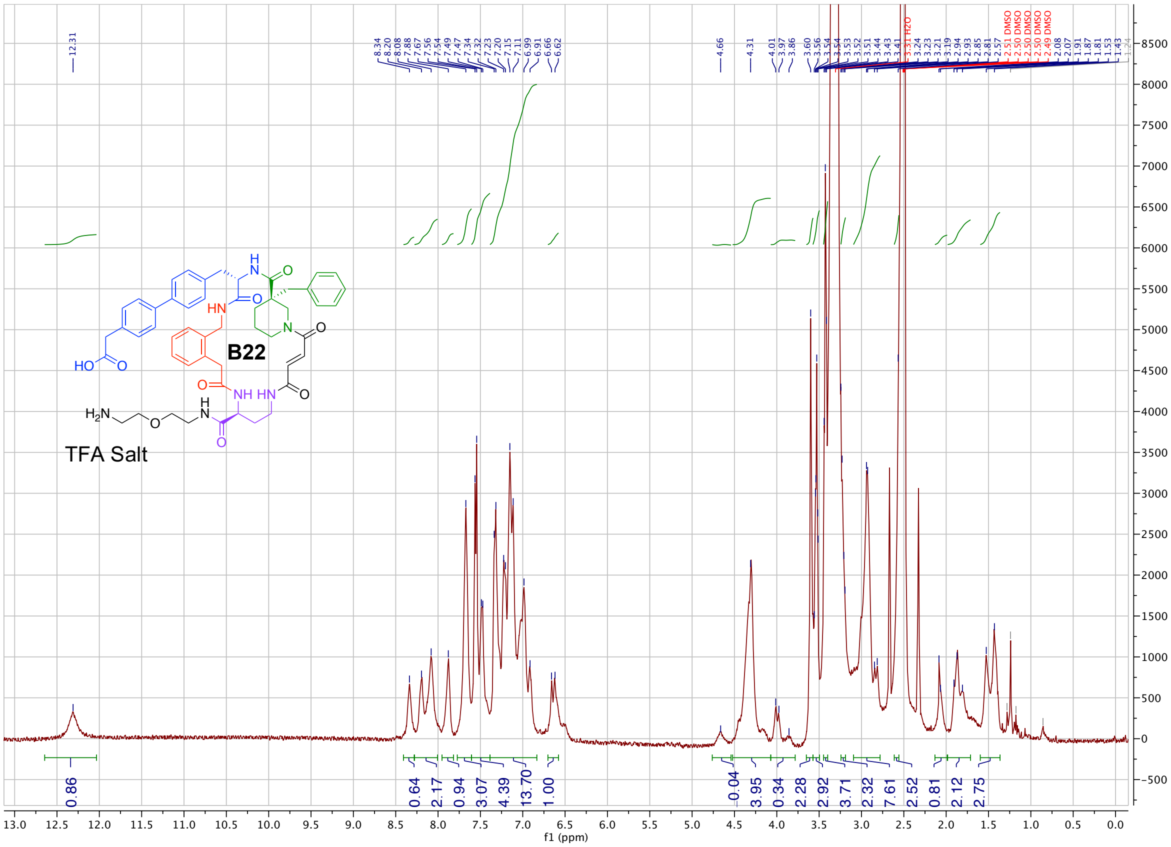

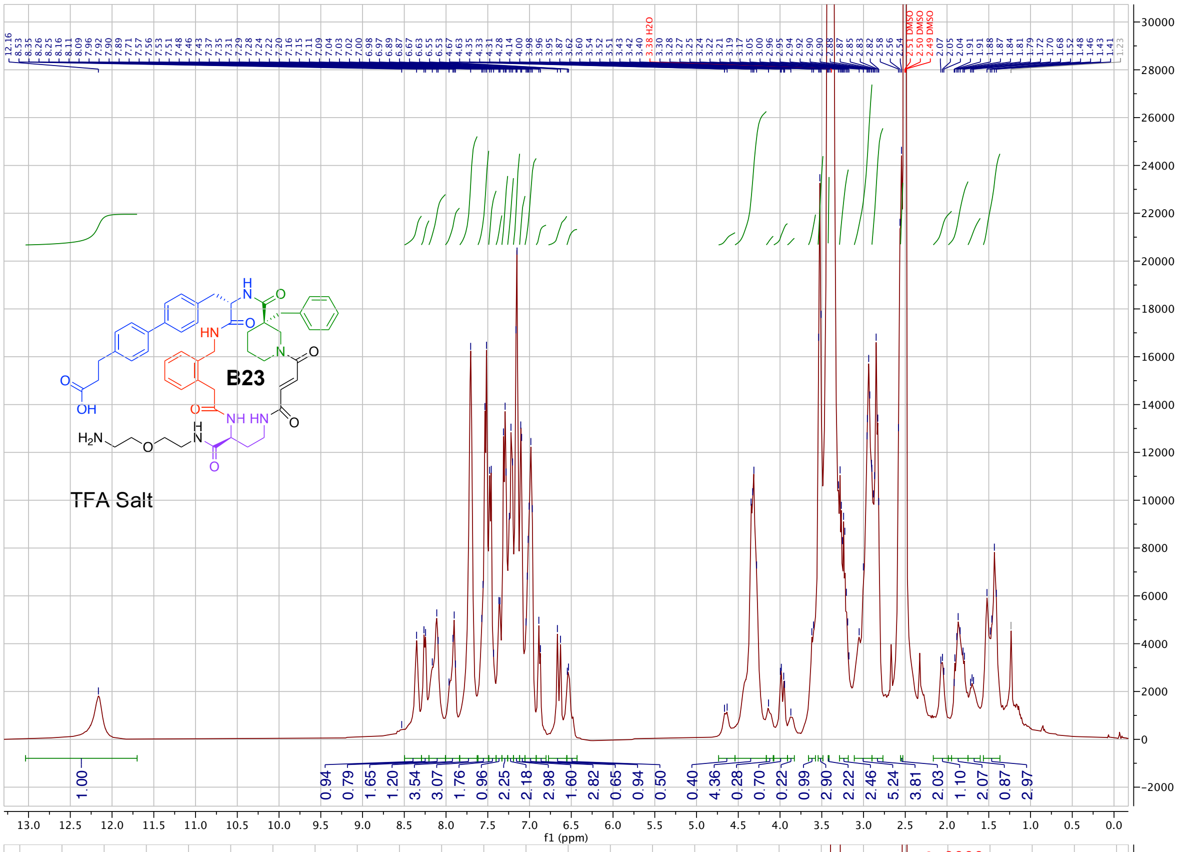
B23
TFA Salt
f1 (ppm)

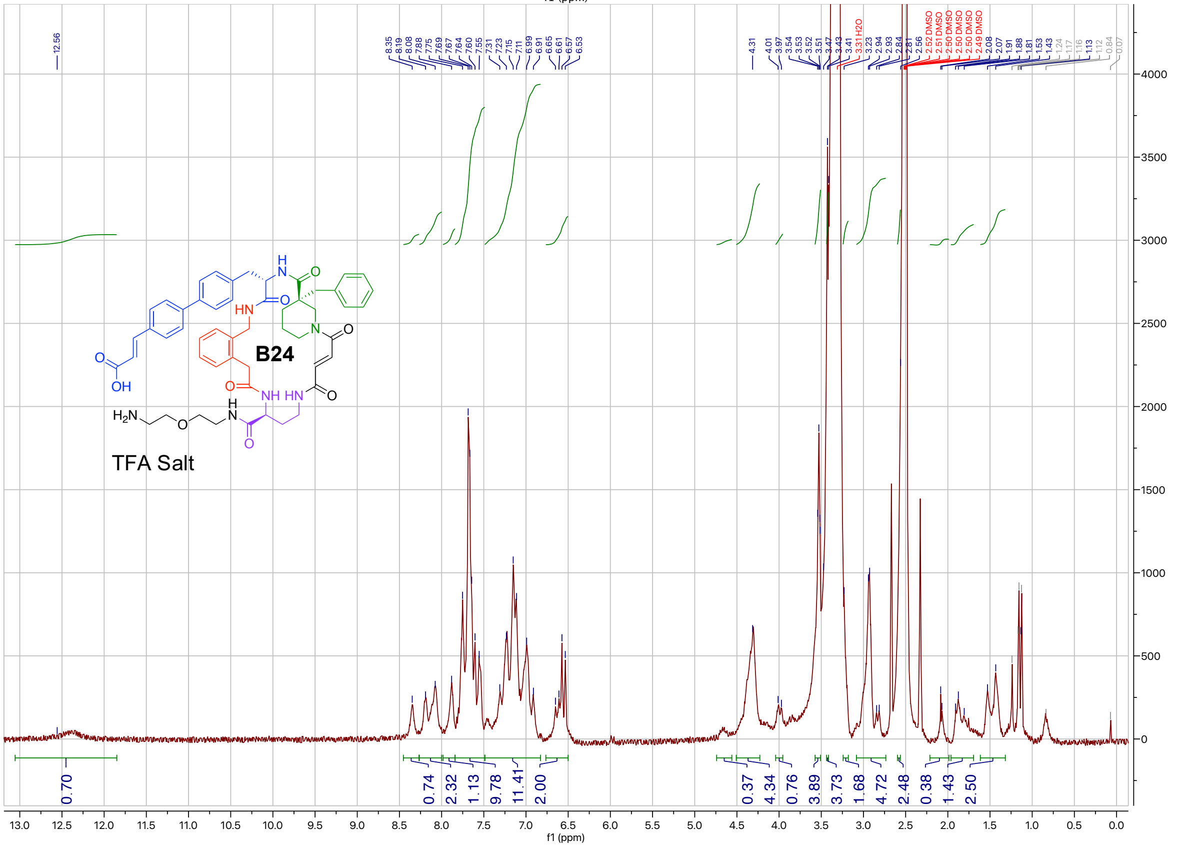
B24
TFA Salt
f1 (ppm)

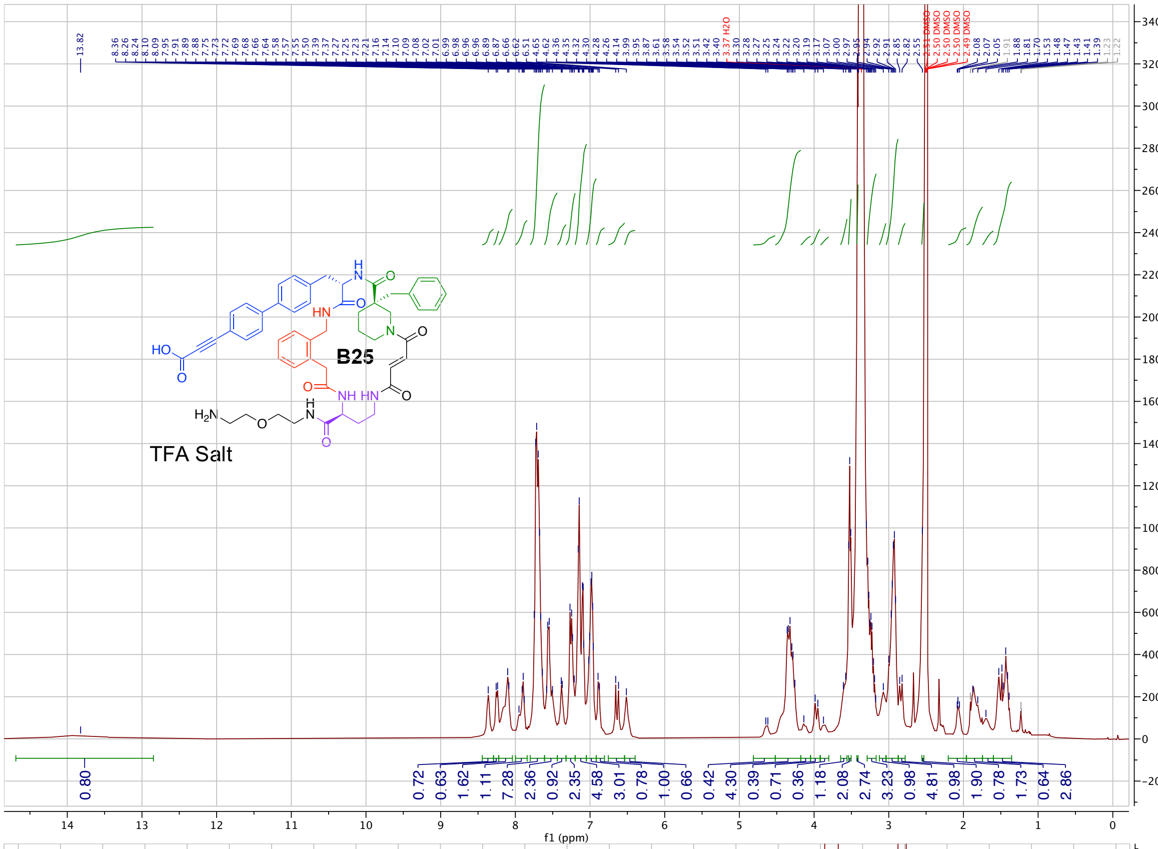
B25
TFA Salt
f1 (ppm)

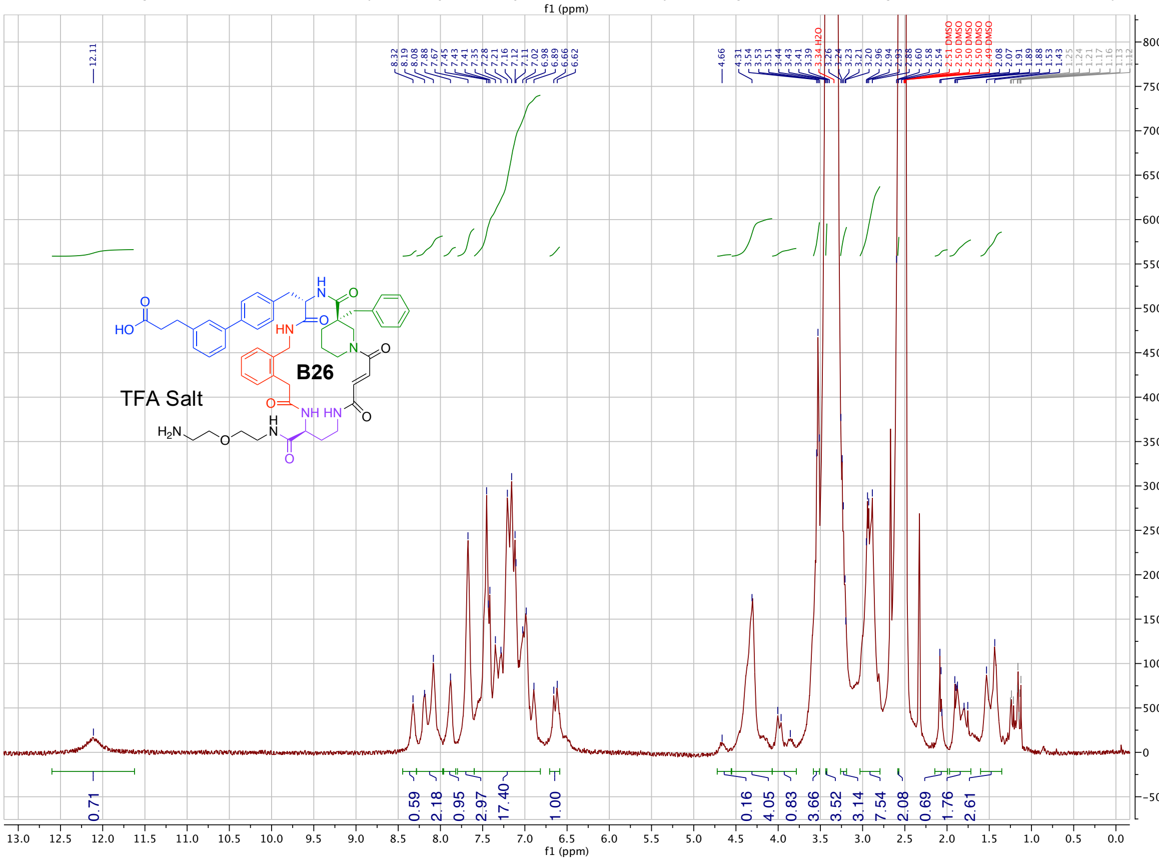
B26
TFA Salt
f1 (ppm)

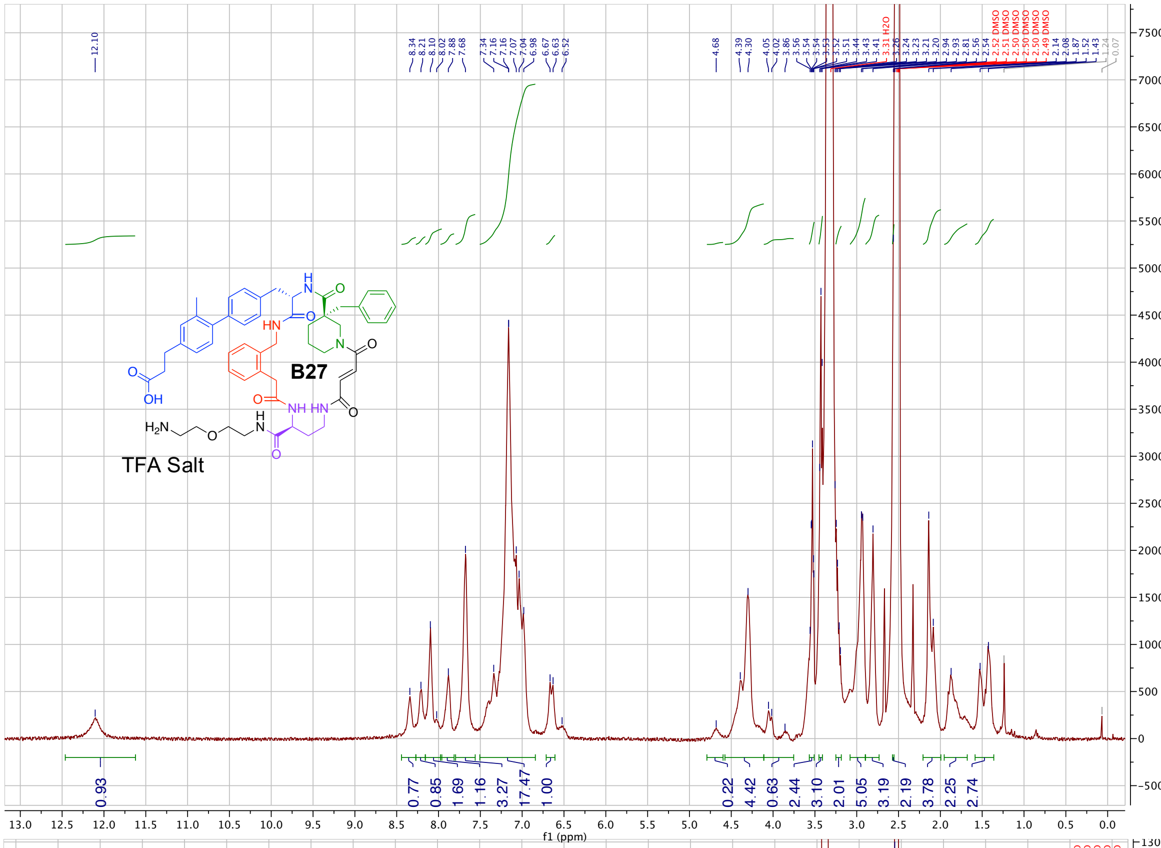
B27
TFA Salt
f1 (ppm)

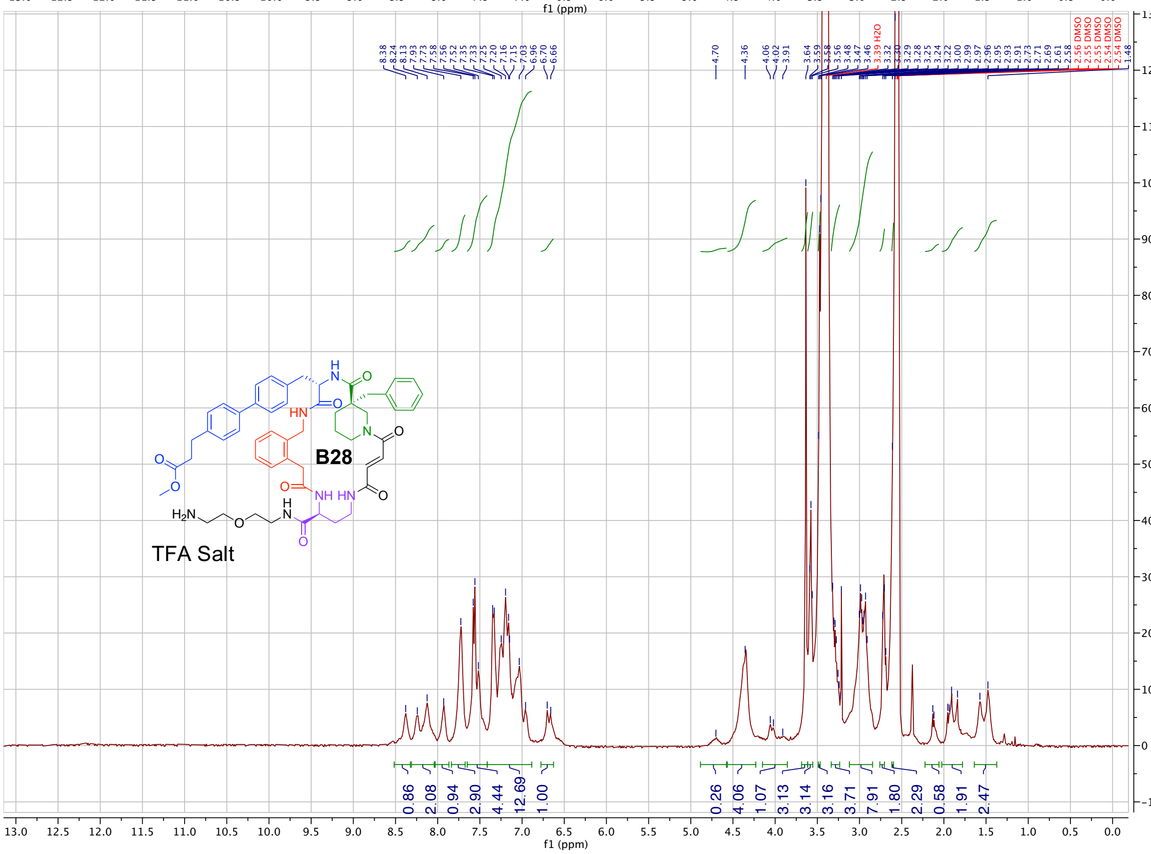
B28
TFA Salt
f1 (ppm)

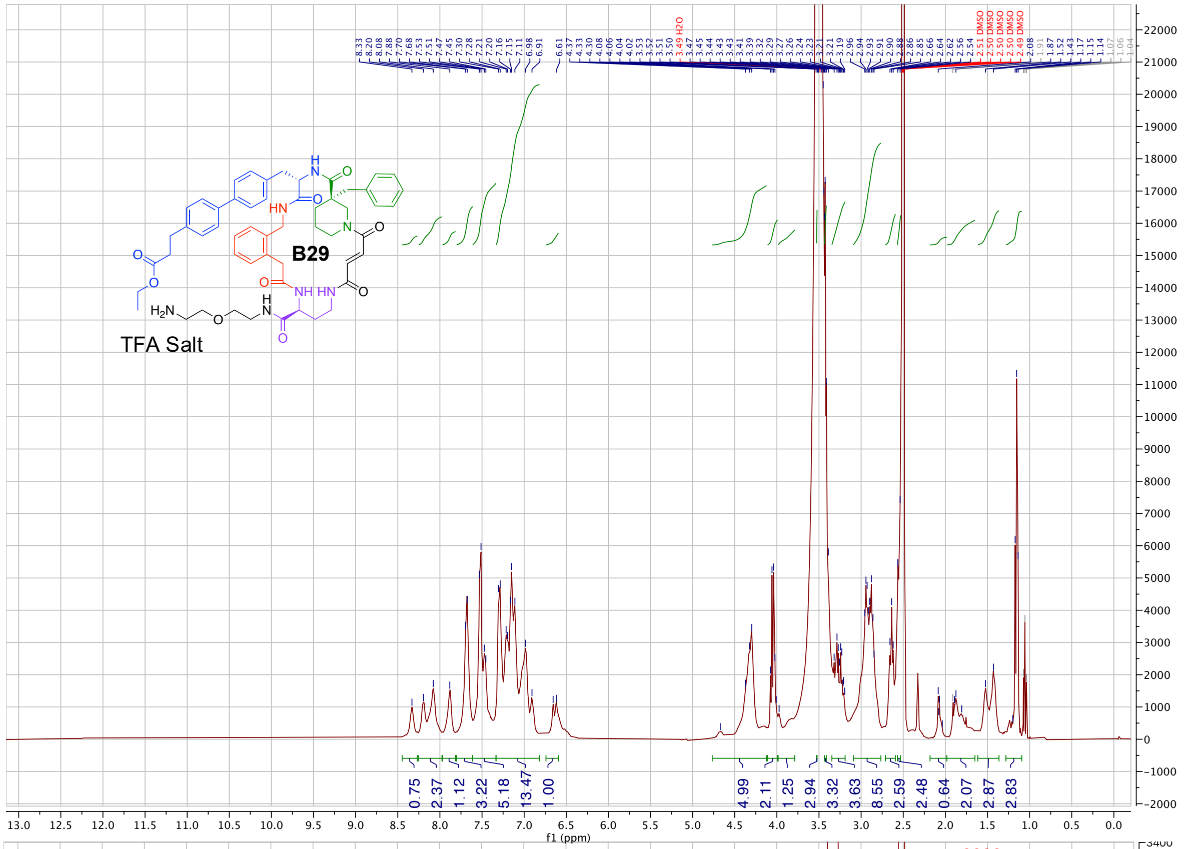
B29
TFA Salt
f1 (ppm)

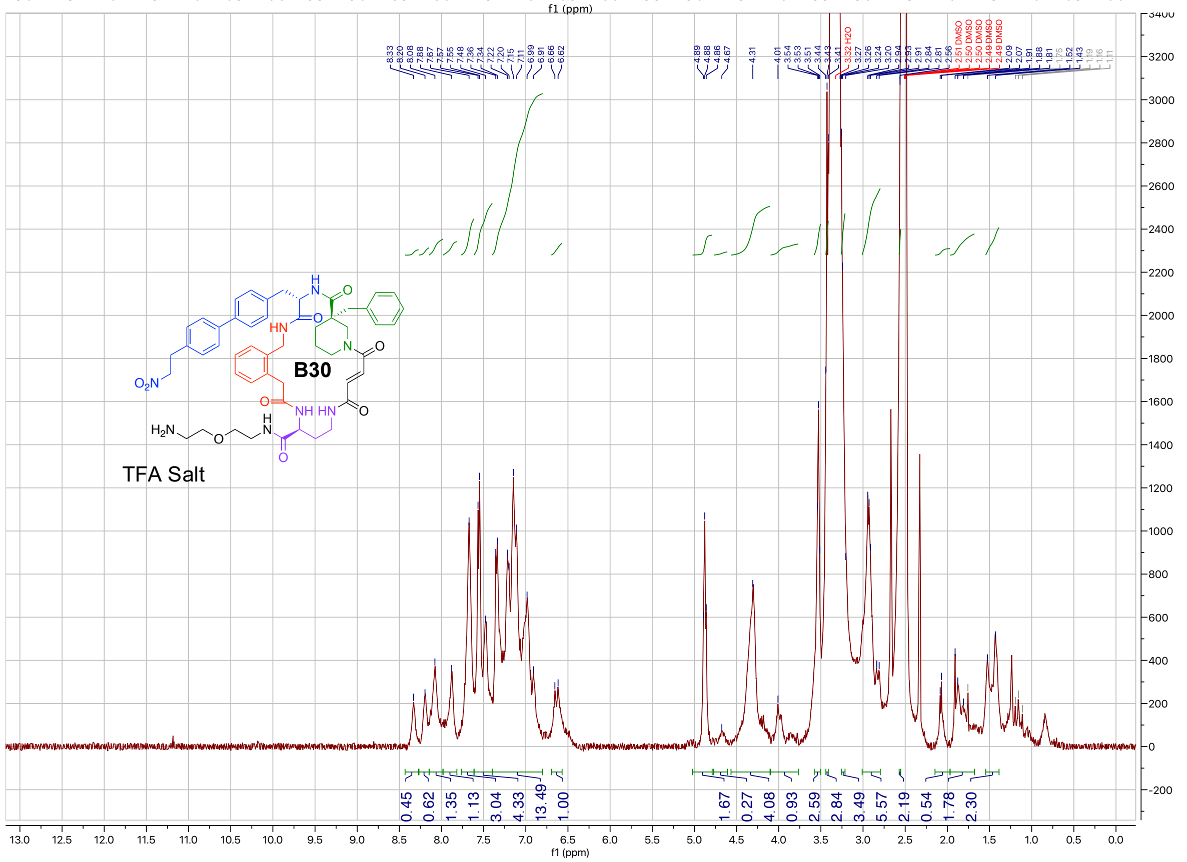
B30
TFA Salt
f1 (ppm)

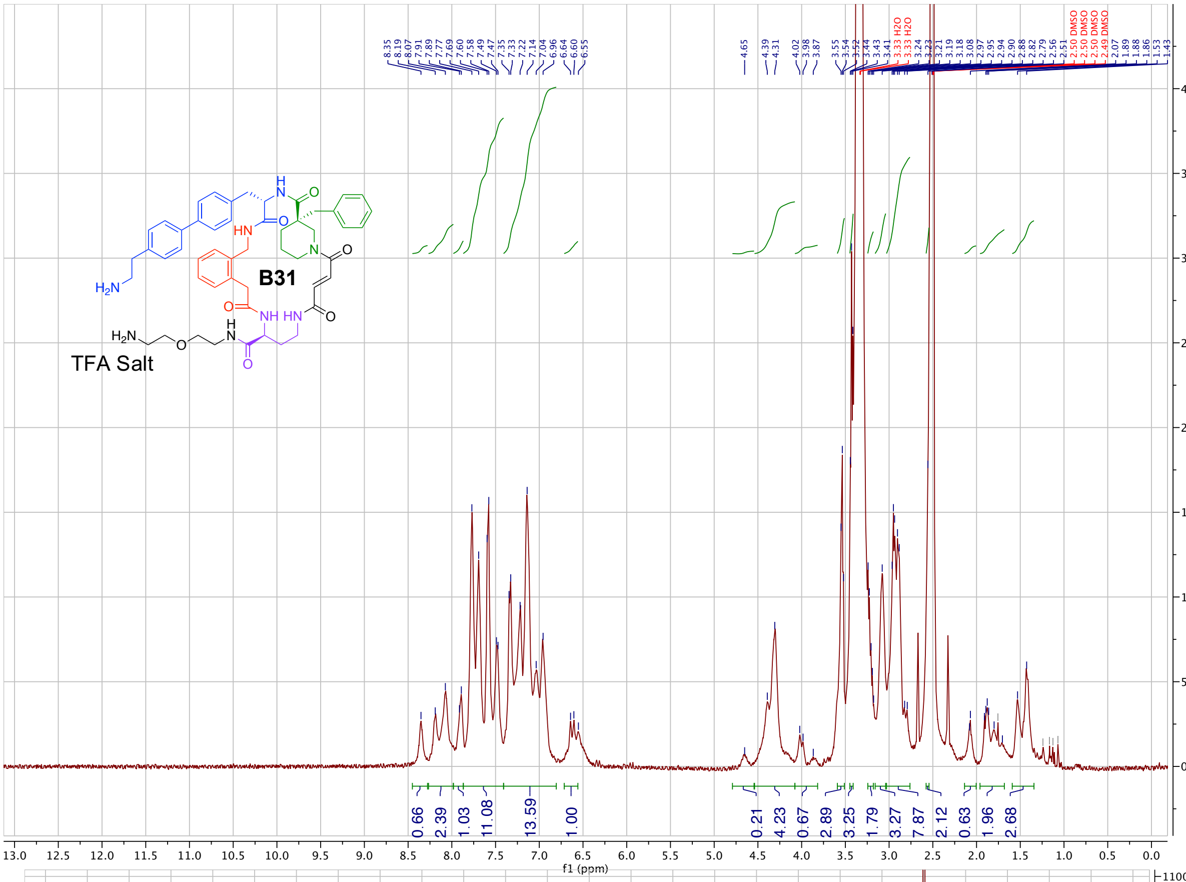
B31
TFA Salt
f1 (ppm)

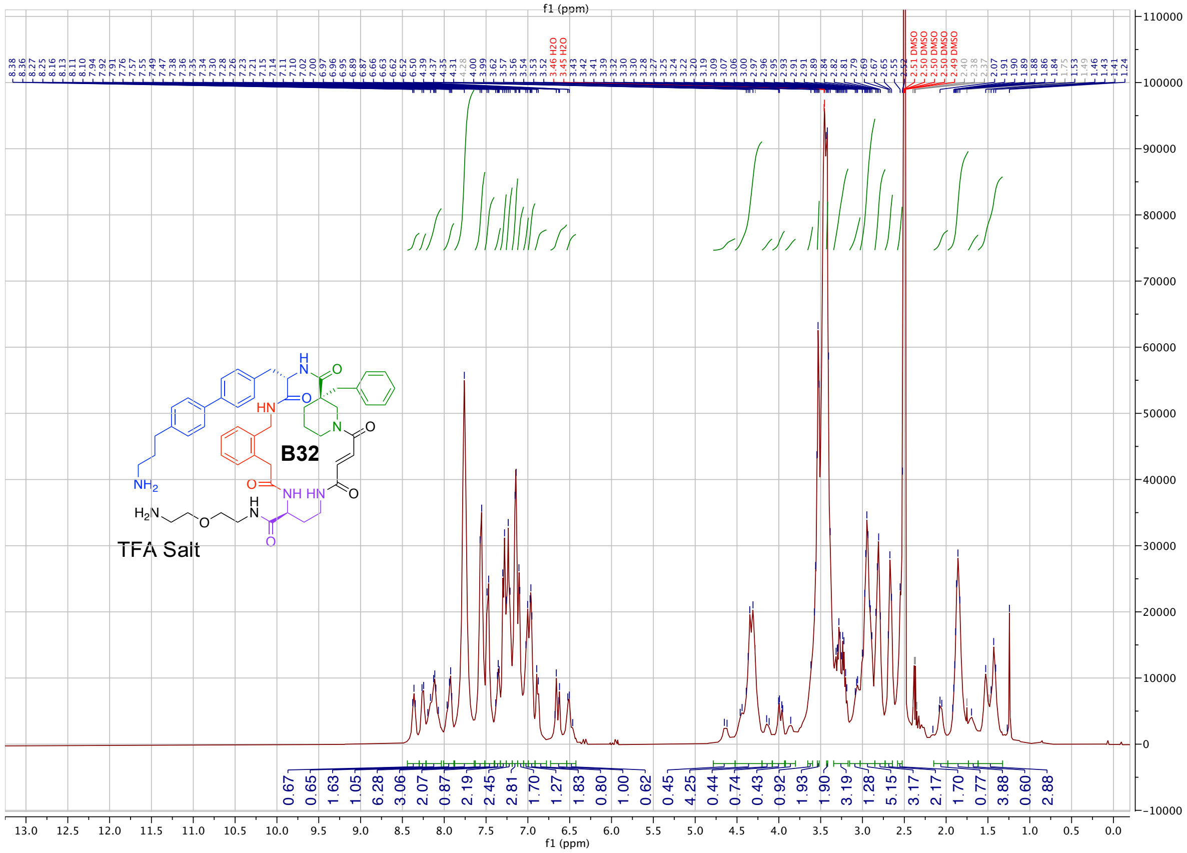
B32
TFA Salt
f1 (ppm)

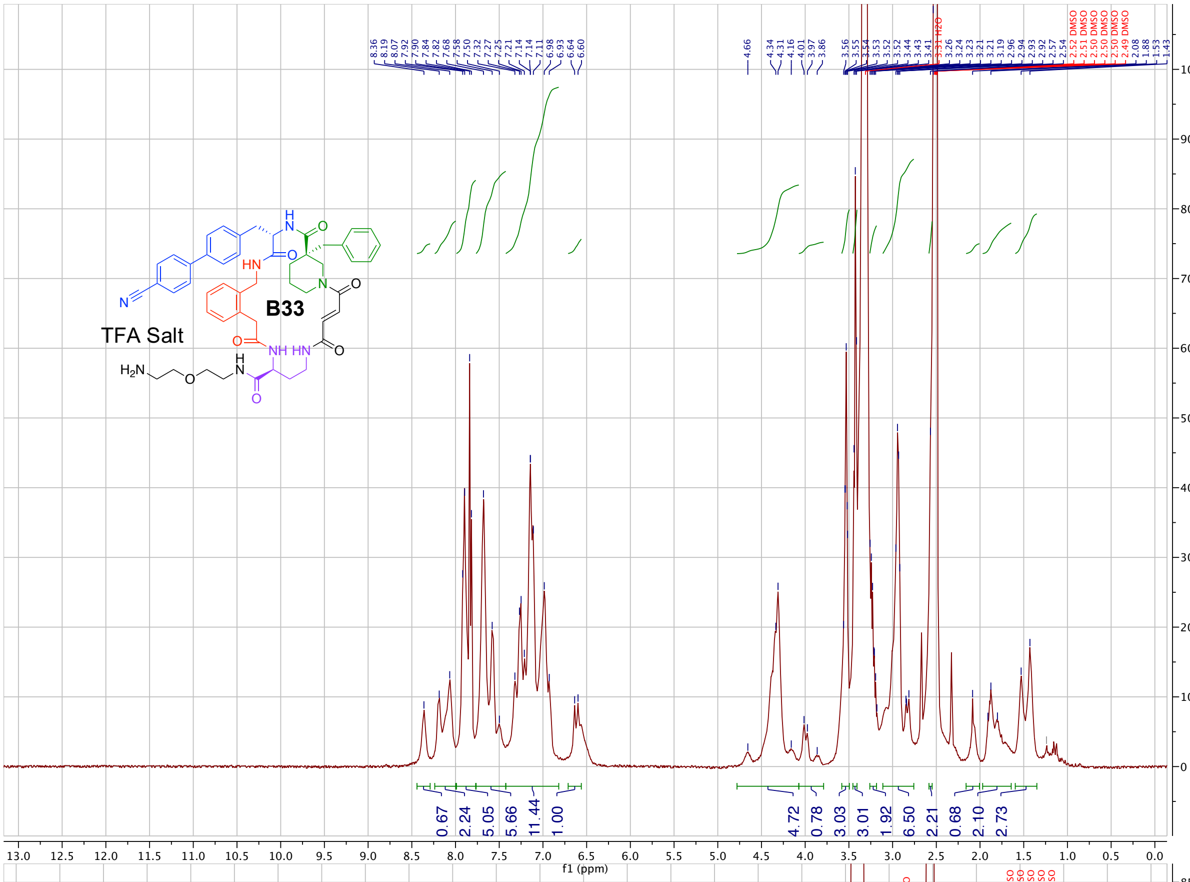
B33
TFA Salt
f1 (ppm)
0.67 2.24 5.05 5.66 11.44 1.00 4.72 0.78 3.03 3.01 1.92 6.50 2.21 0.68 2.10 2.73

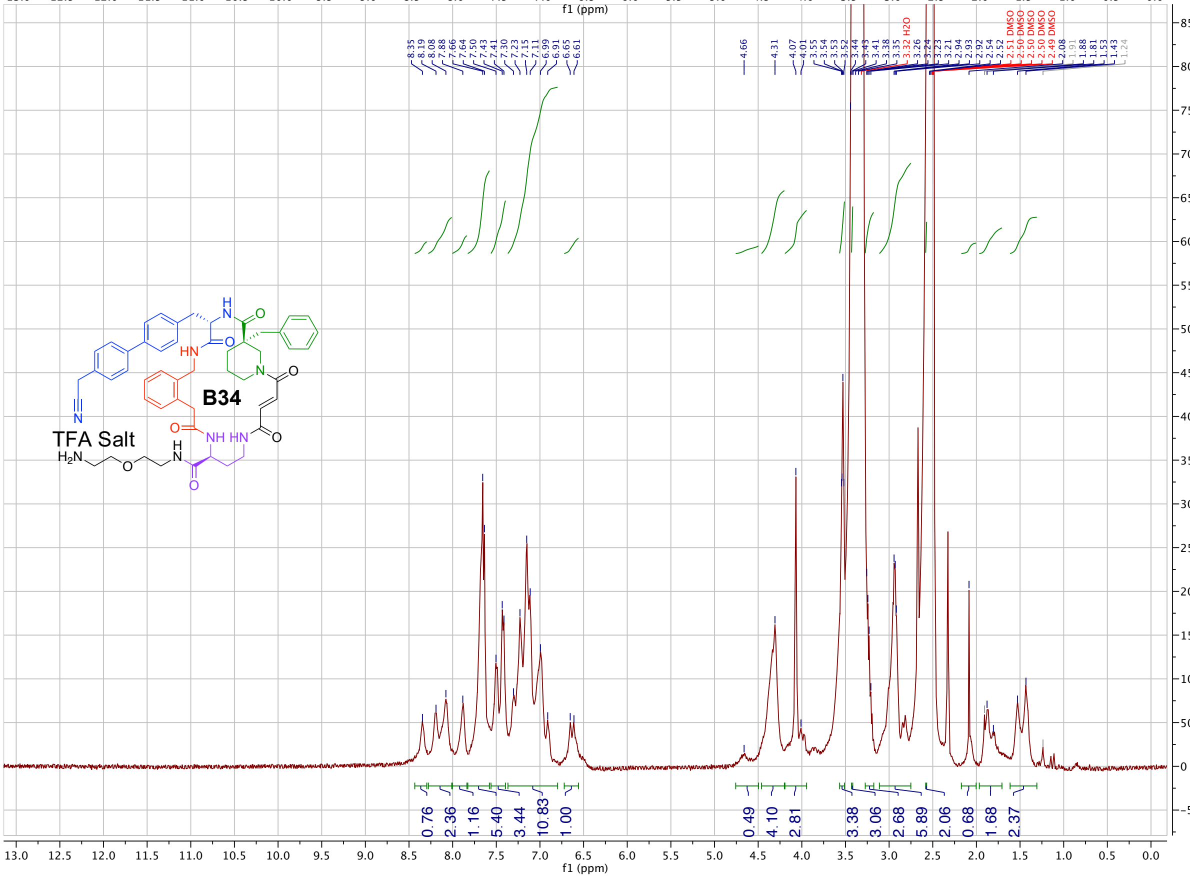
B34
TFA Salt
f1 (ppm)
0.76 2.36 1.16 5.40 3.44 10.83 1.00 0.49 4.10 2.81 3.38 3.06 2.68 5.89 2.06 0.68 1.68 2.37

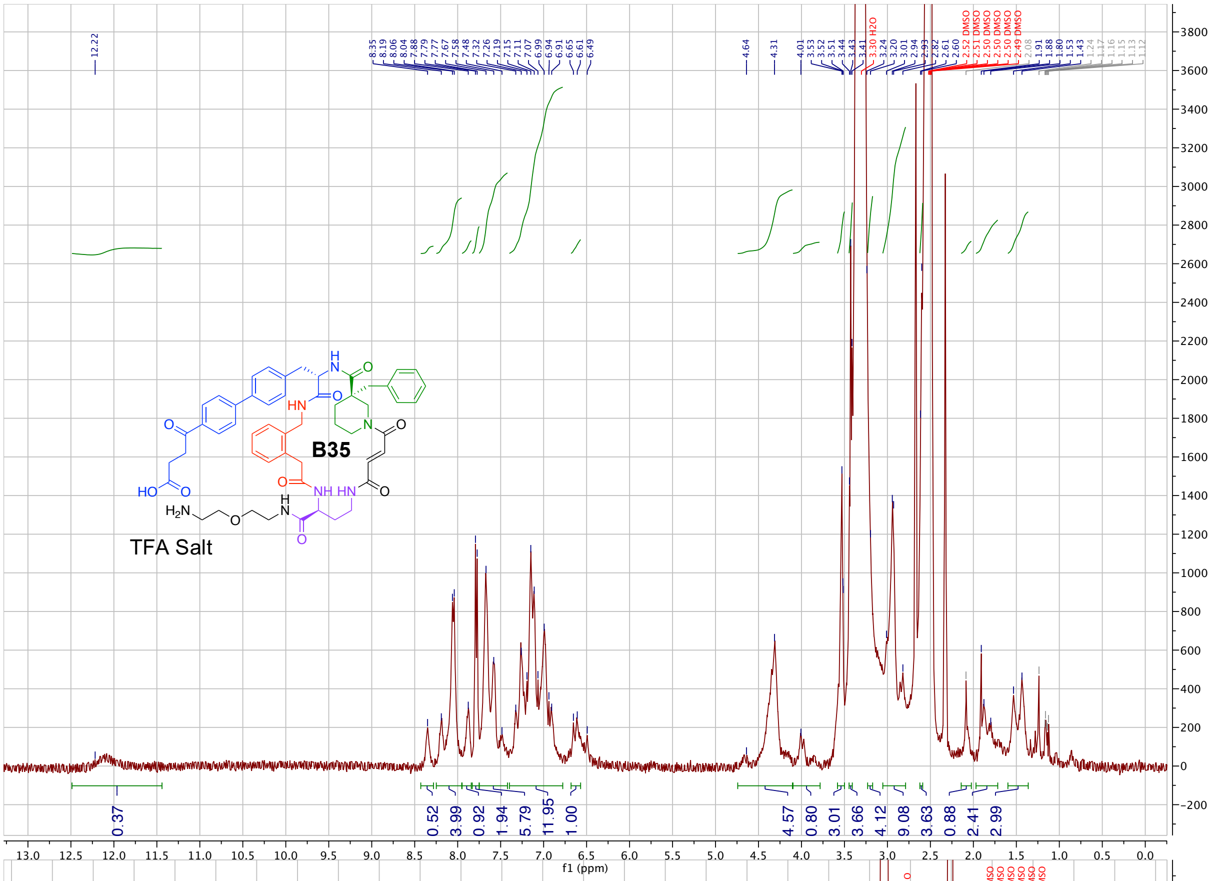
B35
TFA Salt
f1 (ppm)

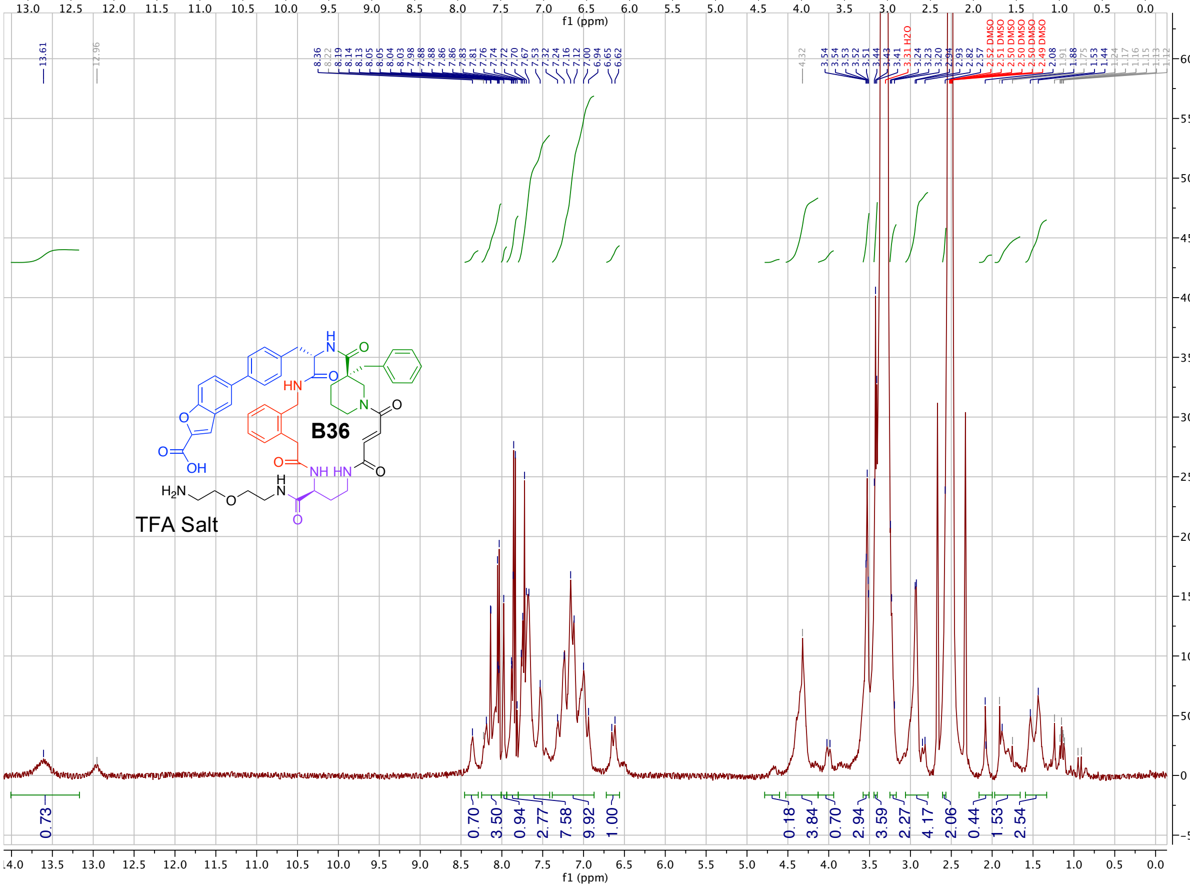
B36
TFA Salt
f1 (ppm)

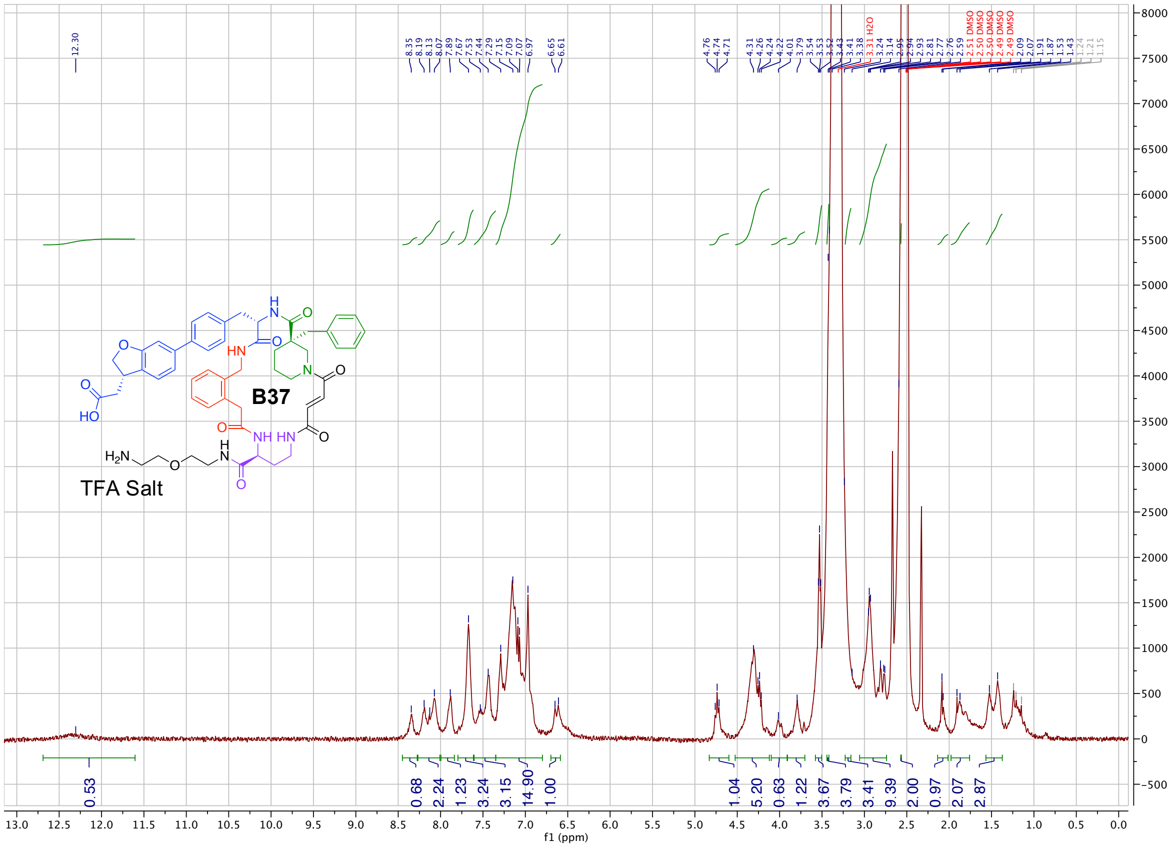
B37
TFA Salt
f1 (ppm)

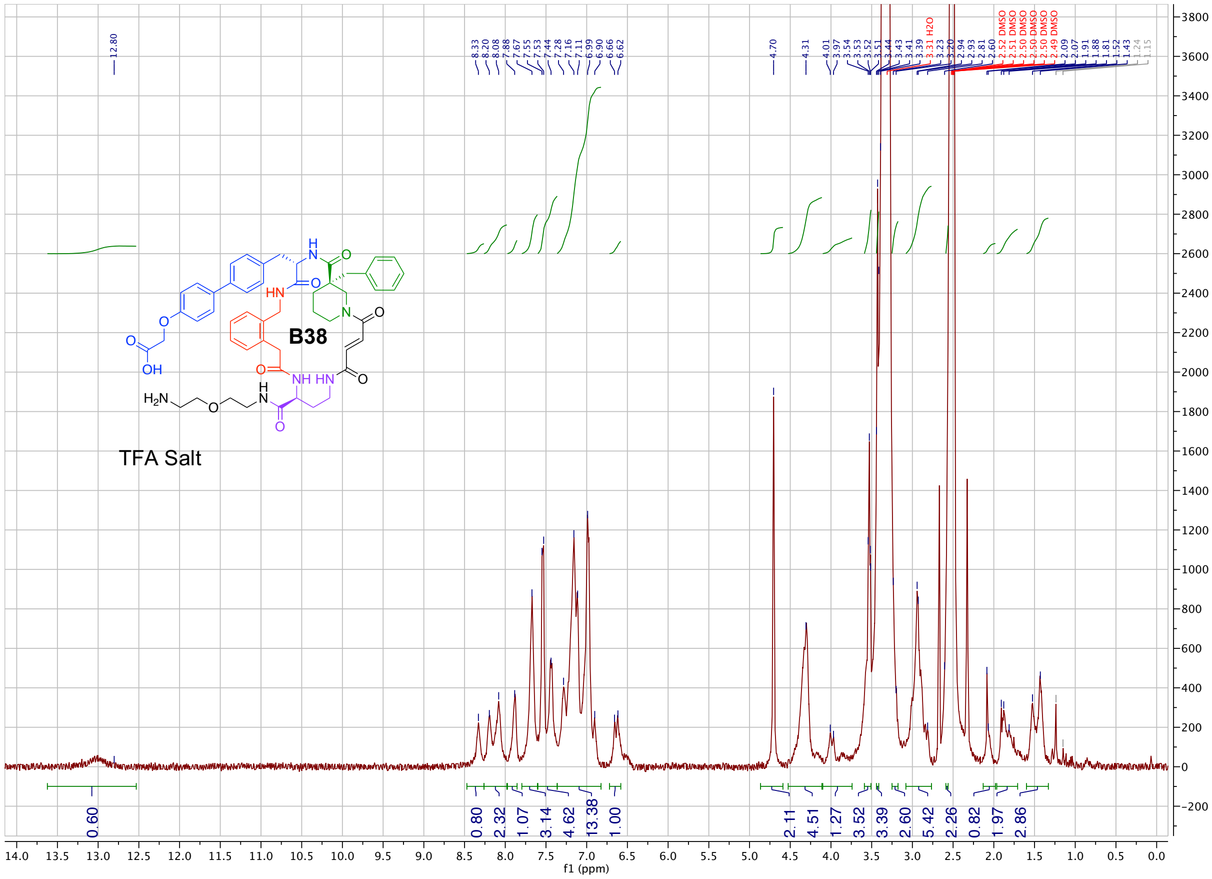
B38
TFA Salt
f1 (ppm)

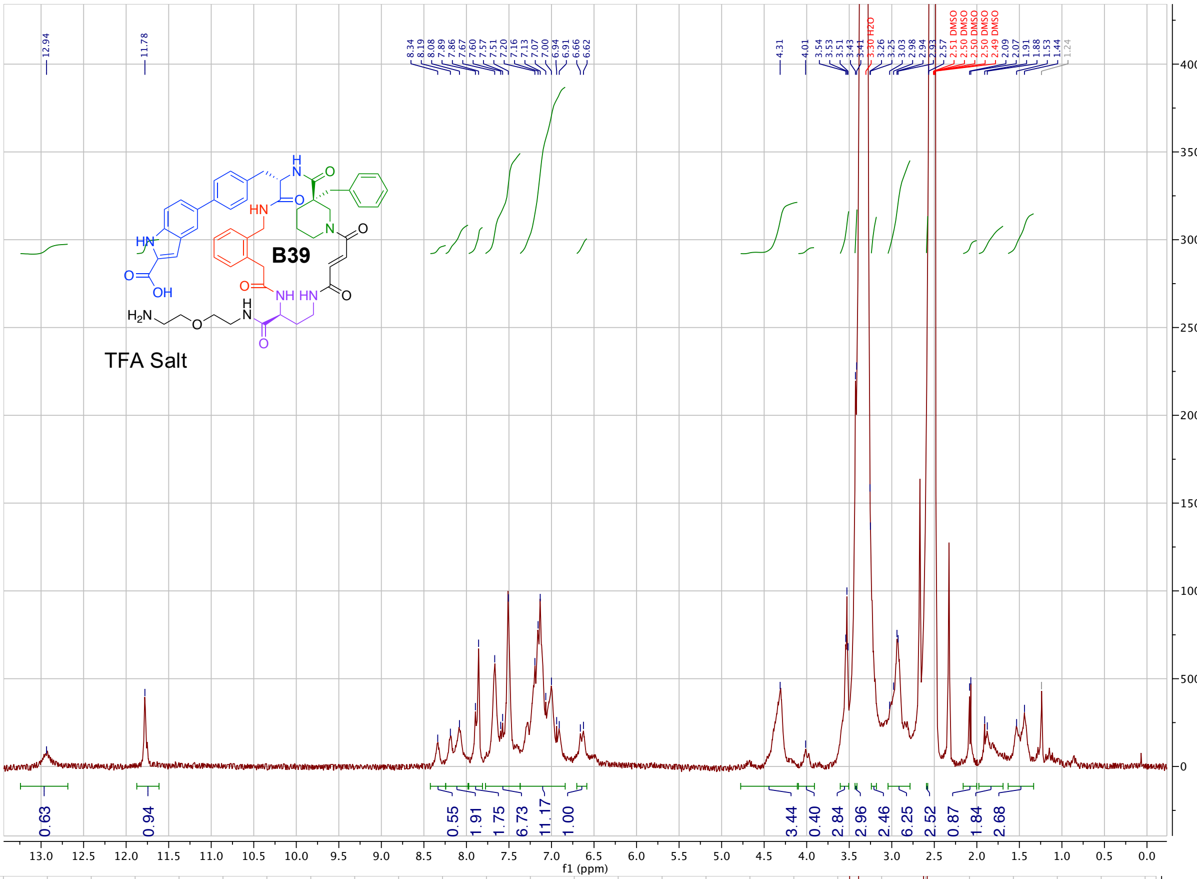
B39
TFA Salt
f1 (ppm)

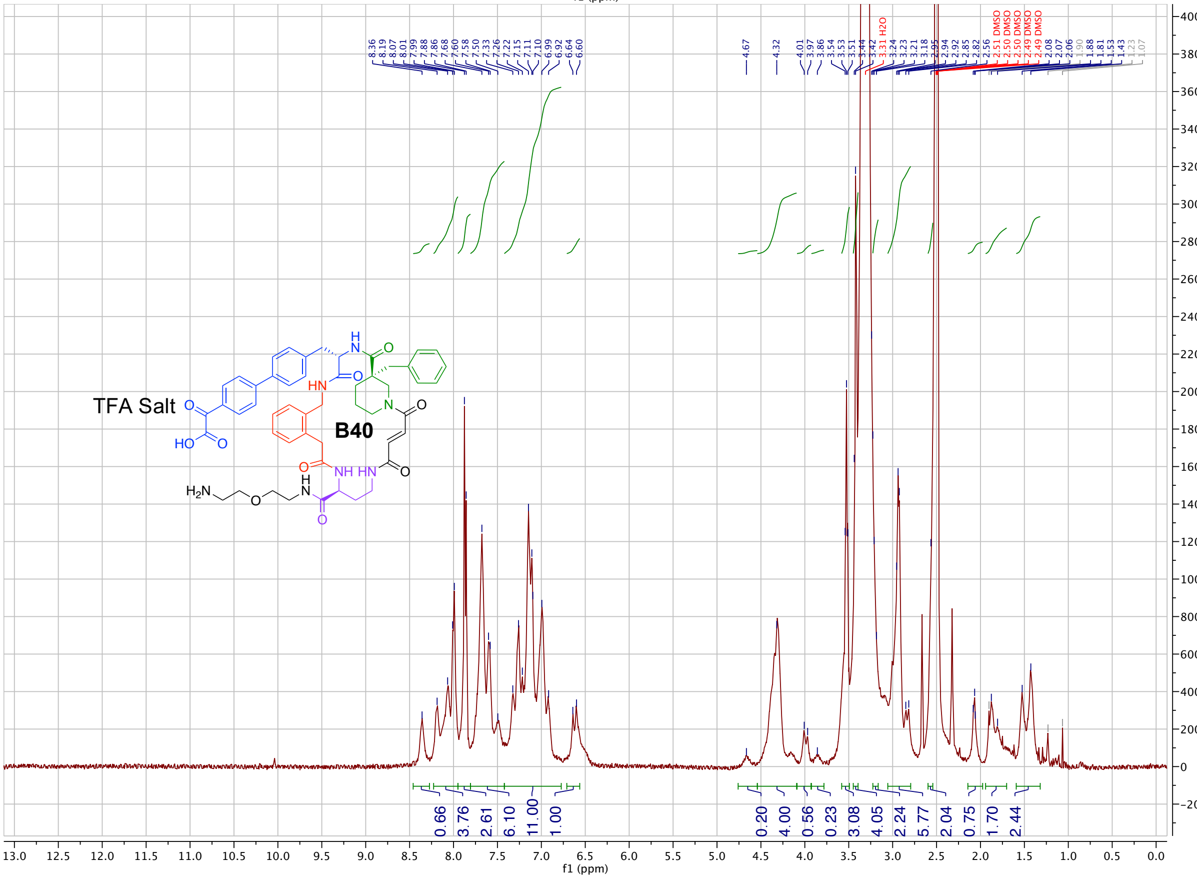
B40
TFA Salt
f1 (ppm)

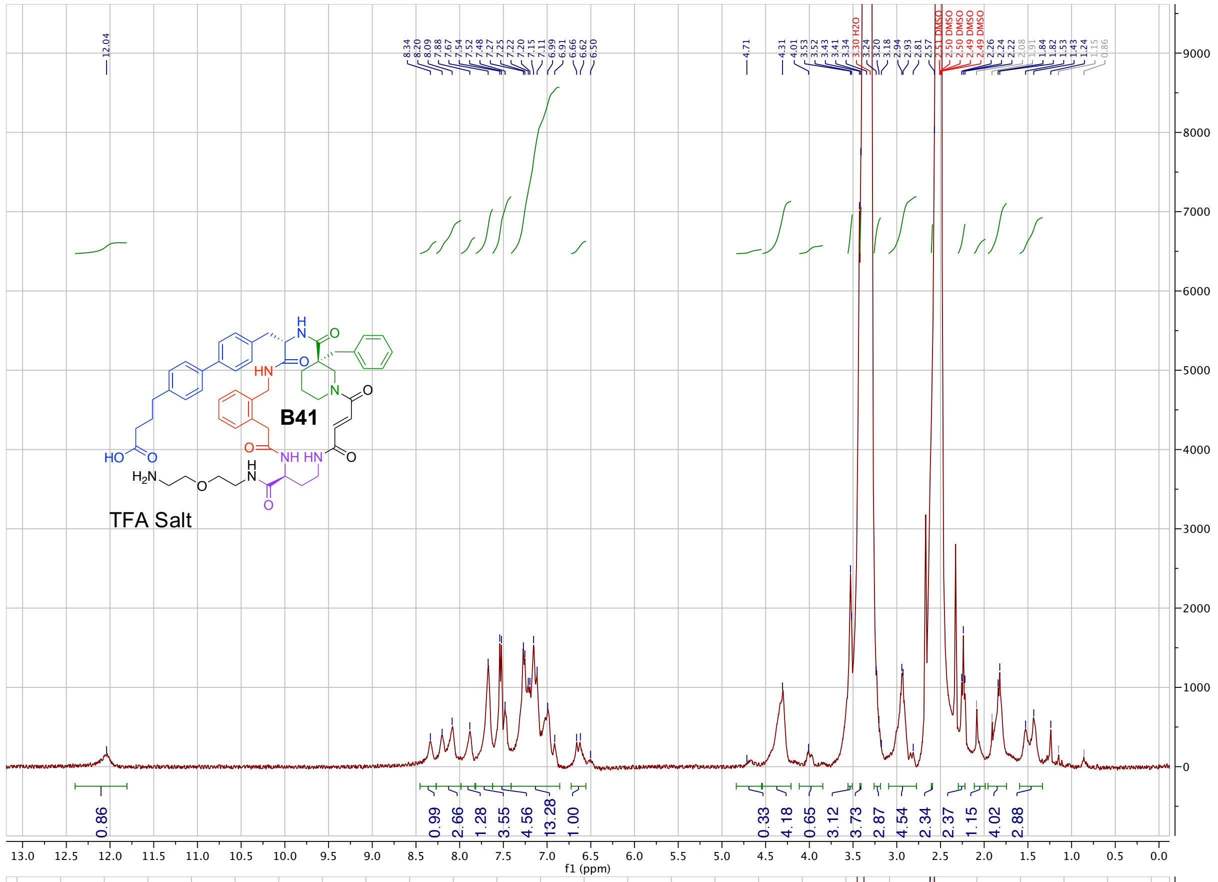
B41
TFA Salt
f1 (ppm)

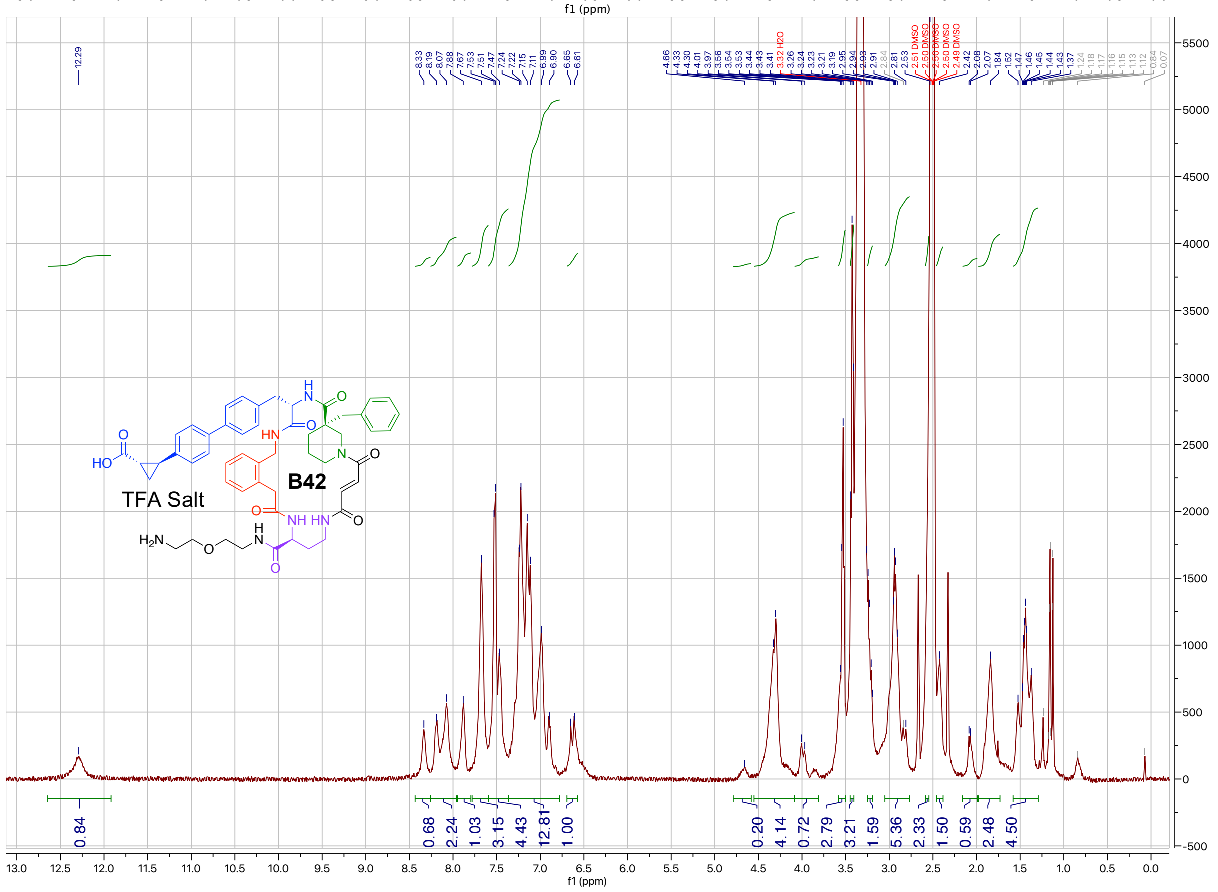
B42
TFA Salt
f1 (ppm)

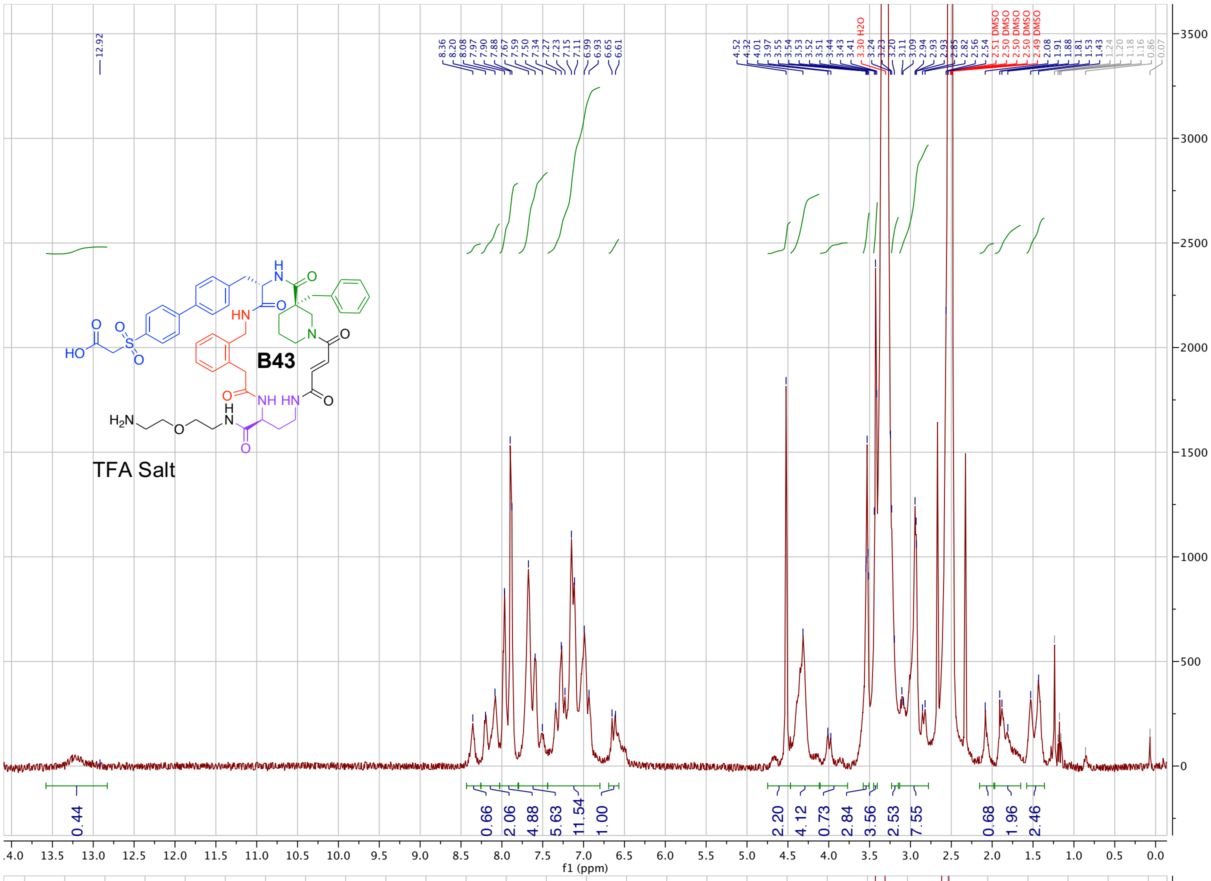
B43
TFA Salt
f1 (ppm)

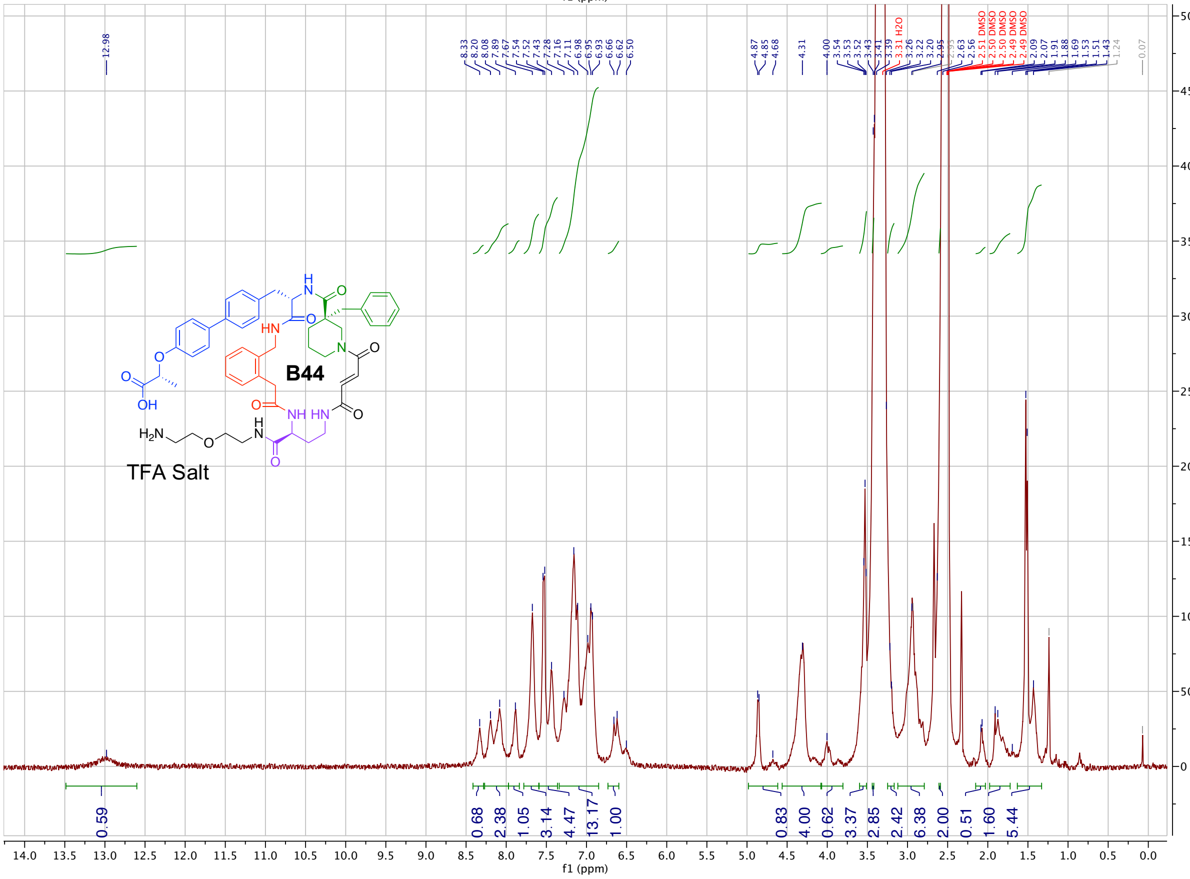
B44
TFA Salt
f1 (ppm)

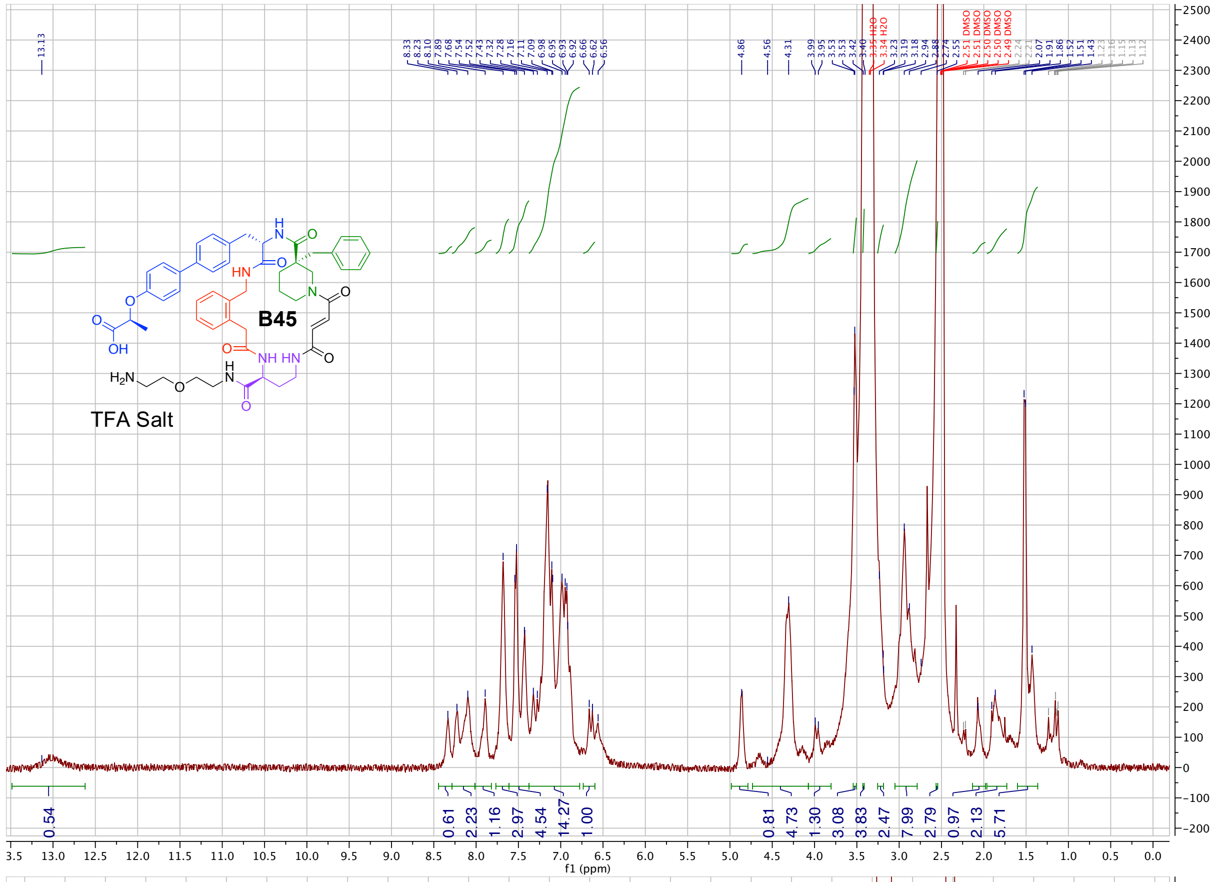
B45
TFA Salt
f1 (ppm)

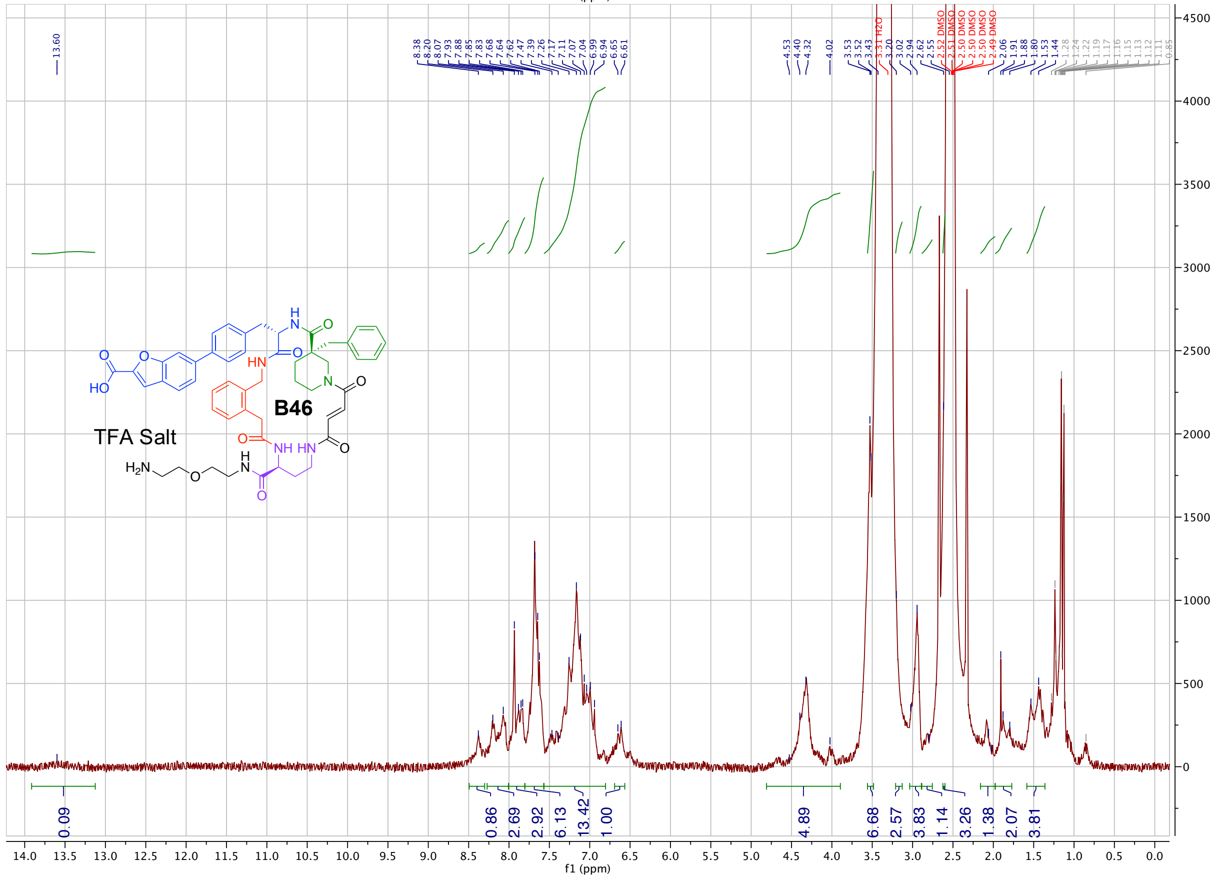
B46
TFA Salt
f1 (ppm)

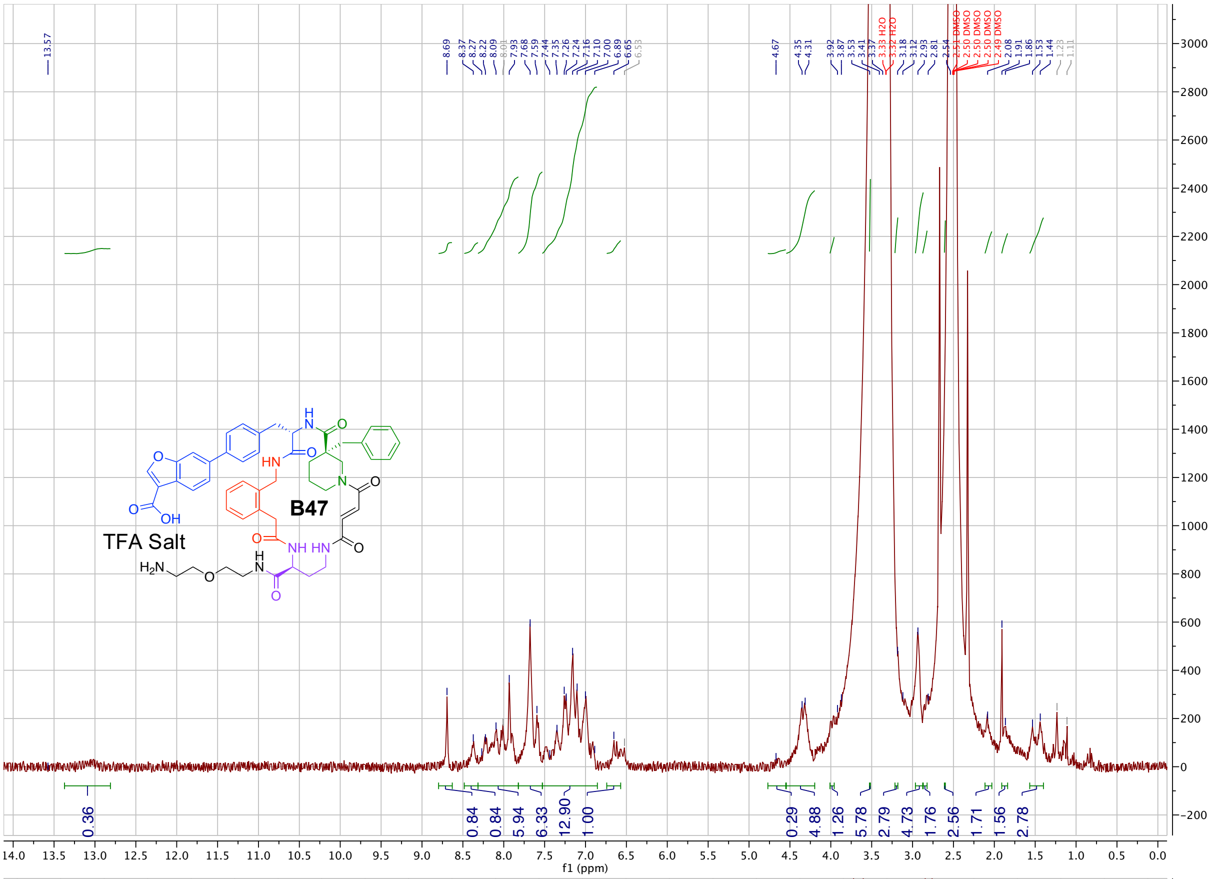
B47
TFA Salt
f1 (ppm)

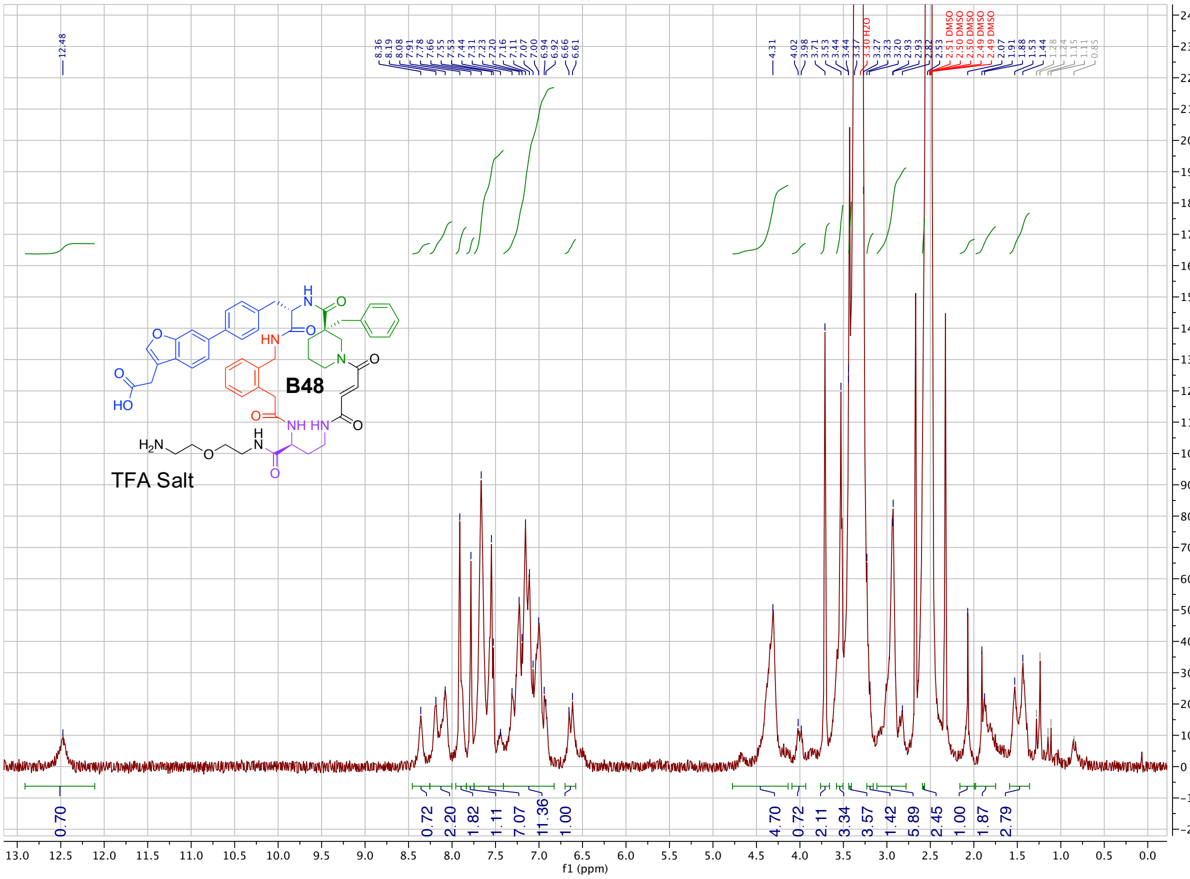
B48
TFA Salt
f1 (ppm)

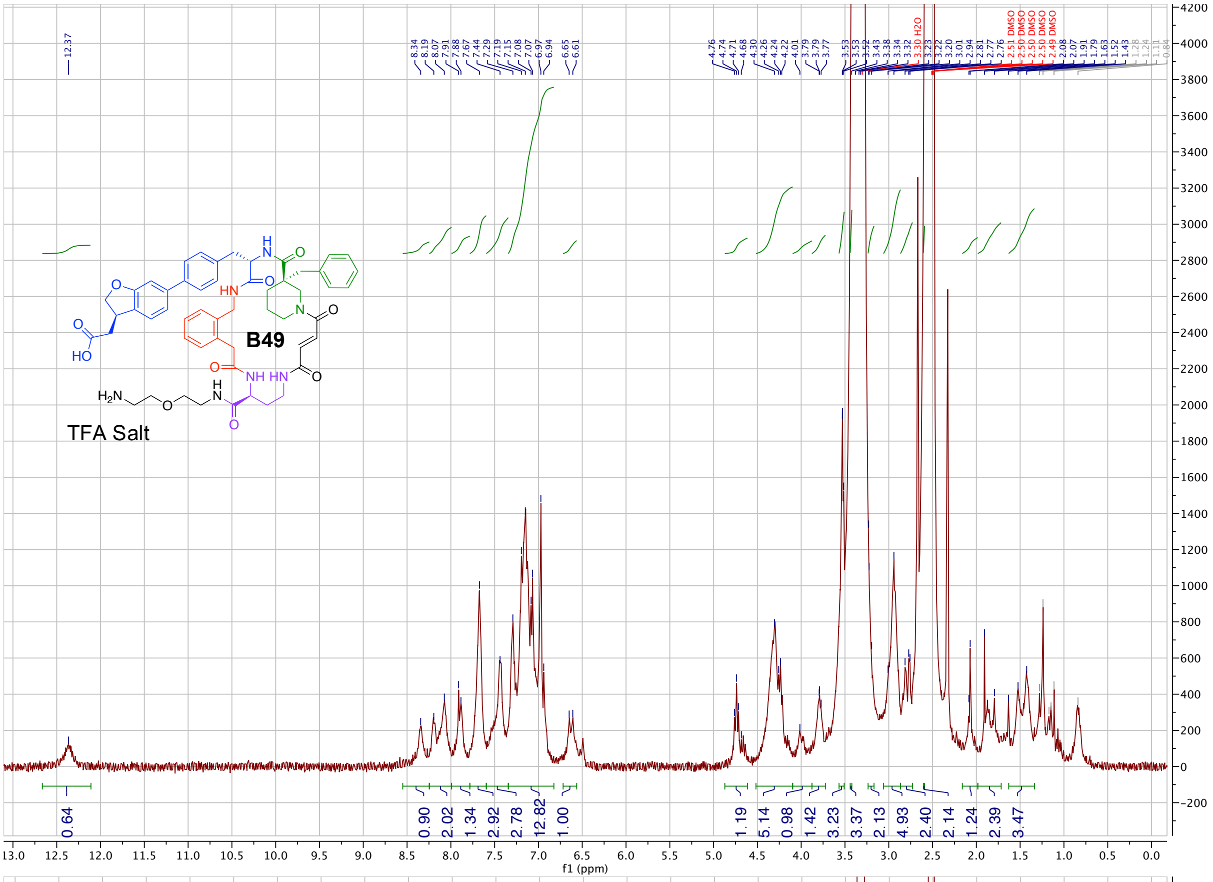
B49
TFA Salt
f1 (ppm)

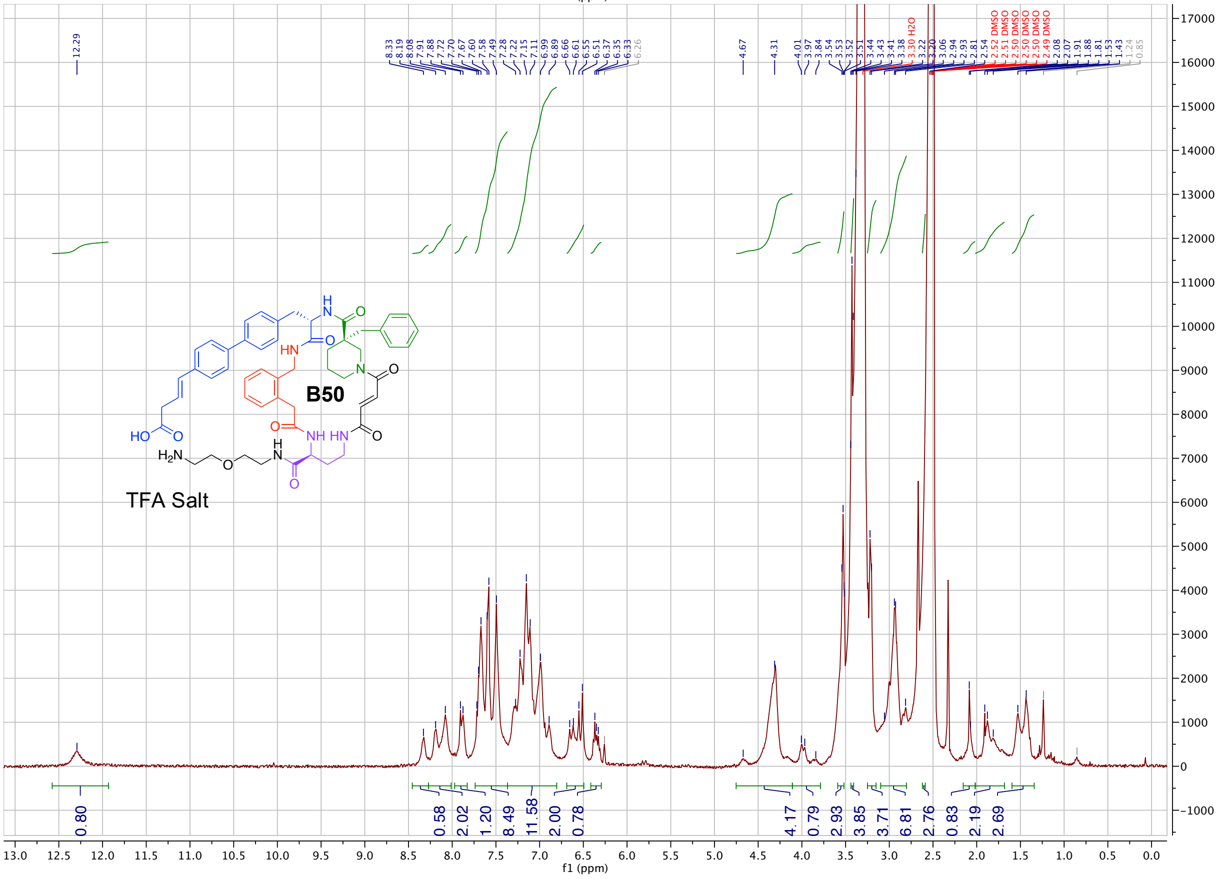
B50
TFA Salt
f1 (ppm)

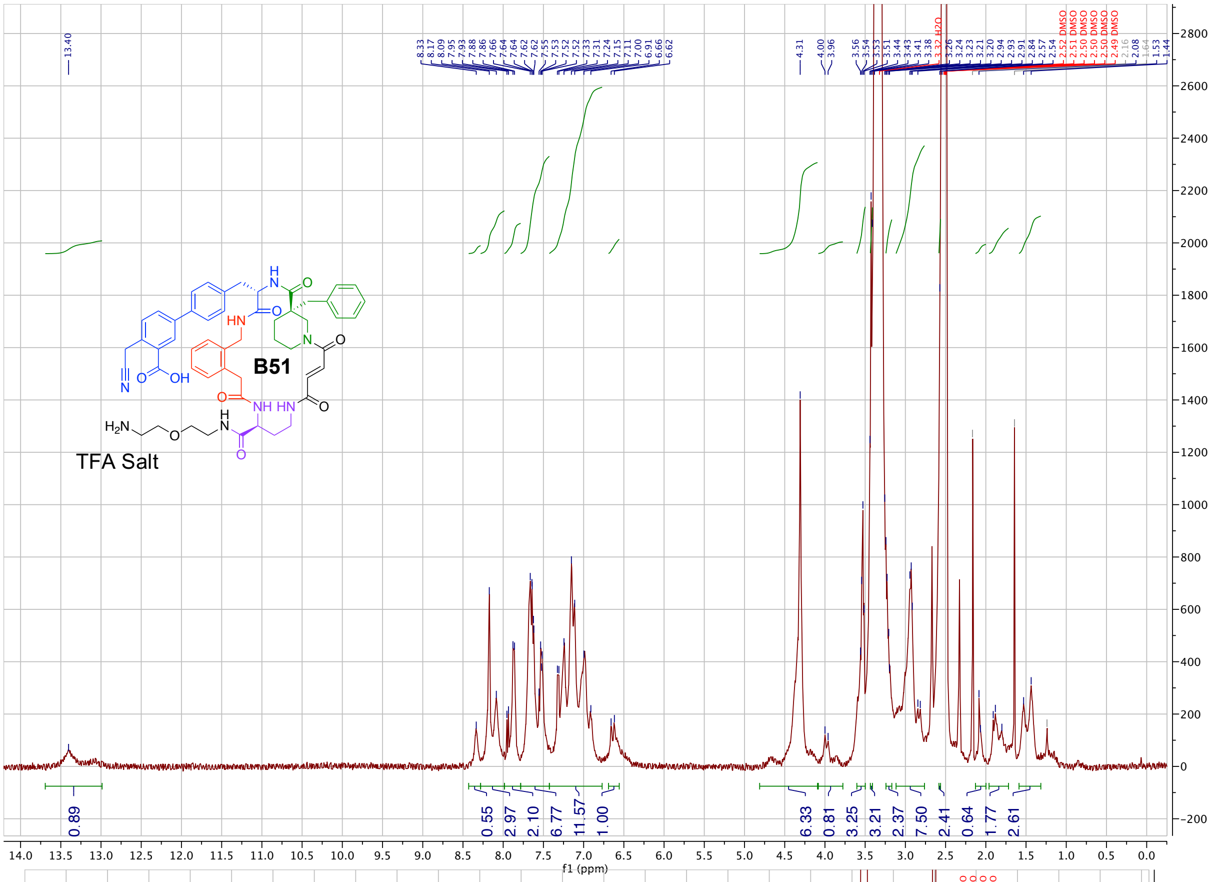
B51
TFA Salt
f1 (ppm)

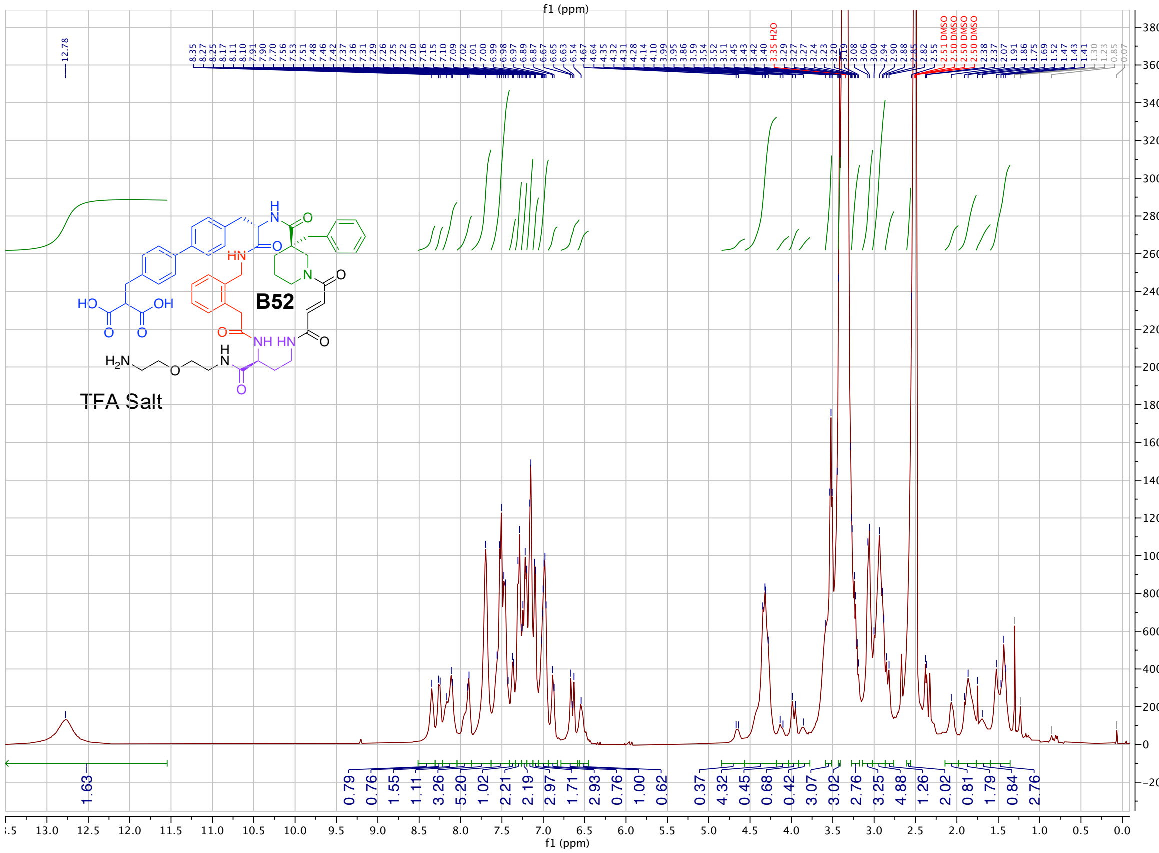
B52
TFA Salt
f1 (ppm)

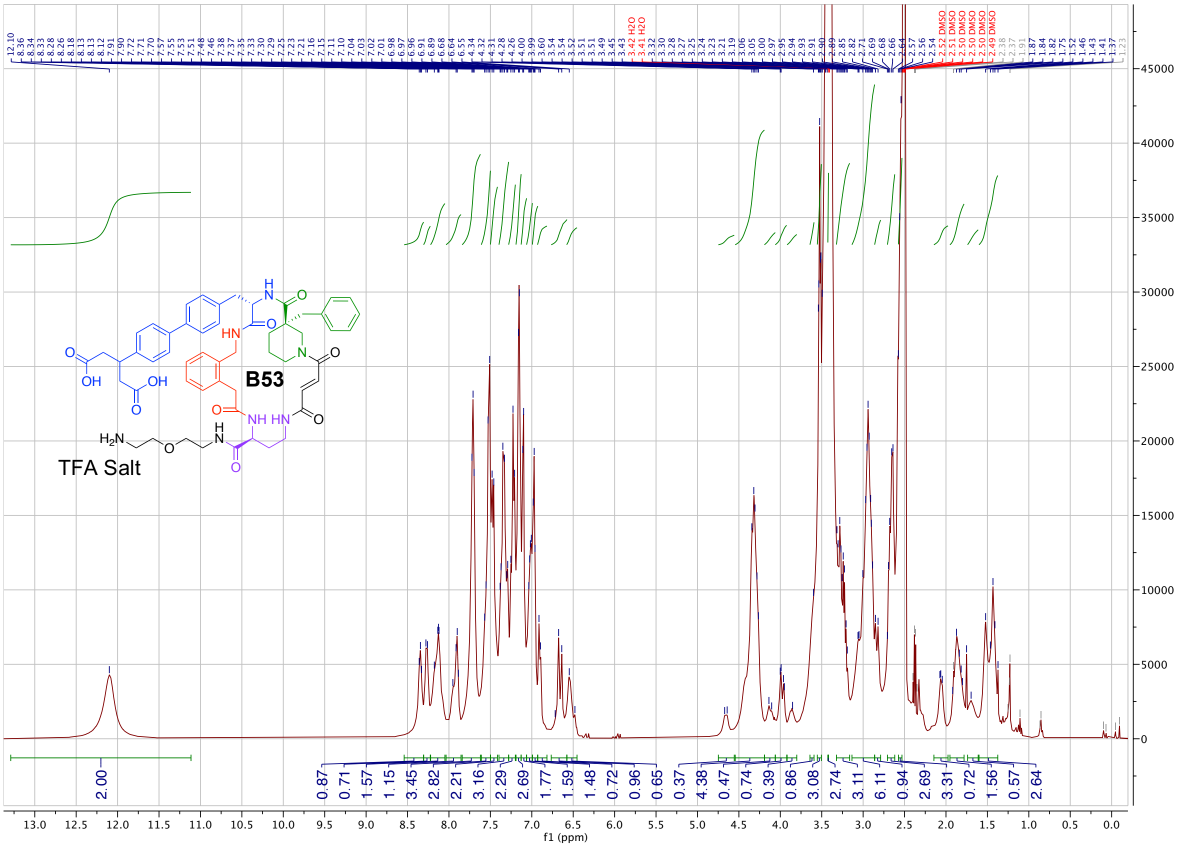

B53
TFA Salt
f1 (ppm)

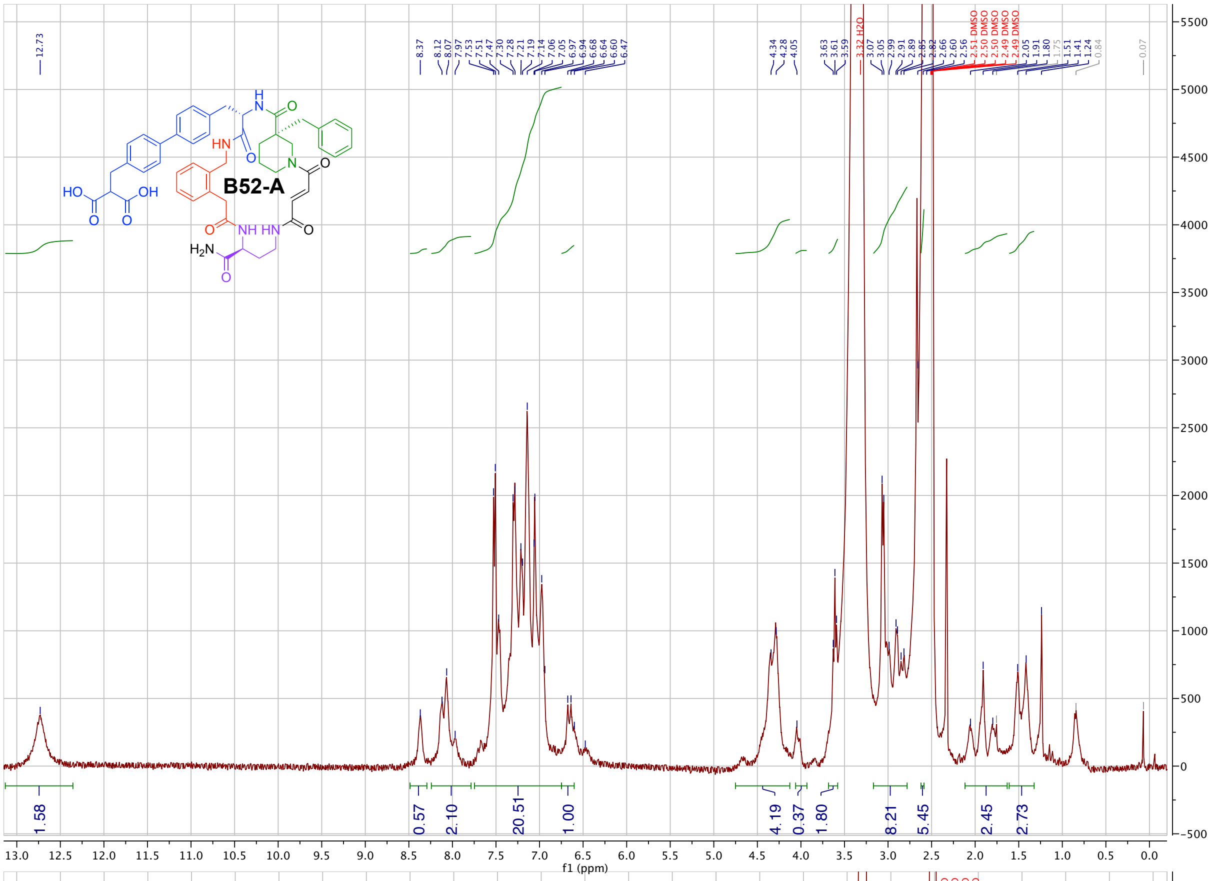
B52-A
f1 (ppm)

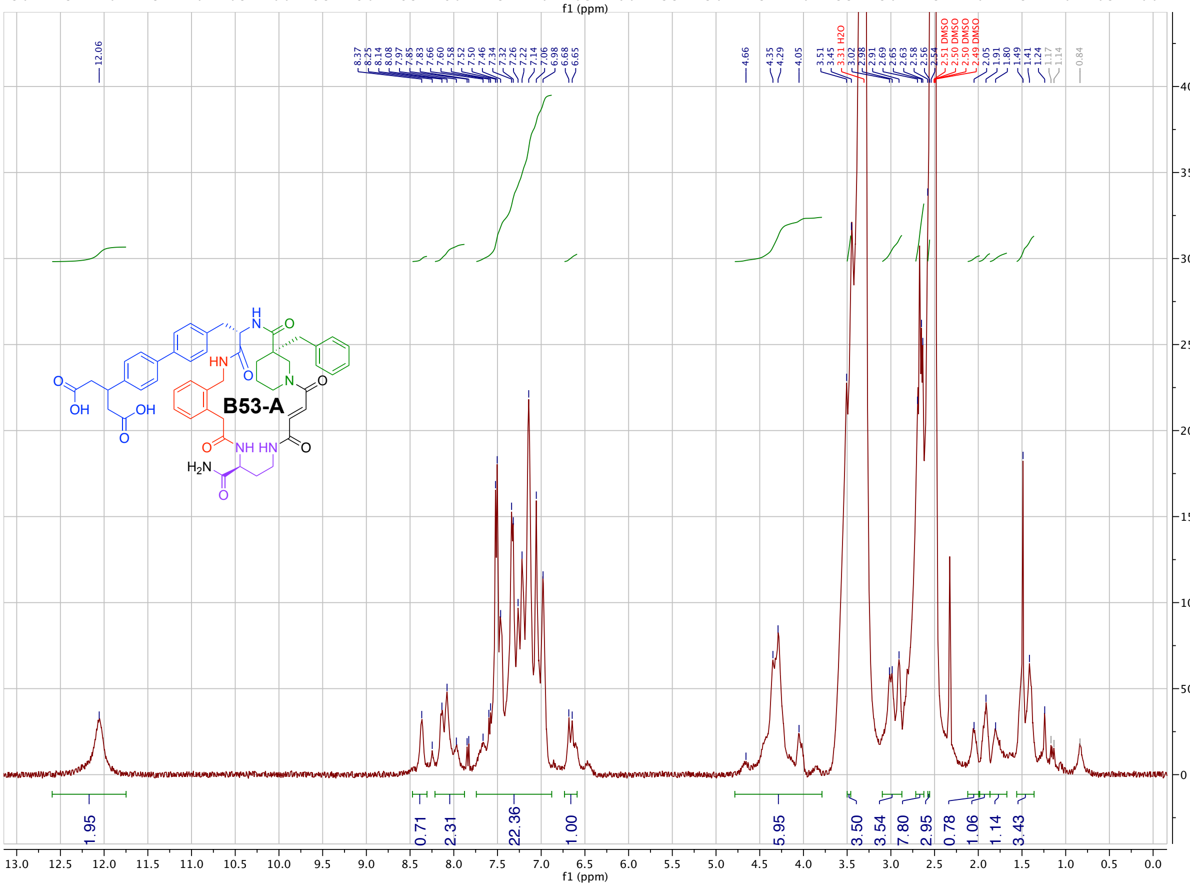
B53-A
f1 (ppm)

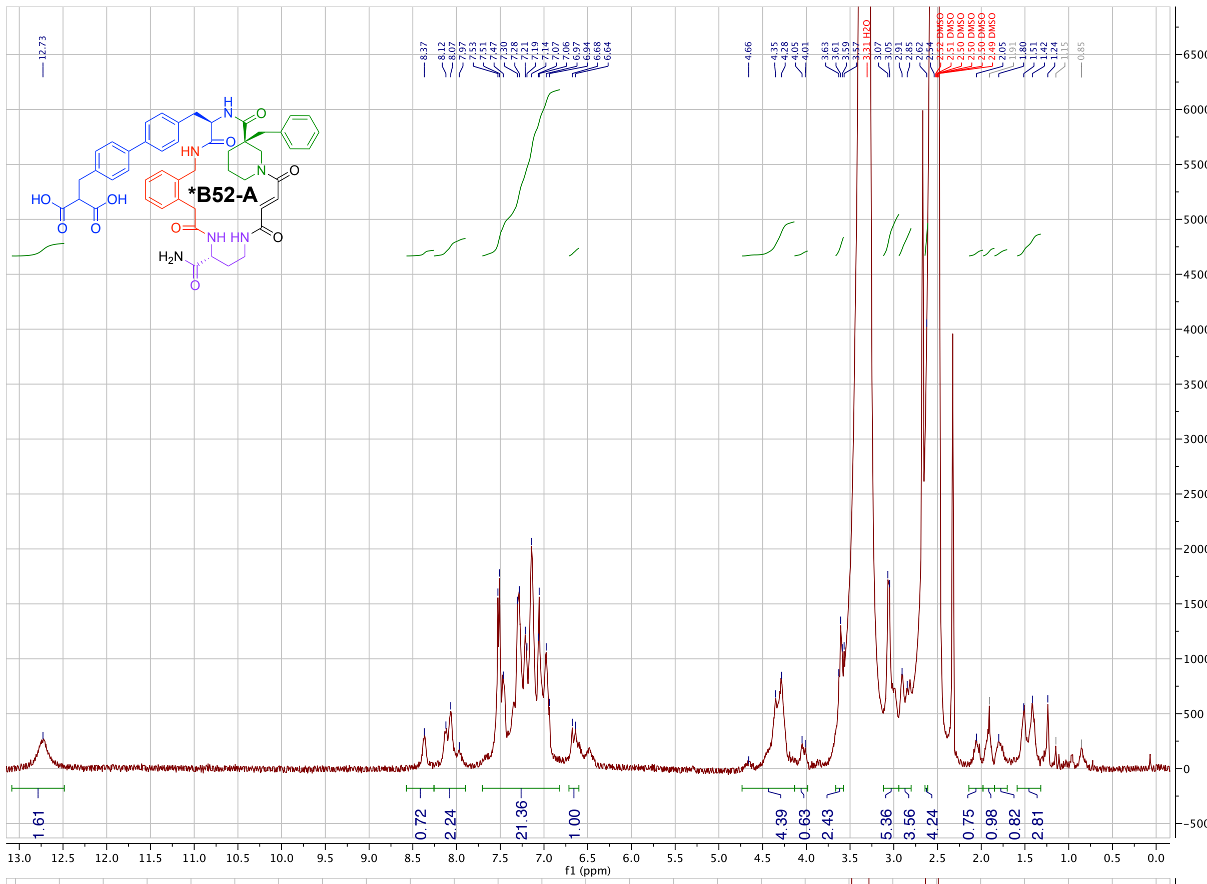

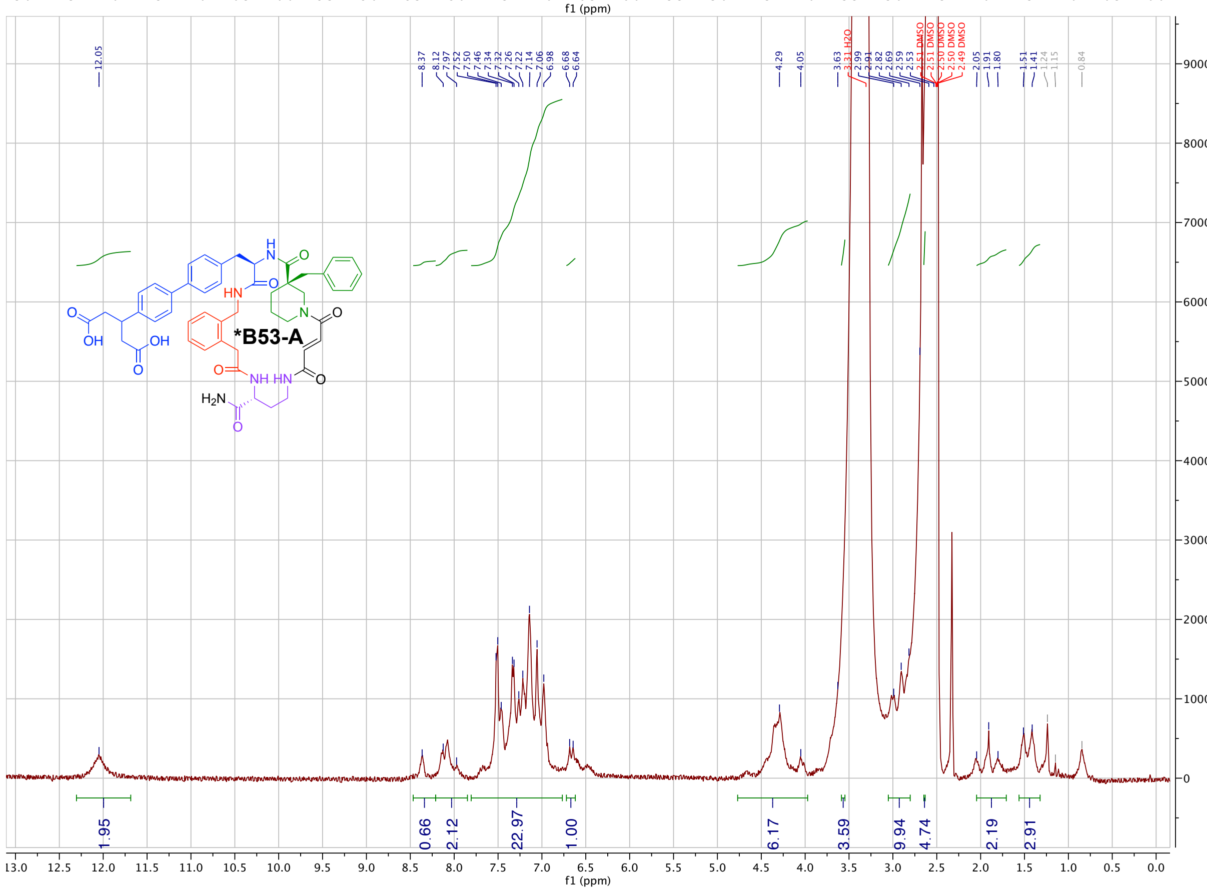

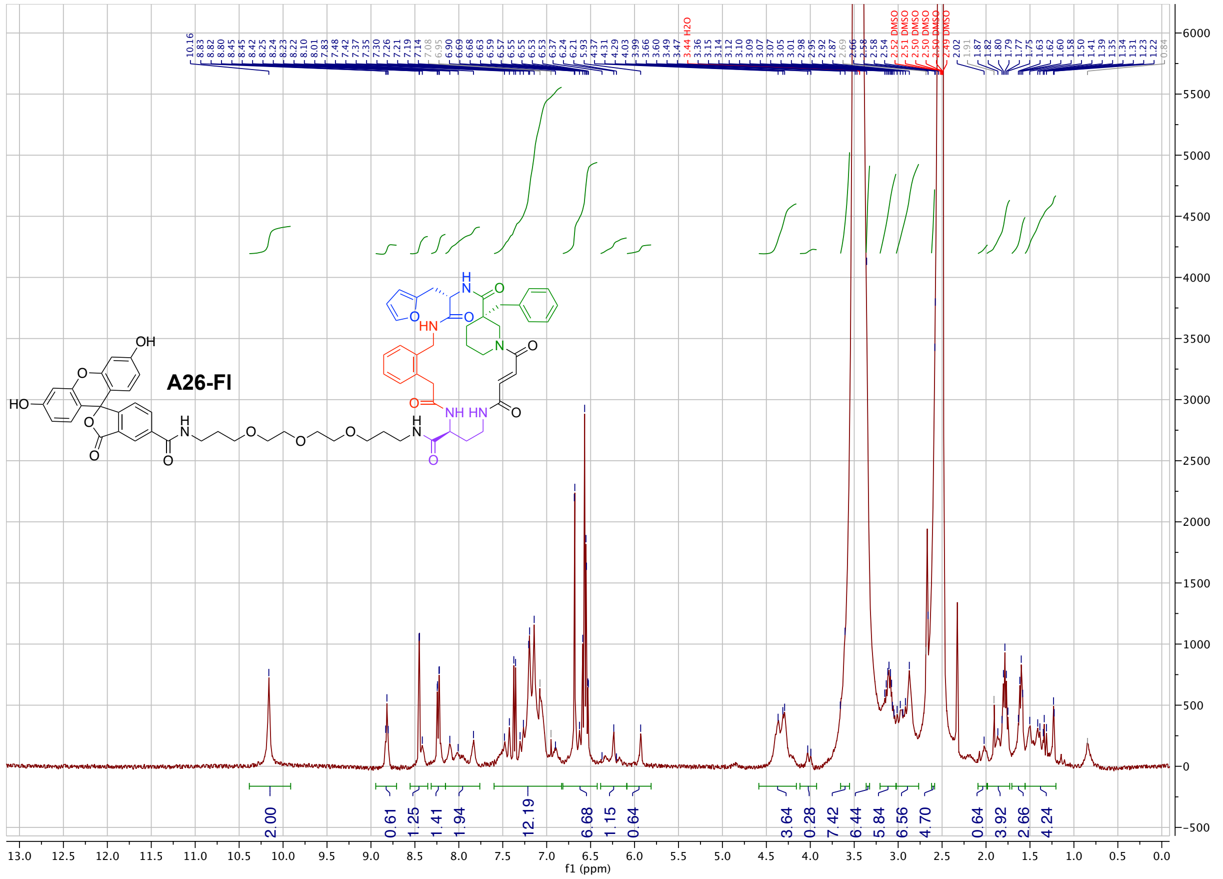
A26-Fl
f1 (ppm)

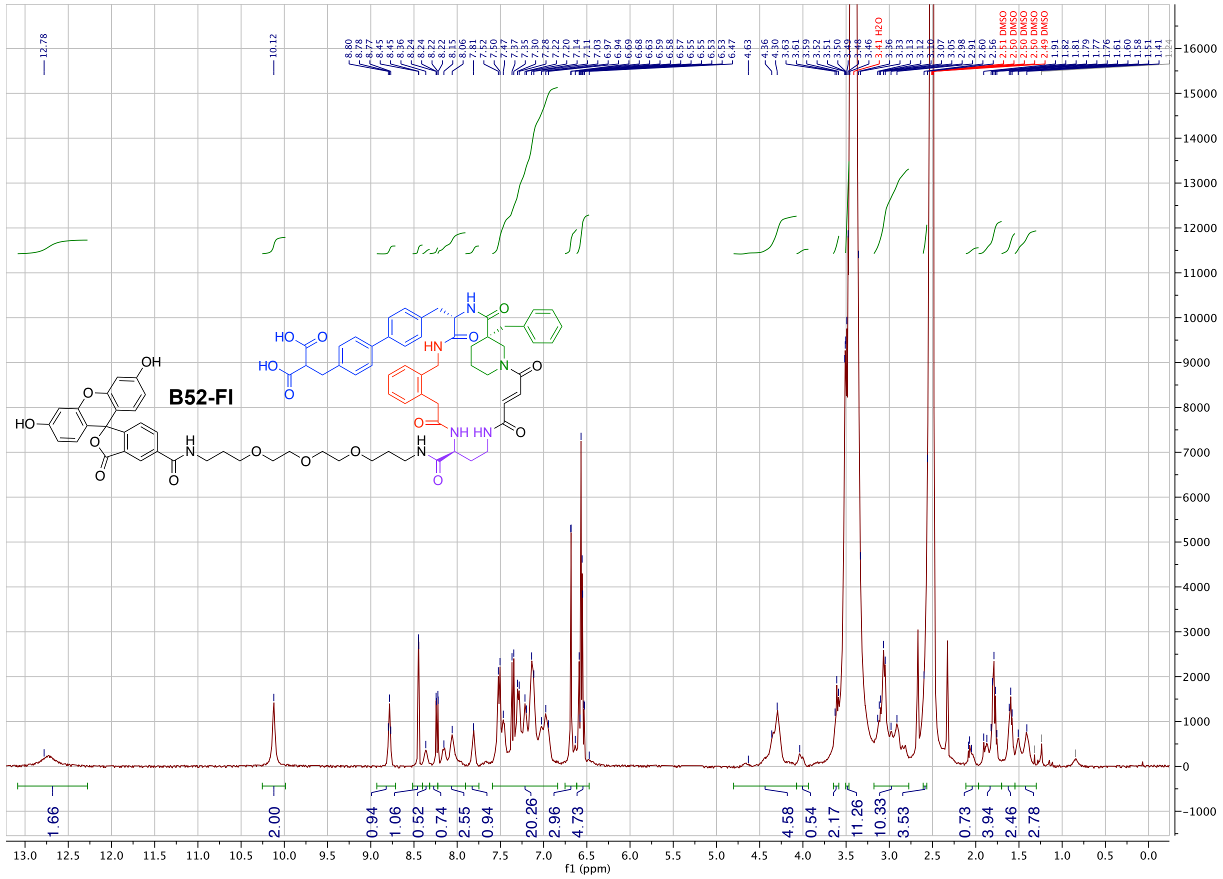
B52-Fl
f1 (ppm)

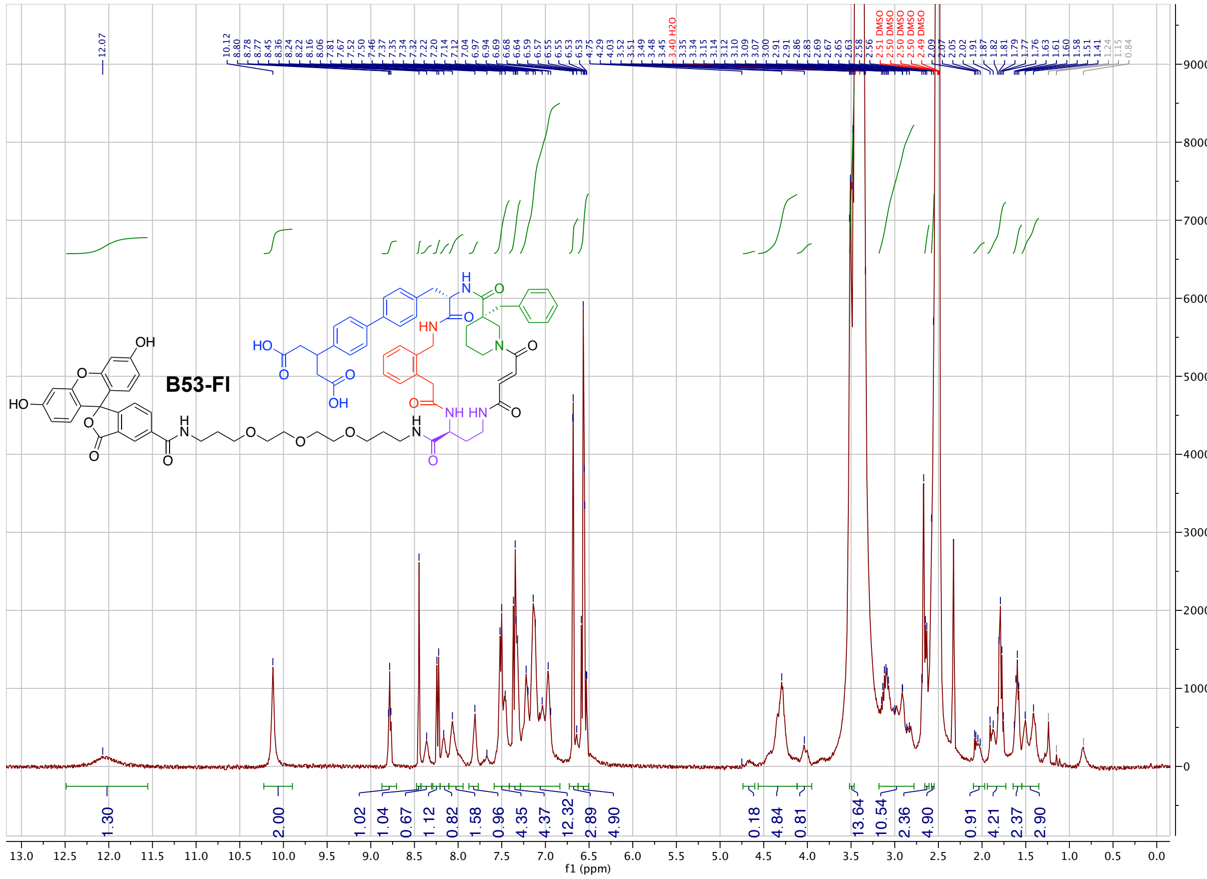
B53-Fl
f1 (ppm)

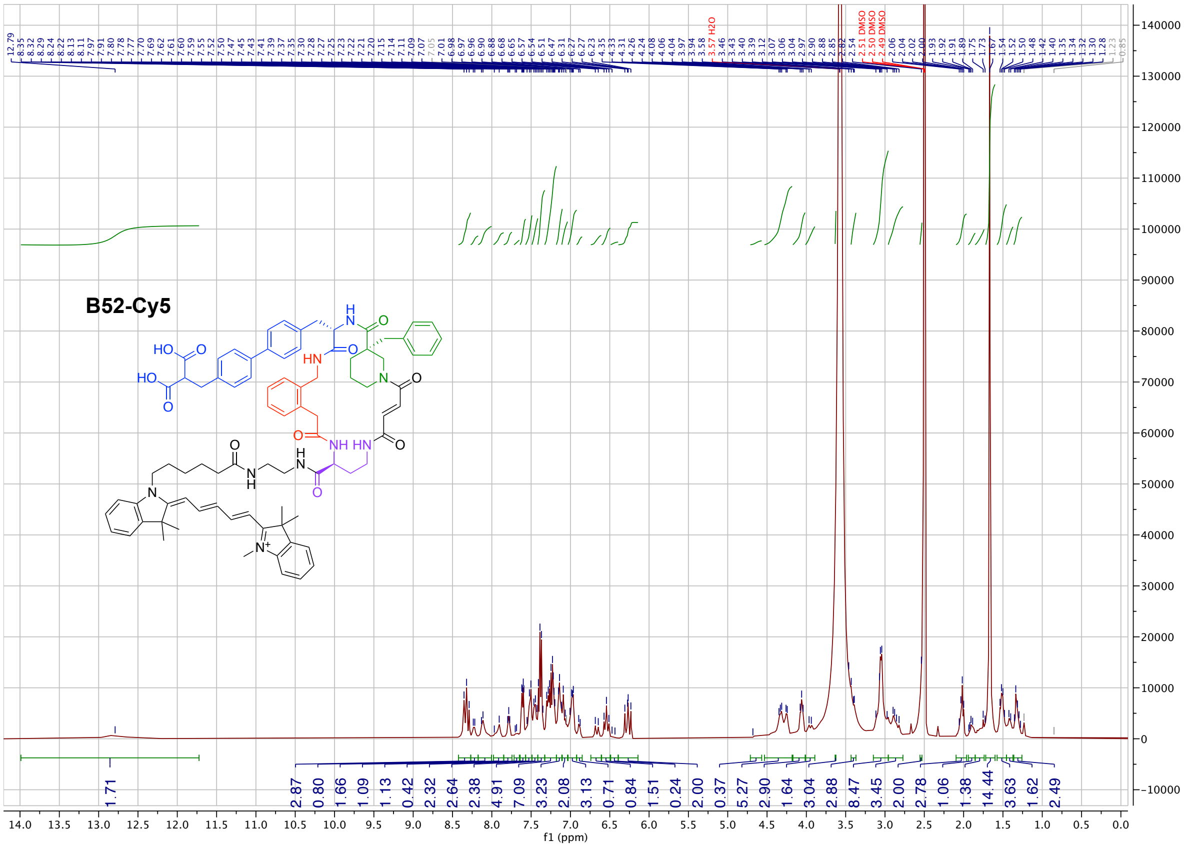
B52-Cy5
f1 (ppm)

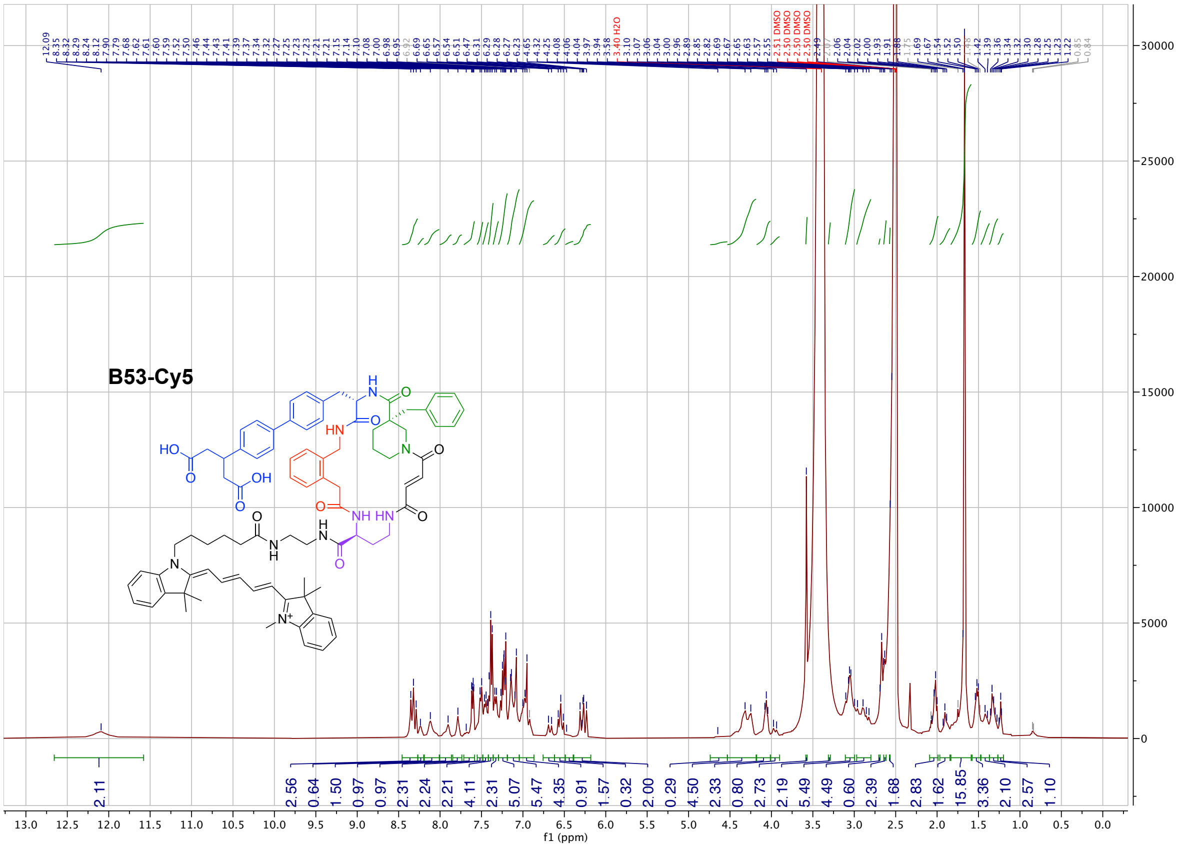
B53-Cy5
f1 (ppm)

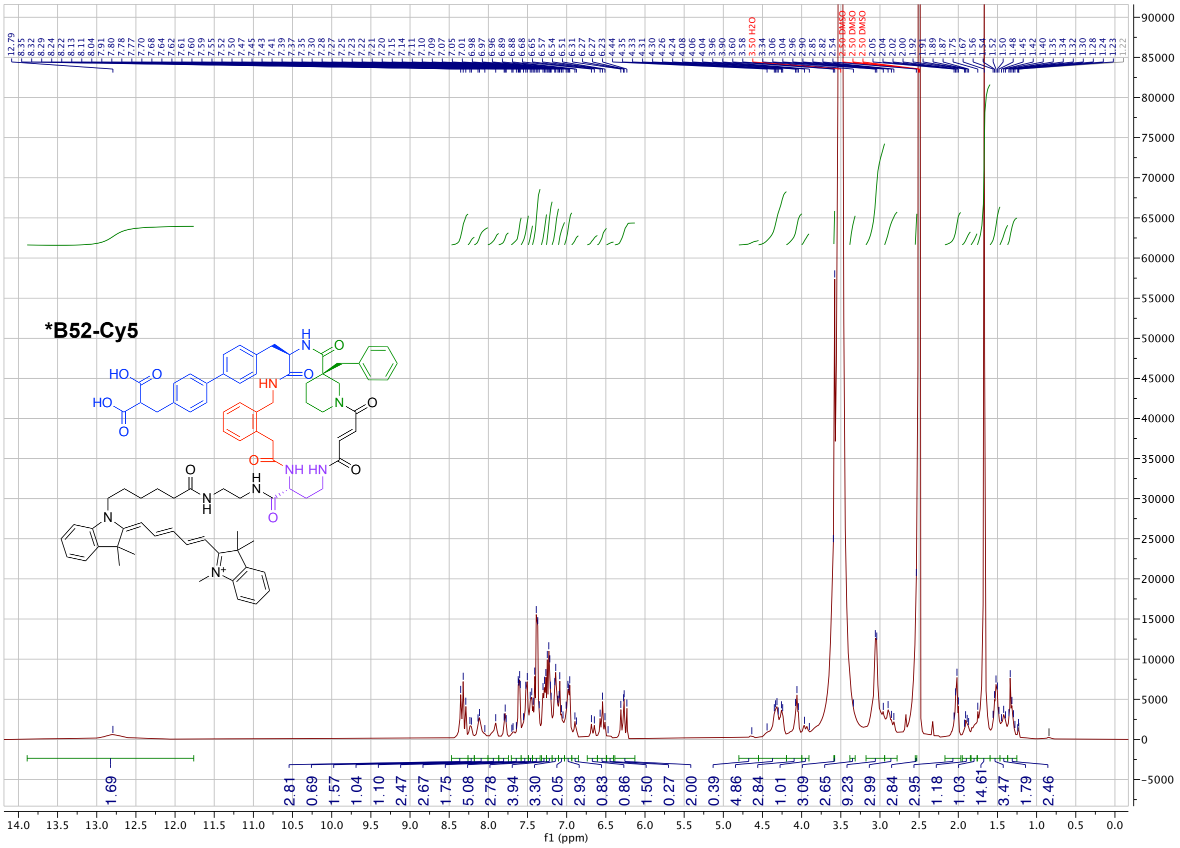
*B52-Cy5
f1 (ppm)

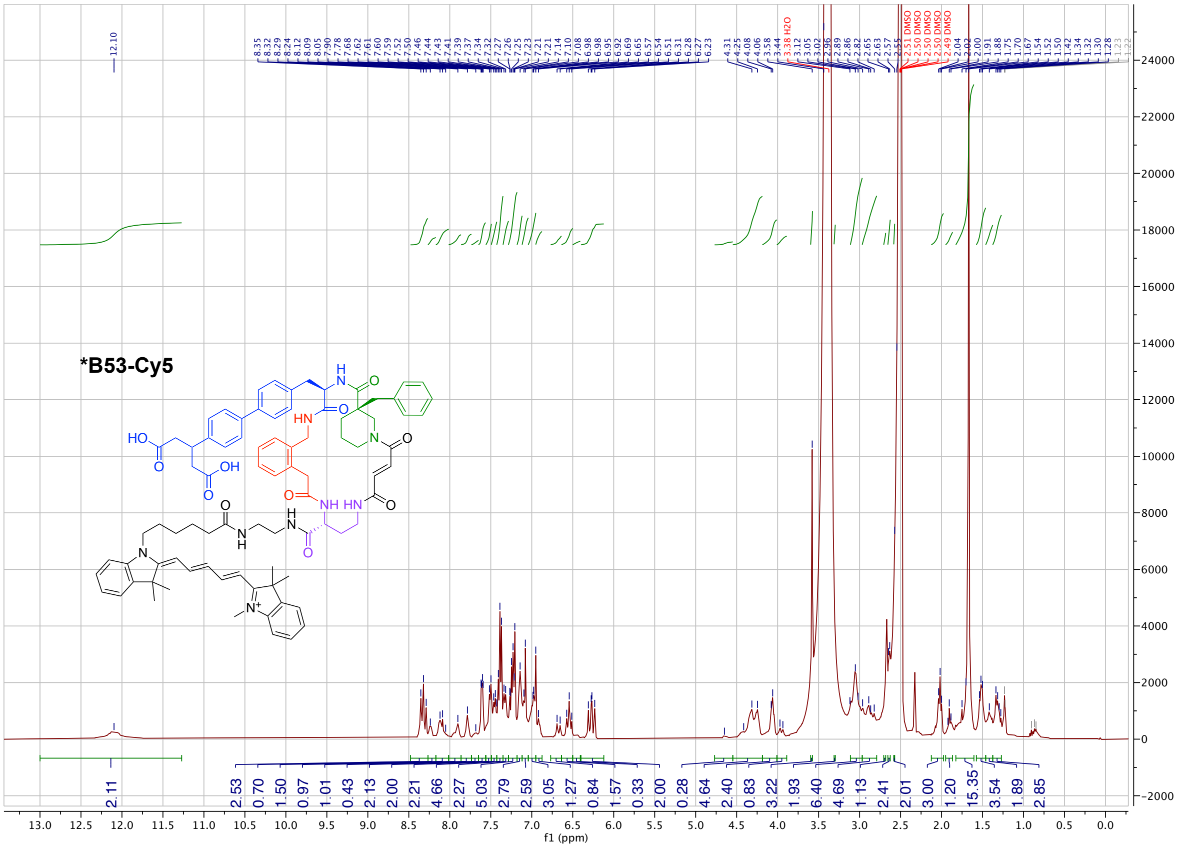
*B53-Cy5
f1 (ppm)

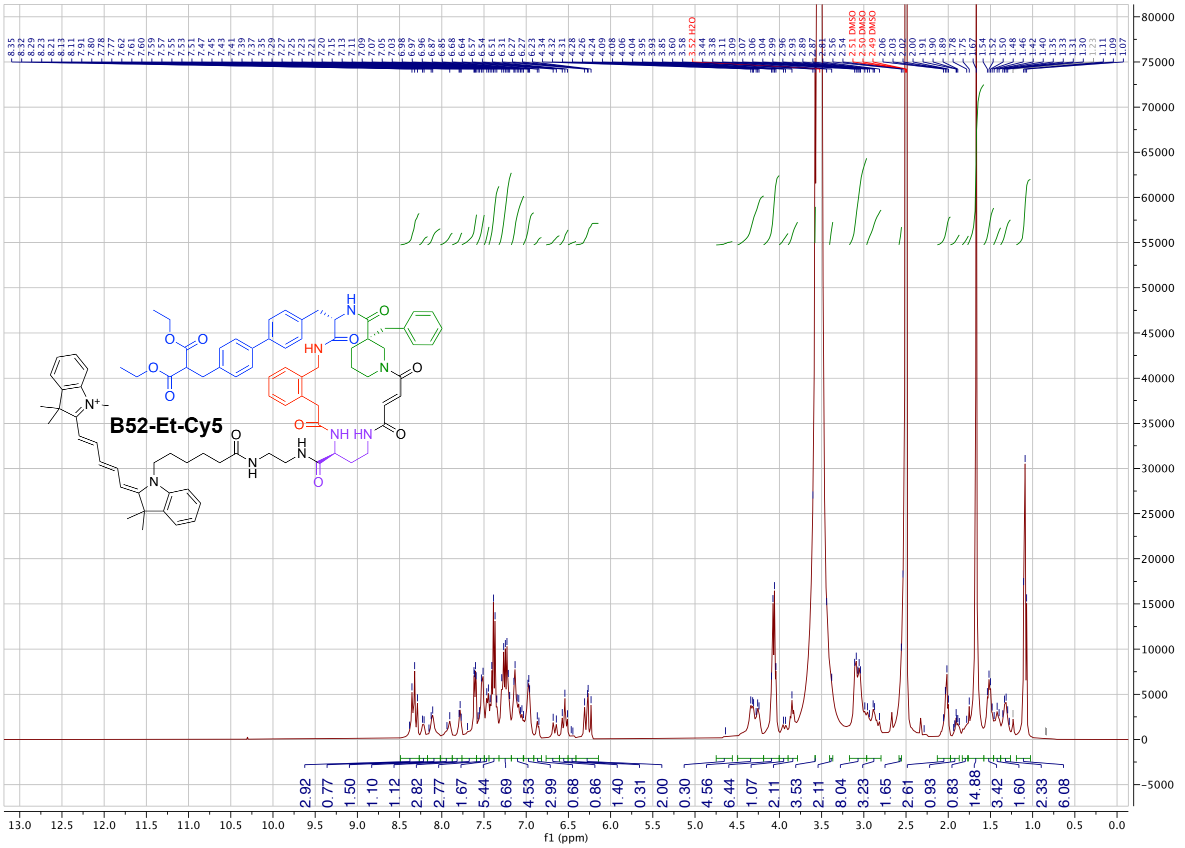
B52-Et-Cy5
f1 (ppm)

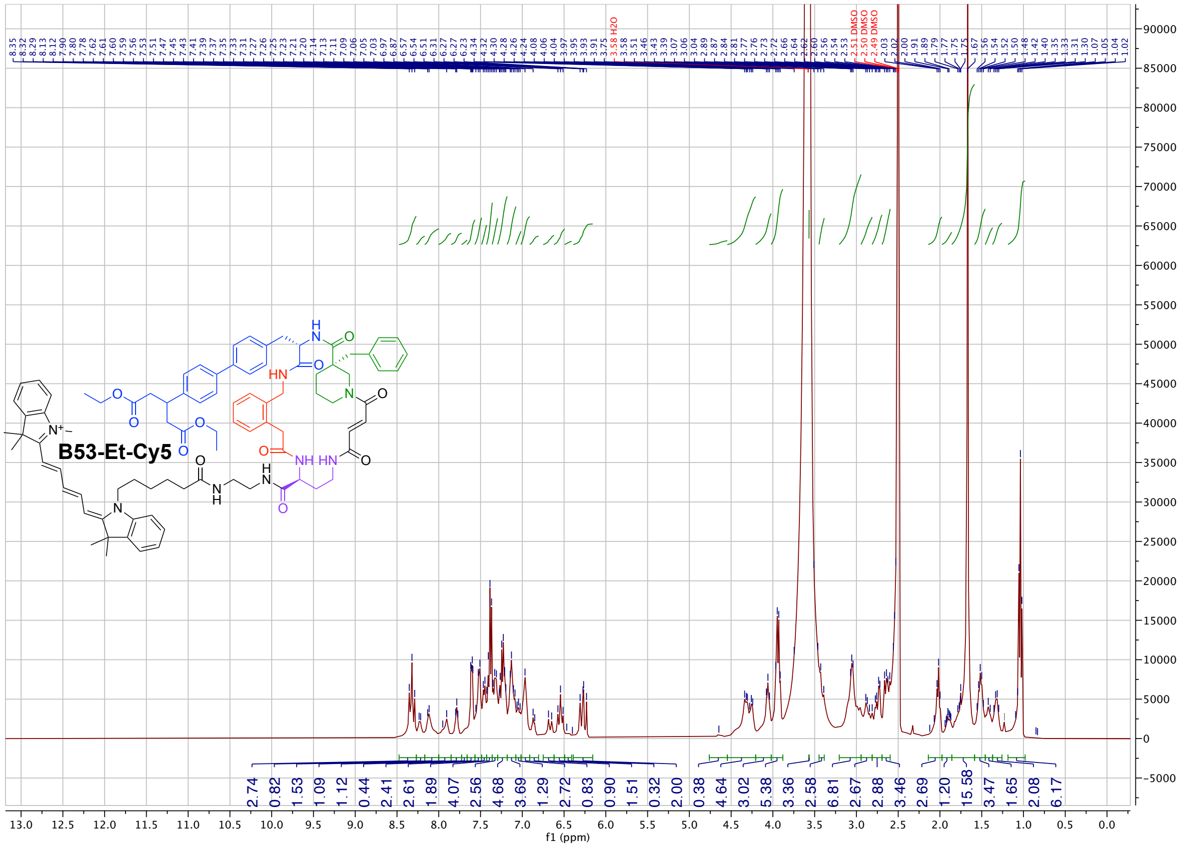
B53-Et-Cy5
f1 (ppm)

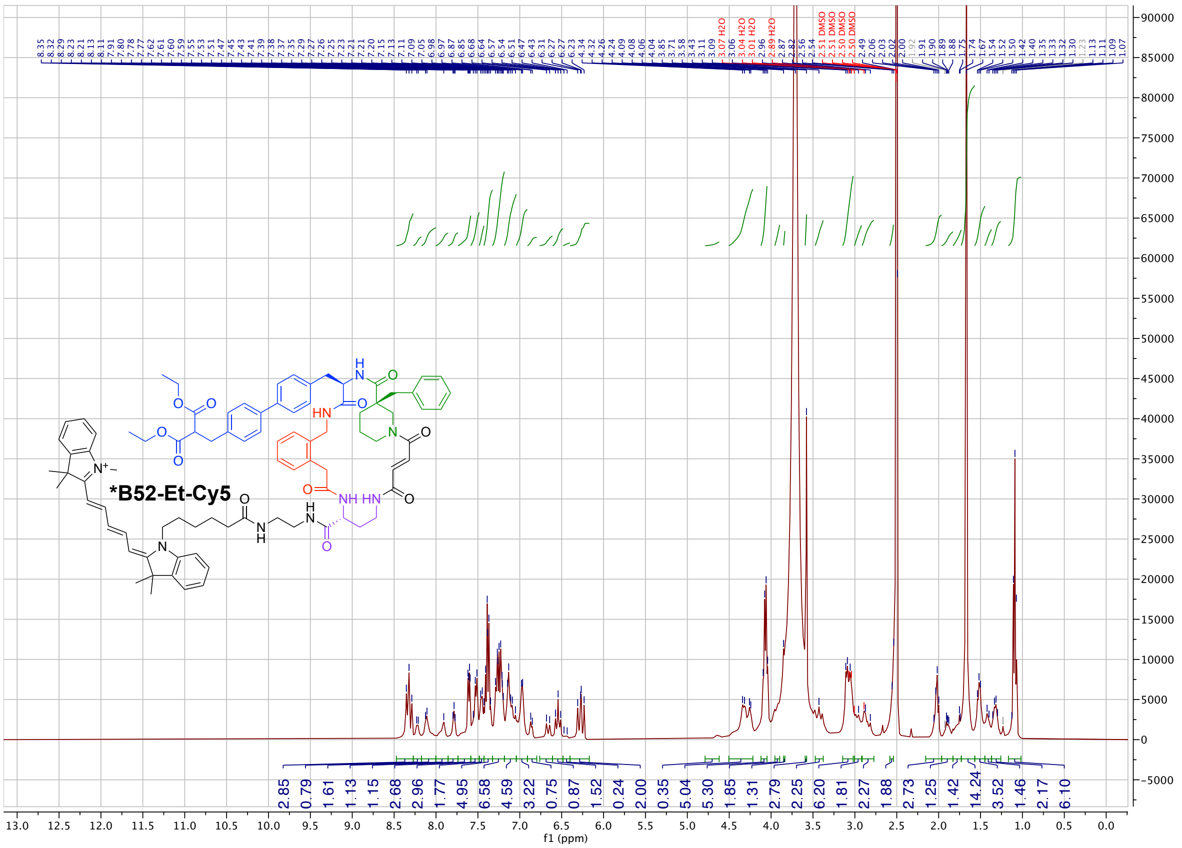
*B52-Et-Cy5
f1 (ppm)

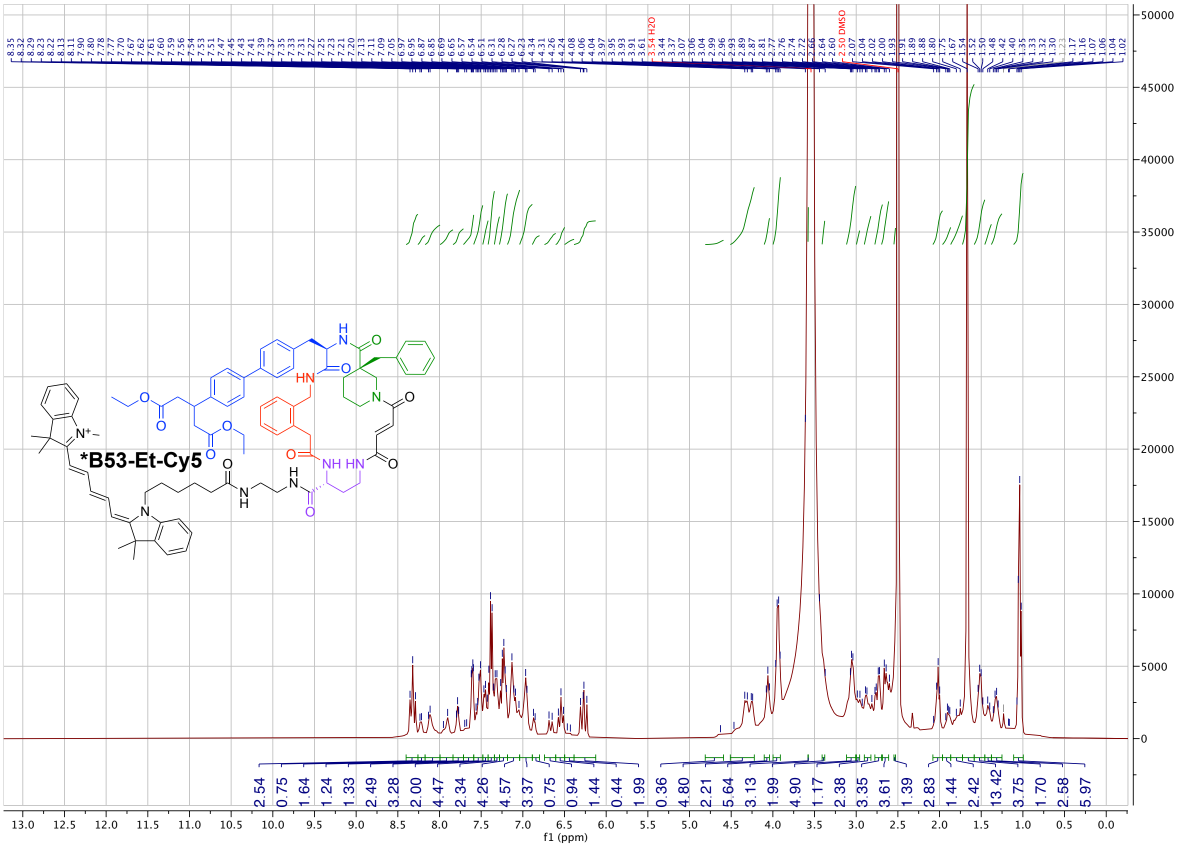
*B53-Et-Cy5
f1 (ppm)

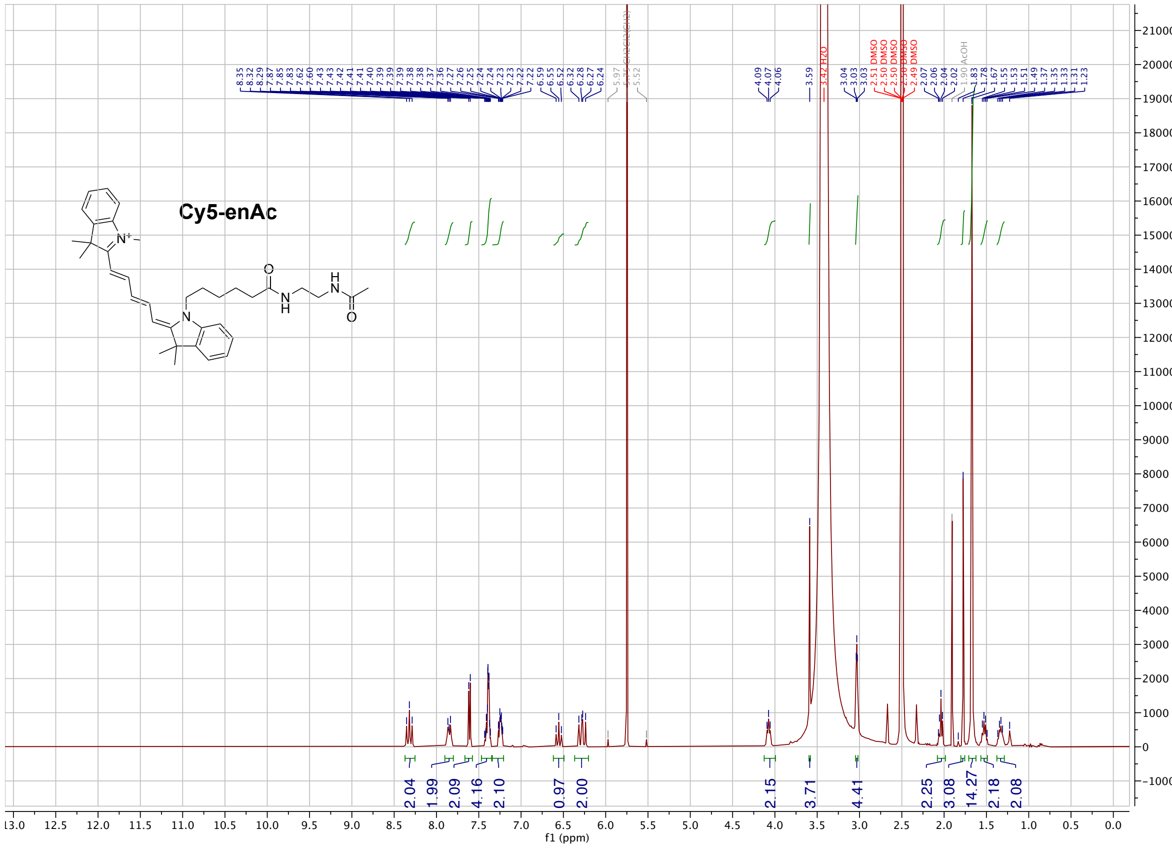
Cy5-enAc
f1 (ppm)

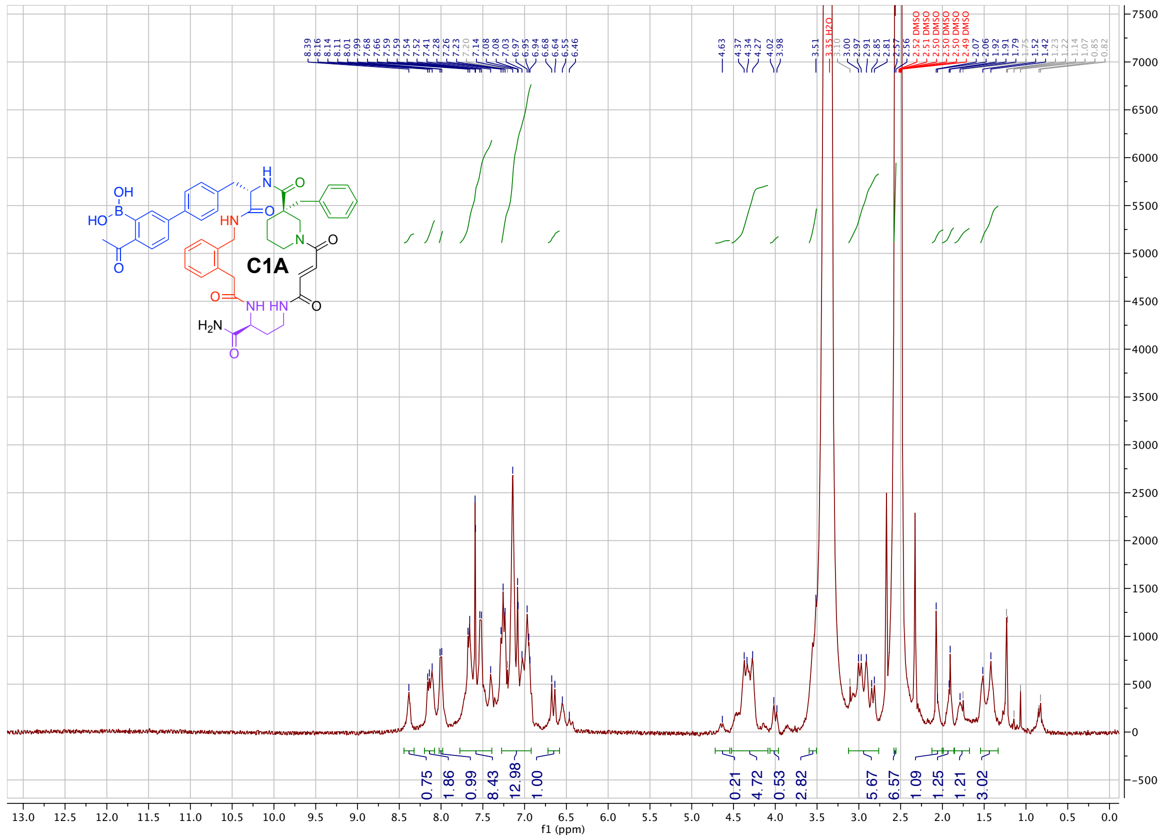
C1A
f1 (ppm)

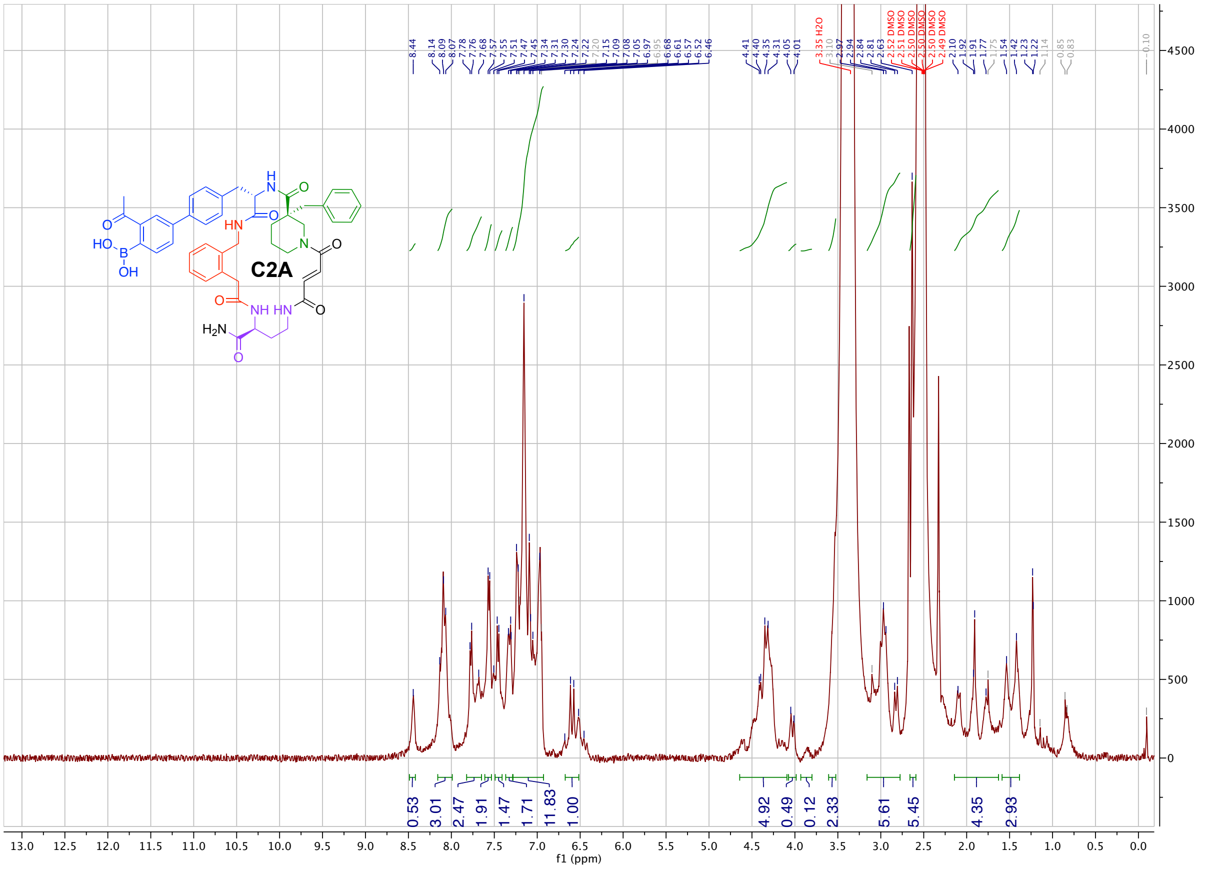
C2A
f1 (ppm)

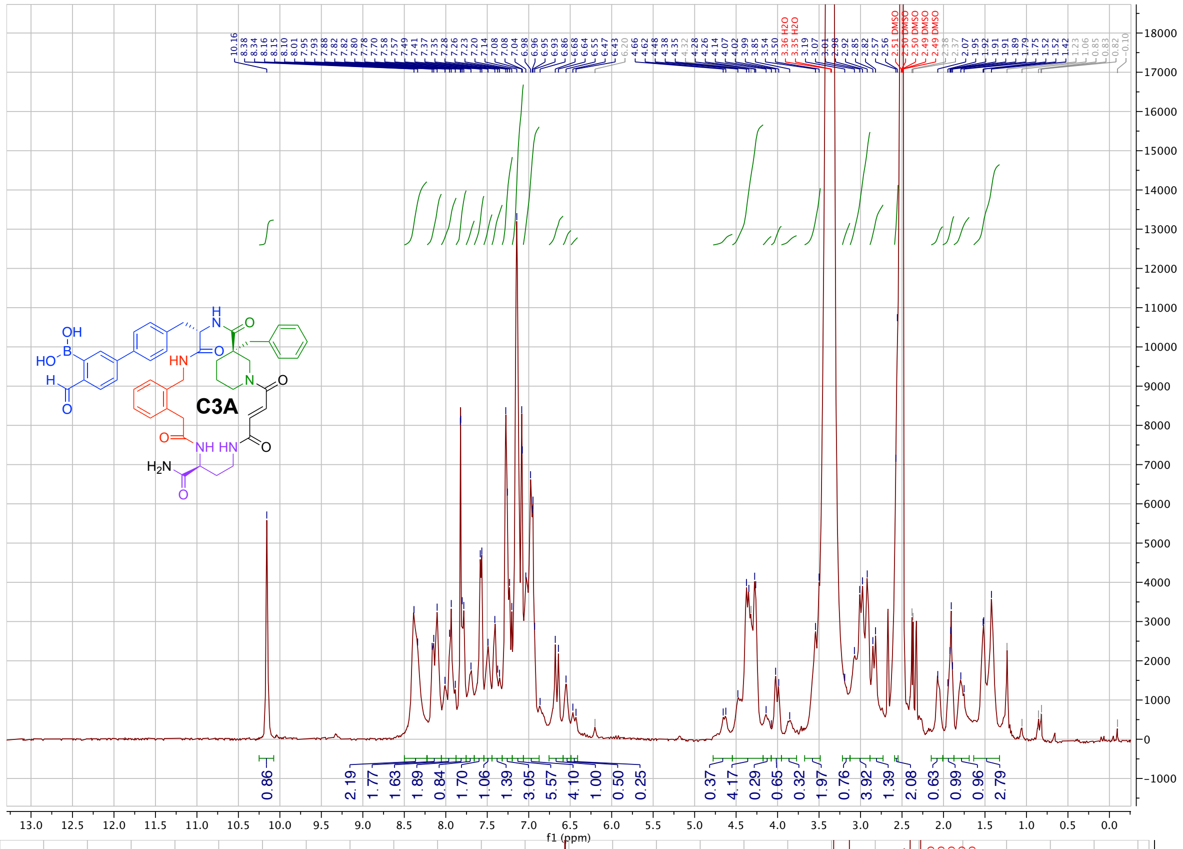
C3A
f1 (ppm)

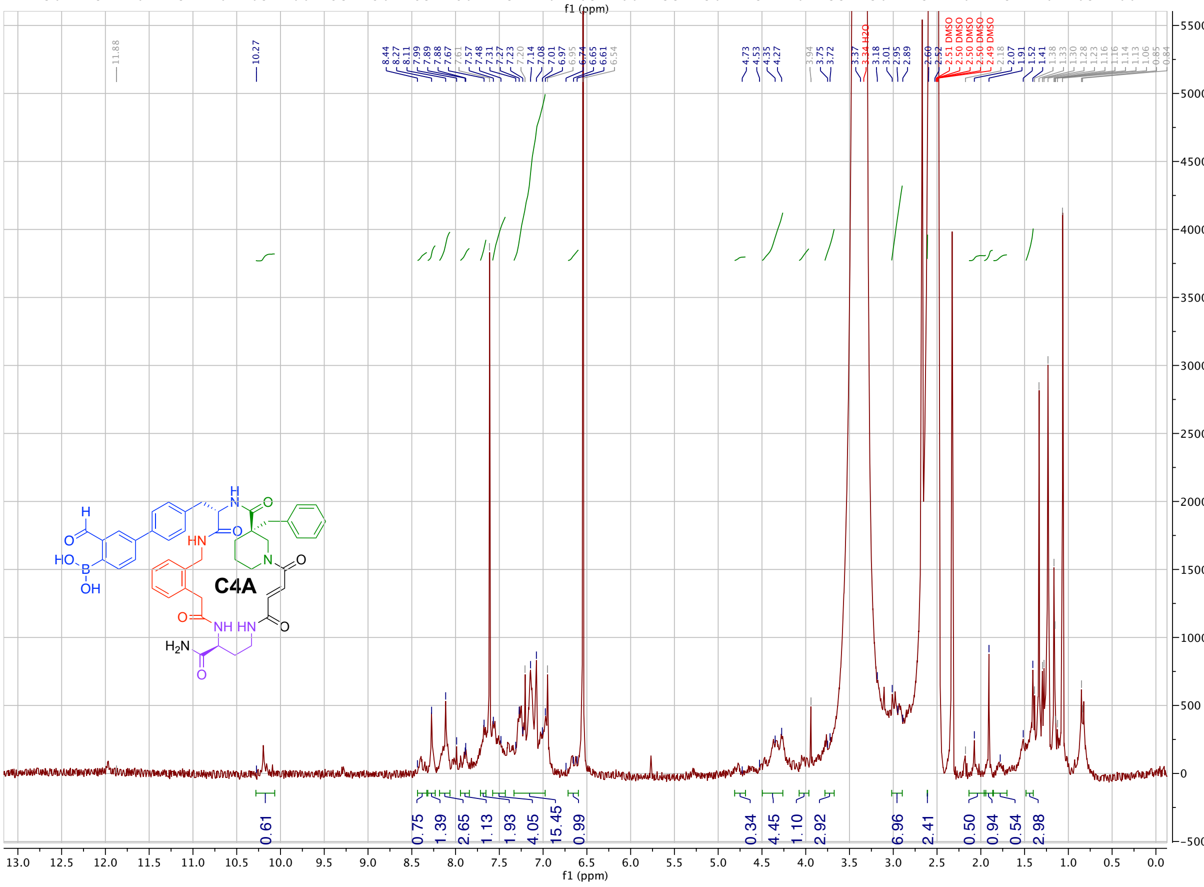
C4A
f1 (ppm)

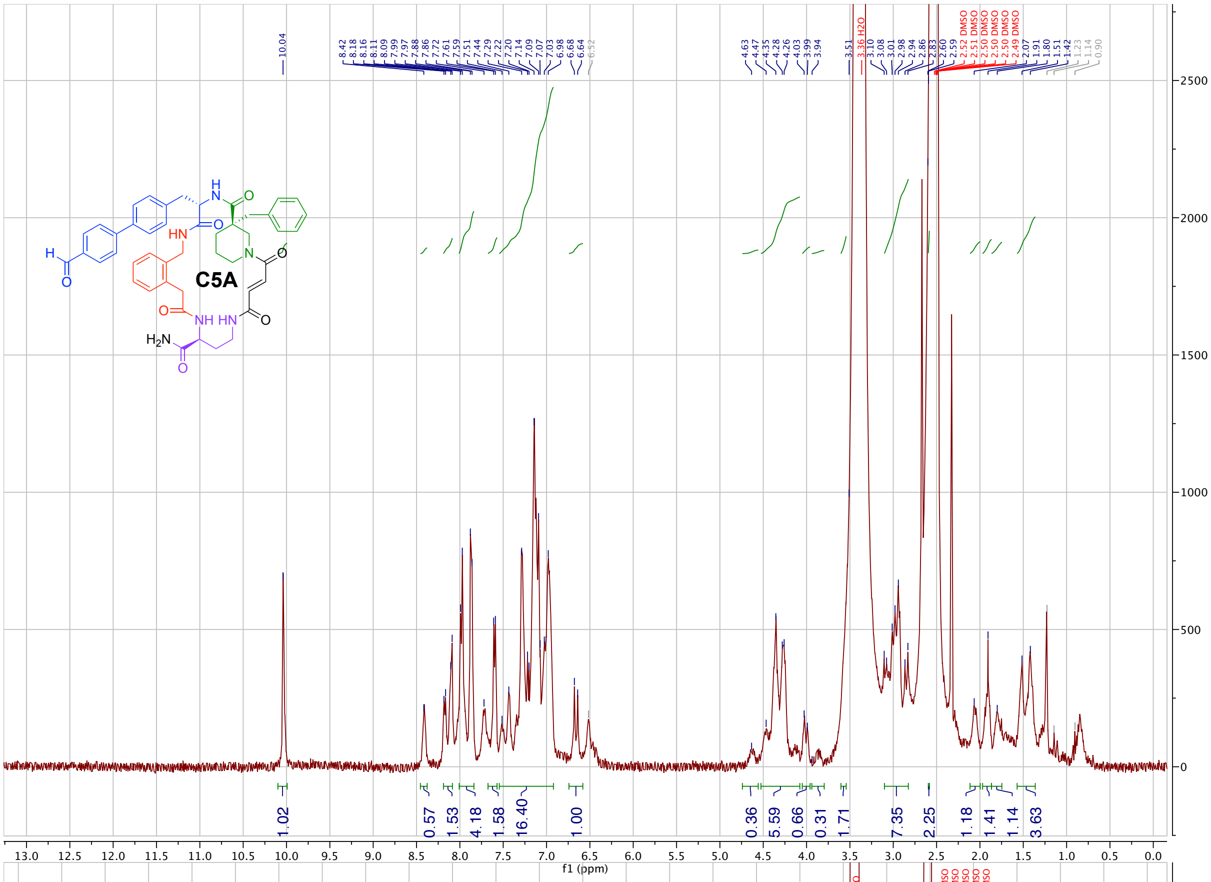
C5A
f1 (ppm)

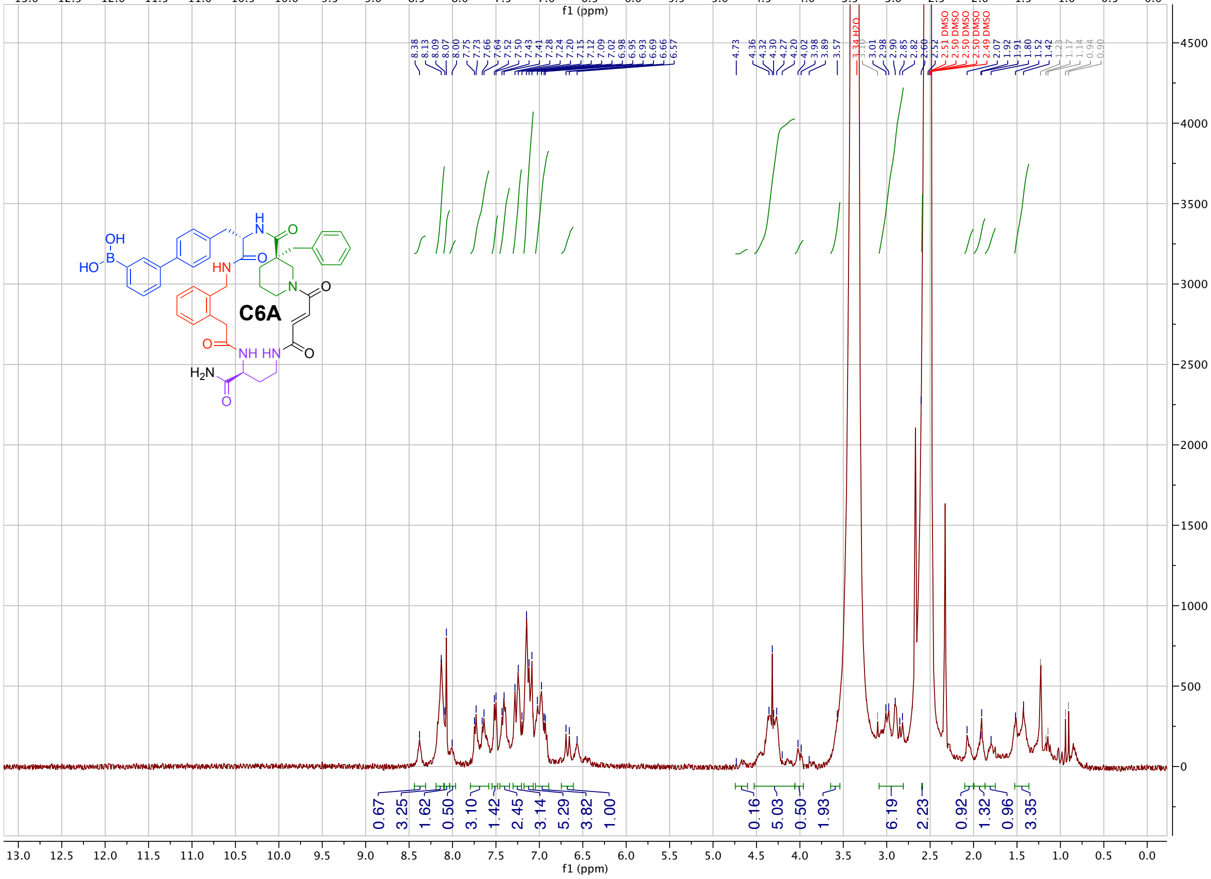
C6A
f1 (ppm)

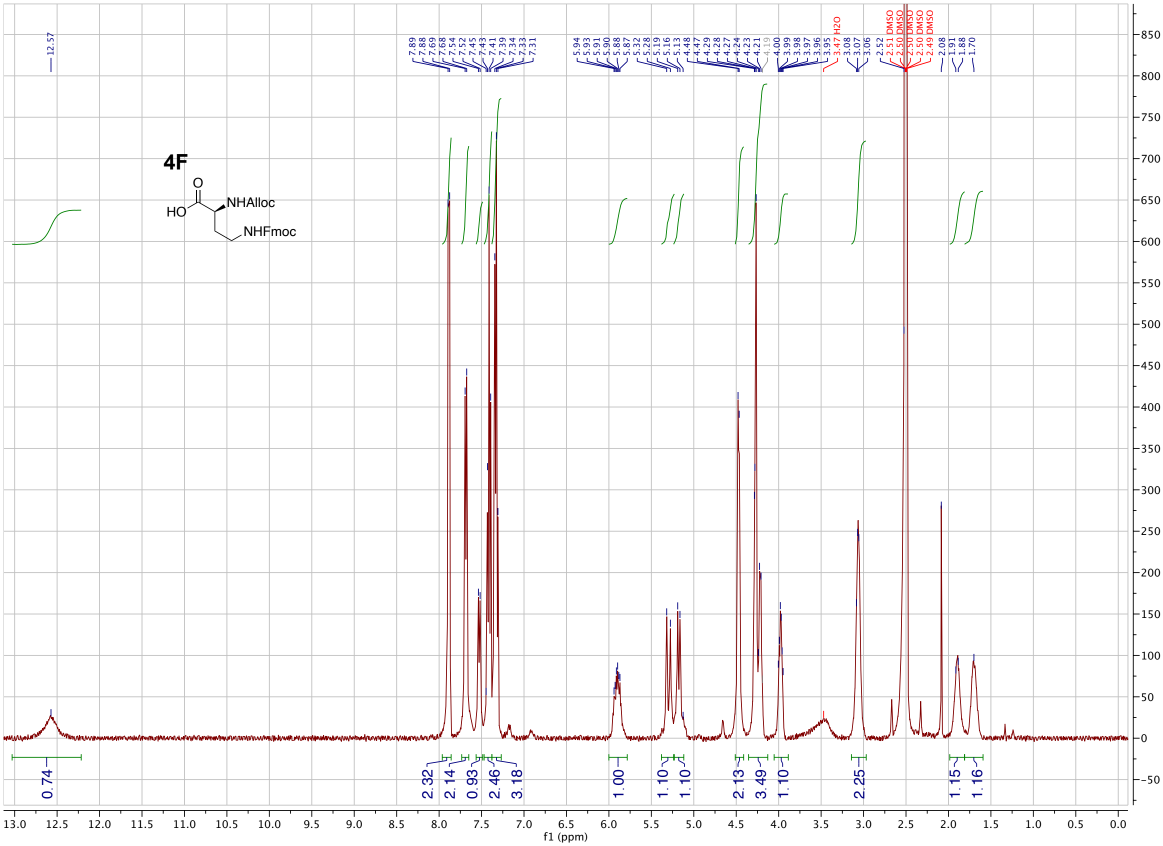
4F
HO
NHAlloc
NHFmoc
f1 (ppm)

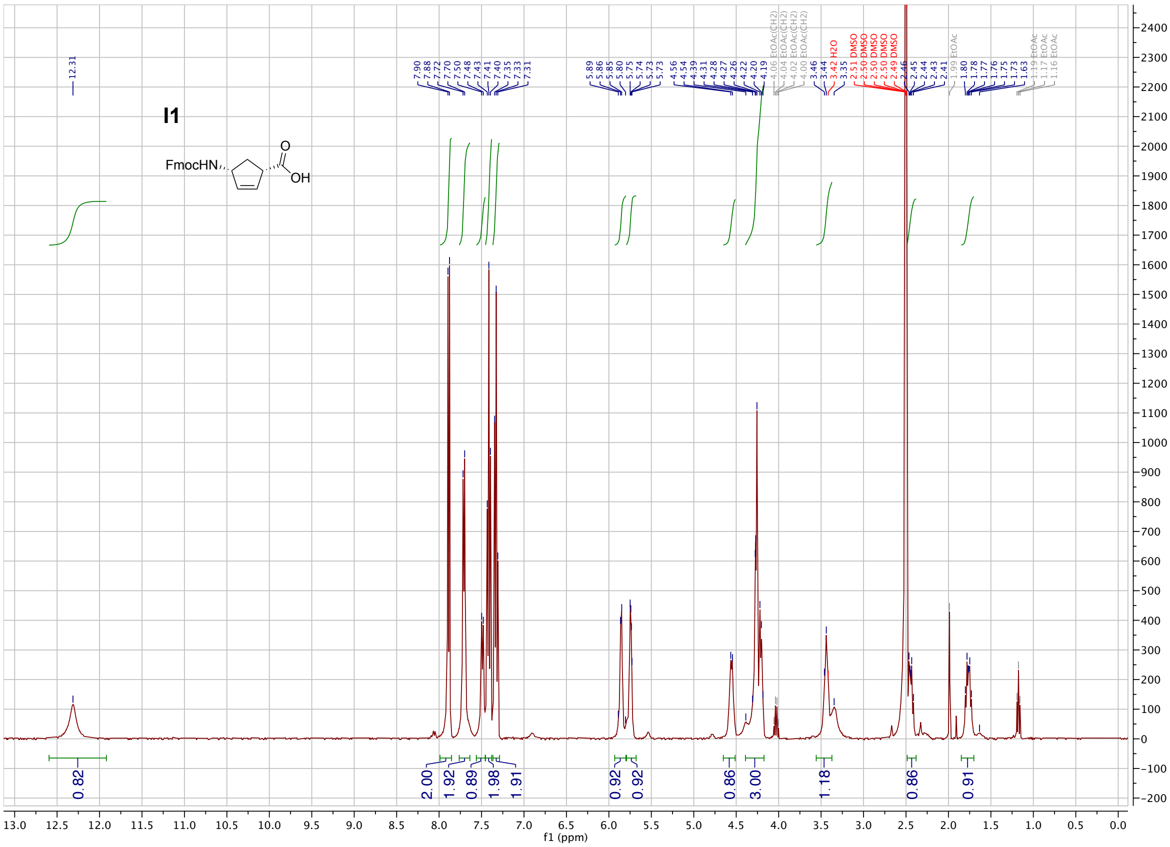
I1
FmocHN
OH
f1 (ppm)

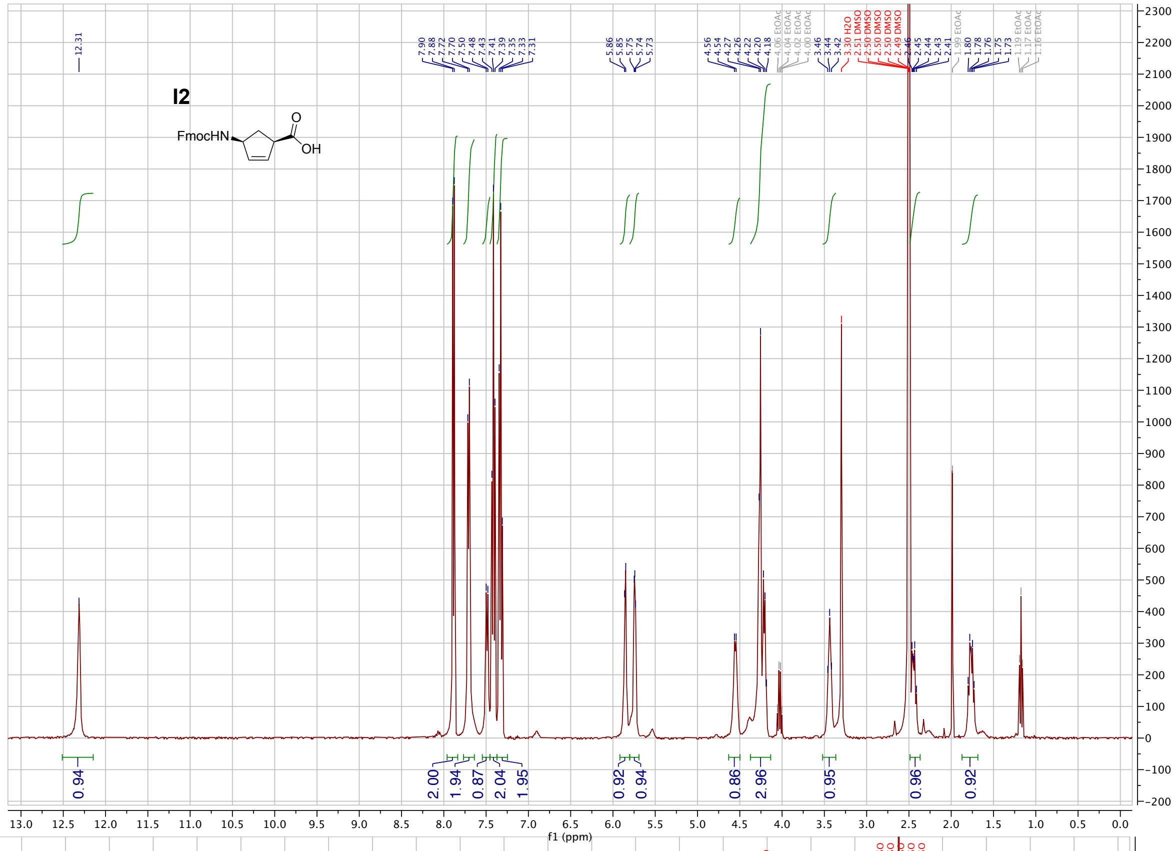
I2
FmocHN
O
OH
f1 (ppm)

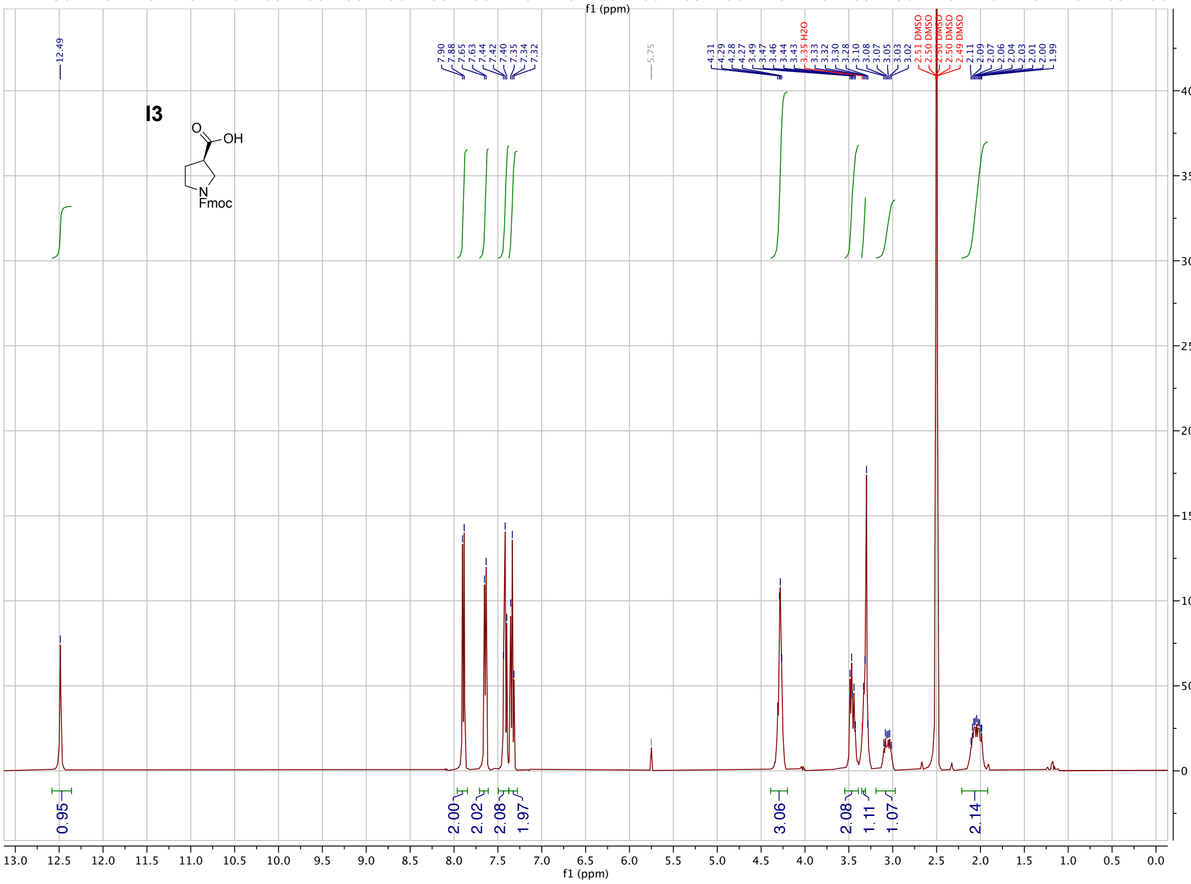
I3
O
OH
N
Fmoc
f1 (ppm)

I4

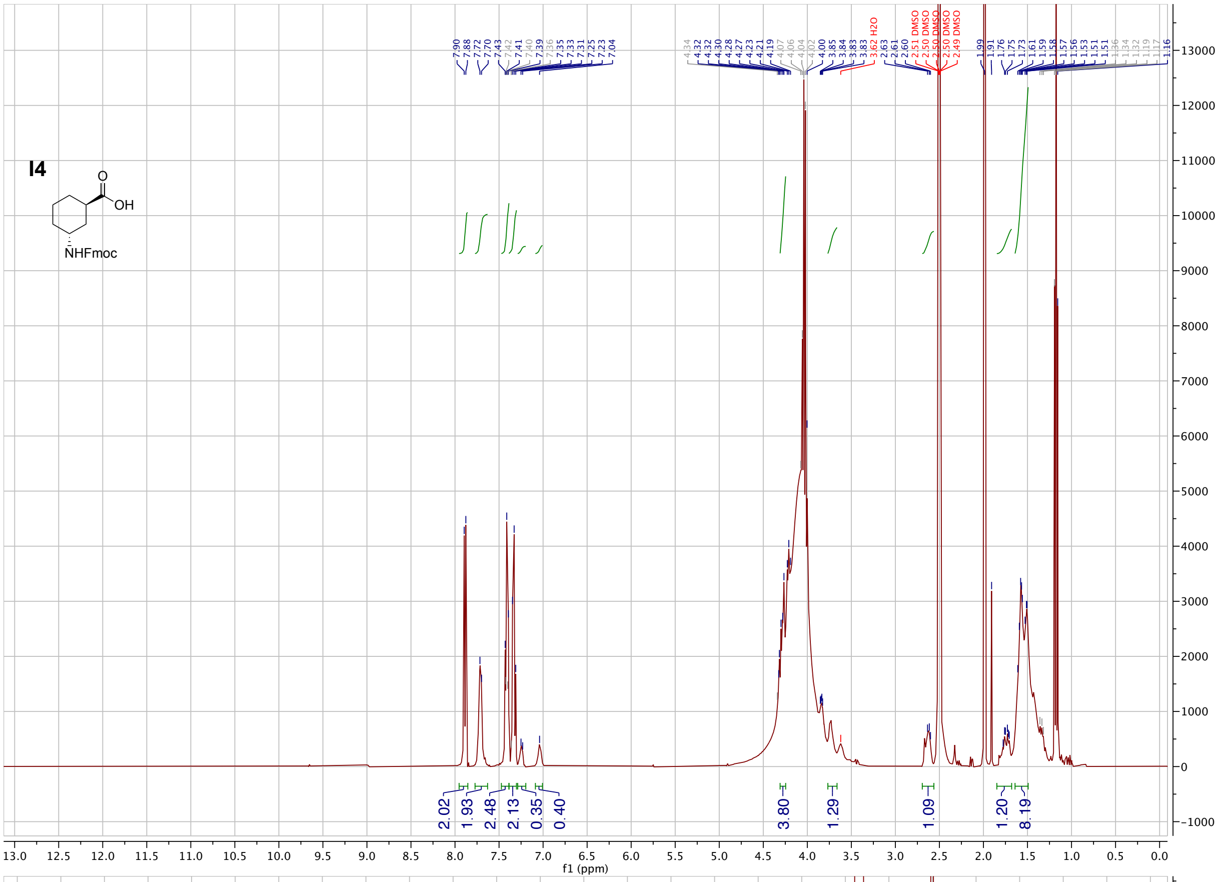

I5

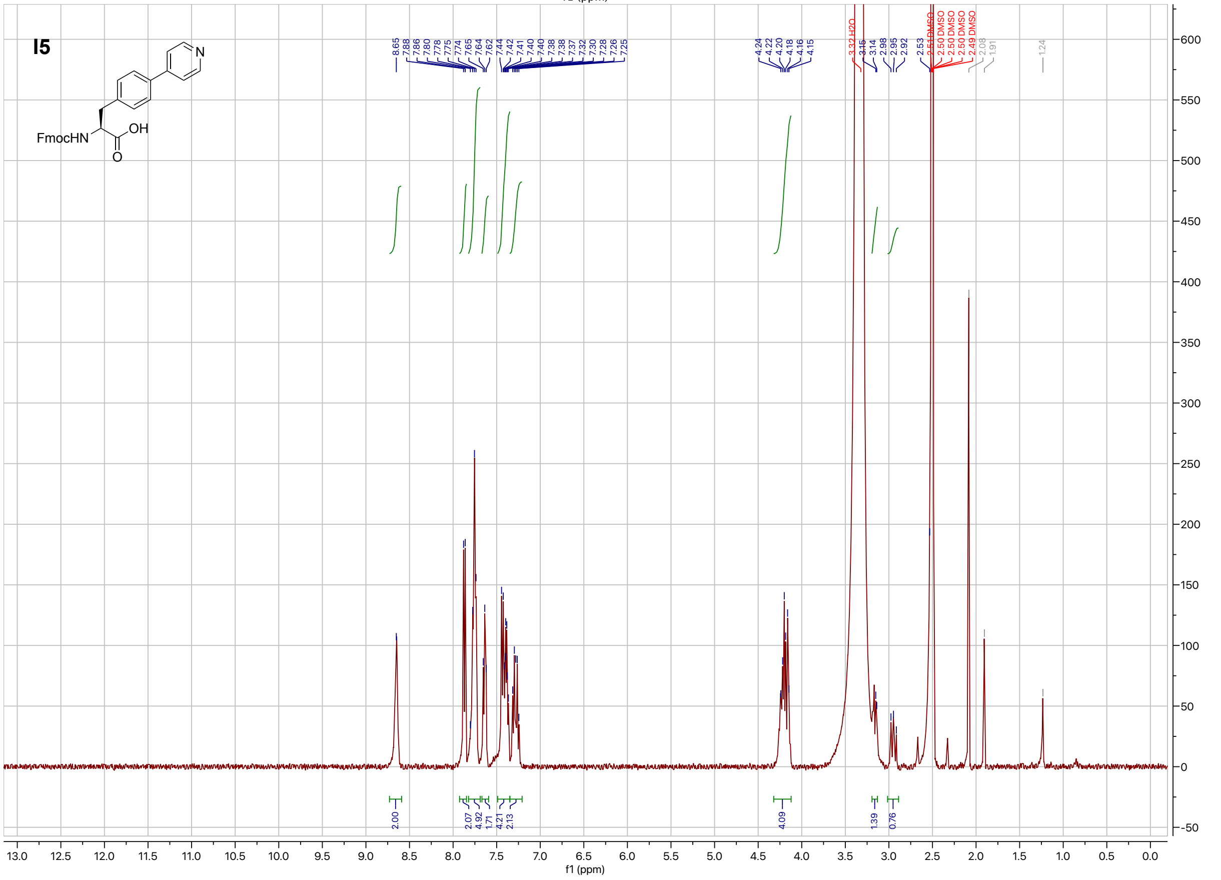

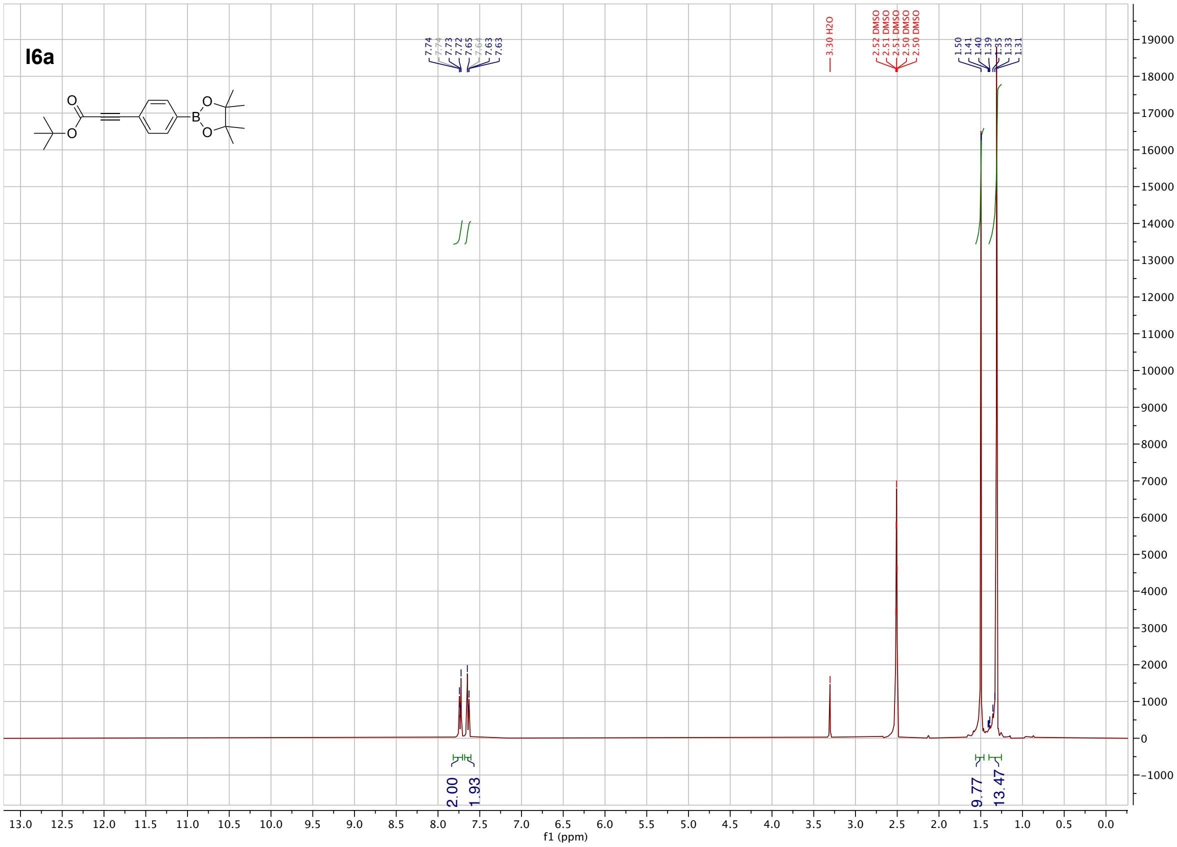
I6a
3.30 H2O
2.52 DMSO
2.51 DMSO
2.51 DMSO
2.50 DMSO
2.50 DMSO
2.00
1.93
9.77
13.47
f1 (ppm)

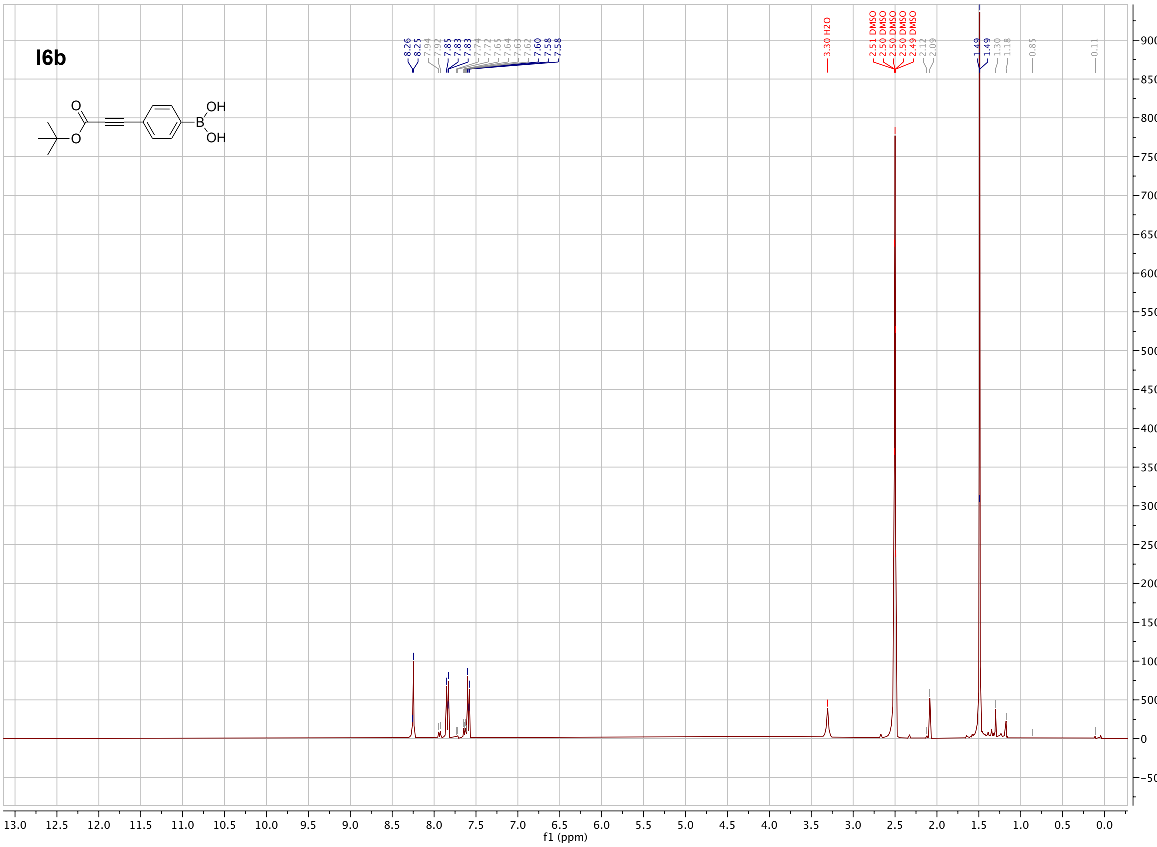
I6b
3.30 H2O
f1 (ppm)

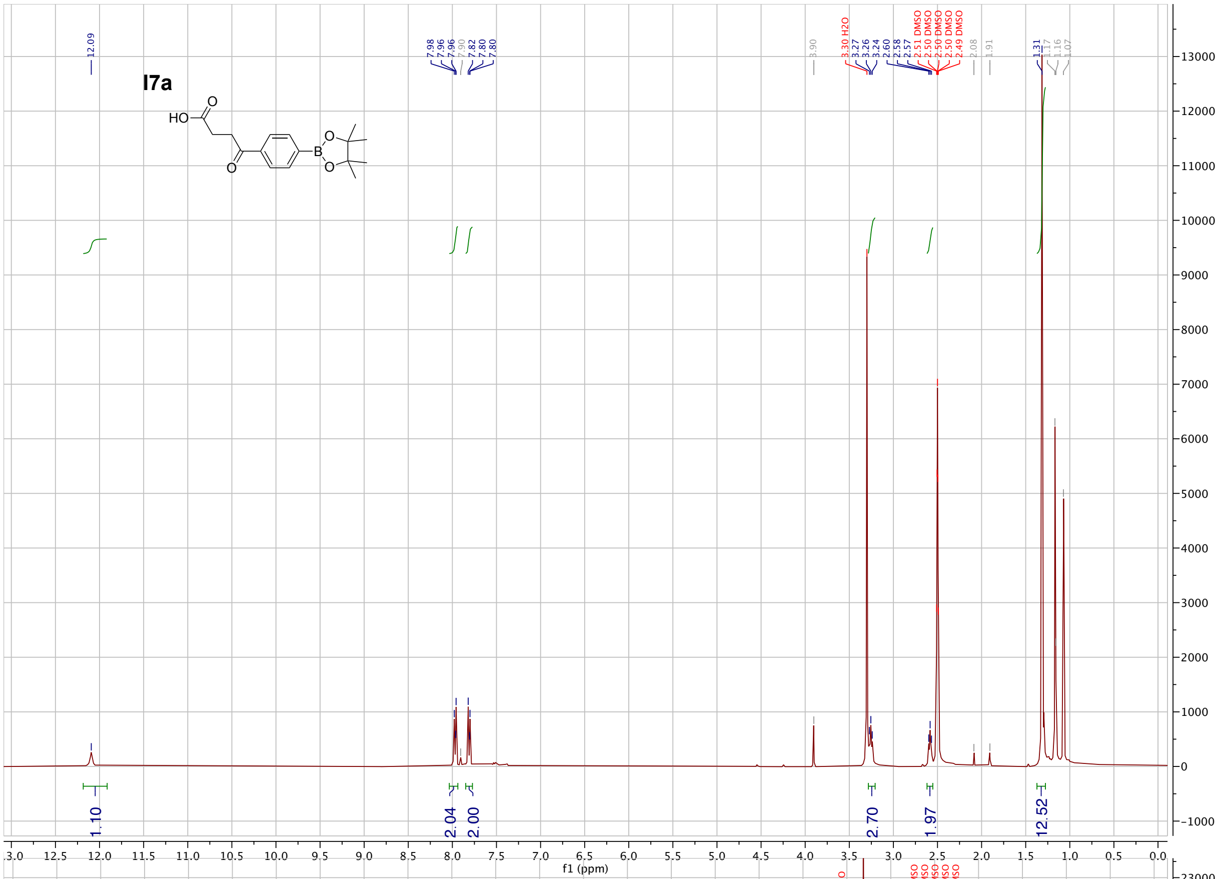
I7a
HO
O
O
B
O
O
12.09
7.98
7.96
7.96
7.90
7.82
7.80
7.80
3.90
3.30 H2O
3.27
3.26
3.24
2.60
2.58
2.57
2.51 DMSO
2.50 DMSO
2.50 DMSO
2.50 DMSO
2.49 DMSO
2.08
1.91
1.31
1.17
1.16
1.07
1.10
2.04
2.00
2.70
1.97
12.52
f1 (ppm)

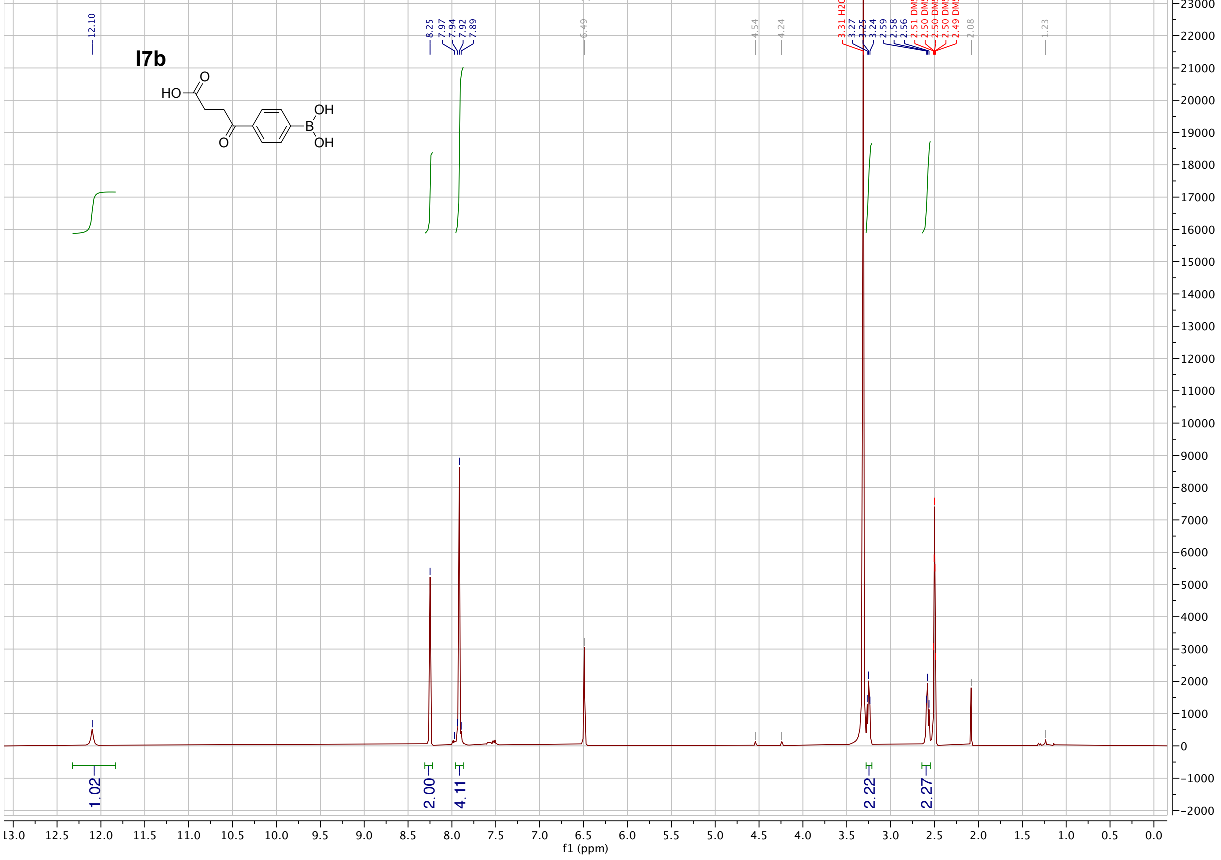
I7b
HO
O
O
B
OH
OH
12.10
8.25
7.97
7.94
7.92
7.89
6.49
4.54
4.24
3.31 H2O
3.27
3.24
2.59
2.58
2.56
2.51 DMSO
2.50 DMSO
2.50 DMSO
2.50 DMSO
2.49 DMSO
2.08
1.23
1.02
2.00
4.11
2.22
2.27
f1 (ppm)

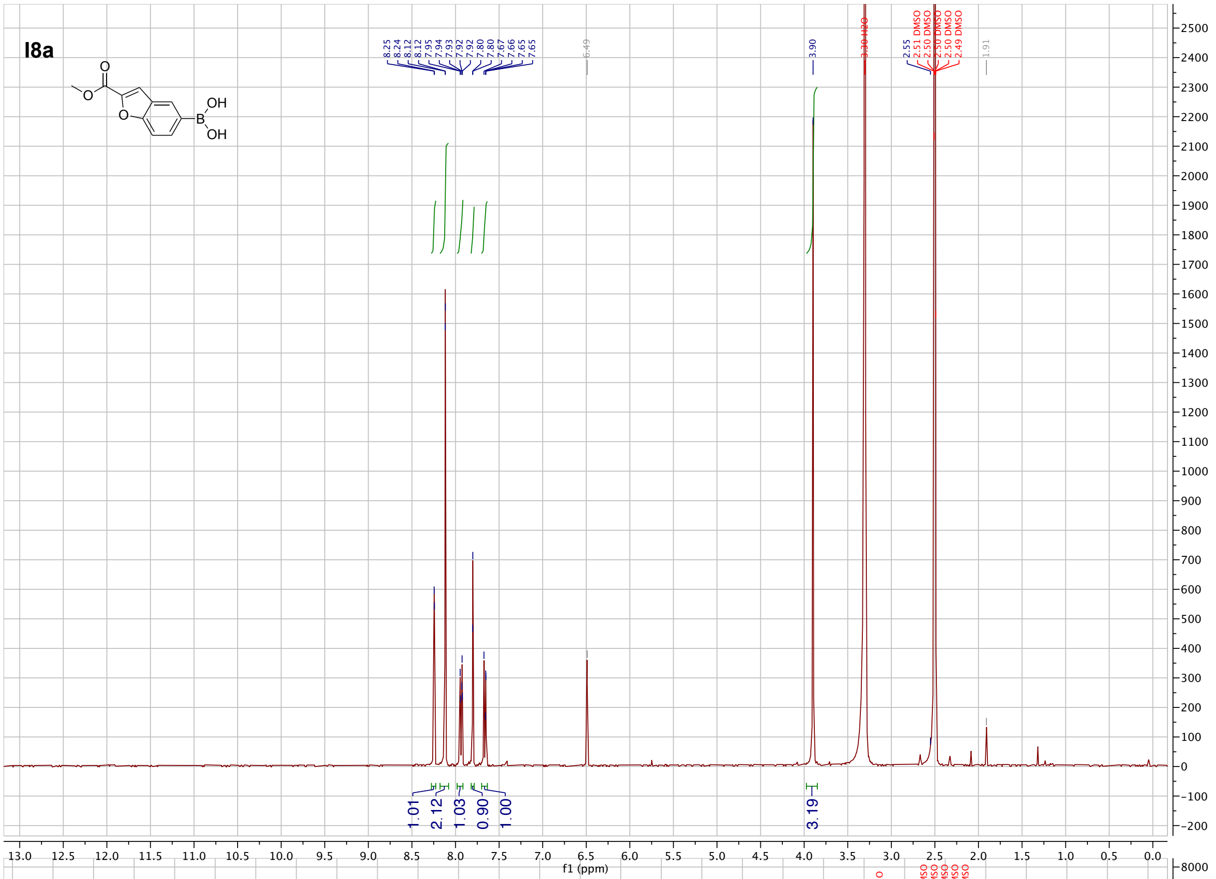
I8a
f1 (ppm)

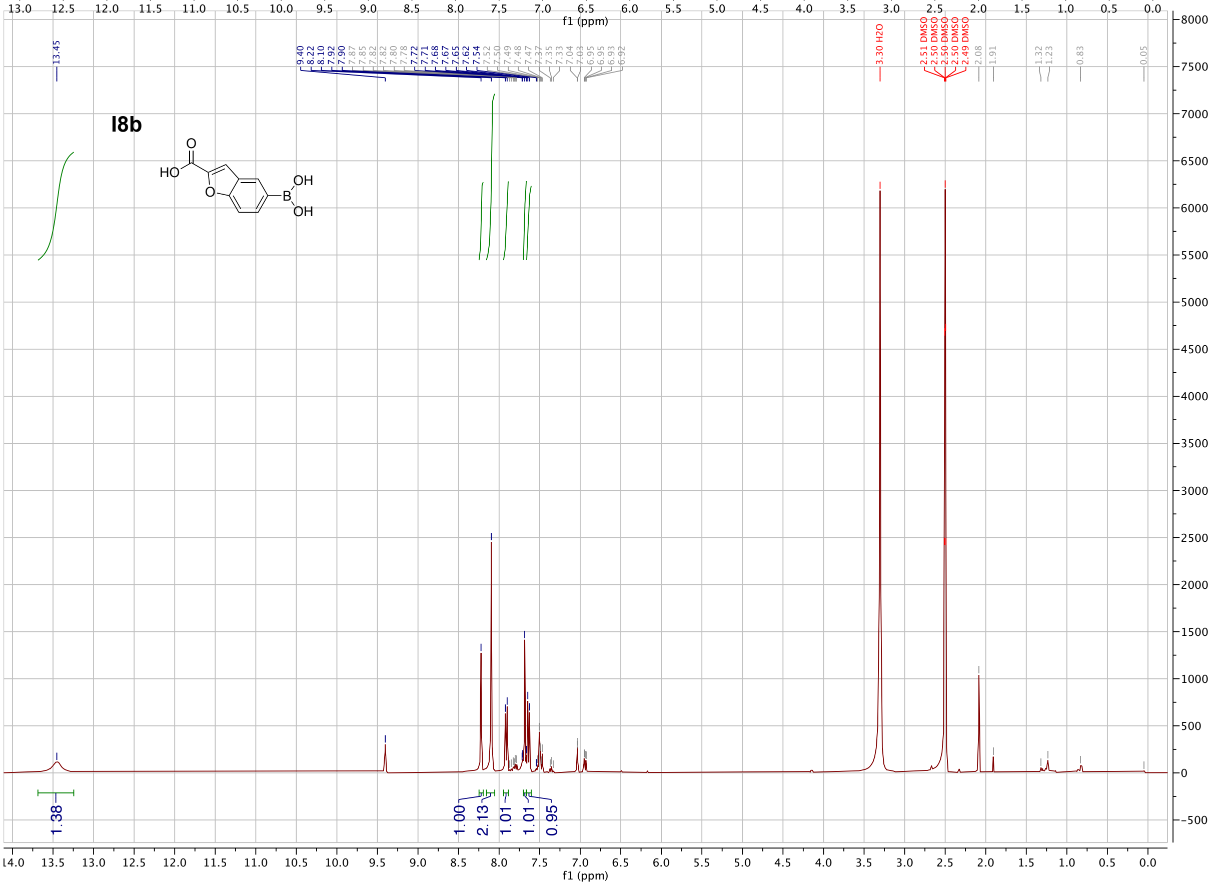
I8b
f1 (ppm)

I9a

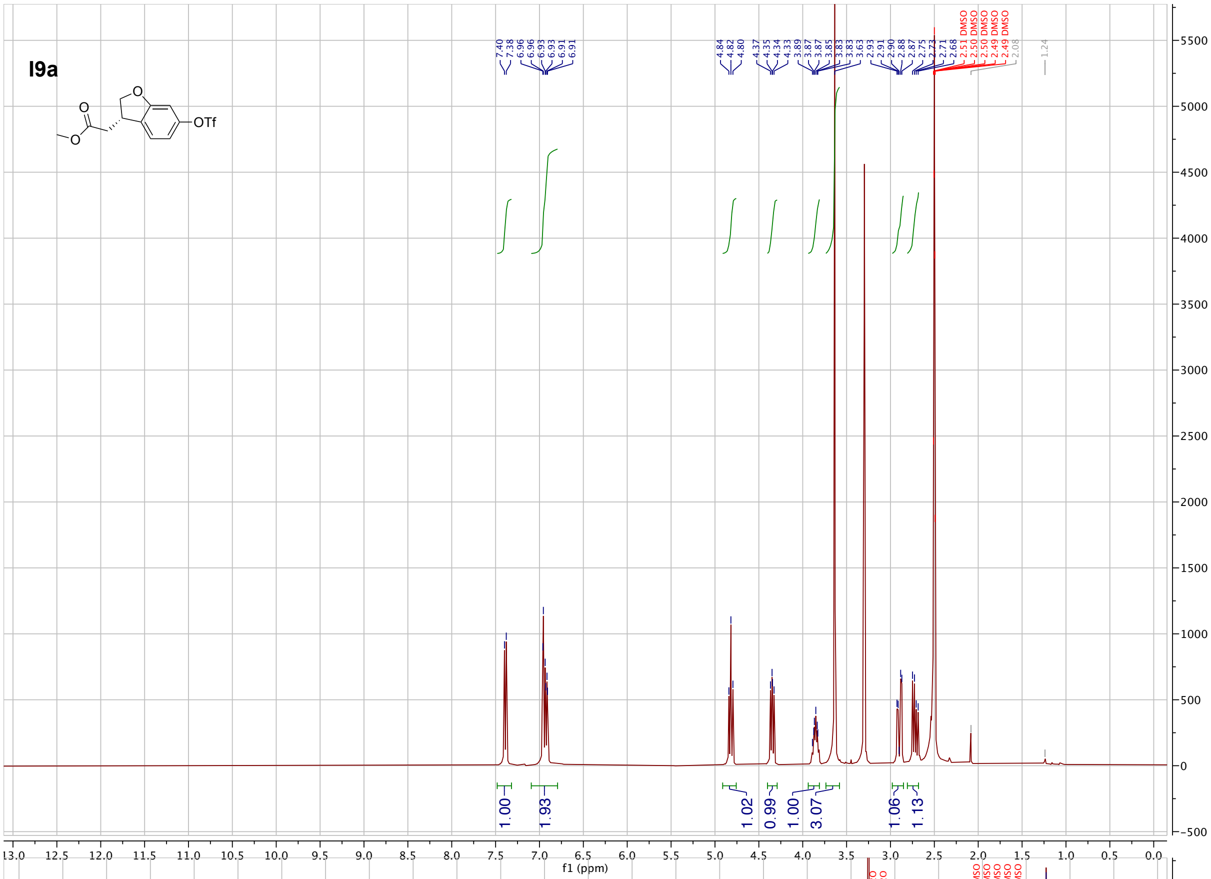

OTf
7.40
7.38
6.96
6.96
6.93
6.93
6.91
6.91
4.84
4.82
4.80
4.37
4.35
4.34
4.33
3.89
3.87
3.87
3.85
3.83
3.83
3.63
2.93
2.91
2.90
2.88
2.87
2.75
2.73
2.71
2.68
2.51 DMSO
2.50 DMSO
2.50 DMSO
2.49 DMSO
2.49 DMSO
2.08
1.24
1.00
1.93
1.02
0.99
1.00
3.07
1.06
1.13
f1 (ppm)

I9b

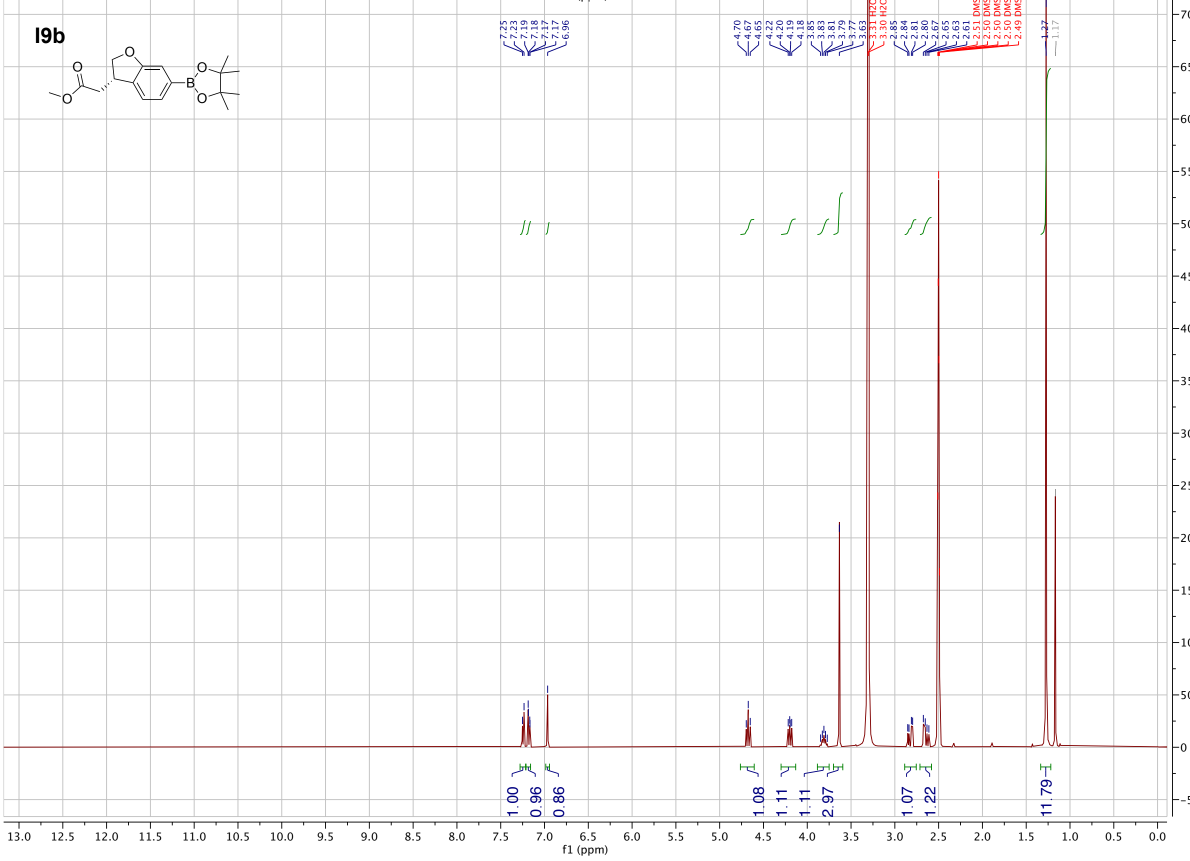

7.25
7.23
7.19
7.18
7.17
7.17
6.96
4.70
4.67
4.65
4.22
4.20
4.19
4.18
3.85
3.83
3.81
3.79
3.77
3.61
3.31 H2O
3.30 H2O
2.85
2.84
2.81
2.80
2.67
2.65
2.63
2.61
2.51 DMSO
2.50 DMSO
2.50 DMSO
2.50 DMSO
2.49 DMSO
1.27
1.17
1.00
0.96
0.86
1.08
1.11
1.11
2.97
1.07
1.22
11.79
f1 (ppm)

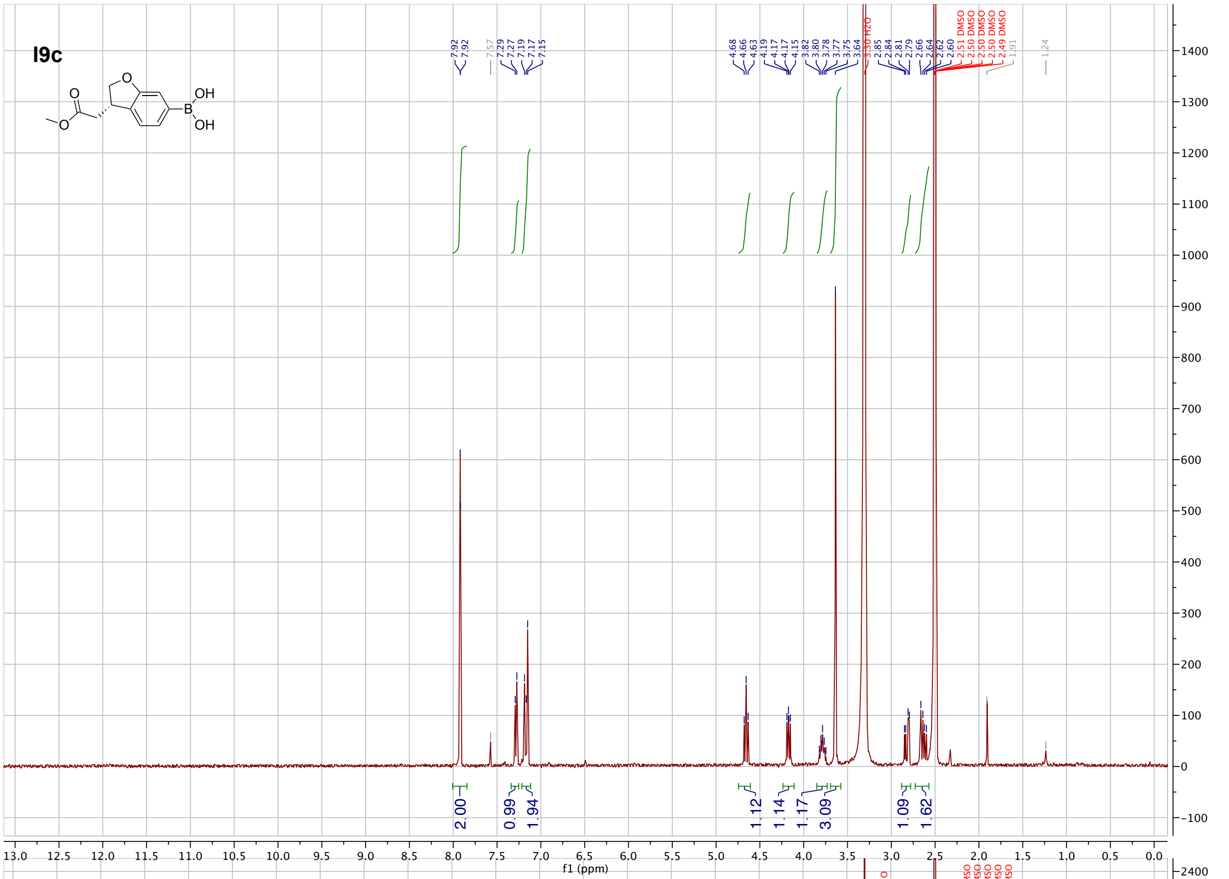
I9c
f1 (ppm)

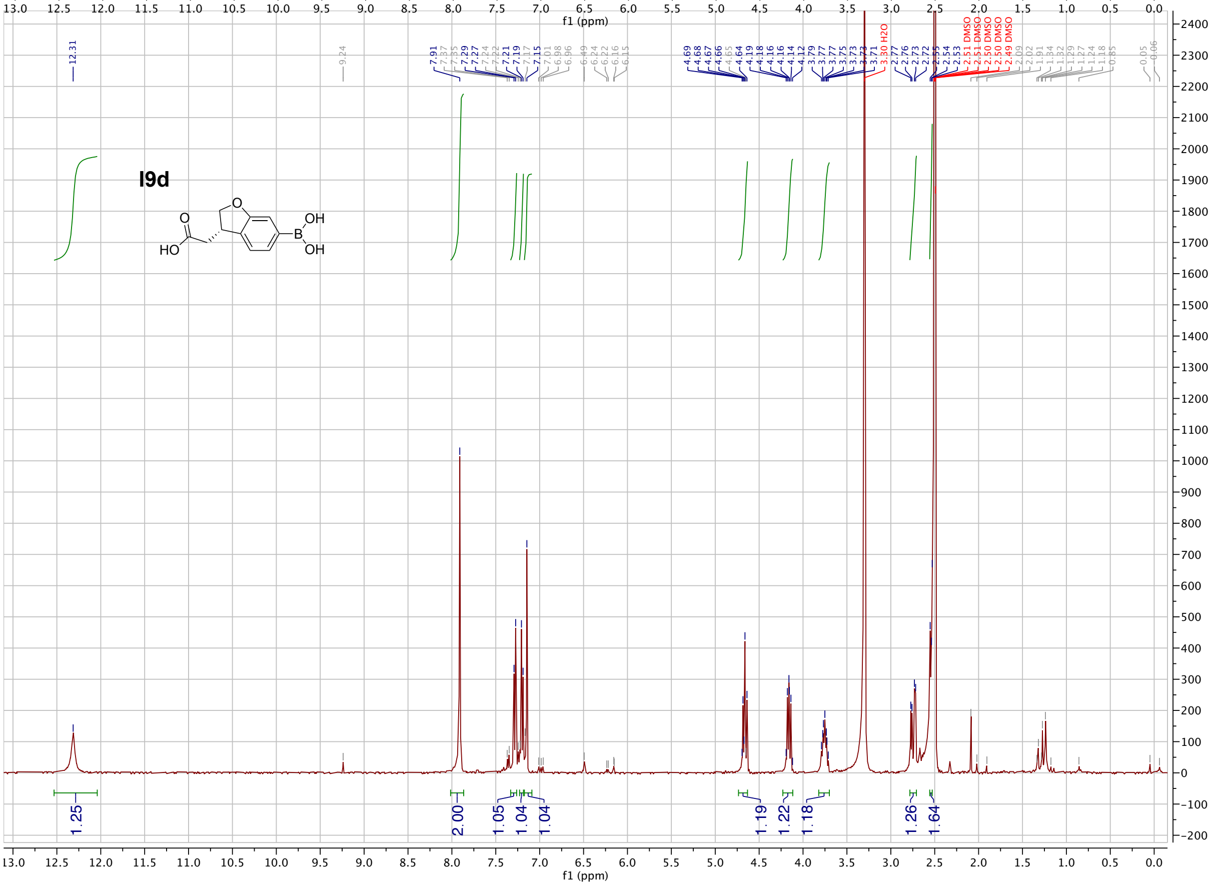
I9d
f1 (ppm)

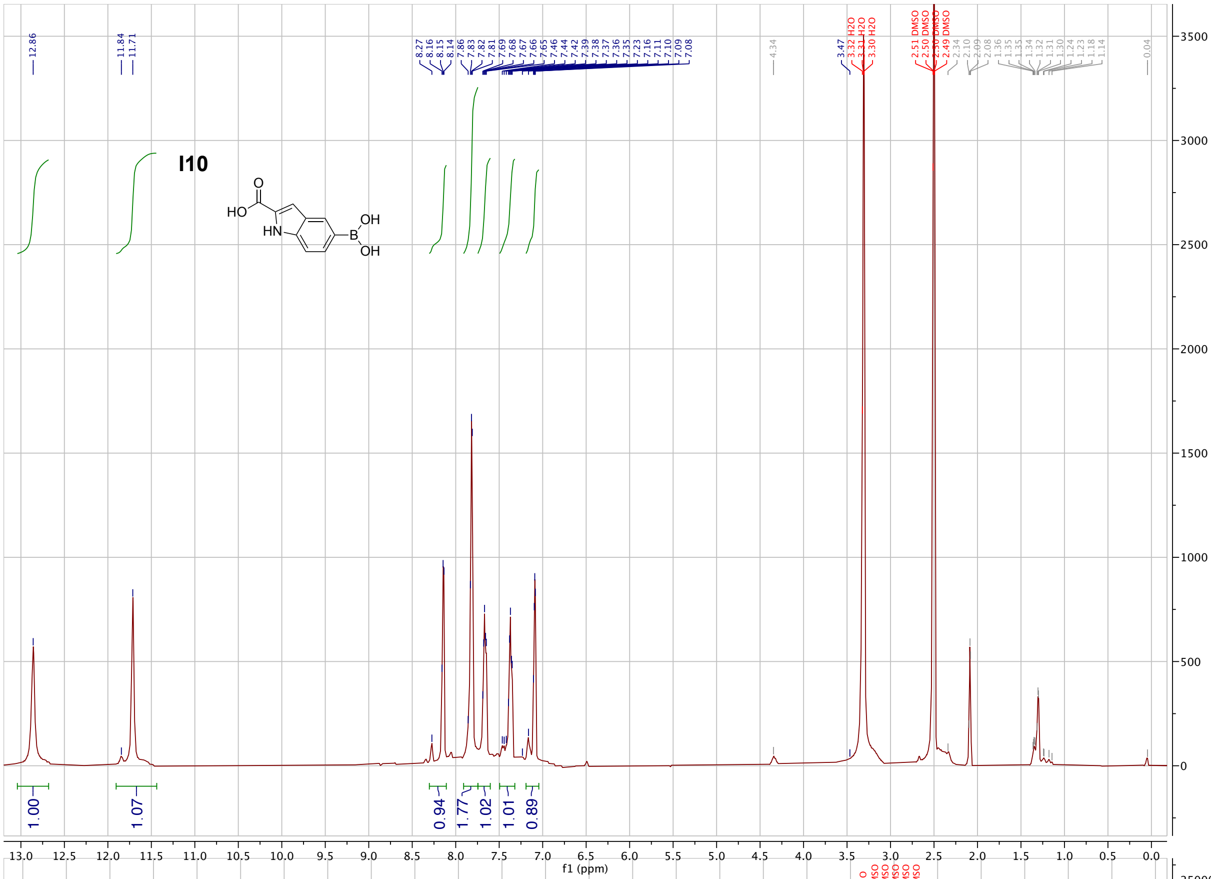
I10
12.86
11.84
11.71
8.27
8.16
8.15
8.14
7.86
7.83
7.82
7.81
3.47
3.32 H2O
3.31 H2O
3.30 H2O
2.51 DMSO
2.50 DMSO
2.50 DMSO
2.49 DMSO
1.00
1.07
0.94
1.77
1.02
1.01
0.89
f1 (ppm)

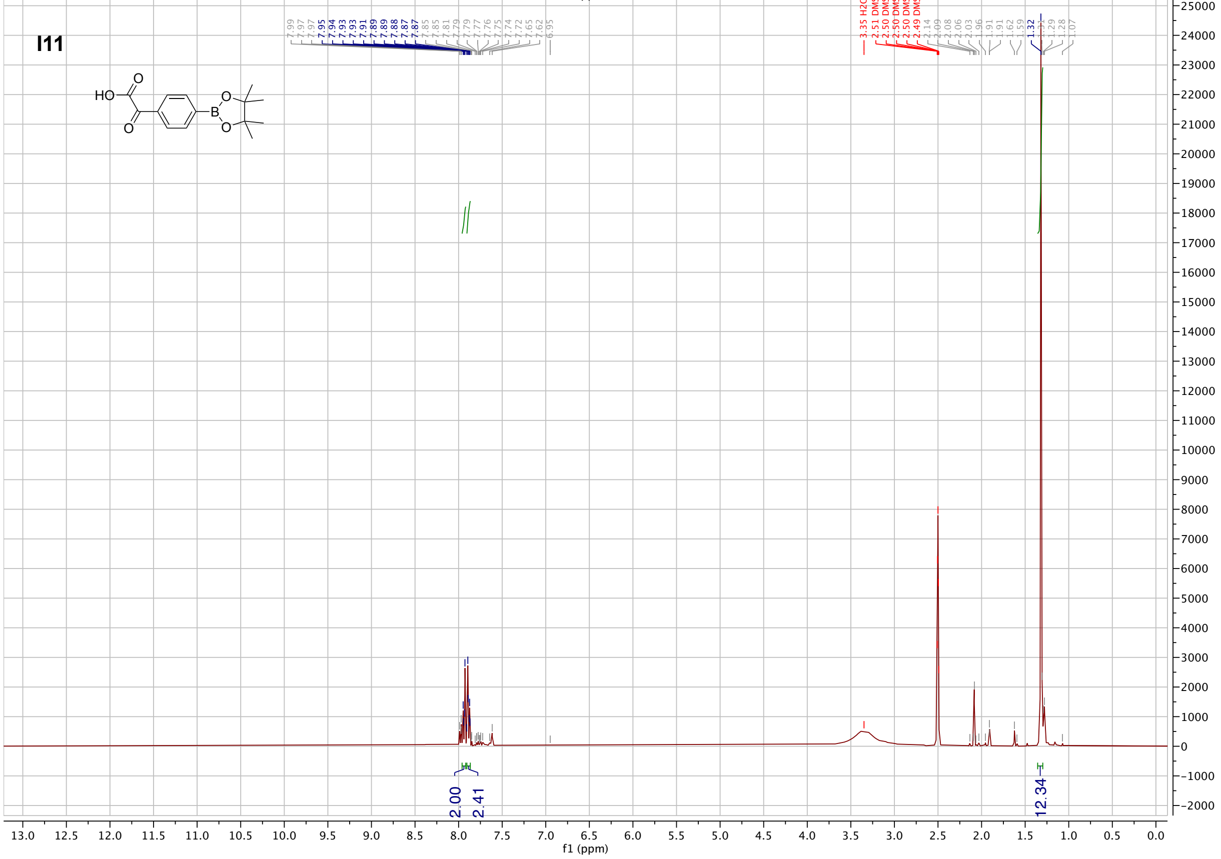
I11
3.35 H2O
2.51 DMSO
2.50 DMSO
2.50 DMSO
2.50 DMSO
2.49 DMSO
2.00
2.41
12.34
f1 (ppm)

I12a

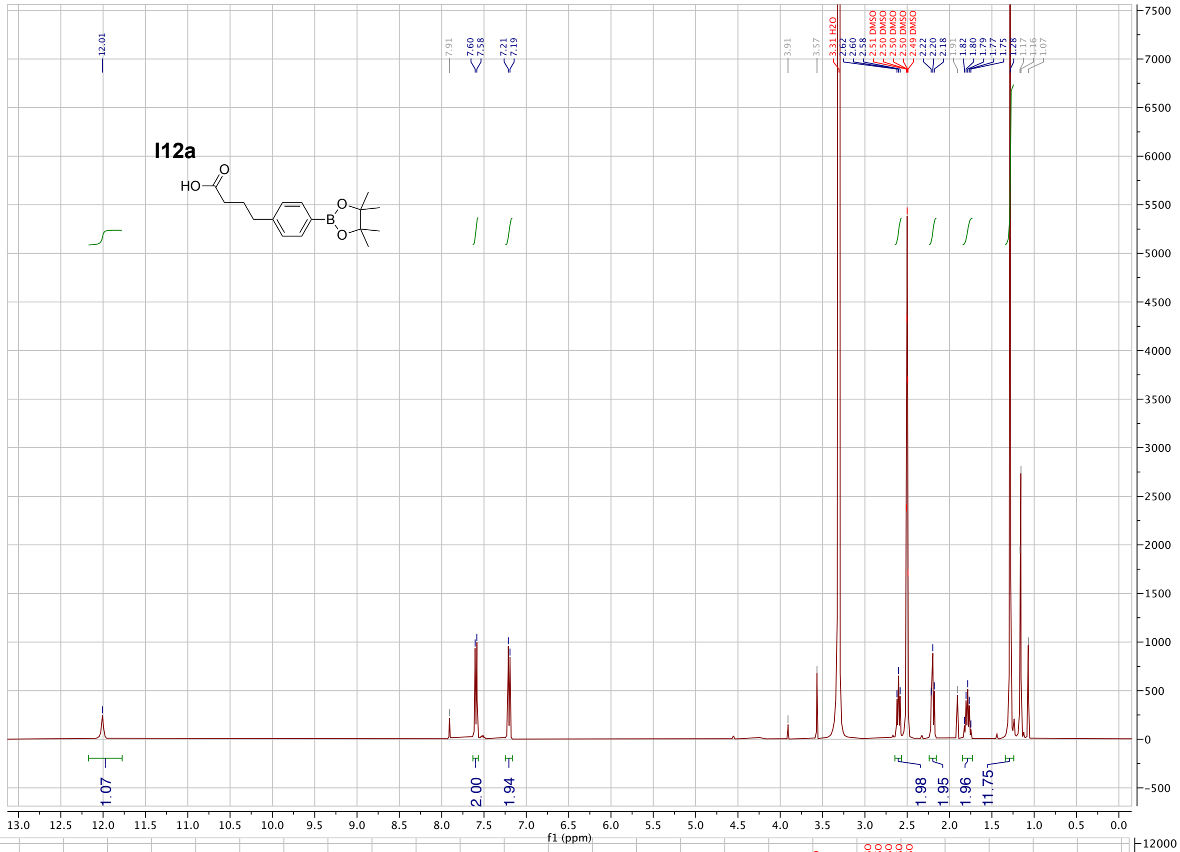

I12b

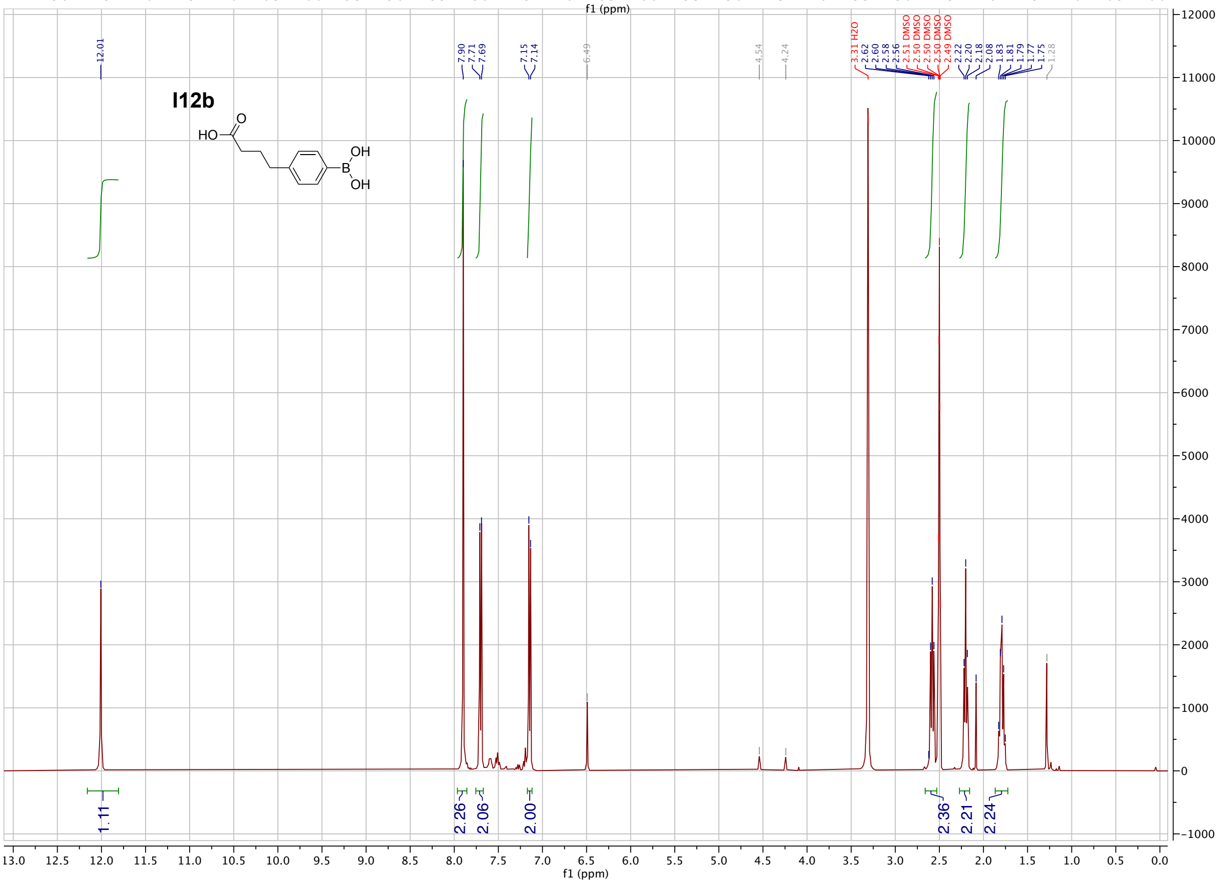

I13a

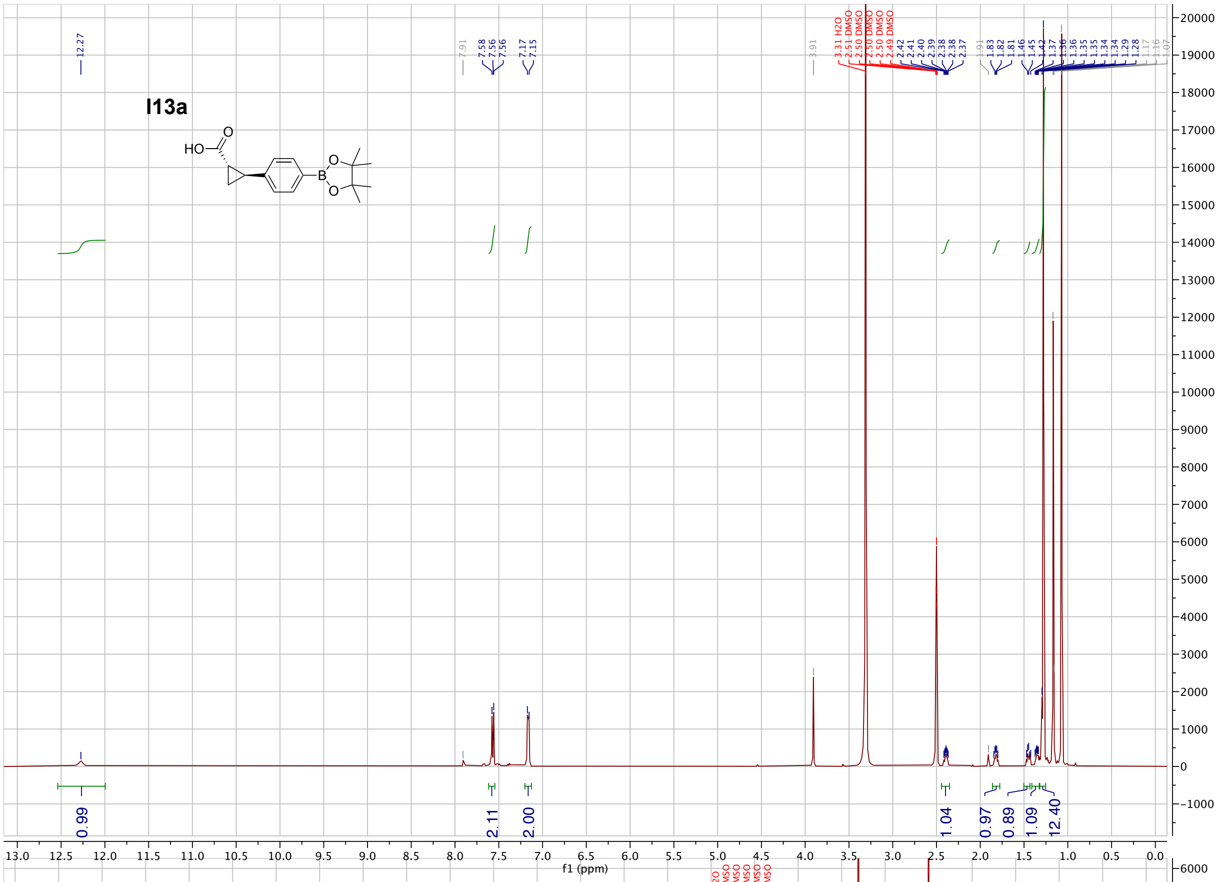

I13b

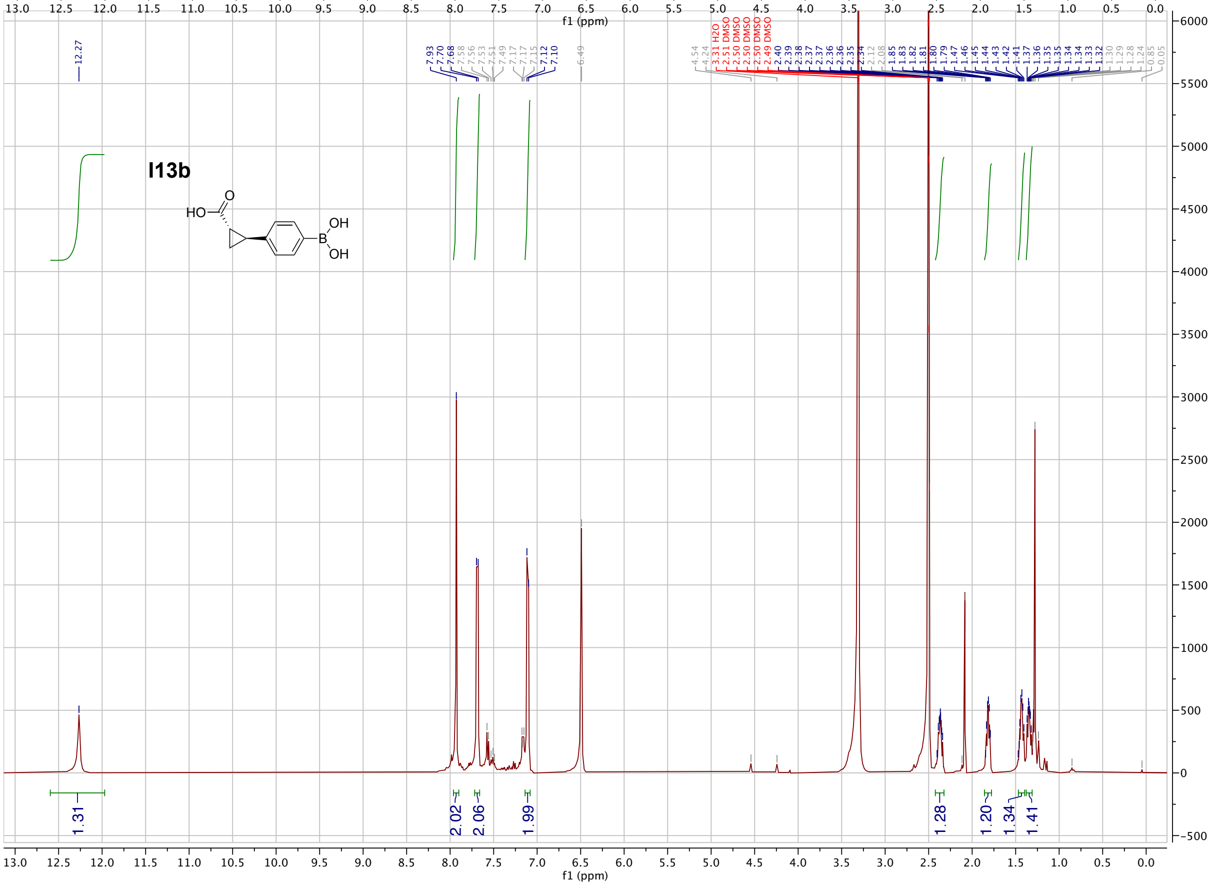

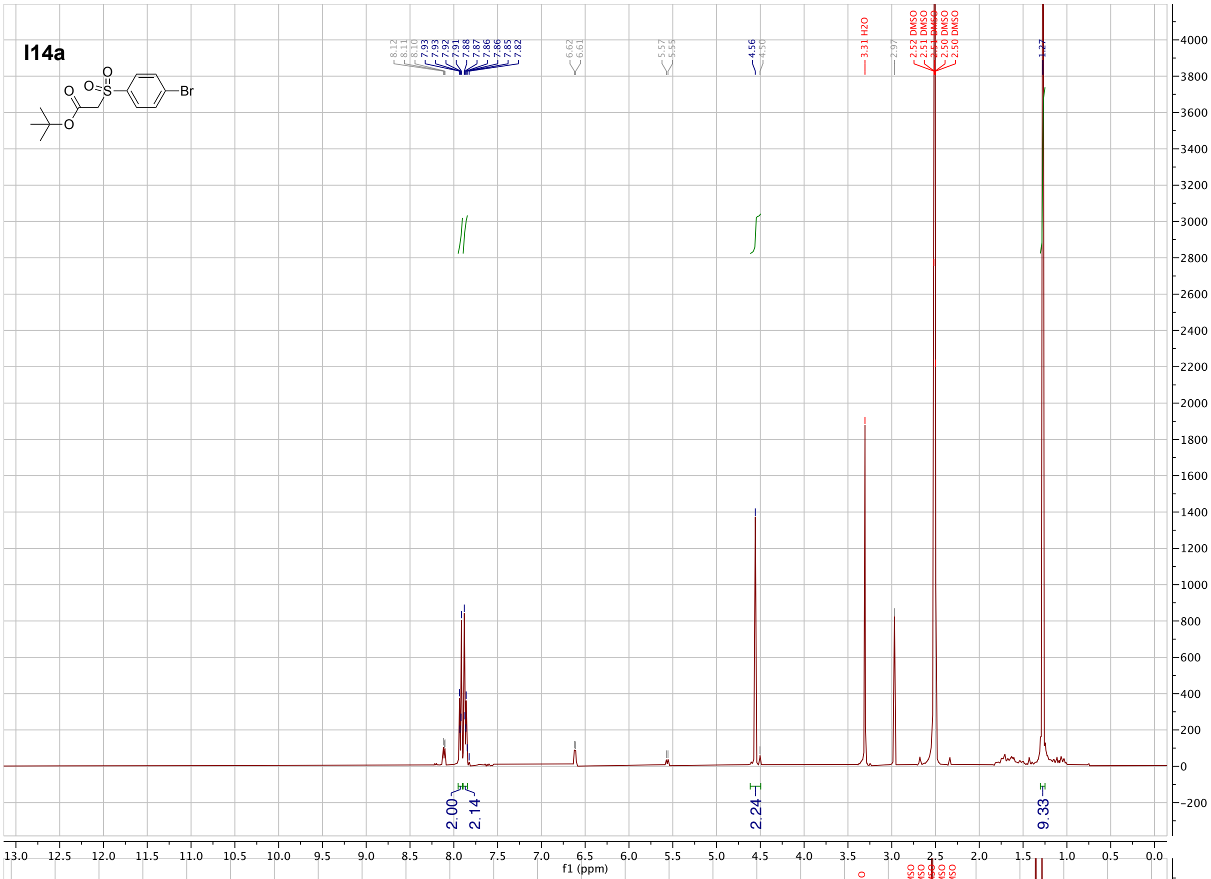
I14a
Br
2.00
2.14
2.24
9.33
3.31 H2O
f1 (ppm)

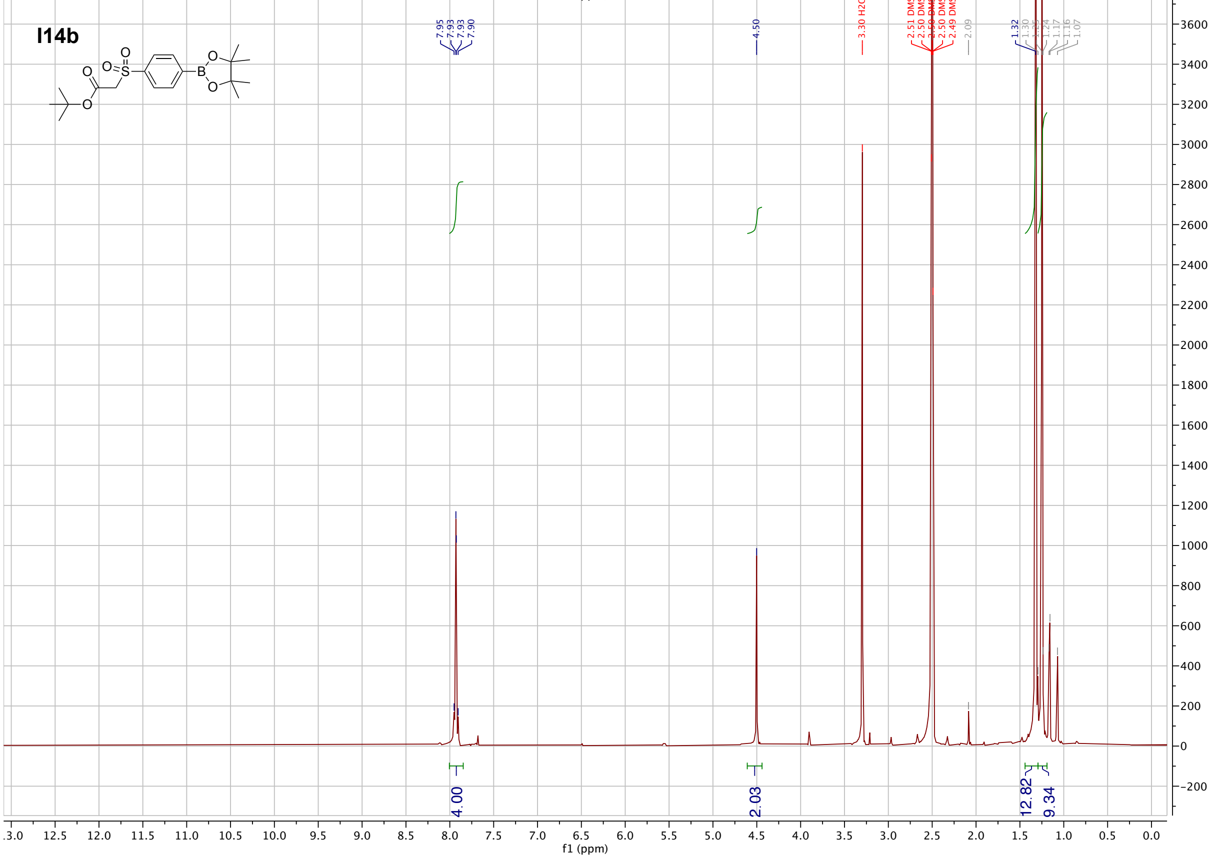
I14b
B
4.00
2.03
12.82
9.34
3.30 H2O
f1 (ppm)

I14c

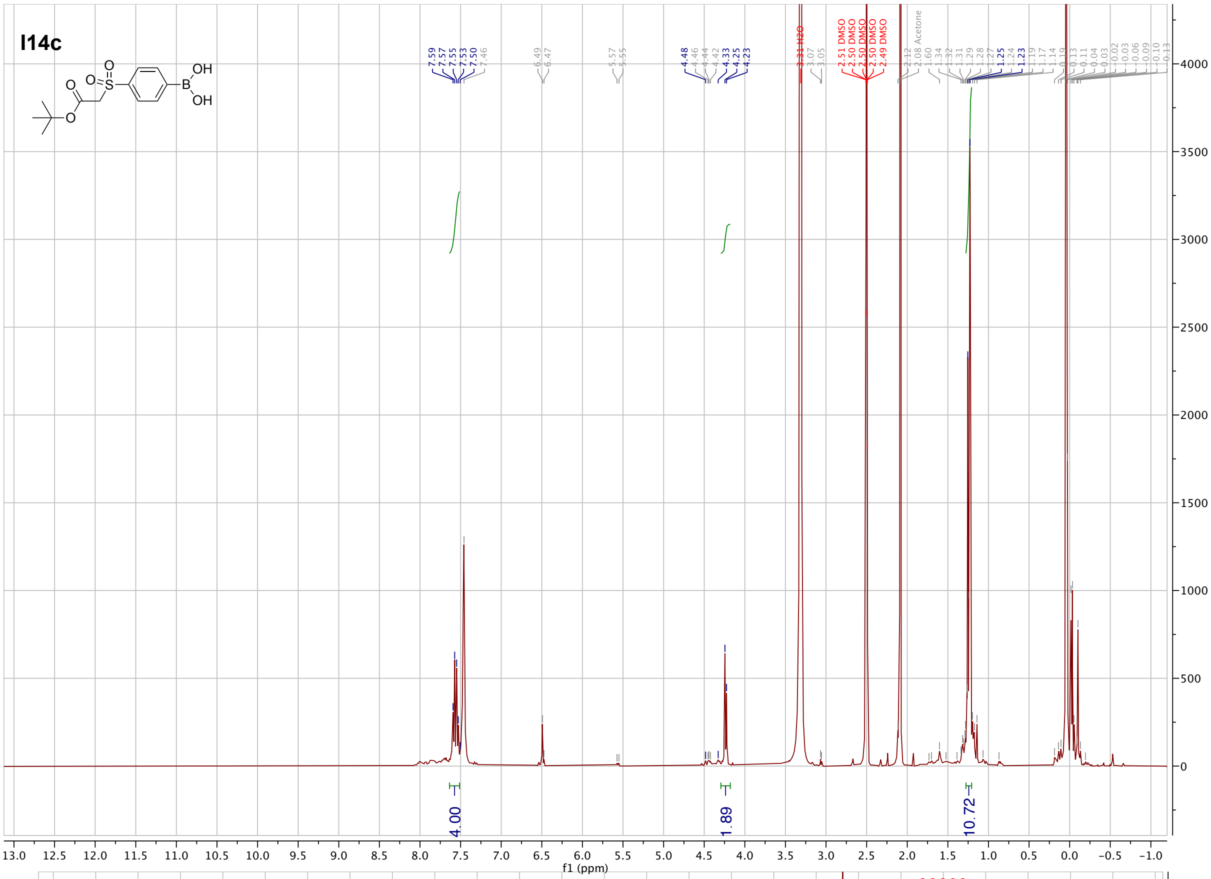

I15a

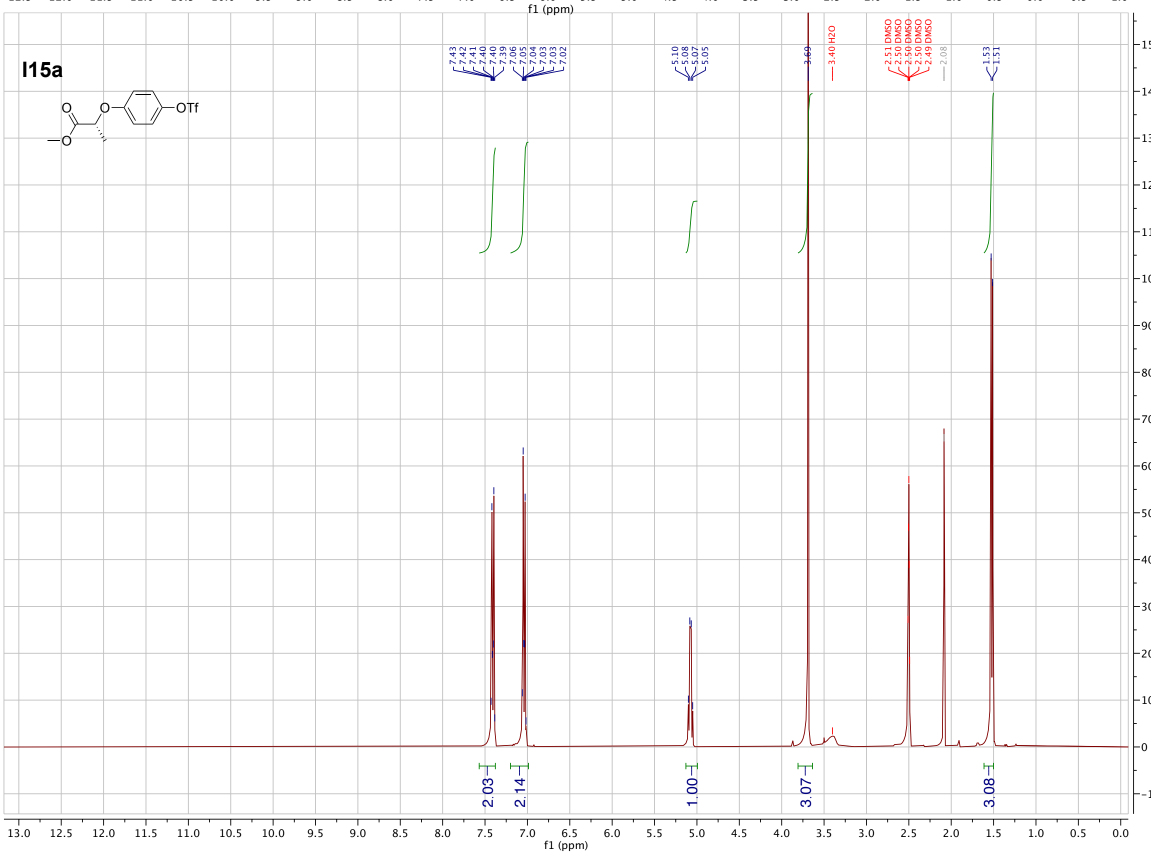

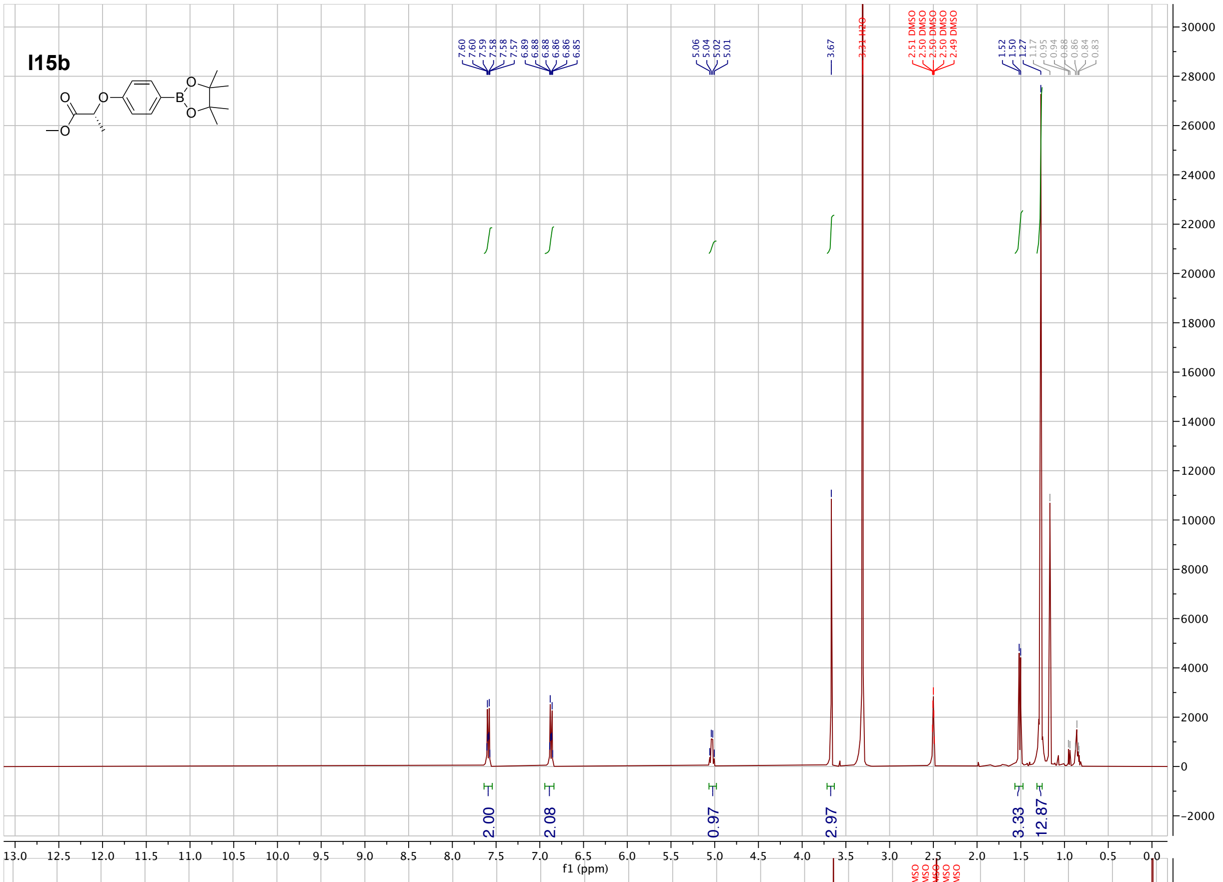
I15b
f1 (ppm)
2.00
2.08
0.97
2.97
3.33
12.87
3.31 H2O
2.51 DMSO
2.50 DMSO
2.49 DMSO

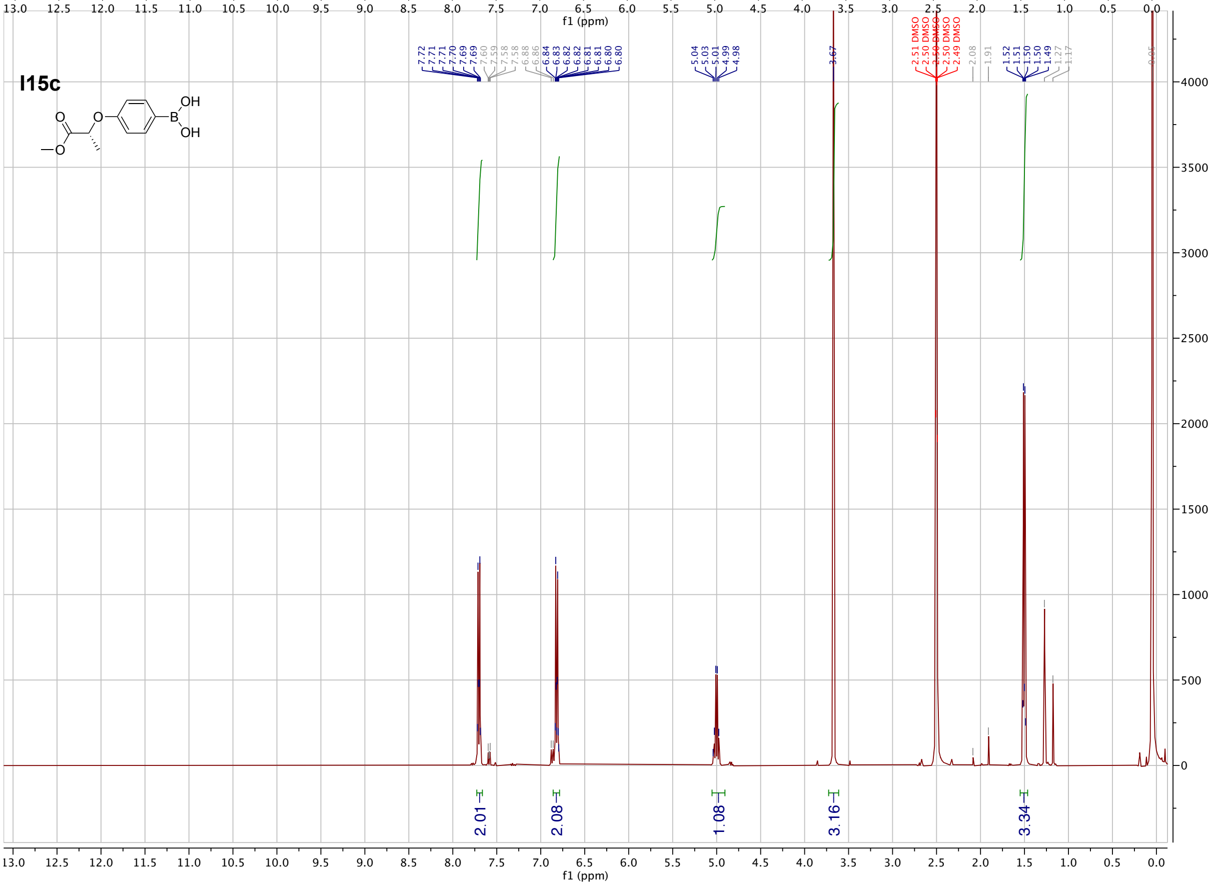
I15c
f1 (ppm)
2.01
2.08
1.08
3.16
3.34
2.51 DMSO
2.50 DMSO
2.49 DMSO

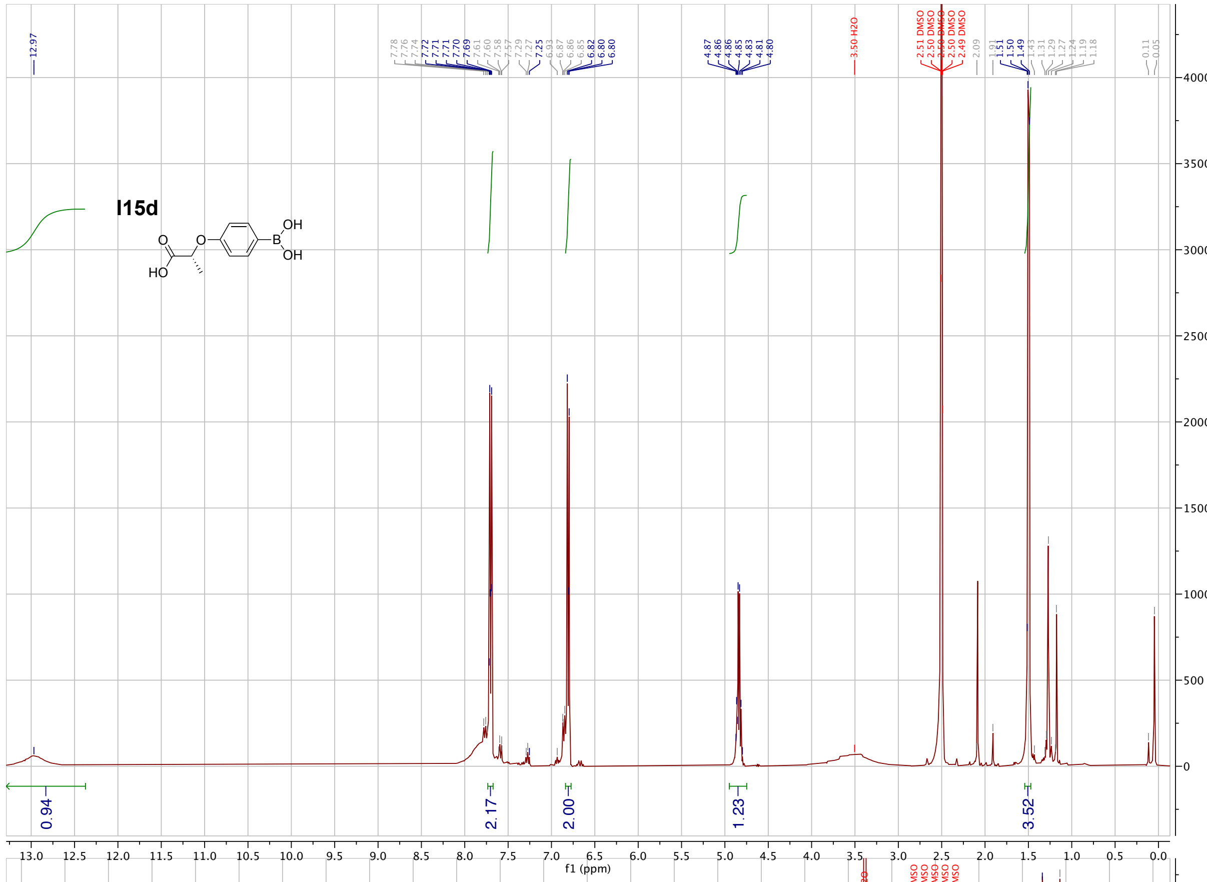
I15d
OH
OH
O
HO
O
B
13.0 12.5 12.0 11.5 11.0 10.5 10.0 9.5 9.0 8.5 8.0 7.5 7.0 6.5 6.0 5.5 5.0 4.5 4.0 3.5 3.0 2.5 2.0 1.5 1.0 0.5 0.0
f1 (ppm)
0.94
2.17
2.00
1.23
3.52
3.50 H2O
2.51 DMSO
2.50 DMSO
2.49 DMSO

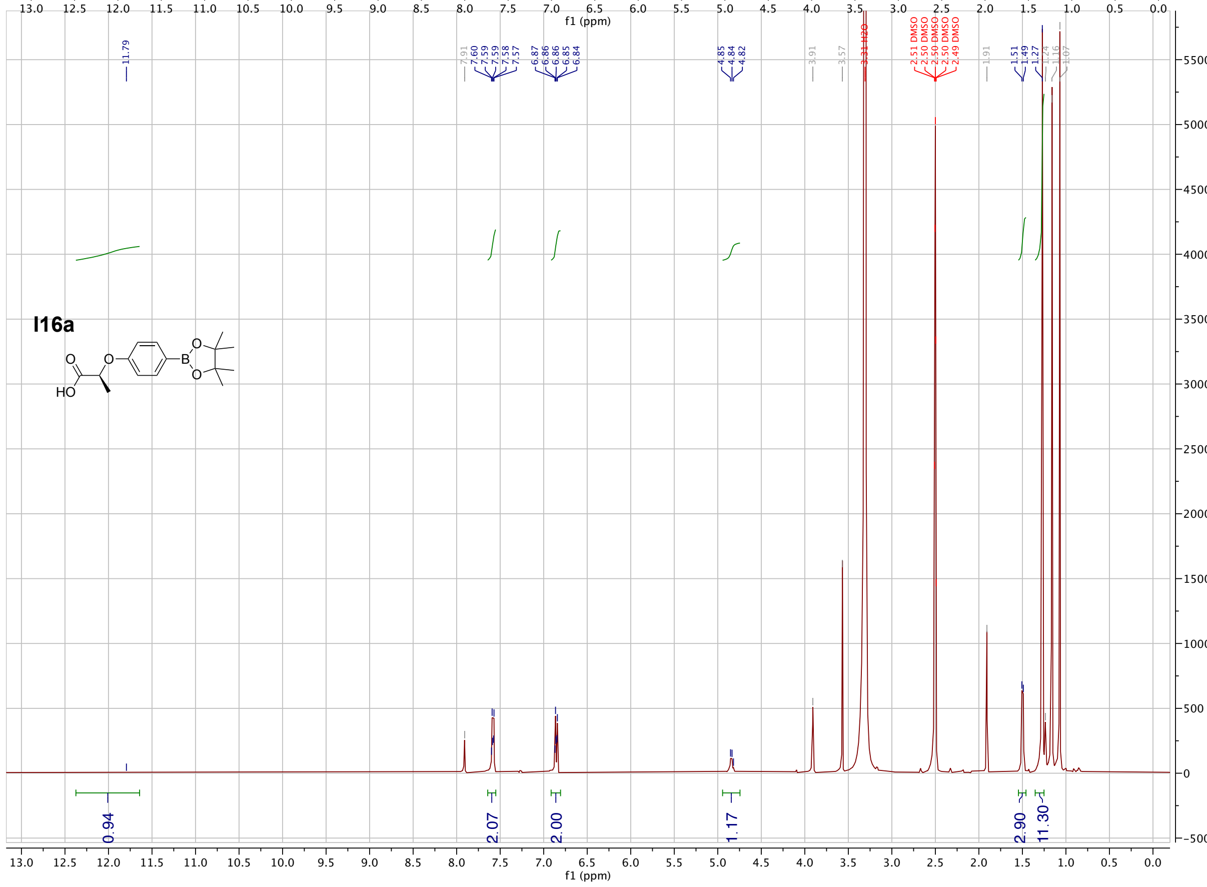
I16a
O
HO
O
B
O
O
13.0 12.5 12.0 11.5 11.0 10.5 10.0 9.5 9.0 8.5 8.0 7.5 7.0 6.5 6.0 5.5 5.0 4.5 4.0 3.5 3.0 2.5 2.0 1.5 1.0 0.5 0.0
f1 (ppm)
0.94
2.07
2.00
1.17
2.90
11.30
11.79
3.31 H2O
2.51 DMSO
2.50 DMSO
2.49 DMSO

I16b

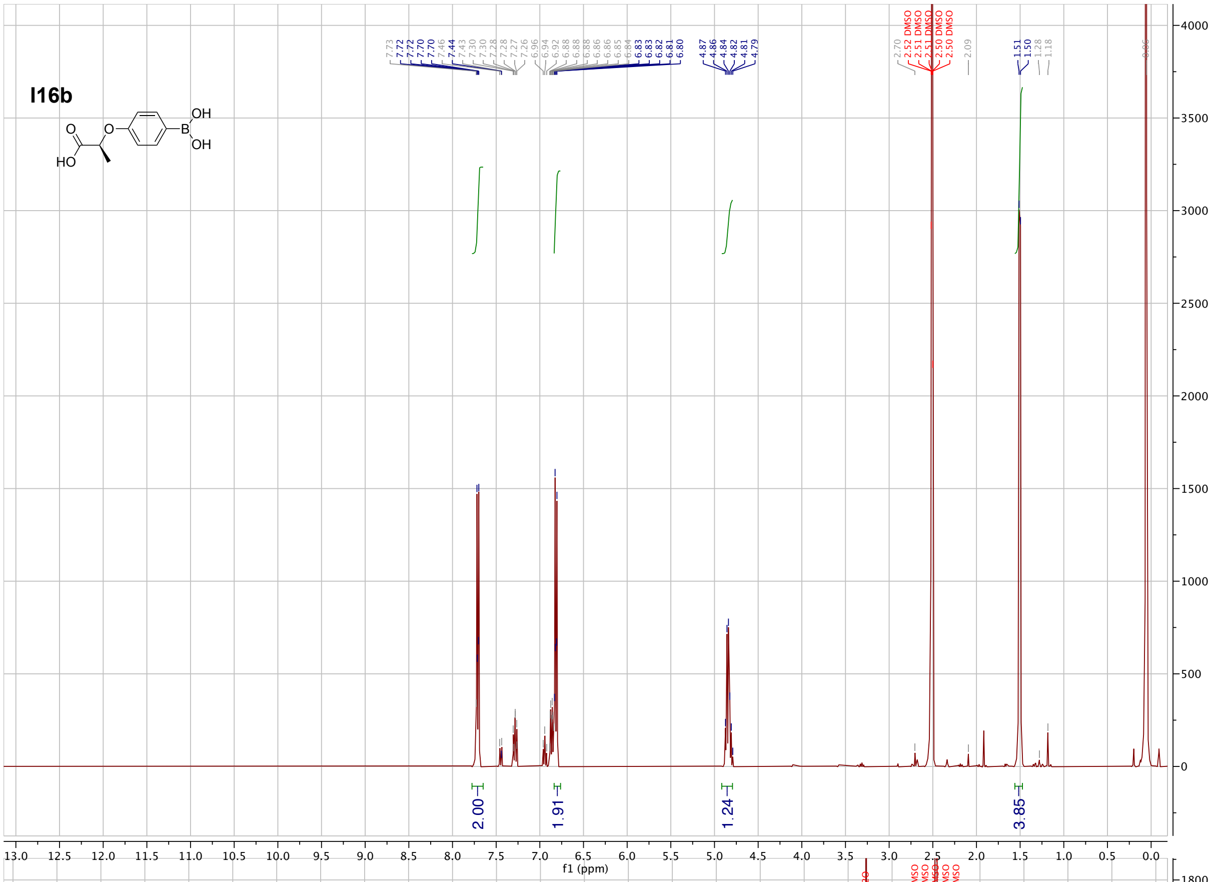

I17a

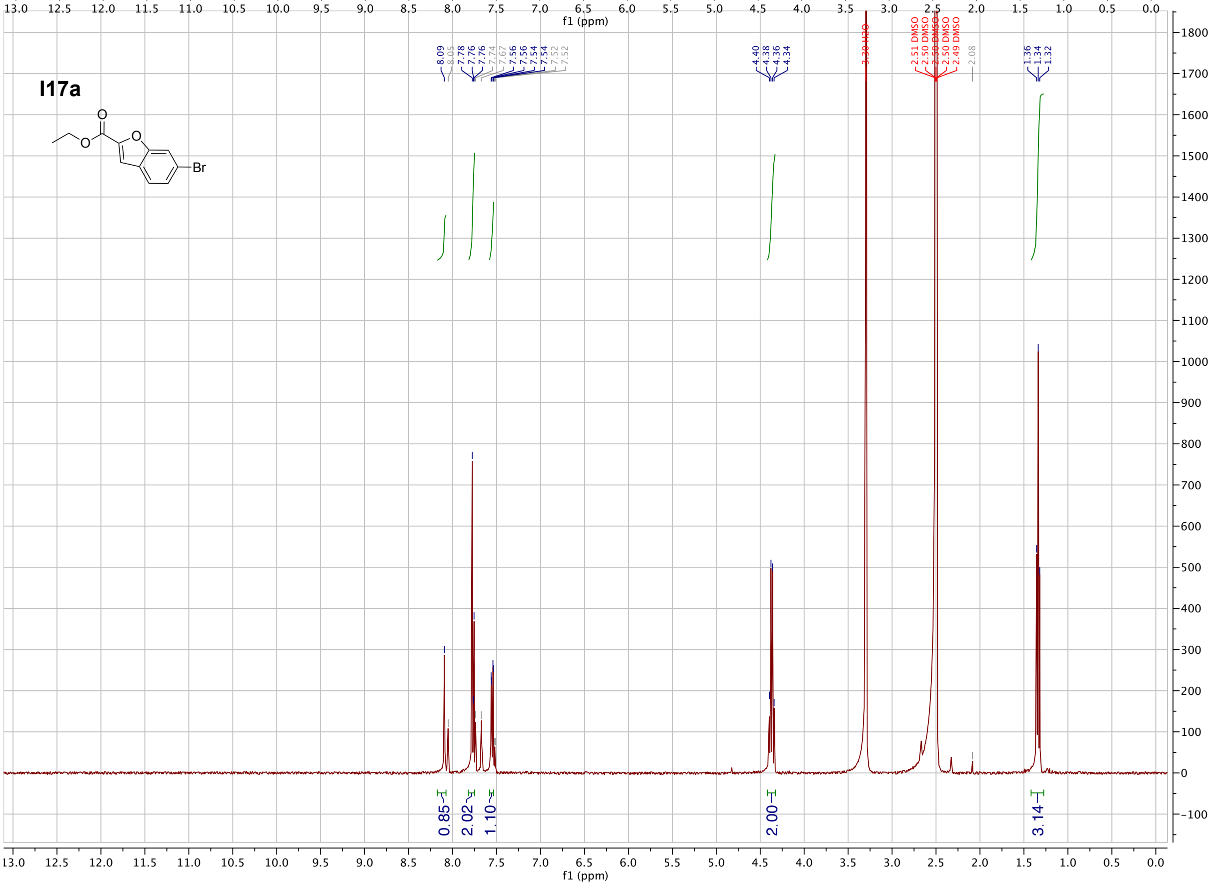

I17b

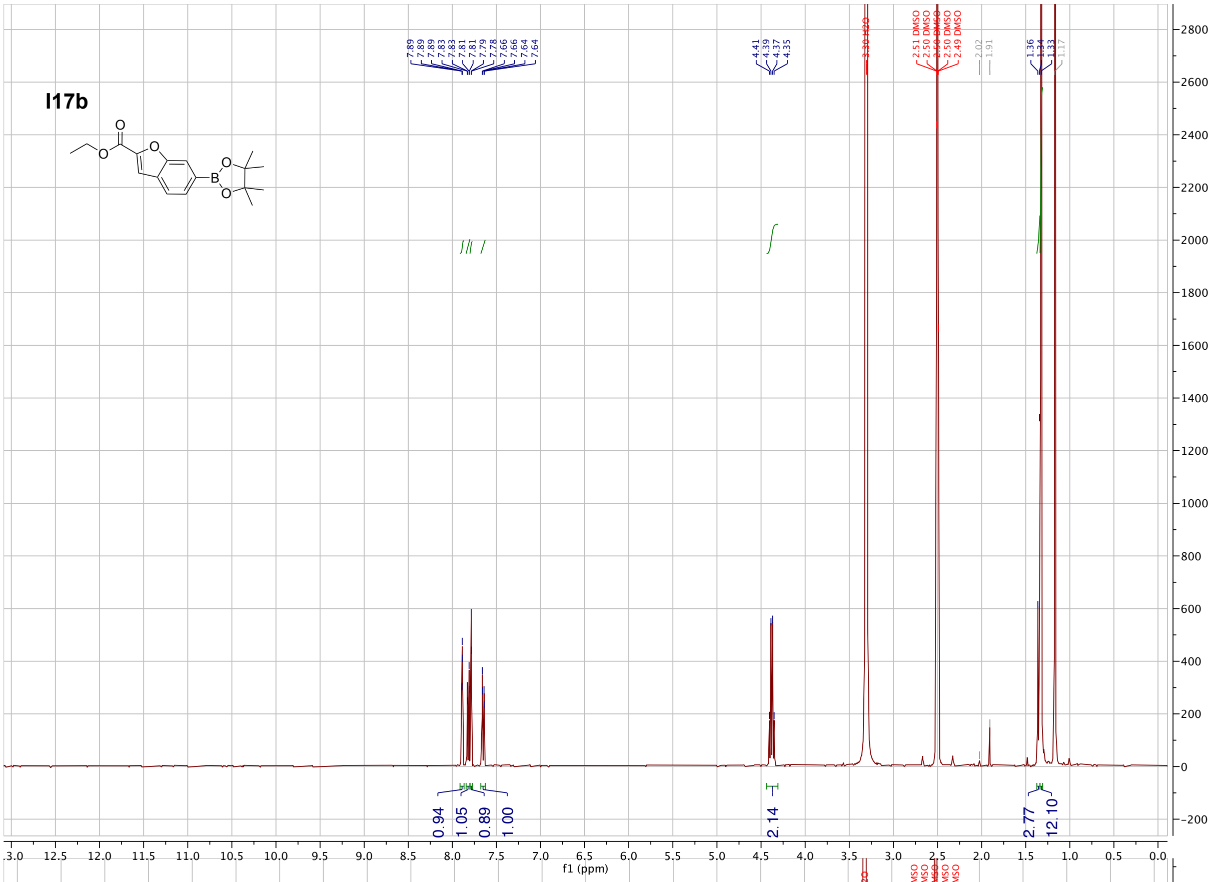

0.94
1.05
0.89
1.00
2.14
2.77
12.10
f1 (ppm)

I17c

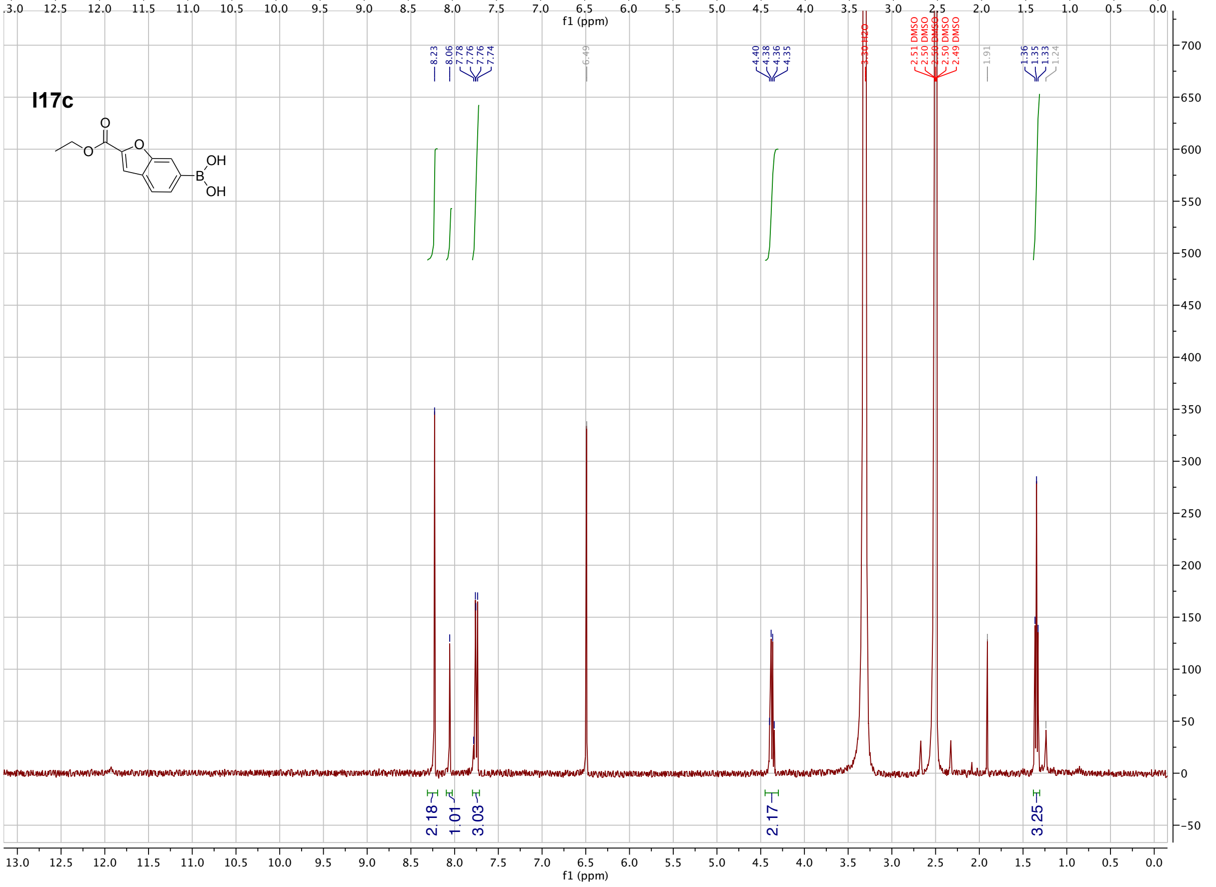

OH
B
OH
2.18
1.01
3.03
2.17
3.25
f1 (ppm)

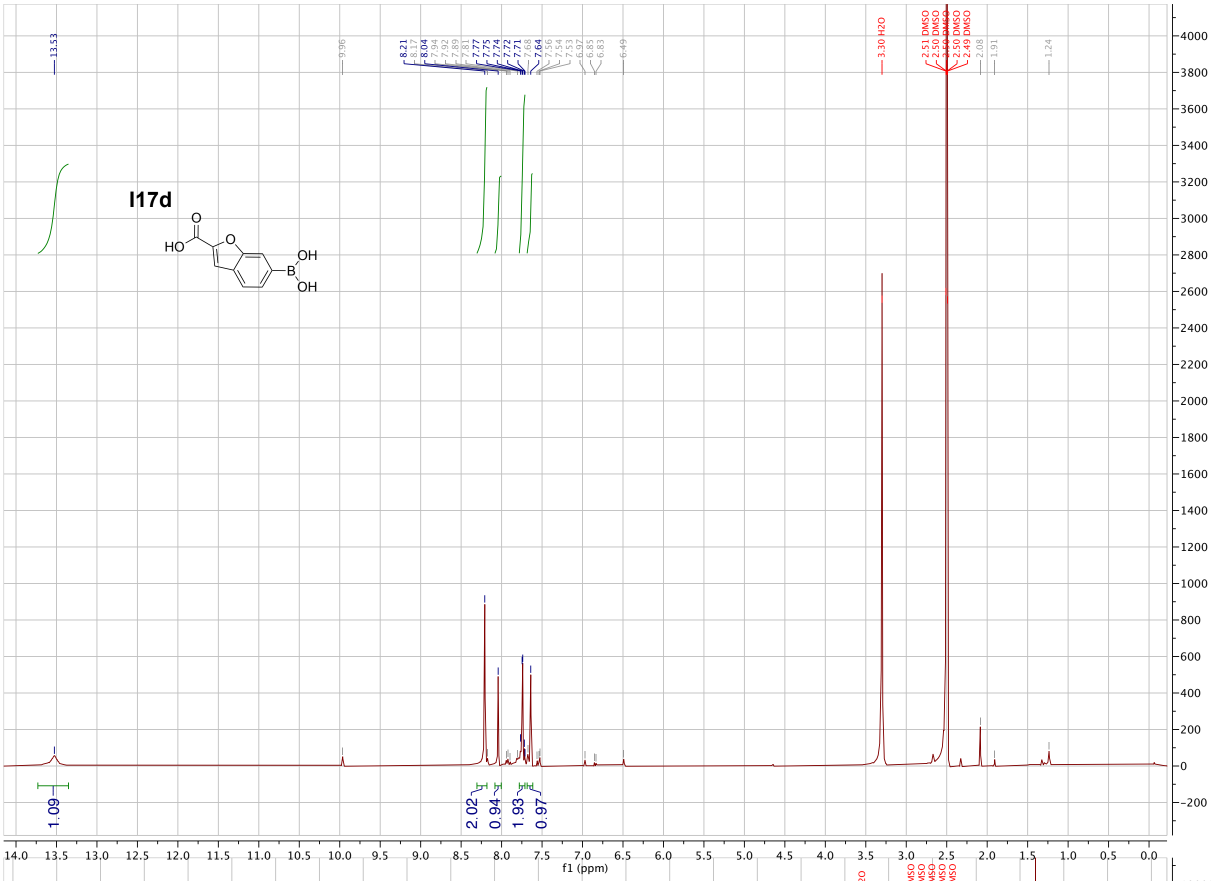
I17d
HO
O
O
B
OH
OH
13.53
9.96
8.21
8.17
8.04
7.94
7.92
7.89
7.81
7.77
7.75
7.74
7.72
7.71
7.68
7.64
7.56
7.54
7.53
6.97
6.85
6.83
6.49
3.30 H2O
2.51 DMSO
2.50 DMSO
2.50 DMSO
2.50 DMSO
2.49 DMSO
2.08
1.91
1.24
1.09
2.02
0.94
1.93
0.97
f1 (ppm)

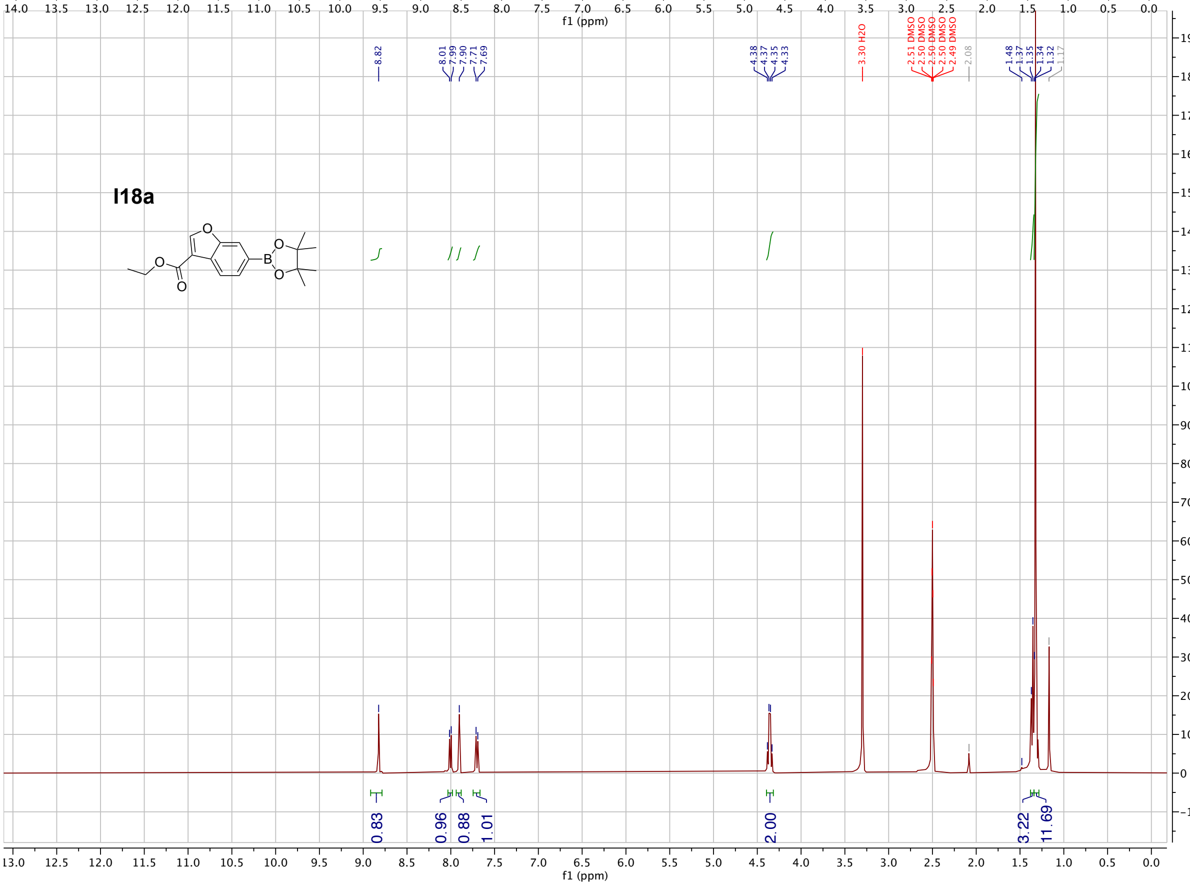
I18a
O
O
O
B
O
O
8.82
8.01
7.99
7.90
7.71
7.69
4.38
4.37
4.35
4.33
3.30 H2O
2.51 DMSO
2.50 DMSO
2.50 DMSO
2.50 DMSO
2.49 DMSO
2.08
1.48
1.37
1.35
1.34
1.32
1.17
0.83
0.96
0.88
1.01
2.00
3.22
11.69
f1 (ppm)

I18b

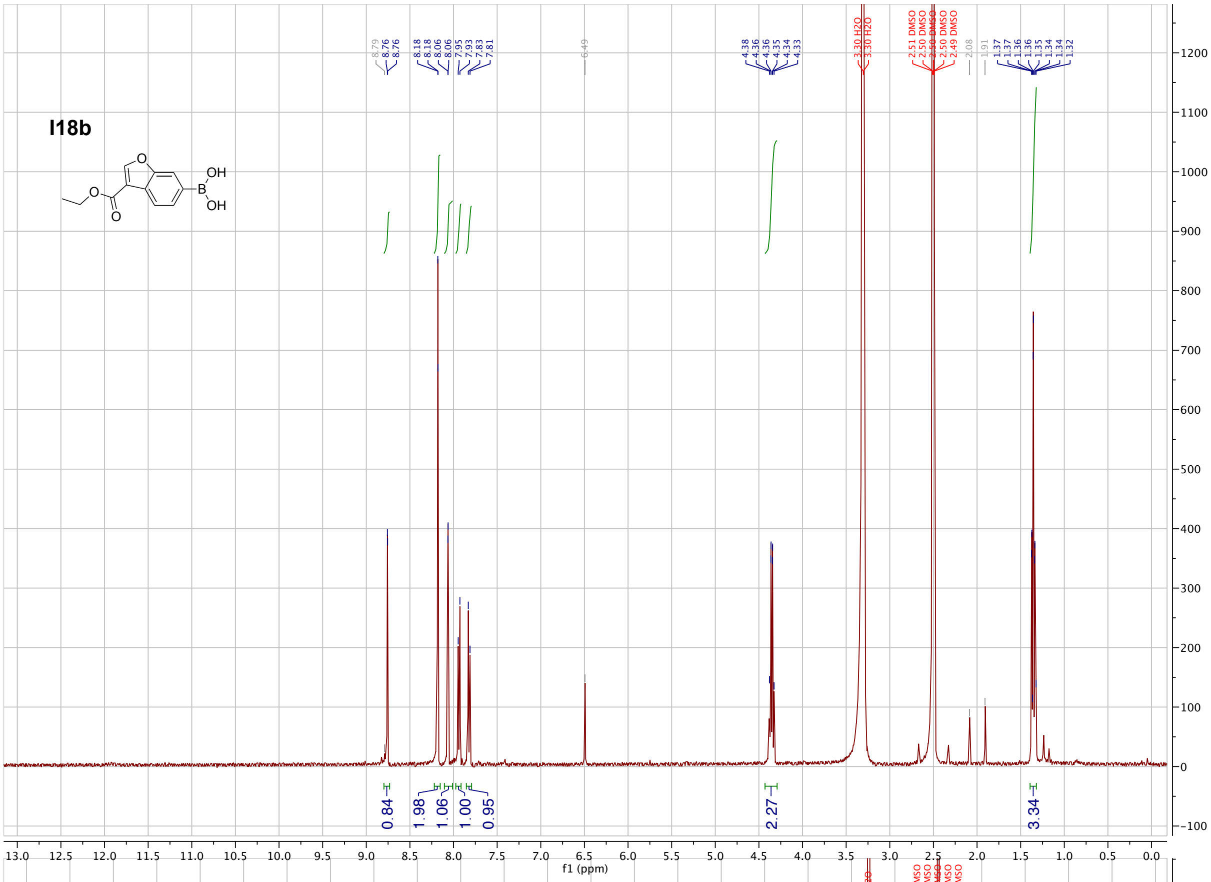

I18c

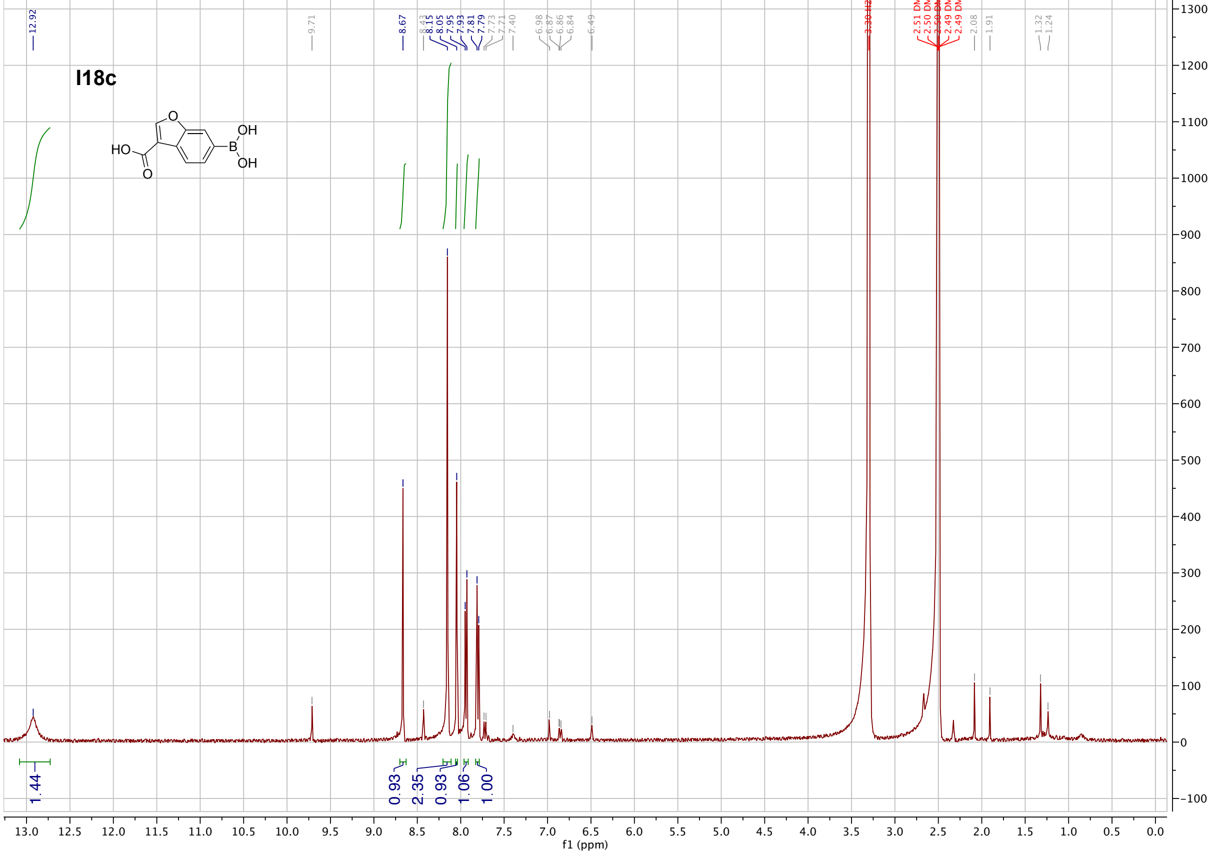

I19a

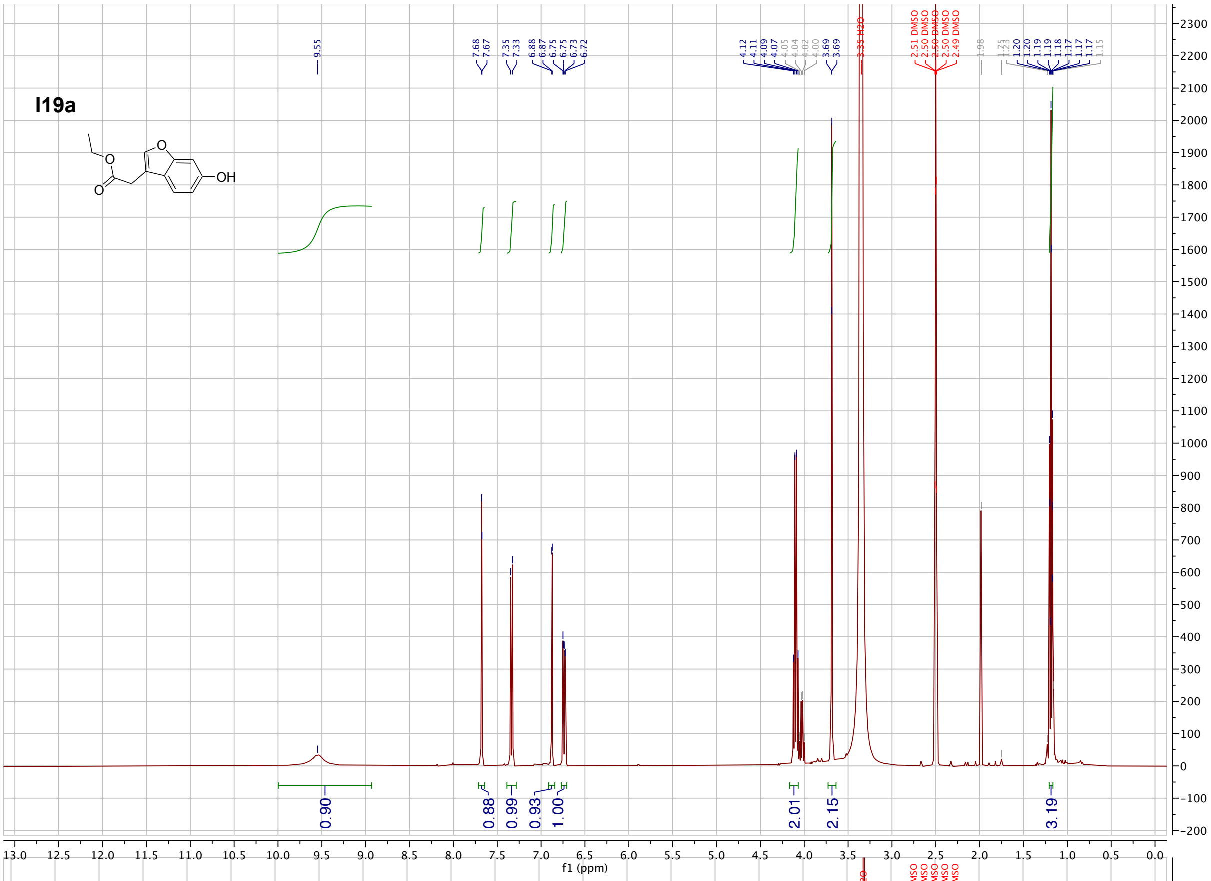

I19b

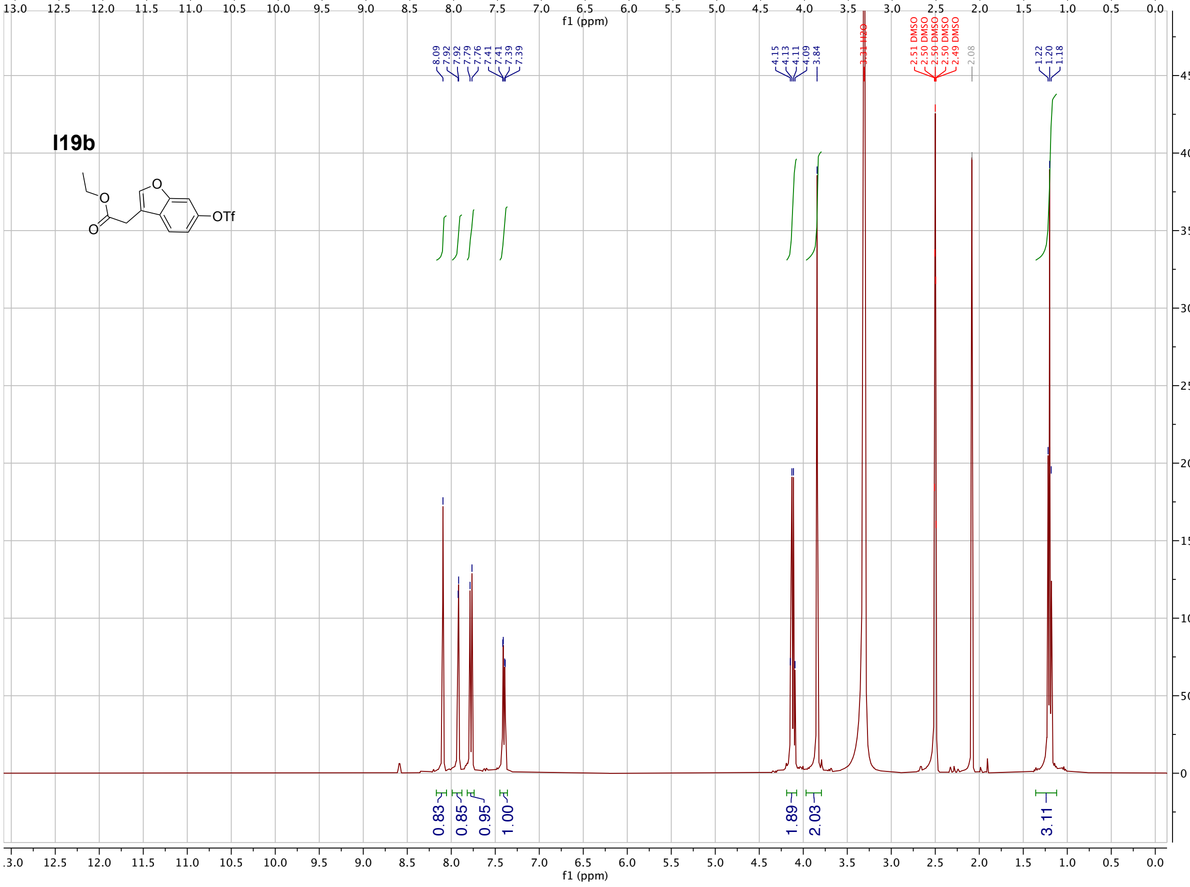

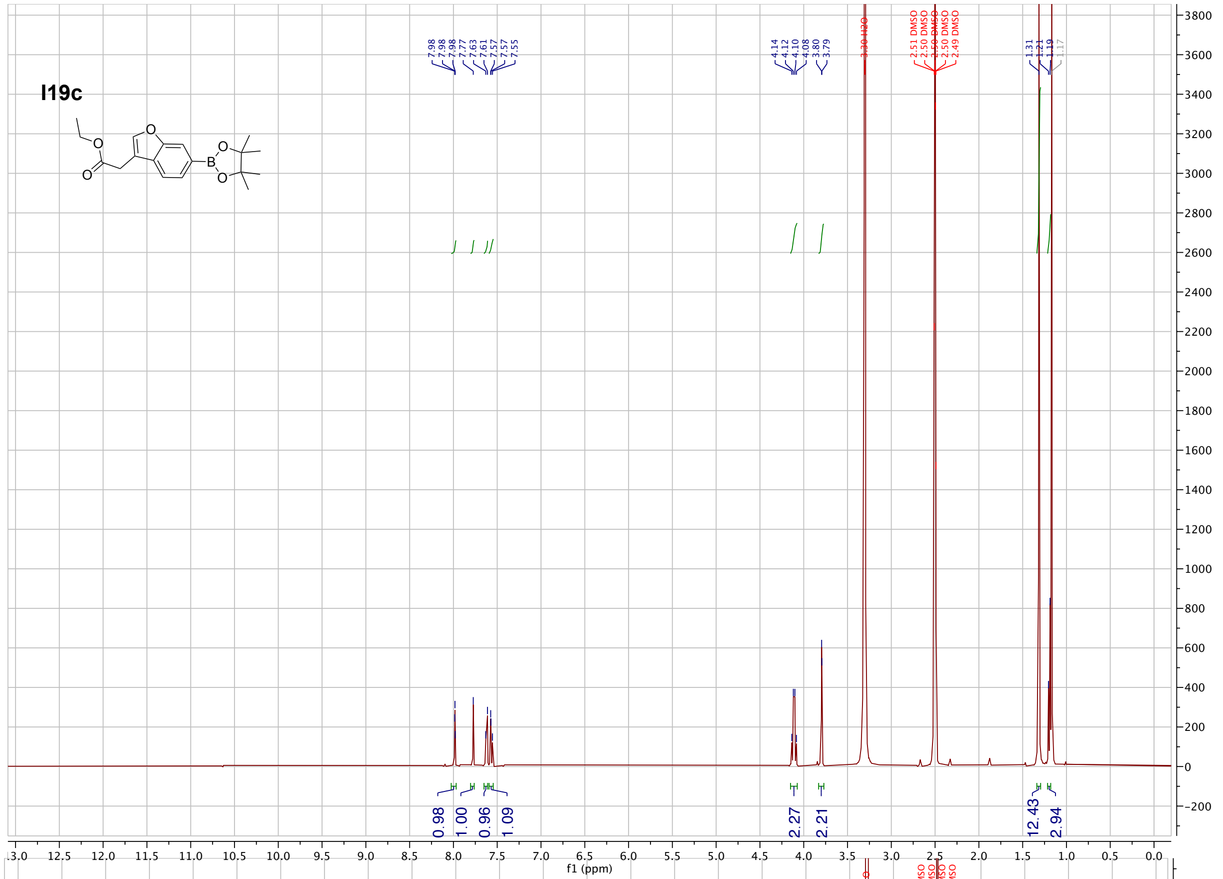
I19c
f1 (ppm)
3.30 H2O
2.51 DMSO
2.50 DMSO
2.49 DMSO
0.98
1.00
0.96
1.09
2.27
2.21
12.43
2.94

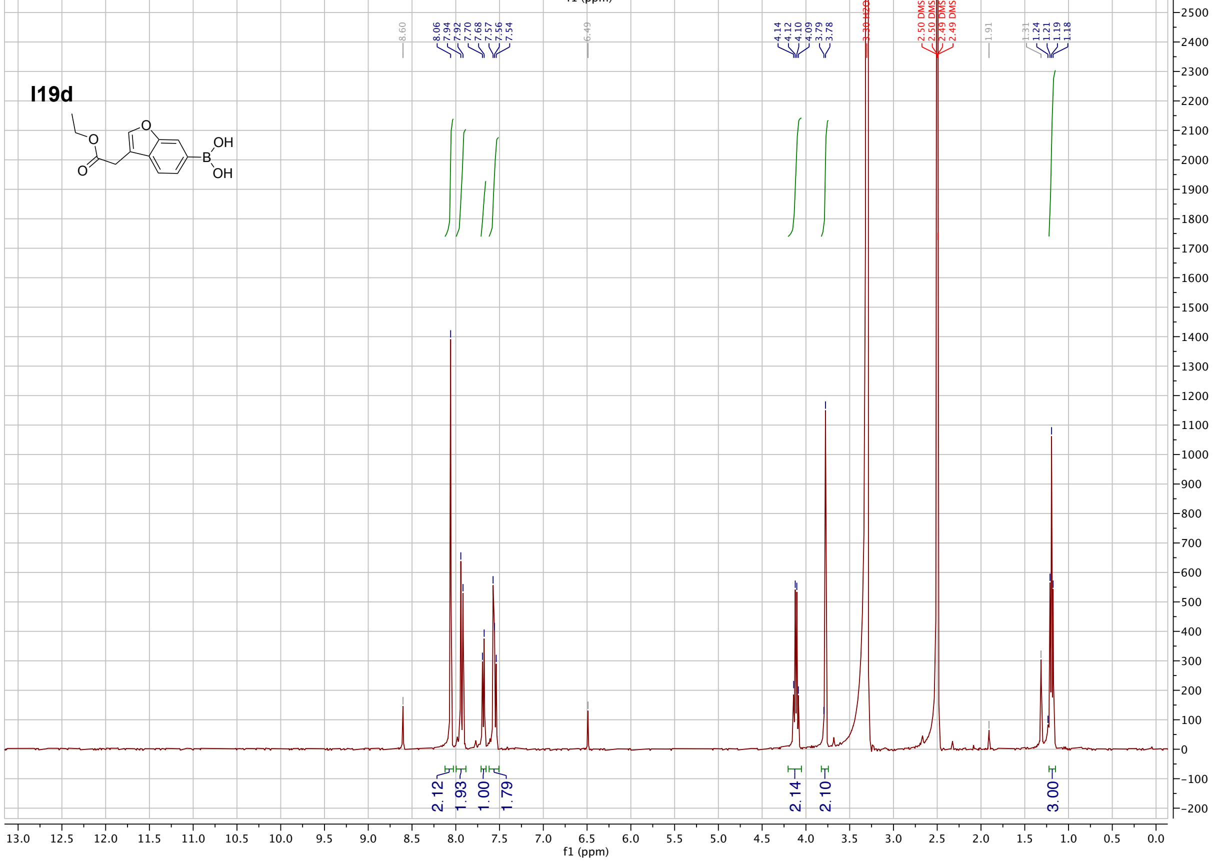
I19d
OH
OH
f1 (ppm)
3.30 H2O
2.50 DMSO
2.49 DMSO
2.12
1.93
1.00
1.79
2.14
2.10
3.00

I19e

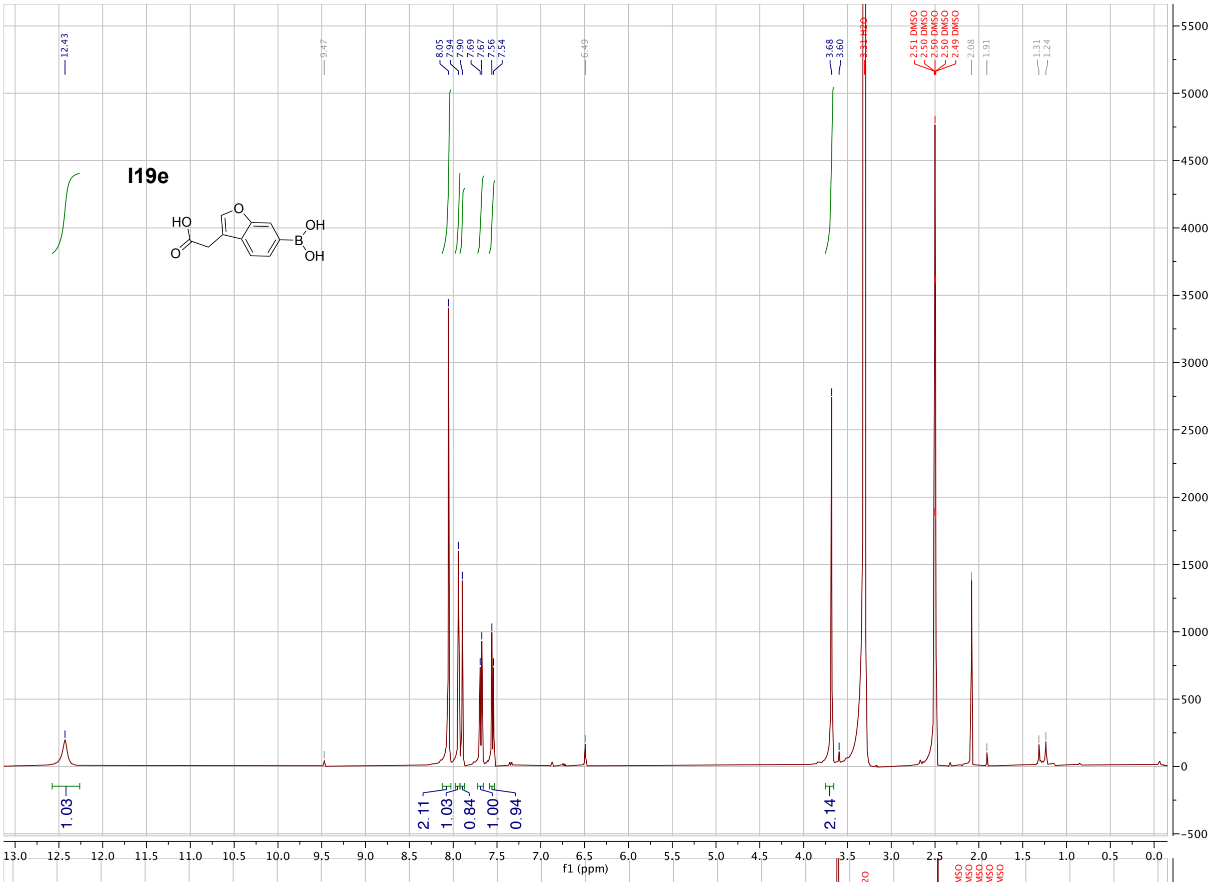

I20a

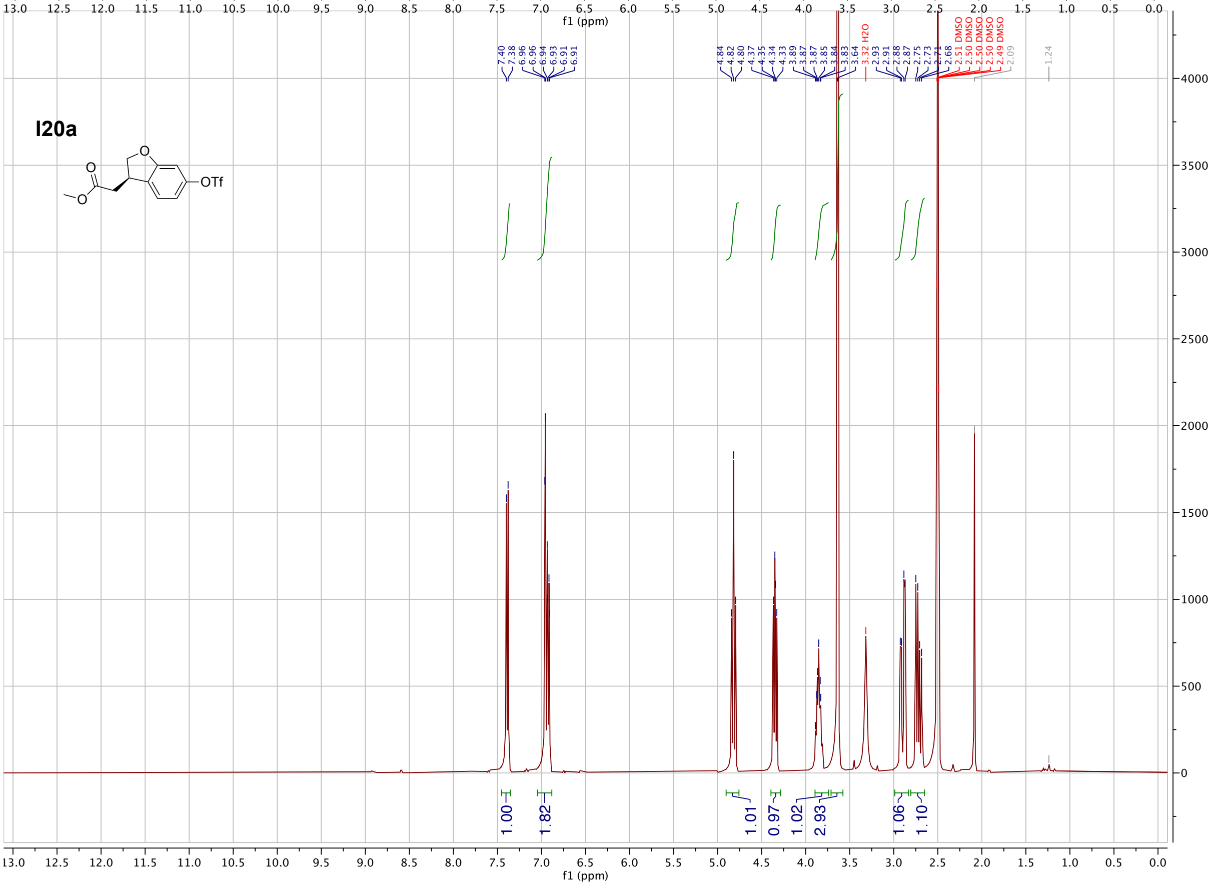

I20b

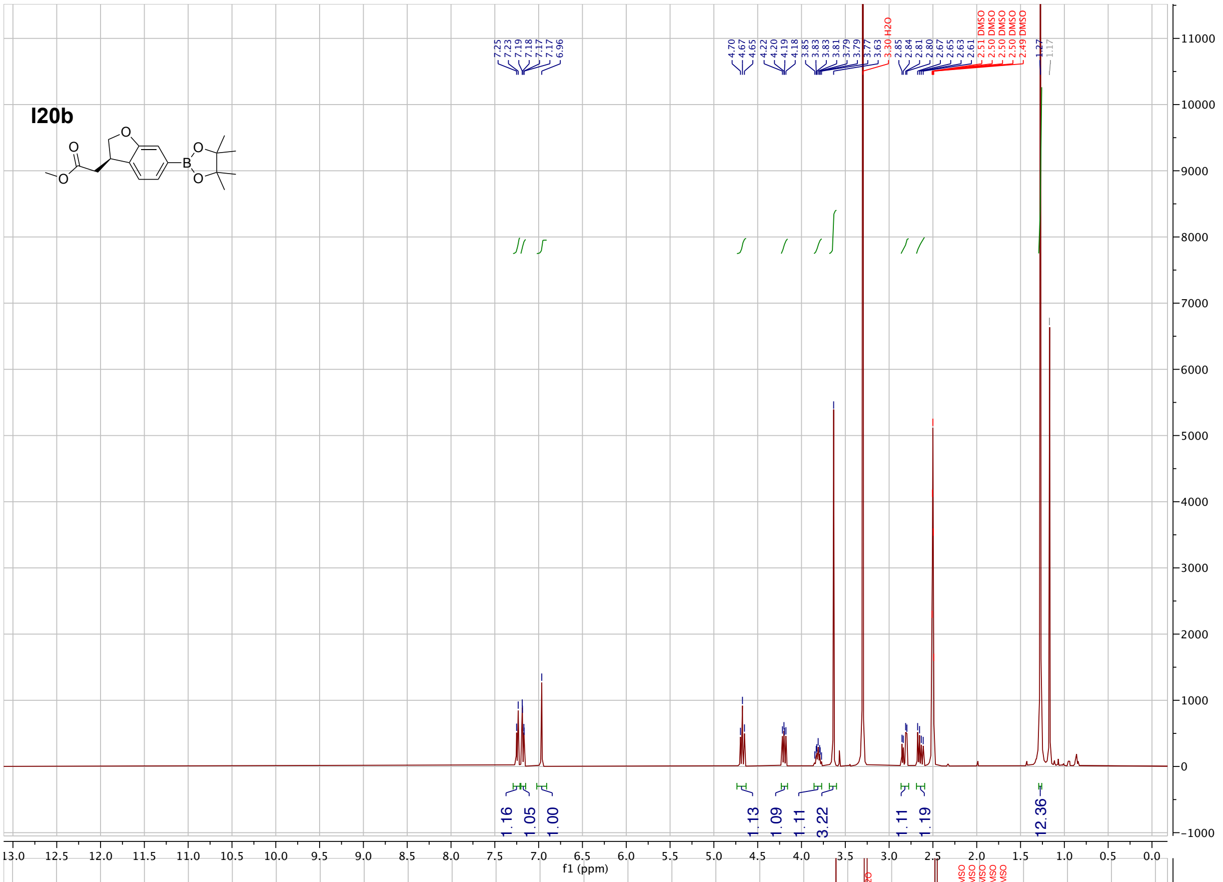
f1 (ppm)
1.16
1.05
1.00
1.13
1.09
1.11
3.22
1.11
1.19
12.36

I20c

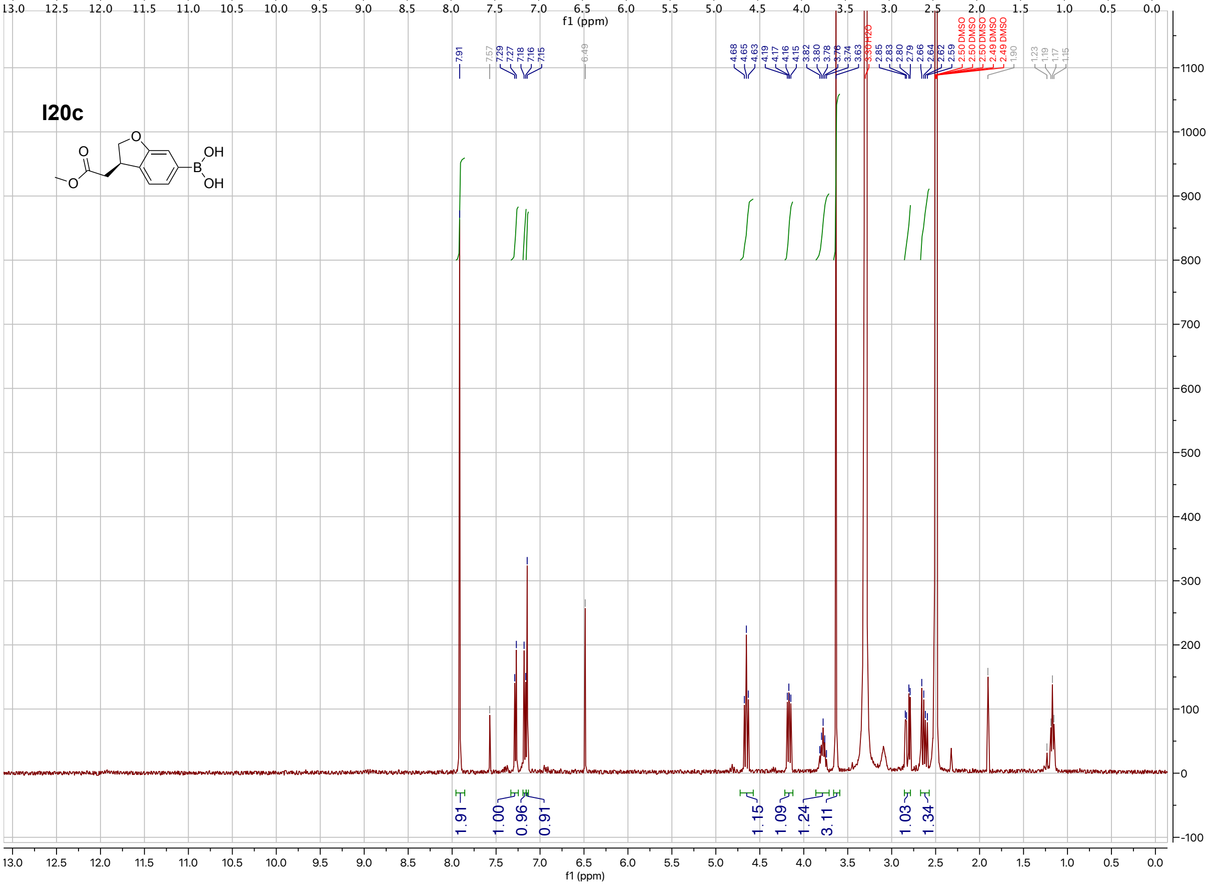
f1 (ppm)
1.91
1.00
0.96
0.91
1.15
1.09
1.24
3.11
1.03
1.34

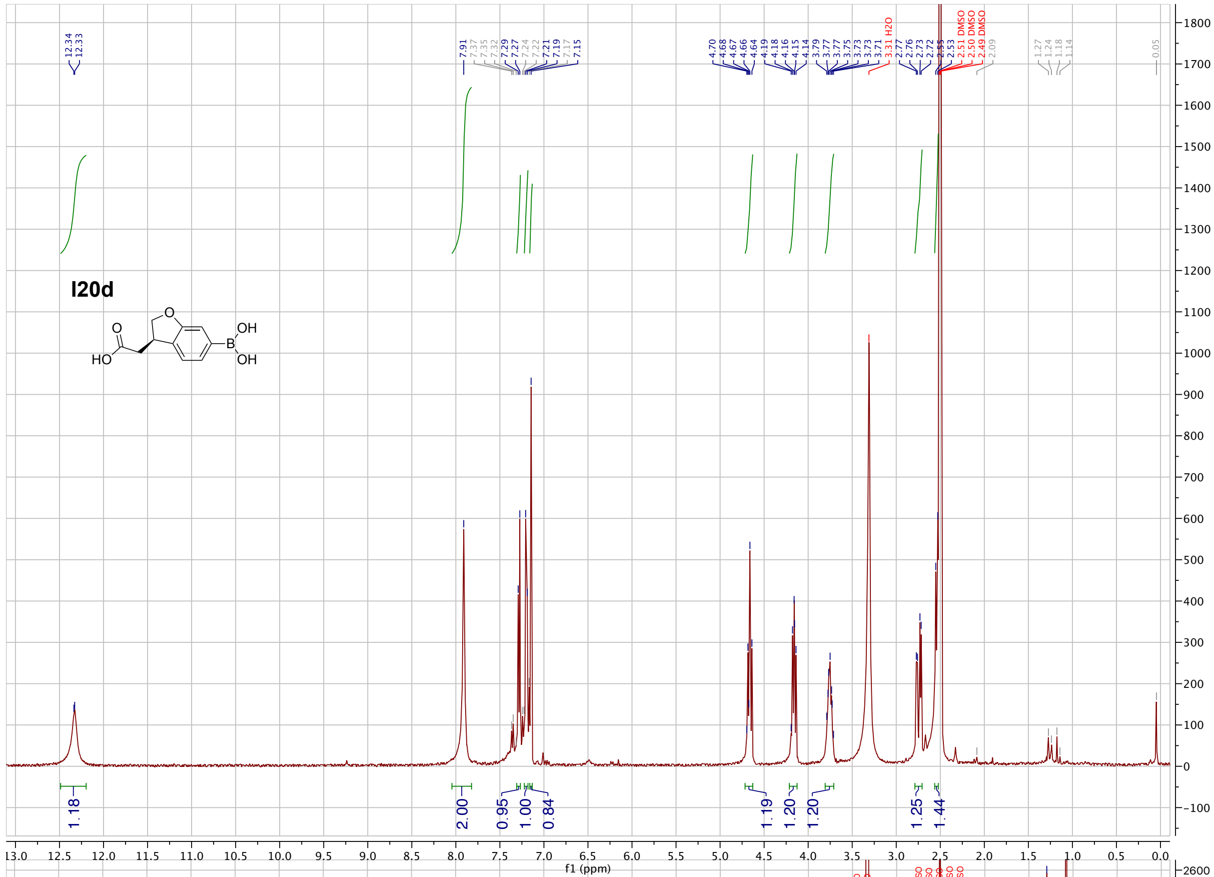
I20d
f1 (ppm)

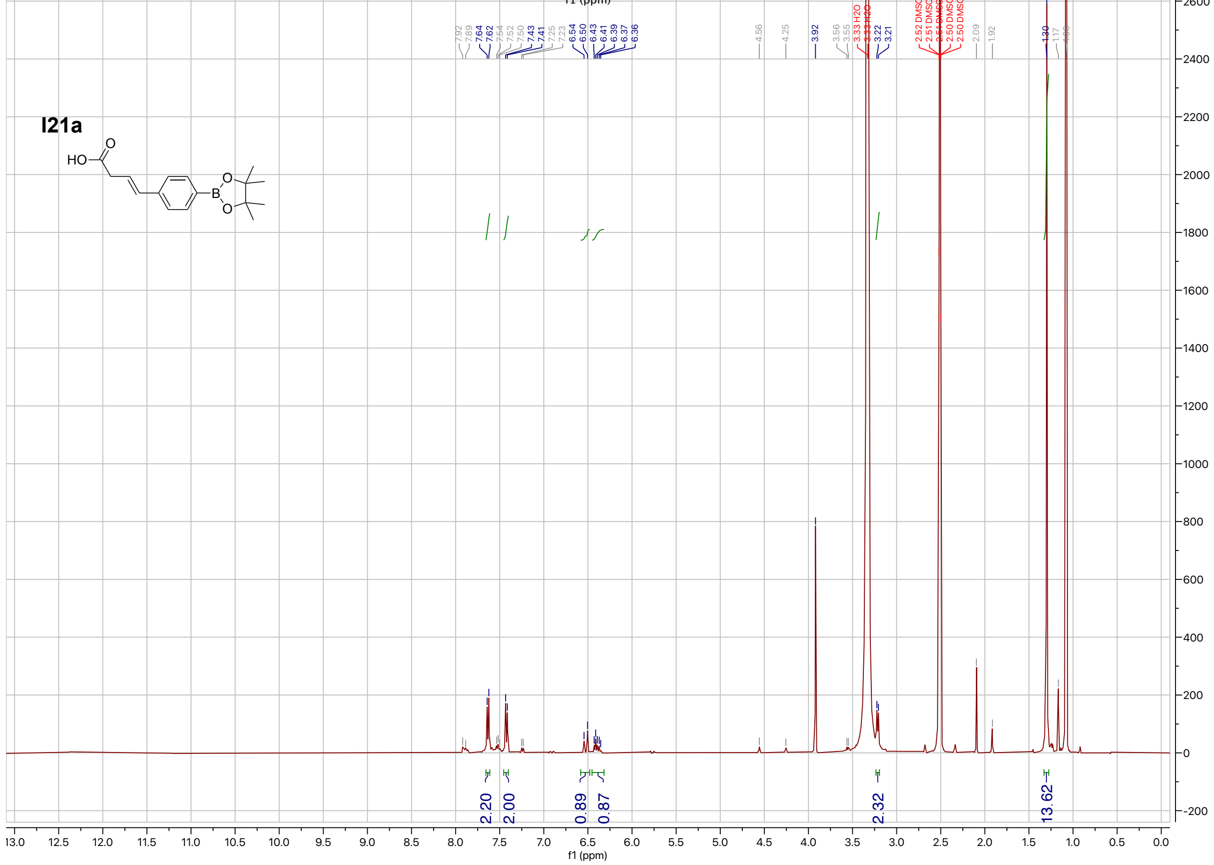
I21a
f1 (ppm)

I21b

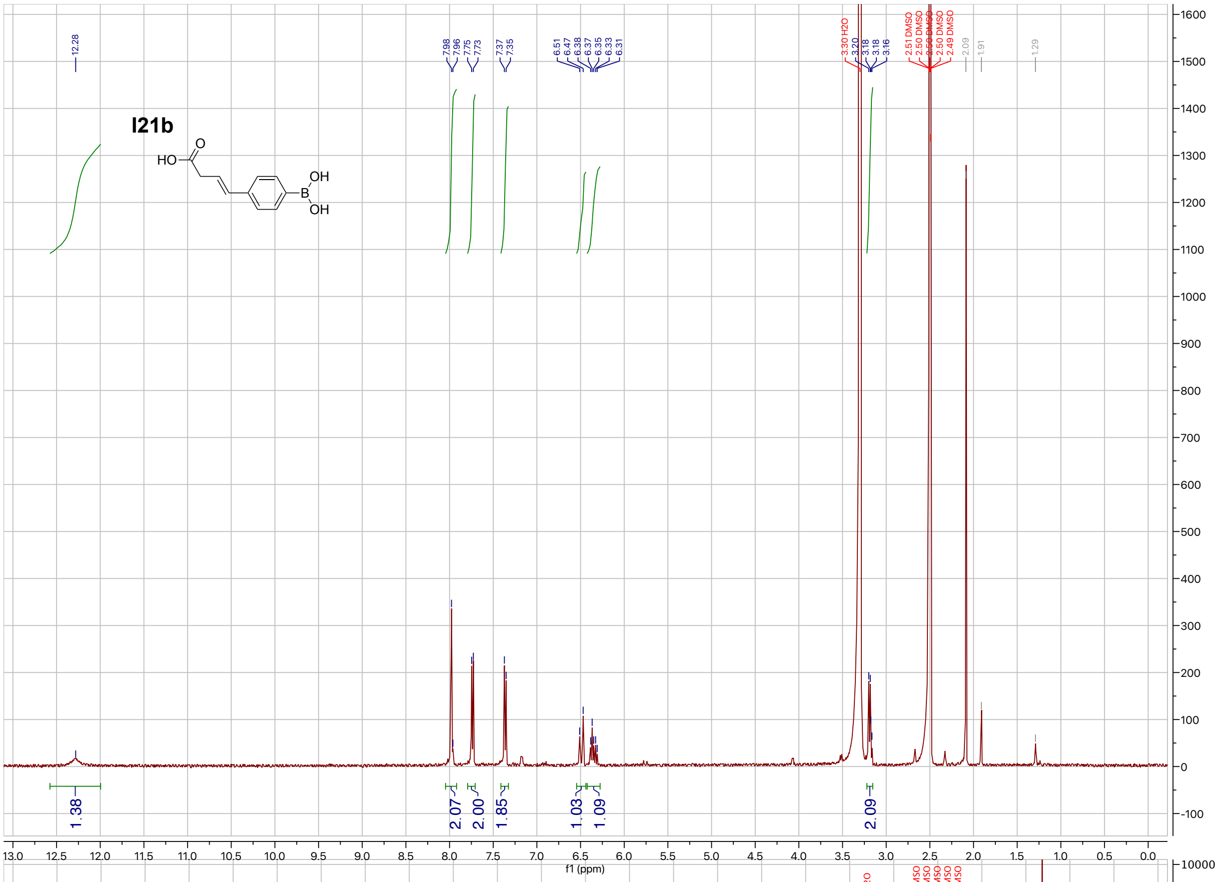

I22a

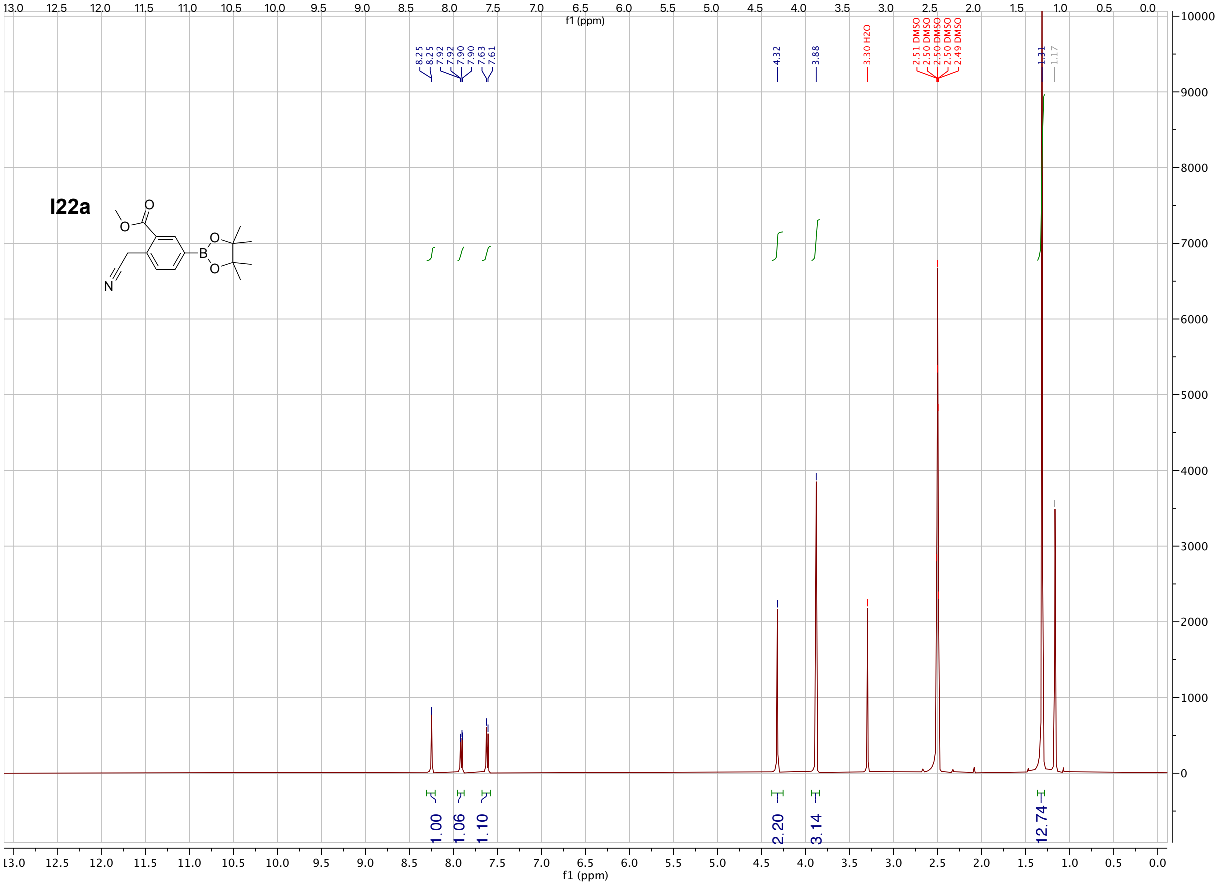

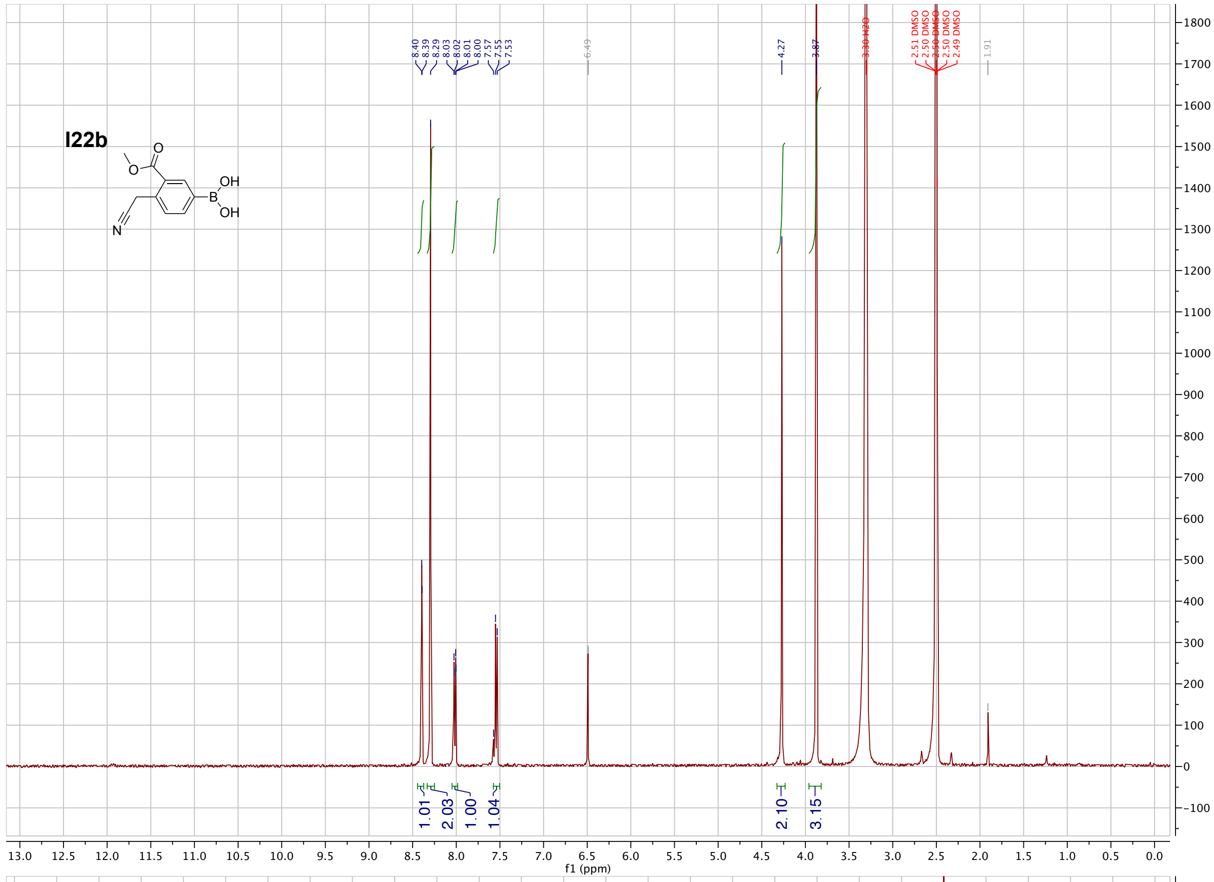
I22b
f1 (ppm)

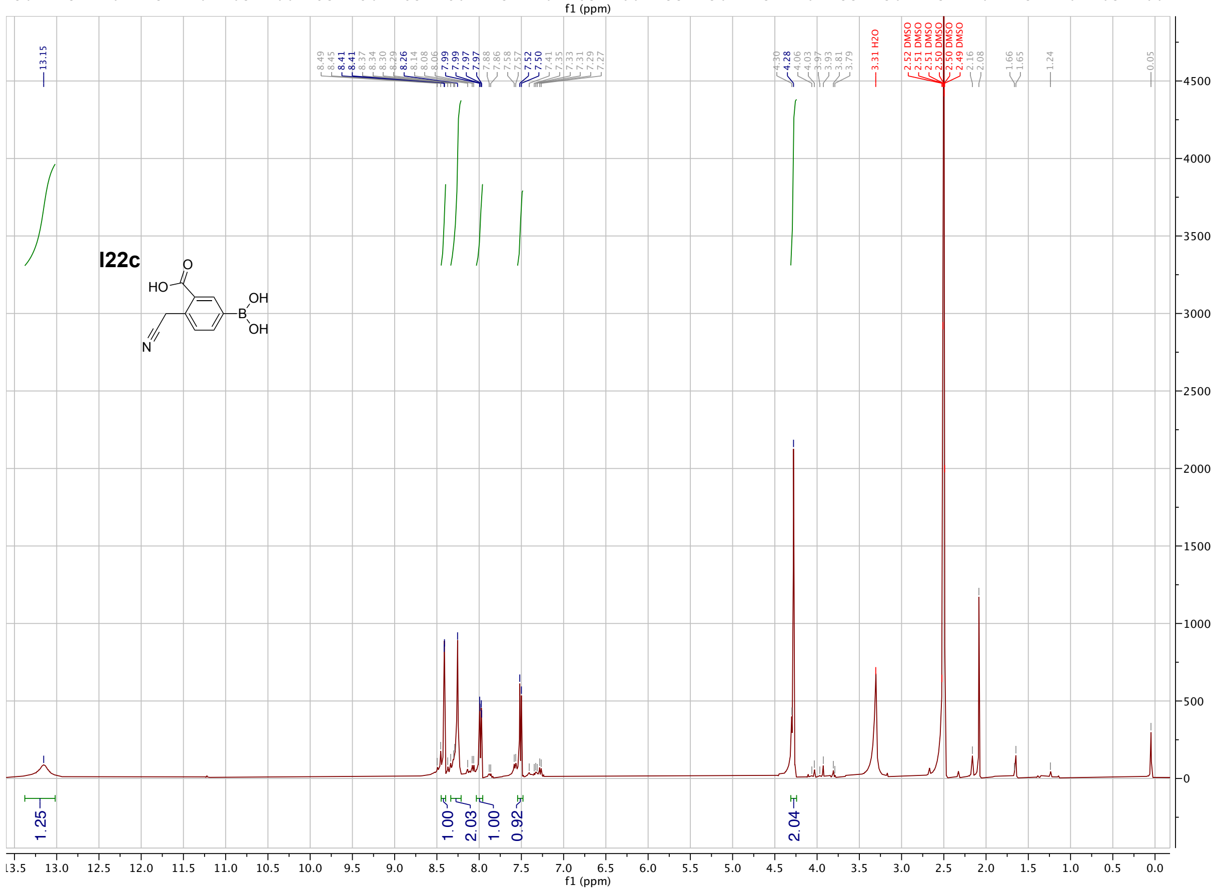
I22c
f1 (ppm)

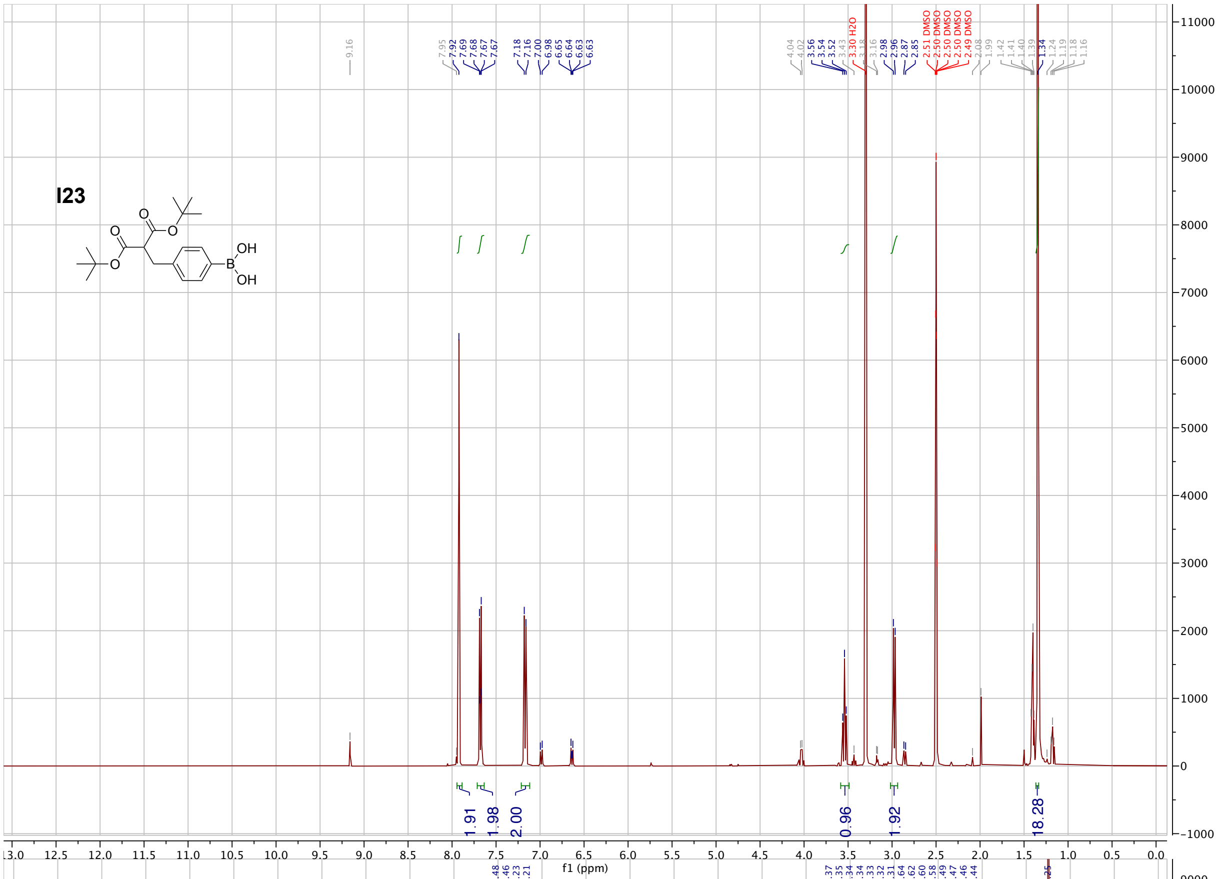
I23
OH
OH
B
O
O
O
O
f1 (ppm)

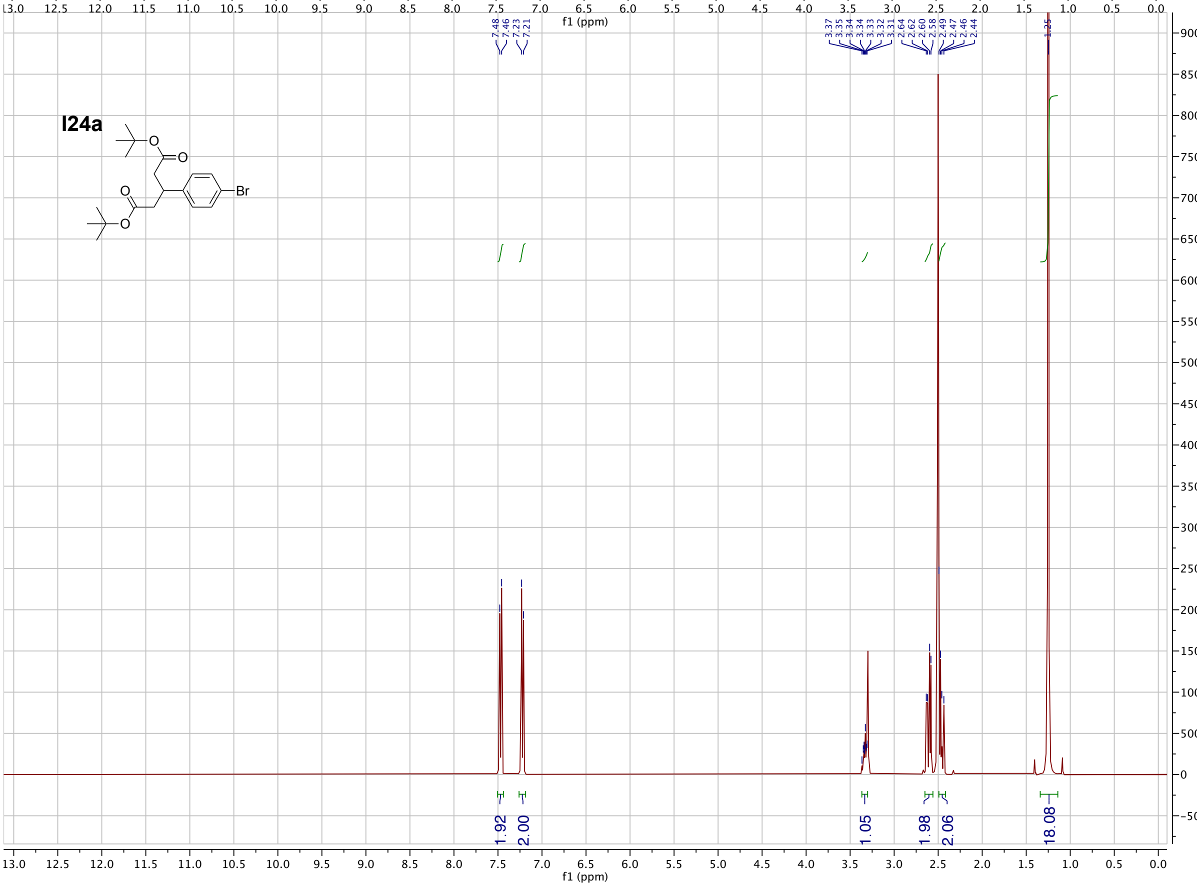
I24a
O
O
O
O
Br
f1 (ppm)

I24b

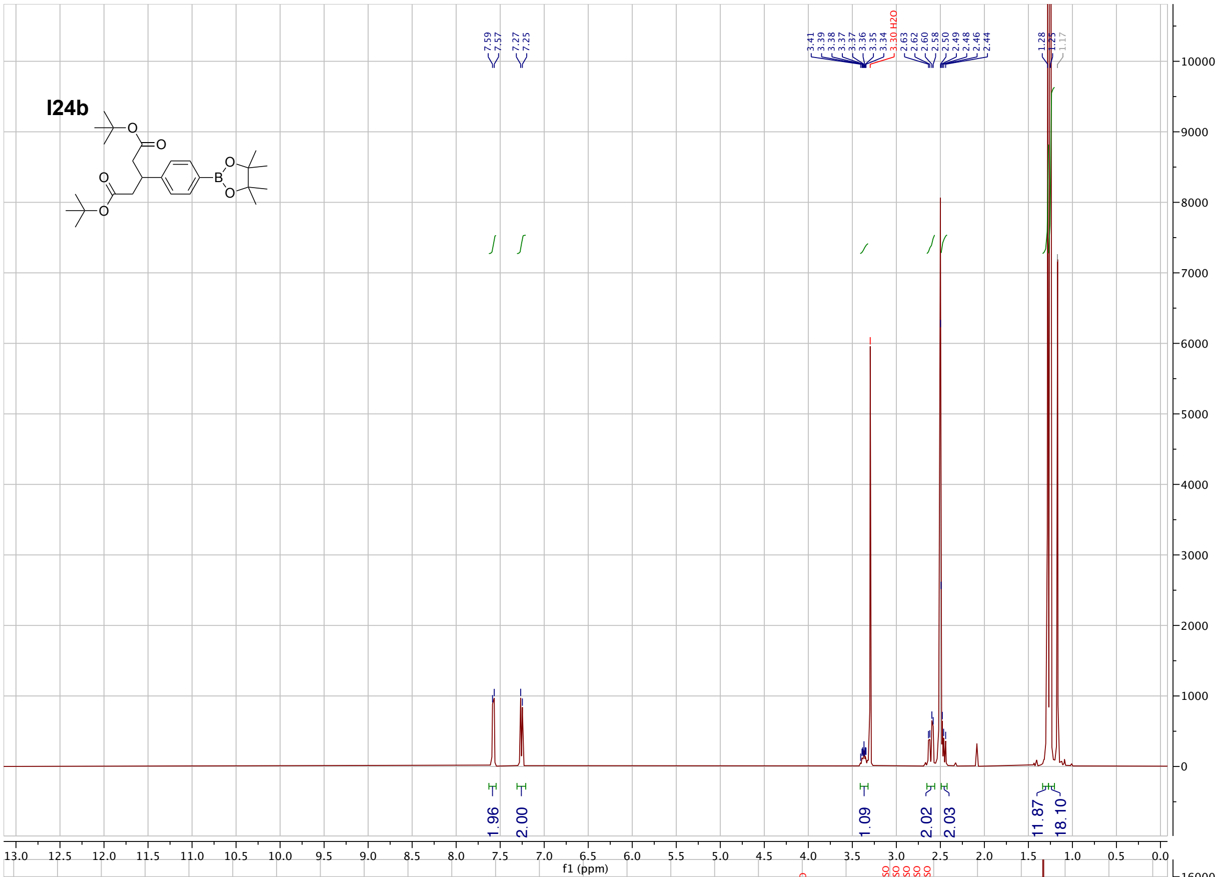

I24b
1.96
2.00
1.09
2.02
2.03
11.87
18.10
3.30 H2O
f1 (ppm)

I24c

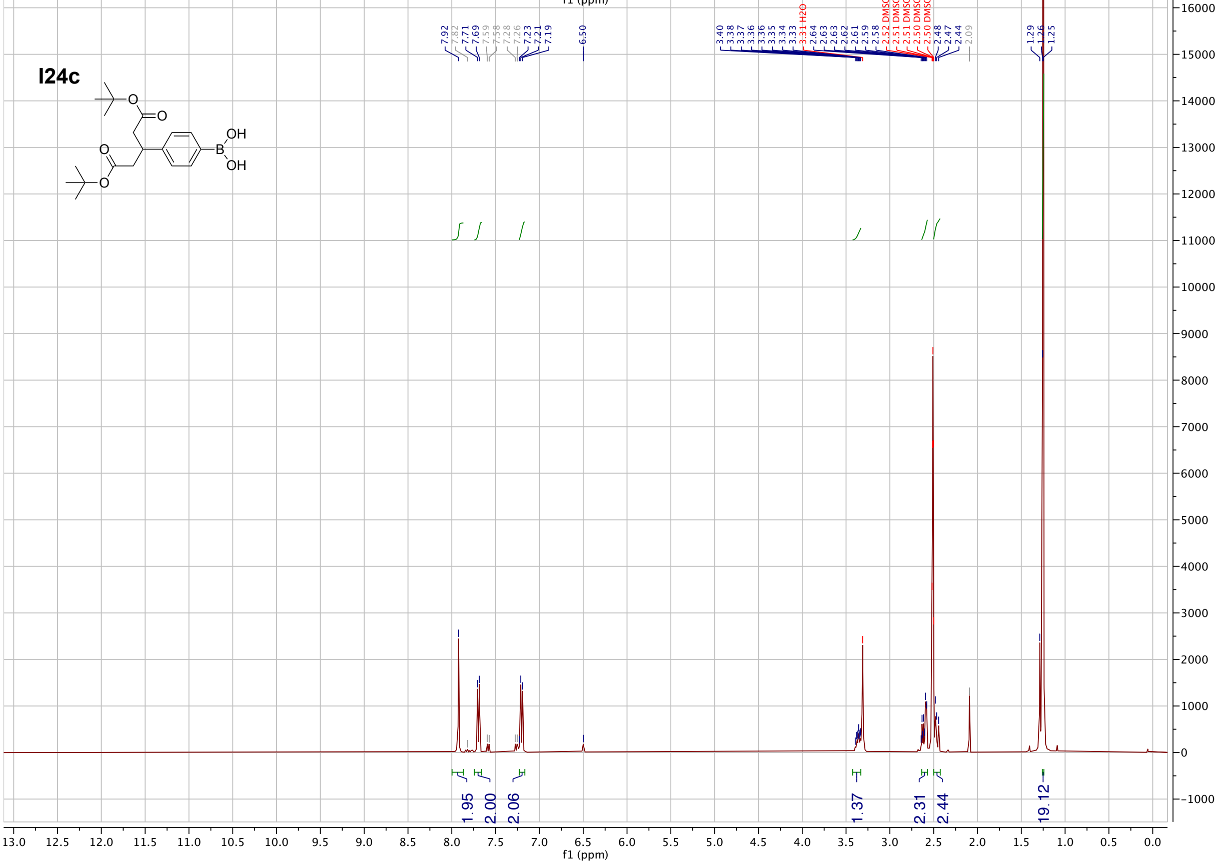

I24c
OH
OH
1.95
2.00
2.06
1.37
2.31
2.44
19.12
3.31 H2O
2.52 DMSO
2.51 DMSO
2.51 DMSO
2.50 DMSO
2.50 DMSO
f1 (ppm)

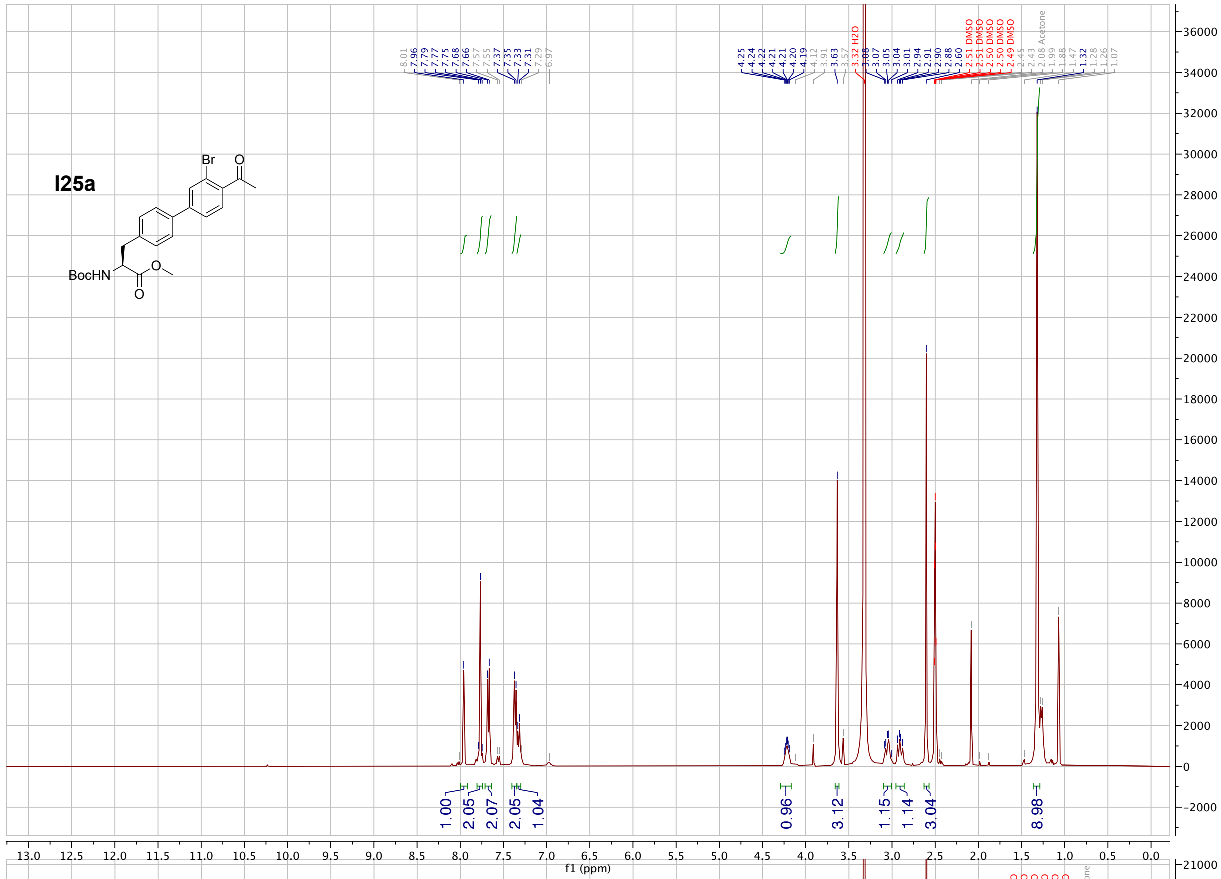
I25a
Br
O
BocHN
O
O
1.00
2.05
2.07
2.05
1.04
0.96
3.12
1.15
1.14
3.04
8.98
3.32 H2O
2.51 DMSO
2.51 DMSO
2.50 DMSO
2.50 DMSO
2.49 DMSO
2.08 Acetone
f1 (ppm)

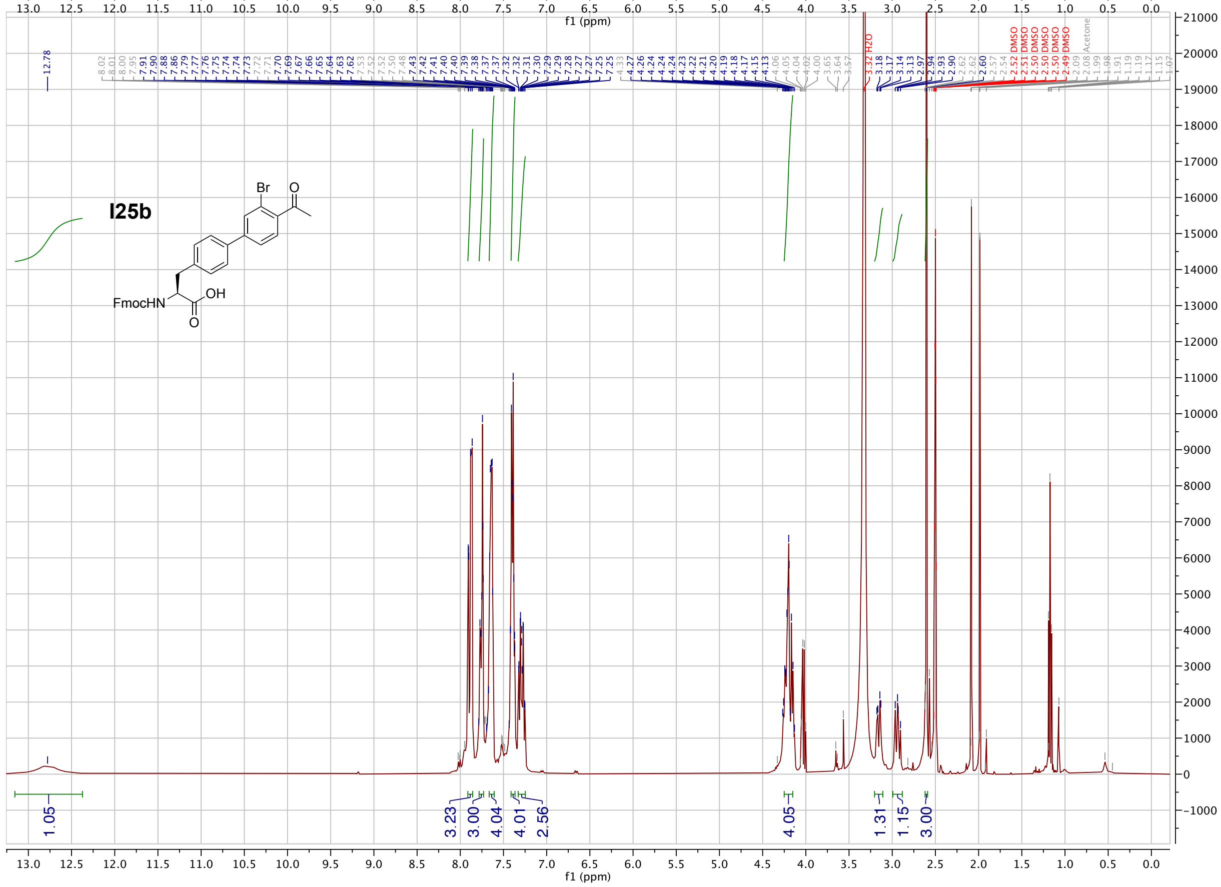
I25b
Br
O
FmocHN
OH
O
12.78
1.05
3.23
3.00
4.04
4.01
2.56
4.05
1.31
1.15
3.00
3.32 H2O
2.52 DMSO
2.51 DMSO
2.50 DMSO
2.50 DMSO
2.49 DMSO
2.09 Acetone
f1 (ppm)

I25c

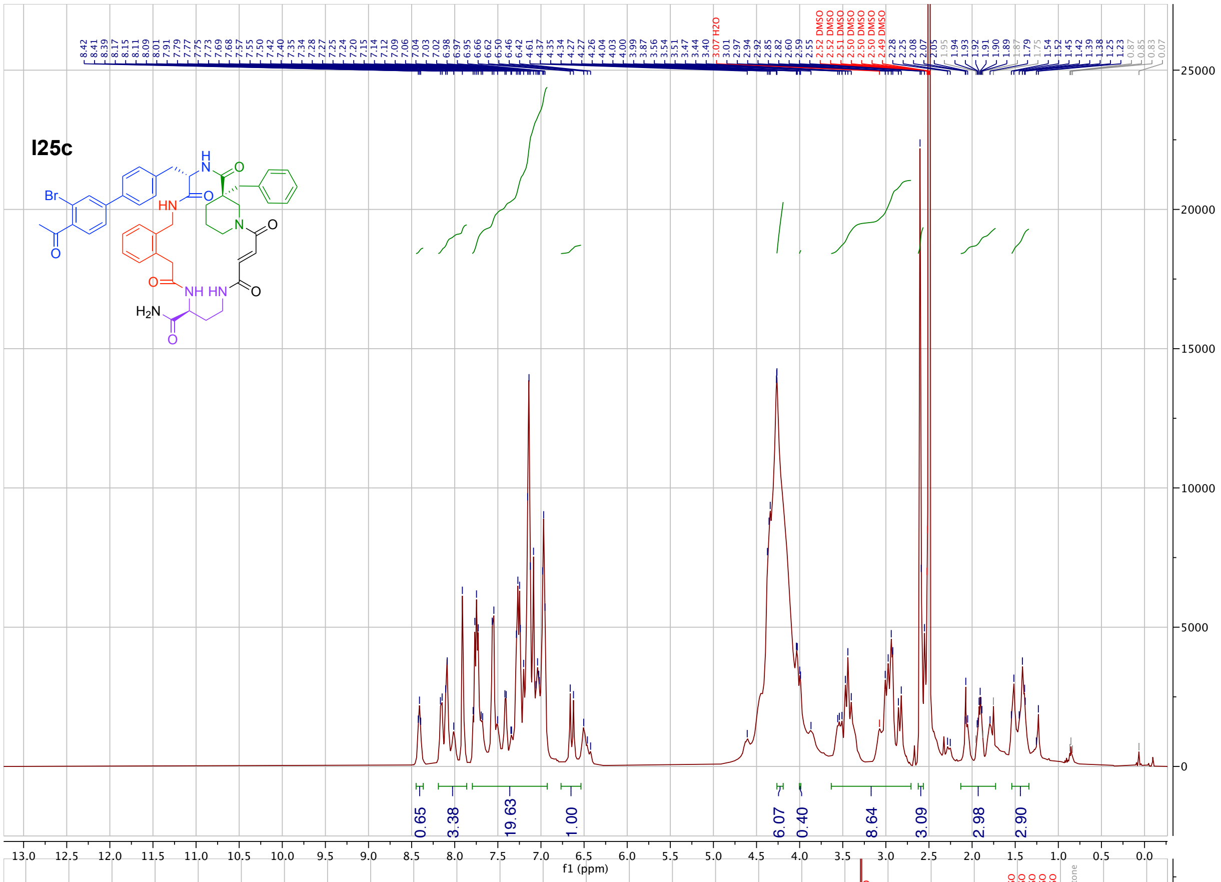

I26a

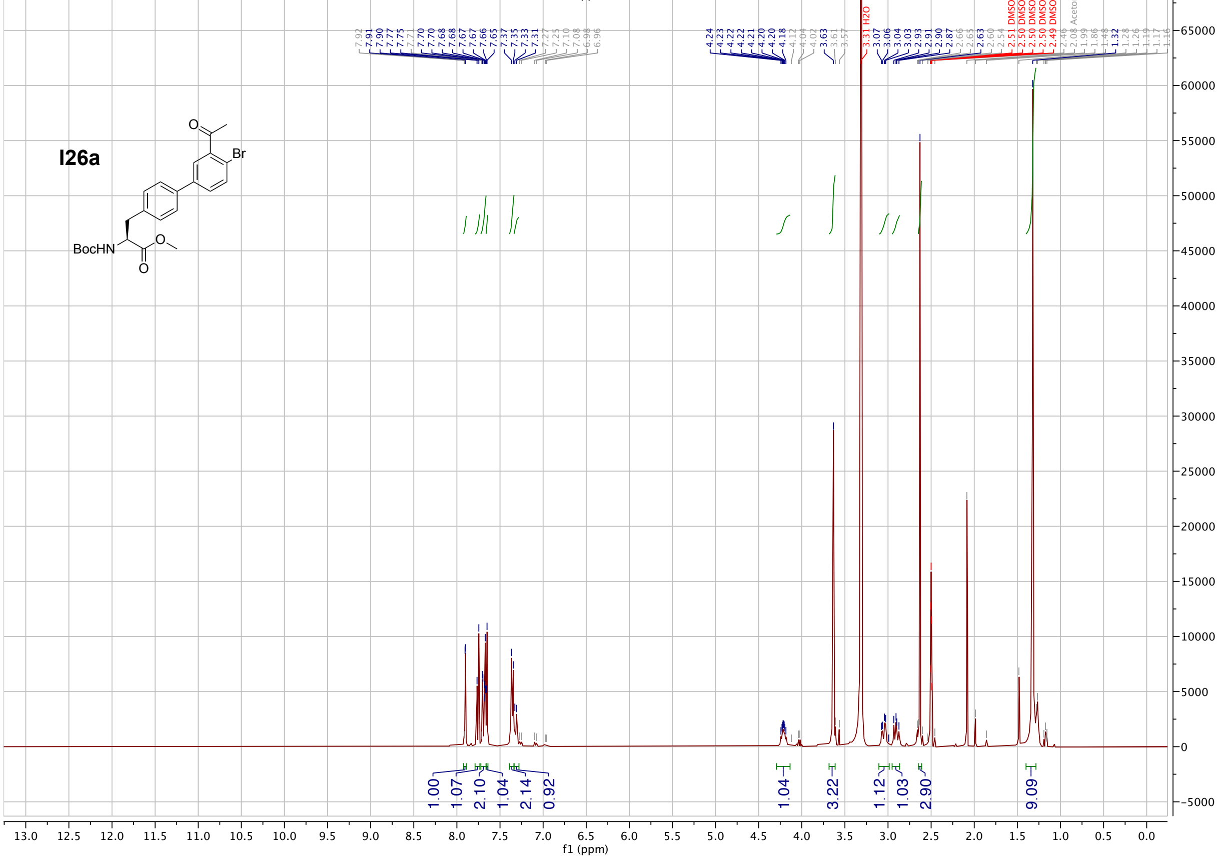

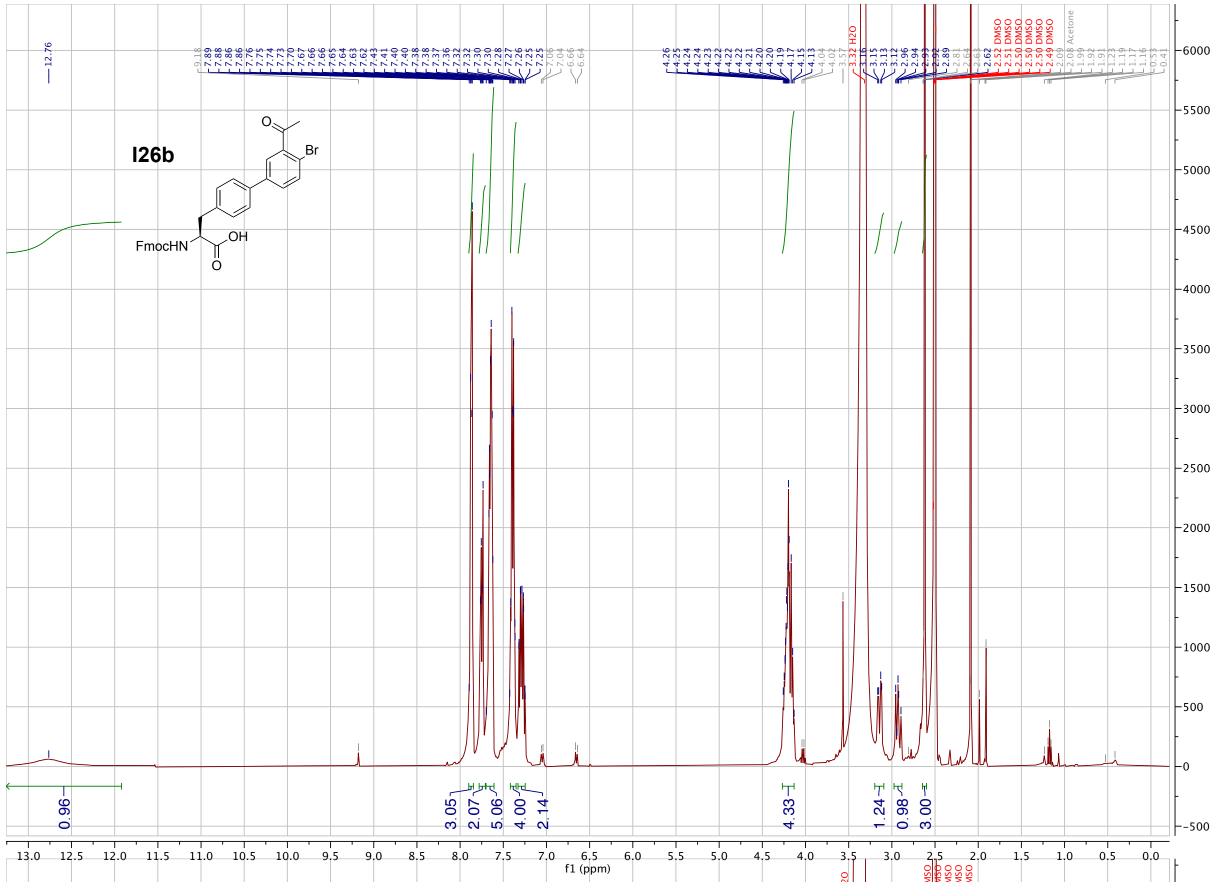
I26b
FmocHN
OH
Br
f1 (ppm)

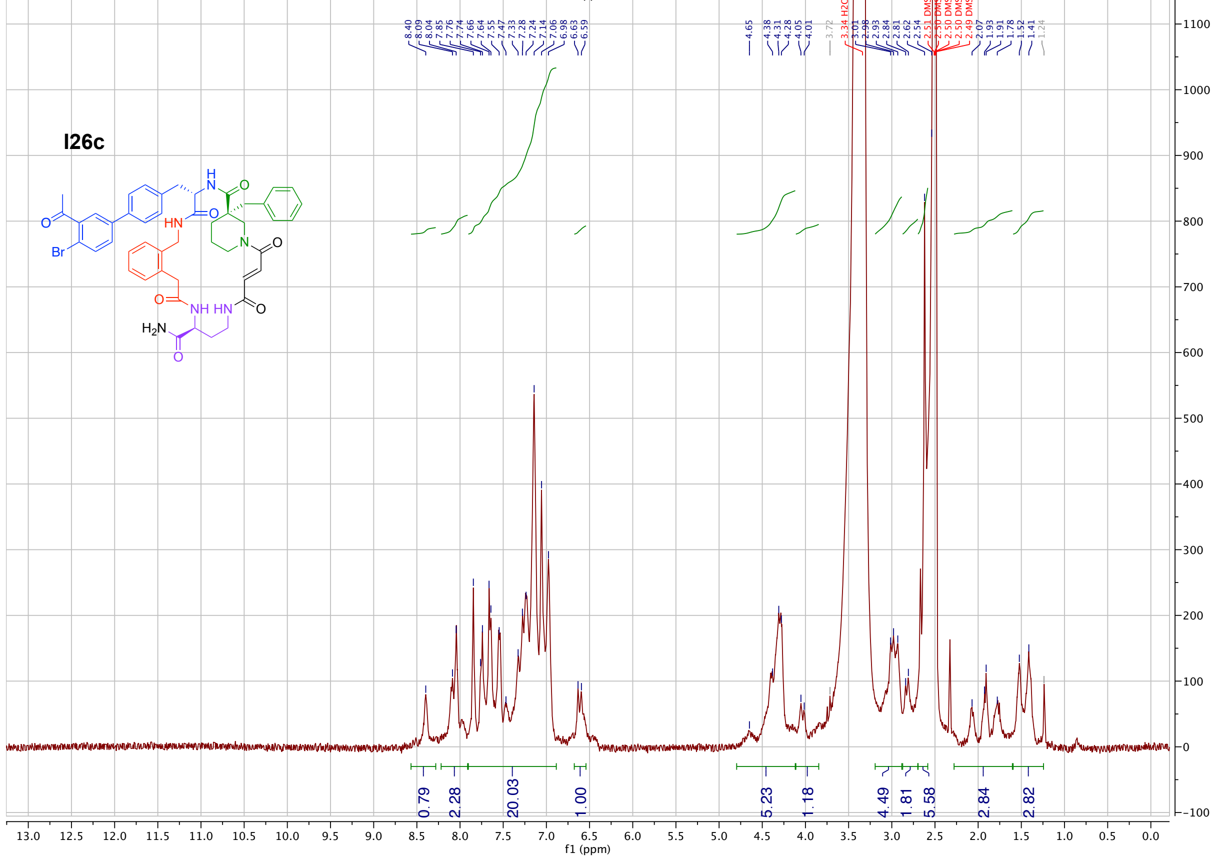
I26c
Br
HN
NH HN
f1 (ppm)

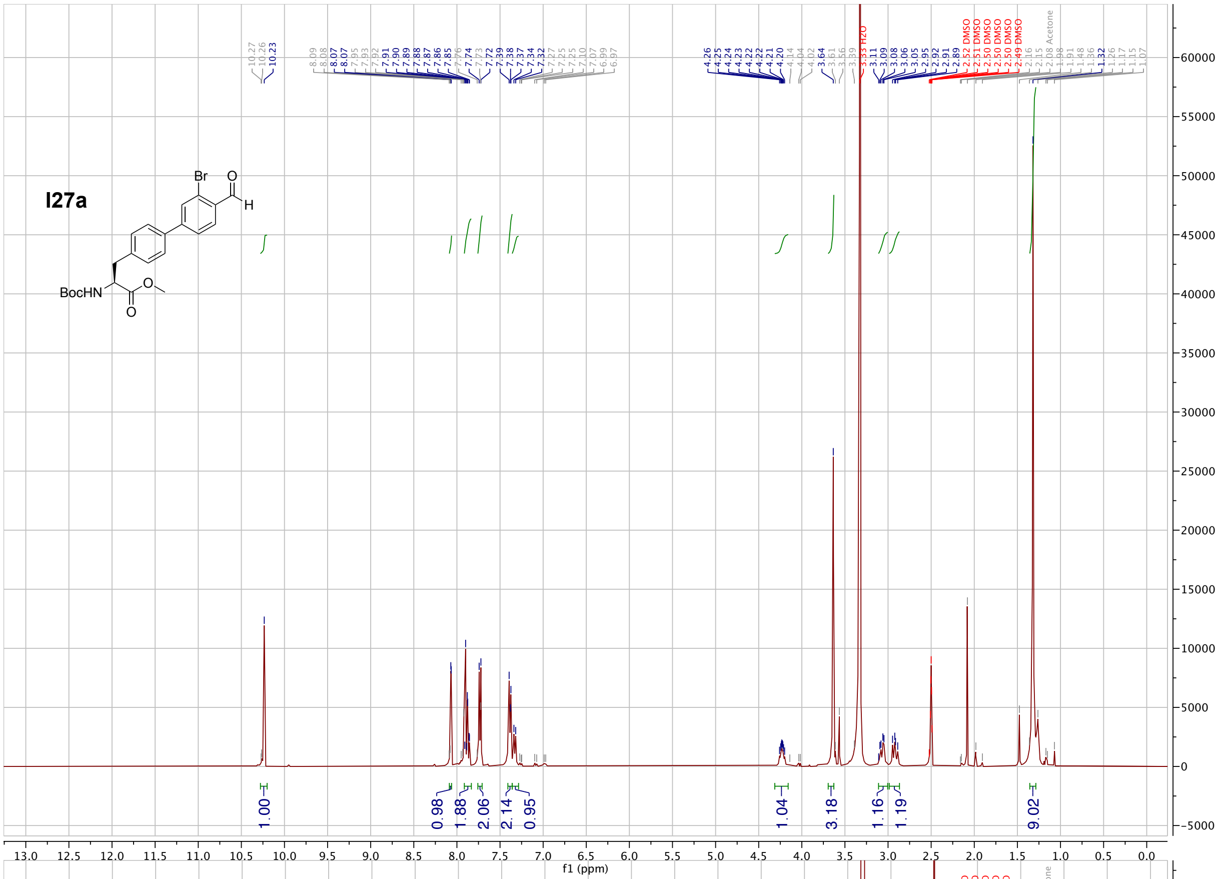
I27a
BocHN
Br
O
H
f1 (ppm)

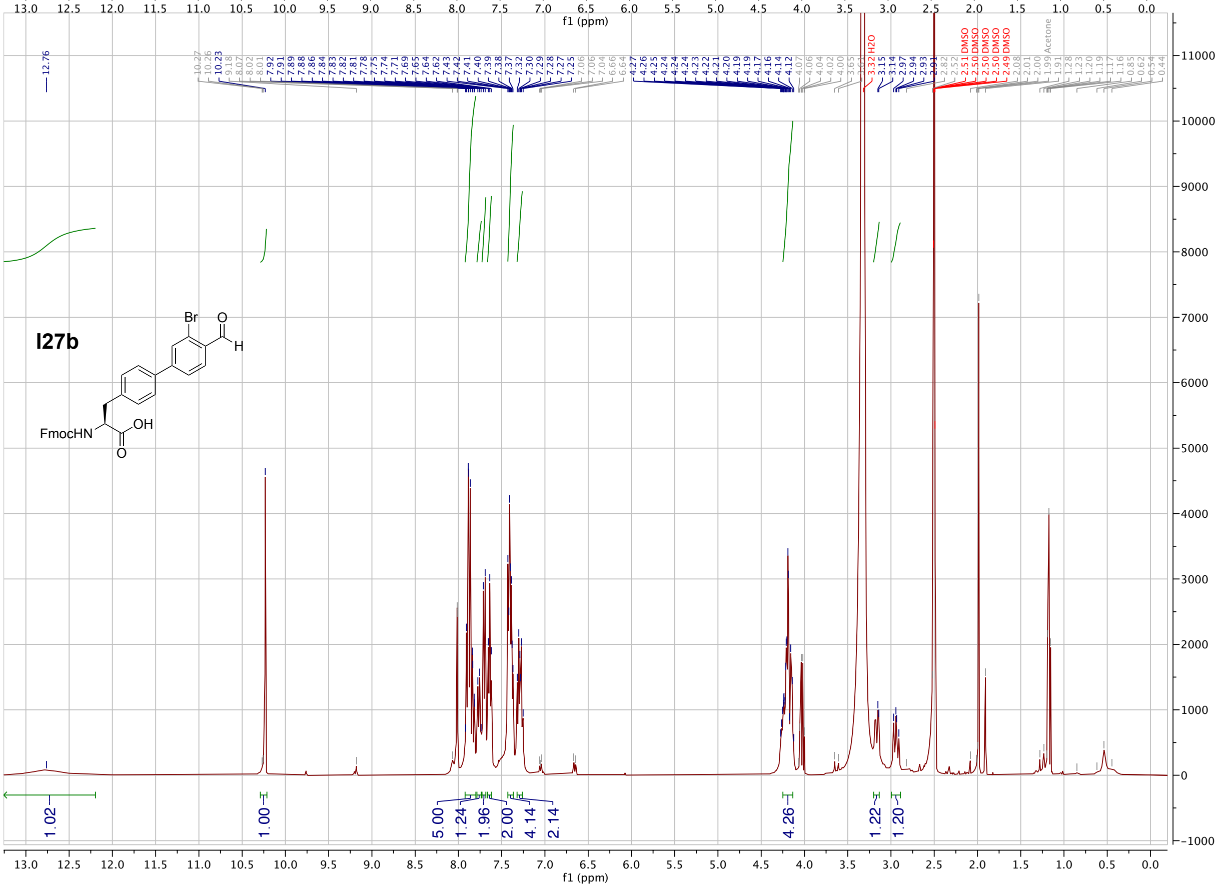
I27b
FmocHN
OH
Br
O
H
f1 (ppm)

I27c

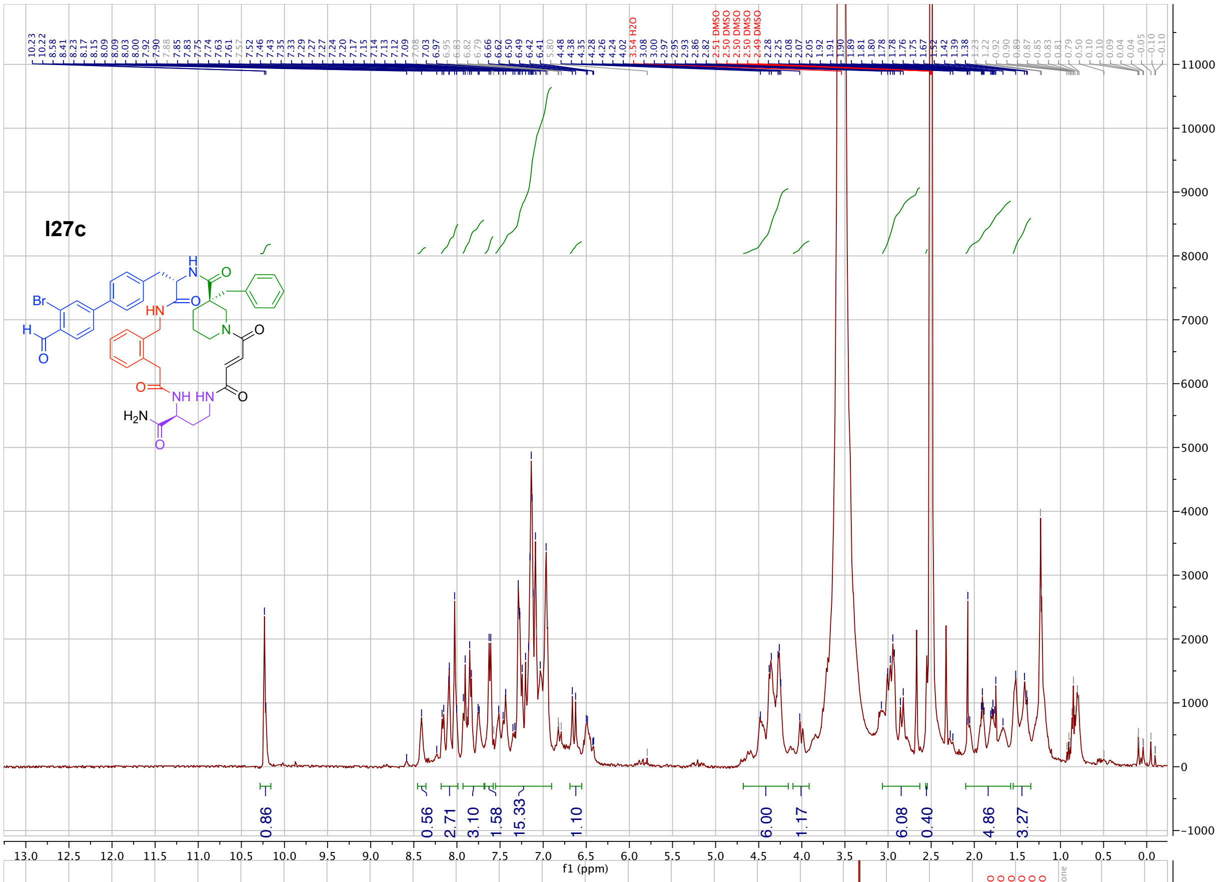

I28a

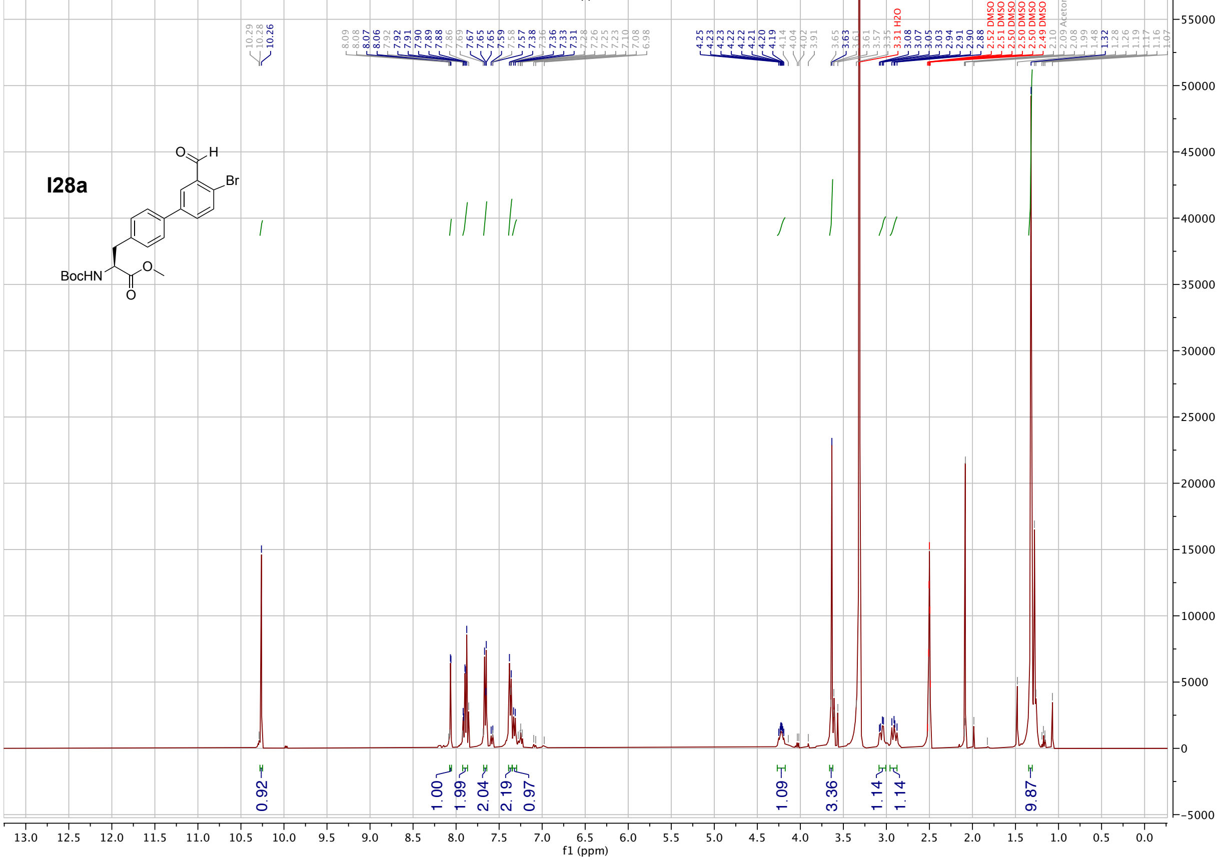

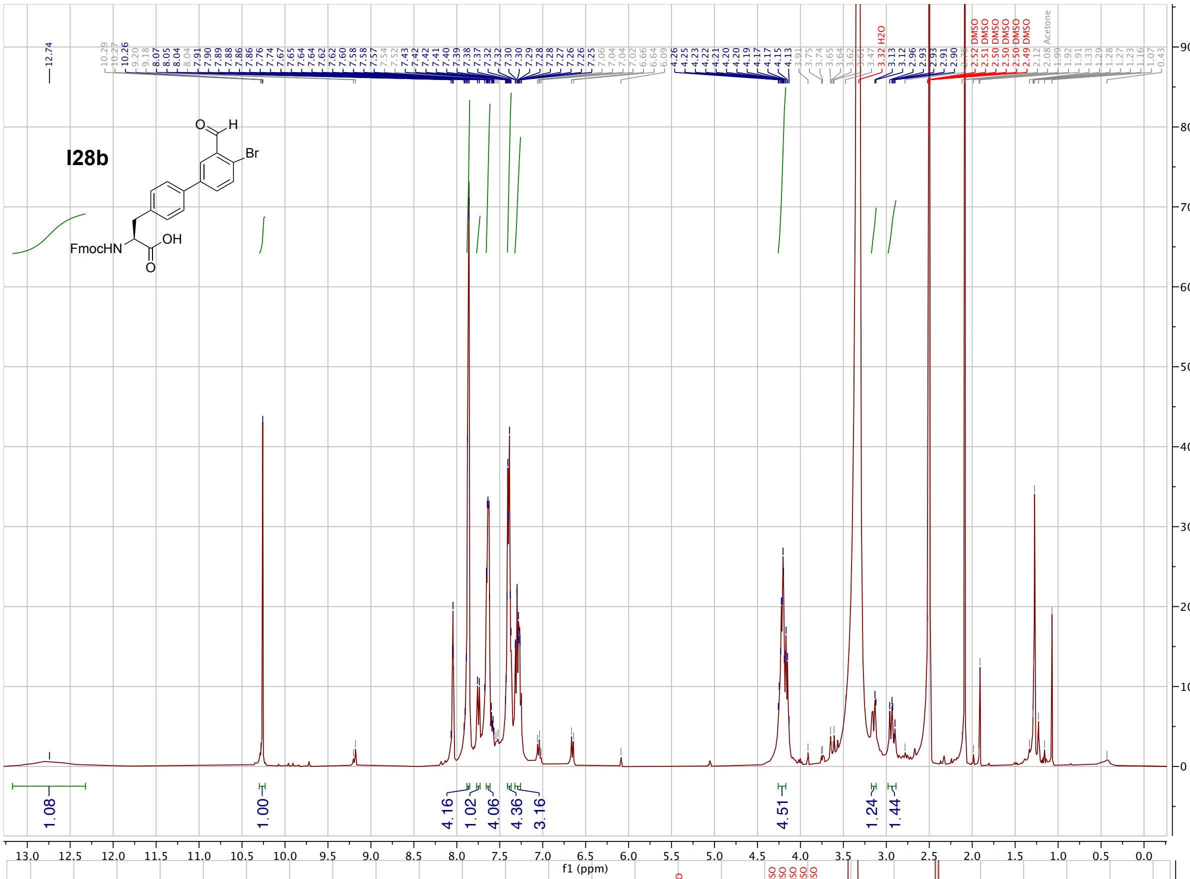
I28b
FmocHN
OH
Br
f1 (ppm)

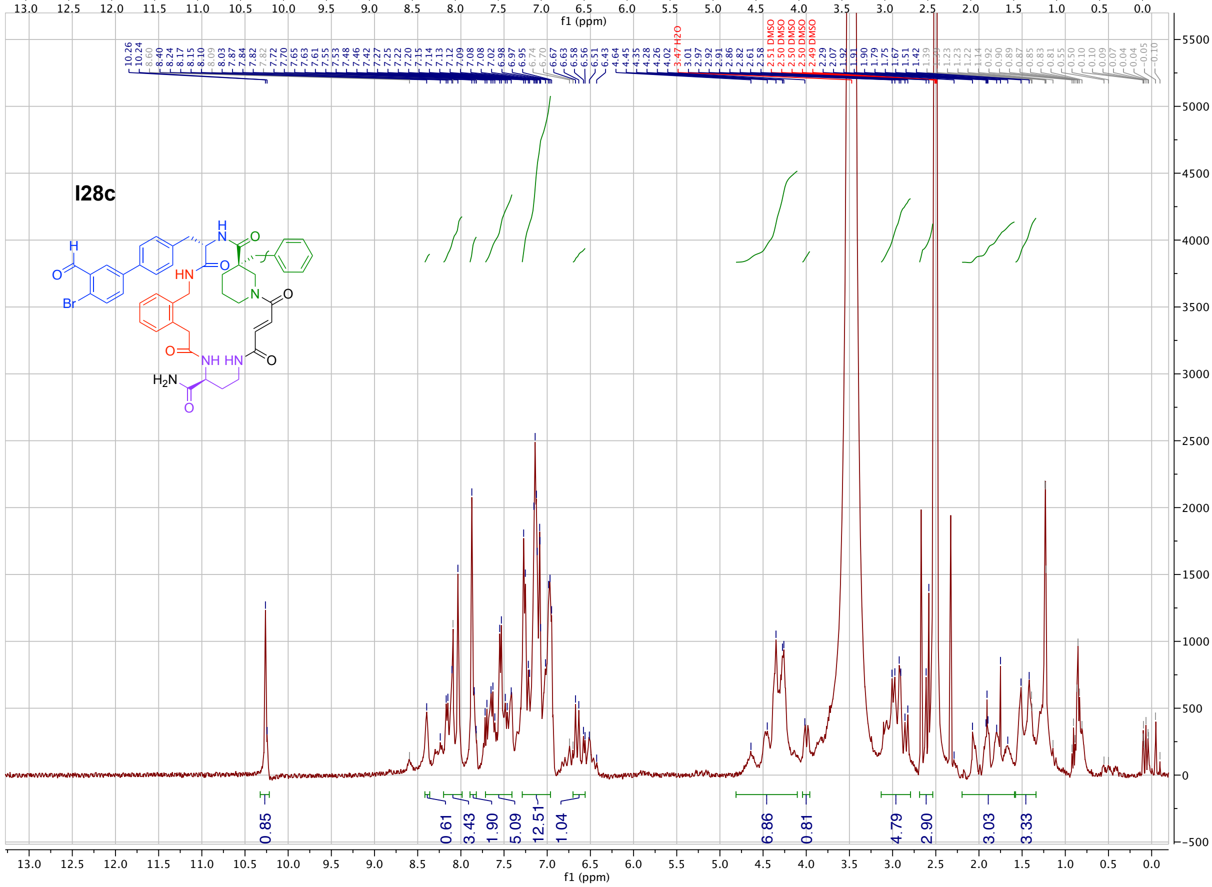
I28c
Br
HN
NH
H2N
f1 (ppm)

I29a

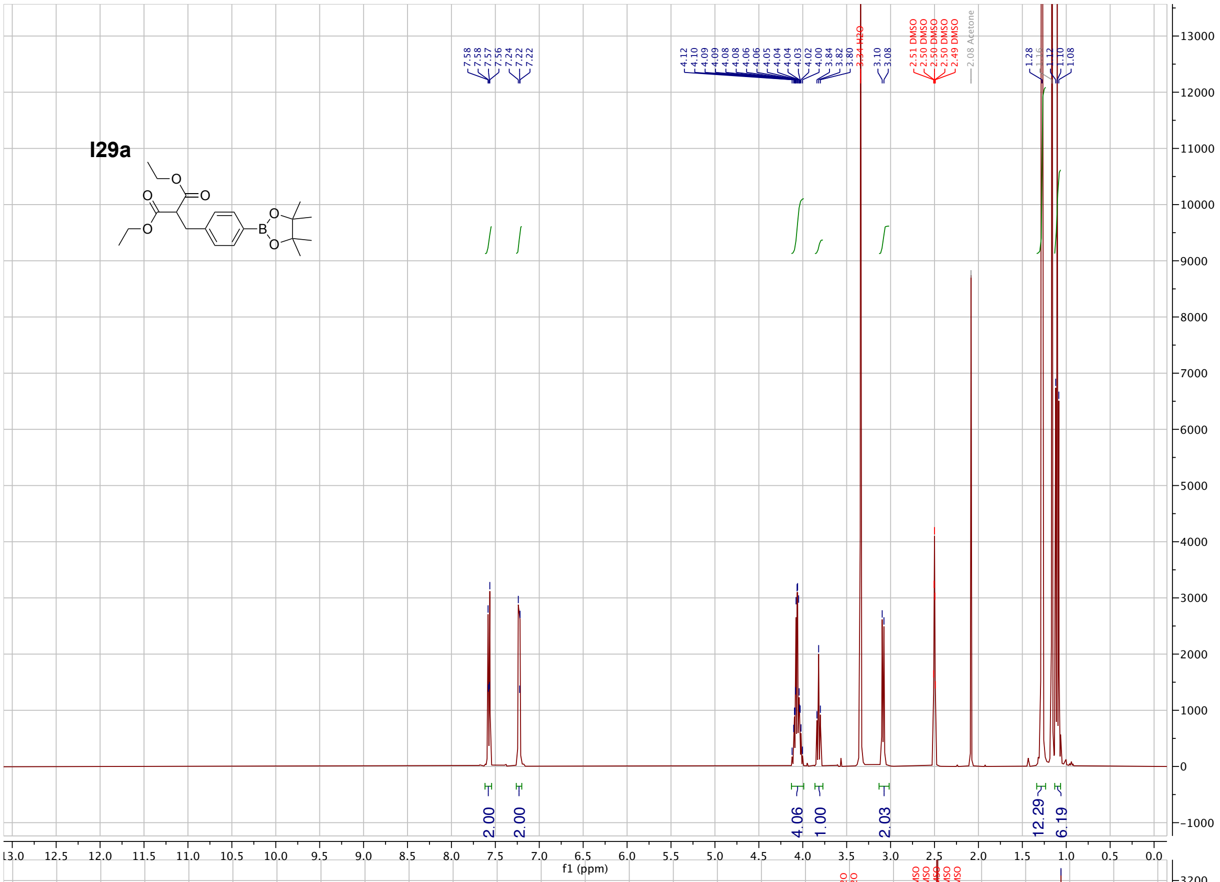
f1 (ppm)
2.00
2.00
4.06
1.00
2.03
12.29
6.19

I29b

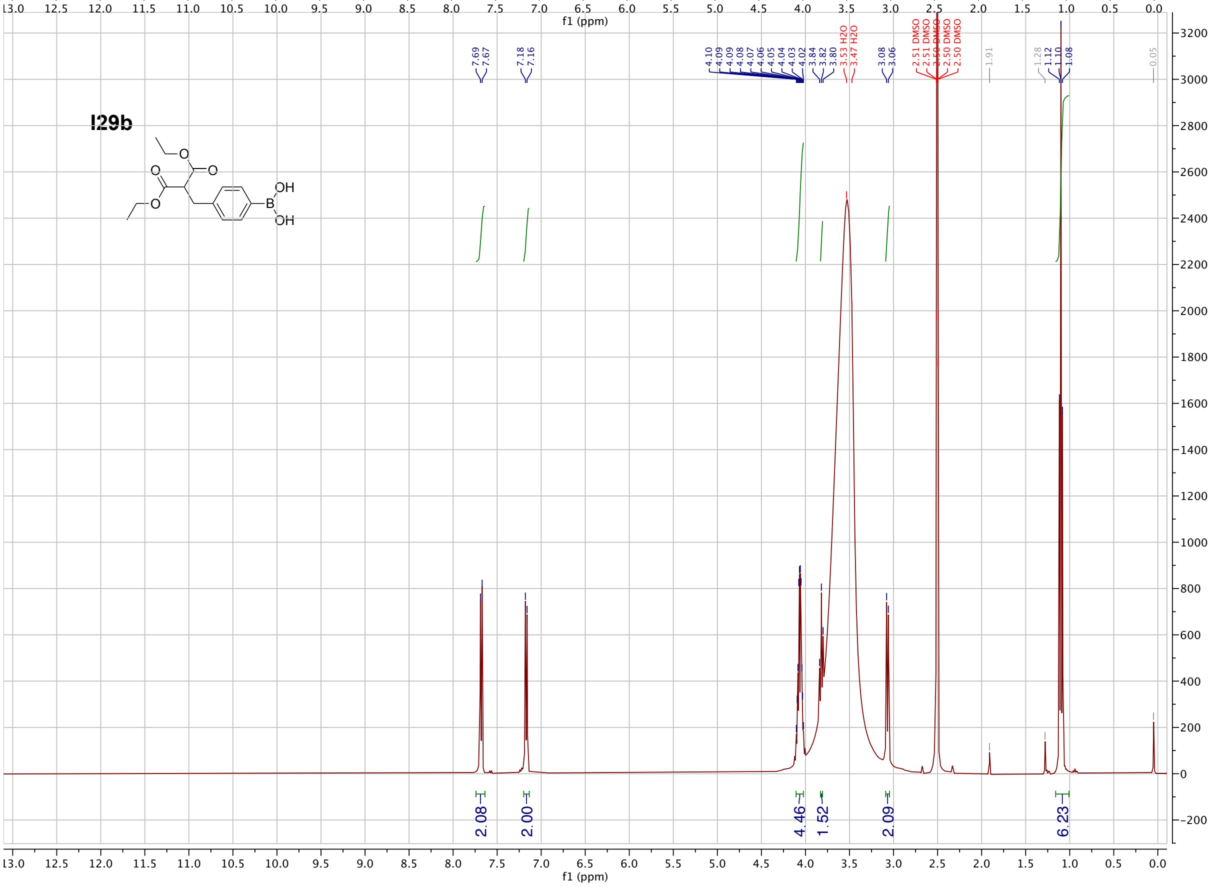
f1 (ppm)
2.08
2.00
4.46
1.52
2.09
6.23

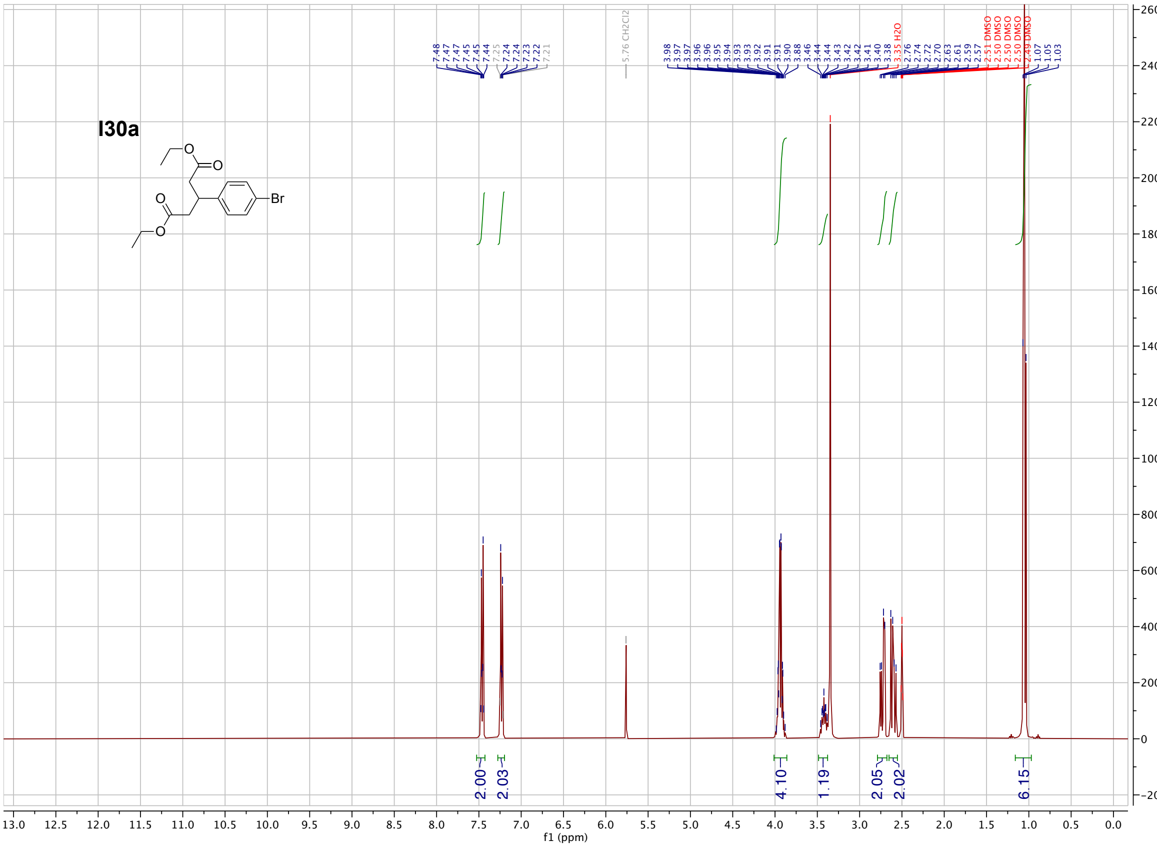
I30a
Br
f1 (ppm)

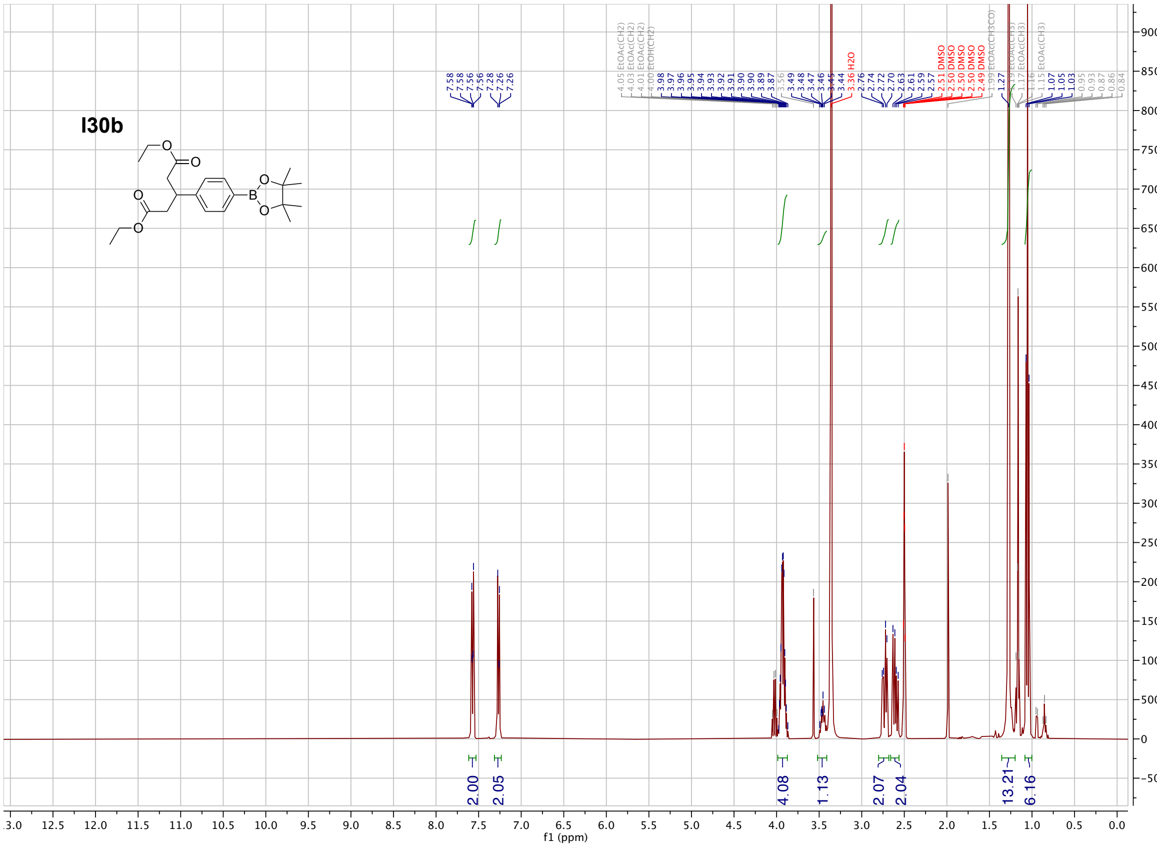
I30b
f1 (ppm)

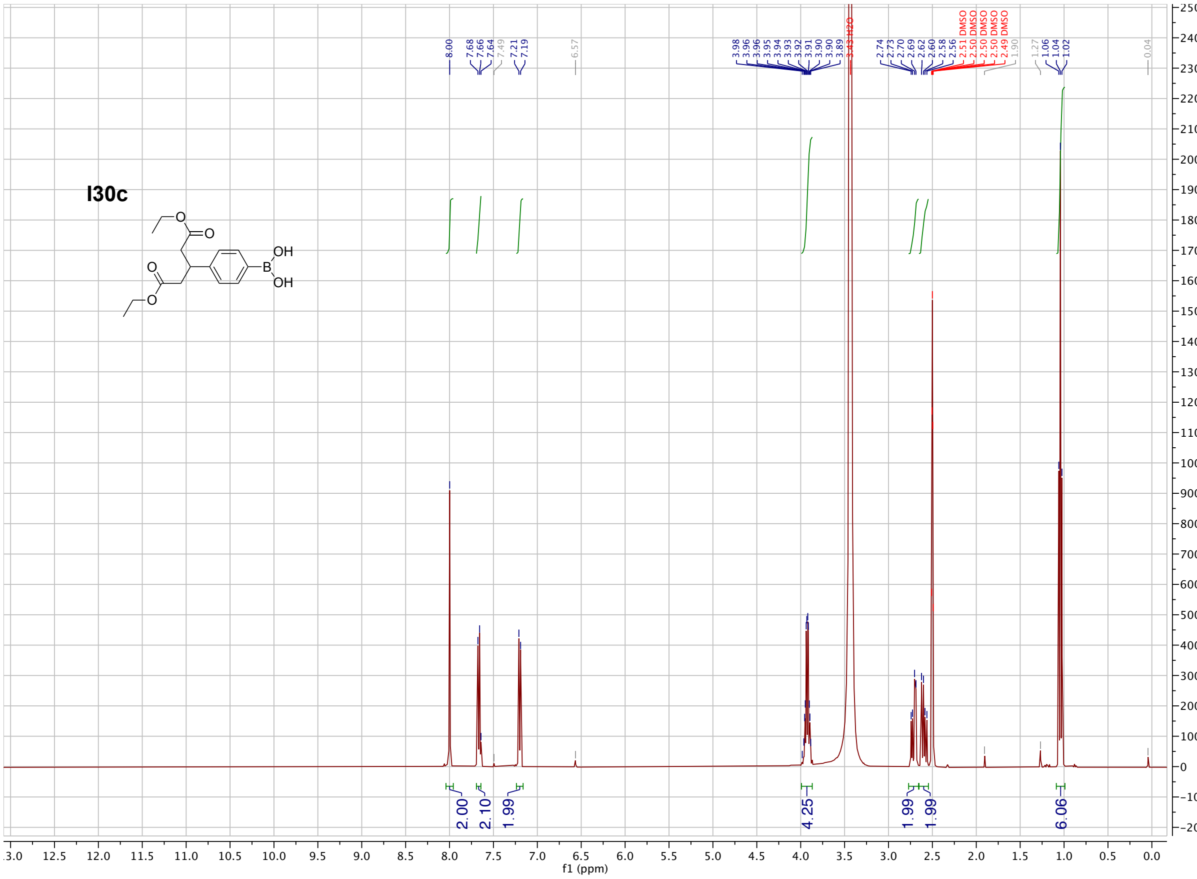

(4Br)B6

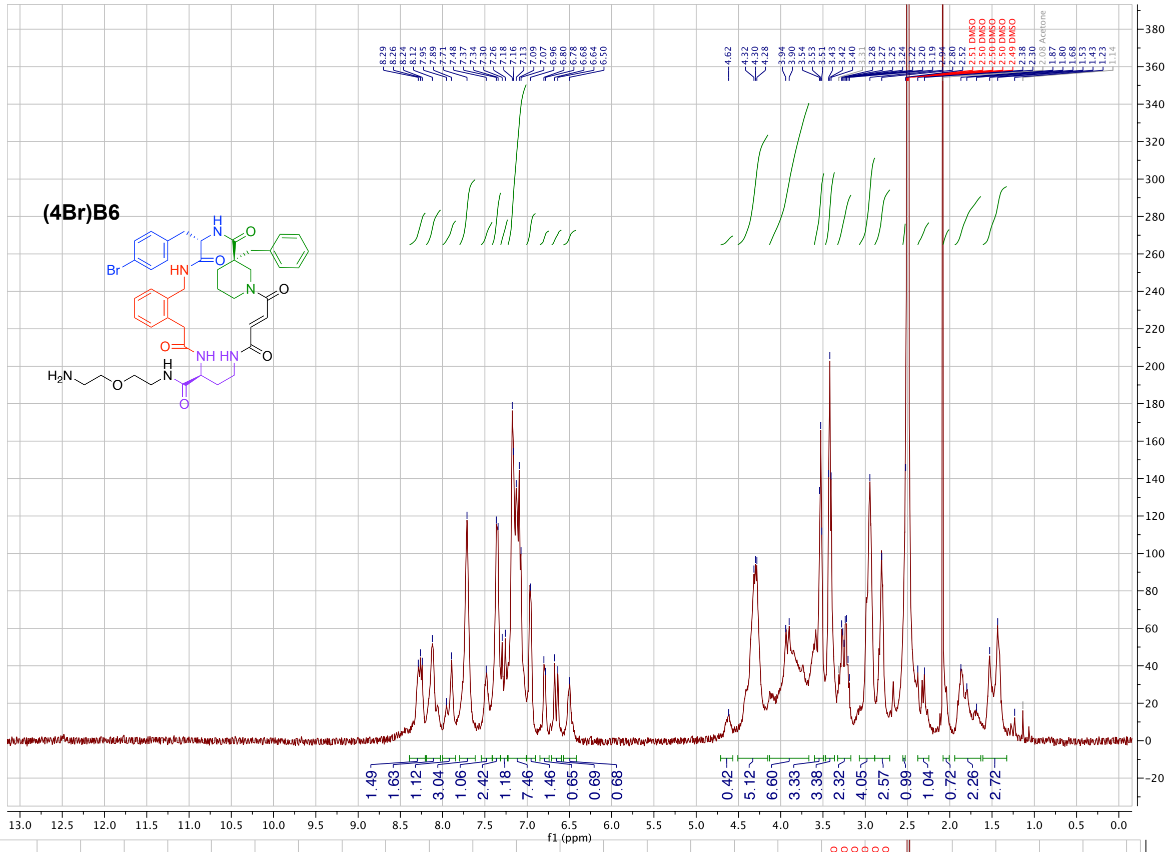

(4Br)B6-A

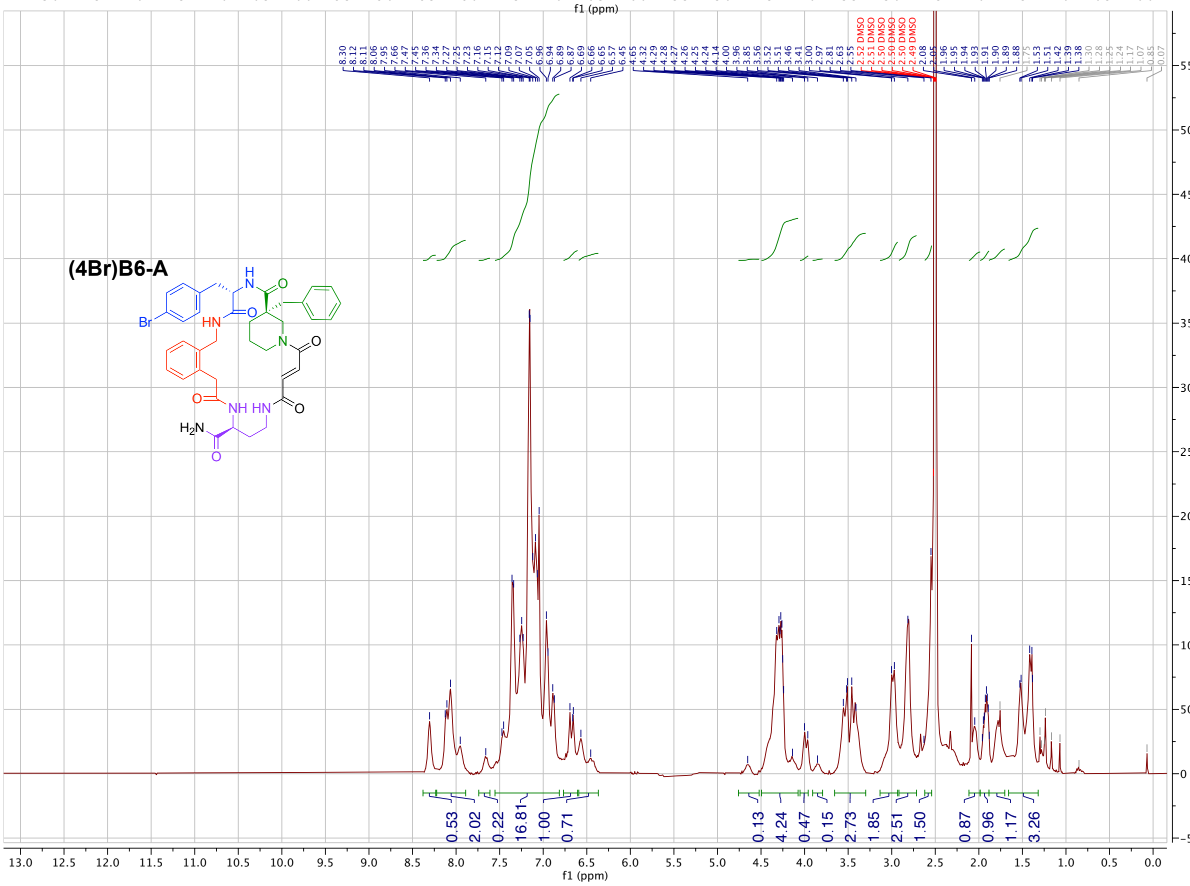

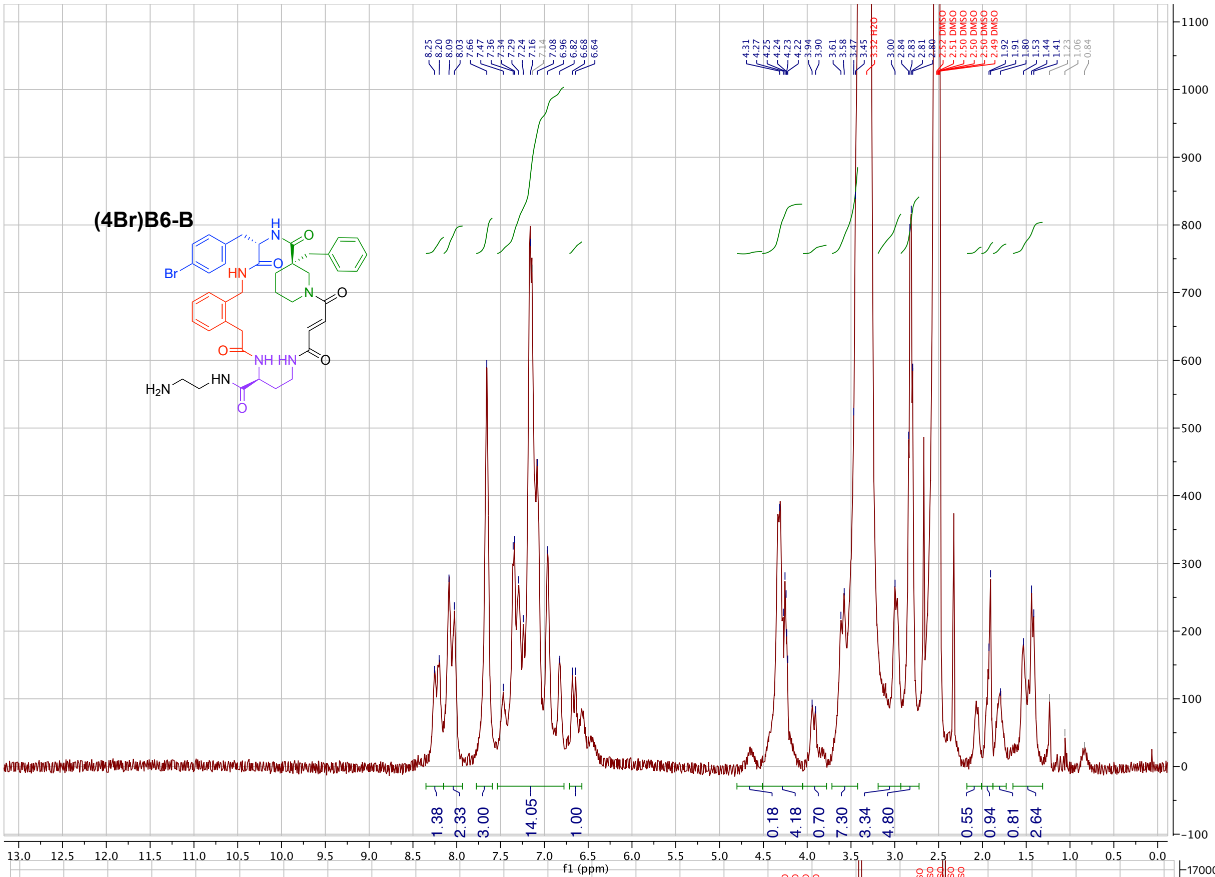
(4Br)B6-B
f1 (ppm)

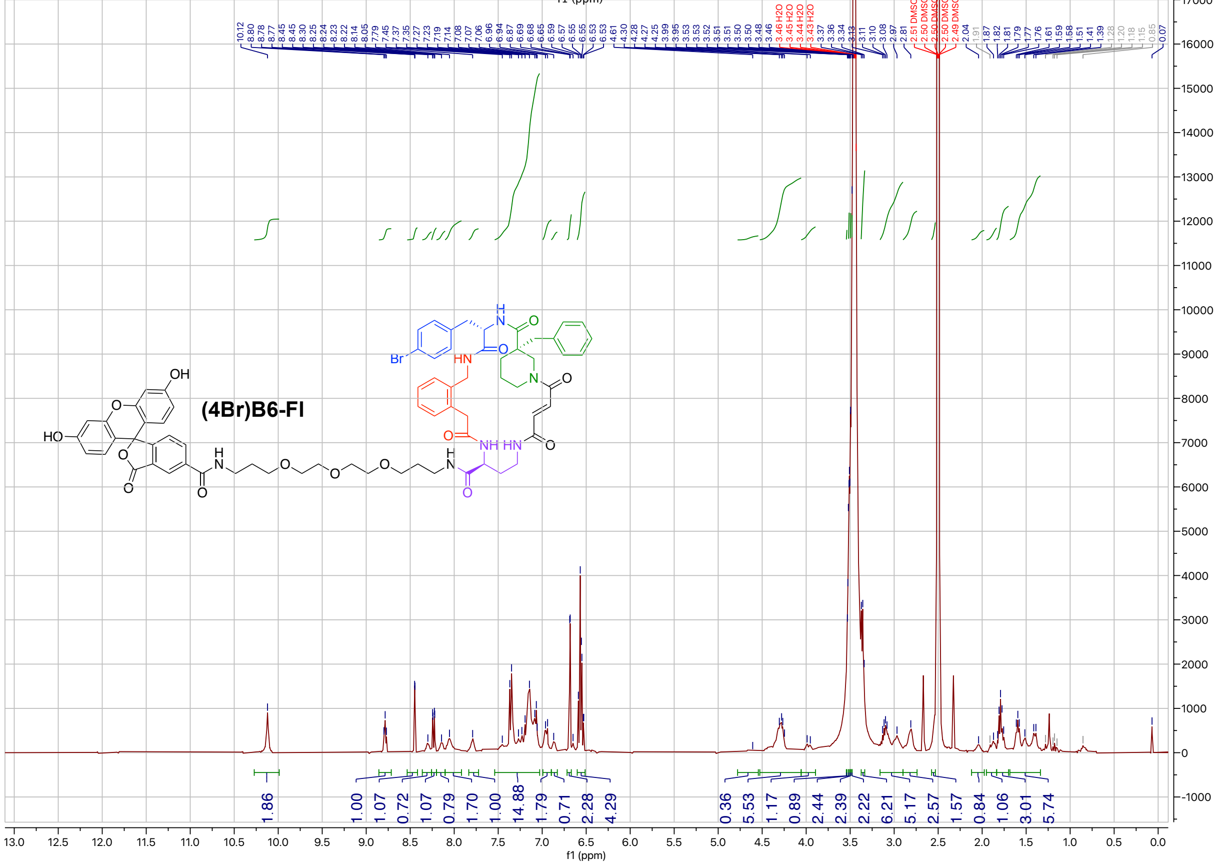
(4Br)B6-Fl
f1 (ppm)

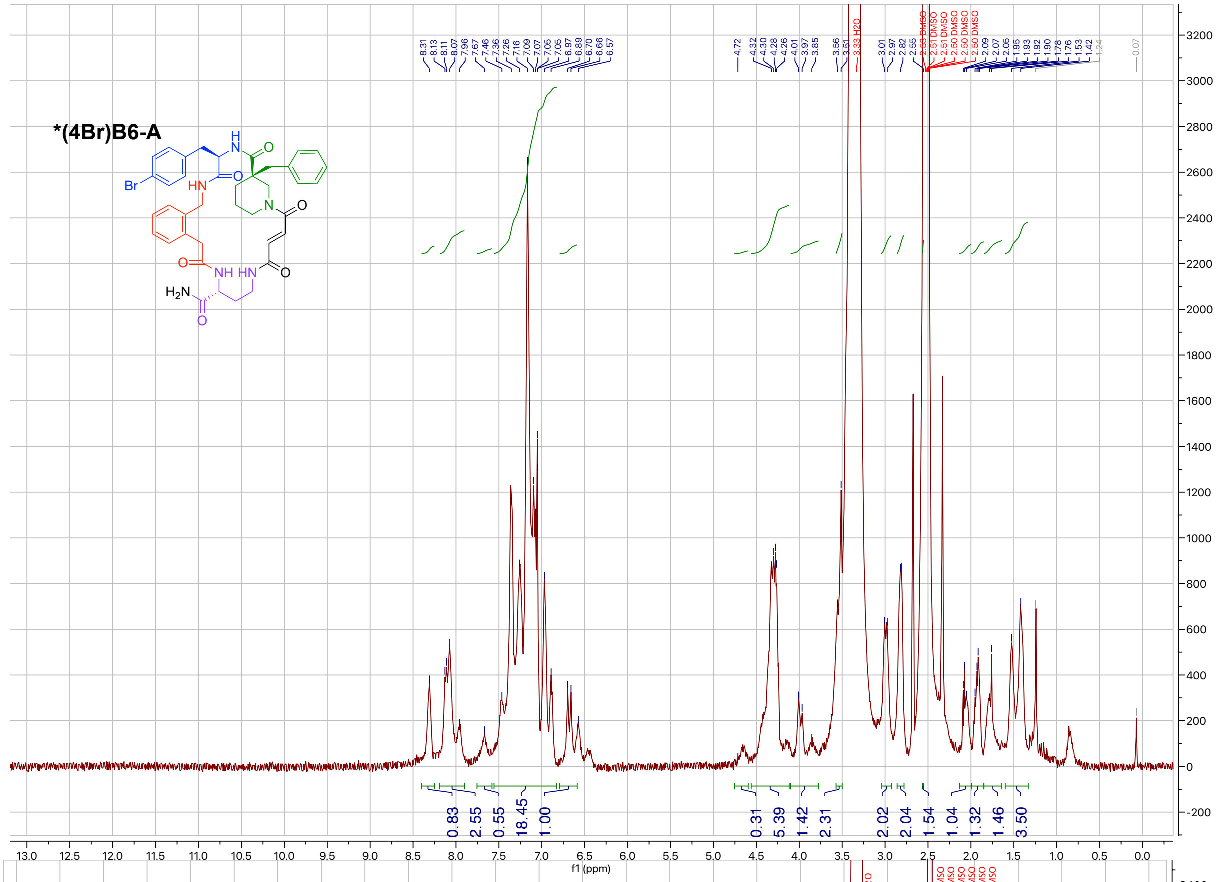
*(4Br)B6-A
f1 (ppm)

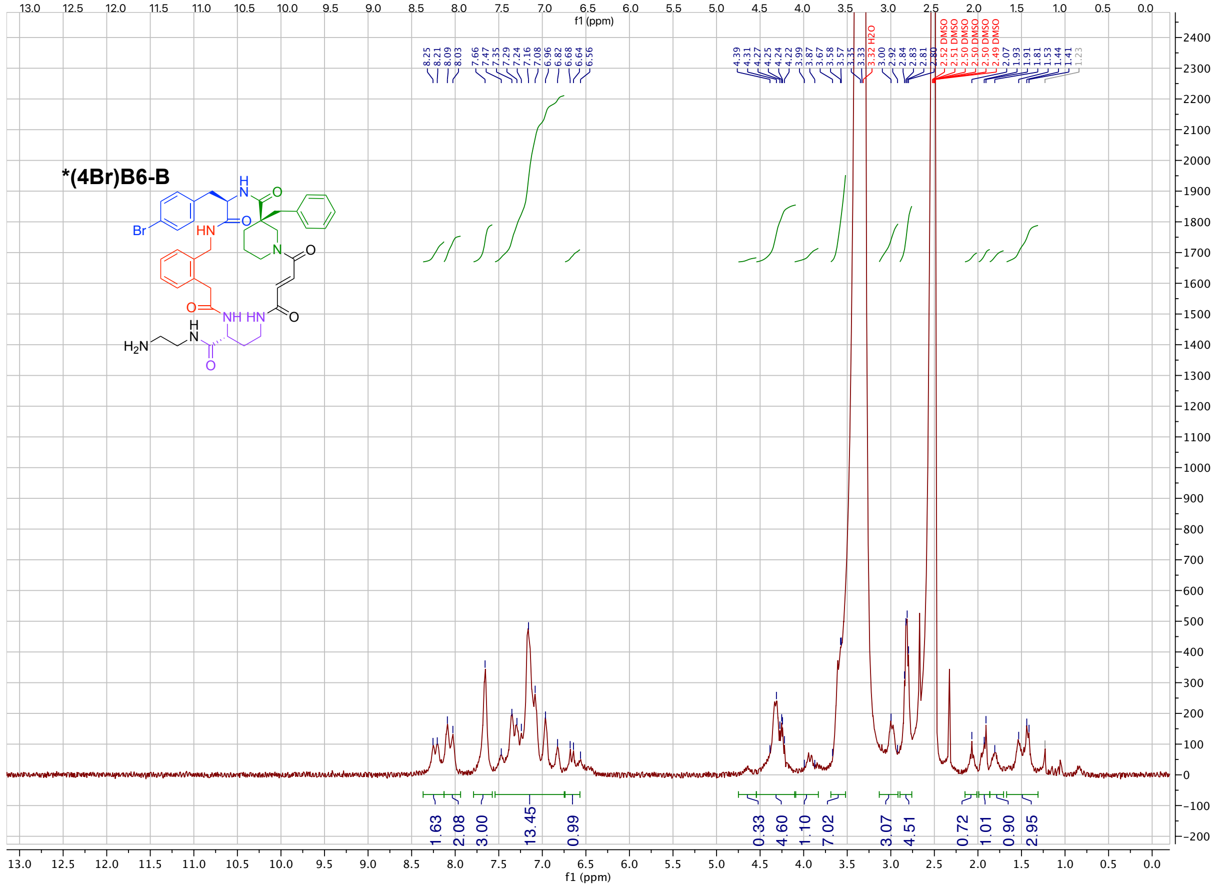
*(4Br)B6-B
f1 (ppm)

HPLC Chromatograms

Panels from top→bottom:
1. MS trace for compound ions (M, $M+H^+$, $M+Na^+$)
2. MS scan trace for all ions
3. Absorbance at 214 nM
4. Absorbance at 230 nM
5. Absorbance at 254 nM
6. Absorbance at 280 nM
7. Solvent gradient, with % acetonitrile shown on y-axis
8. Extracted ion chromatogram for $M+H^+$ ion

Purity of compound was determined by % area of the indicated compound peak at 214 nM relative to all identified peaks between 3 and 17 minutes. DMSO peak occurs at 1.5 minutes.

JOMBt

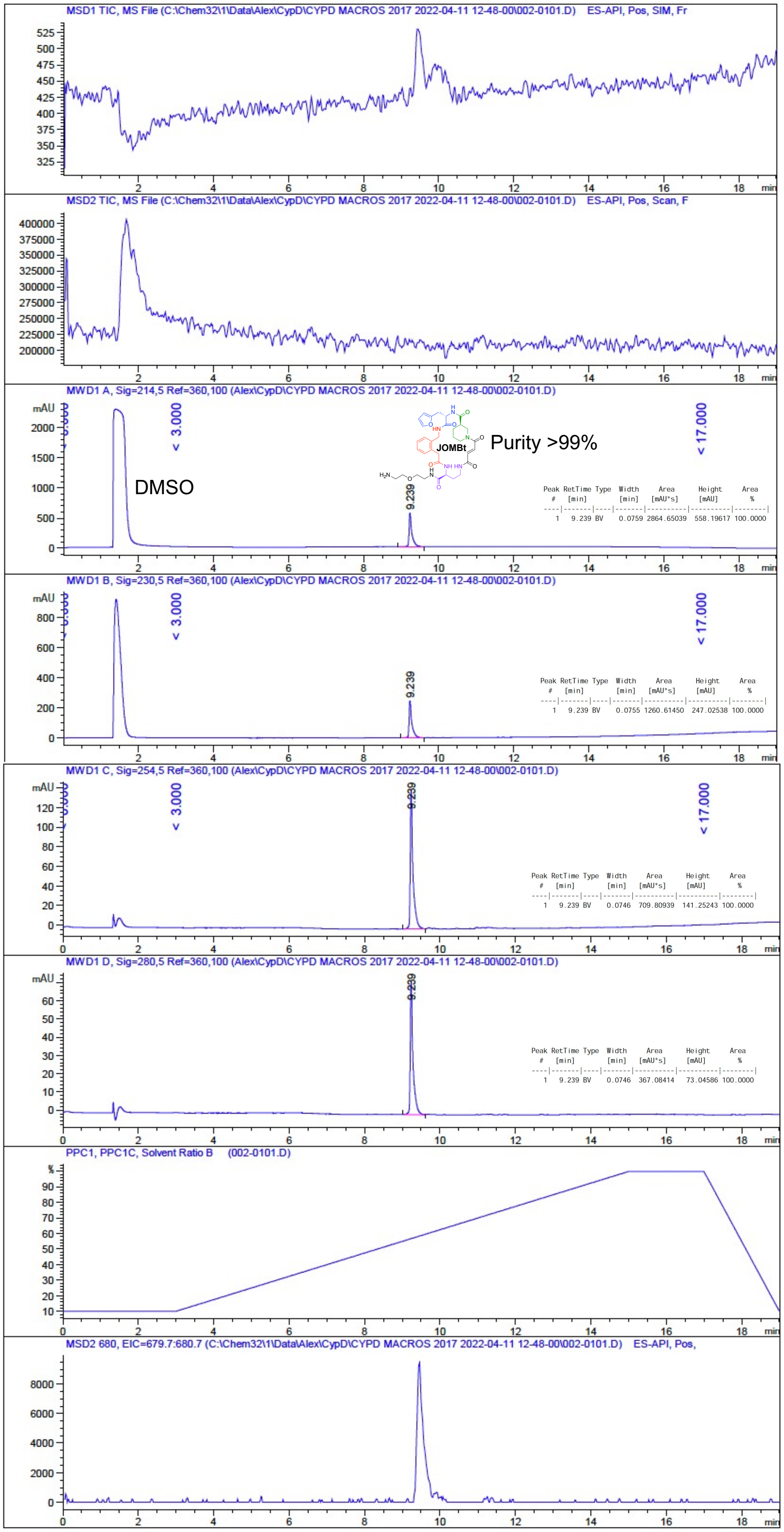
MSD1 TIC, MS File (C:\Chem32\1\Data\Alex\CypD\CYPD MACROS 2017 2022-04-11 12-48-00\002-0101.D) ES-API, Pos, SIM, Fr
MSD2 TIC, MS File (C:\Chem32\1\Data\Alex\CypD\CYPD MACROS 2017 2022-04-11 12-48-00\002-0101.D) ES-API, Pos, Scan, F
MWD1 A, Sig=214,5 Ref=360,100 (Alex\CypD\CYPD MACROS 2017 2022-04-11 12-48-00\002-0101.D)
JOMBt
Purity >99%
DMSO
Peak RetTime Type Width Area Height Area
1 9.239 BV 0.0759 2864.65039 558.19617 100.0000
MWD1 B, Sig=230,5 Ref=360,100 (Alex\CypD\CYPD MACROS 2017 2022-04-11 12-48-00\002-0101.D)
1 9.239 BV 0.0755 1260.61450 247.02538 100.0000
MWD1 C, Sig=254,5 Ref=360,100 (Alex\CypD\CYPD MACROS 2017 2022-04-11 12-48-00\002-0101.D)
1 9.239 BV 0.0746 709.80939 141.25243 100.0000
MWD1 D, Sig=280,5 Ref=360,100 (Alex\CypD\CYPD MACROS 2017 2022-04-11 12-48-00\002-0101.D)
1 9.239 BV 0.0746 367.08414 73.04586 100.0000
PPC1, PPC1C, Solvent Ratio B (002-0101.D)
MSD2 680, EIC=679.7:680.7 (C:\Chem32\1\Data\Alex\CypD\CYPD MACROS 2017 2022-04-11 12-48-00\002-0101.D) ES-API, Pos,

A26

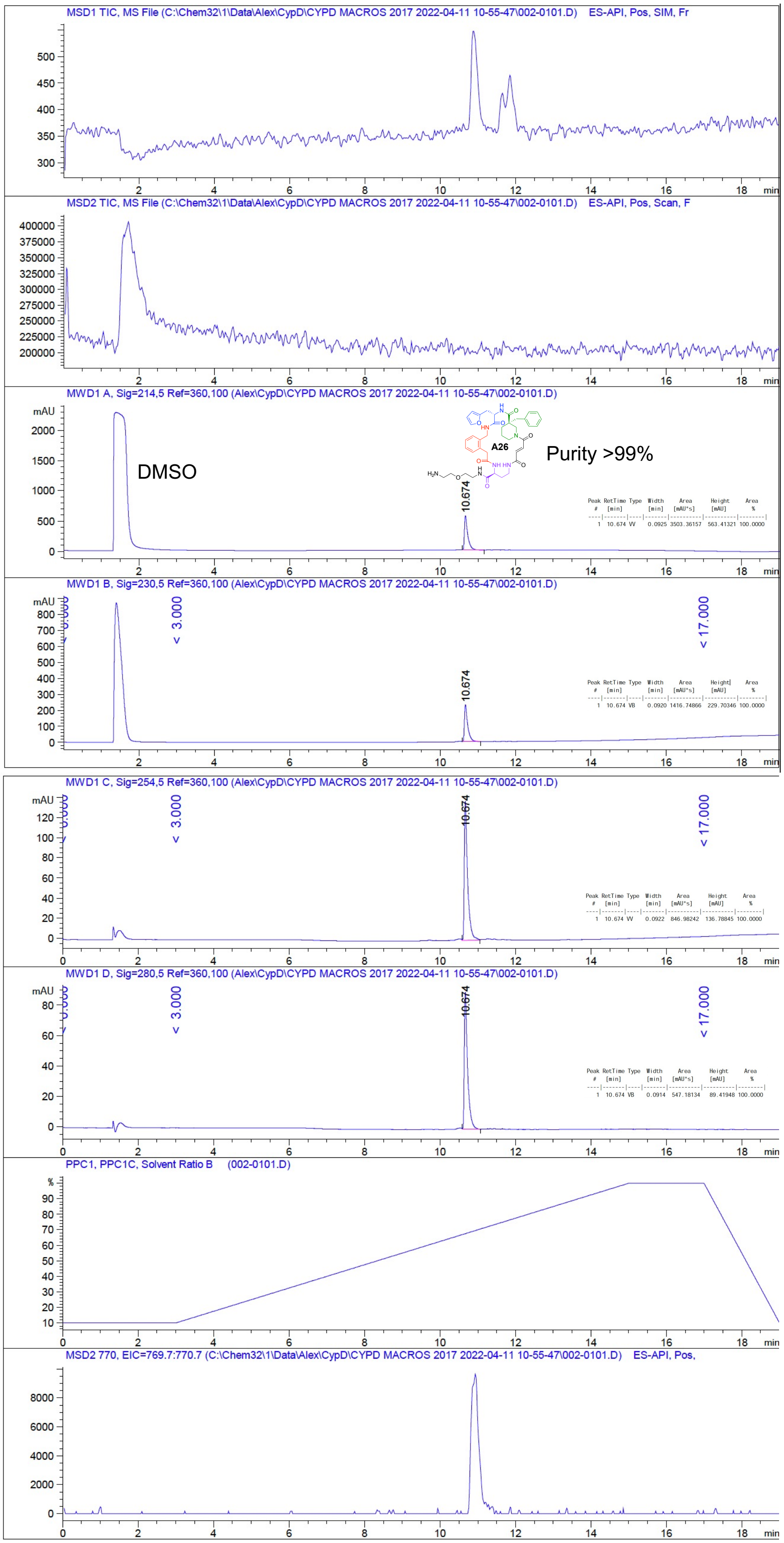
MSD1 TIC, MS File (C:\Chem32\1\Data\Alex\CypD\CYPD MACROS 2017 2022-04-11 10-55-47\002-0101.D) ES-API, Pos, SIM, Fr
MSD2 TIC, MS File (C:\Chem32\1\Data\Alex\CypD\CYPD MACROS 2017 2022-04-11 10-55-47\002-0101.D) ES-API, Pos, Scan, F
MWD1 A, Sig=214,5 Ref=360,100 (Alex\CypD\CYPD MACROS 2017 2022-04-11 10-55-47\002-0101.D)
DMSO
A26
Purity >99%
10.674
Peak # | RetTime [min] | Type | Width [min] | Area [mAU*s] | Height [mAU] | Area %
1 | 10.674 | VV | 0.0925 | 3503.36157 | 563.41321 | 100.0000
MWD1 B, Sig=230,5 Ref=360,100 (Alex\CypD\CYPD MACROS 2017 2022-04-11 10-55-47\002-0101.D)
< 3.000
< 17.000
10.674
Peak # | RetTime [min] | Type | Width [min] | Area [mAU*s] | Height [mAU] | Area %
1 | 10.674 | VB | 0.0920 | 1416.74866 | 229.70346 | 100.0000
MWD1 C, Sig=254,5 Ref=360,100 (Alex\CypD\CYPD MACROS 2017 2022-04-11 10-55-47\002-0101.D)
< 3.000
< 17.000
10.674
Peak # | RetTime [min] | Type | Width [min] | Area [mAU*s] | Height [mAU] | Area %
1 | 10.674 | VV | 0.0922 | 846.98242 | 136.78845 | 100.0000
MWD1 D, Sig=280,5 Ref=360,100 (Alex\CypD\CYPD MACROS 2017 2022-04-11 10-55-47\002-0101.D)
< 3.000
< 17.000
10.674
Peak # | RetTime [min] | Type | Width [min] | Area [mAU*s] | Height [mAU] | Area %
1 | 10.674 | VB | 0.0914 | 547.18134 | 89.41948 | 100.0000
PPC1, PPC1C, Solvent Ratio B (002-0101.D)
MSD2 770, EIC=769.7:770.7 (C:\Chem32\1\Data\Alex\CypD\CYPD MACROS 2017 2022-04-11 10-55-47\002-0101.D) ES-API, Pos,
min

B1

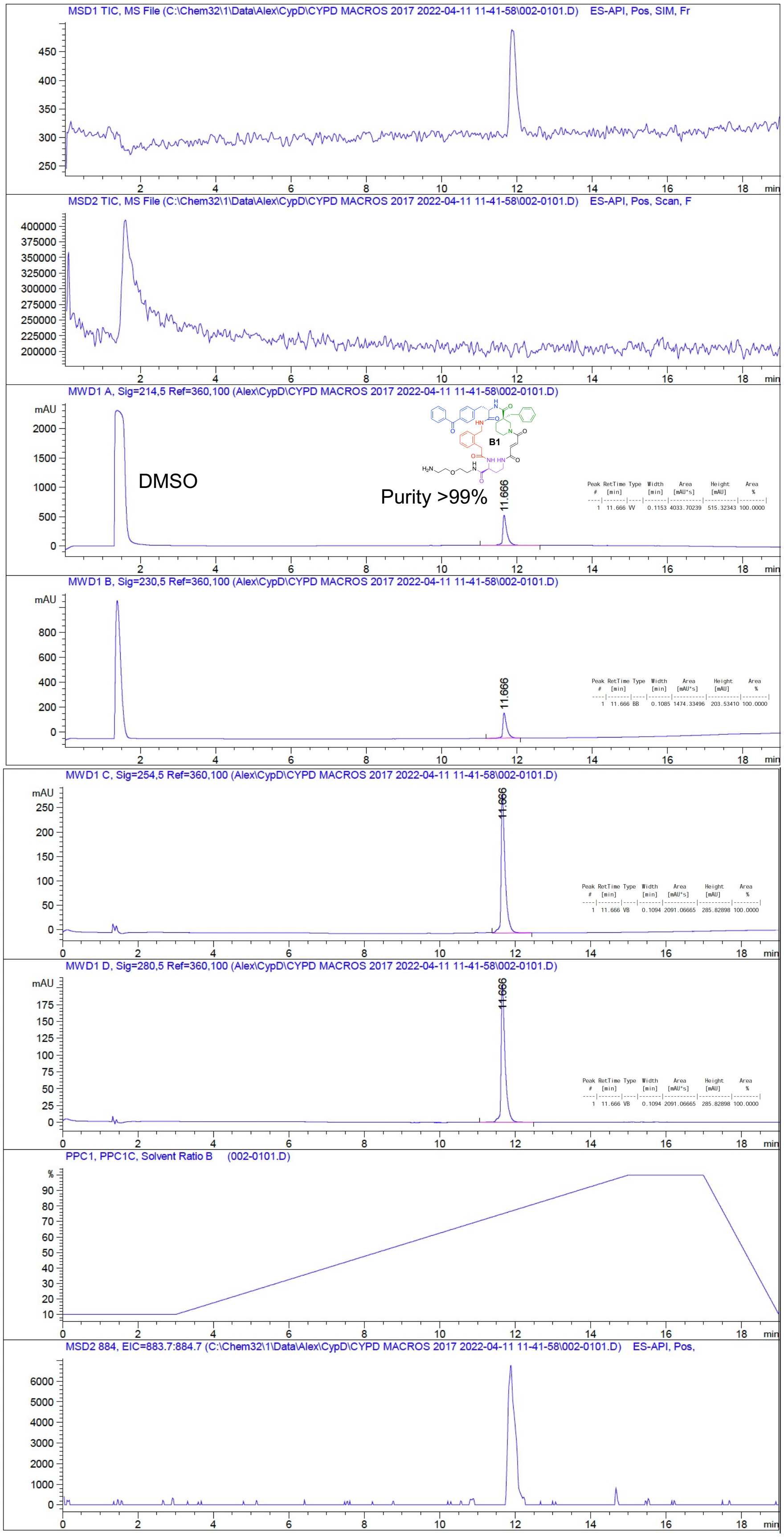
MSD1 TIC, MS File (C:\Chem32\1\Data\Alex\CypD\CYPD MACROS 2017 2022-04-11 11-41-58\002-0101.D) ES-API, Pos, SIM, Fr
MSD2 TIC, MS File (C:\Chem32\1\Data\Alex\CypD\CYPD MACROS 2017 2022-04-11 11-41-58\002-0101.D) ES-API, Pos, Scan, F
MWD1 A, Sig=214,5 Ref=360,100 (Alex\CypD\CYPD MACROS 2017 2022-04-11 11-41-58\002-0101.D)
B1
DMSO
Purity >99%
11.666
MWD1 B, Sig=230,5 Ref=360,100 (Alex\CypD\CYPD MACROS 2017 2022-04-11 11-41-58\002-0101.D)
11.666
MWD1 C, Sig=254,5 Ref=360,100 (Alex\CypD\CYPD MACROS 2017 2022-04-11 11-41-58\002-0101.D)
11.666
MWD1 D, Sig=280,5 Ref=360,100 (Alex\CypD\CYPD MACROS 2017 2022-04-11 11-41-58\002-0101.D)
11.666
PPC1, PPC1C, Solvent Ratio B (002-0101.D)
MSD2 884, EIC=883.7:884.7 (C:\Chem32\1\Data\Alex\CypD\CYPD MACROS 2017 2022-04-11 11-41-58\002-0101.D) ES-API, Pos,
min

B2

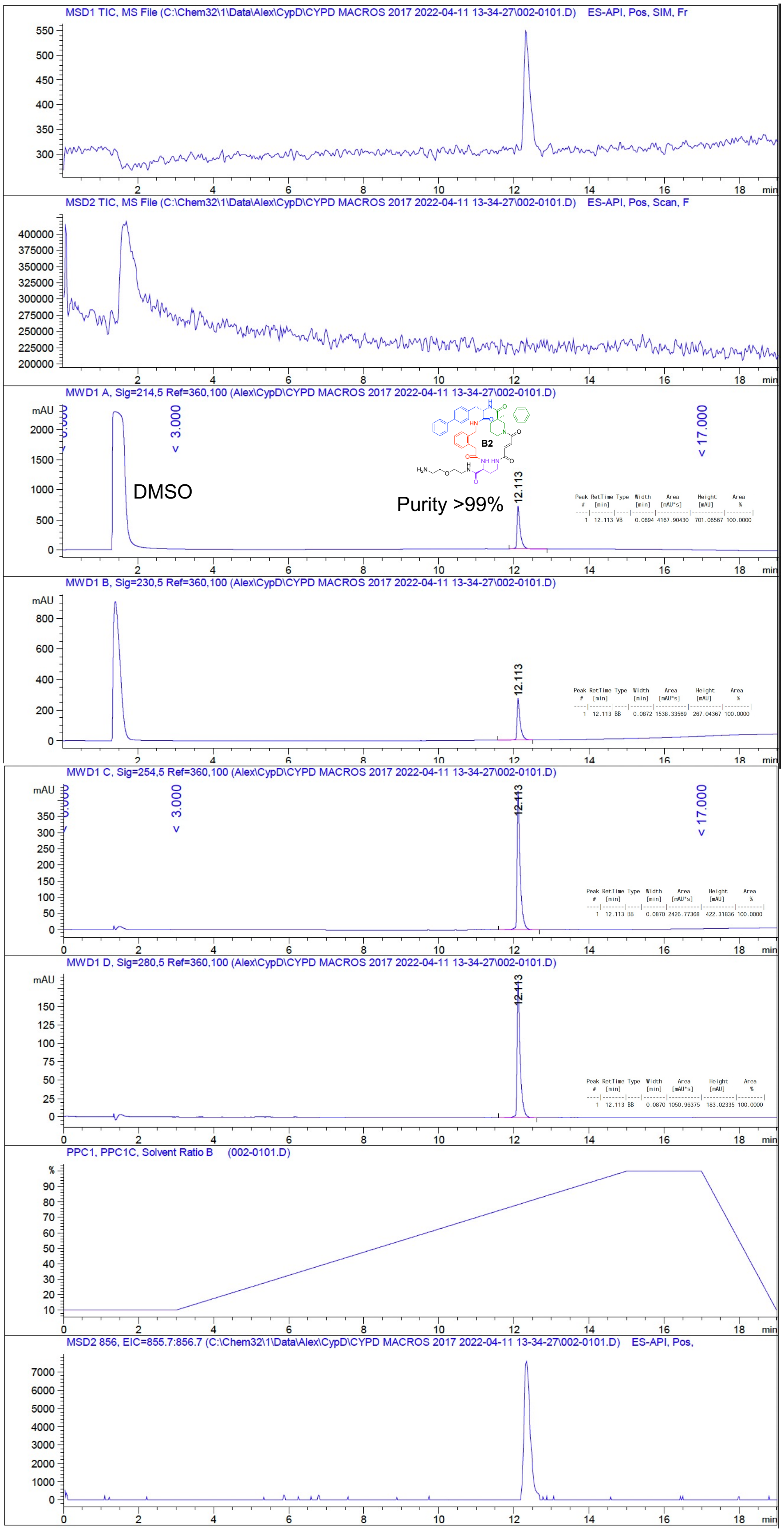
MSD1 TIC, MS File (C:\Chem32\1\Data\Alex\CypD\CYPD MACROS 2017 2022-04-11 13-34-27\002-0101.D) ES-API, Pos, SIM, Fr
MSD2 TIC, MS File (C:\Chem32\1\Data\Alex\CypD\CYPD MACROS 2017 2022-04-11 13-34-27\002-0101.D) ES-API, Pos, Scan, F
MWD1 A, Sig=214,5 Ref=360,100 (Alex\CypD\CYPD MACROS 2017 2022-04-11 13-34-27\002-0101.D)
B2
DMSO
Purity >99%
12.113
Peak # | RetTime [min] | Type | Width [min] | Area [mAU*s] | Height [mAU] | Area %
1 | 12.113 | VB | 0.0894 | 4167.90430 | 701.06567 | 100.0000
MWD1 B, Sig=230,5 Ref=360,100 (Alex\CypD\CYPD MACROS 2017 2022-04-11 13-34-27\002-0101.D)
Peak # | RetTime [min] | Type | Width [min] | Area [mAU*s] | Height [mAU] | Area %
1 | 12.113 | BB | 0.0872 | 1538.33569 | 267.04367 | 100.0000
MWD1 C, Sig=254,5 Ref=360,100 (Alex\CypD\CYPD MACROS 2017 2022-04-11 13-34-27\002-0101.D)
Peak # | RetTime [min] | Type | Width [min] | Area [mAU*s] | Height [mAU] | Area %
1 | 12.113 | BB | 0.0870 | 2426.77368 | 422.31836 | 100.0000
MWD1 D, Sig=280,5 Ref=360,100 (Alex\CypD\CYPD MACROS 2017 2022-04-11 13-34-27\002-0101.D)
Peak # | RetTime [min] | Type | Width [min] | Area [mAU*s] | Height [mAU] | Area %
1 | 12.113 | BB | 0.0870 | 1050.96375 | 183.02335 | 100.0000
PPC1, PPC1C, Solvent Ratio B (002-0101.D)
MSD2 856, EIC=855.7:856.7 (C:\Chem32\1\Data\Alex\CypD\CYPD MACROS 2017 2022-04-11 13-34-27\002-0101.D) ES-API, Pos,

B23

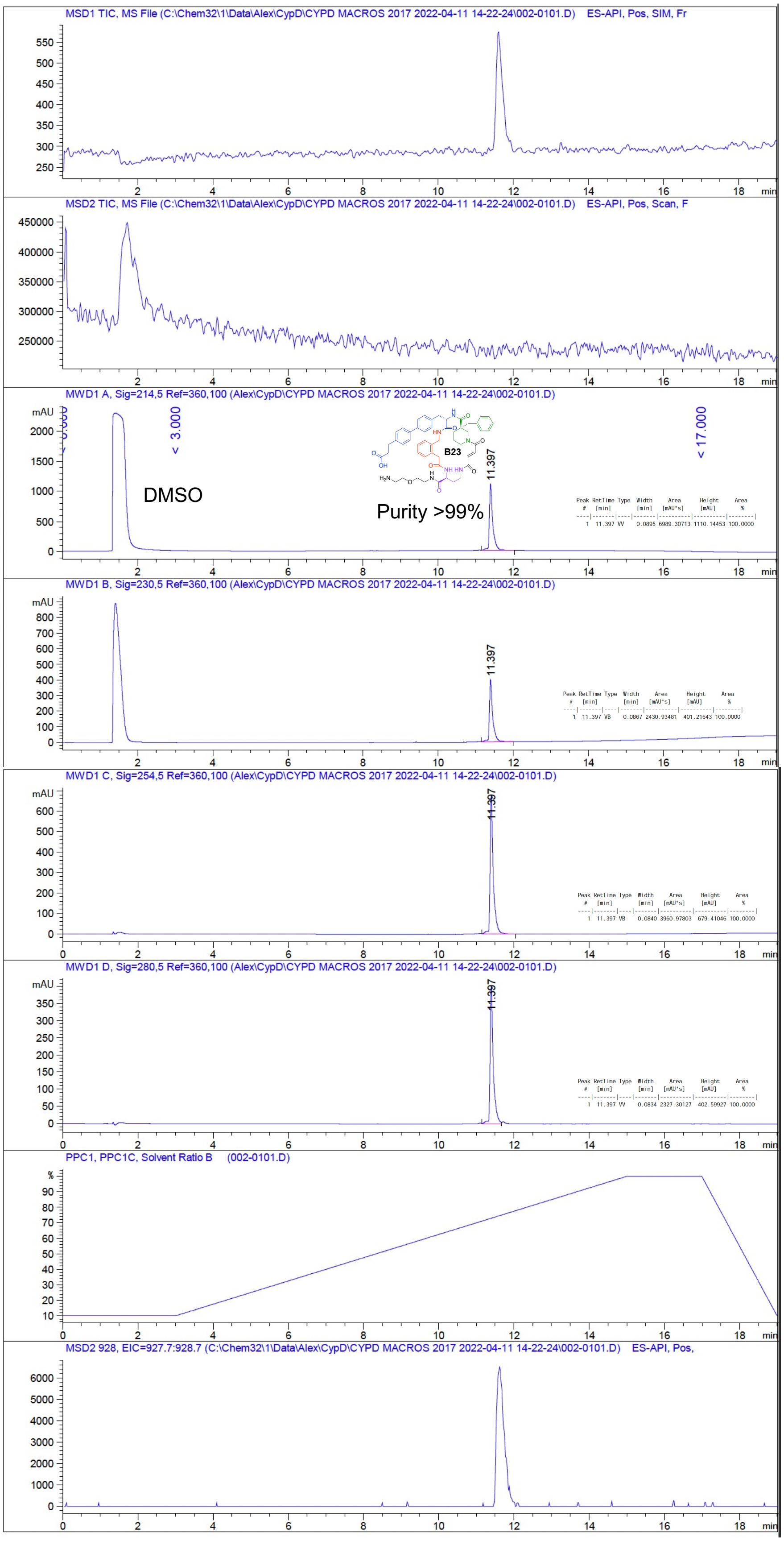
MSD1 TIC, MS File (C:\Chem32\1\Data\Alex\CypD\CYPD MACROS 2017 2022-04-11 14-22-24\002-0101.D) ES-API, Pos, SIM, Fr
MSD2 TIC, MS File (C:\Chem32\1\Data\Alex\CypD\CYPD MACROS 2017 2022-04-11 14-22-24\002-0101.D) ES-API, Pos, Scan, F
MWD1 A, Sig=214,5 Ref=360,100 (Alex\CypD\CYPD MACROS 2017 2022-04-11 14-22-24\002-0101.D)
< 3.000
< 17.000
DMSO
B23
Purity >99%
11.397
Peak RetTime Type Width Area Height Area
1 11.397 VV 0.0895 6989.30713 1110.14453 100.0000
MWD1 B, Sig=230,5 Ref=360,100 (Alex\CypD\CYPD MACROS 2017 2022-04-11 14-22-24\002-0101.D)
11.397
Peak RetTime Type Width Area Height Area
1 11.397 VB 0.0867 2430.93481 401.21643 100.0000
MWD1 C, Sig=254,5 Ref=360,100 (Alex\CypD\CYPD MACROS 2017 2022-04-11 14-22-24\002-0101.D)
11.397
Peak RetTime Type Width Area Height Area
1 11.397 VB 0.0840 3960.97803 679.41046 100.0000
MWD1 D, Sig=280,5 Ref=360,100 (Alex\CypD\CYPD MACROS 2017 2022-04-11 14-22-24\002-0101.D)
11.397
Peak RetTime Type Width Area Height Area
1 11.397 VV 0.0834 2327.30127 402.59927 100.0000
PPC1, PPC1C, Solvent Ratio B (002-0101.D)
MSD2 928, EIC=927.7:928.7 (C:\Chem32\1\Data\Alex\CypD\CYPD MACROS 2017 2022-04-11 14-22-24\002-0101.D) ES-API, Pos,

B25

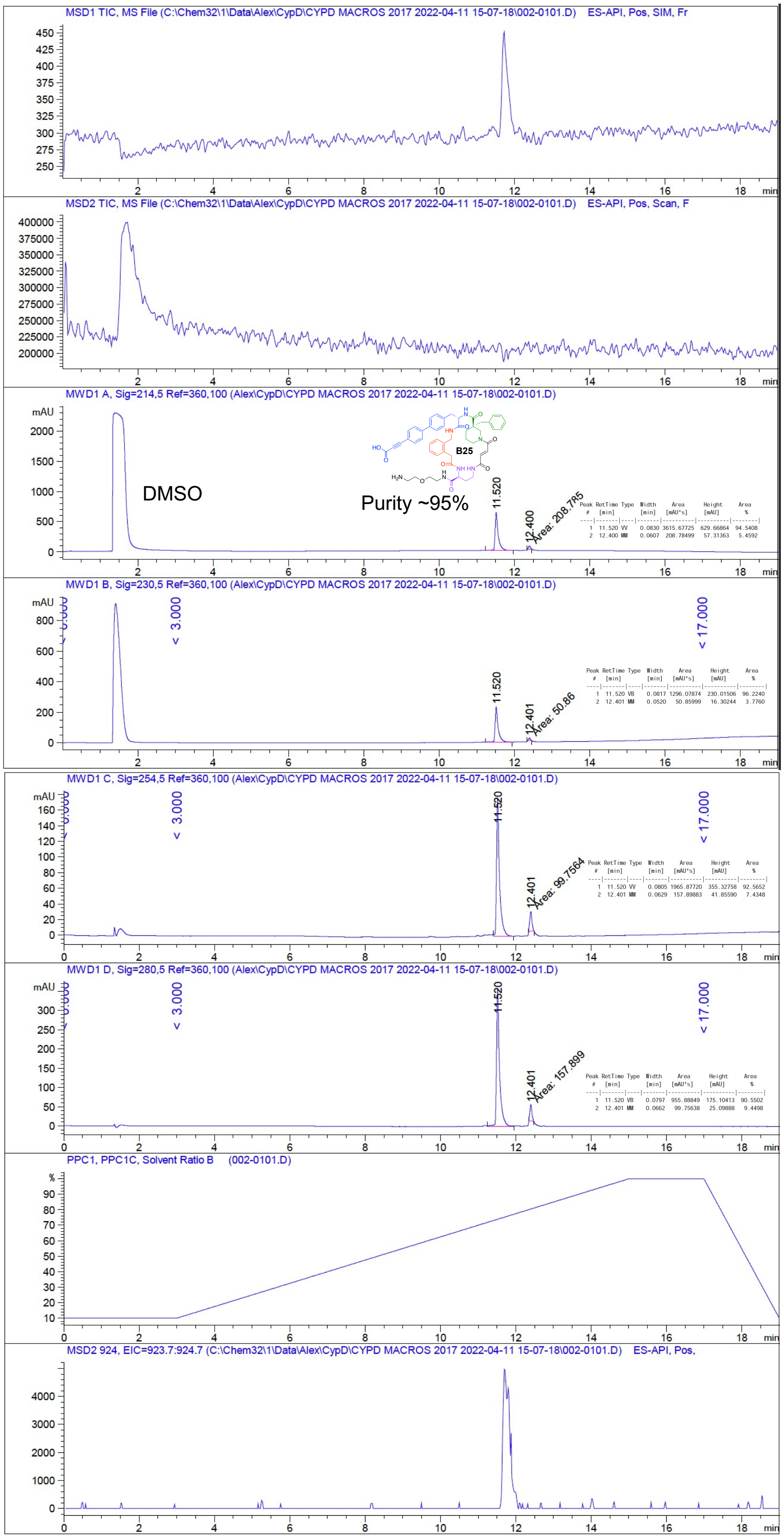
MSD1 TIC, MS File (C:\Chem32\1\Data\Alex\CypD\CYPD MACROS 2017 2022-04-11 15-07-18\002-0101.D) ES-API, Pos, SIM, Fr
MSD2 TIC, MS File (C:\Chem32\1\Data\Alex\CypD\CYPD MACROS 2017 2022-04-11 15-07-18\002-0101.D) ES-API, Pos, Scan, F
MWD1 A, Sig=214,5 Ref=360,100 (Alex\CypD\CYPD MACROS 2017 2022-04-11 15-07-18\002-0101.D)
DMSO
B25
Purity ~95%
11.520
12.400
Area: 208.785
Peak RetTime Type Width Area Height Area
[min] [min] [mAU*s] [mAU] %
1 11.520 VV 0.0830 3615.67725 629.66864 94.5408
2 12.400 MM 0.0607 208.78499 57.31363 5.4592
MWD1 B, Sig=230,5 Ref=360,100 (Alex\CypD\CYPD MACROS 2017 2022-04-11 15-07-18\002-0101.D)
< 3.000
< 17.000
11.520
12.401
Area: 50.86
Peak RetTime Type Width Area Height Area
[min] [min] [mAU*s] [mAU] %
1 11.520 VB 0.0817 1296.07874 230.01506 96.2240
2 12.401 MM 0.0520 50.85999 16.30244 3.7760
MWD1 C, Sig=254,5 Ref=360,100 (Alex\CypD\CYPD MACROS 2017 2022-04-11 15-07-18\002-0101.D)
< 3.000
< 17.000
11.520
12.401
Area: 99.7564
Peak RetTime Type Width Area Height Area
[min] [min] [mAU*s] [mAU] %
1 11.520 VV 0.0805 1965.87720 355.32758 92.5652
2 12.401 MM 0.0629 157.89883 41.85590 7.4348
MWD1 D, Sig=280,5 Ref=360,100 (Alex\CypD\CYPD MACROS 2017 2022-04-11 15-07-18\002-0101.D)
< 3.000
< 17.000
11.520
12.401
Area: 157.899
Peak RetTime Type Width Area Height Area
[min] [min] [mAU*s] [mAU] %
1 11.520 VB 0.0797 955.88849 175.10413 90.5502
2 12.401 MM 0.0662 99.75638 25.09888 9.4498
PPC1, PPC1C, Solvent Ratio B (002-0101.D)
MSD2 924, EIC=923.7:924.7 (C:\Chem32\1\Data\Alex\CypD\CYPD MACROS 2017 2022-04-11 15-07-18\002-0101.D) ES-API, Pos,
min

B32

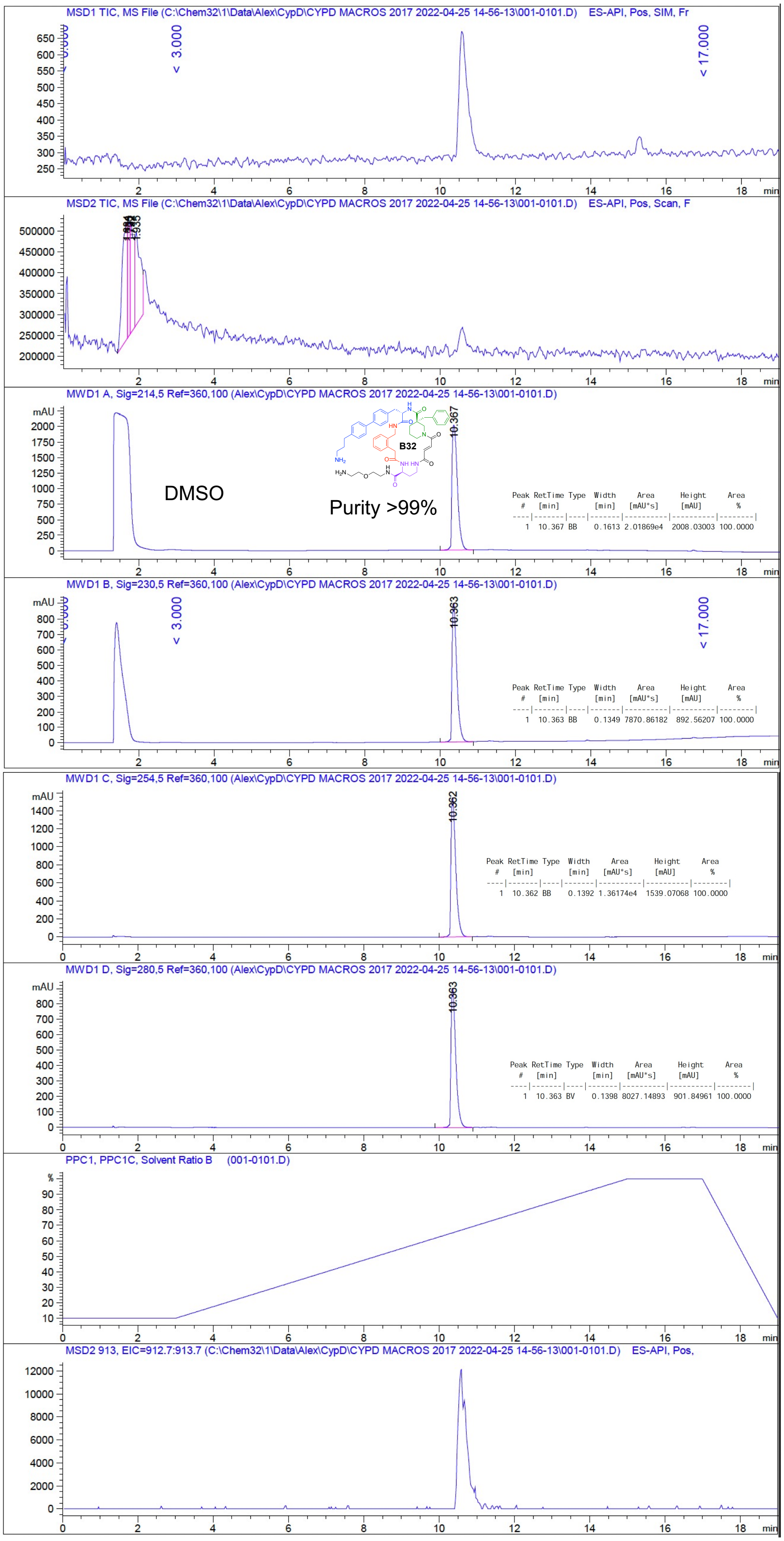

MSD1 TIC, MS File (C:\Chem32\1\Data\Alex\CypD\CYPD MACROS 2017 2022-04-25 14-56-13\001-0101.D) ES-API, Pos, SIM, Fr
MSD2 TIC, MS File (C:\Chem32\1\Data\Alex\CypD\CYPD MACROS 2017 2022-04-25 14-56-13\001-0101.D) ES-API, Pos, Scan, F
MWD1 A, Sig=214,5 Ref=360,100 (Alex\CypD\CYPD MACROS 2017 2022-04-25 14-56-13\001-0101.D)
DMSO
B32
Purity >99%
Peak # | RetTime [min] | Type | Width [min] | Area [mAU*s] | Height [mAU] | Area %
1 | 10.367 | BB | 0.1613 | 2.01869e4 | 2008.03003 | 100.0000
MWD1 B, Sig=230,5 Ref=360,100 (Alex\CypD\CYPD MACROS 2017 2022-04-25 14-56-13\001-0101.D)
1 | 10.363 | BB | 0.1349 | 7870.86182 | 892.56207 | 100.0000
MWD1 C, Sig=254,5 Ref=360,100 (Alex\CypD\CYPD MACROS 2017 2022-04-25 14-56-13\001-0101.D)
1 | 10.362 | BB | 0.1392 | 1.36174e4 | 1539.07068 | 100.0000
MWD1 D, Sig=280,5 Ref=360,100 (Alex\CypD\CYPD MACROS 2017 2022-04-25 14-56-13\001-0101.D)
1 | 10.363 | BV | 0.1398 | 8027.14893 | 901.84961 | 100.0000
PPC1, PPC1C, Solvent Ratio B (001-0101.D)
MSD2 913, EIC=912.7:913.7 (C:\Chem32\1\Data\Alex\CypD\CYPD MACROS 2017 2022-04-25 14-56-13\001-0101.D) ES-API, Pos,

B52 (used for independent replicate 1)

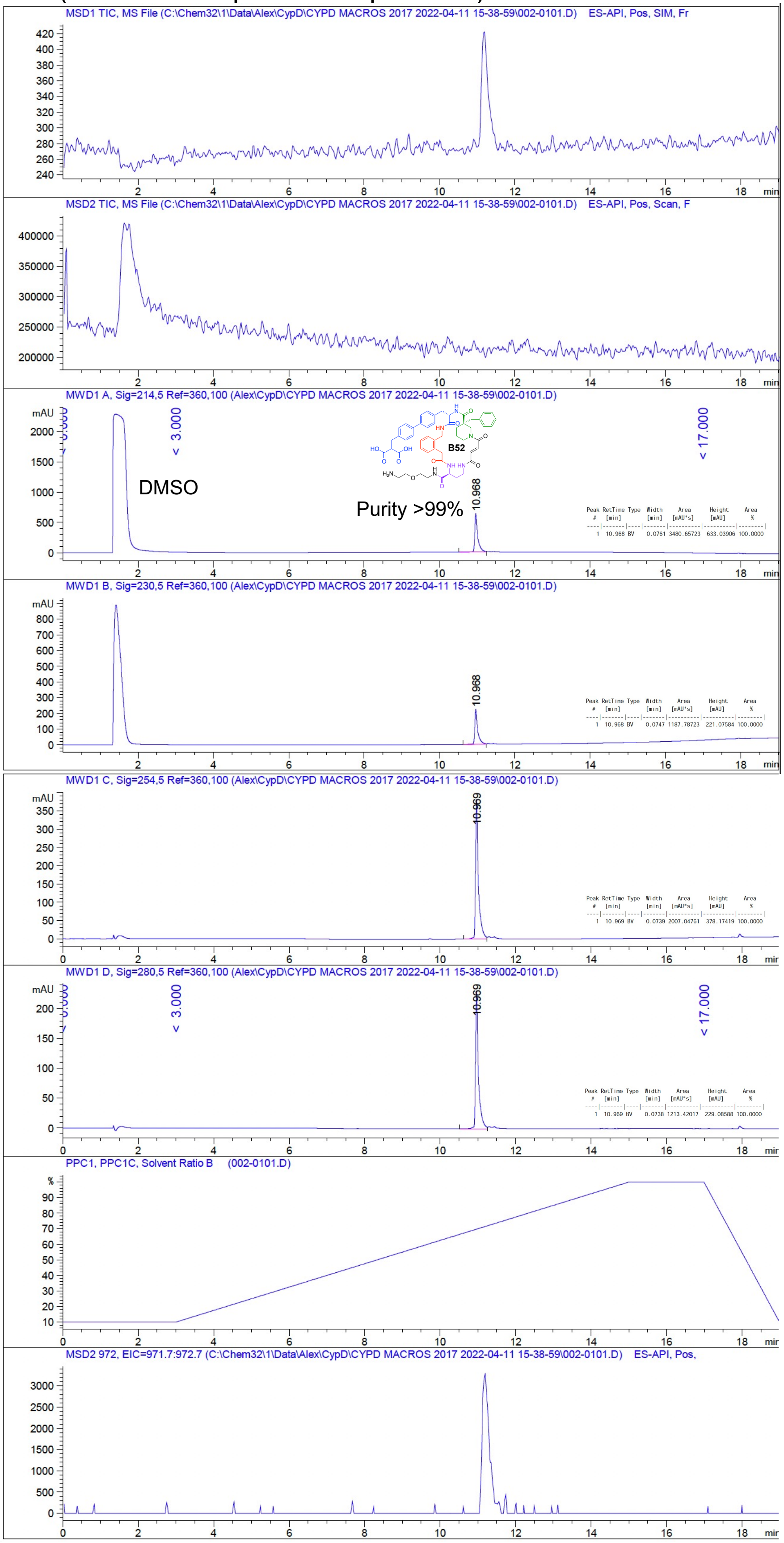

B52 (used for independent replicate 2, 3, and 4)

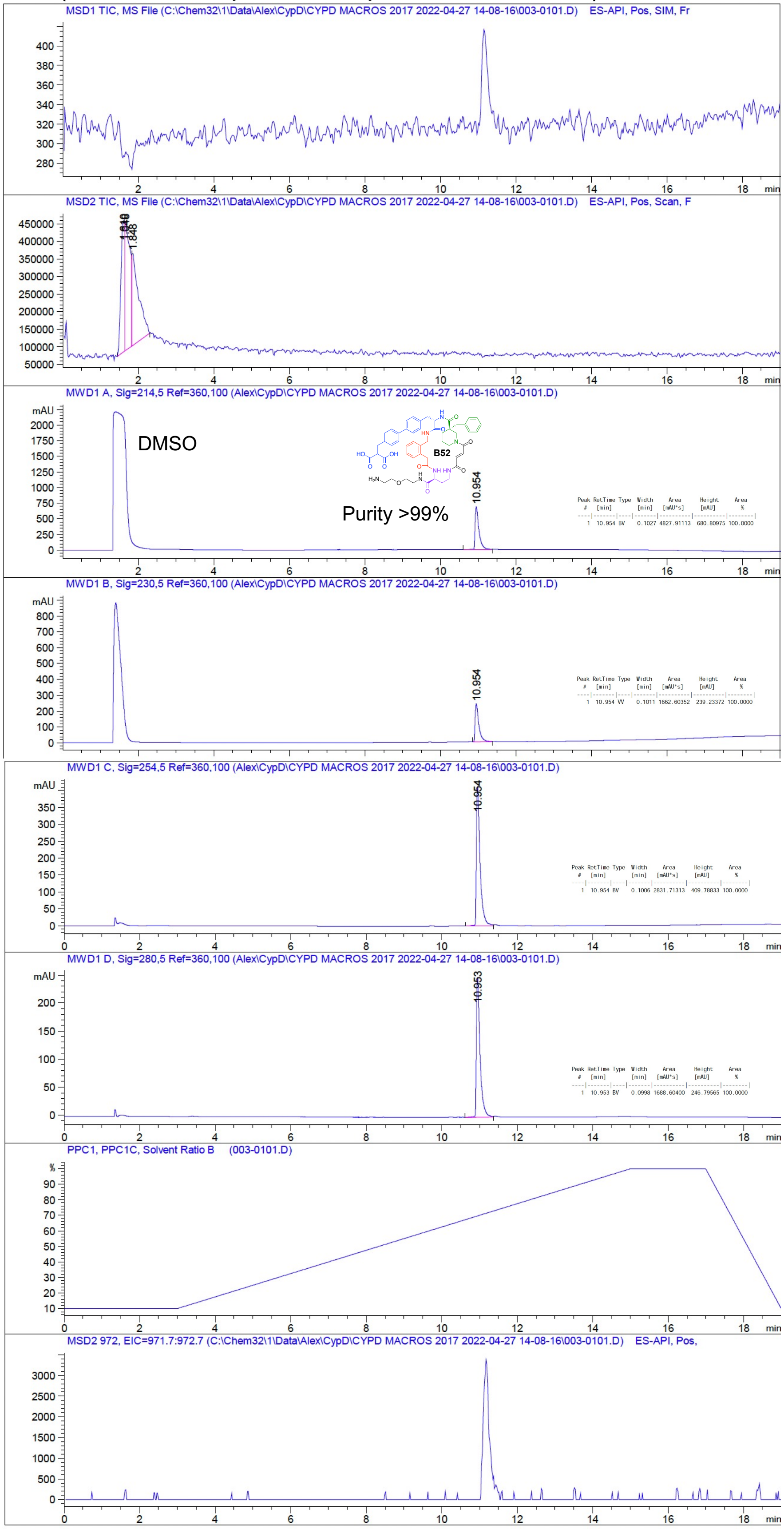

B53 (used for independent replicate 1)

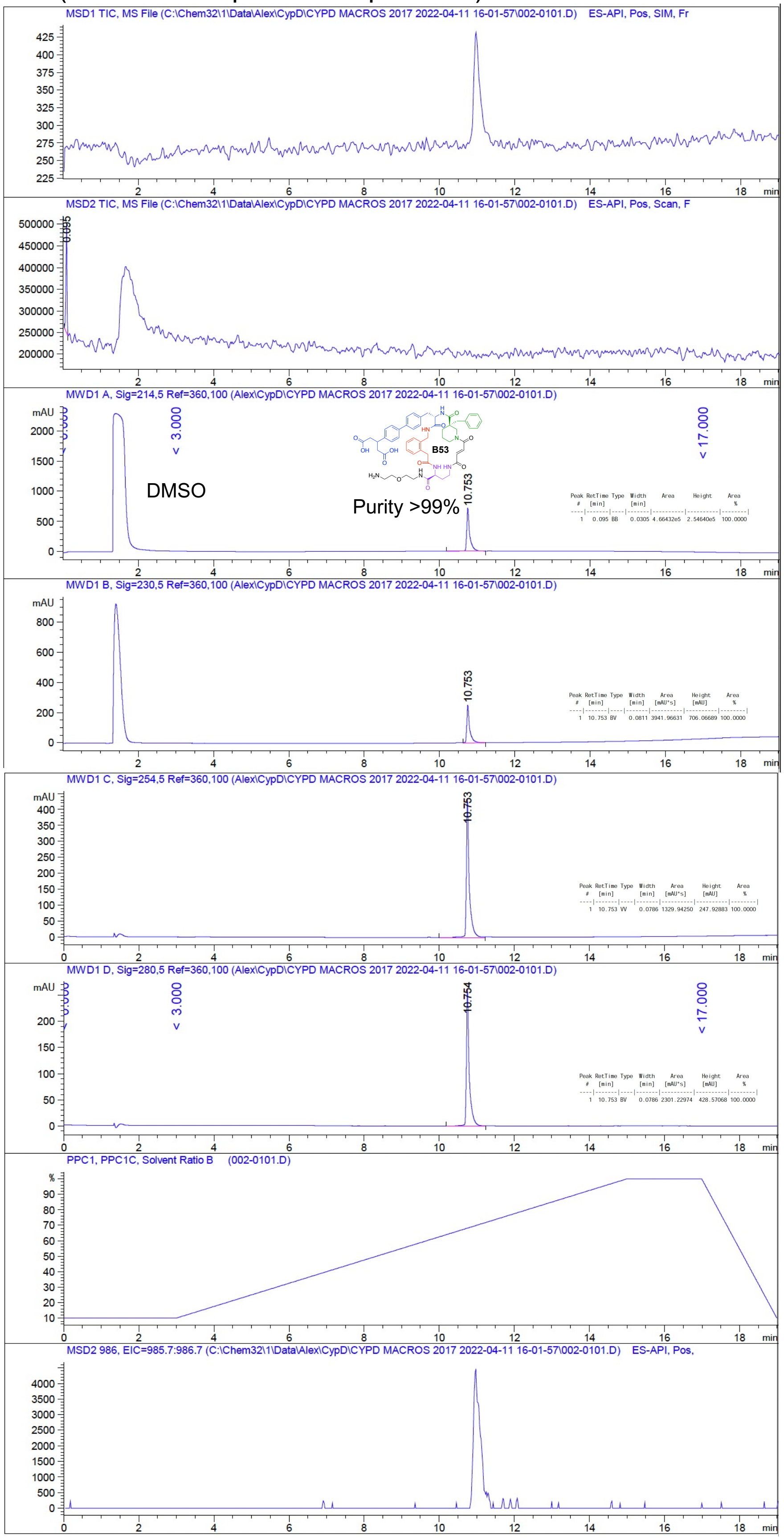

B53 (used for independent replicate 2, 3 and 4)

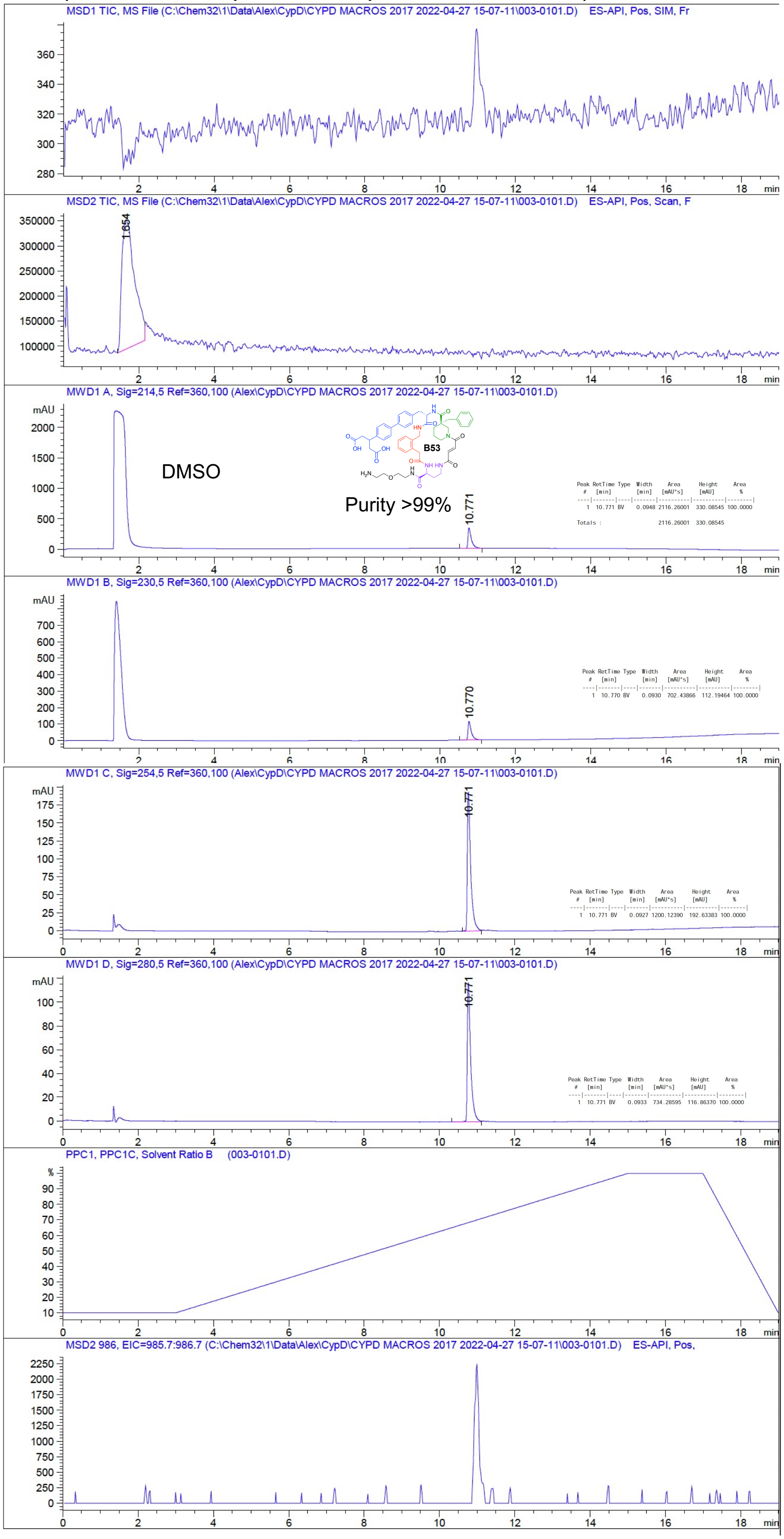

C3A (used for independent replicate 1)

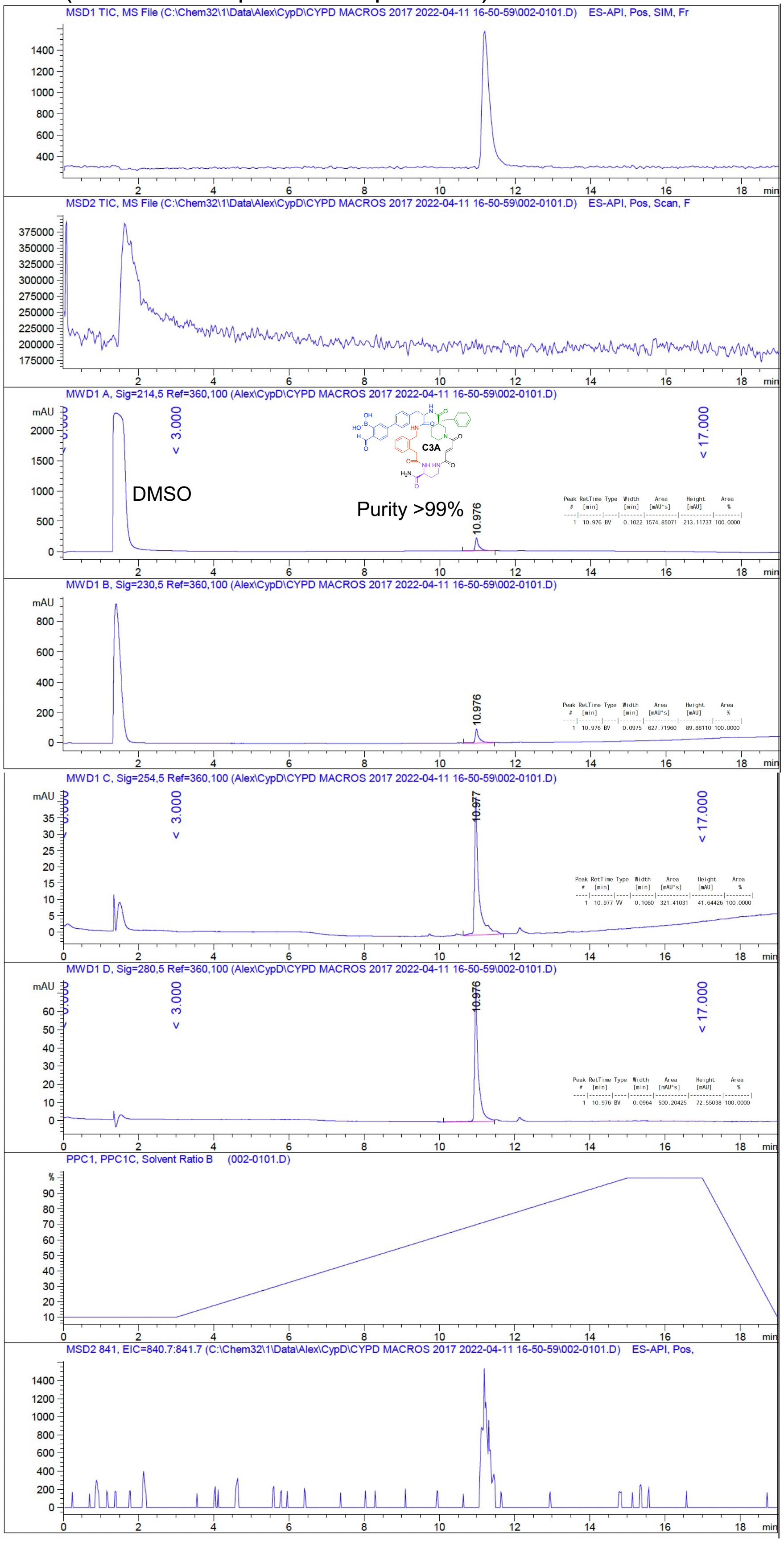

C3A (used for independent replicate 2, 3 and 4)

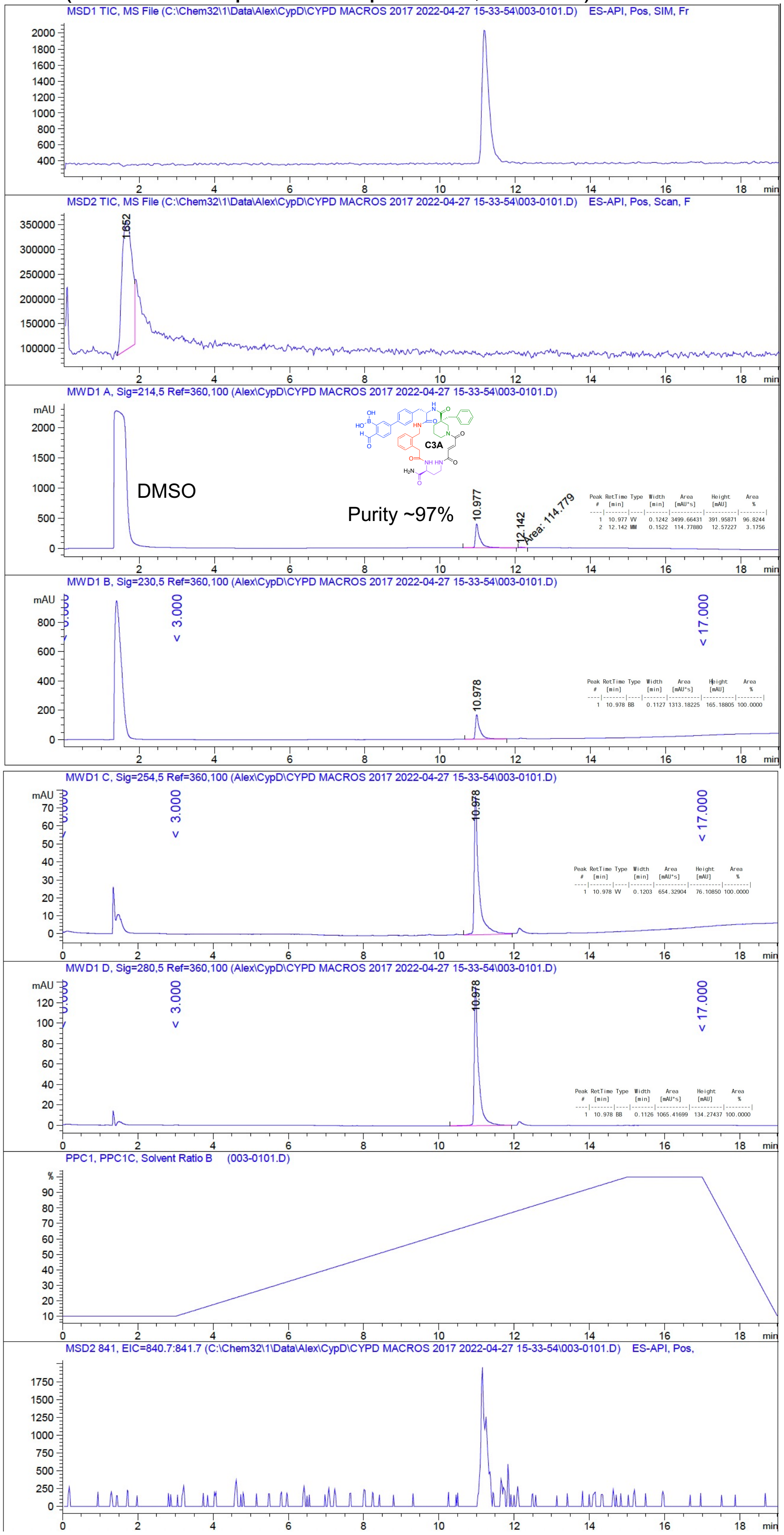

B52-Cy5

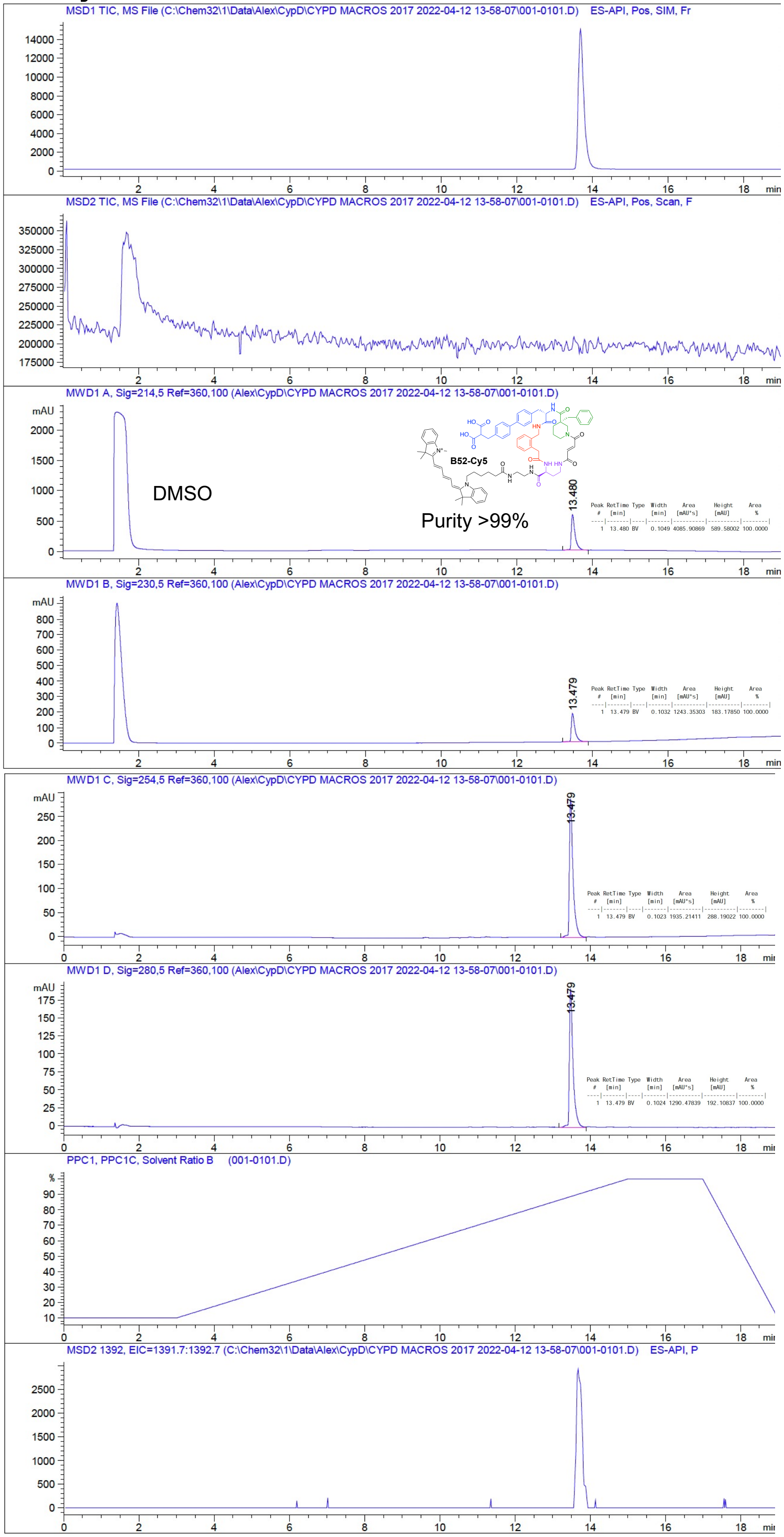
MSD1 TIC, MS File (C:\Chem32\1\Data\Alex\CypD\CYPD MACROS 2017 2022-04-12 13-58-07\001-0101.D) ES-API, Pos, SIM, Fr
MSD2 TIC, MS File (C:\Chem32\1\Data\Alex\CypD\CYPD MACROS 2017 2022-04-12 13-58-07\001-0101.D) ES-API, Pos, Scan, F
MWD1 A, Sig=214,5 Ref=360,100 (Alex\CypD\CYPD MACROS 2017 2022-04-12 13-58-07\001-0101.D)
DMSO
B52-Cy5
Purity >99%
13.480
Peak # | RetTime [min] | Type | Width [min] | Area [mAU*s] | Height [mAU] | Area %
1 | 13.480 | BV | 0.1049 | 4085.90869 | 589.58002 | 100.0000
MWD1 B, Sig=230,5 Ref=360,100 (Alex\CypD\CYPD MACROS 2017 2022-04-12 13-58-07\001-0101.D)
13.479
1 | 13.479 | BV | 0.1032 | 1243.35303 | 183.17850 | 100.0000
MWD1 C, Sig=254,5 Ref=360,100 (Alex\CypD\CYPD MACROS 2017 2022-04-12 13-58-07\001-0101.D)
13.479
1 | 13.479 | BV | 0.1023 | 1935.21411 | 288.19022 | 100.0000
MWD1 D, Sig=280,5 Ref=360,100 (Alex\CypD\CYPD MACROS 2017 2022-04-12 13-58-07\001-0101.D)
13.479
1 | 13.479 | BV | 0.1024 | 1290.47839 | 192.10837 | 100.0000
PPC1, PPC1C, Solvent Ratio B (001-0101.D)
MSD2 1392, EIC=1391.7:1392.7 (C:\Chem32\1\Data\Alex\CypD\CYPD MACROS 2017 2022-04-12 13-58-07\001-0101.D) ES-API, P
min

B53-Cy5

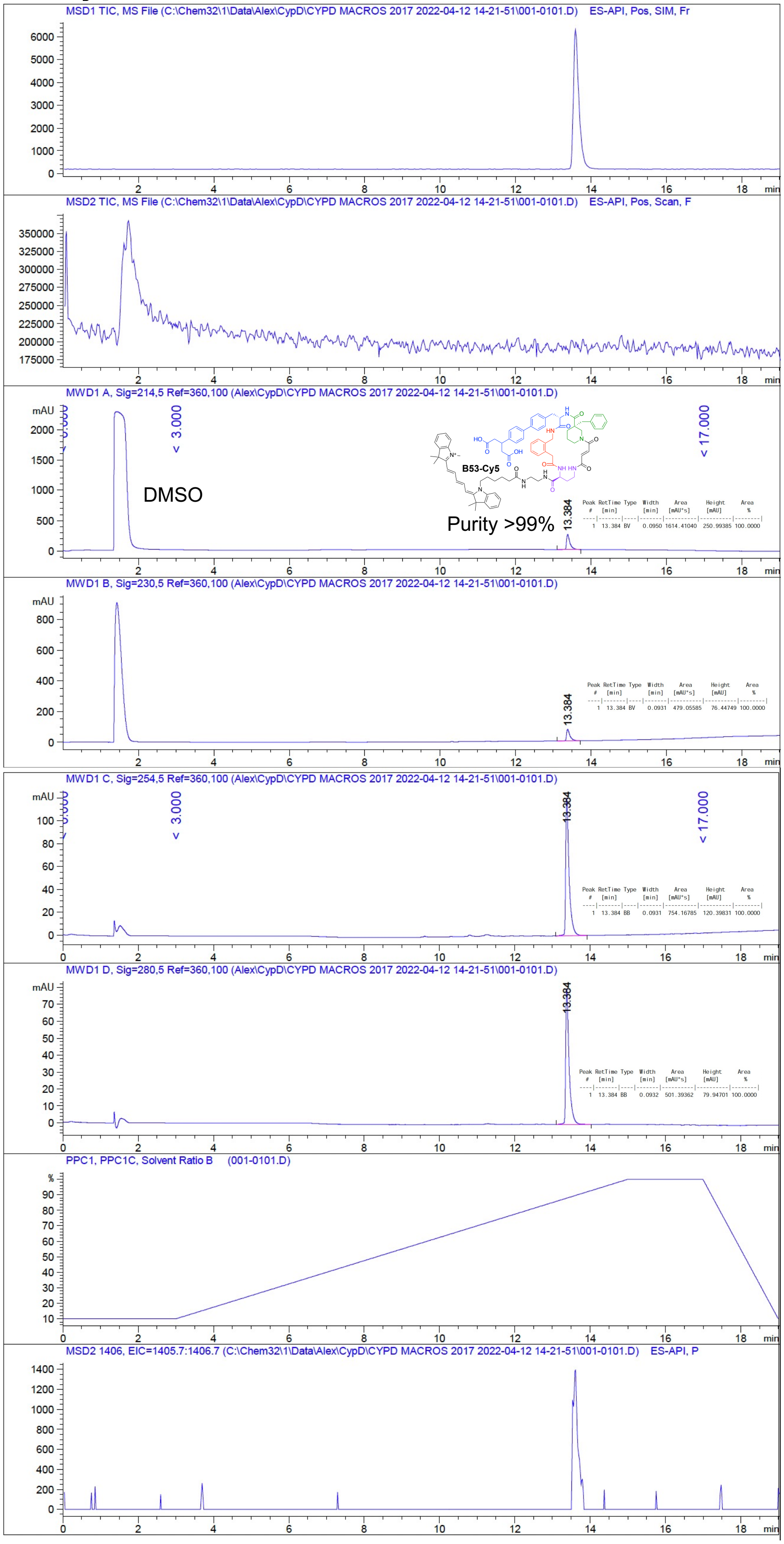
MSD1 TIC, MS File (C:\Chem32\1\Data\Alex\CypD\CYPD MACROS 2017 2022-04-12 14-21-51\001-0101.D) ES-API, Pos, SIM, Fr
MSD2 TIC, MS File (C:\Chem32\1\Data\Alex\CypD\CYPD MACROS 2017 2022-04-12 14-21-51\001-0101.D) ES-API, Pos, Scan, F
MWD1 A, Sig=214,5 Ref=360,100 (Alex\CypD\CYPD MACROS 2017 2022-04-12 14-21-51\001-0101.D)
DMSO
B53-Cy5
Purity >99%
13.384
MWD1 B, Sig=230,5 Ref=360,100 (Alex\CypD\CYPD MACROS 2017 2022-04-12 14-21-51\001-0101.D)
MWD1 C, Sig=254,5 Ref=360,100 (Alex\CypD\CYPD MACROS 2017 2022-04-12 14-21-51\001-0101.D)
MWD1 D, Sig=280,5 Ref=360,100 (Alex\CypD\CYPD MACROS 2017 2022-04-12 14-21-51\001-0101.D)
PPC1, PPC1C, Solvent Ratio B (001-0101.D)
MSD2 1406, EIC=1405.7:1406.7 (C:\Chem32\1\Data\Alex\CypD\CYPD MACROS 2017 2022-04-12 14-21-51\001-0101.D) ES-API, P
min

*B52-Cy5

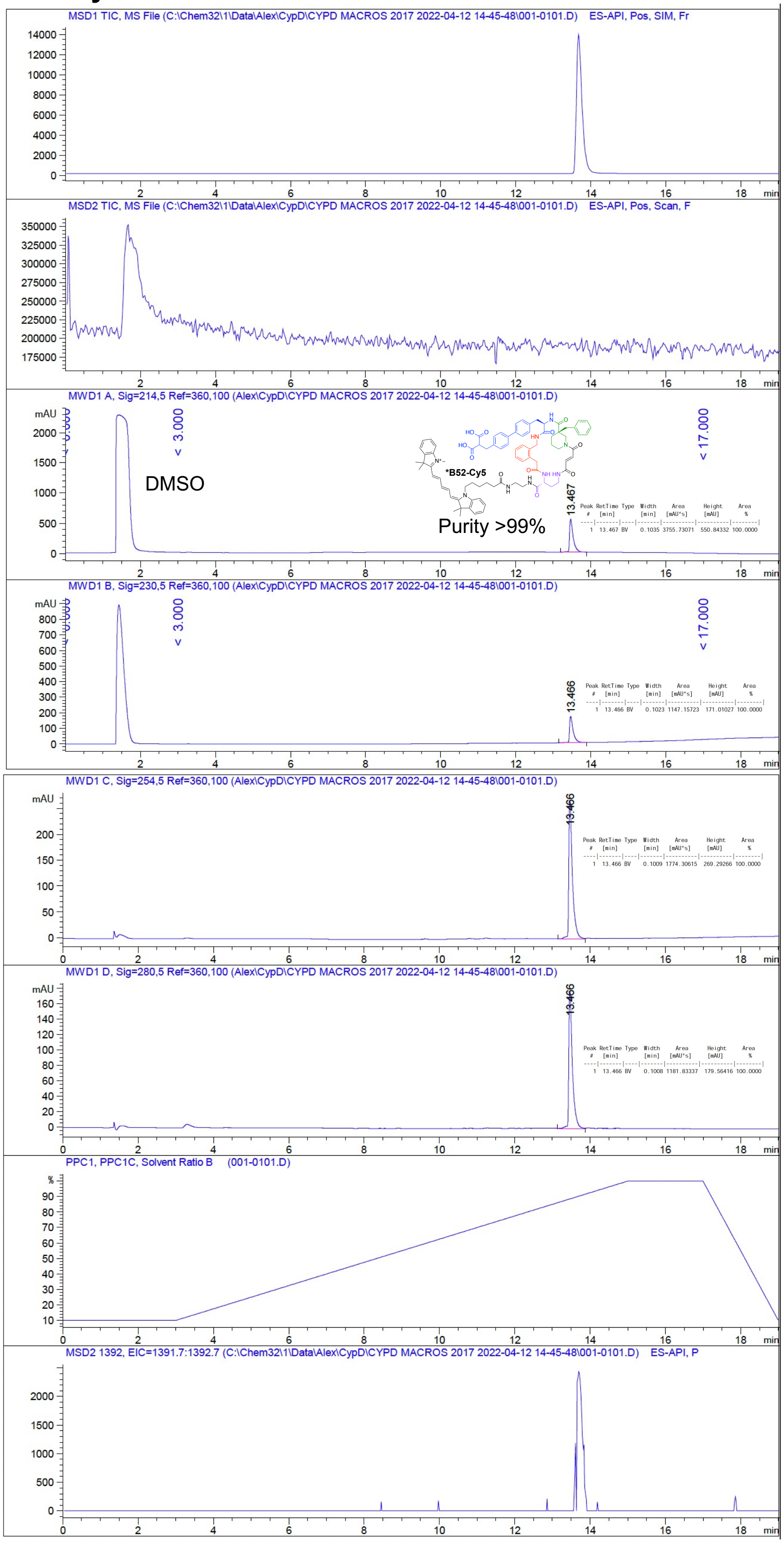

MSD1 TIC, MS File (C:\Chem32\1\Data\Alex\CypD\CYPD MACROS 2017 2022-04-12 14-45-48\001-0101.D) ES-API, Pos, SIM, Fr
MSD2 TIC, MS File (C:\Chem32\1\Data\Alex\CypD\CYPD MACROS 2017 2022-04-12 14-45-48\001-0101.D) ES-API, Pos, Scan, F
MWD1 A, Sig=214,5 Ref=360,100 (Alex\CypD\CYPD MACROS 2017 2022-04-12 14-45-48\001-0101.D)
DMSO
*B52-Cy5
Purity >99%
13.467
MWD1 B, Sig=230,5 Ref=360,100 (Alex\CypD\CYPD MACROS 2017 2022-04-12 14-45-48\001-0101.D)
13.466
MWD1 C, Sig=254,5 Ref=360,100 (Alex\CypD\CYPD MACROS 2017 2022-04-12 14-45-48\001-0101.D)
13.466
MWD1 D, Sig=280,5 Ref=360,100 (Alex\CypD\CYPD MACROS 2017 2022-04-12 14-45-48\001-0101.D)
13.466
PPC1, PPC1C, Solvent Ratio B (001-0101.D)
MSD2 1392, EIC=1391.7:1392.7 (C:\Chem32\1\Data\Alex\CypD\CYPD MACROS 2017 2022-04-12 14-45-48\001-0101.D) ES-API, P
min

*B53-Cy5

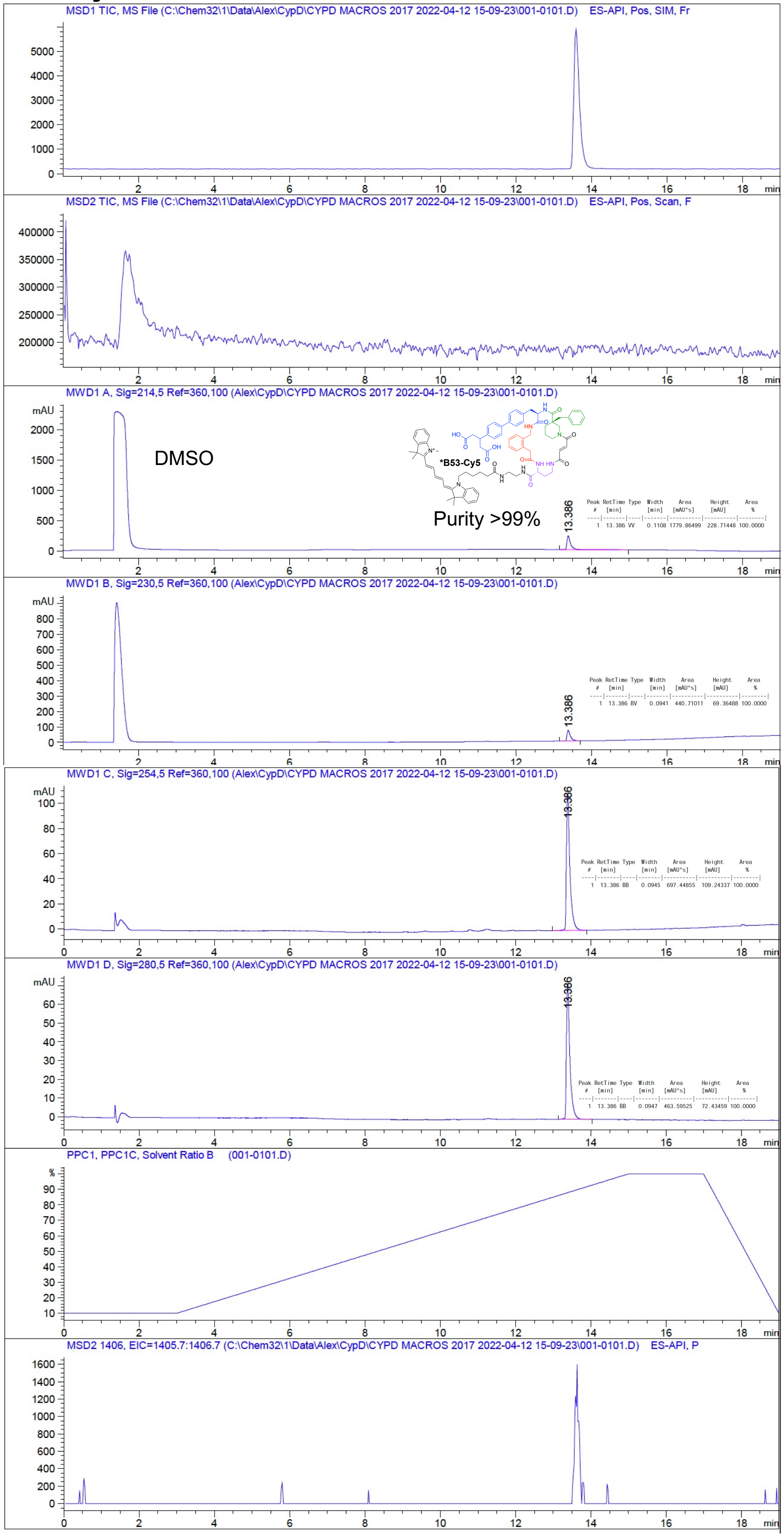
MSD1 TIC, MS File (C:\Chem32\1\Data\Alex\CypD\CYPD MACROS 2017 2022-04-12 15-09-23\001-0101.D) ES-API, Pos, SIM, Fr
MSD2 TIC, MS File (C:\Chem32\1\Data\Alex\CypD\CYPD MACROS 2017 2022-04-12 15-09-23\001-0101.D) ES-API, Pos, Scan, F
MWD1 A, Sig=214,5 Ref=360,100 (Alex\CypD\CYPD MACROS 2017 2022-04-12 15-09-23\001-0101.D)
DMSO
*B53-Cy5
Purity >99%
13.386
Peak RetTime Type Width Area Height Area
1 13.386 VV 0.1108 1779.86499 228.71448 100.0000
MWD1 B, Sig=230,5 Ref=360,100 (Alex\CypD\CYPD MACROS 2017 2022-04-12 15-09-23\001-0101.D)
13.386
1 13.386 BV 0.0941 440.71011 69.36488 100.0000
MWD1 C, Sig=254,5 Ref=360,100 (Alex\CypD\CYPD MACROS 2017 2022-04-12 15-09-23\001-0101.D)
13.386
1 13.386 BB 0.0945 697.44855 109.24337 100.0000
MWD1 D, Sig=280,5 Ref=360,100 (Alex\CypD\CYPD MACROS 2017 2022-04-12 15-09-23\001-0101.D)
13.386
1 13.386 BB 0.0947 463.59525 72.43459 100.0000
PPC1, PPC1C, Solvent Ratio B (001-0101.D)
MSD2 1406, EIC=1405.7:1406.7 (C:\Chem32\1\Data\Alex\CypD\CYPD MACROS 2017 2022-04-12 15-09-23\001-0101.D) ES-API, P
min
mAU

B52-Et-Cy5

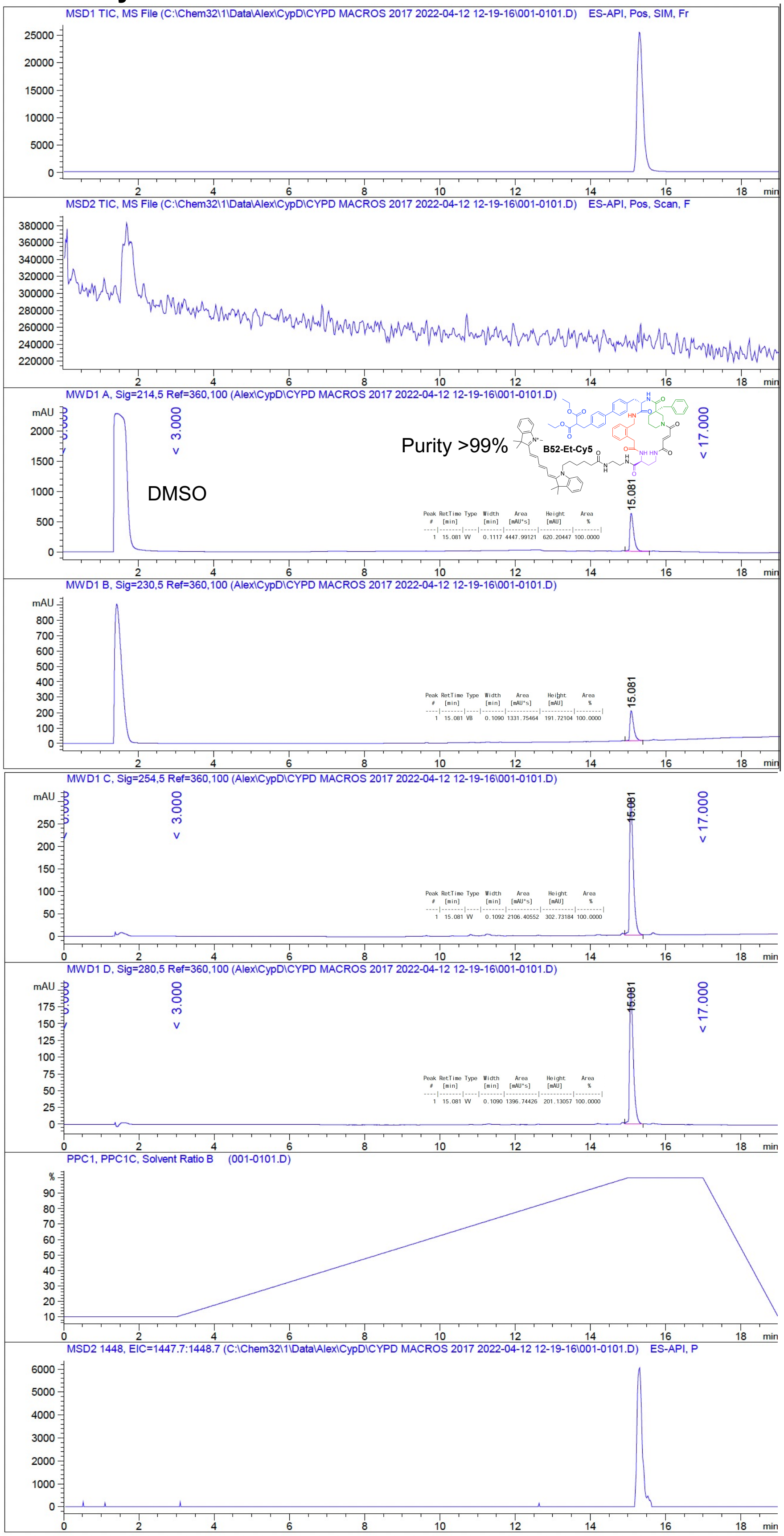
MSD1 TIC, MS File (C:\Chem32\1\Data\Alex\CypD\CYPD MACROS 2017 2022-04-12 12-19-16\001-0101.D) ES-API, Pos, SIM, Fr
MSD2 TIC, MS File (C:\Chem32\1\Data\Alex\CypD\CYPD MACROS 2017 2022-04-12 12-19-16\001-0101.D) ES-API, Pos, Scan, F
MWD1 A, Sig=214,5 Ref=360,100 (Alex\CypD\CYPD MACROS 2017 2022-04-12 12-19-16\001-0101.D)
Purity >99%
B52-Et-Cy5
DMSO
15.081
Peak # | RetTime [min] | Type | Width [min] | Area [mAU*s] | Height [mAU] | Area %
1 | 15.081 | VV | 0.1117 | 4447.99121 | 620.20447 | 100.0000
MWD1 B, Sig=230,5 Ref=360,100 (Alex\CypD\CYPD MACROS 2017 2022-04-12 12-19-16\001-0101.D)
15.081
Peak # | RetTime [min] | Type | Width [min] | Area [mAU*s] | Height [mAU] | Area %
1 | 15.081 | VB | 0.1090 | 1331.75464 | 191.72104 | 100.0000
MWD1 C, Sig=254,5 Ref=360,100 (Alex\CypD\CYPD MACROS 2017 2022-04-12 12-19-16\001-0101.D)
15.081
Peak # | RetTime [min] | Type | Width [min] | Area [mAU*s] | Height [mAU] | Area %
1 | 15.081 | VV | 0.1092 | 2106.40552 | 302.73184 | 100.0000
MWD1 D, Sig=280,5 Ref=360,100 (Alex\CypD\CYPD MACROS 2017 2022-04-12 12-19-16\001-0101.D)
15.081
Peak # | RetTime [min] | Type | Width [min] | Area [mAU*s] | Height [mAU] | Area %
1 | 15.081 | VV | 0.1090 | 1396.74426 | 201.13057 | 100.0000
PPC1, PPC1C, Solvent Ratio B (001-0101.D)
MSD2 1448, EIC=1447.7:1448.7 (C:\Chem32\1\Data\Alex\CypD\CYPD MACROS 2017 2022-04-12 12-19-16\001-0101.D) ES-API, P
min

B53-Et-Cy5

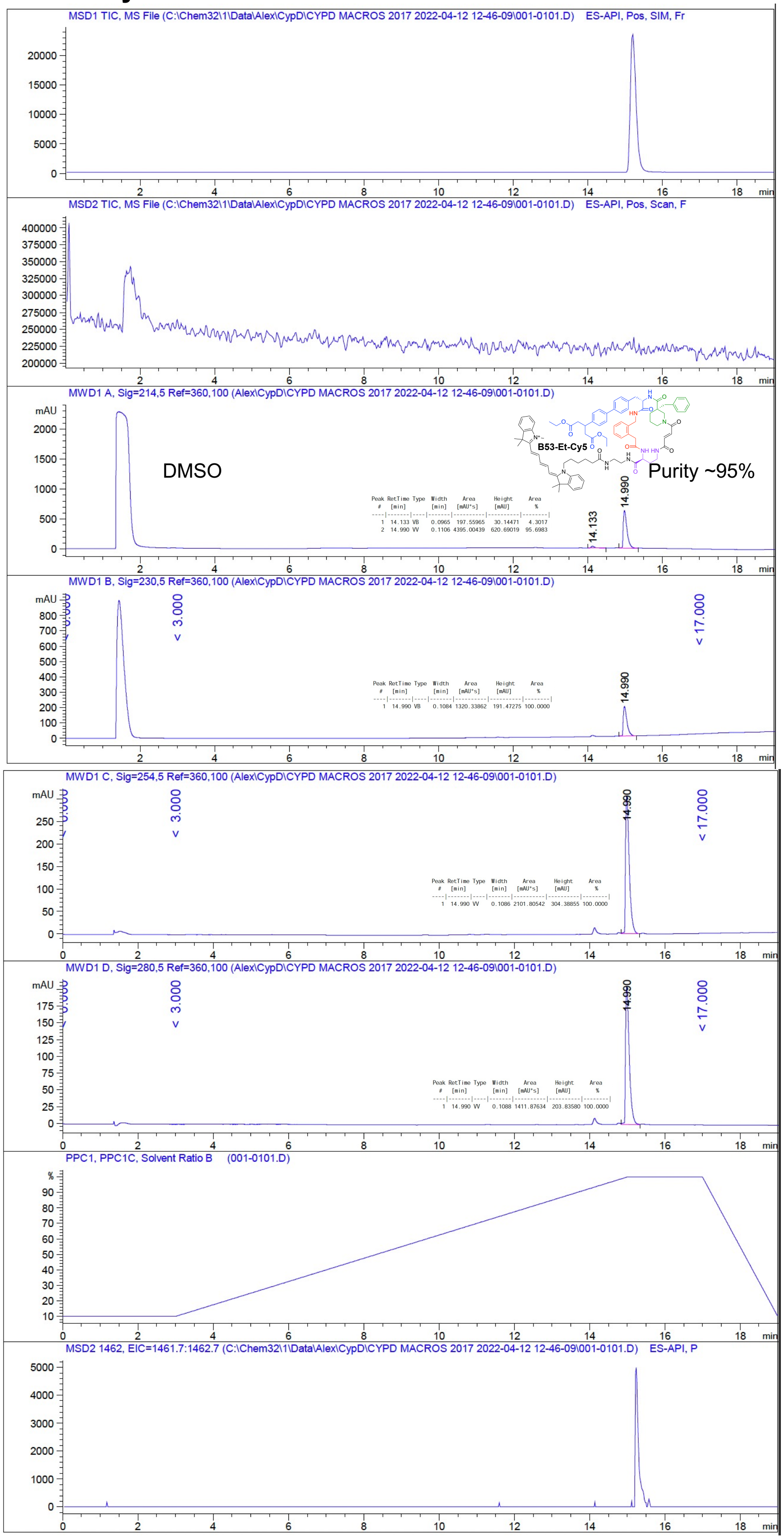
MSD1 TIC, MS File (C:\Chem32\1\Data\Alex\CypD\CYPD MACROS 2017 2022-04-12 12-46-09\001-0101.D) ES-API, Pos, SIM, Fr
MSD2 TIC, MS File (C:\Chem32\1\Data\Alex\CypD\CYPD MACROS 2017 2022-04-12 12-46-09\001-0101.D) ES-API, Pos, Scan, F
MWD1 A, Sig=214,5 Ref=360,100 (Alex\CypD\CYPD MACROS 2017 2022-04-12 12-46-09\001-0101.D)
DMSO
B53-Et-Cy5
Purity ~95%
Peak RetTime Type Width Area Height Area
[min] [min] [mAU*s] [mAU] %
1 14.133 VB 0.0965 197.55965 30.14471 4.3017
2 14.990 VV 0.1106 4395.00439 620.69019 95.6983
14.133
14.990
MWD1 B, Sig=230,5 Ref=360,100 (Alex\CypD\CYPD MACROS 2017 2022-04-12 12-46-09\001-0101.D)
Peak RetTime Type Width Area Height Area
[min] [min] [mAU*s] [mAU] %
1 14.990 VB 0.1084 1320.33862 191.47275 100.0000
MWD1 C, Sig=254,5 Ref=360,100 (Alex\CypD\CYPD MACROS 2017 2022-04-12 12-46-09\001-0101.D)
Peak RetTime Type Width Area Height Area
[min] [min] [mAU*s] [mAU] %
1 14.990 VV 0.1086 2101.80542 304.38855 100.0000
MWD1 D, Sig=280,5 Ref=360,100 (Alex\CypD\CYPD MACROS 2017 2022-04-12 12-46-09\001-0101.D)
Peak RetTime Type Width Area Height Area
[min] [min] [mAU*s] [mAU] %
1 14.990 VV 0.1088 1411.87634 203.83580 100.0000
PPC1, PPC1C, Solvent Ratio B (001-0101.D)
MSD2 1462, EIC=1461.7:1462.7 (C:\Chem32\1\Data\Alex\CypD\CYPD MACROS 2017 2022-04-12 12-46-09\001-0101.D) ES-API, P
min

*B52-Et-Cy5

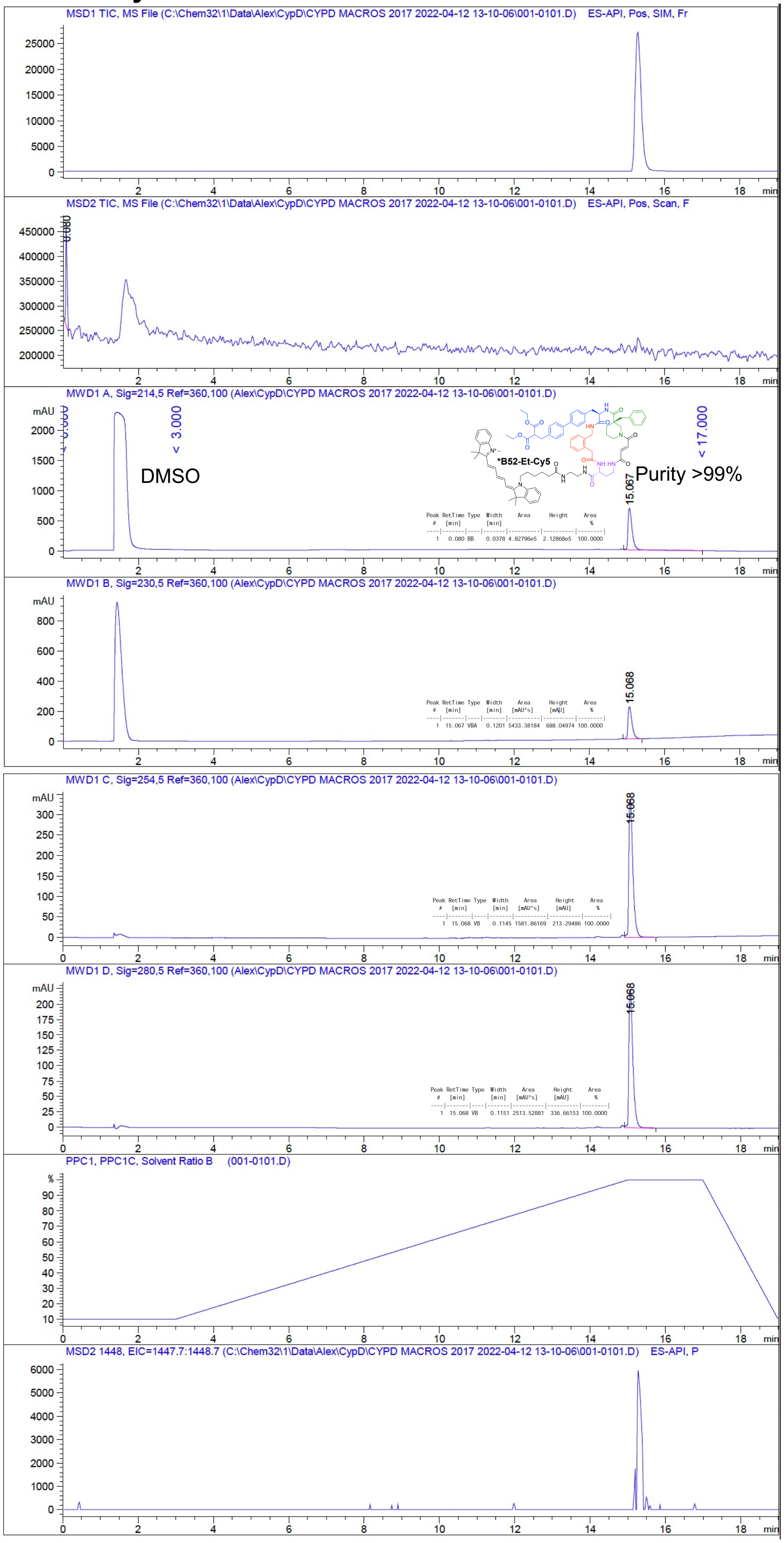
MSD1 TIC, MS File (C:\Chem32\1\Data\Alex\CypD\CYPD MACROS 2017 2022-04-12 13-10-06\001-0101.D) ES-API, Pos, SIM, Fr
MSD2 TIC, MS File (C:\Chem32\1\Data\Alex\CypD\CYPD MACROS 2017 2022-04-12 13-10-06\001-0101.D) ES-API, Pos, Scan, F
MWD1 A, Sig=214,5 Ref=360,100 (Alex\CypD\CYPD MACROS 2017 2022-04-12 13-10-06\001-0101.D)
DMSO
< 3.000
*B52-Et-Cy5
15.067
Purity >99%
< 17.000
MWD1 B, Sig=230,5 Ref=360,100 (Alex\CypD\CYPD MACROS 2017 2022-04-12 13-10-06\001-0101.D)
15.068
MWD1 C, Sig=254,5 Ref=360,100 (Alex\CypD\CYPD MACROS 2017 2022-04-12 13-10-06\001-0101.D)
15.068
MWD1 D, Sig=280,5 Ref=360,100 (Alex\CypD\CYPD MACROS 2017 2022-04-12 13-10-06\001-0101.D)
15.068
PPC1, PPC1C, Solvent Ratio B (001-0101.D)
MSD2 1448, EIC=1447.7:1448.7 (C:\Chem32\1\Data\Alex\CypD\CYPD MACROS 2017 2022-04-12 13-10-06\001-0101.D) ES-API, P
min

*B53-Et-Cy5

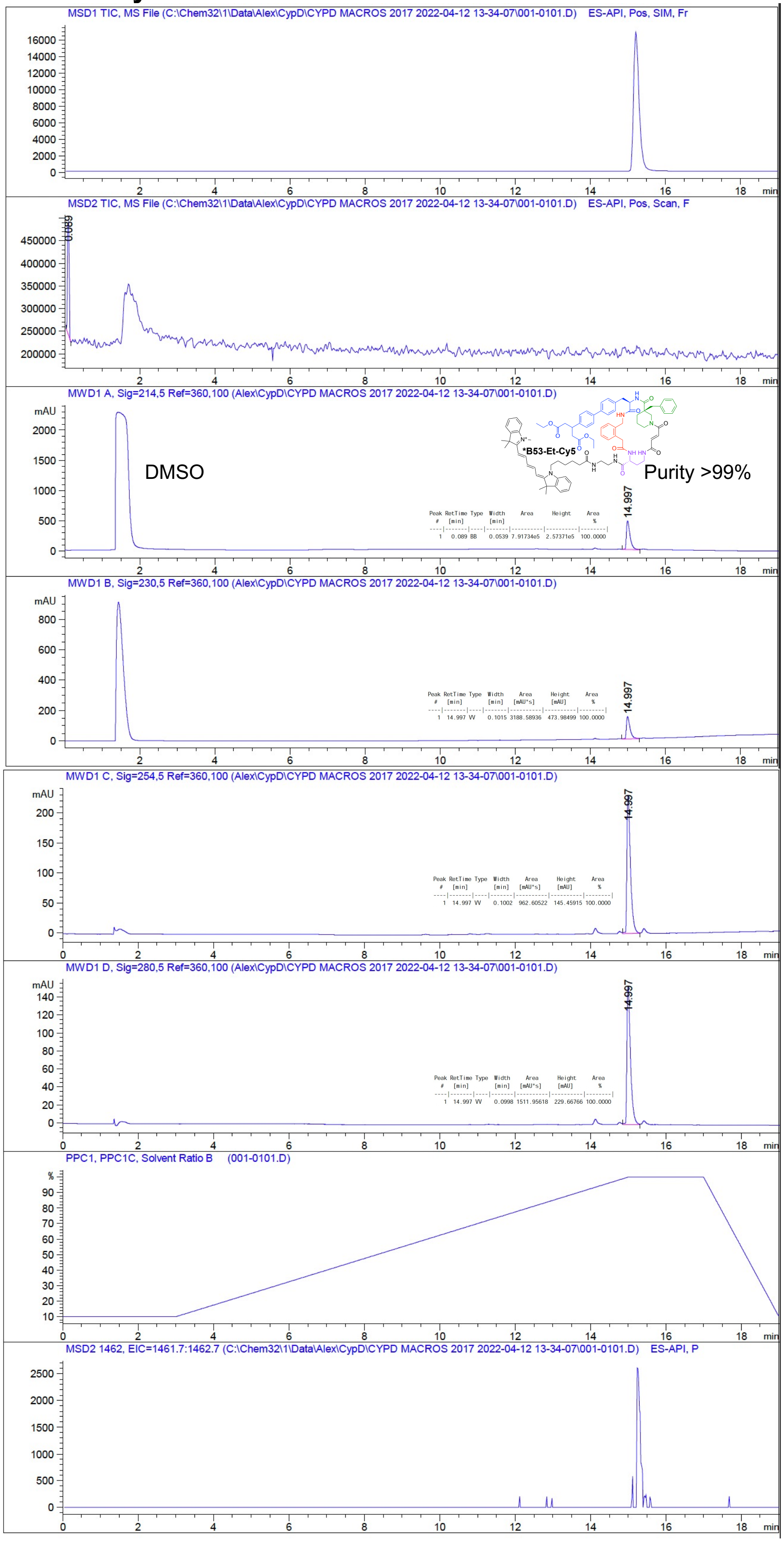
MSD1 TIC, MS File (C:\Chem32\1\Data\Alex\CypD\CYPD MACROS 2017 2022-04-12 13-34-07\001-0101.D) ES-API, Pos, SIM, Fr
MSD2 TIC, MS File (C:\Chem32\1\Data\Alex\CypD\CYPD MACROS 2017 2022-04-12 13-34-07\001-0101.D) ES-API, Pos, Scan, F
MWD1 A, Sig=214,5 Ref=360,100 (Alex\CypD\CYPD MACROS 2017 2022-04-12 13-34-07\001-0101.D)
DMSO
*B53-Et-Cy5
Purity >99%
14.997
MWD1 B, Sig=230,5 Ref=360,100 (Alex\CypD\CYPD MACROS 2017 2022-04-12 13-34-07\001-0101.D)
14.997
MWD1 C, Sig=254,5 Ref=360,100 (Alex\CypD\CYPD MACROS 2017 2022-04-12 13-34-07\001-0101.D)
14.997
MWD1 D, Sig=280,5 Ref=360,100 (Alex\CypD\CYPD MACROS 2017 2022-04-12 13-34-07\001-0101.D)
14.997
PPC1, PPC1C, Solvent Ratio B (001-0101.D)
MSD2 1462, EIC=1461.7:1462.7 (C:\Chem32\1\Data\Alex\CypD\CYPD MACROS 2017 2022-04-12 13-34-07\001-0101.D) ES-API, P
min

Supplementary References

1. Usanov, D. L., Chan, A. I., Maianti, J. P. & Liu, D. R. Second-generation DNA-templated macrocycle libraries for the discovery of bioactive small molecules. *Nat. Chem.* **10**, 1–11 (2018).
2. Qiao, J. X. *et al.* Synthesis of Fmoc-Protected Arylphenylalanines (Bip Derivatives) via Nonaqueous Suzuki-Miyaura Cross-Coupling Reactions. *J. Org. Chem.* **81**, 9499–9506 (2016).
3. Hinkes, S. P. A. & Klein, C. D. P. Virtues of Volatility: A Facile Transesterification Approach to Boronic Acids. *Org. Lett.* **21**, 3048–3052 (2019).
4. Fretz, Heinz; Lyothier, Isabelle; Pothier, Julien; Richard-Bildstein, Sylvia; Sifferlen, Thierry; Wyder Peterson, Lorenza; Pozzi, D. & Corminboeuf, O. N-SUBSTITUTED INDOLE DERIVATIVES AS PGE2 RECEPTOR MODULATORS. (2017). doi:WO 2017/085198A1
5. Yang, D. *et al.* Synthesis and characterization of chiral N-O turns induced by α-aminoxy acids. *J. Org. Chem.* **66**, 7303–7312 (2001).
6. Wilent, J. & Petersen, K. S. Enantioselective desymmetrization of diesters. *J. Org. Chem.* **79**, 2303–2307 (2014).
7. Lunn, D. J. *et al.* Scalable synthesis of an architectural library of well-defined poly(acrylic acid) derivatives: Role of structure on dispersant performance. *J. Polym. Sci. Part A Polym. Chem.* **57**, 716–725 (2019).
8. Quach, D. *et al.* Strategic Design of Catalytic Lysine-Targeting Reversible Covalent BCR-ABL Inhibitors. *Angew. Chemie - Int. Ed.* **60**, 17131–17137 (2021).
9. Crudden, C. M. *et al.* Iterative protecting group-free cross-coupling leading to chiral multiply arylated structures. *Nat. Commun.* **7**, 1–7 (2016).
