# Supplementary material for: Discovery and molecular basis of subtype-selective cyclophilin inhibitors
Source: Nat Chem Biol. 2022 Sep 26;18(11):1184–95. doi: 10.1038/s41589-022-01116-1 (PMC9596378; doi:10.1038/s41589-022-01116-1)
Supplement: Supplementary file 2 — Reporting Summary [file 41589_2022_1116_MOESM2_ESM.pdf]

nature portfolio

Corresponding author(s): David R. Liu and Markus A. Seeliger

Last updated by author(s): YYYY-MM-DD

Reporting Summary

Nature Portfolio wishes to improve the reproducibility of the work that we publish. This form provides structure for consistency and transparency in reporting. For further information on Nature Portfolio policies, see our Editorial Policies and the Editorial Policy Checklist.

Statistics

For all statistical analyses, confirm that the following items are present in the figure legend, table legend, main text, or Methods section.

n/a	Confirmed	
☐	☒	The exact sample size (*n*) for each experimental group/condition, given as a discrete number and unit of measurement
☐	☒	A statement on whether measurements were taken from distinct samples or whether the same sample was measured repeatedly
☐	☒	The statistical test(s) used AND whether they are one- or two-sided<br>*Only common tests should be described solely by name; describe more complex techniques in the Methods section.*
☒	☐	A description of all covariates tested
☒	☐	A description of any assumptions or corrections, such as tests of normality and adjustment for multiple comparisons
☐	☒	A full description of the statistical parameters including central tendency (e.g. means) or other basic estimates (e.g. regression coefficient) AND variation (e.g. standard deviation) or associated estimates of uncertainty (e.g. confidence intervals)
☐	☒	For null hypothesis testing, the test statistic (e.g. *F*, *t*, *r*) with confidence intervals, effect sizes, degrees of freedom and *P* value noted<br>*Give P values as exact values whenever suitable.*
☒	☐	For Bayesian analysis, information on the choice of priors and Markov chain Monte Carlo settings
☒	☐	For hierarchical and complex designs, identification of the appropriate level for tests and full reporting of outcomes
☒	☐	Estimates of effect sizes (e.g. Cohen's *d*, Pearson's *r*), indicating how they were calculated

Our web collection on statistics for biologists contains articles on many of the points above.

Software and code

Policy information about availability of computer code

Data collection	Illumina Miseq Control software v2.6 was used on the Illumina Miseq sequencers to collect high-throughput DNA sequencing data. Fluorescence microscopy data was collected in Harmony 4.9 using a Opera Phenix Plus High-Content Screening System. FLIPR data was collected using Screen Works Peak Pro v4.2.1. Tecan Spark data was collected using SparkControl v3. Surface plasmon resonance data was collected using Biacore T200 control software v3.2. Relative cyclophilin subtype abundances in homo sapiens were collected using paxdb4.1.
Data analysis	UCSF DOCK6.9 was used for molecular footprinting analyses. Crystal structures solved using the Phenix Software Suite (Ver 1.171-3660). Structures analyzed in PyMOL (Ver 2.5.2) and UCSF Chimera (Ver 1.16). Custom Python scripts used for analyzing high-throughput sequencing data is provided in the supplementary information. Fluorescence microscopy was analyzed in Harmony 4.9. Biochemical data analysis was conducted using Prism 9.3.1. Surface plasmon resonance data was analyzed using Biacore T200 control software v3.2 and Prism 9.3.1.

For manuscripts utilizing custom algorithms or software that are central to the research but not yet described in published literature, software must be made available to editors and reviewers. We strongly encourage code deposition in a community repository (e.g. GitHub). See the Nature Portfolio guidelines for submitting code & software for further information.

Data

Policy information about availability of data

All manuscripts must include a data availability statement. This statement should provide the following information, where applicable:

- Accession codes, unique identifiers, or web links for publicly available datasets
- A description of any restrictions on data availability
- For clinical datasets or third party data, please ensure that the statement adheres to our policy

X-ray structures of CypD in complex with macrocycles JOMBt, A26, B1, B2, B3, B21, B23, B25, B52, and B53 are available in the PDB (PDB IDs 7TGS, 7TGT, 7TGU,

7TGV, 7TH1, 7TH6, 7TH7, 7THC, 7THD, and 7THF, respectively). CypD-CsA co-crystal structure was obtained from PDB (2Z6W). High-throughput sequencing data for both replicates of His6-CypD selection using a 256,000 DNA-templated library are available on NCBI's Sequence Read Archive (SRA) website (Accession: PRJNA797008). Human cyclophilin abundance was calculated using paxdb4.1 (https://www.pax-db.org)

Field-specific reporting

Please select the one below that is the best fit for your research. If you are not sure, read the appropriate sections before making your selection.

☒ Life sciences ☐ Behavioural & social sciences ☐ Ecological, evolutionary & environmental sciences

For a reference copy of the document with all sections, see nature.com/documents/nr-reporting-summary-flat.pdf

Life sciences study design

All studies must disclose on these points even when the disclosure is negative.

Sample size	Sample sizes are indicated in figure captions. For experiments with isolated mouse liver mitochondria, sample size was pre-specified at 3 per group due to availability of laboratory animals and no sample size calculation was performed. The pre-specified sample size was sufficient to arrive at statistically significant and reproducible results. Samples sizes were chosen to be 3 or 4 for in vitro biochemical analysis to achieve statistical significance.
Data exclusions	No data were excluded from the analyses.
Replication	All attempts at replication were successful. For all in vitro biochemical analyses, microscopy, and cellular assays, three technical replicates were used, with four independent experiments for key data. For isolated mitochondria experiments, three independent experiments were performed on three independent days and replication was successful.
Randomization	N/A. All experiments were done in vitro.
Blinding	N/A. All experiments were done in vitro.

Reporting for specific materials, systems and methods

We require information from authors about some types of materials, experimental systems and methods used in many studies. Here, indicate whether each material, system or method listed is relevant to your study. If you are not sure if a list item applies to your research, read the appropriate section before selecting a response.

Materials & experimental systems

n/a	Involved in the study	
☒	☐	Antibodies
☐	☒	Eukaryotic cell lines
☒	☐	Palaeontology and archaeology
☐	☒	Animals and other organisms
☒	☐	Human research participants
☒	☐	Clinical data
☒	☐	Dual use research of concern

Methods

n/a	Involved in the study	
☒	☐	ChIP-seq
☒	☐	Flow cytometry
☒	☐	MRI-based neuroimaging

Eukaryotic cell lines

Policy information about cell lines

Cell line source(s)	HEK293T (ATCC CRL-3216), HeLa (ATCC CCL-2), mouse embryonic fibroblasts (MEFs) (ATCC CRL-2991), HepG2 (ATCC HB-8065), and A549 (ATCC CCL-185).
Authentication	Cell lines were authenticated by their suppliers using STR analysis.
Mycoplasma contamination	Cell lines tested negative for mycoplasma.
Commonly misidentified lines (See ICLAC register)	No commonly misidentified cell lines were used in this study.

Animals and other organisms

Policy information about studies involving animals; ARRIVE guidelines recommended for reporting animal research

Laboratory animals	C57Bl6/J mice, female, 10-12 weeks old. Mice were housed in the MGH Animal Research Facility on a 12-hour light/dark cycle with stable temperature (22°C) and humidity (60%).
Wild animals	No wild animals were used in this study
Field-collected samples	No field collected samples were used in this study.
Ethics oversight	Institutional Animal Care and Use Committee at Massachusetts General Hospital.

Note that full information on the approval of the study protocol must also be provided in the manuscript.
